# Synthesis and Structure-Activity Relationship Studies of Hydrazide-Hydrazones as Inhibitors of Laccase from *Trametes versicolor*

**DOI:** 10.3390/molecules25051255

**Published:** 2020-03-10

**Authors:** Halina Maniak, Michał Talma, Konrad Matyja, Anna Trusek, Mirosław Giurg

**Affiliations:** 1 Department of Micro, Nano and Bioprocess Engineering, Faculty of Chemistry, Wroclaw University of Science and Technology, Wybrzeże Wyspiańskiego 27, 50-370 Wrocław, Poland; konrad.matyja@pwr.edu.pl (K.M.); anna.trusek@pwr.edu.pl (A.T.); 2 Department of Bioorganic Chemistry, Faculty of Chemistry, Wroclaw University of Science and Technology, Wybrzeże Wyspiańskiego 27, 50-370 Wrocław, Poland; michal.talma@pwr.edu.pl; 3 Department of Organic and Medicinal Chemistry, Faculty of Chemistry, Wroclaw University of Science and Technology, Wybrzeże Wyspiańskiego 27, 50-370 Wrocław, Poland

**Keywords:** oxidoreductase, hydrazones, salicylaldehyde derivatives, 4-hydroxybenzhydrazide

## Abstract

A series of hydrazide-hydrazones **1–3**, the imine derivatives of hydrazides and aldehydes bearing benzene rings, were screened as inhibitors of laccase from *Trametes versicolor*. Laccase is a copper-containing enzyme which inhibition might prevent or reduce the activity of the plant pathogens that produce it in various biochemical processes. The kinetic and molecular modeling studies were performed and for selected compounds, the docking results were discussed. Seven 4-hydroxybenzhydrazide (4-HBAH) derivatives exhibited micromolar activity *K*_i_ = 24–674 µM with the predicted and desirable competitive type of inhibition. The structure–activity relationship (SAR) analysis revealed that a slim salicylic aldehyde framework had a pivotal role in stabilization of the molecules near the substrate docking site. Furthermore, the presence of phenyl and bulky *tert*-butyl substituents in position 3 in salicylic aldehyde fragment favored strong interaction with the substrate-binding pocket in laccase. Both 3- and 4-HBAH derivatives containing larger 3-*tert*-butyl-5-methyl- or 3,5-di-*tert*-butyl-2-hydroxy-benzylidene unit, did not bind to the active site of laccase and, interestingly, acted as non-competitive (*K*_i_ = 32.0 µM) or uncompetitive (*K*_i_ = 17.9 µM) inhibitors, respectively. From the easily available laccase inhibitors only sodium azide, harmful to environment and non-specific, was over 6 times more active than the above compounds.

## 1. Introduction

The discovery of new eco-friendly pesticides, having readily biodegradable natural units, is an essential need of the agrochemical industry [1]. The global human population is constantly growing, therefore, food production needs to be still enhanced. Synthetic pesticides, which usually act indirectly or non-specifically, cause considerable contamination of the environment. Furthermore, crop losses result from the rapid evolution of pest resistance to these chemicals. These problems lead to a high demand for new cultivation approaches and less toxic plant protection products [2,3]. Commonly occurring and troublesome pathogens of crop plants are filamentous fungi that belong to the *Sclerotniaceae* family, for instance, gray mold (*Botrytis cinerea*) which attacks flowers, green tissues of crop plants [4] and fruits such grapes [5]. The increasing problem is the abundancy of wood-decay fungi that attack young and healthy but also old vintage trees in forestry, fruit orchard culture, horticulture, and in the old parks [6]. Insects e.g., aphids, *Drosophila* species, termites, etc., also cause plant damage which influences the crop production, vitality and aesthetic values of gardens and domestic ornamental plants [7]. Therefore, new natural and natural product-derived plant protection agents with an easily biodegradable structure and specific unique mechanisms of action are highly desirable [8]. A common feature of the fungi and insects mentioned above is the production of laccase, which is specifically involved in various survival and physiological processes [9,10]. White-root fungi, for instance, use laccase to destroy the lignin that is the physical barrier in trees to prevent the degradation of cellulose [6]. Laccases of some molds neutralize phenolic antibiotics, such as phytoanticipins or phytoalexins, produced by the plant immune system as a response to a pathogen attack [11,12]. Laccase together with tyrosinase also play a crucial role in the sclerotia formation and virulence of *Sclerotinia sclerotiorum* [13,14] as well as hardening cuticle in insects [15]. Thus, regulation of the activity of this enzyme might weaken pathogen activity and provide time to strengthen the plant defense system.

**Scheme 1:**
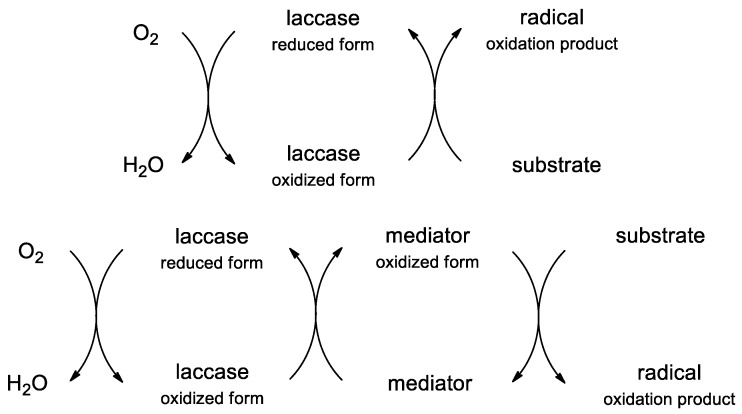
Oxidation mechanism through the laccase and laccase-mediator system (modified from [20]).

Laccase (EC 1.10.3.2) is a copper-containing polyphenol oxidase that is widely distributed in nature. The enzyme catalyzes the radical reduction of atmospheric oxygen to water with simultaneous oxidation of electron-rich aromatic compounds such as polyphenols and anilines [16]. The broad spectrum of substrates that can be converted, mostly by laccases of fungal origin, is due to the high redox potential of a specific copper atom in the enzyme active site [17]. Laccase from *Trametes versicolor* is a typical “blue” oxidase which contains a cluster of four copper atoms categorized as type 1, 2 and 3 [18]. Type 1 is a paramagnetic blue copper and a site of a substrate molecule binding. One copper atom of type 2 and two copper atoms of type 3, form a trinuclear center which binds and reduces dioxygen. The electron transfer from oxidized substrate follows from type 1 Cu center through His458–Cys453–His452 tripeptide to the trinuclear-copper cluster of 2 and 3 types where it is used for the reduction of dioxygen to water molecules [19]. The catalytic cycle includes oxidation of four single-center substrates to four radical products with simultaneous reduction of one molecule of O_2_ to two molecules of H_2_O. The oxidation of a substrate results in the active radicals, which either could be mediators in the radical oxidation reactions or could be non-enzymatically converted to the final oxidation products (Scheme 1).

**Figure 1:**
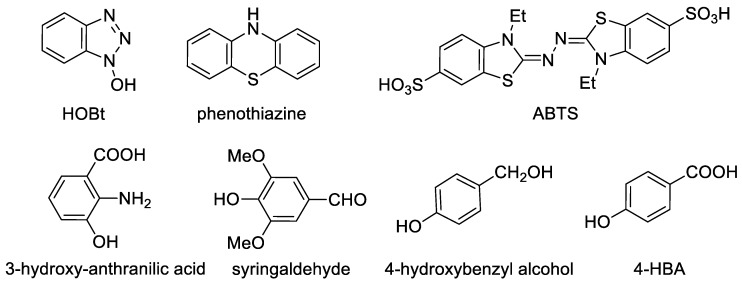
Representative synthetic (upper row) and natural (lower row) laccase mediators (based on [22,24]).

Laccase mediators are low-molecular-weight compounds of great importance in the oxidation of complex high-molecular-weight molecules such as lignin since accessibility to the substrate-binding pocket in the enzyme is spatially limited [20,21]. At least one hundred of these mediators have been described in the literature so far, including the synthetic phenothiazine, 1-hydroxybenzotriazole (HOBt), 2,2′-azino-bis-(3-ethylbenzothiazoline-6-sulfonic acid) (ABTS), or the natural laccase substrates such 3-hydroxyanthranilic acid, 4-formyl-2,6-dimethoxyphenol (syringaldehyde), 4-hydroxybenzyl alcohol and 4-hydroxybenzoic acid (4-HBA) (Figure 1) [20,21,22,23].

Both 4-hydroxybenzyl alcohol and 4-hydroxybenzoic acid (4-HBA) are naturally occurring and are recognized as useful mediators in laccase catalyzed reactions. These simple compounds are also readily available. Moreover, the 4-HBA is only needed in 0.1 mM concentration to significantly increase the oxidation efficacy of resistant compounds in laccase-catalyzed reactions [22]. It seems that such high affinity of the 4-HBA for the substrate pocket in laccase and the presence of an easily chemically modified free carboxyl group would enable us to prepare a collection of 4-HBA derivatives with better fitting to the enzyme cavity and control of their inhibition potency.

Isolation of bioactive natural compounds from plant sources and microorganisms for agricultural applications is usually a challenge due to the small amount of the active ingredient(s), availability of the biomass and cost of the process. Therefore, in our studies, we focused on the use of commonly available natural salicylic aldehydes and carboxylic acids combined with hydrazine linkers to form natural product-derived compounds such hydrazide-hydrazones. Furthermore, we aimed at discovering a new group of organic low-molecular-weight compounds which activity that weakens a pest’s activity and consequently provides conditions for improving the plant defense system. The imines **1–3** were synthesized as part of a program to discover low-molecular-weight compounds that were both the inhibitors of essential enzymes overexpressed during disease development [25,26,27] and act directly against pathogenic microorganisms [28,29]. A common polypore fungus *Trametes versicolor*, which is an abundant tree parasite causing a white-rot of wood, was chosen as a model organism. Infection by *T. versicolor* results in a degradation of lignin by the laccase mediator system, and ultimately to decomposition of the wood structure.

Hydrazide-hydrazones are known as antitumor [30,31,32] and antimicrobial agents [33,34,35], as well as enzyme inhibitors [31,36,37,38,39,40,41]. Hydrazones are less recognized as inhibitors of metallo- enzymes and only the rare examples can be found in the literature so far [38,40,41,42,43]. Therefore, this work provides insight into a relationship between hydrazide-hydrazones and laccase.

## 2. Results and Discussion

### 2.1. Syntheses and Characterizations

The various twenty-three imine derivatives **1–3** were synthesized using 4-hydroxybenzoic acid hydrazide (**4a**) and diverse aldehydes **5–7** having a benzene ring. In two cases, 4-methoxy- and 3-hydroxybenzoic acid hydrazide **4b** and **4c** were used for the preparation of hydrazide-hydrazones **2h** and **3g**, respectively. Among them, fourteen products are new. The hydrazide-hydrazones listed in Table 1 were thus prepared as pure compounds in good to quantitative (76–100%) yields.

Generally, in their Fourier-transform infrared spectroscopy (FT-IR) spectra, the salicylaldehyde derivatives **2a–c** and **3a–b**, which were functionalized in the neighborhood of the hydroxyl group, had a very wide absorption band at 2500–3300 cm^−1^, caused by hydrogen bonds in the crystal lattice.

For all of the remaining hydrazide-hydrazones **1–3**, the wavenumbers corresponding to the stretching vibration of an amide single bond N–H was around 3016–3307 cm^−1^, the carbonyl group (C=O) bands were at 1580–1644 cm^−1^, and the imine bond (C=N) at 1538–1589 cm^−1^.

A sole exception was compound **1b**, in which the aliphatic aldimine C=N group was bound to an ethylene linker that shifted the band a lower wavelength (1507 cm^−1^). The ^1^H-NMR and ^13^C-NMR spectra were measured in DMSO-*d*_6_ except for the insoluble sodium 2-{(*E*)-[2-(4-hydroxy- benzoyl)hydrazinylidene]methyl}benzenesulfonate (**1d**). For this compound, the spectra were measured in fully deuterated CH_3_OH. In the ^1^H-NMR spectra measured in DMSO-*d*_6_, the amide proton (NHCO), phenolic hydroxy group of the 4-HBAH fragment (4–OH) and conjugate imine (CH=N) resonances were observed at ca.: 11.24–12.18, 9.83–10.80 and 8.32–8.93 ppm, respectively. In the ^13^C-NMR spectra, the resonances of the conjugated carbonyl and imine C=O and C=N carbond, and tertiary C-4 phenolic carbon atoms, and ArC-2 were observed at ca.: 161.93–162.80 and 142.19–150.36, 160.43–161.04 and 154.72–160.77 ppm, respectively. For the sulfonic acid sodium salt derivative **1d**, similar chemical shifts were observed in the ^1^H-NMR resonance. The hydrazide-hydrazones which had phenyl or alkyl group near the salicylic hydroxy group caused a weak bathochromic effect. The spectroscopic data were consistent with the data published in the literature [46,47,48,50,51,52,53]. The geometry of the imine CH=N double bond of the aroyl hydrazones has an *E* configuration, which was proved by crystallographic studies of the imine derivatives of 4-hydroxybenzhydrazide with aldehydes such as 2,3-dimethoxybenzaldehyde [50], 5-bromosalicylaldehyde (**2d**) [53] and vanillic aldehyde (**2g**) [52].

**Scheme 2:**
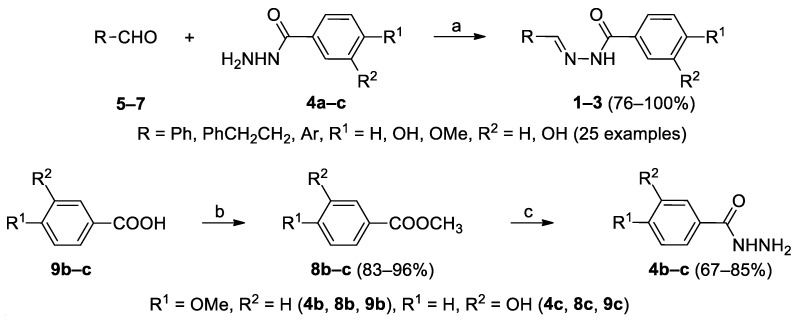
Hydrazide-hydrazones 1a–j, 2a–h, 3a–g, 4-methoxybenzoic acid hydrazide (**4b**), and 3-hydroxybenzoic acid hydrazide (**4c**) preparations. *Reagents and conditions*: a) CH_3_OH, cat. AcOH, reflux, 2–6h, b) CH_3_OH, cat. SOCl_2_, reflux, 20–48h, c) H_2_NNH_2_ × H_2_O, CH_3_OH, reflux, 48h.

Methods of preparation of the hydrazides **4b–c** and aldehydes of choice **6a–b**, **6d–f**, **7a–b**, and **7d–f** were adapted from the original literature [54,55,56,57,58,59,60,61,62,63,64]. 4-Methoxy- and 3-hydroxybenzoic acid hydrazides **4b** and **4c** were prepared in 67–85% yields from the appropriate benzoic acid methyl esters **8b** and **8c** and hydrazine monohydrate following a literature procedure [63]. The suitable benzoic acid methyl esters **8b–c** were obtained in good to excellent (83–96%) yields from benzoic acids **9b–c** through esterification in methanol medium in the presence of added SOCl_2_ (Scheme 2) [65].

**Scheme 3:**
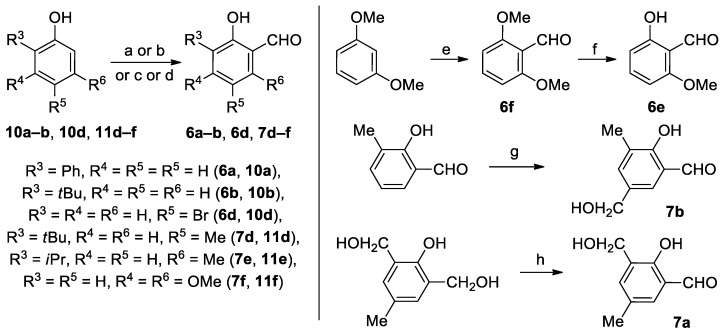
The key aldehydes **6a–b**, **6d–f**, **7a–b**, **7d–f** preparation. *Reagents and conditions*: a) (*i*) NaH, ClMOM, THF, 0 °C; (*ii*) LICTMEDA, DEE, 0 °C, 1h; (*iii*) DMF, −75 °C; (*iv*) HCl, RT, 1h (carried out in accordance with [54]) b) (*i*) *i*PrNCO, cat. DMAP, THF, reflux; (*ii*) TMEDATMSOTf, DEE, RT; (*iii*) LICTMEDA, −78 °C, 1h; (*iv*) DMF, −78 °C; (*v*) NaOH, EtOH, RT; (*vi*) HCl, −78 °C to RT, total 86% (carried out in accordance with [62]), c) (*i*) KOH or NaOH, CHCl_3_, EtOH and/or H_2_O, 60–65 °C; (*ii*) HCl (carried out in accordance with [55,64]), d) (*i*) paraformaldehyde, toluene, cat., SnCl_4_ and (*n*-Bu)_3_N, RT to 100 °C, 8h at 100 °C; (*ii*) HCl aq. (carried out in accordance with [60]), e) (*i*) LICTMEDA, THF, 0 °C; (*ii*) DMF, 0 °C; (*iii*) HCl (carried out in accordance with [58]), f) AlCl_3_, DCM (carried out in accordance with [57]), g) HCHO, H_2_O, 80 °C, 1h (carried out in accordance with [56]), h) MnO_2_, acetone, RT, 6h (carried out in accordance with [59]).

The key 3-phenyl- and 3-*tert*-butyl-salicylaldehydes **6a** and **6b** were obtained in two steps via methoxymethoxy (MOM) protection of the appropriate phenolic compounds **10a** and **10b** with subsequent LICTMEDA *ortho*-lithiation followed by dimethylformamide (DMF) formylation [54]. In the case of 4,6-dimethoxysalicylic aldehyde (**7f**), the two-step synthesis via direct *ortho*-lithiation at position 2 of 3,5-dimethoxyphenol isopropyl carbamate formed in situ from 3,5-dimethoxyphenol, followed by formylation with DMF was adapted from the original literature (Scheme 3) [62].

5-Bromosalicylaldehyde (**6d**) and 3-isopropyl-6-methylsalicylaldehyde (**7e**) were prepared by the Reimer-Tiemann formylation of 4-bromophenol (**10d**) [55] and thymol (**11e**) [64], respectively. 2,6-Dimethoxybenzaldehyde (**6f**) was prepared from resorcinol dimethyl ether by regiospecific LICTMEDA lithiation at position 2, followed by DMF formylation [58]. The selective deprotection of one methoxy group in 2,6-dimethoxybenzaldehyde (**6f**) with AlCl_3_ results in 6-methoxysalicylaldehyde (**6e**) [57]. 3-Hydroxymethyl-5-methylsalicylic aldehyde (**7a**) was obtained by oxidation of one hydroxymethyl group of 2,6-bis(hydroxymethyl)-4-methylphenol with MnO_2_ [59]. In the case of 5-hydroxymethyl-3-methylsalicylic aldehyde (**7b**), 3-methylsalicylic aldehyde underwent hydroxymethylation with formaldehyde in the *para* position to the free phenolic hydroxy group [56]. 3-*tert*-Butyl-5-methylsalicylaldehyde (**7d**) was obtained by monoformylation of 2-*tert*-butyl-4-methylphenol (**11d**) at position six with 2 molar equivalents of paraformaldehyde in the presence of a catalytic amount of SnCl_4_ and tri-*n*-butylamine additives [60].

### 2.2. Kinetic Studies

The studies of laccase inhibitors were fully reviewed [66]. Factors affecting laccase activity were identified as the following: heavy metals, chelating agents, halides, ionic strength, organic solvents, and surfactants. Inorganic species such as hydrogen peroxide [67], bases [68], and organic inhibitors such as dipeptides [69], β-mercaptoethanol [70] as well as hardly available natural compounds such high-molecular-weight humic acids [71], ptilomycalin A [72], and medicarpin [73,74] complete the above list. To the best of our knowledge, only the semi-synthetic hydrazide-hydrazone ether derivative of coumarin and gentisaldehyde (2,5-dihydroxybenzaldehyde) [41] is known to inhibit laccase, with a mixed type of inhibition (*K*_iu_ = 35 µM and *K*_ic_ = 490 µM).

Concerning the aspects of aldehyde substituents, the tested aldimine derivatives were formally divided into three groups (Table 2). The first group contained the derivatives of benzaldehyde (**1a**), phenylethyl aldehyde (**1b**), and benzaldehydes **1c–j** having one Me, OH, OMe, and SO_3_Na substituent on the phenyl ring at different positions. The second group comprised eight aldimine derivatives of monosubstituted salicylic aldehydes (**2a–h**). Seven compounds were imine derivatives of 4-hydroxybenzoic acid hydrazide (**4a**) with the following aldehydes: vanillin aldehyde (**6g**), 2,6-dimethoxybenzaldehyde (**6f**) and salicylaldehyde, having one Ph, *t*Bu, OH, Br and OMe substituents (compounds **6a–e**). The last aldimine was a derivative of 3-phenylsalicylaldehyde (**6a**) with 4-methoxybenzoic acid hydrazide (**4b**). The third group contained the six imines **3a–f**. These are derivatives of hydrazide **4a** with the disubstituted salicylaldehydes **7a–f** containing two identical or different CH_2_OH, Me, *t*Bu, *i*Pr or OMe substituents. All twenty-five selected hydrazide-hydrazones mentioned above and 4-hydroxybutyl acrylate (4-HBA), 4-HBAH were used in kinetic experiments. The reference sodium azide (positive control) was chosen as a well-known inorganic laccase inhibitor.

Determination of the kinetic parameters for laccase and inhibition constants (*K*_i_) for the tested hydrazide-hydrazones was performed in aqueous or aqueous-organic solution at 25 °C and pH 5.3. An organic solvent, CH_3_OH or dimethyl sulfoxide (DMSO), in 6.25% *v/v* concentration was necessary to enhance the solubility of the tested compounds since the experiments in buffer solution gave inaccurate results. At such concentrations, methanol influenced laccase stability to a negligible extent when compared to DMSO solvent, which significantly decreased the activity of the enzyme [75] (see Supplementary Materials). The kinetic parameters in 6.25% *v/v* of methanol solution were estimated using Michaels-Menten model [76] as follows: *K*_m_ = 4.276 µM, *k*_3_ = 4.343 µmol/min/mg of protein, the coefficients for the goodness of fit were R^2^: 0.9745, sum of squared errors(SSE): 1.6767, root mean squared error (RMSE): 0.0984. Fifteen derivatives showed no effect on the tested oxidoreductase for an arbitrarily assumed criterion of *K*_i_ ≥ 1000 µM. Eleven compounds had inhibition potency at the level of *K*_i_ values between 939 µM and 17.9 µM. The predominant mode of interaction of 4-HBAH derivatives **1c**, **1e**, **1i**, **2a–b**, **2g**, **3d** with laccase was the competitive type of inhibition. The hydrazide-hydrazones of the first group containing the only one biogenic substituent in different positions of aldehyde fragment (Table 2) appeared to be weak or moderate inhibitors. Therefore, for these compounds, the enzyme activity was measured for an extended range of *K*_i_ concentrations (up to 2400 µM).

Among the most interesting potent oxa substituents, the inhibition constants ranged between 416 µM and 674 µM for hydroxy and methoxy groups localized at the *ortho* and *para* or *meta* position for **1e**, **1g**, and **1i**, respectively. Interestingly, among alkylated ones, the best *K*_i_ = 251 µM was observed for compound **1c** having a small methyl substituent at position 4 of the benzylidene unit. This is in agreement with general trends for enzymes for which alkyl groups provided better filling of the hydrophobic area in substrate binding pocket [77]. Consequently, for further studies, generally, we concentrated on C or O alkylated the 2- and/or 4-hydroxy- and/or 3-methoxy- benzaldehyde derivatives **2a–h** and **3a–g**.

In the second group, the combination of the hydroxy and methoxy substituents, in the *para* and *meta* positions of vanillic aldehyde, resulted in an approximately two-fold improvement in the *K*_i_ value to 251 µM for compound **2g** compared to related monosubstituted benzaldehyde derivatives **1g** and **1i**. This compound is a derivative of naturally occurring vanillic aldehyde, commonly found in vegetation. Therefore, the constant inhibition and biodegradable scaffold components of vanillic derivative **2g** seemed acceptable for the protection of plants, especially ornamental plants. An opposite effect to the derivative **2g** was observed for isomer **2e** when the hydroxy and methoxy groups were present in the *ortho* and *ortho*’ positions in the benzaldehyde framework. Similarly, the pairs of two methoxy and two hydroxy groups in the *ortho*, *ortho*’ and *ortho, para* positions in **2f** and **2c**, respectively, were practically inactive towards laccase. The hydrazide-hydrazone derivatives of 2,5-dihydroxybenzaldehyde (gensinealdehyde) acted as laccase inhibitors when they were linked to coumarin with an ether bond [34]. In our studies, the hydrazide-hydrazone derivative of this aldehyde with 4-HBAH displayed intense pigmentation. Furthermore, the reaction results were inconclusive (unpublished data) because gensinaldehyde as the hydroquinone was known to be a good laccase substrate. Generally, *O*-methylation of phenolic hydroxy groups in the Ar-2-OH, Ar-4-OH or 4-OH compounds **1e**, **1g**, and **2a** to **1h**, **1j**, and **2h**, respectively, caused the loss of inhibition potency (*K*_i_ ≥ 1000 µM). Interestingly, the introduction of phenyl and *tert*-butyl substituents at position 3 of salicylic aldehyde fragments, in the hydrazide-hydrazones **2a** and **2b**, provided the expected competitive type of inhibition with *K*_i_ values of 25.3 µM and 24.0 µM, respectively (Table 2).

In the third group, the salicylic aldehyde fragment was modified with alkyls on purpose to provide better interaction with the hydrophobic area of the enzyme-substrate cavity (Table 2). The introduction of hydroxymethyl and hydroxy groups in positions 3 and 5 in the aldehyde framework of **3a** or conversely in positions 5 and 3 of **3b** caused a total decrease in the activity with *K*_i_ ≥ 1000 µM. Even the presence of the smallest alkyl unit, the methyl group, at position 6 and an isopropyl group in position 3 in **3e** compound, was also not desirable. On the other hand, the transfer of the methyl group to position 5, in a structurally similar molecule, **3d**, maintained *K*_i_ = 26.4 µM at a comparable level to **2a** and **2b**. Analogous modification in position 5 with the bulky *tert*-butyl group, in the case of hydrazide-hydrazone **3c**, resulted in improvement to the best *K*_i_ value to 17.9 µM with a simultaneous change in the enzyme-inhibitor interaction. Finally, the transfer of the hydroxy group to the *meta* position in the hydrazide decoy fragment in **3g**, in line with our expectations, changed the mechanism of action to non-competitive with quite good *K*_i_ = 32.0 µM.

**Figure 2:**
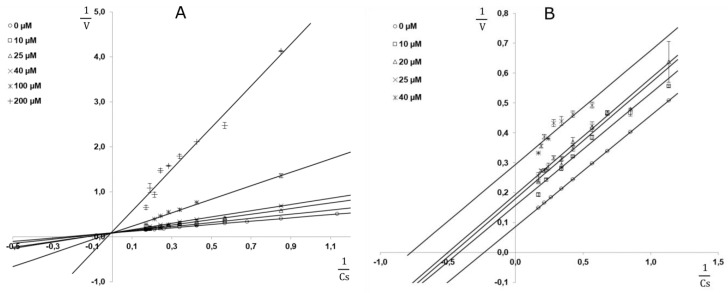
The representative Lineweaver-Burk plots 1/V [min µmol^−1^] = f(1/C_S_) [µM^–1^] for laccase from *Trametes versicolor* in the presence of hydrazide-hydrazone **2b** (**A**) and hydrazide-hydrazone **3c** (**B**) tested in the range of concentrations 10–200 µM and 10–40 µM, respectively.

The representative Lineweaver-Burk plots for laccase from *T. versicolor* in the presence of competitive **2b** and uncompetitive **2c** inhibitors are depicted in Figure 2. Further results of our research, that is focused on naturally-derived arenecarboxylic acid hydrazide-hydrazones, will be the subject of our future publications.

### 2.3. Structure-Activity Relationship (SAR)

**Figure 3:**
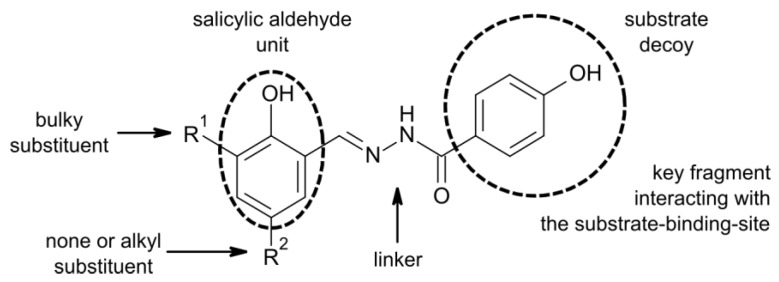
The preferential structure of hydrazide-hydrazones as inhibitors of laccase from *T. versicolor*.

The resulting hydrazide-hydrazones contain two terminal benzene units combined with a hydrazide-hydrazone linker (–(CO)–NH–N=CH–). The compounds act as aza derivatives of naturally occurring 4-hydroxybenzoic acid (4-HBA) or related arenecarboxylic acids which aromatic fragments were found in plants, e.g., in the lignin structural units such as *para*-coumaryl, coniferyl, and sinapyl alcohols [78]. 4-HBA is also a mediator of laccase [22]. Furthermore, this acid presented in a hydrazide fragment is a naturally occurring motif found in the shikimate biosynthesis pathway. Thus, this part of the molecule was a natural substrate for laccase and serves as a decoy. The second terminal aromatic ring might act similarly, especially if it contains a hydroxy group. The amide group in the linker connected with an azomethine group with a nitrogen–nitrogen single bond (–(CO)–NH–N=CH–) is known to form coordination bonds with metals, i.e., copper cations [79,80,81]. We assumed that these three elements would provide the necessary interactions with the laccase substrate-binding pocket. As presented in Figure 3, all highly active compounds contained a mono- or disubstituted salicylic aldehyde framework. Single *tert*-butyl or phenyl substituenta (R^1^) located directly at position 3 show *K*_i_ = 24–25 µM, while disubstituted salicylic aldehydes having a *tert*-butyl R^1^ and methyl or a second *tert*-butyl substituents R^2^ at positions 3 and 5, respectively, provided a comparable inhibition effect with *K*_i_ = 18–26 µM.

The compounds 4-HBAH and 4-HBA had no inhibitory potency towards the target protein. The results were in agreement with the literature data in which the 4-HBA was tested as a natural mediator in laccase-catalyzed reactions [22]. To the best of our knowledge, only humic acids are proved to be potent natural inhibitors of laccase from *Panus tigrinus*, with an inhibition constant *K*_i_ = 3–25 mg/dm^3^ (*K*_i_ = 0.23–1.62 µM) [71].

Unfortunately, these soil acids were a mixture of high molecular organic compounds with a variable composition depending on the organic matter they originated from [82]. Also, the mechanism of action was not specific since humic acids have been found recently to be polymerized efficiently by laccase from *Streptomyces anulatus* [83]. A guanidinium alkaloid isolated from marine organisms, ptilomycalin A, is another example of an organic laccase inhibitor reported in the literature so far [72]. Just like the aforementioned humic acids, they seem unsuitable for the needs of the agricultural industry. The azide ion is a well-known and the most potent inorganic inhibitor of laccases. Furthermore, it is not specific only for laccase, and its inhibition efficacy for other metalloenzymes is generally recognized [84]. Therefore, it was used in our study as a positive reference compound. Until now, the *K*_i_ values and mechanism of inhibition were determined simultaneously for laccase from two *Pleurotus* species using ABTS as substrate. The laccase from *Pleurotus eryngii* was inhibited by azide ion via a mixed type of inhibition with *K*_ic_ = 17.6 µM and *K*_iu_ = 10.6 µM constants [85]. The latter studies performed by Patel and co-workers for laccase from closely related species, *Pleurotus ostreatus*, showed a similar value of constant inhibition *K*_i_ = 16.5 µM, nonetheless, with a completely different non-competitive type of inhibition [86]. Taking into consideration a discrepancy between these mechanisms of inhibition for NaN_3_ determined by using ABTS, we carried out measurements with syringaldazine. For this substrate, we obtained data similar to the latter [86], a non-competitive mechanism of inhibition towards laccase from *Trametes versicolor*. The determined constant is six-fold higher (Table 2) than the corresponding *K*_i_ value measured for laccase from *Pleurotus ostreatus*.

### 2.4. The Docking Studies

The crystal structures of laccases are widely available in databases. Therefore, the choice of high-resolution structure allowed for careful consideration of the interaction of inhibitors with the enzyme. The laccase from *Trametes versicolor* PDB: 1GYC [87] was used for SAR studies.

**Figure 4:**
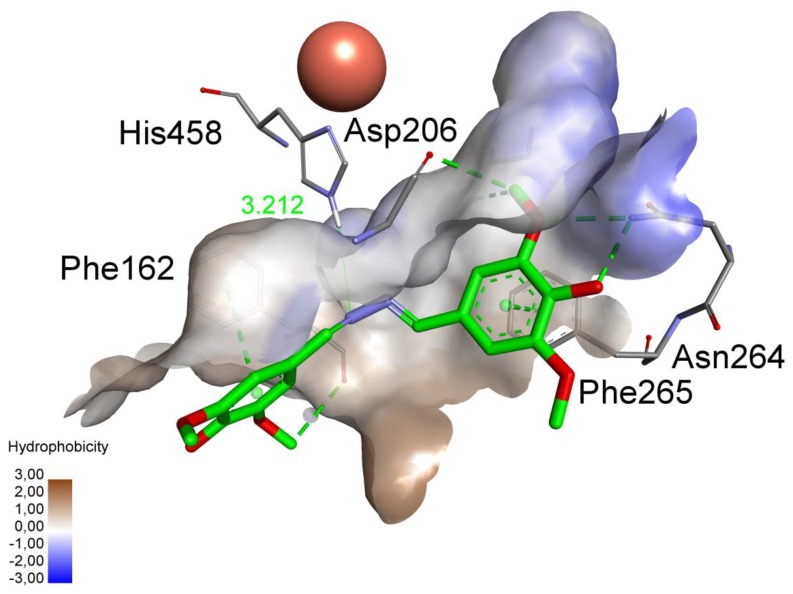
Structure of laccase enzyme from Trametes versicolor (PDB: 1GYC) with the syringaldazine in the active substrate centre. The interactions are shown as green dashed lines. The distance of the ligand to His-458 is 3.212 Å and is shown as regular green line. The copper ion (type 1) is shown as an orange sphere.

The inhibitors and syringaldazine were docked in the active sites of laccase. It is known that the substrates take part in electron transfer by the interactions with at least one of the following amino acids: Asp206, Asn264 and His458 [88]. The histidine stabilizes one of the copper ions catalyzing the reaction (Figure 4).

**Figure 5:**
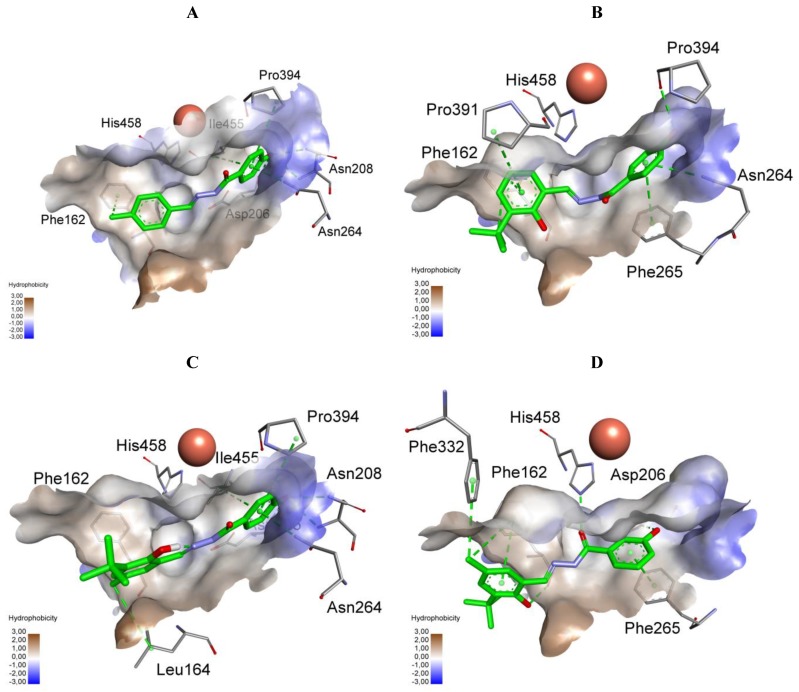
The representation of 1c (**A**), 2b (**B**), 3d (**C**), 3g (**D**) compounds interactions in the active site of laccase from Trametes versicolor (PDB: 1GYC). The copper ions are shown as orange spheres. A surface of the enzyme (**A**–**D**) represents the hydrophobicity of nearby amino acid side chains.

The alkylated hydrazide-hydrazones 1c, 2b, and 3d presented a competitive type of inhibition which was determined experimentally (Table 2). The similarity in their structures might allow interactions with the amino acids in the active centre (Figure 5A–C) affecting the measured inhibition constant values. The substrate cavity was divided into two parts. The first was a deep pocket made of amino acids readily forming hydrogen bonds with Asp206, Asn208, Asn264; but also those stabilizing the position of the aromatic ring, characteristic for the substrate: Phe265, Pro394, Ile455. The second part had a completely different, hydrophobic character with an ample space likely to pre-capture the substrate. The methylated compound 1c (Figure 5A) fitted this system perfectly. However, the inhibition constant was beneficially lowered for molecule 3d by the following changes: the addition of a hydroxy group near the imine group, the change of the position of the methyl group and the addition of the second lipophilic substituent near the hydroxy group (Figure 5C). We thought that Leu164 might play a significant role, but the removal of the methyl group did not change the *K*_i_ value markedly. The inhibitor 2b (Figure 5B) interacted with amino acid Pro391 by positioning the aromatic ring similar to Leu164. Therefore, we concluded that beneficial changes could be caused by increasing the hydrophobicity of the aldehyde ring and possible OH···N intramolecular interactions that stiffened the structure.

**Figure 6:**
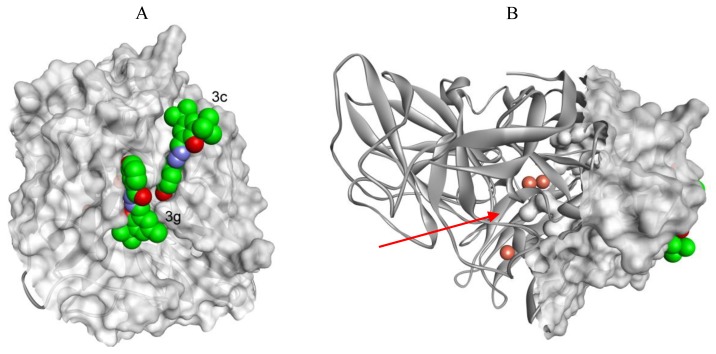
Interaction of hydrazide-hydrazones **3c** and **3g** on the surface of laccase (PDB: 1GYC): (**A**). The red arrow indicates the oxygen tunnel (**B**); the copper ions (type 2 and 3) are shown as orange spheres.

Another case concerns compound 3g, which showed non-competitive inhibition. It could bind to the enzyme at the substrate centre, at any allosteric centre, or also to the enzyme-substrate complex. Its behaviour might mimic the natural ligand (Figure 5D). Although it did not bind in precisely the same way as syringaldazine, as it interacted with His458 as well as the carbonyl oxygen atom as the H-bond acceptor. For compounds 3g and 3c, we also conducted theoretical studies of binding at other places on the enzyme surface (Figure 6). The position of the inhibitors near the oxygen-binding site worked like a cork (Figure 6A). Access to the copper ions was blocked. However, the position in a different place on the surface was preferable. Comparing the energy of the binding for near the oxygen surface to the energy of the binding to unspecific position on the surface for 3c (−33.93 kcal/mol vs −38.91 kcal/mol) and 3g (−36.21 kcal/mol vs −44.51 kcal/mol) revealed that their affinity to a non-oxygen cavity was higher. The oxygen tunnel did not change the original form then (Figure 6B).

## 3. Materials and Methods

### 3.1. Reagents and Materials

Chemicals and solvents were purchased as pure “for synthesis” or “analytical grade” reagents from Sigma-Aldrich (St. Louis, MO, USA), ARMAR (Döttingen, Switzerland), and POCh (Gliwice, Poland) and were used mostly without further purification. In particular, 4-hydroxybenzhydrazide (**4a**—4-HBAH) (≥97%, Fluka, Hamburg, Germany) syringaldazine (SNG, 4-hydroxy-3,5-dimethoxy- benzaldehyde azine) (Sigma-Aldrich), dimethylsulfoxide (DMSO) (Sigma-Aldrich, 99.5%, (GC) suitable for plant cell culture), 2,6-bis(hydroxymethyl)-4-methylbenzene (Sigma-Aldrich). Citric acid monohydrate, and sodium phosphate dibasic dodecahydrate were purchased in Avantor (Gliwice, Poland), and were used without further purification. Laccase from *Trametes versicolor* was purchased as a lyophilized powder (Sigma-Aldrich).

Diethyl ether (DEE) was distilled slowly over a mixture of LiAlH_4_ and CaH_2_ powders using a water bath. Methyl alcohol (CH_3_OH) was distilled prior to condensation reactions from Mg shavings in the presence of I_2_. Hydrazide-hydrazones **1–3** were prepared by condensation of an equimolar mixture of an appropriate carboxylic acid hydrazide **4a–c** with the appropriate aldehyde **5–7** in CH_3_OH in the presence of a catalytic amount of AcOH, as described below. Analytical TLC was performed on PET foils precoated with silica gel (silica gel 60 F254, Merck, Sigma-Aldrich, Saint Louis, MO, USA), visualized under UV light (λ_max_ = 254 nm), or by staining with iodine vapor. Melting points (m.p.) were determined on an IA 91100 digital melting-point apparatus (Electrothermal, Sigma-Aldrich, Saint Louis, MO, USA) using the standard open capillary method. FT-IR spectra (4000–400 cm^−1^) were recorded as KBr plates on a 2000 FT-IR (Perkin–Elmer, Manchester, UK) or a VERTEX 70V spectrometer (Bruker, Ettlingen Germany) using a diamond attenuated total reflectance (ATR) accessory. Absorption maxima are reported in wavenumbers (cm^−1^). ^1^H- and ^13^C-NMR spectra (399.78 for ^1^H and 100.52 for ^13^C) were recorded on a 400YH (JEOL, Tokyo, Japan) or an Avance 600 Spectrometer (600.58 for ^1^H and 151.03 for ^13^C, Bruker, Poznań, Poland) at 295 K. Chemical shifts (δ) are given in parts per million (ppm) downfield relative to TMS, and coupling constants (*J*) are in Hz. Residual solvent central signals were recorded as follows: DMSO-*d*_6_, δ_H_ = 2.50, δ_C_ = 39.43; CH_3_OH-*d*_4_, δ_H_ = 3.31, δ_C_ = 49.05; CDCl_3_, δ_H_ = 7.263, δ_C_ = 77.00. Signals of DEPT experiment were referred to (+) or (−), when measured. High-resolution mass spectra (HRMS) were recorded on a LCD Premier XE instrument (Waters, Manchester, UK) and only the [M + H]^+^ or [M + Na]^+^ molecular species are reported. The literature procedure was adapted for the preparation of benzoic acid hydrazide **4b–c** [63], aldehydes **6a** [54], **6b** [54], **6d** [55], **6e** [58], **6f** [58], **7a** [59], **7b** [56], **7d** [60,61], **7e** [64], **7f** [62], and benzoic acid methyl ester **8b–c** [65] (see Section 4.1.1. of [65]). Purity and homogeneity of known compounds were confirmed by measuring their m.p. for **1a** [44], **1c** [46], **1e** [47], **1f** [48], **1g** [49], **1h–j** [50], **2d** [49], **2g** [51], **4b** [89], **4c** [90], **6a** [60], **6d** [91], **6e** [57], **6f** [92], **7a** [93], **7b** [56], **7d** [61], **7f** [62], **8b** [94], and **8c** [95], or boiling points for **6b** [96], **7e** [97], FT-IR spectra for **1a** [51], **1g** [52], **1h–i** [50], **2g** [51], ^1^H- and ^13^C-NMR spectra for **1c** [46], **1e** [47], **1f** [48], **1g** [46], **1h–j** [50], **2c** [48], **2d** [53] and/or HRMS for **2c** [48], and comparing them with literature data. All new hydrazide-hydrazones **1b**, **1d**, **2a–b**, **2e–f**, **2h**, **3a–g**, were fully characterized. The hydrogen and carbon atoms positions in the NMR spectra were supported by the standard dept-135 or ATP experiments and by map analysis of the Heteronuclear Multiple-Quantum Correlation (2D HMQC), Heteronuclear Multiple Bond Correlation (HMBC), Nuclear Overhauser Enhancement Spectroscopy (NOESY) experiments, is measured (see Supplementary Materials).

### 3.2. Syntheses

#### General procedure for the Synthesis of Hydrazide-Hydrazones **1a–j, 2a–h, 3a–g**

To a mixture of aldehyde **5–7** (2.0 mmol), and carboxylic acid hydrazide **4a–c** (2.0 mmol) in dry CH_3_OH (5.0 mL), AcOH (0–200 µL) was added at room temperature (RT) and then the resulting mixture was gently refluxed under stirring. The reaction progress was monitored by TLC. When the reaction was finished, after slow cooling to RT and further cooling to ca. 4 °C, the reaction mixture was left overnight in a refrigerator (−24 °C). The crystals formed were collected by filtration to give pure products **1–3** which were identified by comparison of their melting points and FT-IR and/or NMR spectra with literature data and/or by HRMS measurements. The new compounds **1b**, **1d**, **2a–b**, **2e–f**, **2h**, and **3a–g**, were fully characterized.

*4-Hydroxy-N’-[(E)-benzylidene]benzohydrazide* (**1a**). The general procedure starting from benzaldehyde (**5a**, 212 mg, 2.0 mmol), 4-hydroxybenzohydrazide (**4b**, 304 mg, 2.0 mmol), CH_3_OH (5.0 mL), and AcOH (100 µL) was employed with a 2 h reaction time, and solvent was slowly distilled to a volume of cas 2.5 mL before crystallization to obtain hydrazide-hydrazone **1a**. Colorless crystals; 462 mg, 1.92 mmol, 96% yield; m.p. 242–244 °C (from CH_3_OH) (240–242 °C [44]); selected FT-IR (ATR) ν_max_/cm^−1^: 3217 (O-H, N-H), 3056 (C-H), 3039 (C-H), 3027 (C-H), 1614 (C=O), 1588 (C=C), 1548 (CH=N), 1509, 1356, 1280 (C-O), 1212, 1172, 1110, 1056, 960, 844, 763, 661, 630, 614, 510, 492; ^1^H-NMR (DMSO-*d*_6_, 400 MHz): δ 11.64 (s, 1H, NHCO), 10.13 (s, 1H, OH), 8.43 (s, 1H, CH=N), 7.81 (d, ^3^*J* = 8.7 Hz, 2H, H-2,6), 7.71 (d, ^3^*J* = 6.4 Hz, 2H, ArH-2,6), 7.38–7.48 (m, 3H, ArH-3,4,5), 6.87 (d, ^3^*J* = 8.7 Hz, 2H, H-3,5) ppm; ^13^C-NMR (DMSO-*d*_6_, 100 MHz): δ 162.77 (C=O), 160.66 (C-4), 146.82 (CH=N), 130.47 (ArC-1), 129.79 (ArC-4), 129.65 (C-2,6), 128.75 (ArC-3,5), 126.90 (ArC-2,6), 123.81 (C-1), 114.98 (C-3,5) ppm; HRMS (Time-of-Flight (TOF), Mass spectrometry (MS), Electrospray ionization (ESI)) calculated for C_14_H_12_N_2_O_2_ [*M* + Na]^+^ *m/z* 263.0786, found 263.0806.

*4-Hydroxy-N’-[(E)-3-phenylpropylidene]benzohydrazide* (**1b**) [45]. The general procedure starting from 3-phenylpropionaldehyde (**5b**, 805 mg, 6.0 mmol), 4-hydroxybenzohydrazide (**4b**, 716 mg, 5.0 mmol), CH_3_OH (6.0 mL), and AcOH (100 µL) was employed with a 6 h reaction time. H_2_O (3.0 mL) was added with vigorous stirring at water-ice bath temperature (+4 °C) to induce precipitate formation, and the mixture was left in a refrigerator (−24 °C) overnight for crystallization to obtain predominantly the hydrazide-hydrazone **1b**. Colorless powder; 960 mg, 3.58 mmol, 72% yield; m.p. 243.5–244.0 °C (from CH_3_OH); selected FT-IR (ATR) ν_max_/cm^–1^: 3238 (O-H), 3159 (N-H), 3029 (C-H), 2948 (C-H), 2925 (C-H), 2857 (C-H), 1626 (C=C), 1599 (C=O), 1537, 1507 (CH=N), 144, 1364, 1279 (C-O), 1240, 1172, 848, 753, 701, 618, 515; ^1^H-NMR (DMSO-*d*_6_, 400 MHz): δ 11.24 (s, 1H, NHCO), 10.07 (s, 1H, OH), 6.68–7.77 (m, 3H, CH=N, H-2,6), 7.23–7.33 (m, 4H, ArH-2,3,5,6), 7.19 (tt, ^3^*J* = 6.9 Hz, ^4^*J* = 1.8 Hz, 1H, ArH-4), 6.82 (d, ^3^*J* = 8.6 Hz, 2H, H-3,5), 2.81 (t, ^3^*J* = 7.6 Hz, 2H, PhCH_2_), 2.56 (td, ^3^*J* = 7.6 Hz, ^3^*J* = 5.1 Hz, 2H, CH_2_CH=N) ppm; ^13^C-NMR (DMSO-*d*_6_, 100 MHz): δ 162.54 (C=O), 160.49 (C-4), 150.36 (CH=N), 140.95 (PhC-1), 129.51 (C-2,6), 128.38 (ArC-2,6), 128.34 (ArC-3,5), 125.94 (PhC-4), 123.91 (C-1), 114.91 (C-3,5), 33.77 (CH_2_CH=N), 32.07 (PhCH_2_) ppm; HRMS (TOF, MS, ESI) calculated for C_16_H_16_N_2_O_2_ [*M* + H]^+^ *m/z* 269.1279, found 269.1288.

*4-Hydroxy-N’-[(E)-(4-methylphenyl)methylidene]benzohydrazide monomethanolate* (**1c**). The general procedure starting from 4-methylbenzaldehyde (**5c**, 240 mg, 2.0 mmol), 4-hydroxybenzohydrazide (**4b**, 304 mg, 2.0 mmol), in CH_3_OH (15 mL) was employed with a 2 h reaction time to obtain hydrazide-hydrazone **1c** in 92% yield, which was recrystallized slowly from hot CH_3_OH (30 mL/g) to obtain hydrazide-hydrazone **1c** crystallized with one molecule of CH_3_OH. Colorless crystals; 456 mg, 1.59 mmol, 80% yield; m.p. 246–247 °C (from CH_3_OH (252.6 °C [46]); selected FT-IR (ATR) ν_max_/cm^−1^: 3210 (br, O-H, N-H), 1622 (C=C), 1605 (C=O), 1538 (CH=N), 1500, 1351, 1280 (C-O), 1265, 1208, 1173, 1110, 1012, 956, 907, 854, 843, 814, 767, 742, 591 (br), 513, 478; ^1^H-NMR (DMSO-*d*_6_, 400 MHz): δ 11.59 (s, 1H, CONH), 10.13 (s, 1H, OH), 8.39 (s, 1H, CH=N), 7.81 (d, ^3^*J* = 8.7 Hz, 2H, H-2,6), 7.60 (d, ^3^*J* = 7.8 Hz, 2H, ArH-2,6), 7.25 (d, ^3^*J* = 7.8 Hz, 2H, ArH-3,5), 6.86 (d, ^3^*J* = 8.7 Hz, 2H, H-3,5), 4.12 (q, ^3^*J* = 4.8 Hz, 1H, CH_3_OH), 3.17 (d, ^3^*J* = 4.8 Hz, 3H, CH_3_OH), 2.33 (s, 3H, Me) ppm; ^13^C-NMR (DMSO-*d*_6_, 100 MHz): δ 162.67 (C=O), 160.61 (C-4), 146.85 (CH=N), 139.60 (ArC-4), 131.76 (ArC-1), 129.60 (C-3,5), 129.38 (ArC-3,5), 126.89 (ArC-2,6), 123.88 (C-1), 114.96 (C-2,6), 48.56 (CH_3_OH), 20.97 (Me) ppm.

*Sodium 2-{(E)-[2-(4-hydroxybenzoyl)hydrazinylidene]methyl}benzenesulfonate* (**1d**). The general procedure starting from ≥95% sodium 2-formylbenzenesulfonate (**5d**, 437 mg, ≥2.0 mmol), 4-hydroxybenzohydrazide (**4b**, 304 mg, 2.0 mmol), CH_3_OH (5.0 mL), in the absence of AcOH, was employed with a 2 h reaction time. The reaction mixture was concentrated in vacuum to a small volume of ca. 2 mL, then the product was isolated by slow crystallization in an open vessel to obtain hydrazide-hydrazone **1d** which crystallized with 2/3 a molecule of CH_3_OH. Yellow powder; 720 mg, 1.94 mmol, 97% yield, m.p. 251 °C (from CH_3_OH) with decomposition; selected FT-IR (ATR) ν_max_/cm^−1^: 3635 (CH_3_OH), 3377 (O-H), 3307 (N-H), 1608 (C=O), 1578 (C=C), 1556 (CH=N), 1510, 1272 (C-O), 1178 (SO_3_Na), 1134, 1017, 850, 760, 615, 563, 526, 482; ^1^H-NMR (CH_3_OH-*d*_4_, 400 MHz): δ 9.24 (s, 1H, CH=N), 8.32 (d, ^3^*J* = 7.6 Hz, 1H, H-3), 7.98 (dd, ^3^*J* = 7.7 Hz, ^4^*J* = 1.3 Hz, 1H, H-6), 7.87 (d, ^3^*J* = 8.8 Hz, 2H, ArH-2,6), 7.51 (dd, ^3^*J* = 7.7 Hz, ^3^*J* = 7.0 Hz, 1H, H-5), 7.45 (ddd, ^3^*J* = 7.6 Hz, ^3^*J* = 7.0 Hz, ^4^*J* = 1.3 Hz, 1H, H-4), 6.88 (d, ^3^*J* = 8.8 Hz, 2H, ArH-3,5), 3.35 (s, 3H, CH_3_OH) ppm; ^13^C-NMR (CH_3_OH-*d*_4_, 100 MHz): δ 167.07 (C=O), 162.80 (C-4), 148.93 (CH=N), 145.22 (ArC-2), 132.84 (ArC-1), 131.51 (ArC-5), 131.02 (C-2,6), 130.60 (ArC-4), 128.29 (ArC-3,6), 124.62 (C-1), 116.32 (C-3,5), 49.90 (CH_3_OH) ppm; HRMS (TOF, MS, ESI) calculated for C_14_H_11_N_2_O_5_S [*M* + Na]^+^ *m/z* 365.0173, found 365.0185.

*4-Hydroxy-N’-[(E)-(2-hydroxyphenyl)methylidene]benzohydrazide* (**1e**). The general procedure starting from salicylic aldehyde (**5e**, 244 mg, 2.0 mmol), 4-hydroxybenzohydrazide (**4b**, 304 mg, 2.0 mmol), CH_3_OH (40 mL) and AcOH (200 µL) was employed, with a 2 h reaction time. The reaction mixture was slowly concentrated to a small volume before crystallization to obtain compound **1e**. Colorless powder; 440 mg, 1.72 mmol, 86% yield; m.p. 265.0–266.5 °C (from CH_3_OH) (257–258 °C [47]); selected FT-IR (ATR) ν_max_/cm^−1^: 3318 (O-H), 3151 (br, O-H, N-H), 3025 (C-H), 1638 (C=C), 1607 (C=O), 1586, 1539 (CH=N), 1508, 1487, 1447, 1353, 1279 (C-O), 1264, 1235 (C-O), 1172, 1151, 1113, 953, 875, 842, 776, 769, 745, 730, 658, 616, 556, 472; ^1^H-NMR (DMSO-*d*_6_, 400 MHz): δ 11.93 (s, 1H, CONH), 11.43 (s, 1H, Ar-2-OH), 10.18 (s, 1H, 4-OH), 8.60 (s, 1H, CH=N), 7.83 (d, ^3^*J* = 8.6 Hz, 2H, H-2,6), 7.50 (dd, ^3^*J* = 7.7 Hz, ^4^*J* = 1.6 Hz, 1H, ArH-6), 7.19 (ddd, ^3^*J* = 8.7 Hz, ^3^*J* = 8.3 Hz, ^4^*J* = 1.6 Hz, 1H, ArH-4), 6.85–6.95 (m, 2H, ArH-3,5), 6.88 (d, ^3^*J* = 8.6 Hz, 2H, H-3,5) ppm; ^13^C-NMR (DMSO-*d*_6_, 100 MHz): δ 162.39 (C=O), 160.87 (C-4), 157.38 (ArC-2), 147.55 (CH=N), 131.09 (ArC-4), 129.68 (C-2,6), 129.57 (ArC-6), 123.13 (C-1), 119.23 (ArC-5), 118.64 (ArC-1), 116.34 (ArC-3), 115.07 (C-3,5) ppm; HRMS (TOF, MS, ESI) calculated for C_14_H_12_N_2_O_3_ [*M* + Na]^+^ *m/z* 279.0735, found 279.0745.

*4-Hydroxy-N’-[(E)-(3-hydroxyphenyl)methylidene]benzohydrazide monohydrate* (**1f**). The general procedure starting from 3-hydroxybenzaldehyde (**5f**, 122 mg, 1.0 mmol), 4-hydroxybenzohydrazide (**4b**, 152 mg, 1.0 mmol), CH_3_OH (2.5 mL), and AcOH (100 μL) was employed, with a 2 h reaction time. To the reaction mixture, cooled to RT, H_2_O (2.5 mL) was added before crystallization, to obtain **1f** which crystallized with one molecule of H_2_O. Pale crystals; 255 mg, 0.93 mmol, 93% yield; m.p. 254 °C (from mixture of CH_3_OH/H_2_O, 1/1, *v/v*) with decomposition (>250 °C [48]); selected FT-IR (ATR) ν_max_/cm^−1^: 3312 (O-H), 3054 (br, N-H, C-H), 2946 (C-H), 2825 (C-H), 1643 (C=O), 1609 (C=C), 1552 (CH=N), 1497, 1445, 1363, 1335, 1286, 1228 (C-O), 1169 (C-O), 1115, 900, 840, 787, 759, 692, 621, 563, 497, 461; ^1^H-NMR (DMSO-*d*_6_, 400 MHz): δ 11.58 (s, 1H, CONH), 10.12 (s, 1H, 4-OH), 9.61 (s, 1H, Ar-3-OH), 8.33 (s, 1H, CH=N), 7.80 (d, ^3^*J* = 8.7 Hz, 2H, H-2,6), 7.24 (dd, ^3^*J* = 8.0 Hz, ^3^*J* = 7.5 Hz, 1H, ArH-5), 7.18 (br s, 1H, ArH-2), 7.07 (d, ^3^*J* = 7.5 Hz, 1H, ArH-6), 6.86 (d, ^3^*J* = 8.7 Hz, 2H, H-3,5), 6.81 (dd, ^3^*J* = 8.0 Hz, ^4^*J* = 1.9 Hz, 1H, ArH-4) ppm; ^13^C-NMR (DMSO-*d*_6_, 100 MHz): δ 162.70 (C=O), 160.64 (C-4), 157.62 (ArC-3), 146.90 (CH=N), 135.75 (ArC-1), 129.83 (ArC-5), 129.63 (C-2,6), 123.84 (C-1), 118.65 (ArC-6), 117.20 (ArC-4), 114.99 (C-3,5), 112.47 (ArC-2) ppm; HRMS (TOF, MS, ESI) calculated for C_14_H_12_N_2_O_3_ [*M* + Na]^+^ *m/z* 279.0735, found 279.0747. ^1^H-NMR and ^13^C-NMR data and elementary analysis calculated for C_14_H_12_N_2_O_3_ × 1H_2_O are consistent with literature values [48].

*4-Hydroxy-N’-[(E)-(4-hydroxyphenyl)methylidene]benzohydrazide* (**1g**). The general procedure starting from 4-hydroxybenzaldehyde (**5g**, 244 mg, 2.0 mmol), 4-hydroxybenzohydrazide (**4b**, 304 mg, 2.0 mmol), CH_3_OH (2.5 mL) and AcOH (200 μL) was employed, with a 2 h reaction time to obtain of hydrazide-hydrazone **1g** which crystallizes with 1/3 a molecule of CH_3_OH. Pale crystals; 512 mg, 1.92 mmol, 96% yield; m.p. 261 °C (from CH_3_OH) with decomposition (>265 °C [49]); selected FT-IR (ATR) ν_max_/cm^−1^: 3653 (CH_3_OH), 3259 (br, O-H, N-H), 3024 (C-H), 1604 (C=O), 1577 (CH=N), 1507, 1449, 1371, 1248 (C-O), 1176, 1164, 1123, 1061, 1018, 840, 831, 625, 490, 444; ^1^H-NMR (DMSO-*d*_6_, 400 MHz): δ 11.45 (s, 1H, CONH), 10.09 (s, 1H, OH), 9.90 (s, 1H, OH), 8.32 (s, 1H, CH=N), 7.79 (d, ^3^*J* = 8.7 Hz, 2H, H-2,6), 7.54 (d, ^3^*J* = 8.2 Hz, 2H, ArH-2,6), 6.85 (d, ^3^*J* = 8.7 Hz, 2H, ArH-3,5), 6.83 (d, ^3^*J* = 8.2 Hz, 2H, H-3,5), 4.12 (q, ^3^*J* = 5.2 Hz, 1/3H, CH_3_OH), 3.17 (d, 3/3H, ^3^*J* = 5.2 Hz, CH_3_OH) ppm; ^13^C-NMR (DMSO-*d*_6_, 100 MHz): δ 162.64 (C=O), 160.54 (C-4), 159.25 (ArC-4), 147.29 (CH=N), 129.57 (C-2,6), 128.74 (ArC-2,6), 125.49 (ArC-1), 124.06 (C-1), 115.70 (ArC-3,5), 115.00 (C-3,5), 48.62 (CH_3_OH) ppm; HRMS (TOF, MS, ESI) calculated for C_14_H_12_N_2_O_3_ [*M* + Na]^+^ *m/z* 279.0735, found 279.0747.

*4-Hydroxy-N’-[(E)-(2-methoxyphenyl)methylidene]benzohydrazide* (**1h**). The general procedure starting from 2-methoxybenzaldehyde (**5h**, 272 mg, 2.0 mmol), 4-hydroxybenzohydrazide (**4b**, 304 mg, 2.0 mmol), CH_3_OH (10 mL), and AcOH (150 μL) was employed, with a 4 h reaction time. The reaction mixture was cooled to RT and H_2_O (2.5 mL) was added before crystallization to obtain hydrazide-hydrazone **1h**. Pale powder; 383 mg, 1.42 mmol, 71% yield; m.p. 236–237 °C (from mixture of CH_3_OH/H_2_O, 4/1, *v/v*) (230–231 °C [50]); the second fraction of hydrazide-hydrazone 1h was obtain from concentrated filtrates: 107 mg, 0.40 mmol, 20% yield; m.p. 236–237 °C; selected FT-IR (KBr) ν_max_/cm^−1^: 3090 (br, O-H, N-H), 2971 (C-H), 2942 (C-H), 2840 (C-H), 1607 (C=O), 1558 (C=N), 1508, 1468, 1360, 1315, 1286 (C-O), 1253 (C-O), 1235, 1172, 1055 (C-O), 1046, 835, 755, 618, 489; ^1^H-NMR (DMSO-*d*_6_, 400 MHz): δ 11.66 (s, 1H, NHCO), 10.11 (s, 1H, OH), 8.79 (s, 1H, CH=N), 7.86 (d, ^3^*J* = 7.6 Hz, 1H, ArH-6), 7.83 (d, ^3^*J* = 8.7 Hz, 2H, H-2,6), 7.40 (ddd, ^3^*J* = 8.8 Hz, ^3^*J* = 7.4 Hz, ^4^*J* = 1.6 Hz, 1H, ArH-4), 7.09 (d, ^3^*J* = 8.2 Hz, 1H, ArH-3), 7.01 (dd, ^3^*J* = 7.6 Hz, ^3^*J* = 7.4 Hz, 1H, ArH-5), 6.86 (d, ^3^*J* = 8.7 Hz, 2H, H-3,5), 3.86 (s, 3H, OMe) ppm; ^13^C-NMR (DMSO-*d*_6_, 100 MHz): δ 162.52 (C=O), 160.57 (C-4), 157.57 (ArC-2), 142.19 (CH=N), 131.24 (ArC-4), 129.59 (C-2,6), 125.36 (ArC-6), 123.79 (C-1), 122.46 (ArC-1), 120.66 (ArC-5), 114.92 (C-3,5), 111.73 (ArC-3), 55.58 (OMe) ppm; HRMS (TOF, MS, ESI) calculated for C_15_H_14_N_2_O_3_ [*M* + Na]^+^ *m/z* 293.0891, found 293.0889.

*4-Hydroxy-N’-[(E)-(3-methoxyphenyl)methylidene]benzohydrazide* (**1i**). The general procedure starting from 3-methoxybenzaldehyde (**5i**, 136 mg, 1.0 mmol), 4-hydroxybenzohydrazide (**4b**, 152 mg, 1.0 mmol), CH_3_OH (5.0 mL), and AcOH (100 µL) was employed, with a 5 h reaction time. The reaction mixture was concentrated to a volume of ca. 2.2 mL, and H_2_O (300 µL) was added before crystallization to obtain hydrazide-hydrazone **1i**. Colorless powder; 213 mg, 0.79 mmol, 79% yield; m.p. 210–212 °C (from a mixture of H_2_O/CH_3_OH, 1/7, *v/v*) (205–206 °C [50]); selected FT-IR (ATR) ν_max_/cm^−1^: 3327 (O-H), 3282 (N-H), 3074 (C-H), 2942 (C-H), 2831 (C-H), 1645 (C=C), 1605 (C=O), 1581, 1539 (CH=N), 1508, 1363, 1264 (C-O), 1240 (C-O), 1171, 1104, 1029 (C-O), 850, 783, 752, 688, 621, 529, 504; ^1^H-NMR (DMSO-*d*_6_, 400 MHz): δ 11.66 (s, 1H, CONH), 10.14 (s, 1H, OH), 8.41 (s, 1H, CH=N), 7.81 (d, ^3^*J* = 8.7 Hz, 2H, H-2,6), 7.36 (dd, ^3^*J* = 8.3 Hz, ^3^*J* = 7.8 Hz, 1H, ArH-5), 7.23–7.30 (m, 2H, ArH-2, ArH-6), 7.00 (dd, ^3^*J* = 7.8 Hz, ^4^*J* = 2.0 Hz, 1H, ArH-4), 6.87 (d, ^3^*J* = 8.7 Hz, 2H, H-3,5), 3.80 (s, 3H, OMe) ppm; ^13^C-NMR (DMSO-*d*_6_, 100 MHz): δ 162.80 (C=O), 160.68 (C-4), 159.49 (ArC-3), 146.74 (CH=N), 135.91 (ArC-1), 129.88 (ArC-5), 129.66 (C-2,6), 123.78 (C-1), 119.92 (ArC-6), 115.99 (ArC-4), 114.99 (C-3,5), 111.00 (ArC-2), 55.09 (OMe) ppm; HRMS (TOF, MS, ESI) calculated for C_15_H_14_N_2_O_3_ [*M* + Na]^+^ *m/z* 293.0891, found 293.0895.

*4-Hydroxy-N’-[(E)-(4-methoxyphenyl)methylidene]benzohydrazide* (**1j**). The general procedure starting from 4-methoxybenzaldehyde (**5j**, 272 mg, 2.0 mmol), 4-hydroxybenzohydrazide (**4b**, 304 mg, 2.0 mmol), and CH_3_OH (10 mL) in the absence of AcOH, with a 5 h reaction time to obtain hydrazide-hydrazone **1j**. White needless; 443 mg, 1.64 mmol, 82% yield; m.p. 223–224 °C (from CH_3_OH) (220–221 °C [50]); selected FT-IR (ATR) ν_max_/cm^−1^: 3156 (br, O-H, N-H), 3043 (C-H), 3006 (C-H), 2839 (C-H), 1595 (C=O), 1576 (C=C), 1553 (CH=N), 1505, 1439, 1361, 1312, 1277, 1249 (C-O), 1230 (C-O), 1167, 1060 (C-O), 1020, 962, 842, 827, 756, 682, 619, 546, 529; ^1^H-NMR (DMSO-*d*_6_, 601 MHz): δ 11.50 (s, 1H, CONH), 10.09 (s, 1H, OH), 8.37 (s, 1H, CH=N), 7.79 (d, ^3^*J* = 8.7 Hz, 2H, H-2,6), 7.65 (d, ^3^*J* = 8.6 Hz, 2H, ArH-2,6), 7.01 (d, ^3^*J* = 8.6 Hz, 2H, ArH-3,5), 6.85 (d, ^3^*J* = 8.7 Hz, 2H, H-3,5), 3.80 (s, 3H, OMe) ppm; ^13^C-NMR (DMSO-*d*_6_, 151 MHz): δ 162.58 (C=O), 160.60 (ArC-4), 160.52 (C-4), 146.73 (CH=N), 129.52 (C-2,6), 128.47 (ArC-2,6), 127.01 (ArC-1), 123.96 (C-1), 114.93 (C-3,5), 114.23 (ArC-3,5), 55.20 (OMe) ppm; ^1^H-NMR (DMSO-*d*_6_, 400 MHz): δ 11.52 (s, 1H, CONH), 10.11 (s, 1H, OH), 8.37 (s, 1H, CH=N), 7.80 (d, ^3^*J* = 8.7 Hz, 2H, H-2,6), 7.65 (d, ^3^*J* = 8.5 Hz, 2H, ArH-2,6), 7.01 (d, ^3^*J* = 8.5 Hz, 2H, ArH-3,5), 6.86 (d, ^3^*J* = 8.7 Hz, 2H, H-3,5), 3.80 (s, 3H, OMe) ppm; ^13^C-NMR (DMSO-*d*_6_, 100 MHz); δ 162.56 (C=O), 160.61 (ArC-4), 160.52 (C-4), 146.71 (CH=N), 129.53 (C-2,6), 128.47 (ArC-2,6), 127.02 (ArC-1), 123.97 (C-1), 114.93 (C-3,5), 114.25 (ArC-3,5), 55.21 (OMe) ppm; HRMS (TOF, MS, ESI) calculated for C_15_H_14_N_2_O_3_ [*M* + H]^+^ *m/z* 271.1072, found 271.1098.

*4-Hydroxy-N’-[(E)-(2-hydroxy-3-phenyl-phenyl)methylidene]benzohydrazide monomethanolate* (**2a**). The general procedure starting from 3-phenylsalicylic aldehyde (**6a**, 99 mg, 0.50 mmol) [54], 4-hydroxybenzohydrazide (**4b**, 76 mg, 0.50 mmol), CH_3_OH (6.0 mL), and AcOH (50 µL) was employed, with a 6 h reaction time. The reaction mixture was left for slow crystallization to obtain **2a** which crystallizes with one molecule of CH_3_OH. Colorless prisms; 155 mg, 0.425 mmol, 85% yield; m.p. 215–218 °C; selected FT-IR (ATR) ν_max_/cm^−1^: 3616 (CH_3_O-H), 3401 (O-H), 3249 (O-H), 3018 (C-H), 2500–3300 (br., N-H), 2939 (C-H), 1648 (C-C), 1608 (C=O), 1547 (CH=N), 1509, 1429, 1359, 1283 (C-O), 1240 (C-O), 1164, 1016, 951, 844, 746, 699, 650, 577 (br), 532, 454; ^1^H-NMR (DMSO-*d*_6_, 400 MHz): δ 12.44 (s, 1H, Ar-2-OH), 12.09 (s, 1H, CONH), 10.22 (s, 1H, 4-OH), 8.62 (s, 1H, CH=N), 7.84 (d, ^3^*J* = 8.7 Hz, 2H, H-2,6), 7.60 (dd, ^3^*J* = 7.8 Hz, ^4^*J* = 1.3 Hz, 2H, PhH-2,6), 7.44 (dd, ^3^*J* = 7.8 Hz, ^3^*J* = 7.4 Hz, 2H, PhH-3,5), 7.44 (dd, ^3^*J* = 7.6 Hz, ^4^*J* = 1.6 Hz, 1H, ArH-6), 7.38 (dd, ^3^*J* = 7.6 Hz, ^4^*J* = 1.6 Hz, 1H, ArH-4), 7.35 (tt, ^3^*J* = 7.4 Hz, ^4^*J* = 1.3 Hz, 1H, PhH-4), 7.03 (dd, ^3^*J* = 7.6 Hz, ^3^*J* = 7.6 Hz, 1H, ArH-5), 6.89 (d, ^3^*J* = 8.7 Hz, 2H, H-3,5), 4.12 (q, ^3^*J* = 5.1 Hz, 1H, CH_3_OH), 3.17 (d, ^3^*J* = 5.1 Hz, 3H, CH_3_OH) ppm; ^13^C-NMR (DMSO-*d*_6_, 100 MHz): δ 162.41 (C=O), 161.04 (C-4), 154.89 (ArC-2), 149.06 (CH=N), 137.49 (PhC-1), 131.99 (ArC-4), 130.39 (ArC-6), 129.77 (C-2,6), 129.18 (PhC-2,6), 128.75 (ArC-3), 128.01 (PhC-3,5), 126.96 (PhC-4), 122.89 (C-1), 119.42 (ArC-5), 118.30 (ArC-1), 115.16 (C-3,5), 48.57 (CH_3_OH) ppm; HRMS (TOF, MS, ESI) calculated for C_20_H_16_N_2_O_3_ [*M* + H]^+^ *m/z* 333.1228, found: 333.1227.

*4-Hydroxy-N’-[(E)-(3-tert-butyl-2-hydroxyphenyl)methylidene]benzohydrazide* (**2b**). The general procedure starting from 3-*tert*-butyl-salicylic aldehyde (**6b**, 356 mg, 2.0 mmol) [54], 4-hydroxybenzohydrazide (**4b**, 304 mg, 2.0 mmol), CH_3_OH (5.0 mL), and AcOH (100 µL) was employed, with a 6 h reaction time to obtain hydrazide-hydrazone **2b**. Pale powder; 580 mg, 1.86 mmol, 93% yield; m.p. 240–242 °C; selected FT-IR (ATR) ν_max_/cm^−1^: 3540 (O-H), 3438 (O-H), 2500–3300 (br, N-H), 3002 (C-H), 2956 (C-H), 2911 (C-H), 2871 (C-H), 1640 (C=C), 1602 (C=O), 1564 (CH=N), 1508, 1433, 1359, 1269 (C-O), 1239 (C-O), 1175, 1111, 846, 745, 634, 515; ^1^H-NMR (DMSO-*d*_6_, 400 MHz): δ 12.54 (s, 1H, Ar-2-OH), 12.01 (s, 1H, NHCO), 10.22 (s, 1H, 4-OH), 8.54 (s, 1H, CH=N), 7.84 (d, ^3^*J* = 8.7 Hz, 2H, H-2,6), 7.28 (dd, ^3^*J* = 7.7 Hz, ^4^*J* = 1.6 Hz, 1H, ArH-4), 7.26 (dd, ^3^*J* = 7.7 Hz, ^4^*J* = 1.6 Hz, 1H, ArH-6), 6.90 (d, ^3^*J* = 8.7 Hz, 2H, H-3,5), 6.87 (dd, ^3^*J* = 7.7 Hz, ^3^*J* = 7.7 Hz, 1H, ArH-5), 1.40 (s, 9H, *t*Bu) ppm; ^13^C-NMR (DMSO-*d*_6_, 100 MHz): δ 162.30 (C=O), 160.96 (C-4), 156.80 (ArC-2), 149.78 (CH=N), 136.24 (ArC-3), 129.68 (C-2,6), 129.29 (ArC-6), 128.23 (ArC-4), 122.88 (C-1), 118.62 (ArC-5), 117.72 (ArC-1), 115.12 (C-3,5), 34.42 (C – *t*Bu), 29.16 (3 × CH_3_ – *t*Bu) ppm; HRMS (TOF, MS, ESI) calculated for C_18_H_20_N_2_O_3_ [*M* + H]^+^ *m/z* 313.1547, found: 313.1540.

*4-Hydroxy-N’-[(E)-(2,4-dihydroxyphenyl)methylidene]benzohydrazide* (**2c**) [48]. The general procedure starting from 4-hydroxysalicylic aldehyde (**6c**, 276 mg, 2.0 mmol), 4-hydroxybenzohydrazide (**4b**, 304 mg, 2.0 mmol), CH_3_OH (3.0 mL), and AcOH (100 µL) was employed, with a 2 h reaction time to obtain of hydrazide-hydrazone **2c**. Colorless crystals; 490 mg, 1.80 mmol, 90% yield; m.p. 294 °C with decomposition; selected FT-IR (ATR) ν_max_/cm^−1^: 3342 (O-H), 2500–3300 (O-H, N-H), 3078 (C-H), 3029 (C-H), 1632 (C=C), 1602 (C=O), 1589 (CH=N), 1504, 1451, 1357, 1255 (C-O), 1217 (C-O), 1172 (C-O), 1119, 980, 909, 842, 690, 633, 516, 482, 442; ^1^H-NMR (DMSO-*d*_6_, 400 MHz): δ 11.72 (s, 1H, CONH), 11.60 (s, 1H, Ar-2-OH), 10.13 (s, 1H, OH), 9.93 (s, 1H, OH), 8.46 (s, 1H, CH=N), 7.80 (d, ^3^*J* = 8.7 Hz, 2H, H-2,6), 7.27 (d, ^3^*J* = 8.4 Hz, 1H, ArH-6), 6.87 (d, ^3^*J* = 8.7 Hz, 2H, H-3,5), 6.35 (dd, ^3^*J* = 8.4 Hz, ^4^*J* = 2.3 Hz, 1H, ArH-5), 6.31 (d, ^4^*J* = 2.3 Hz, 1H, ArH-3) ppm; ^13^C-NMR (DMSO-*d*_6_, 100 MHz): δ 162.16 (C=O), 160.72 (ArC-4), 160.48 (C-4), 159.43 (ArC-2), 148.47 (CH=N), 131.34 (ArC-6), 129.56 (C-2,6), 123.37 (C-1), 115.05 (C-3,5), 110.57 (ArC-1), 107.57 (ArC-5), 102.65 (ArC-3) ppm; HRMS (TOF, MS, ESI) calculated for C_14_H_12_N_2_O_4_ [*M* + H]^+^ *m/z* 273.0870, found 273.0872.

*4-Hydroxy-N’-[(E)-(5-bromo-2-hydroxyphenyl)methylidene]benzohydrazide* (**2d**). The general procedure starting from 5-bromosalicylic aldehyde (**6d**, 201 mg, 1.0 mmol) [55], 4-hydroxybenzohydrazide (**4b**, 152 mg, 1.0 mmol), CH_3_OH (15 mL), and AcOH (100 µL) was employed, with a 3 h reaction time to obtain of hydrazide-hydrazone **2d**. Pale shine prisms; 303 mg, 0.90 mmol, 90%; m.p. 276–278 °C (279–280 °C [49]); selected FT-IR (ATR) ν_max_/cm^−1^: 3291 (O-H), 3238 (N-H, O-H), 3039 (C-H), 2917 (C-H), 1616 (C=C), 1604 (C=O), 1543 (CH=N), 1507, 1472, 1341, 1276 (C-O), 1265 (C-O), 1197, 1187, 1170, 1105, 847, 831, 765, 689, 649, 623, 610, 560, 476; ^1^H-NMR (DMSO-*d*_6_, 400 MHz): δ 12.01 (s, 1H, NHCO), 11.42 (s, 1H, Ar-2-OH), 10.19 (s, 1H, 4-OH), 8.57 (s, 1H, CH=N), 7.83 (d, ^3^*J* = 8.7 Hz, 2H, H-2,6), 7.76 (d, ^4^*J* = 2.5 Hz, 1H, ArH-6), 7.41 (dd, ^3^*J* = 8.7 Hz, ^4^*J* = 2.5 Hz, 1H, ArH-4), 6.89 (d, ^3^*J* = 8.7 Hz, 1H, ArH-3), 6.87 (d, ^3^*J* = 8.7 Hz, 2H, ArH-3,5) ppm; ^13^C-NMR (DMSO-*d*_6_, 100 MHz): δ 162.50 (C=O), 160.91 (C-4), 156.33 (ArC-2), 148.92 (CH=N), 133.25 (ArC-4), 130.54 (ArC-6), 129.74 (C-2,6), 123.04 (C-1), 121.26 (ArC-1), 118.59 (ArC-3), 115.05 (C-3,5), 110.30 (ArC-5) ppm; HRMS (TOF, MS, ESI) calculated for C_14_H_11_BrN_2_O_3_ [M + Na]^+^ *m/z* 356.9845, found 356.9857.

*4-Hydroxy-N’-[(E)-(6-methoxy-2-hydroxyphenyl)methylidene]benzohydrazide* (**2e**). The general procedure starting from 6-methoxysalicylic aldehyde (**6**e, 152 mg, 1.0 mmol) [57], 4-hydroxybenzohydrazide (**4b**, 152 mg, 1.0 mmol), CH_3_OH (25 mL), and AcOH (100 µL) was employed, with a 2 h reaction time to obtain of hydrazide-hydrazone **2e**. Colorless solids; 202 mg, 0.71 mmol, 71% yield; m.p. 256.5–257.5 °C; selected FT-IR (ATR) ν_max_/cm^−1^: 3139 (O-H), 3016 (N-H), 2947 (C-H), 1601 (C=O), 1544 (CH=N), 1509, 1498, 1466, 1347, 1283 (C-O), 1230 (C-O), 1169, 1058 (C-O), 954, 846, 769, 671, 650, 612, 486; ^1^H-NMR (DMSO-*d*_6_, 400 MHz): δ 12.31 (s, 1H, Ar-2-OH), 11.98 (s, 1H, CONH), 10.20 (s, 1H, 4-OH), 8.93 (s, 1H, CH=N), 7.48 (d, ^3^*J* = 8.7 Hz, 2H, H-2,6), 7.25 (dd, ^3^*J* = 8.3 Hz, ^3^*J* = 8.3 Hz, 1H, ArH-4), 6.88 (d, ^3^*J* = 8.7 Hz, 2H, H-3,5), 6.55 (d, ^3^*J* = 8.3 Hz, 1H, ArH-3), 6.54 (d, ^3^*J* = 8.3 Hz, 1H, ArH-5), 3.85 (s, 3H, OMe) ppm; ^13^C-NMR (DMSO-*d*_6_, 100 MHz): δ 162.07 (C=O), 160.93 (C-4), 159.12 (ArC-2), 158.37 (ArC-6), 144.65 (CH=N), 132.07 (ArC-4), 129.65 (C-2,6), 122.87 (C-1), 115.11 (C-3,5), 109.35 (ArC-3), 106.87 (ArC-1), 101.51 (ArC-5), 55.83 (Me) ppm; HRMS (TOF, MS, ESI) calculated for C_15_H_14_N_2_O_4_ [*M* + H]^+^ *m/z* 287.10263, found 287.1035.

*4-Hydroxy-N’-[(E)-(2,6-dimethoxyphenyl)methylidene]benzohydrazide* (**2f**). The general procedure starting from 2,6-dimethoxybenzaldehyde (**6f**, 166 mg, 1.0 mmol) [58], 4-hydroxybenzohydrazide (**4b**, 152 mg, 1.0 mmol), CH_3_OH (3.0 mL), and AcOH (100 µL) was employed, with a 6 h reaction time and concentrated before crystallization to obtain of hydrazide-hydrazone **2f**. Colorless powder; 290 mg, 0.97 mmol, 97% yield; in ratio 4:1 of *E*/*Z* conformation, respectively, with respect to the C=N double bond determined by ^1^H-NMR spectroscopy in DMSO-*d*_6_; m.p. 214.5–216.0 °C (from CH_3_OH); selected FT-IR (ATR) ν_max_/cm^−1^: 3222 (N-H, O-H), 3085 (C-H), 3049 (C-H), 2938 (C-H), 2836 (C-H), 1605 (C=O), 1578 (C=C, CH=N), 1546, 1468, 1365, 1254 (C-O), 1216, 1172, 1111 (C-O), 1051, 1032, 842, 776, 671, 632, 610, 526, 481; ^1^H-NMR (DMSO-*d*_6_, 400 MHz): δ 11.46 (s, 1H, CONH), 10.80 (s, 1H, 4-OH), 8.59 (s, 1H, CH=N), 7.81 (d, ^3^*J* = 8.1 Hz, 2H, H-2,6), 7.32 (t, ^3^*J* = 8.4 Hz, 1H, ArH-4), 6.85 (d, ^3^*J* = 8.1 Hz, 2H, H-3,5), 6.71 (d, ^3^*J* = 8.4 Hz, 2H, ArH-3,5), 3.79 (s, 6H, OMe) ppm; ^13^C-NMR (DMSO-*d*_6_, 100 MHz): δ 162.36 (C=O), 160.43 (C-4), 158.57 (ArC-2,6), 142.48 (CH=N), 130.90 (ArC-4), 129.65 (C-2,6), 124.05 (C-1), 114.88 (C-3,5), 111.24 (ArC-1), 104.31 (ArC-3,5), 55.87 (2 × OMe) ppm; HRMS (TOF, MS, ESI) calculated for C_16_H_16_N_2_O_4_ [*M* + Na]^+^ *m/z* 323.1002, found 323.1002.

*4-Hydroxy-N’-[(E)-(4-hydroxy-3-methoxyphenyl)methylidene]benzohydrazide* (**2g**) [52]. The general procedure starting from vanillic aldehyde (**6g**, 1.14 g, 7.5 mmol), 4-hydroxybenzohydrazide (**4b**, 1.14 g, 7.5 mmol), CH_3_OH (4.5 mL), and AcOH (400 µL) was employed, with a 3 h reaction time and H_2_O (4.5 mL) was added at RT before crystallization to quantitatively obtain compound **2g**. Colorless prisms; 2.15 g, 7.5 mmol, 100% yield; m.p. 218.5–220.0 °C (from CH_3_OH) (226–227 [51]); selected FT-IR (ATR) ν_max_/cm^−1^: 3257 (N-H, O-H), 3053 (C-H), 2999 (C-H), 2969 (C-H), 2883 (C-H), 1639 (C=C), 1584 (C=O, CH=N), 1507, 1259 (C-O), 1244 (C-O), 1176, 1167, 1128, 1066, 1038, 848, 808, 648, 623, 548, 490; ^1^H-NMR (DMSO-*d*_6_, 400 MHz): δ 11.48 (s, 1H, CONH), 10.10 (s, 1H, 4-OH), 9.51 (s, 1H, Ar-4-OH), 8.32 (s, 1H, CH=N), 7.80 (d, ^3^*J* = 8.7 Hz, 2H, H-2,6), 7.30 (s, 1H, ArH-2), 7.06 (dd, ^3^*J* = 8.1 Hz, ^4^*J* = 1.2 Hz, 1H, ArH-6), 6.86 (d, ^3^*J* = 8.7 Hz, 2H, H-3,5), 6.83 (d, ^3^*J* = 8.1 Hz, 1H, ArH-5), 3.82 (s, 3H, OMe) ppm; ^13^C-NMR (DMSO-*d*_6_, 100 MHz): δ 162.67 (C=O), 160.56 (C-4), 148.80 (ArC-4), 148.01 (ArC-3), 147.53 (CH=N), 129.57 (C-2,6), 125.90 (ArC-1), 124.02 (C-1), 122.05 (ArC-6), 115.04 (ArC-5), 114.99 (C-3,5), 108.76 (ArC-2), 55.47 (OMe) ppm; HRMS (TOF, MS, ESI) calculated for C_15_H_14_N_2_O_4_ [*M* + Na]^+^ *m/z* 309.0851, found 309.0845.

*4-Methoxy-N’-[(E)-2-hydroxy-3-phenyl-benzylidene]benzohydrazide* (**2h**). The general procedure starting from 3-phenylsalicylic aldehyde (**6a**, 198 mg, 1.0 mmol) [54], 4-methoxybenzohydrazide (**4c**, 166 mg, 1.0 mmol) [89], CH_3_OH (50 mL), and AcOH (100 μL) was employed, with a 3 h reaction time to obtain of hydrazide-hydrazone **2h**. White cotton; 266 mg, 0.77 mmol, 77% yield; m.p. 240.0–242.5 °C (from CH_3_OH); the second fraction of hydrazide-hydrazone 2h was obtain from concentrated filtrates: 25 mg, 0.07 mmol, 7% yield; m.p. 239.5–242.0 °C; selected FT-IR (ATR) ν_max_/cm^−1^: 3168 (br, N-H, O-H), 3019 (C-H), 2989 (C-H), 2963 (C-H), 2931 (C-H), 1647 (C=C), 1603 (C=O), 1545 (CH=N), 1508, 1427, 1359, 1281, 1252 (C-O), 1176, 1111, 1027 (C-O), 961, 883, 847, 757, 748, 698, 649, 611, 562, 483; ^1^H-NMR (DMSO-*d*_6_, 400 MHz): δ 12.40 (s, 1H, OH), 12.18 (s, 1H, NHCO), 8.64 (s, 1H, CH=N), 7.95 (d, ^3^*J* = 8.9 Hz, 2H, H-2,6), 7.61 (dd, ^3^*J* = 7.8 Hz, ^4^*J* = 1.3 Hz, 2H, PhH-2,6), 7.46 (dd, ^3^*J* = 7.6 Hz, ^4^*J* = 1.4 Hz, 1H, ArH-4), 7.44 (dd, ^3^*J* = 7.8 Hz, ^3^*J* = 7.6 Hz, 2H, PhH-3,5), 7.39 (dd, ^3^*J* = 7.6 Hz, ^4^*J* = 1.4 Hz, 1H, ArH-6), 7.35 (tt, ^3^*J* = 7.6 Hz, ^4^*J* = 1.3 Hz, 1H, PhH-4), 7.09 (d, ^3^*J* = 8.9 Hz, 2H, H-3,5), 7.04 (dd, ^3^*J* = 7.6 Hz, ^3^*J* = 7.6 Hz, 1H, ArH-5), 3.84 (s, 3H, OMe) ppm; ^13^C-NMR (DMSO-*d*_6_, 100 MHz): δ 162.25 (C=O), 162.15 (C-4), 154.87 (ArC-2), 149.35 (CH=N), 137.43 (PhC-1), 132.05 (ArC-4), 130.44 (ArC-6), 129.58 (C-2,6), 128.73 (ArC-3), 127.99 (PhC-3,5), 126.95 (PhC-4), 124.39 (C-1), 119.43 (ArC-5), 118.22 (ArC-1), 113.82 (C-3,5), 55.41 (OMe) ppm; HRMS (TOF, MS, ESI) calculated for C_21_H_18_N_2_O_3_ [*M* + Na]^+^ *m/z* 369.1210, found 369.1198.

*4-Hydroxy-N’-[(E)-2-hydroxy-3-hydroxymethyl-5-methyl-benzylidene]benzohydrazide* (**3a**). The general procedure starting from 3-hydroxymethyl-5-methylsalicylic aldehyde (**7a**, 166 mg, 1.0 mmol) [59], 4-hydroxybenzohydrazide (**4b**, 152 mg, 1.0 mmol), CH_3_OH (4.0 mL), and AcOH (100 µL) was employed with a 3 h reaction time to obtain of hydrazide-hydrazone **3a**. Colorless crystals; 230 mg, 0.77 mmol, 77% yield; m.p. 229–230 °C; selected FT-IR (ATR) ν_max_/cm^−1^: 3550 (CH_2_O-H), 3265 (O-H), 3211 (O-H), 2500–3200 (br, N-H), 3046 (C-H), 2878 (C-H), 1625 (C=C), 1602 (C=O), 1582, 1559 (CH=N), 1511, 1456, 1357, 1297, 1258 (C-O), 1178, 1051 (CH_2_-O), 1024, 1000, 959, 848, 766, 675, 652, 621, 504, 419; ^1^H-NMR (DMSO-*d*_6_, 400 MHz): δ 11.98 (s, 1H, NHCO), 11.80 (s, 1H, Ar-2-OH), 10.20 (s, 1H, 4-OH), 8.50 (s, 1H, CH=N), 7.83 (d, ^3^*J* = 8.6 Hz, 2H, H-2,6), 7.24 (d, ^4^*J* = 1.6 Hz, 1H, ArH-4), 7.10 (d, ^4^*J* = 1.6 Hz, 1H, ArH-6), 6.88 (d, ^3^*J* = 8.6 Hz, 2H, H-3,5), 5.07 (t, ^3^*J* = 5.3 Hz, 1H, CH_2_OH), 4.55 (d, ^3^*J* = 5.3 Hz, 2H, CH_2_OH), 2.27 (s, 3H, Me) ppm; ^13^C-NMR (DMSO-*d*_6_, 100 MHz): δ 162.34 (C=O), 160.94 (C-4), 152.39 (ArC-2), 148.98 (CH=N), 129.72 (C-2,6, ArC-4), 129.46 (ArC-3), 128.81 (ArC-6), 127.24 (ArC-5), 123.00 (C-1), 116.91 (ArC-1), 115.12 (C-3,5), 57.57 (CH_2_), 20.10 (Me) ppm; HRMS (TOF, MS, ESI) calculated for C_16_H_16_N_2_O_4_ [*M* + Na]^+^ *m/z* 323.1002, found 323.1008.

*4-Hydroxy-N’-[(E)-(5-hydroxymethyl-3-methyl-2-hydroxyphenyl)methylidene]benzohydrazide* (**3b**). The general procedure starting from 5-hydroxymethyl-3-methylsalicylic aldehyde (**7b**, 83.1 mg, 0.50 mmol) [56], 4-hydroxybenzohydrazide (**4b**, 76.1 mg, 0.50 mmol), CH_3_OH (35 mL), and AcOH (50 μL) was employed with a 3 h reaction time to obtain of hydrazide-hydrazone **3b**. Colorless crystals; 129 mg, 0.43 mmol, 86% yield; m.p. 266 °C (with decomposition); selected FT-IR (ATR) ν_max_/cm^−1^: 3370 (O-H), 3234 (O-H), 2200–3200 (br, O-H, N-H), 1647 (C=C), 1580 (C=O), 1570 (CH=N), 1511, 1434, 1364, 1261, 1241 (C-O), 1177 (C-O), 1167, 1090, 1003 (C-O), 957, 847, 749, 662, 641, 618, 532, 449; ^1^H-NMR (DMSO-*d*_6_, 400 MHz): δ 11.99 (s, 1H, NHCO), 11.90 (s, 1H, Ar-2-OH), 10.21 (s, 1H, 4-OH), 8.52 (s, 1H, CH=N), 7.84 (d, ^3^*J* = 8.7 Hz, 2H, H-2,6), 7.18 (s, 1H, ArH-6), 7.15 (s, 1H, ArH-4), 6.89 (d, ^3^*J* = 7.8 Hz, 2H, H-3,5), 5.08 (t, ^3^*J* = 5.6 Hz, 1H, CH_2_OH), 4.41 (d, ^3^*J* = 5.6 Hz, 2H, CH_2_OH), 2.21 (s, 3H, Me) ppm; ^13^C-NMR (DMSO-*d*_6_, 100 MHz): δ 162.33 (C=O), 160.93 (C-4), 154.72 (ArC-2), 149.01 (CH=N), 132.75 (ArC-5), 130.99 (ArC-4), 129.71 (C-2,6), 126.50 (ArC-6), 124.57 (ArC-3), 123.00 (C-1), 116.77 (ArC-1), 115.11 (C-3,5), 62.36 (CH_2_), 15.48 (Me) ppm; HRMS (TOF, MS, ESI) calculated for C_16_H_16_N_2_O_4_ [*M* + Na]^+^ *m/z* 323.1002, found 323.1002.

*4-Hydroxy-N’-[(E)-(3,5-di-tert-butyl-2-hydroxyphenyl)methylidene]benzohydrazide* (**3c**). The general procedure starting from 3,5-di-*tert*-butyl-salicylic aldehyde (**7c**, 937 mg, 4.0 mmol), 4-hydroxybenzohydrazide (**4b**, 0.61g, 4.0 mmol), CH_3_OH (10 mL), and AcOH (100 µL) was employed, with a 6 h reaction time to obtain of compound **3c**. Colorless needles; 1.22 g, 3.31 mmol, 83% yield; m.p. 274.5–275.5 °C (from CH_3_OH); the second fraction of **3c** was obtain from concentrated filtrates: 156 mg, 0.42 mmol, 10.5% yield; m.p. 274–275 °C; selected FT-IR (ATR) ν_max_/cm^−1^: 3161 (br, O-H, N-H), 2955 (C-H), 2907 (C-H), 2867 (C-H), 1642 (C=C), 1607 (C=O), 1583 (CH=N), 1558, 1511, 1435, 1355, 1280, 1248, 1233 (C-O), 1172 (C-O), 891, 844, 769, 665, 618, 494; ^1^H-NMR (DMSO-*d*_6_, 400 MHz): δ 12.35 (s, 1H, Ar-OH), 11.99 (s, 1H, NHCO), 10.21 (s, 1H, 4-OH), 8.54 (s, 1H, CH=N), 7.84 (d, ^3^*J* = 8.6 Hz, 2H, H-2,6), 7.30 (d, ^4^*J* = 2.1 Hz, 1H, ArH-4), 7.20 (d, ^4^*J* = 2.1 Hz, 1H, ArH-6), 6.89 (d, ^3^*J* = 8.6 Hz, 2H, H-3,5), 1.41 (s, 9H, Ar-3-*t*Bu), 1.28 (s, 9H, Ar-5-*t*Bu) ppm; ^13^C-NMR (DMSO-*d*_6_, 100 MHz): δ 162.37 (C=O), 160.95 (C-4), 154.60 (ArC-2), 150.26 (CH=N), 140.26 (ArC-5), 135.53 (ArC-3), 129.70 (C-2,6), 125.52 (ArC-6), 125.26 (ArC-4), 123.00 (C-1), 117.06 (ArC-1), 115.13 (C-3,5), 34.59 (C-3-*t*Bu), 33.81 (C-5-*t*Bu), 31.25 (3 × CH_3_ – 5-*t*Bu), 29.24 (3 × CH_3_ – 3-*t*Bu) ppm; HRMS (TOF, MS, ESI) calculated for C_22_H_28_N_2_O_3_ [*M* + H]^+^ *m/z* 369.2173, found 369.2177.

*4-Hydroxy-N’-[(E)-3-tert-butyl-2-hydroxy-5-methylbenzylidene]benzohydrazide* (**3d**). The general procedure starting from 3-*tert*-butyl-5-methylsalicylic aldehyde (**7d**, 385 mg, 2.0 mmol) [98], 4-hydroxybenzohydrazide (**4b**, 304 mg, 2.0 mmol), CH_3_OH (6.0 mL), and AcOH (100 μL) was employed, with a 2 h reaction time to obtain of hydrazide-hydrazone **3d** as colorless powder; 496 mg, 1.52 mmol, 76% yield; m.p. 261–263 °C (from CH_3_OH); selected FT-IR (ATR) ν_max_/cm^−1^: 3337 (O-H), 3217 (N-H), 3064 (C-H), 2957 (C-H), 2913 (C-H), 2866 (C-H), 1646 (C=C), 1607 (C=O), 1583 (CH=N), 1509, 1437, 1357, 1269 (C-O), 1224, 1207, 1172, 862, 757, 724, 700, 621, 488; ^1^H-NMR (DMSO-*d*_6_, 400 MHz): δ 12.30 (s, 1H, Ar-2-OH), 11.98 (s, 1H, NHCO), 10.19 (s, 1H, 4-OH), 8.49 (s, 1H, CH=N), 7.83 (d, ^3^*J* = 8.8 Hz, 2H, H-2,6), 7.08 (d, ^4^*J* = 2.0 Hz, 1H, ArH-4), 7.05 (d, ^4^*J* = 2.0 Hz, 1H, ArH-6), 6.89 (d, ^3^*J* = 8.8 Hz, 2H, H-3,5), 2.25 (s, 3H, Me), 1.39 (s, 9H, *t*Bu) ppm; ^13^C-NMR (DMSO-*d*_6_, 100 MHz): δ 162.28 (C=O), 160.93 (C-4), 154.62 (ArC-2), 149.78 (CH=N), 136.03 (ArC-3), 129.66 (C-2,6), 129.22 (ArC-4), 129.01 (ArC-6), 126.87 (ArC-5), 122.93 (C-1), 117.41 (ArC-1), 115.10 (C-3,5), 34.32 (C – *t*Bu), 29.18 (3 × CH_3_ – *t*Bu), 20.19 (Me) ppm; HRMS (TOF, MS, ESI) calculated for C_19_H_22_N_2_O_3_ [*M* + Na]^+^ *m/z* 349.1523, found 349.1517.

*4-Hydroxy-N’-[(E)-(2-hydroxy-3-isopropyl-6-methylphenyl)methylidene]benzohydrazide* (**3e**). The general procedure starting from 3-isopropyl-6-methylsalicylic aldehyde (**7e**, 356 mg, 2.0 mmol) [64], 4-hydroxybenzohydrazide (**4b**, 304 mg, 2.0 mmol), CH_3_OH (6.0 mL), and AcOH (100 μL) was employed, with a 6 h reaction time to obtain of hydrazide-hydrazone **3e**. Colorless powder; 382 mg, 1.22 mmol, 61% yield; m.p. 258.5–260.5 °C (from CH_3_OH). After concentration of the filtrate the second fraction of hydrazide-hydrazone 3e was obtained; 209 mg, 0.67 mmol, 33.5%; m.p. 258.5–260.5 °C (from CH_3_OH); selected FT-IR (ATR) ν_max_/cm^−1^: 3502 (O-H), 3100 (br, O-H, N-H), 2940 (C-H), 2839 (C-H), 1626 (C=C), 1606 (C=O), 1587 (CH=N), 1563, 1502, 1460, 1449, 1332, 1263, 1245, 1218 (C-O), 1156, 1118, 1079, 1016, 853, 757, 619, 436, 424; ^1^H-NMR (DMSO-*d*_6_, 400 MHz): δ 12.66 (s, 1H, NHCO), 11.95 (s, 1H, ArOH), 10.22 (s, 1H, 4-OH), 8.87 (s, 1H, CH=N), 7.84 (d, ^3^*J* = 8.8 Hz, 2H, H-2,6), 7.11 (d, ^3^*J* = 7.9 Hz, 1H, ArH-4), 6.89 (d, ^3^*J* = 8.8 Hz, 2H, H-3,5), 6.70 (d, ^3^*J* = 7.9 Hz, 1H, ArH-5), 3.27 (m, ^3^*J* = 6.7 Hz, 1H, CH(CH_3_)_2_), 2.37 (s, 3H, Me), 1.18 (d, ^3^*J* = 7.9 Hz, 6H, CH(CH_3_)_2_) ppm; ^13^C-NMR (DMSO-*d*_6_, 100 MHz): δ 162.13 (C=O), 160.99 (C-4), 155.92 (ArC-2), 147.49 (CH=N), 135.23 (ArC-6), 133.23 (ArC-3), 129.64 (C-2,6), 127.67 (ArC-4), 122.93 (C-1), 120.71 (ArC-5), 115.19 (ArC-1), 115.16 (C-3,5), 26.01 (CH(CH_3_)_2_), 22.24 (CH(CH_3_)_2_), 18.63 (Me) ppm; HRMS (TOF, MS, ESI) calculated for C_18_H_20_N_2_O_3_ [*M* + H]^+^ *m/z* 313.1547, found: 313.1548.

*4-Hydroxy-N’-[(E)-(2-hydroxy-4,6-dimethoxyphenyl)methylidene]benzohydrazide* (**3f**). The general procedure starting from 4,6-dimethoxysalicylic aldehyde (**7f**, 364 mg, 2.0 mmol) [62] and 4-hydroxybenzohydrazide (**4b**, 304 mg, 2.0 mmol) in CH_3_OH (5.0 mL), and AcOH (100 µL) was employed, with a 5 h reaction time to obtain of hydrazide-hydrazone **3f** which crystallize with CH_3_OH in 3/2 molar ratio. Pale yellow powder; 668 mg, 1.98 mmol, 99% yield; loss crystalline solvent from 130 °C to 142 °C, m.p. 231.5–234.5 °C (from CH_3_OH); selected FT-IR (ATR) ν_max_/cm^−1^: 3502 (O-H), 3447 (O-H), 3414 (O-H), 3170 (N-H), 3023 (C-H), 2957 (C-H), 2929 (C-H), 2866 (C-H), 1607 (C=O), 1588, 1545 (CH=N), 1427, 1355, 1276 (C-O), 1260 (C-O), 1248 (C-O), 1229 (C-O), 1175, 1108, 955, 846, 813, 613, 483; ^1^H-NMR (DMSO-*d*_6_, 400 MHz): δ 12.51 (s, 1H, Ar-2-OH), 11.81 (s, 1H, NHCO), 10.16 (s, 1H, 4-OH), 8.81 (s, 1H, CH=N), 7.81 (d, ^3^*J* = 8.7 Hz, 2H, H-2,6), 6.87 (d, ^3^*J* = 8.7 Hz, 2H, H-3,5), 6.14 (d, ^4^*J* = 2.3 Hz, 1H, ArH-3 or ArH-5), 6.13 (d, ^4^*J* = 2.3 Hz, 1H, ArH-3 or ArH-5), 4.11 (q, ^3^*J* = 5.1 Hz, 2/3H, CH_3_OH), 3.83 (s, 3H, OMe), 3.78 (s, 3H, OMe), 3.17 (d, ^3^*J* = 5.1 Hz, 2H, CH_3_OH) ppm; ^13^C-NMR (DMSO-*d*_6_, 100 MHz): δ 162.92 (ArC-6), 161.93 (C=O), 160.81 (ArC-2 or C-4), 160.77 (ArC-2 or C-4), 159.34 (ArC-4), 144.86 (CH=N), 129.57 (C-2,6), 123.12 (C-1), 115.10 (C-3,5), 100.64 (ArC-1), 93.81 (ArC-3 or ArC-5), 90.35 (ArC-3 or ArC-5), 55.84 (OMe), 55.35 (OMe), 48.57 (CH_3_OH) ppm; HRMS (TOF, MS, ESI) calculated for C_16_H_16_N_2_O_5_ [*M* + H]^+^ *m/z* 317.1132, found 317.1145.

*3-Hydroxy-N’-[(E)-(3-tert-butyl-2-hydroxy-5-methylphenyl)methylidene]benzohydrazide* (**3g**). The general procedure starting from 3-*tert*-butyl-5-methylsalicylic aldehyde (**7d**, 192 mg, 1.0 mmol) [98], 3-hydroxybenzohydrazide (**4c**, 152 mg, 1.0 mmol), CH_3_OH (3.0 mL), and AcOH (100 µL) was employed with a 2 h reaction time and concentrated by slow distillation (to a volume of ca. 1.0 mL) before crystallization to obtain of hydrazide-hydrazone **3g**. Colorless prisms; 306 mg, 0.94 mmol, 94% yield, m.p. 240.0–242.5 °C; selected FT-IR (ATR) ν_max_/cm^−1^: 3226 (N-H and O-H), 3065 (Ar-H), 2957 (C-H), 2914 (C-H), 2867 (C-H), 1644 (C=O), 1614 (C=C), 1586, 1559 (CH=N), 1455, 1438, 1360, 1309, 1263, 1236 (C-O), 1209 (C-O), 1166, 1091, 952, 850, 786, 742, 716, 689, 674, 607, 500, 467, 448, 406; ^1^H-NMR (DMSO-*d*_6_, 400 MHz): δ 12.25 (s, 1H, Ar-2-OH), 12.13 (s, 1H, NHCO), 9.83 (s, 1H, 3-OH), 8.52 (s, 1H, CH=N), 7.31–7.39 (m, 3H, H-2, H-5, H-6), 7.09 (d, ^4^*J* = 1.7 Hz, 1H, ArH-4), 7.08 (d, ^4^*J* = 1.7 Hz, 1H, ArH-6), 7.01 (ddd, ^3^*J* = 7.3 Hz, ^4^*J* = 2.6 Hz, ^4^*J* = 1.7 Hz, 1H, H-4), 2.25 (s, 3H, Me), 1.39 (s, 9H, *t*Bu) ppm; ^13^C-NMR (DMSO-*d*_6_, 100 MHz): δ 162.70 (C=O), 157.45 (C-3), 154.72 (ArC-2), 150.66 (CH=N), 136.09 (ArC-3), 133.89 (C-1), 129.60 (C-5), 129.44 (ArC-4), 129.17 (ArC-6), 126.95 (ArC-5), 118.98 (C-4), 118.06 (C-6), 117.30 (ArC-1), 114.45 (C-2), 34.33 (C – *t*Bu), 29.18 (3 × CH_3_ – *t*Bu), 20.18 (CH_3_) ppm; HRMS (TOF, MS, ESI) calculated for C_19_H_22_N_2_O_3_ [*M* + Na]^+^ *m/z* 349.1523, found 349.1524.

### 3.3. Enzyme Inhibition Assay

The activity of laccase was determined using the procedure described by Leonowicz and Grzywnowicz [99]. Basically, a standard reaction mixture contained the enzyme in McIlvaine buffer (1.5 mL, pH 5.3) in the presence or absence of tested compounds (0.1 mL) and substrate – syringaldazine (0.1 mL). Two water-miscible media were tested as suitable co-solvents for hydrazide-hydrazones which are poorly soluble in water. For that purpose, the stability of laccase (40 nM, McIlvaine buffer, pH 5.3) in methanol and DMSO (6.25% *v/v*) at 25 °C was investigated (see Supplementary Material). The samples containing the enzyme, with a volumetric buffer–co-solvent ratio of 15:1, were withdrawn in different time intervals for 1500 min. A reaction system contained the 1.6 mL of the collected sample and 0.1 mL of syringaldazine (5.9 µM). Laccase activity was determined spectrophotometrically (UV-VIS 1800, Shimadzu, Reinach, Switzerland) by monitoring the changes in absorbance at 525 nm resulted from oxidation of the substrate syringaldazine with an extinction coefficient ε_0_ = 65000 M^−1^·cm^−1^ [99]. Kinetic parameters, *K*_m_ and *k*_3_ were determined for two protein concentrations (20 and 40 nM) and with the nine concentrations of syringaldazine (0.59 to 20 µM), using the Michaelis-Menten model, Equation (1) [76]:

V = V_max_ · C_S_/(K_m_ + C_S_), k_3_ = V_max_/C_E_ (1)

where K_m_ [µM], k_3_ [min^–1^] – kinetic constants, V_max_ – maximal velocity [µM·min^–1^], C_S_ – substrate concentration [µM], C_E_ – enzyme concentration [µM].

The parameters of the function were estimated using the nonlinear least square method in Matlab (The Mathworks, R2019a Version 9.6, academic licence). The method was used simultaneously for datasets obtain for different enzyme concertation. The common minimum of error function was found for all considered datasets resulting in one common estimate of each model parameter. The goodness of fit was assessed using the coefficient of determination (R^2^); the sum of squared errors (SSE) and root mean squared error (RMSE). The 25 hydrazide-hydrazones, 4-hydroxybenzoic acid (control, 4-HBA), 4-hydroxybenzohydrazide (**4a** – 4-HBAH), and sodium azide (control) were tested against laccase in the standard reaction mixture. Prior to the substrate addition, the laccase and tested compound were pre-incubated for 10 min at 25 °C. At least three inhibitor concentrations were used in each experiment. All measurements were performed in triplicate. To determine the type of inhibition Lineweaver–Burk transformation was used applying the following equations for competitive (2), uncompetitive (3) and non-competitive model (4) [76]. The parameters of these equations were estimated using the linear least square method.

1/V = *K*_m_/V_max_ · (1 + C_i_/*K*_i_) · 1/C_S_ + 1/V_max_ (2)

1/V = Km/Vmax · 1/CS + 1/Vmax · (1 + Ci/Ki)(3)

1/V = Km/Vmax · (1 + Ci/Ki) · 1/CS + 1/Vmax · (1 + Ci/Ki)(4)

where *K*_m_ [µM]—Michaelis-Menten constant, V_max_—maximal velocity [µM·min^–1^], *K*_i_—inhibition constant [µM], C_S_—substrate concentration [µM], C_i_—inhibitor concentration [µM].

### 3.4. Molecular Docking

A crystal structure of an enzyme was obtained from the RCSB Protein Data Bank (PDB). The 1GYC [87] was prepared using Schrödinger’s Protein Preparation Wizard module [100]. Water molecules and ligands were deleted and then hydrogen atoms were added and set states generated using the Epik with consideration of experimental pH. The next step was the optimization of structure with the use of PROPKA3 [101] with the experimental pH as well and final minimization with OPLS3e force field. All ligands were prepared and optimized with the OPLS3e force field in LigPrep Schrödinger’s module [102]. The stereochemistry of the imine carbon atom was considered. Inhibitors were docked with the Induced Fit Docking Protocole [103]. The copper atoms were selected as centroids of the box with a size of 20 Å. Glide docking and prime refinement were followed as the recommended procedures when the XP precision was selected in the Glide redocking. All of the docked ligands were primed again with Prime MM-GBSA protocol [104] and the compounds with the lower ΔG_Bind energy_ for each inhibitor were selected as the final results.

## 4. Conclusions

In this article, we identified a novel class of low-molecular-weight hydrazide-hydrazones, which contained naturally derived units of aldehydes having an aromatic motif and/or hydroxybenzoic acids. We presented the characteristics of 25 molecules, including 14 new ones, and their inhibition potency toward laccase of fungal origin. The results of the syringaldazine oxidation in the presence of the compounds indicated that they essentially acted as reversible competitive inhibitors, with some exceptions. The computational docking simulations showed that both aromatic fragments connected by a hydrazide linker in the molecules were crucial for their activity. The hydrazide fragment contained a *para*-hydroxybenzoic acid unit that served as a decoy, reminiscent of typical phenolic laccase substrates. In addition, salicylic aldehydes with bulky groups in the adjacent 3rd position provided strong interaction with the hydrophobic part of the substrate-binding centre. In this regard, the reversible competitive action on laccase of the tested hydrazide-hydrazones is beneficial for further design of inhibitors for bio-application.

## Figures and Tables

**Table 1:** Characteristics of hydrazide-hydrazones **1a–j**, **2a–h**, and **3a–g**.

No.	R	R^1^	R^2^	Rx time	Yield(%)	Mp (°C)	Mp lit.(°C)	Lit.
**1a**	Ph	OH	H	2h	96	242.0–244.0	240–242	[44]
**1b**	C_6_H_5_CH_2_CH_2_	OH	H	6h	72	243.5–244.0	–	[45]
**1c**	4-MeC_6_H_4_	OH	H	2h	80	246.0–247.0	252.6	[46]
**1d**	2-NaSO_3_C_6_H_4_	OH	H	2h	97	251 dec.	–	–
**1e**	2-HOC_6_H_4_	OH	H	2h	86	265.0–266.5	257–258	[47]
**1f**	3-HOC_6_H_4_	OH	H	2h	93 ^a^	254 dec.	>250	[48]
**1g**	4-HOC_6_H_4_	OH	H	2h	96 ^b^	261 dec.	265	[49]
**1h**	2-MeOC_6_H_4_	OH	H	4h	91	236.0–237.0	230–231	[50]
**1i**	3-MeOC_6_H_4_	OH	H	5h	79	210.0–212.0	205–206	[50]
**1j**	4-MeOC_6_H_4_	OH	H	5h	82	223.0–224.0	220–221	[50]
**2a**	2-HO-3-PhC_6_H_3_	OH	H	6h	85 ^c^	215.0–218.0	–	–
**2b**	2-HO-3-*t*BuC_6_H_3_	OH	H	6h	93	240.0–242.0	–	–
**2c**	2,4-(HO)_2_C_6_H_3_	OH	H	2h	90	294 dec.	–	[48]
**2d**	2-HO-5-BrC_6_H_3_	OH	H	3h	90	276.0–278.0	279–280	[49]
**2e**	2-HO-6-MeOC_6_H_3_	OH	H	2h	71	256.5–257.5	–	–
**2f**	2,6-(MeO)_2_C_6_H_3_	OH	H	6h	97	214.5–216.0	–	–
**2g**	3-MeO-4-HOC_6_H_3_	OH	H	3h	100	218.5–220.0	226–227	[51]
**2h**	2-HO-3-PhC_6_H_3_	OMe	H	3h	84	240.0–242.5	–	–
**3a**	2-HO-3-CH_2_OH-5-MeC_6_H_2_	OH	H	3h	77	229.0–230.0	–	–
**3b**	2-HO-3-Me-5-CH_2_OHC_6_H_2_	OH	H	3h	86	266 dec.	–	–
**3c**	2-HO-3,5-(*t*Bu)_2_C_6_H_2_	OH	H	6h	93.5	274.5–275.5	–	–
**3d**	2-HO-3-*t*Bu-5-MeC_6_H_2_	OH	H	2h	76	261.0–262.0	–	–
**3e**	2-HO-3-*i*Pr-6-MeC_6_H_2_	OH	H	6h	94.5	258.5–260.5	–	–
**3f**	2-HO-4,6-(MeO)_2_C_6_H_2_	OH	H	5h	99	231.5–234.5	–	–
**3g**	2-HO-3-*t*Bu-5-MeC_6_H_2_	H	OH	2h	94	240.0–242.5	–	–

^a^ Monohydrate was isolated, ^b^ Crystallize with 1/3 molecule of CH_3_OH, ^c^ Crystallize with one molecule of CH_3_OH.

**Table 2:** Structures of the hydrazide-hydrazones 1a–j, 2a–h, 3a–g and their inhibition constants (*K*_i_) determined for laccase from *Trametes versicolor*, and the skeleton numbering system. Significant inhibition is marked in bold. The activity of the reference compounds, 4-hydroxybutylacrylate (4-HBA), and NaN_3_ (a dioxygen reduction centre inhibitor), are given.

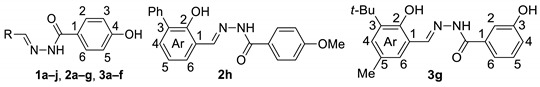					
**Nr**	**R**	***K*** **_i_** **[µM]**	**Nr**	**R**	***K*** **_i_** **[µM]**
**1a**	Ph	1468 ± 58 ^a^	**2e**	2-HO-6-MeOC_6_H_3_	≥1000 ^c^
**1b**	C_6_H_5_CH_2_CH_2_	1919 ± 163 ^b^	**2f**	2,6-(MeO)_2_C_6_H_3_	≥1000 ^c^
**1c**	4-MeC_6_H_4_	**251 ± 26 ^a^**	**2g**	3-MeO-4-HOC_6_H_3_	**251 ± 38 ^a^**
**1d**	2-NaSO_3_C_6_H_4_	≥2400 ^c^	**2h**	–	≥1000 ^c^
**1e**	2-HOC_6_H_4_	674 ± 10 ^a^	**3a**	2-HO-3-CH_2_OH-5-MeC_6_H_2_	≥1000 ^c^
**1f**	3-HOC_6_H_4_	2396 ± 334 ^a^	**3b**	2-HO-3-Me-5-CH_2_OHC_6_H_2_	≥1000 ^c^
**1g**	4-HOC_6_H_4_	638 ± 35 ^b^	**3c**	2-HO-3,5-(*t*Bu)_2_C_6_H_2_	**17.9 ± 1.3 ^b^**
**1h**	2-MeOC_6_H_4_	1064 ± 18 ^a^	**3d**	2-HO-3-*t*Bu-5-MeC_6_H_2_	**26.4 ± 3.4 ^a^**
**1i**	3-MeOC_6_H_4_	416 ± 1.5 ^a^	**3e**	2-HO-3-*i*Pr-6-MeC_6_H_2_	≥1000 ^c^
**1j**	4-MeOC_6_H_4_	≥2400 ^c^	**3f**	2-HO-4,6-(MeO)_2_C_6_H_2_	≥1000 ^c^
**2a**	2-HO-3-PhC_6_H_3_	**25.3 ± 6.9 ^a^**	**3g**	–	**32.0 ± 8.0 ^d^**
**2b**	2-HO-3-*t*BuC_6_H_3_	**24.0 ± 5.9 ^a^**	**4b**	4-HBAH	≥2400 ^c^
**2c**	2,4-(HO)_2_C_6_H_3_	939 ± 30 ^b^	control	4-HBA	≥2400 ^c^
**2d**	2-HO-5-BrC_6_H_3_	≥1000 ^c^	control	NaN_3_	2.72 ± 0.3 ^d^

The type of inhibition: ^a^ competitive; ^b^ uncompetitive; ^c^ not determined; ^d^ non-competitive.

## Supplementary Materials

The following are available online, Figure S1–S354: NMR spectra and 2D experiments of selected compounds **1–8**; Figure S355: The stability of laccase from *T. versicolor* in buffer-organic co-solvent media.
