# Supplementary material for: Synthesis and Structure-Activity Relationship Studies of Hydrazide-Hydrazones as Inhibitors of Laccase from Trametes versicolor
Source: Molecules. 2020 Mar 10;25(5):1255. doi: 10.3390/molecules25051255 (PMC7179439; doi:10.3390/molecules25051255)
Supplement: Supplementary file 1 [file molecules-25-01255-s001.pdf]

SUPPLEMENTARY MATERIALS

Synthesis and Structure-Activity Relationship Studies of Hydrazide-Hydrazones as Inhibitors of Laccase from *Trametes versicolor*

Halina Maniak [1,*], Michał Talma [2], Konrad Matyja [1], Anna Trusek [1] and Mirosław Giurg [3,*]

[1] Department of Bioprocess Engineering, Micro- and Nanoengineering, Faculty of Chemistry, Wroclaw University of Science and Technology, Wybrzeże Wyspiańskiego 27, Wrocław 50-370, Poland; <konrad.matyja@pwr.edu.pl) (K.M.), <anna.tusek@pwr.edu.pl> (A.T.)

[2] Department of Bioorganic Chemistry, Faculty of Chemistry, Wroclaw University of Science and Technology, Wybrzeże Wyspiańskiego 27, Wrocław 50-370, Poland; <michal.talma@pwr.edu.pl> (M.T.)

[3] Department of Organic and Medicinal Chemistry, Faculty of Chemistry, Wroclaw University of Science and Technology, Wybrzeże Wyspiańskiego 27, Wrocław 50-370, Poland

* Correspondence:
Tel.: +48-71-320-3314; fax: +48-71-328-1318/e-mail: halina.maniak@pwr.edu.pl (H.M.);
Tel.: +48-71-320-3616/ e-mail: miroslaw.giurg@pwr.edu.pl (M.G.)

List of Contents check page mumbering – may have changed with editing

1. Materials and General Methods S2
2. General procedure for the synthesis of benzoic acid hydrazides **4b–c** S2
3. Preparation of salicylic aldehydes **6a–b**, **6d–f**, **7a–b**, **7d–f** S3
4. Synthesis of benzoic acid methyl esters **8b** and **8c** S5
5. NMR spectra and 2D experiments of selected compounds **1–8** S5–S181
6. Laccase stability in organic solvents S182
7. References S183–S184

Molecules **2020**, *25*, 1255; doi:10.3390/molecules25051255 www.mdpi.com/journal/molecules

1. Materials and General Methods

Chemicals and solvents were purchased as pure "for synthesis" or "analytical grade" reagents from Sigma-Aldrich (St. Louis, MO, USA), ARMAR, and POCh and were used mostly without further purification. In particular, 4-hydroxybenzhydrazide (4a - 4-HBAH,), dimethylsulfoxide (DMSO), syringaldazine (SNG, 4-hydroxy-3,5-dimethoxybenzaldehyde azine), 2,6-bis(hydroxymethyl)-4-methylbenzene were purchased in Sigma-Aldrich; citric acid monohydrate, sodium phosphate dibasic dodecahydrate purchased in POCh (Poland), and were used without further purification. Laccase from *Trametes versicolor* was purchased in lyophilized powder from Sigma-Aldrich.

Methyl alcohol (CH_3OH) was distilled prior to condensation reaction from Mg element shavings in the presence of I_2. Diethyl ether (DEE) was distilled slowly over a mixture of $LiAlH_4$ and CaH_2 powders from the water bath. Hydrazide-hydrazones 1–3 were prepared by condensation of an equimolar mixture of an appropriate carboxylic acid hydrazide (4a - 4-HBAH, 4b, or 4c - 3-HBAH) with the appropriate unit of an aldehyde or their derivatives (5–7) in CH_3OH in the presence of the catalytic amount of AcOH, as given below. Analytical TLC was performed on PET foils precoated with silica gel (Merck silica gel, 60 F254), and were made visual under UV light (λ_{max} = 254 nm), or by staining with iodine vapor. Melting points were determined on an Electrothermal IA 91100 digital melting-point apparatus using the standard open capillary method. FT-IR spectra (4000–400 cm^{-1}) were recorded as KBr plates on a Perkin–Elmer 2000 FT-IR or on Bruker VERTEX 70V spectrometer using diamond ATR accessory. Absorption maxima are reported in wavenumbers (cm^{-1}). ^{1}H-NMR and ^{13}C-NMR spectra were recorded on a Jeol 400yh (399.78 for ^{1}H and 100.52 for ^{13}C) or on a Bruker Avance 600 Spectrometer (600.58 for ^{1}H and 151.03 for ^{13}C) at 295 K. Chemical shifts (δ) are given in parts per million (ppm) downfield relative to TMS, and coupling constants (*J*) are in Hz. Residual solvent central signals were recorded as follows: DMSO-d_6, δ_H = 2.50, δ_C = 39.43; $\underline{CH_3}OH$-d_4, δ_H = 3.31, δ_C = 49.05; $CDCl_3$, δ_H = 7.263, δ_C = 77.00. When measured, signals of DEPT experiment were referred to (+) or (–). High-resolution mass spectra (HRMS) were recorded on a Waters LCD Premier XE instrument, and only the $[M + H]^+$ or $[M + Na]^+$ molecular species are reported. Purity and homogeneity of known hydrazide-hydrazones **1–3**, 4-methoxybenzhydrazide (**4b**), 3-hydroxybenzhydrazide (**4c**), and aldehydes **6–7** were confirmed by measuring their melting points, FT-IR, ^{1}H-NMR, and ^{13}C-NMR spectra and/or HRMS and compared them with literature data [1–6,8–10,12–14,16–17,19–24,26–28,30–31]. All new hydrazide-hydrazones **1b**, **1d**, **2a–b**, **2e–f**, **2h**, and **3a–g,** were fully characterized. The positions of hydrogen and carbon atoms in the NMR data were determined by supporting the standard dept-135 and ATP experiments and by the 2D HMQC, HMBC, NOESY experiments map analysis, if measured.

2. General procedure for the synthesis of benzoic acid hydrazides 4b–c

The synthesis procedure was adapted from the literature [7]. To a solution of substituted benzoic acid methyl ester (10 mmol) in dry CH_3OH (25 mL), $H_2NNH_2 \times H_2O$ (98%, 1.0 mL, 20 mmol) was added. The reaction mixture was stirred and carried out under gentle reflux for two days. Reaction mixture was concentrated before crystallization to obtain the hydrazides **4b–c**.

4-Methoxybenzohydrazide (**4b**): The general procedure starting from 4-methoxybenzoic acid methyl ester (**8b**, 1.66 g, 10 mmol) was employed. Colorless powder; 1.42 g, 8.5 mmol, 85% yield; m.p.: 136–138 °C (from CH_3OH) (m.p.: 136 °C [8]); ^{1}H-NMR (DMSO-d_6, 400 MHz): δ 9.62 (s, 1H, NH), 7.81 (d, ^3J = 9.0 Hz, 1H, H-2,6), 6.97 (d, ^3J = 9.0 Hz, 1H, H-3,5), 4.43 (s, 2H, NH_2), 3.79 (s, 3H, OMe) ppm; ^{13}C-NMR (DMSO-d_6, 100 MHz): δ 165.61 (C=O), 161.39 (C-4), 128.68 (C-2,6), 125.45 (C-1), 113.47 (C-3,5), 55.23 (OMe) ppm. ^{1}H-NMR and ^{13}C-NMR data are consistent with literature values [8].

3-Hydroxybenzohydrazide (**4c**): The general procedure starting from 3-hydroxybenzoic acid methyl ester (**8c**, 1.52 g, 10 mmol) was employed. Colorless powder; 1.02 g, 6.5 mmol, 67% yield; m.p.: 157.5–159.5 °C (from CH_3OH) (m.p.: 157–159 °C [9]); ^{1}H-NMR (DMSO-d_6, 400 MHz): δ 9.66 (s, 1H, OH), 9.48 (s, 1H, NH), 7.18–7.24 (m, 3H, ArH), 6.85–6.92 (m, 1H, ArH), 4.45 (s, 2H, NH_2) ppm; ^{13}C-NMR (DMSO-d_6, 100

MHz): δ 165.97 (C=O), 157.25 (C), 134.72 (C), 129.27 (CH), 117.91 (CH), 117.34 (CH), 114.02 (CH) ppm. ^{1}H-NMR and ^{13}C-NMR data are consistent with literature values [10].

3. Preparation of salicylic aldehydes 6a–b, 6d–f, 7a–b, 7d–f

3-Phenylsalicylic aldehyde (**6a**). The salicylic aldehyde **6a** was obtained from 2-hydroxybiphenyl via 2-(methoxymethoxy)-1,1′-biphenyl regiospecific LICTMEDA (*n*-butyllithium in the presence of *N,N,N′,N′*-tetramethylethylenediamine) metallation in dry DEE and by formylation of the formed metaloorganic intermediate with DMF according to the literature procedure [11]. Pale yellowish crystals; 82% overall yield; m.p.: 47.5–48.5 °C (m.p.: 47–48 °C [12]); selected FT-IR (ATR) ν_{max}/cm^{-1}: 3210 (O-H), 3076 (C-H), 3054 (C-H), 3030 (C-H), 2829 (CHO), 2740 (CHO), 1670 (C=O), 1609, 1451, 1432, 1303, 1264 (C-O), 1213, 1065, 1026, 762, 721, 695, 651, 530; ^{1}H-NMR ($CDCl_3$, 400 MHz): δ 11.55 (s, 1H, OH), 9.96 (s, 1H, CHO), 7.63 (dd, ^{3}J = 7.5 Hz, ^{4}J = 1.7 Hz, 1H, ArH), 7.61 (dd, ^{3}J = 8.0 Hz, ^{4}J = 1.5 Hz, 2H, PhH-2,6), 7.57 (dd, ^{3}J = 7.7 Hz, ^{4}J = 1.7 Hz, 1H, ArH), 7.46 (dd, ^{3}J = 8.0 Hz, ^{3}J = 7.4 Hz, 2H, PhH-3,5), 7.39 (tt, ^{3}J = 7.4 Hz, ^{4}J = 1.5 Hz, 1H, PhH-4), 7.12 (dd, ^{3}J = 7.7 Hz, ^{3}J = 7.5 Hz, 1H, ArH-5) ppm; ^{13}C-NMR ($CDCl_3$, 150 MHz): δ 196.80 (CHO), 158.85 (C), 137.78 (CH), 136.26 (C), 133.16 (CH), 130.45 (C), 129.23 (2 × CH), 128.76 (2 × CH), 127.64 (CH), 120.84 (C), 119.89 (CH) ppm. ^{13}C-NMR data are consistent with literature value [13].

3-tert-Butylsalicylic aldehyde (**6b**). The salicylic aldehyde **6b** was obtained from 2-*tert*-butylphenol via 2-(methoxymethoxy)-1-*tert*-butyl-benzene via regiospecyfic metallation with LICTMEDA (*n*-butyllithium in the presence of *N,N,N′,N′*-tetramethylethylenediamine) in dry DEE in the 6-position and formylation of the formed intermediate with DMF according to literature procedure [11]. Pale transparent oil; 70% overall yield; b.p.: 147–148 °C (b.p.: 147.0–148.5 °C [14]); ^{1}H-NMR ($CDCl_3$, 400 MHz): δ 11.80 (d, ^{5}J = 0.6 Hz, 1H, OH), 9.88 (s, 1H, CHO), 7.54 (ddd, ^{3}J = 7.7 Hz, ^{4}J = 1.7 Hz, ^{5}J = 0.6 Hz, 1H, H-4), 7.40 (dd, ^{3}J = 7.7 Hz, ^{4}J = 1.7 Hz, 1H, H-6), 6.96 (dd, ^{3}J = 7.7 Hz, ^{3}J = 7.7 Hz, 1H, H-5), 1.43 (s, 9H, 3 × CH_3) ppm; ^{13}C-NMR ($CDCl_3$, 100 MHz): δ 197.12 (CHO), 161.17 (C-2), 138.19 (C-3), 134.07 (C-4), 131.95 (C-6), 120.59 (C-1), 119.17 (C-5), 34.80 (C – *t*-Bu), 29.15 (3 × CH_3) ppm.

5-Bromosalicylic aldehyde (**6d**). The salicylic aldehyde **6d** was obtained with Reimer-Thiemann formylation of 4-bromophenol according to the literature procedure [15] adapted to the laboratory gram scale. Pale needles; m.p.: 104.5–105.5 °C (m.p.: 104–105 °C [16]); selected FT-IR (ATR) ν_{max}/cm^{-1}: 3225 (br, O-H), 3068 (C-H), 3042 (C-H), 2880 (CHO), 1670 (C=O), 1652 (C=C), 1464, 1271 (C-O), 1153, 1114, 892, 828, 766, 694 (C-Br), 626, 538, 448, 424; ^{1}H-NMR ($CDCl_3$, 400 MHz): δ 10.93 (d, ^{5}J = 0.4 Hz, 1H, OH), 9.84 (d, ^{5}J = 0.6 Hz, 1H, CHO), 7.67 (dd, ^{4}J = 2.5 Hz, ^{5}J = 0.3 Hz, 1H, H-6), 7.60 (ddd, ^{3}J = 8.9 Hz, ^{4}J = 2.5 Hz, ^{5}J = 0.4 Hz, 1H, H-4), 6.91 (ddd, ^{3}J = 8.9 Hz, ^{5}J = 0.6 Hz, ^{5}J = 0.3 Hz, 1H, H-3) ppm; ^{13}C-NMR ($CDCl_3$, 100 MHz): δ 195.41 (CHO), 160.50 (C-2), 139.66 (C-4), 135.60 (C-6), 121.69 (C-1), 119.77 (C-3), 11.32 (C-5) ppm. The ^{1}H-NMR data are consistent with literature values [16].

6-Methoxysalicylic aldehyde (**6e**). The salicylic aldehyde **6e** was prepared from 1,3-dimethoxybenzene via regiospecific metallation with LICTMEDA (*n*-butyllithium in the presence of *N,N,N′,N′*-tetramethylethylenediamine) in the 2-position, DMF formylation of the formed intermediate, and by $AlCl_3$ monodemethylation [17] adapted to the laboratory gram scale. Colorless powder; 62% overall yield; m.p.: 67–69 °C (m.p.: 68–70 °C [17]); selected FT-IR (ATR) ν_{max}/cm^{-1}: 2500–3300 (br, OH), 3035 (C-H), 2985 (C-H), 2953 (C-H), 2892 (CHO), 2849 (CHO), 1638 (C=C), 1615 (C=O), 1579, 1461, 1447, 1307, 1237 (br, C-O), 1199, 1161, 1085 (C-O), 832, 756, 702, 652, 476; ^{1}H-NMR ($CDCl_3$, 400 MHz): δ 11.98 (s, 1H, OH), 10.34 (d, ^{4}J = 0.7 Hz, 1H, CHO), 7.41 (dd, ^{3}J = 8.5 Hz, ^{3}J = 8.3 Hz, 1H, H-4), 6.53 (ddd, ^{3}J = 8.5 Hz, ^{4}J = 0.8 Hz, ^{4}J = 0.7 Hz, 1H, H-3), 6.37 (dd, ^{3}J = 8.3 Hz, ^{4}J = 0.8 Hz, 1H, H-5), 3.90 (s, 3H, OMe) ppm; ^{13}C-NMR ($CDCl_3$, 100 MHz): δ 194.31 (CHO), 163.58 (C-2), 162.44 (C-6), 138.38 (C-4), 110.78 (C-1), 109.81 (C-3), 100.92 (C-5), 55.77 (OMe) ppm. ^{13}C-NMR data are consistent with literature values [17].

2,6-Dimethoxybenzaldehyde (**6f**). The dimethoxybenzaldehyde **6f** was prepared from 1,3-dimethoxy-benzene via regiospecific metallation with LICTMEDA (*n*-butyllithium in the presence of stoichiometric amount of *N,N,N′,N′*-tetramethylethylenediamine) in anhydrous THF at the 2-position followed by DMF formylation following a literature procedure [18]. Pale prisms; 86% yield; m.p.: 95–97 °C (m.p.: 95.5–97.0 °C [19]); selected FT-IR (ATR) ν_{max}/cm^{-1}: 3015 (C-H), 2979 (C-H), 2956 (C-H), 2889

(C-H), 2847 (CHO), 2798 (CHO), 1671 (C=O), 1590 (C=C), 1578 (C=C), 1480, 1461, 1419, 1254 (C-O), 1109 (C-O), 819, 776, 715, 645, 559, 496; ^{1}H-NMR (DMSO-d_6, 400 MHz): δ 10.51 (s, 1H, CHO), 7.44 (t, ^3J = 8.5 Hz, 1H, H-5), 6.57 (d, ^3J = 8.5 Hz, 2H, H-4,6), 3.89 (s, 6H, OMe) ppm; ^{13}C-NMR (DMSO-d_6, 100 MHz): δ 189.38 (CHO), 152.13 (C-2,6), 135.89 (C-4), 114.23 (C-1), 103.79 (C-3,5), 55.99 (2 × OMe) ppm. The ^{1}H- and ^{13}C-NMR data are consistent with literature values [19].

3-Hydroxymethyl-5-methylsalicylic aldehyde (**7a**). The salicylic aldehyde **7a** was prepared by MnO_2 oxidation of 2,6-bis(hydroxymethyl)-*p*-cresol in acetone according to a procedure described in [20]. Colorless crystals; m.p.: 72–73 °C (m.p.: 70–72 °C [21]); selected FT-IR (ATR) ν_{max}/cm^{-1}: 3284 (O-H), 3141 (O-H), 2942 (C-H), 2899 (C-H), 2855 (CHO), 1640 (C=O), 1603 (C=C), 1454, 1378, 1320, 1251 (C-O), 1215, 1161, 1077, 1042 (C-O), 992, 870, 739, 718, 538, 470; ^{1}H-NMR ($CDCl_3$, 400 MHz): δ 11.17 (s, 1H, Ar-OH), 9.85 (s, 1H, CHO), 7.40 (d, ^4J = 2.3 Hz, 1H, H-4), 7.29 (d, ^4J = 2.3 Hz, 1H, H-6), 4.72 (s, 2H, CH_2), 2.33 (s, 3H, Me), 2.30 (s, 1H, $CH_2\underline{OH}$) ppm; ^{13}C-NMR ($CDCl_3$, 100 MHz): δ 196.58 (CHO), 155.37 (C-2), 135.32 (C-4), 130.73 (C-6), 130.31 (C-3), 128.23 (C-5), 120.33 (C-1), 57.14 (CH_2), 19.93 (Me) ppm. The ^{1}H-NMR and ^{13}C-NMR data are consistent with literature values [20] and [21], respectively.

5-Hydroxymethyl-3-methylsalicylic aldehyde (**7b**). The aldehyde **7b** was prepared as colorless needles in a moderate 41% yield by formaldehyde hydroxymethylation of 3-methylsalicylic aldehyde according to a literature procedure [22]; m.p.: 82–83 °C (m.p.: 83 °C [22]); selected FT-IR (ATR) ν_{max}/cm^{-1}: 3264 (br, O-H), 2956 (C-H), 2925 (C-H), 2864 (CHO), 1647 (C=O), 1607 (C=C), 1450, 1383, 1321, 1262 (C-O), 1208, 1152, 1032 (C-O), 992, 953, 868, 711, 606, 550, 479; ^{1}H-NMR ($CDCl_3$, 400 MHz): δ 11.25 (s, 1H, Ar-OH), 9.87 (s, 1H, CHO), 7.40 (s, 2H, H-4,6), 4.65 (s, 2H, CH_2), 2.28 (s, 3H, Me), 1.74 (br s, 1H, OH) ppm; ^{13}C-NMR ($CDCl_3$, 100 MHz): δ 196.61 (CHO), 159.55 (C-2), 136.95 (C-4), 131.85 (C-5), 129.64 (C-6), 127.22 (C-3), 119.65 (C-1), 64.36 (CH_2), 15.05 (Me) ppm. The ^{1}H-NMR data are consistent with literature value [22].

3-tert-Butyl-5-methylsalicylic aldehyde (**7d**). The aldehyde **7d** was prepared in 86% yield via 2-*tert*-butyl-4-methylphenol monoformylation with paraformaldehyde according to a literature procedure [23] . Colorless prisms; m.p.: 77–78 °C (m.p: 74–75 °C [23]); selected FT-IR (ATR) ν_{max}/cm^{-1}: 2600–3300 (br, O-H), 3002 (C-H), 2960 (C-H), 2913 (C-H), 2865 (C-H), 2839 (CHO), 1644 (C=O), 1617 (C=C), 1597, 1463, 1439, 1355, 1320, 1264, 1229 (C-O), 1211, 1159, 971, 866, 745, 708, 517, 408; ^{1}H-NMR ($CDCl_3$, 400 MHz): δ 11.61 (d, ^5J = 0.6 Hz, 1H, OH), 9.83 (s, 1H, CHO), 7.34 (d, ^4J = 2.2 Hz, 1H, ArH), 7.18 (dd, ^4J = 2.2 Hz, ^5J = 0.6 Hz, 1H, ArH), 2.33 (s, 3H, Me), 1.42 (s, 9H, *t*-Bu) ppm; ^{13}C-NMR ($CDCl_3$, 150 MHz): δ 197.05 (CHO), 159.12 (C), 137.95 (C), 135.37 (CH), 131.41 (CH), 128.12 (C), 120.35 (C), 34.72 (C), 29.21 (3 × CH_3), 20.55 (Me) ppm. The ^{1}H-NMR data are consistent with literature value [24].

3-Isopropyl-6-methylsalicylic aldehyde (**7e**). Synthesis of '*ortho*-formylthymol' **7e** was performed by *ortho* formylation of thymol according to a procedure described in [25]. Transparent oil; 67% yield; b.p.: 261 °C (b.p.: 261 °C at 760 mmHg [26]); ^{1}H-NMR ($CDCl_3$, 400 MHz): δ 12.30 (s, 1H, OH), 10.31 (s, 1H, CHO), 7.32 (d, ^3J = 7.7 Hz, 1H, H-4), 6.68 (d, ^3J = 7.7 Hz, 1H, H-5), 3.33 (sept, ^3J = 6.9 Hz, 1H, $\underline{CH}(CH_3)_2$), 2.58 (s, 3H, Me), 1.22 (d, ^3J = 6.9 Hz, 6H, $CH(\underline{CH_3})_2$) ppm; ^{13}C-NMR ($CDCl_3$, 100 MHz): δ 195.64 (CHO), 160.82 (C-2), 139.19 (C-6), 135.29 (C-3), 133.93 (C-4), 121.36 (C-5), 117.97 (C-1), 25.96 ($\underline{CH}(CH_3)_2$), 22.22 ($CH(\underline{CH_3})_2$), 17.88 (Me) ppm. ^{1}H-NMR and ^{13}C-NMR data are generally consistent with literature values [27] with the discrepancy of the formyl carbon signal reported at 95.6 ppm.

4,6-Dimethoxysalicylic aldehyde (**7f**). Salicylic aldehyde **7f** was prepared via 3,5-dimethoxyphenol isopropyl carbamate by direct *ortho* lithiation followed by DMF formylation by a literature procedure [28]. Colorles crystals; m.p.: 73–74 °C (m.p.: 72 °C [28]); selected FT-IR (ATR) ν_{max}/cm^{-1}: 2500–3300 (br, O-H), 3024 (C-H), 2985 (C-H), 2898 (C-H), 2843 (CHO), 2767 (CHO), 1641 (C=C), 1617 (C=O), 1581, 1476, 1424, 1373, 1335, 1301 (C-O), 1218 (C-O), 1155, 1108 (C-O), 1043, 936, 803, 789, 600, 515, 463; ^{1}H-NMR ($CDCl_3$, 400 MHz): δ 12.52 (s, 1H, OH), 10.09 (s, 1H, CHO), 6.01 (d, ^4J = 2.2 Hz, 1H, H-3), 5.91 (d, ^4J = 2.2 Hz, 1H, H-5), 3.85 (s, 3H, 6-OMe), 3.83 (s, 3H, 4-OMe) ppm; ^{13}C-NMR ($CDCl_3$, 100 MHz): δ 191.81 (CHO), 168.10 (C-4), 166.29 (C-2), 163.50 (C-6), 105.94 (C-1), 92.85 (C-3), 90.51 (C-5), 55.68 (2 × OMe) ppm. The ^{1}H-NMR and ^{13}C-NMR data are consistent with the literature values [28].

4. Synthesis of benzoic acid methyl esters 8b and 8c

General procedure: The synthesis was adapted from a literature procedure [29] (see Section 4.1.1.). To a mixture of substituted benzoic acid **9a–b** (0.10 mmol) in dry CH_3OH (140 mL), $SOCl_2$ was added slowly at water-ice bath (0–5 °C) temparature. The solution was stirred for 2 days under gentle reflux (ca. +65 °C), and then the solvent was evaporated under 20 mmHg pressure. The residue was repeatedly evaporated with toluene until the smell of residual $SOCl_2$ disappears (2 × 60 mL). The crude product was filtered through silica gel (70–230 mesh, 100g) eluted with $CHCl_3$ to obtain the 4-methoxy- or 3-hydroxy-benzoic acid methyl esters **8b** and **8c**, respectively.

4-Methoxybenzoic acid methyl ester (**8b**). The general procedure starting from 4-methoxybenzoic acid (**9b**, 15.2 g, 0.10 mmol) and $SOCl_2$ (1.0 mL, 14 mmol) was employed to obtain methyl ester **8b**. Colorless solid; 15.5 g, 0.093 mol, 93% yield; m.p.: 46–47 °C (from DEE) (m.p.: 48–49 °C [30]). The crude oil product was used in the hydrazide **4c** synthesis without further purification.

3-Hydroxybenzoic acid methyl ester (**8c**). The general procedure starting from 3-hydroxybenzoic acid (**9c**, 13.8 g, 0.10 mmol) and $SOCl_2$ (5.0 mL, 69 mmol) was employed to obtain methyl ester **8c**. Colorless solids; 13.1 g, 0.086 mol, 86% yield; m.p.: 70–71 °C (m.p: 69–71 °C [31]); ^{1}H-NMR ($CDCl_3$, 400 MHz): δ 7.58–7.62 (m, 2H, ArH), 7.31 (dd, ^3J = 8.1 Hz, ^3J = 8.1 Hz, 1H, ArH), 7.08 (ddd, ^3J = 8.1 Hz, ^4J = 2.6 Hz, ^4J = 1.1 Hz, 1H, ArH), 6.23 (s br, 1H, OH), 3.92 (s, 3H, OMe) ppm; ^{13}C-NMR ($CDCl_3$, 100 MHz): δ 167.75 (C=O), 156.06 (C), 131.04 (C), 129.70 (CH), 121.71 (CH), 120.50 (CH), 116.36 (CH), 52.43 (OMe) ppm. ^{1}H-NMR and ^{13}C-NMR data are consistent with literature values [31].

5. NMR spectra and 2D experiments of selected compounds 1–8

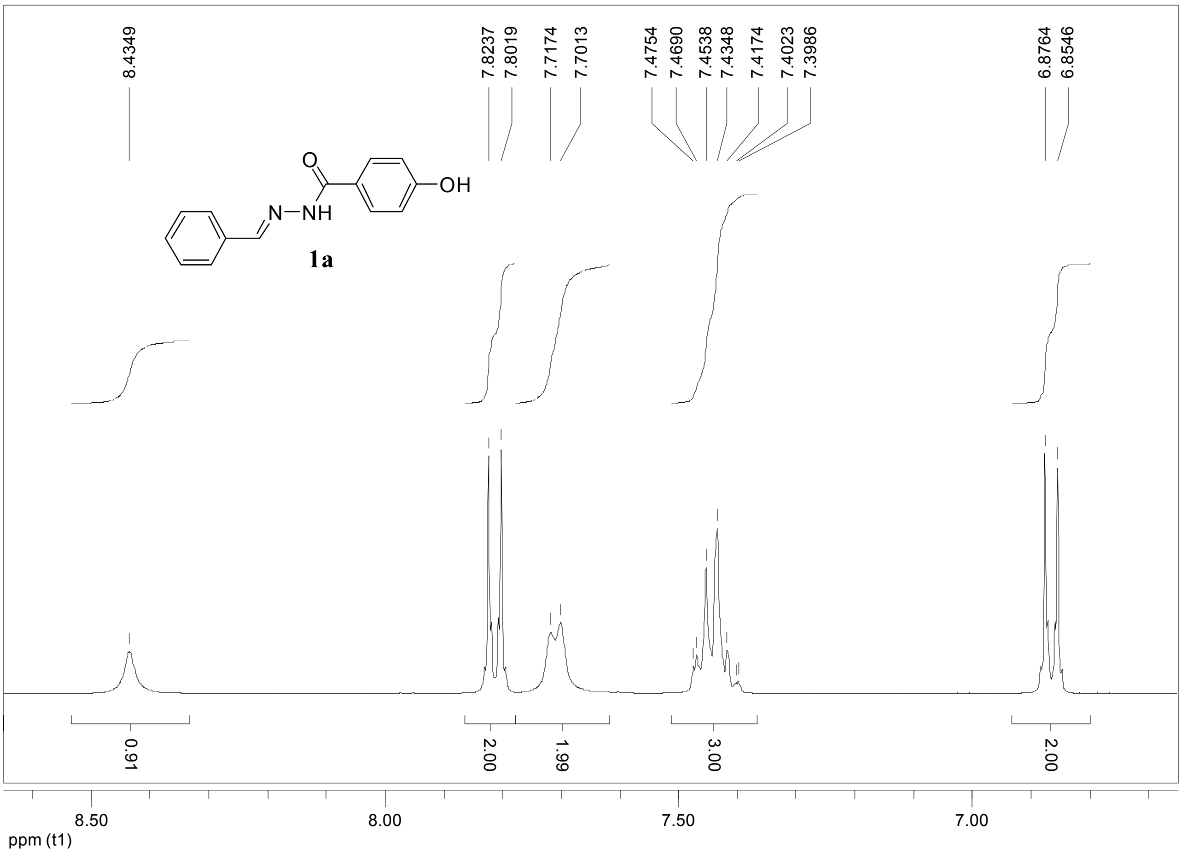

Figure S1. Expansion of the ^{1}H-NMR (400 MHz, DMSO-d_6) spectrum of compound **1a**.

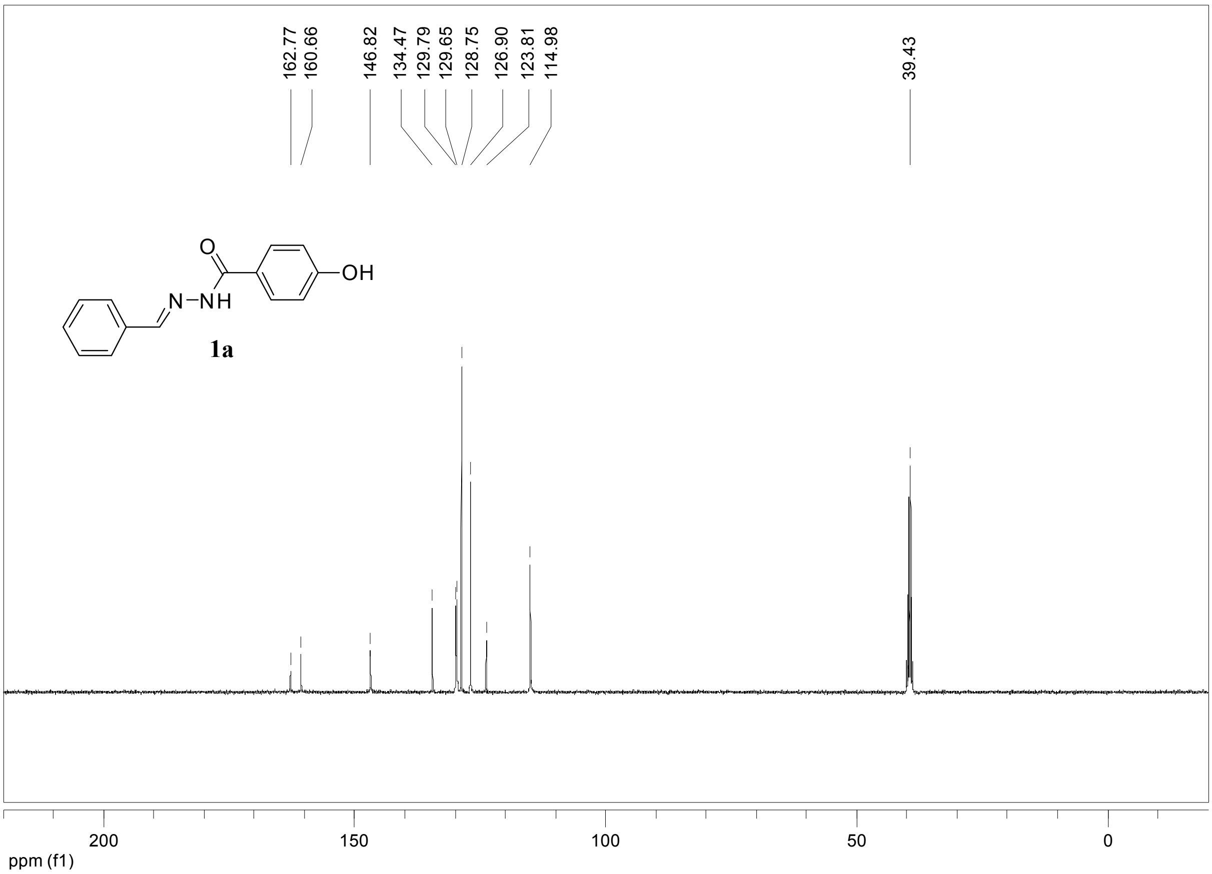

Figure S2. ^{13}C-NMR (100 MHz, DMSO-d_6) spectrum of compound **1a**.

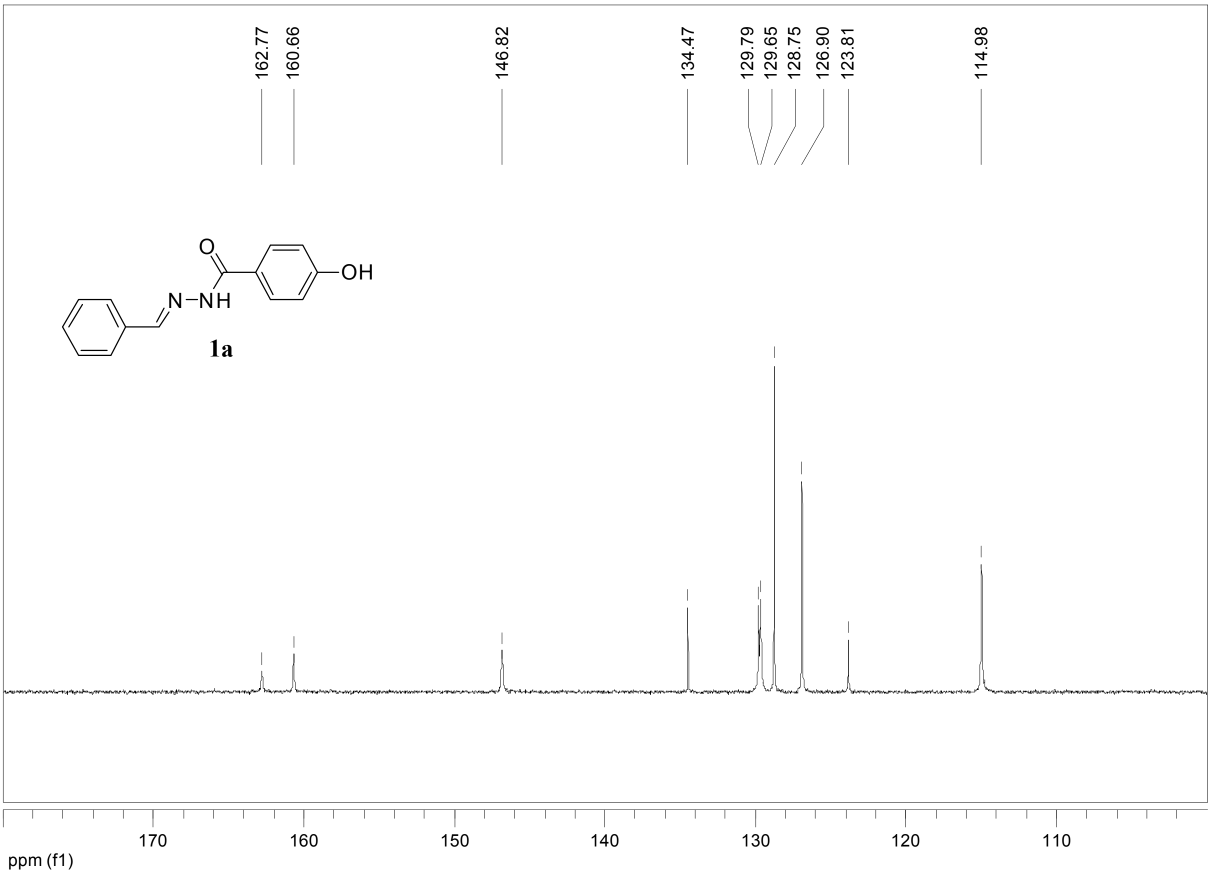

Figure S3. Expansion of ^{13}C-NMR (100 MHz, DMSO-d_6) spectrum of compound **1a**.

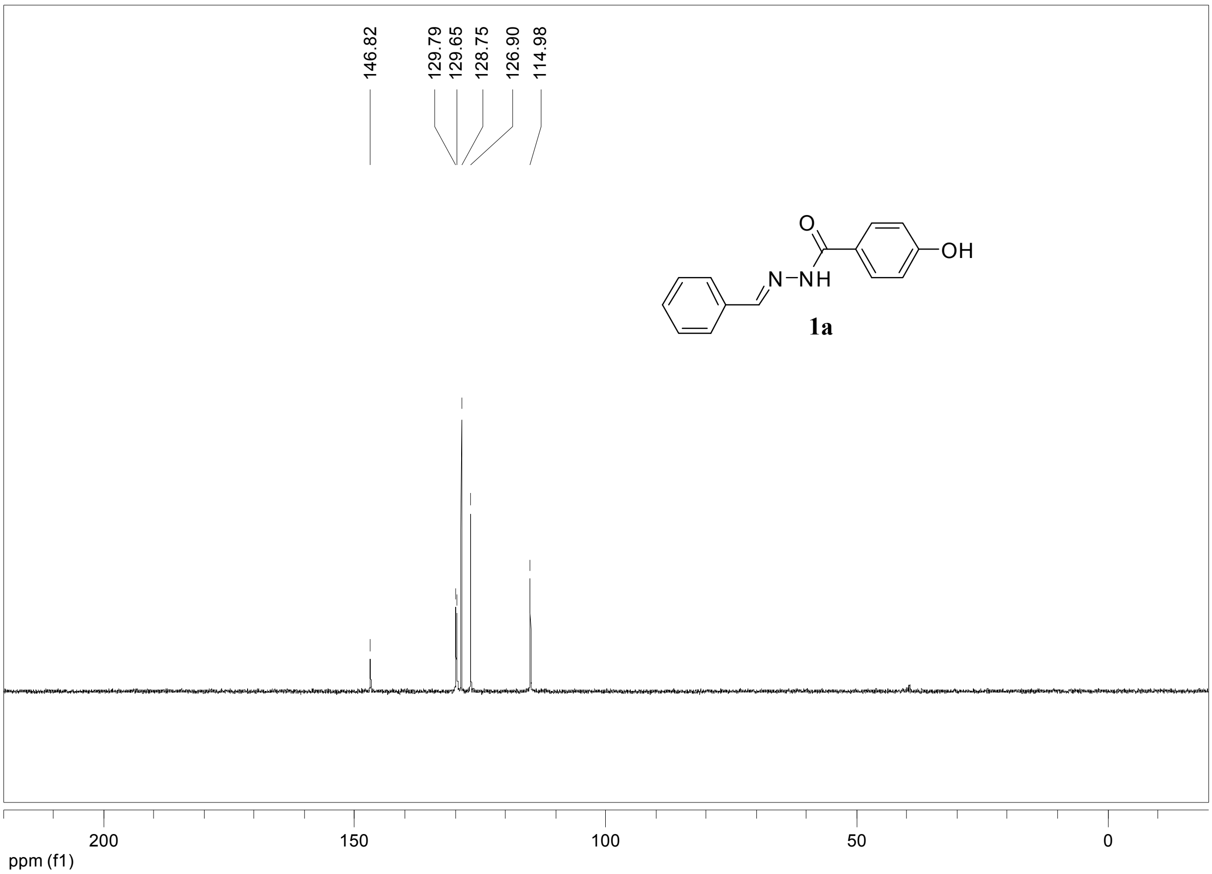

Figure S4. ^{13}C-NMR (100 MHz, DMSO-d_6) experiment dept-135 of compound **1a**.

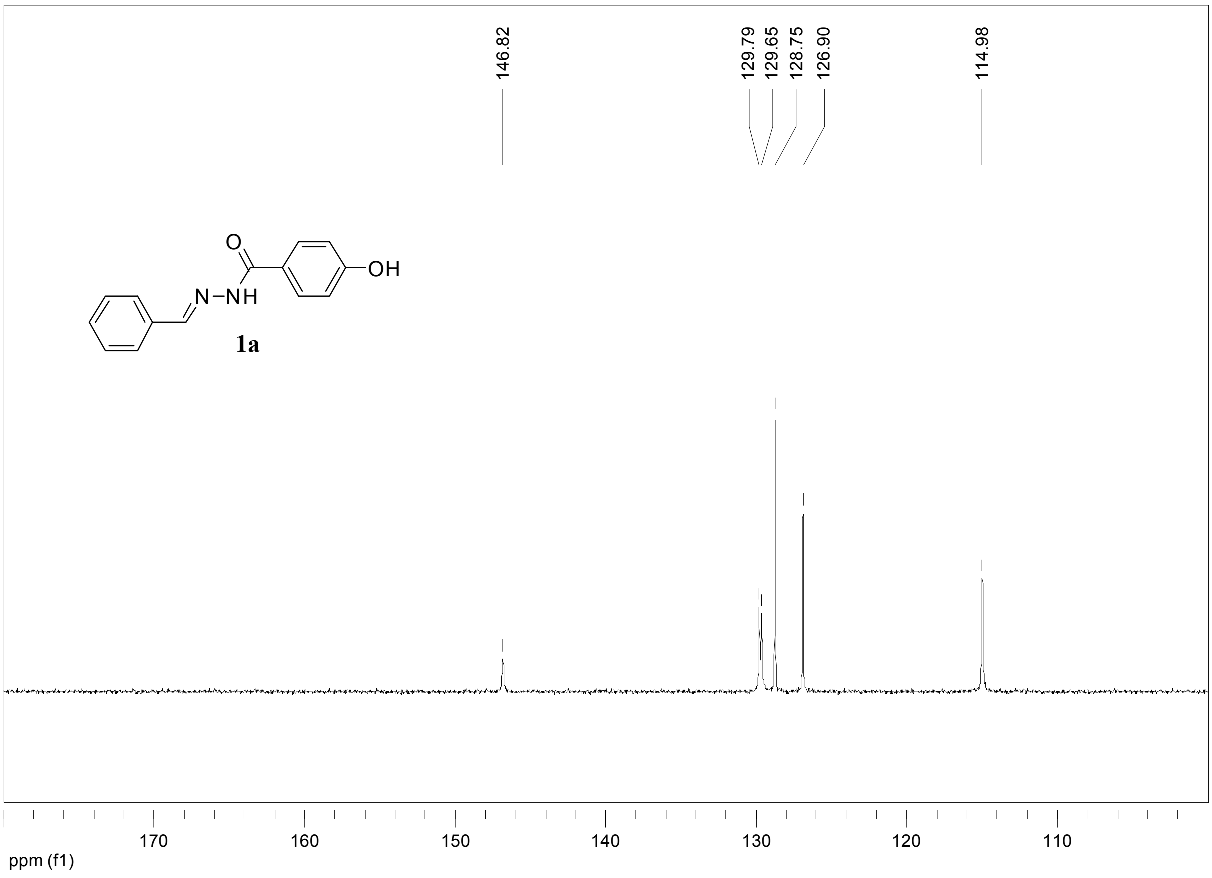

Figure S5. Expansion of ^{13}C-NMR (100 MHz, DMSO-d_6) experiment dept-135 of compound **1a**.

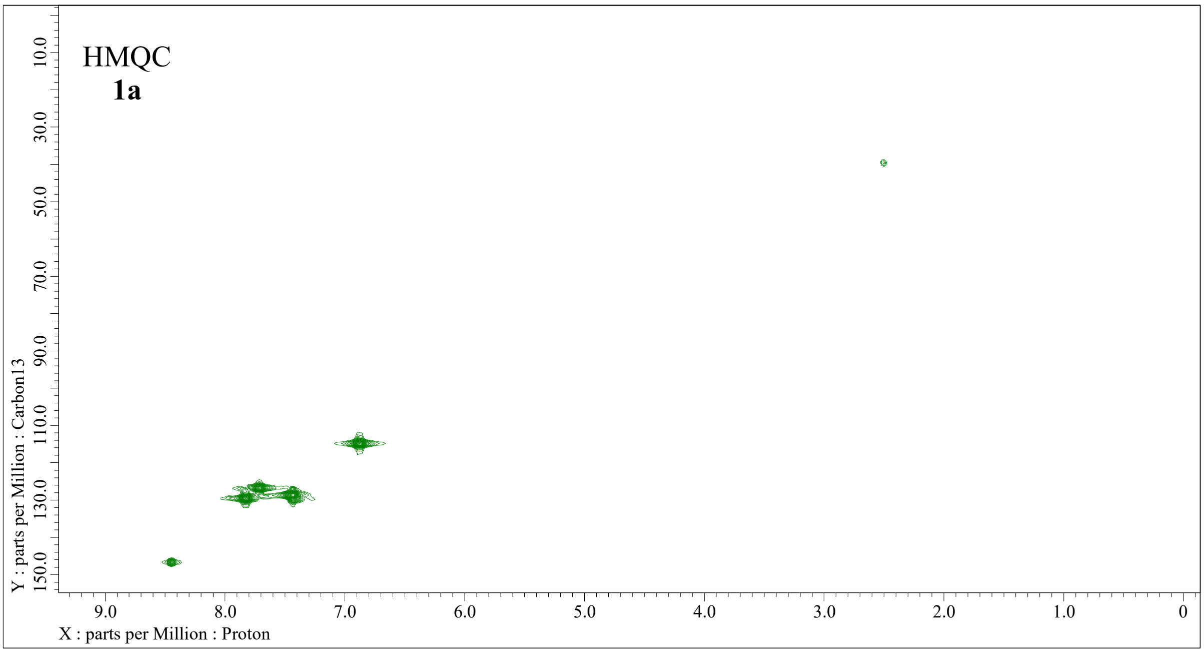

Figure S6. 2D-NMR (400 MHz, DMSO-d_6) HMQC experiment of 4-hydroxy-*N*'-[(*E*)-benzylidene]-benzohydrazide (**1a**).

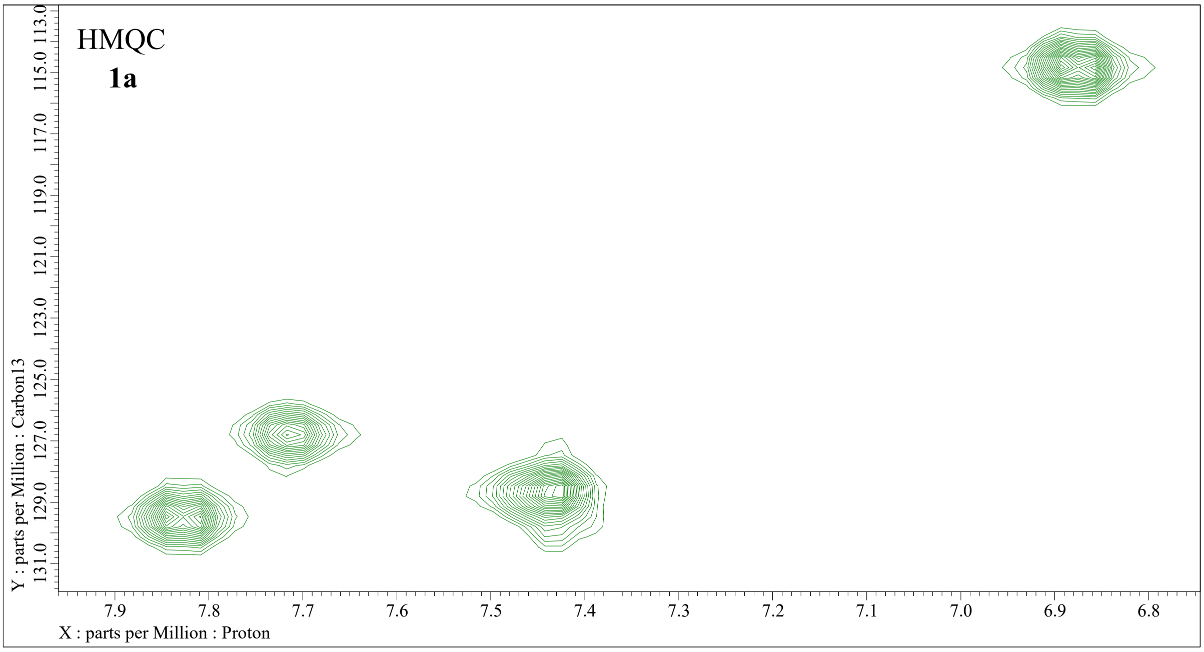

Figure S7. Expansion of 2D-NMR (400 MHz, DMSO-d_6) HMQC experiment of 4-hydroxy-*N*'-[(*E*)-benzylidene]benzohydrazide (**1a**).

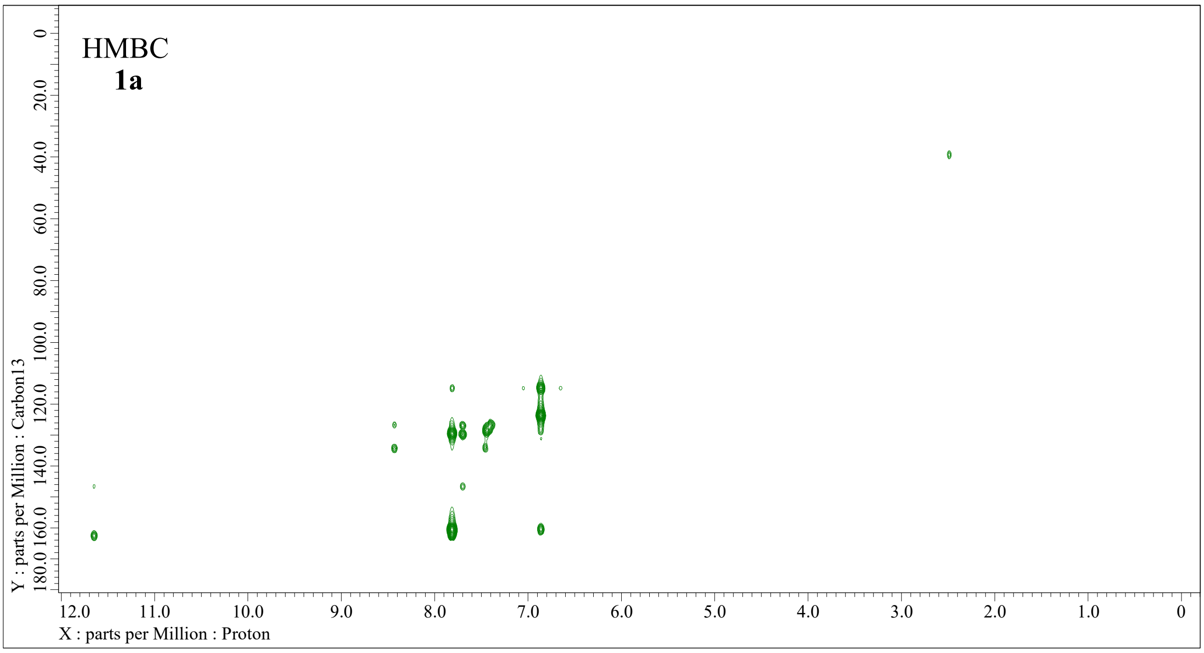

Figure S8. 2D-NMR (400 MHz, DMSO-d_6) HMBC experiment of 4-hydroxy-*N*'-[(*E*)-benzylidene]benzohydrazide (**1a**).

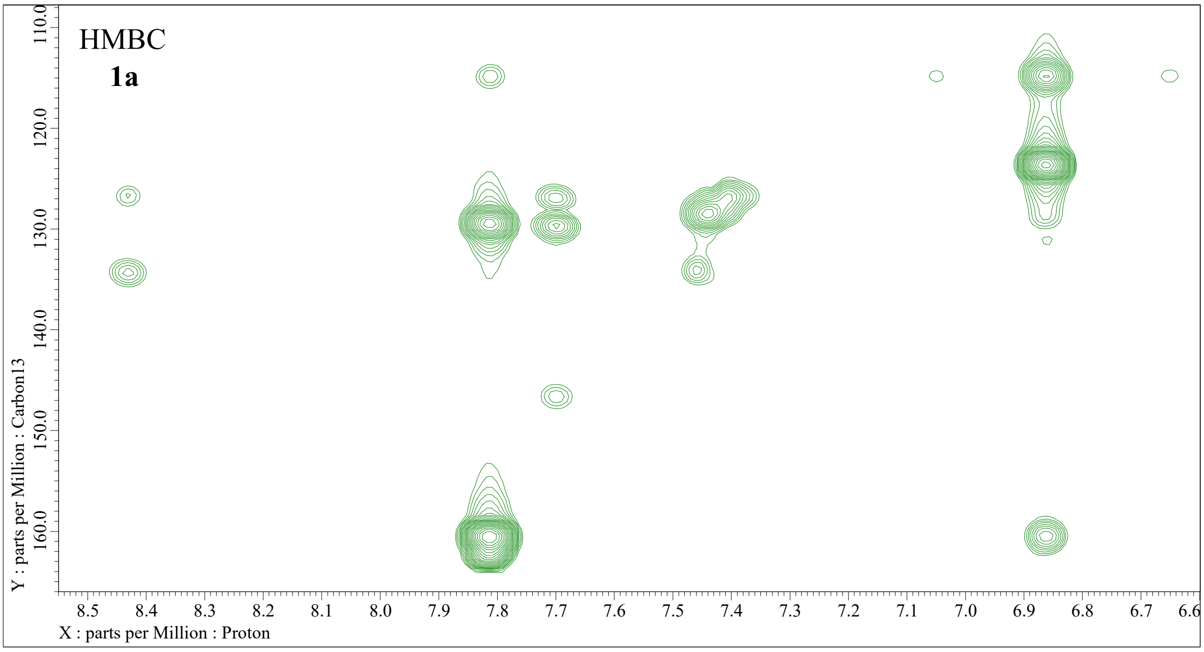

Figure S9. Expansion of 2D-NMR (400 MHz, DMSO-d_6) HMBC experiment of 4-hydroxy-*N*'-[(*E*)-benzylidene]benzohydrazide (**1a**).

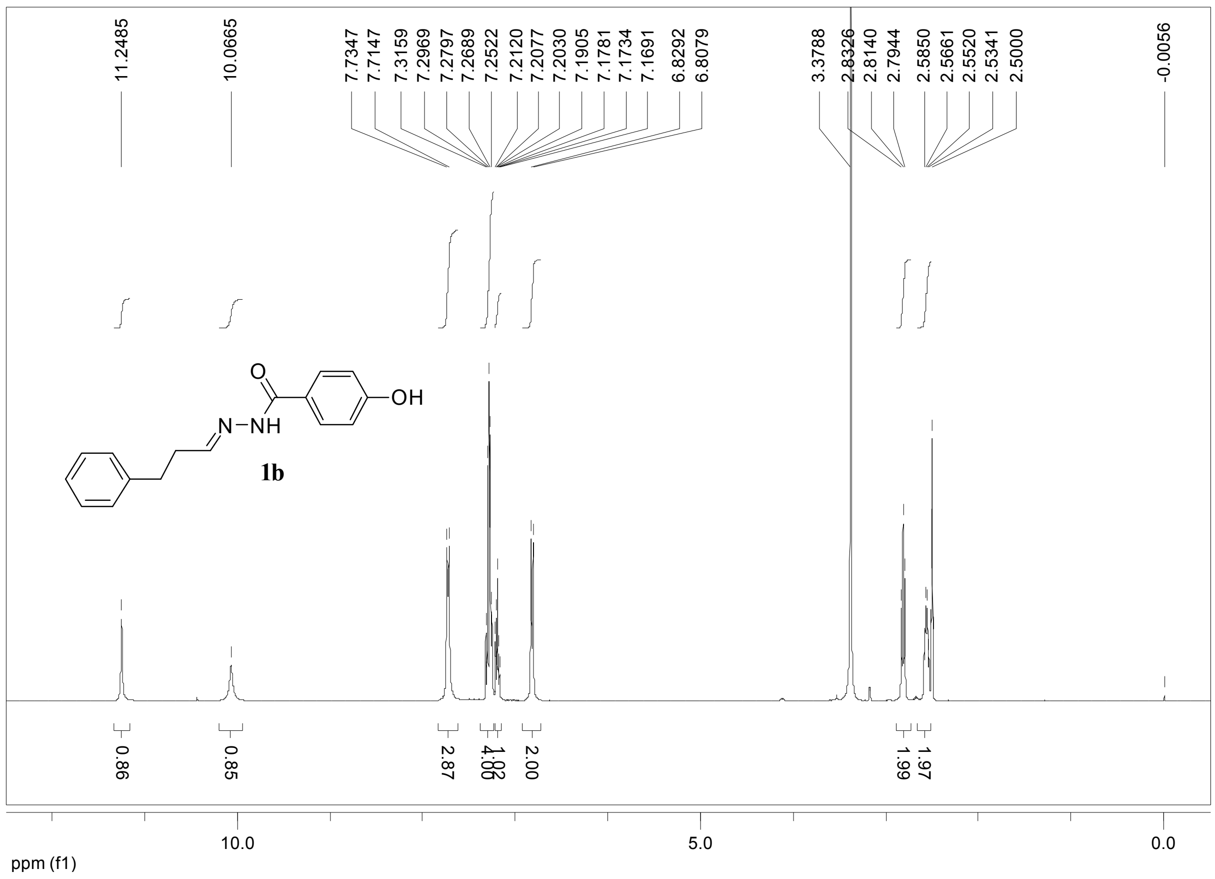

Figure S10. ^{1}H-NMR (400 MHz, DMSO-d_6) spectrum of compound **1b**.

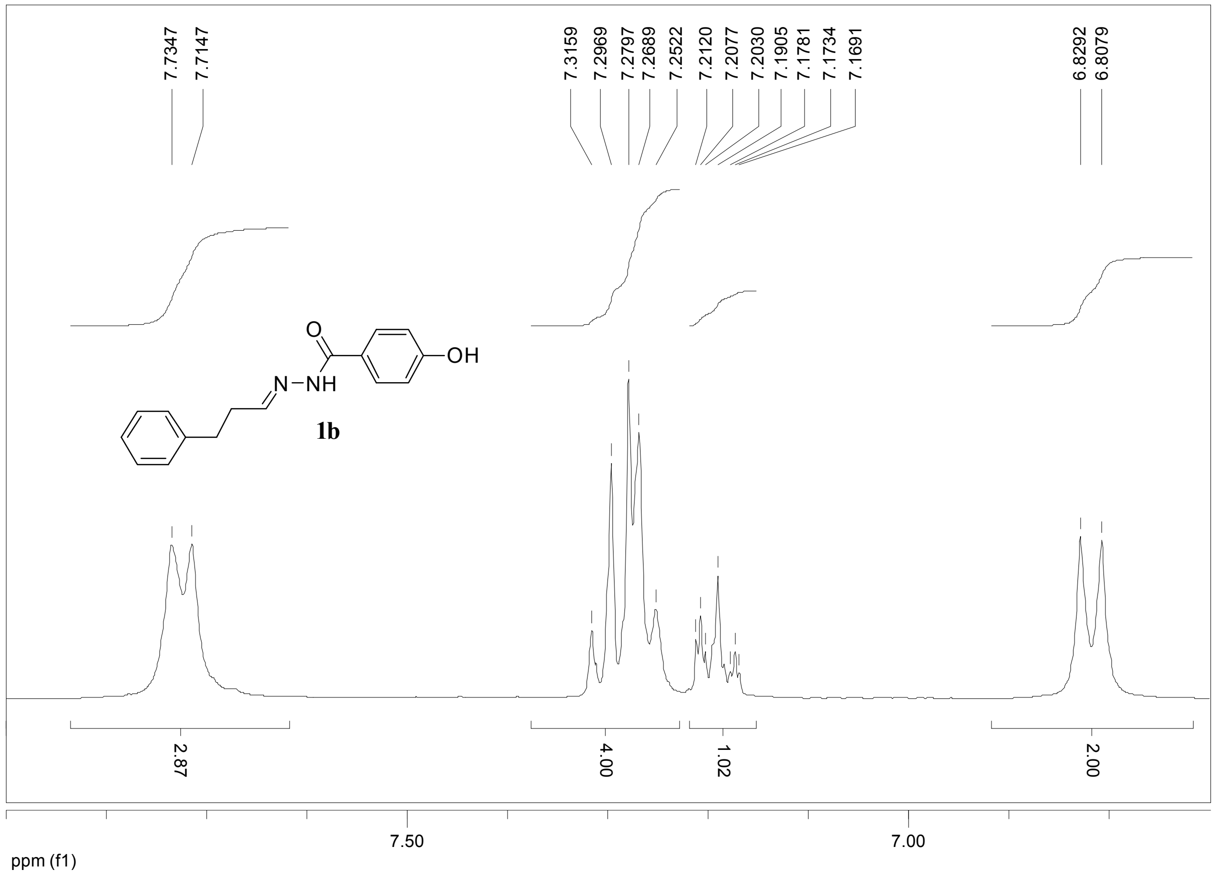

Figure S11. Expansion of ^{1}H-NMR (400 MHz, DMSO-d_6) spectrum of compound **1b**.

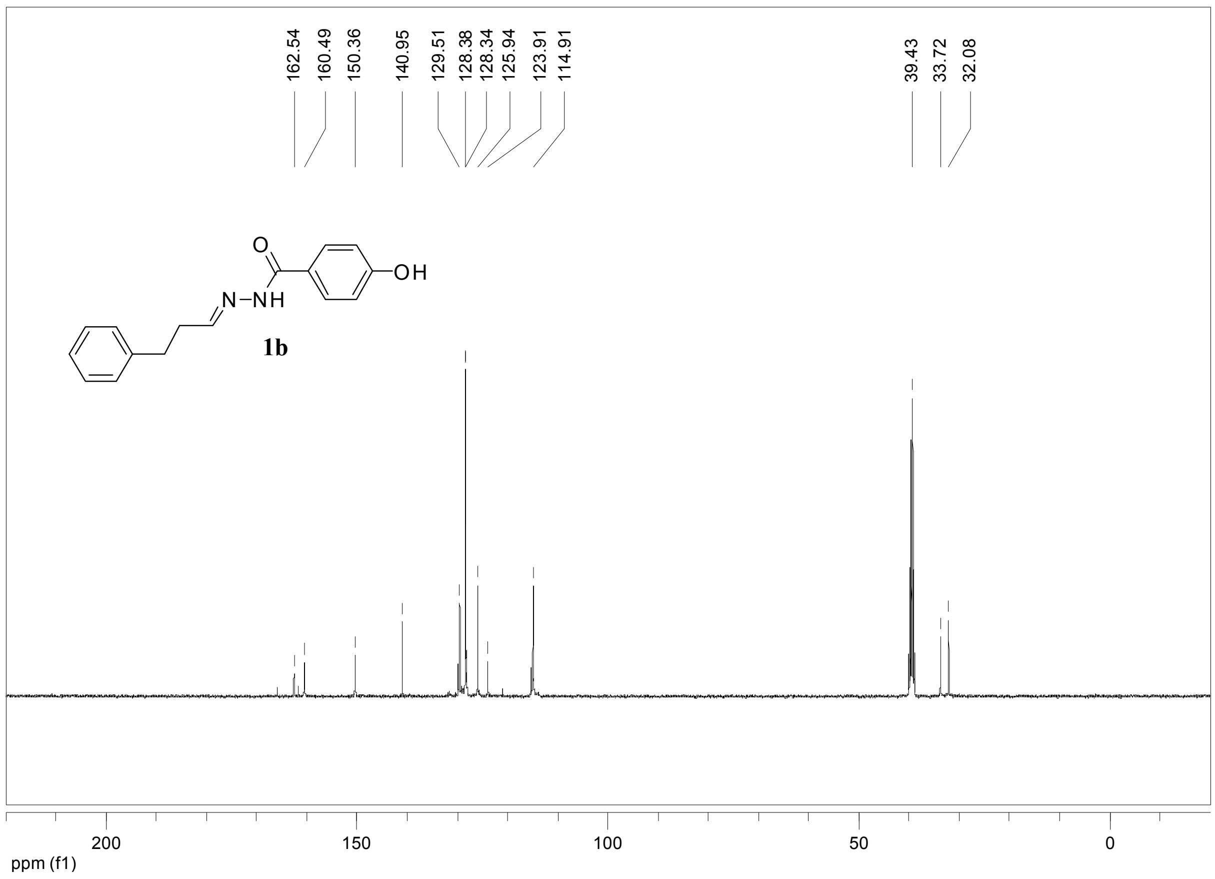

Figure S12. ^{13}C-NMR (100 MHz, DMSO-d_6) spectrum of compound **1b**.

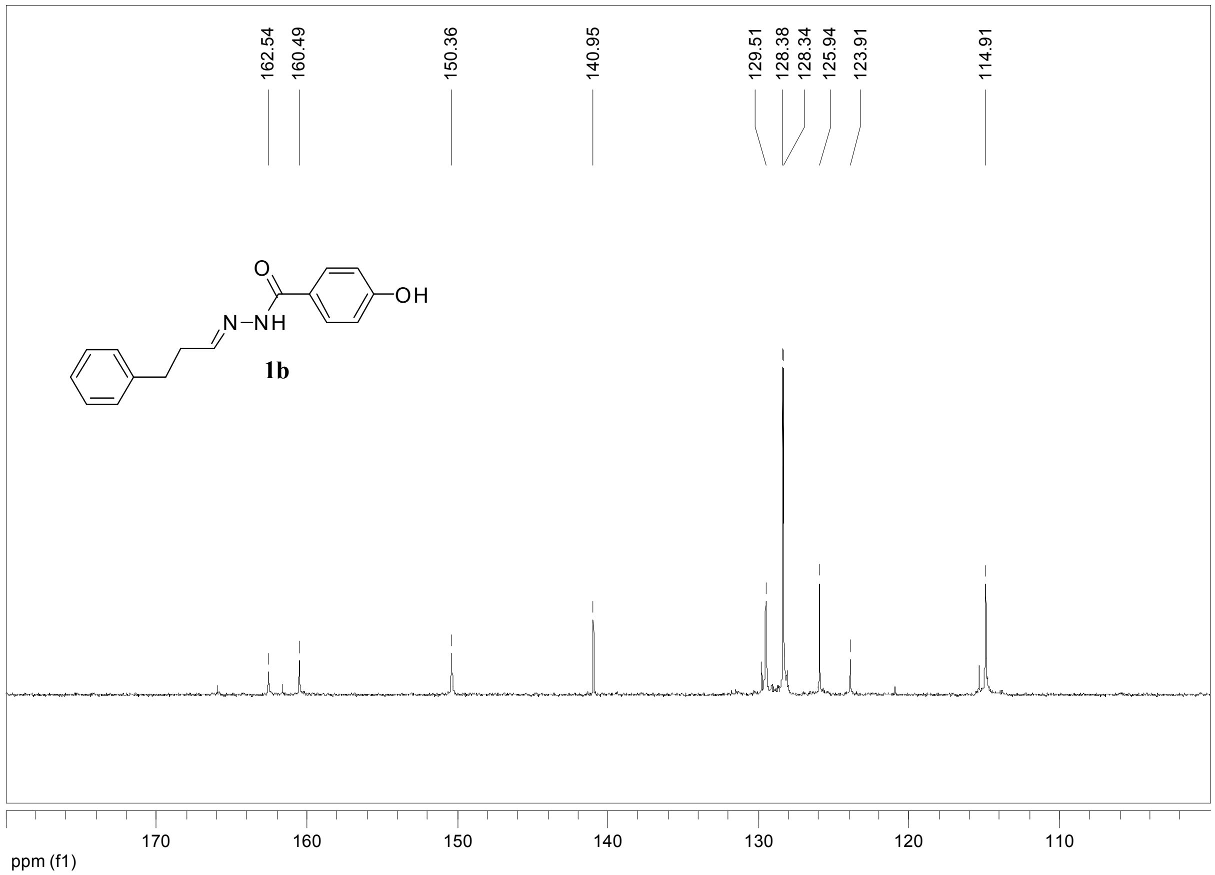

Figure S13. Expansion of ^{13}C-NMR (100 MHz, DMSO-d_6) spectrum of compound **1b**.

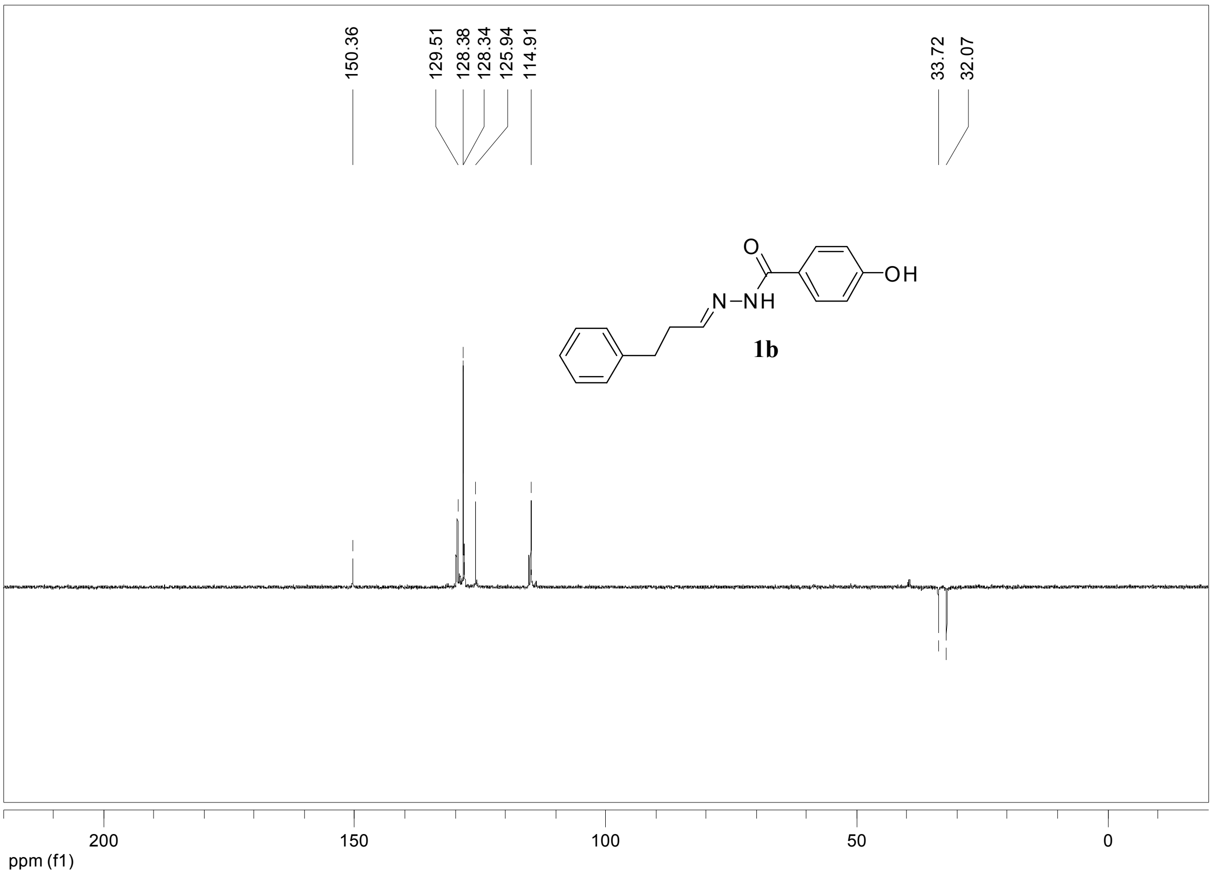

Figure S14. ^{13}C-NMR (100 MHz, DMSO-d_6) dept-135 experiment of compound **1b.**

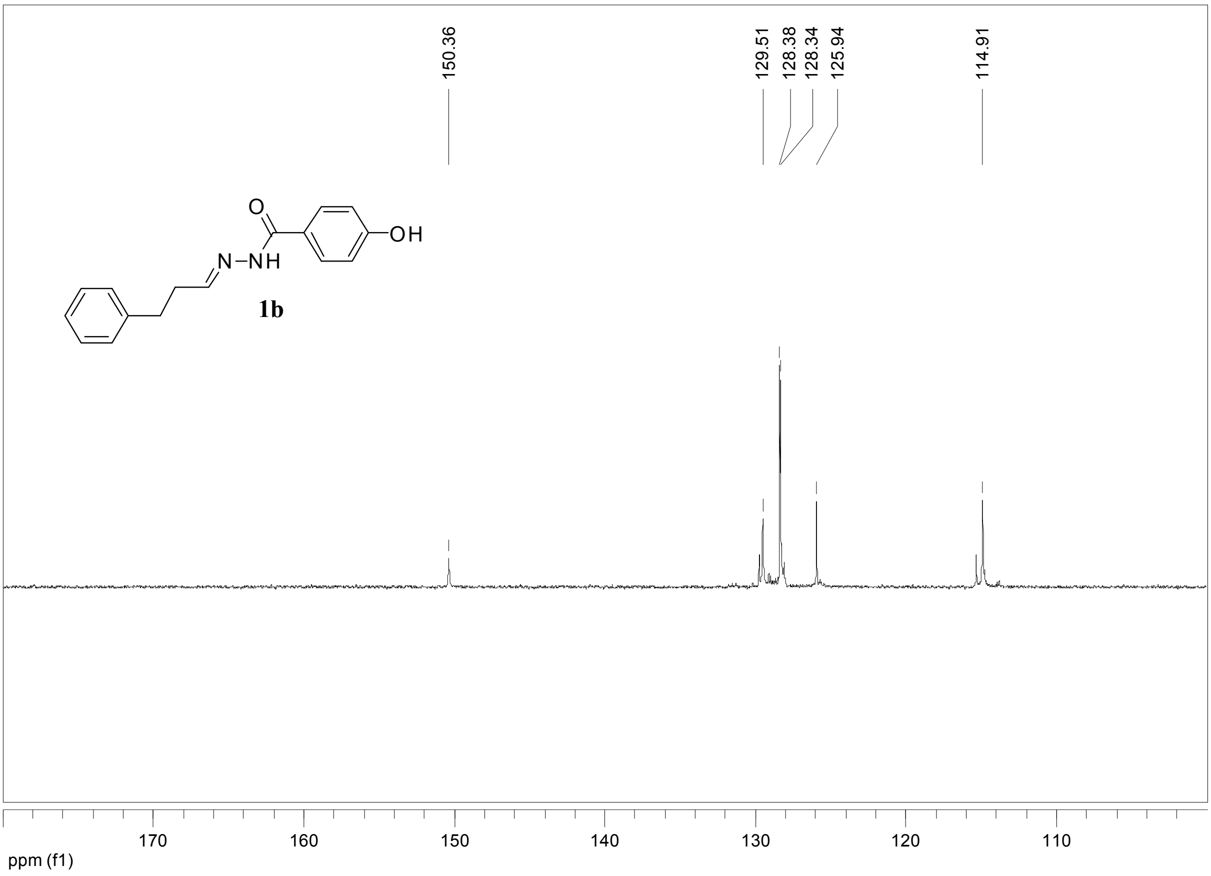

Figure S15. Expansion of ^{13}C-NMR (100 MHz, DMSO-d_6) dept-135 experiment of compound **1b**.

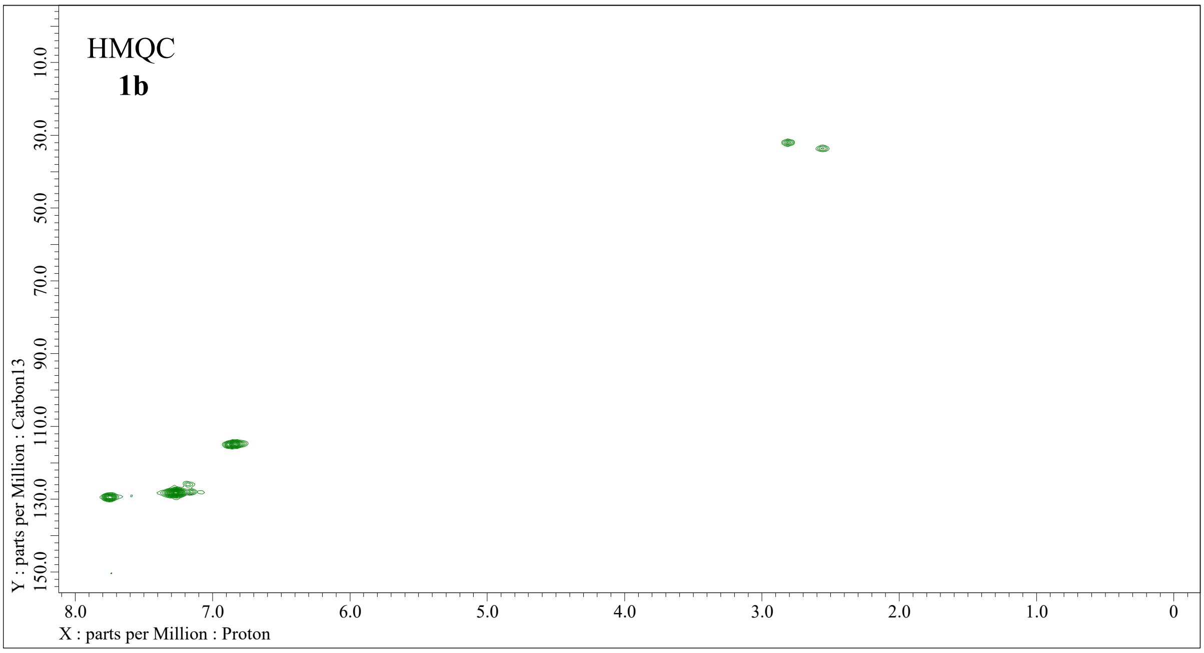

Figure S16. 2D-NMR (400 MHz, DMSO-d_6) HMQC experiment of 4-hydroxy-*N*'-[(1*E*)-3-phenylpropylidene]benzohydrazide (**1b**).

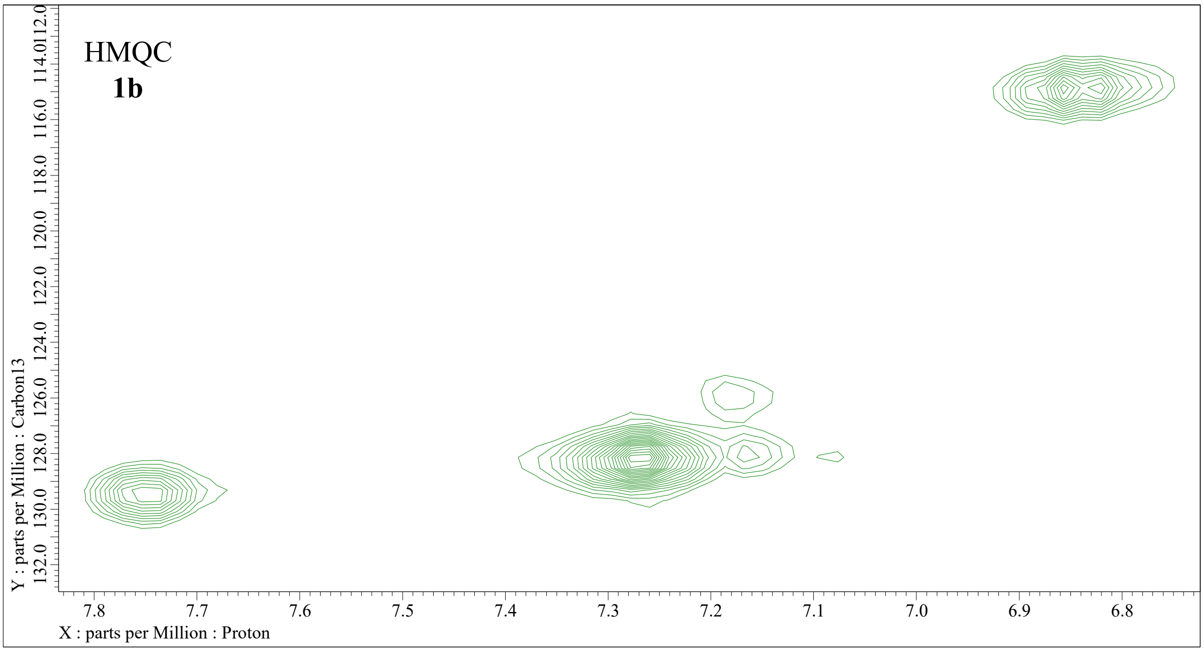

Figure S17. Expansion of 2D-NMR (400 MHz, DMSO-d_6) HMQC experiment of 4-hydroxy-*N*'-[(1*E*)-3-phenylpropylidene]benzohydrazide (**1b**).

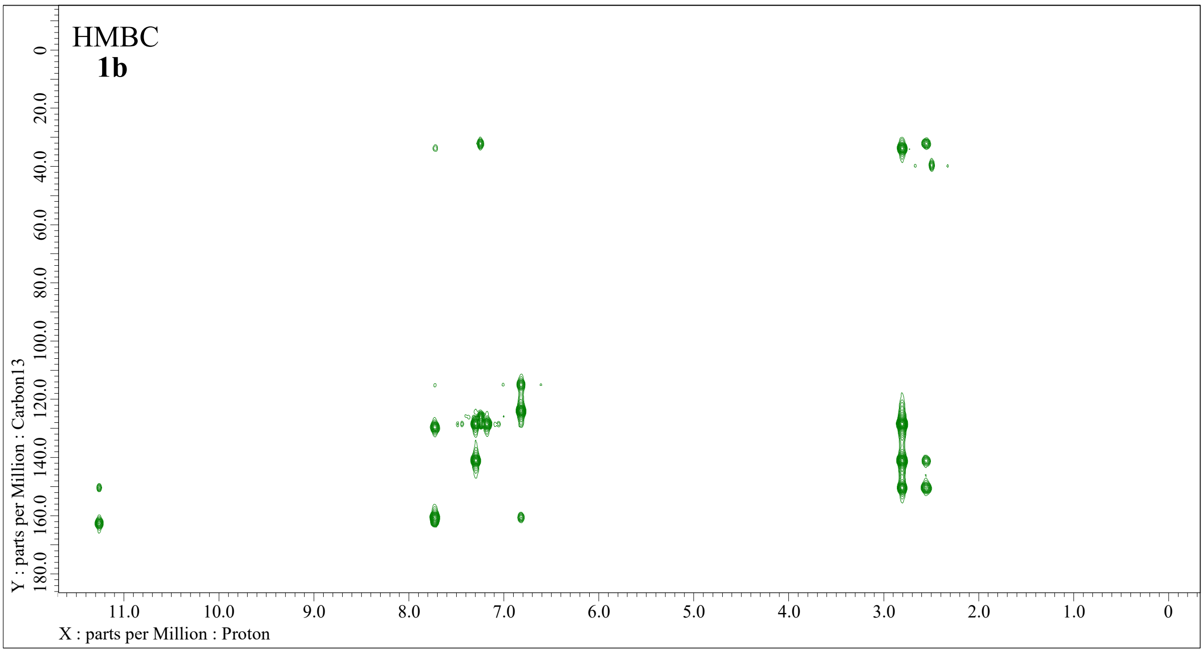

Figure S18. 2D-NMR (400 MHz, DMSO-*d*6) HMBC experiment of 4-hydroxy-*N*'-[(1*E*)-3-phenylpropylidene]benzohydrazide (**1b**).

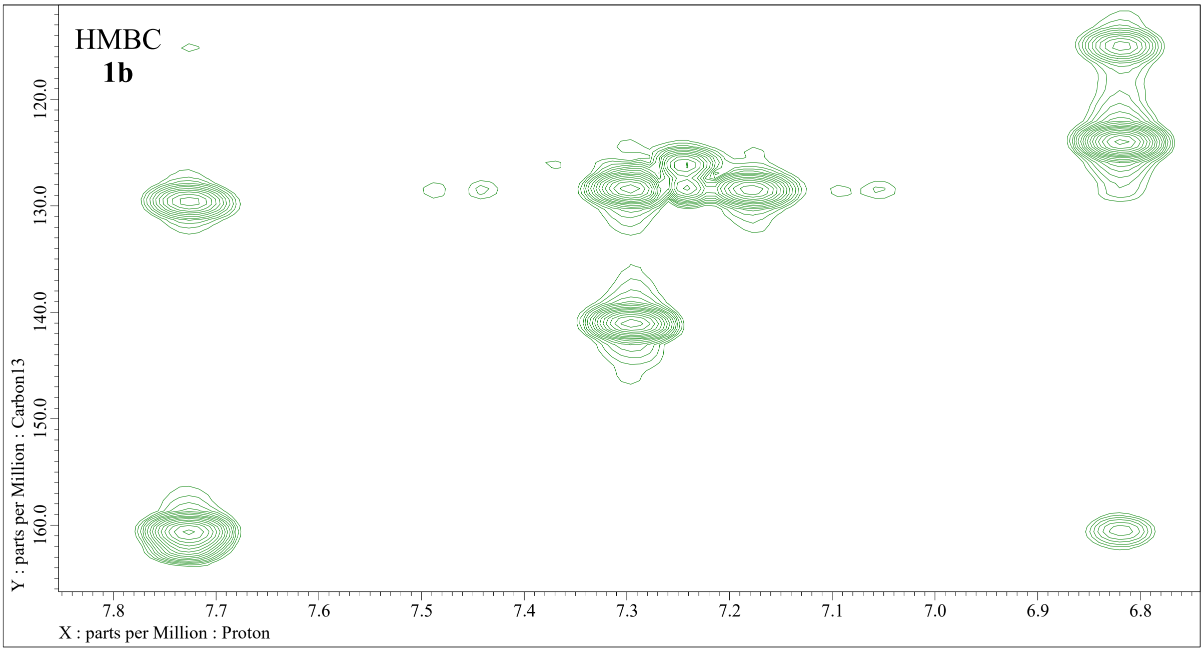

Figure S19. Expansion of 2D-NMR (400 MHz, DMSO-*d*6) HMBC experiment of 4-hydroxy-*N*'-[(1*E*)-3-phenylpropylidene]benzohydrazide (**1b**).

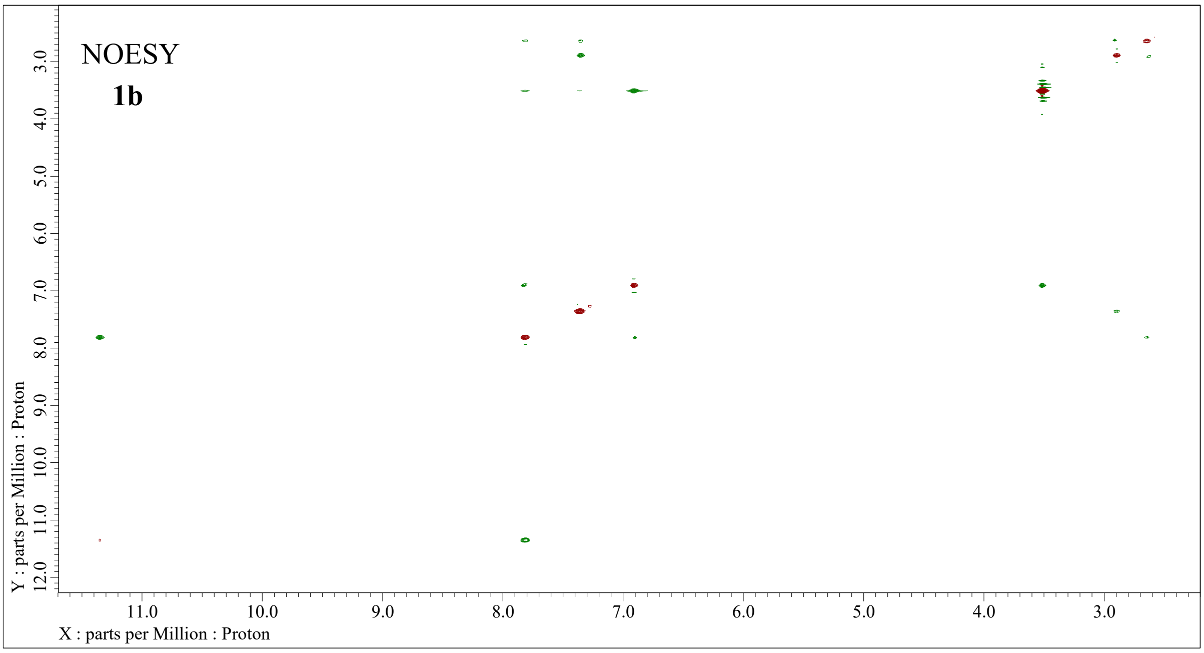

Figure S20. ^{1}H-^{1}H NMR (400 MHz, DMSO-d_6) NOESY experiment of 4-hydroxy-*N*'-[(1*E*)-3-phenylpropylidene]benzohydrazide (**1b**).

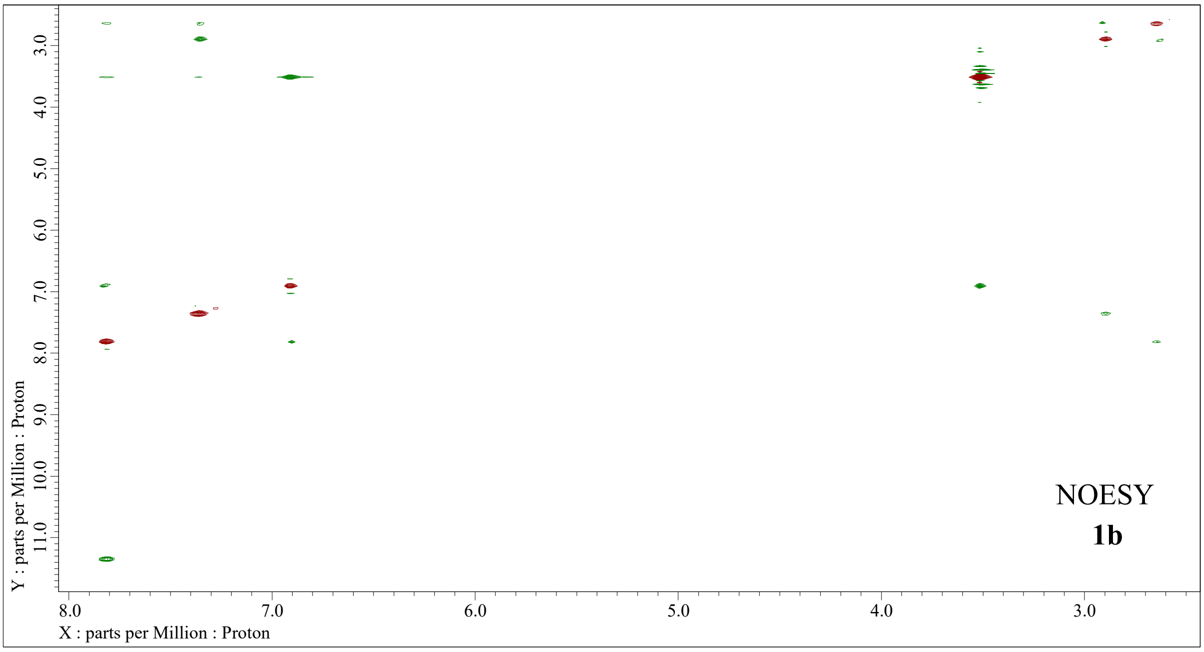

Figure S21. Expansion of ^{1}H-^{1}H NMR (400 MHz, DMSO-d_6) NOESY experiment of 4-hydroxy-*N*'-[(1*E*)-3-phenylpropylidene]benzohydrazide (**1b**).

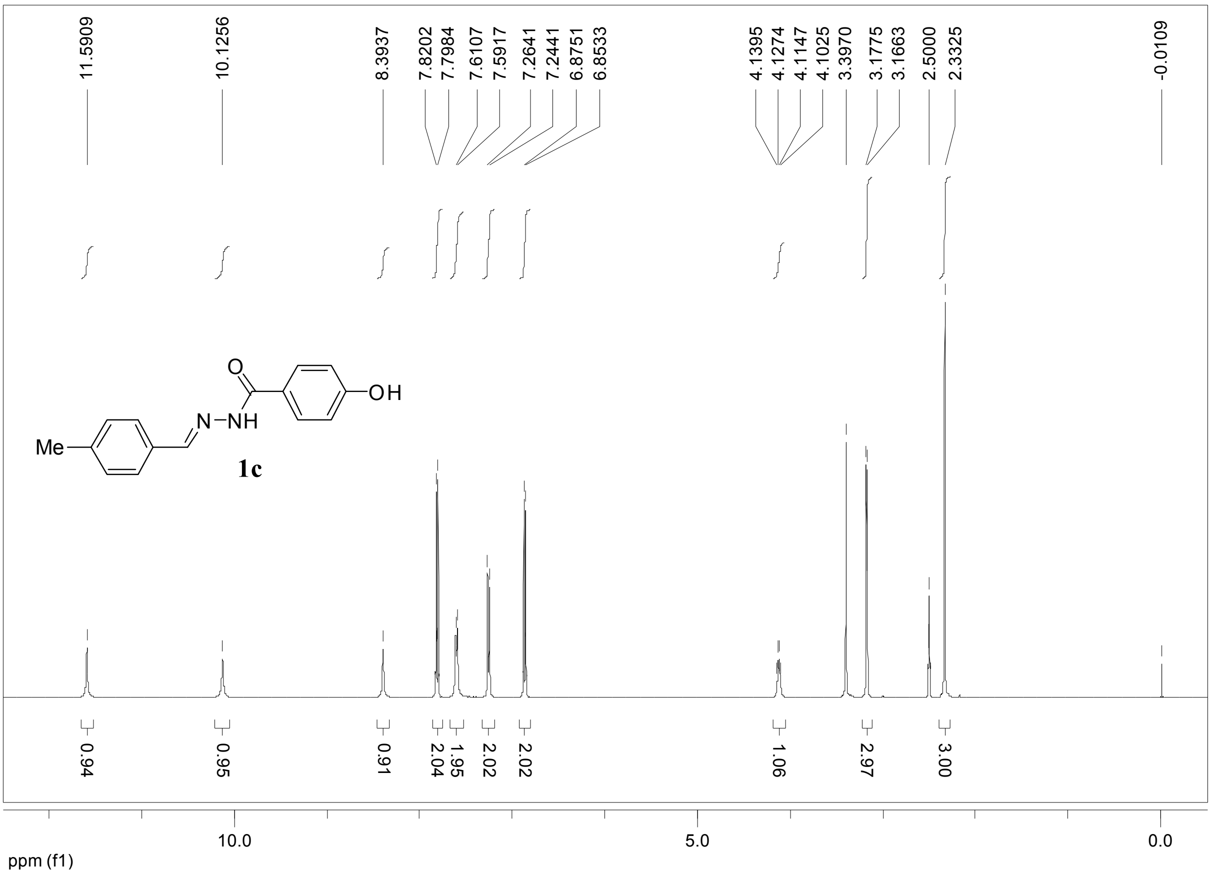

Figure S22. ^{1}H-NMR (400 MHz, DMSO-d_6) spectrum of compound **1c**.

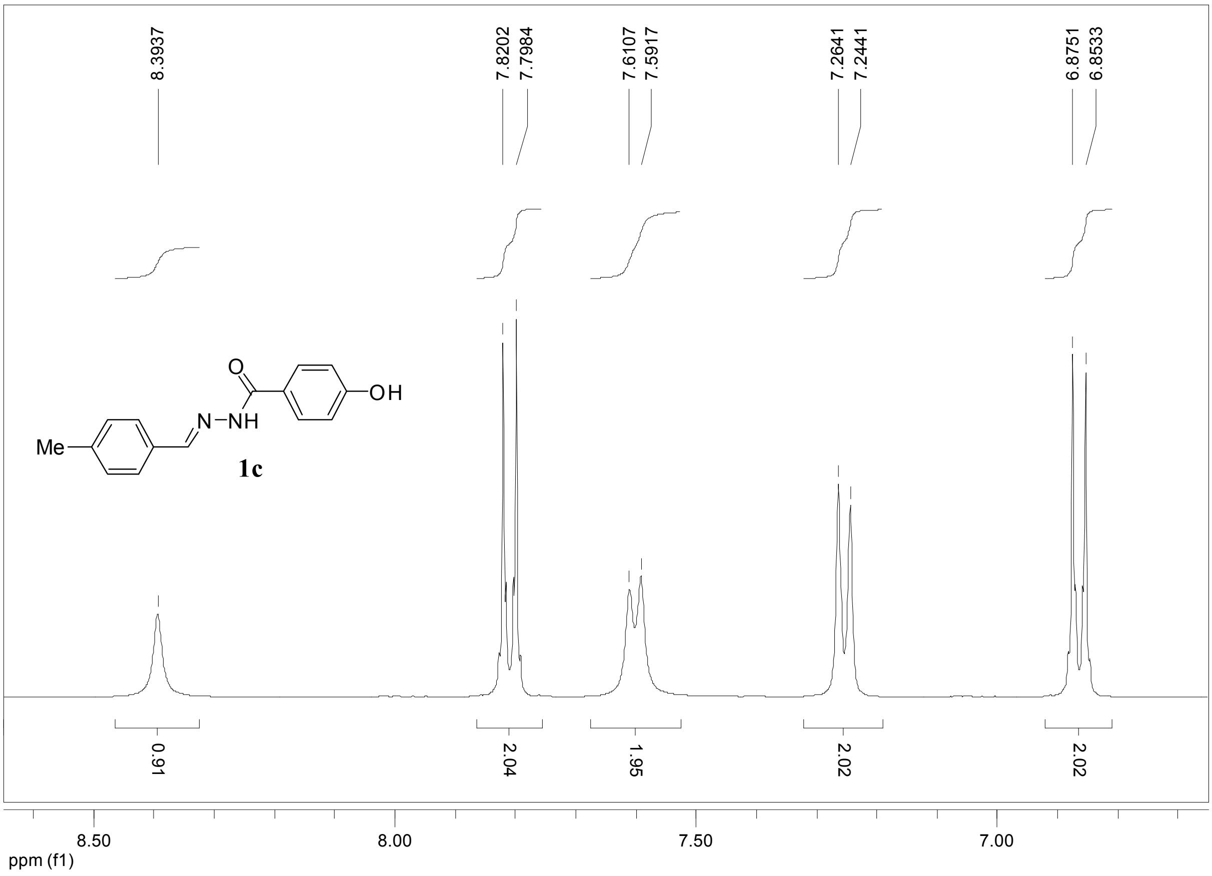

Figure S23. Expansion of ^{1}H-NMR (400 MHz, DMSO-d_6) spectrum of compound **1c**.

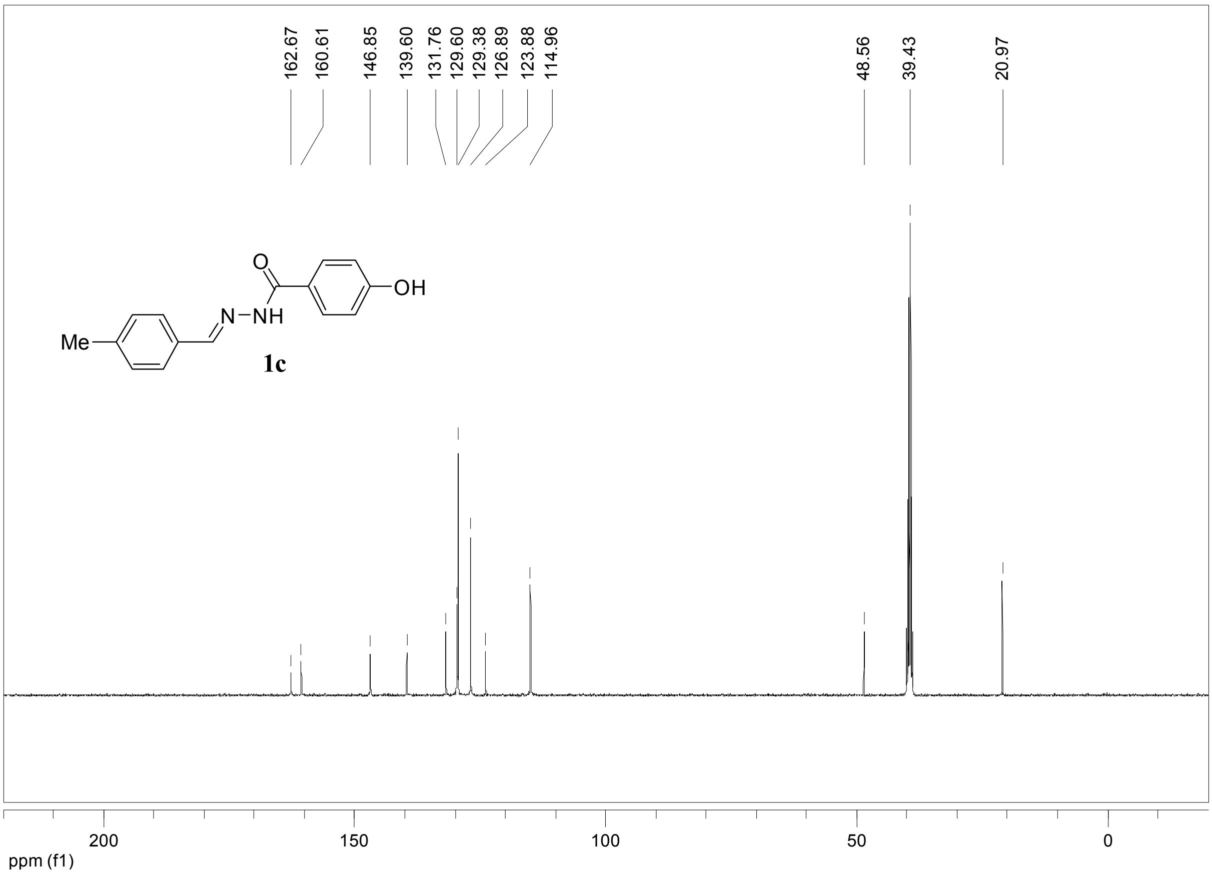

Figure S24. ^{13}C-NMR (100 MHz, DMSO-d_6) spectrum of compound **1c**.

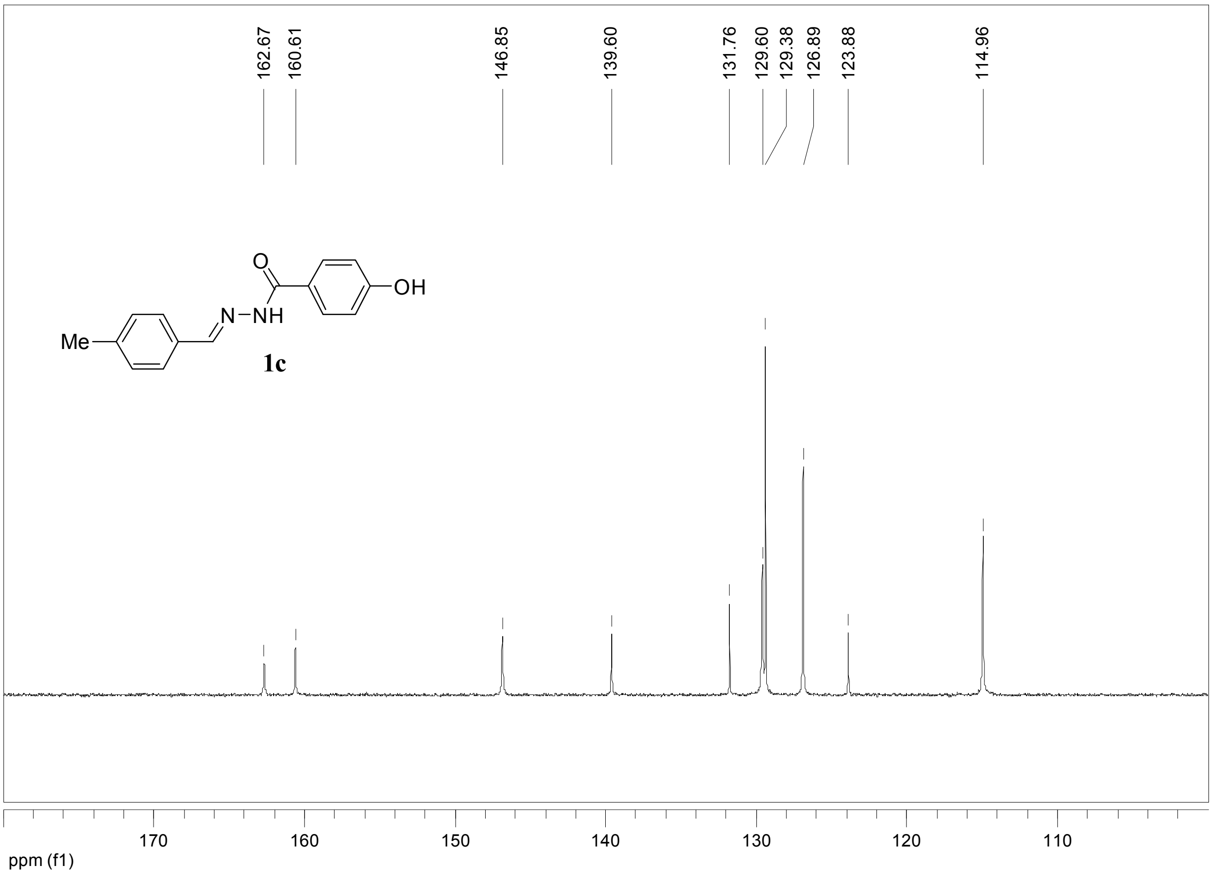

Figure S25. Expansion of ^{13}C-NMR (100 MHz, DMSO-d_6) spectrum of compound **1c**.

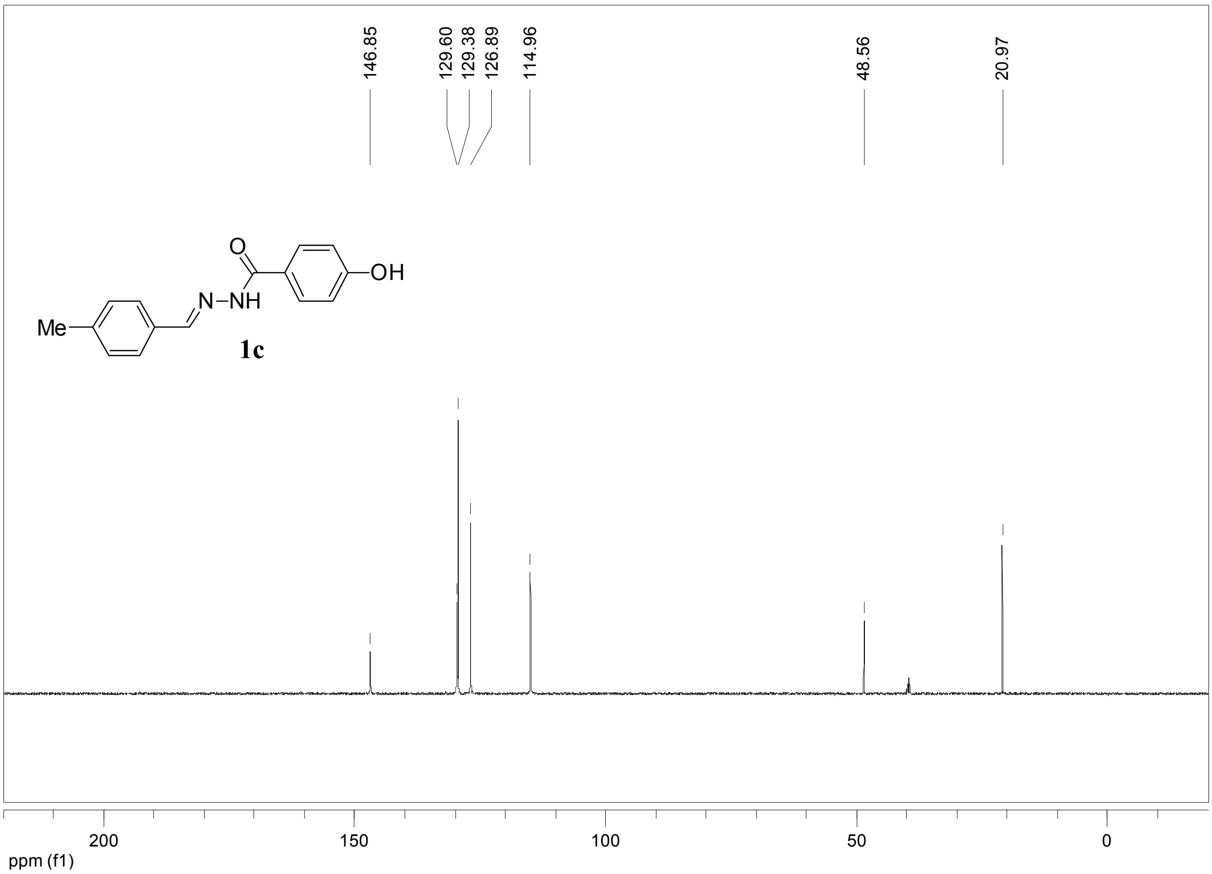

Figure S26. ^{13}C-NMR (100 MHz, DMSO-d_6) dept-135 experiment of compound **1c**.

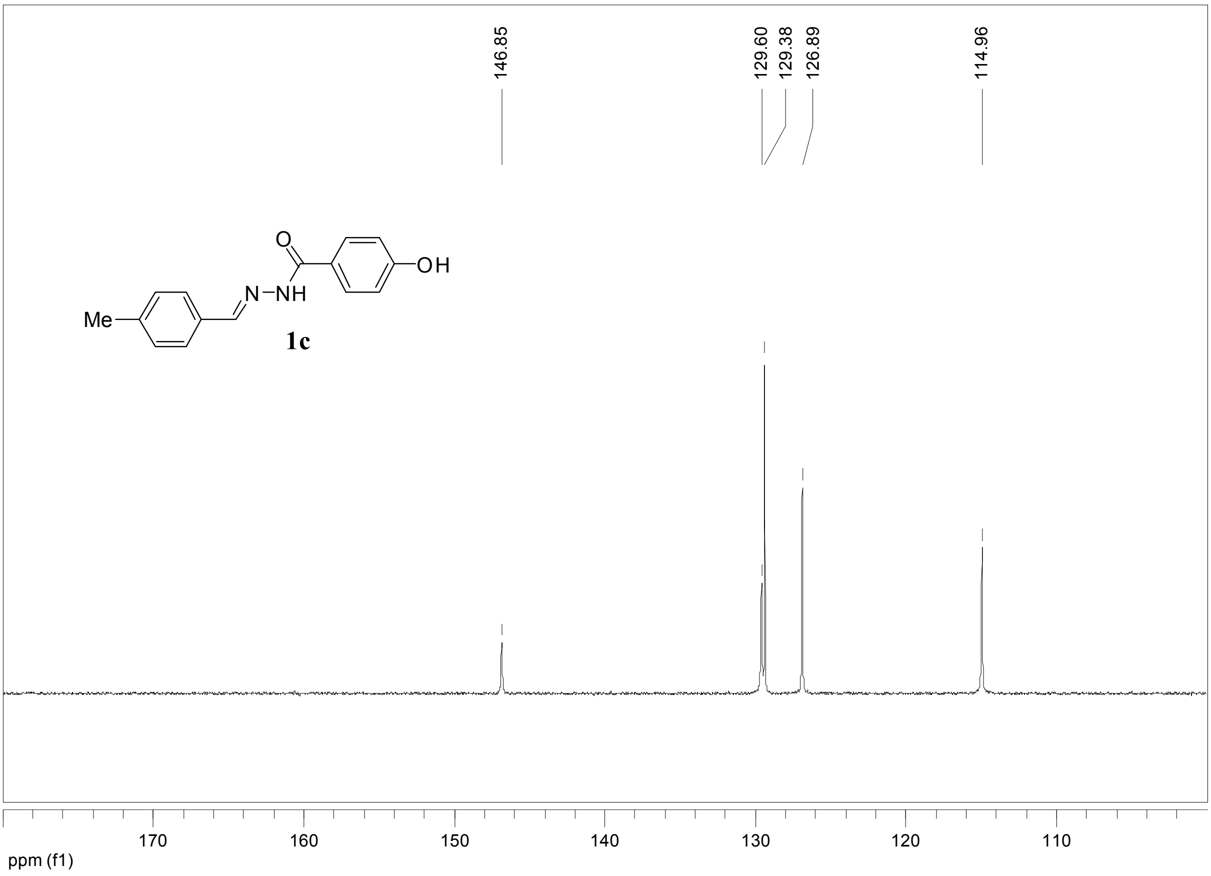

Figure S27. Expansion of ^{13}C-NMR (100 MHz, DMSO-d_6) dept-135 experiment of compound **1c**.

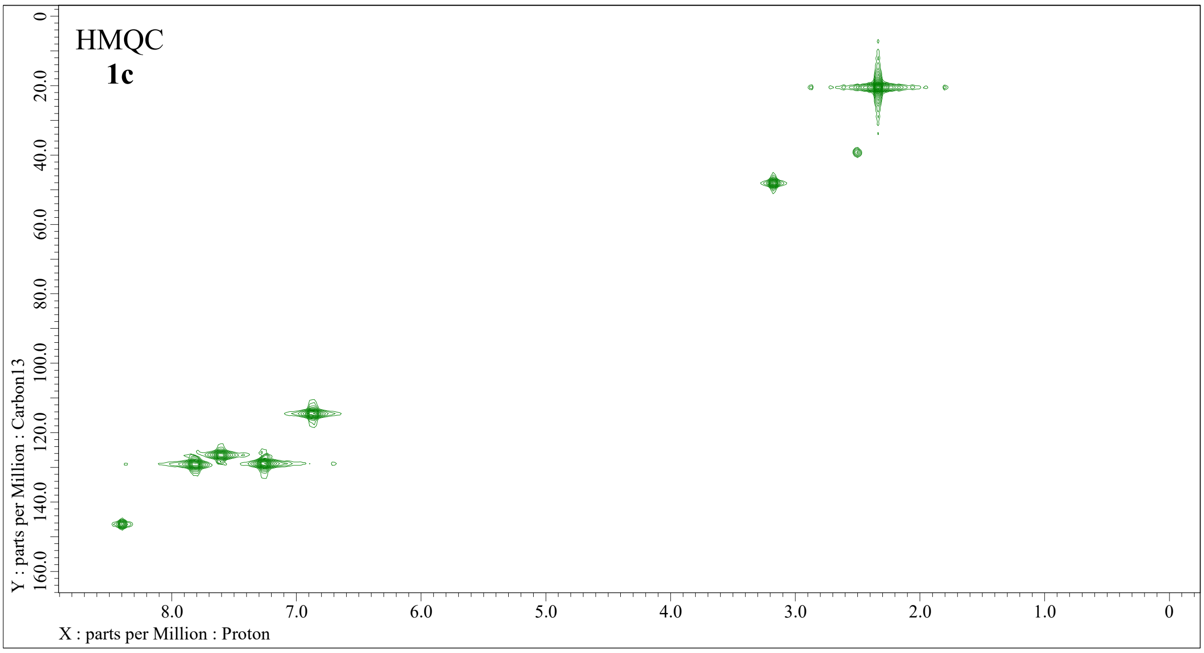

Figure S28. 2D-NMR (400 MHz, DMSO-d_6) HMQC experiment of 4-hydroxy-*N*'-[(*E*)-(4-methylphenyl)methylidene]benzohydrazide (**1c**).

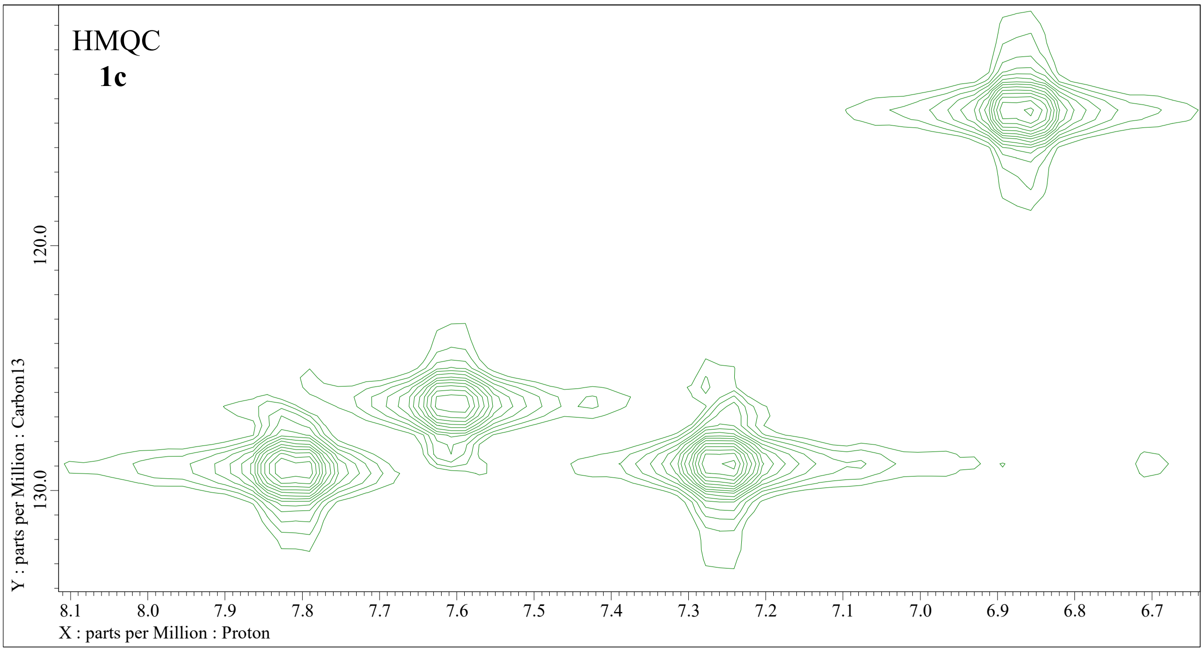

Figure S29. Expansion of 2D-NMR (400 MHz, DMSO-d_6) HMQC experiment of 4-hydroxy-*N*'-[(*E*)-(4-methylphenyl)methylidene]benzohydrazide (**1c**).

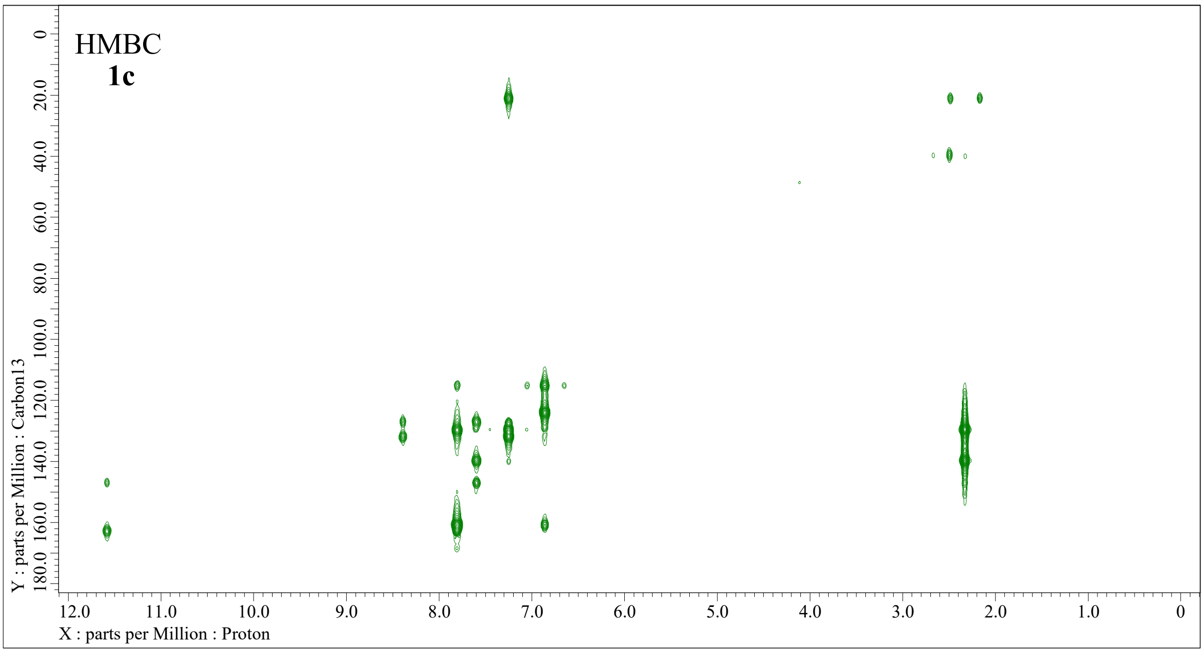

Figure S30. 2D-NMR (400 MHz, DMSO-d_6) HMBC experiment of 4-hydroxy-*N*'-[(*E*)-(4-methylphenyl)methylidene]benzohydrazide (**1c**).

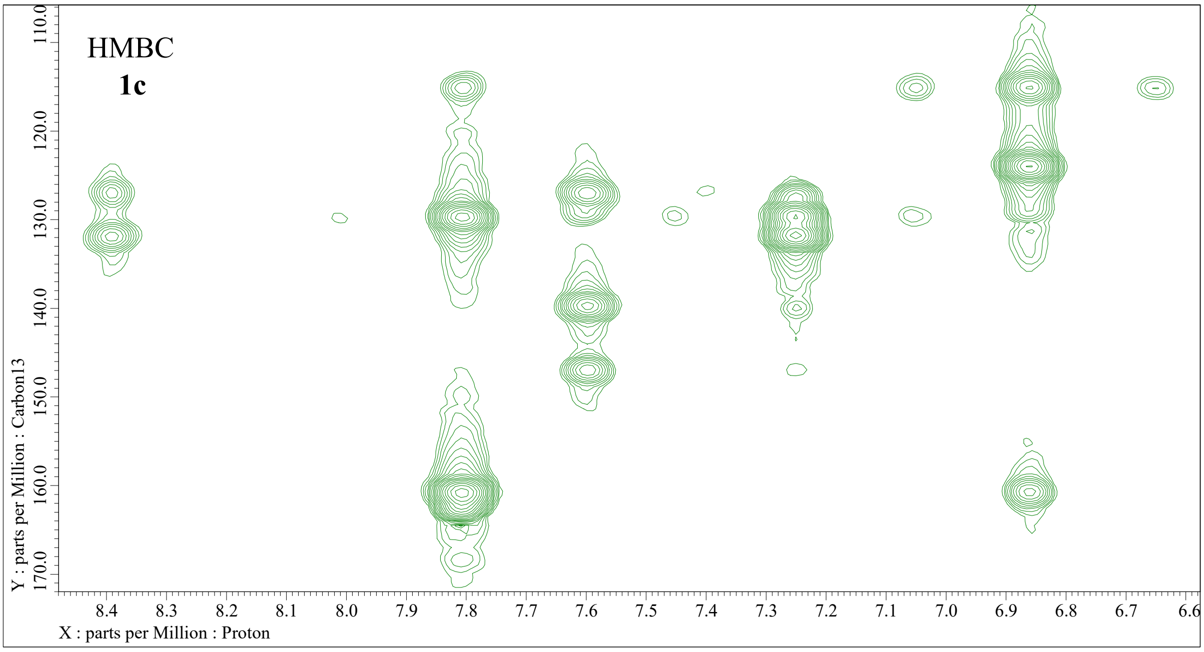

Figure S31. Expansion of 2D-NMR (400 MHz, DMSO-d_6) HMBC experiment of 4-hydroxy-*N*'-[(*E*)-(4-methylphenyl)methylidene]benzohydrazide (**1c**).

Molecules **2020**, *25*, 1255; doi:10.3390/molecules25051255 www.mdpi.com/journal/molecules

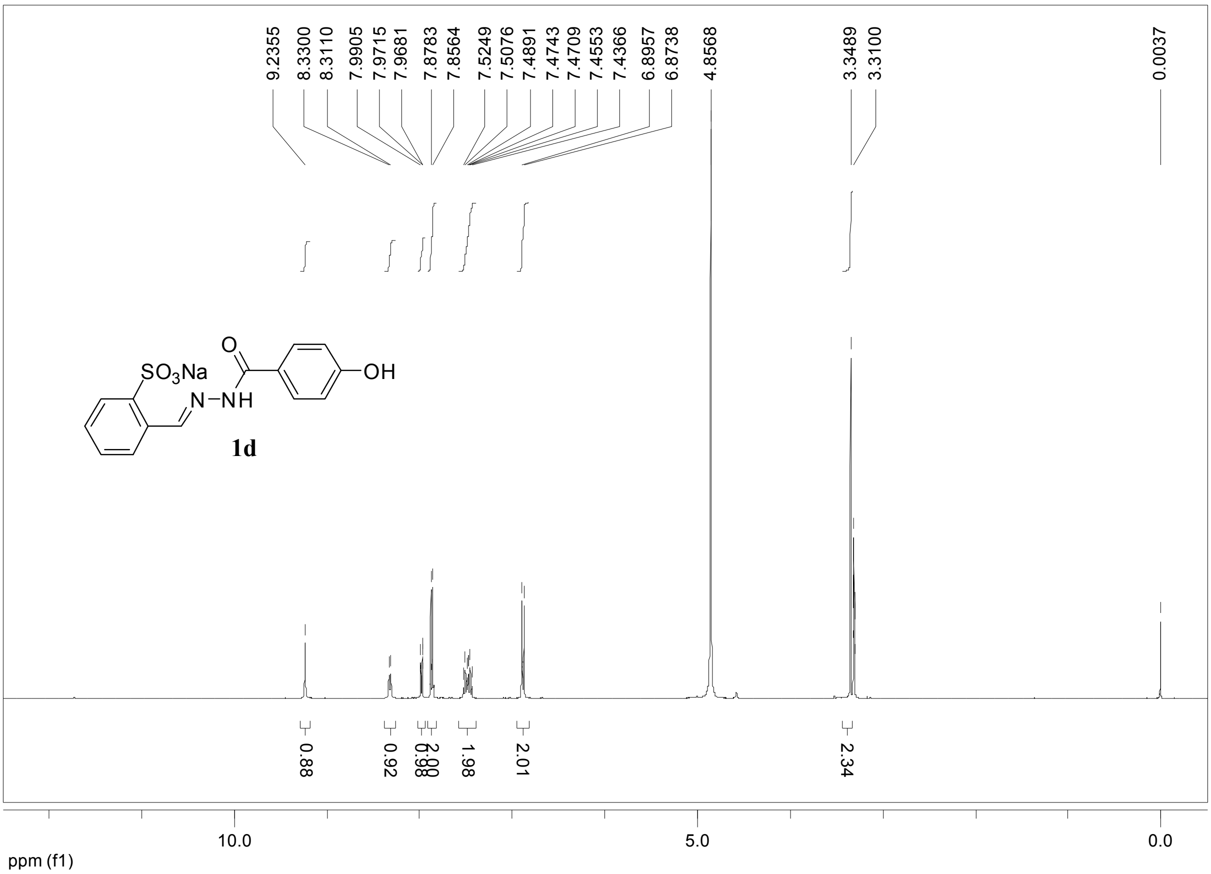

Figure S32. ^{1}H-NMR (400 MHz, CH_3OH-d_4) spectrum of compound **1d**.

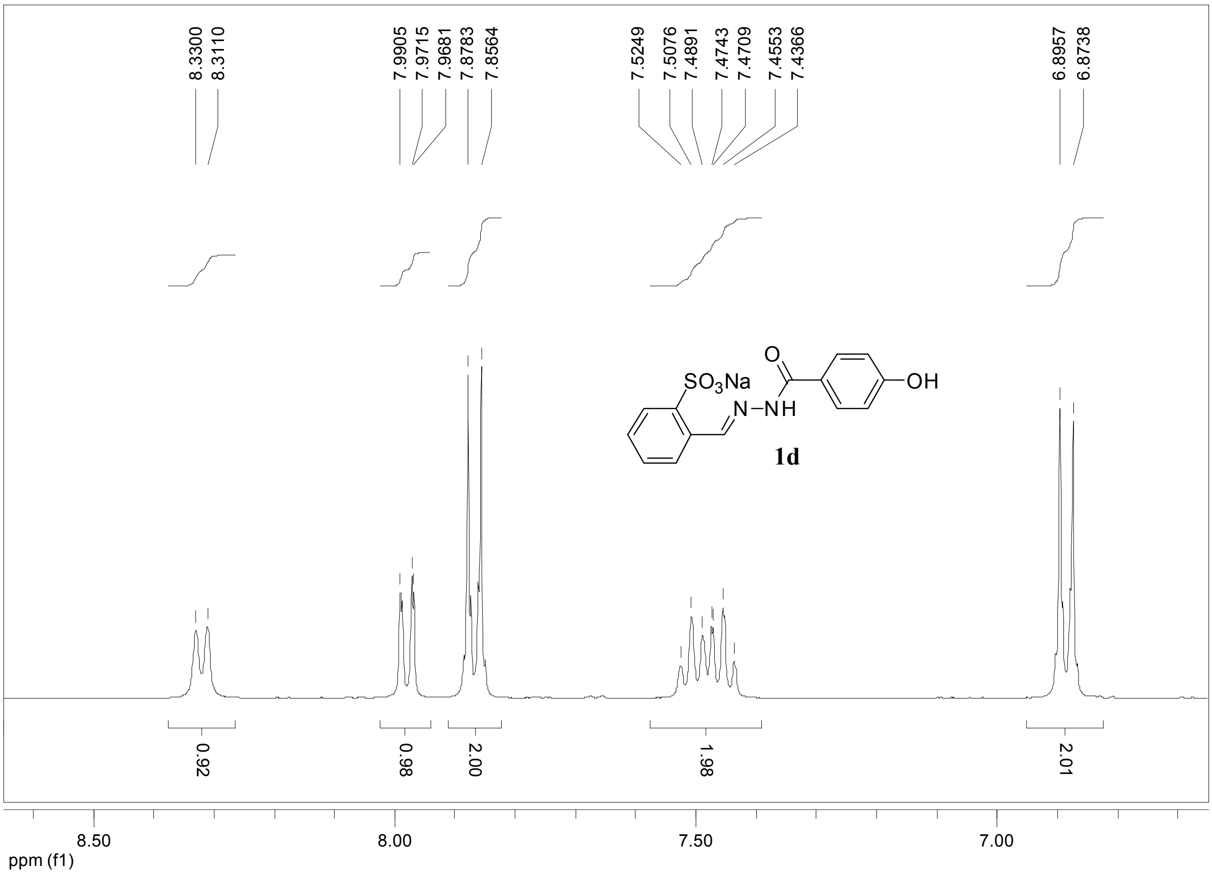

Figure S33. Expansion of ^{1}H-NMR (400 MHz, CH_3OH-d_4) spectrum of compound **1d**.

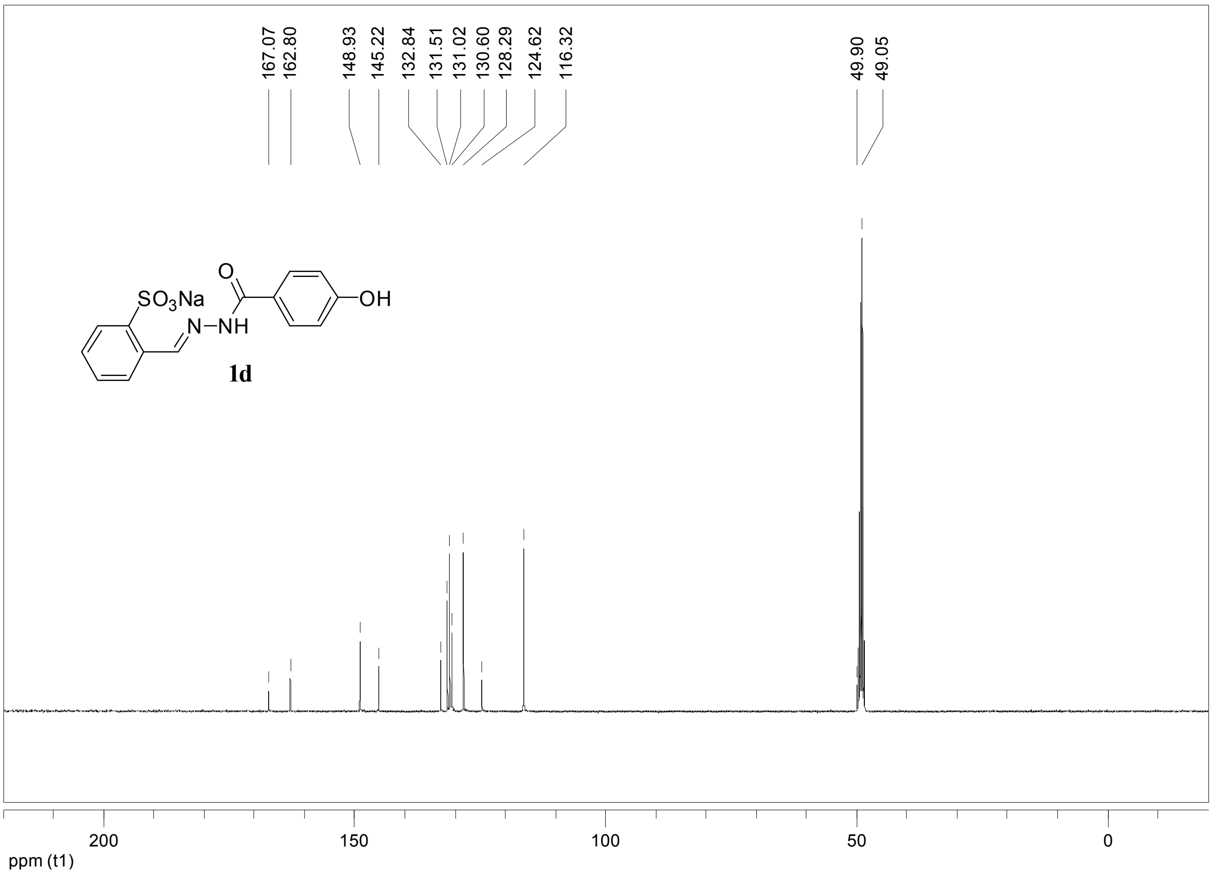

Figure S34. ^{13}C-NMR (100 MHz, CH_3OH-d_4) spectrum of compound **1d**.

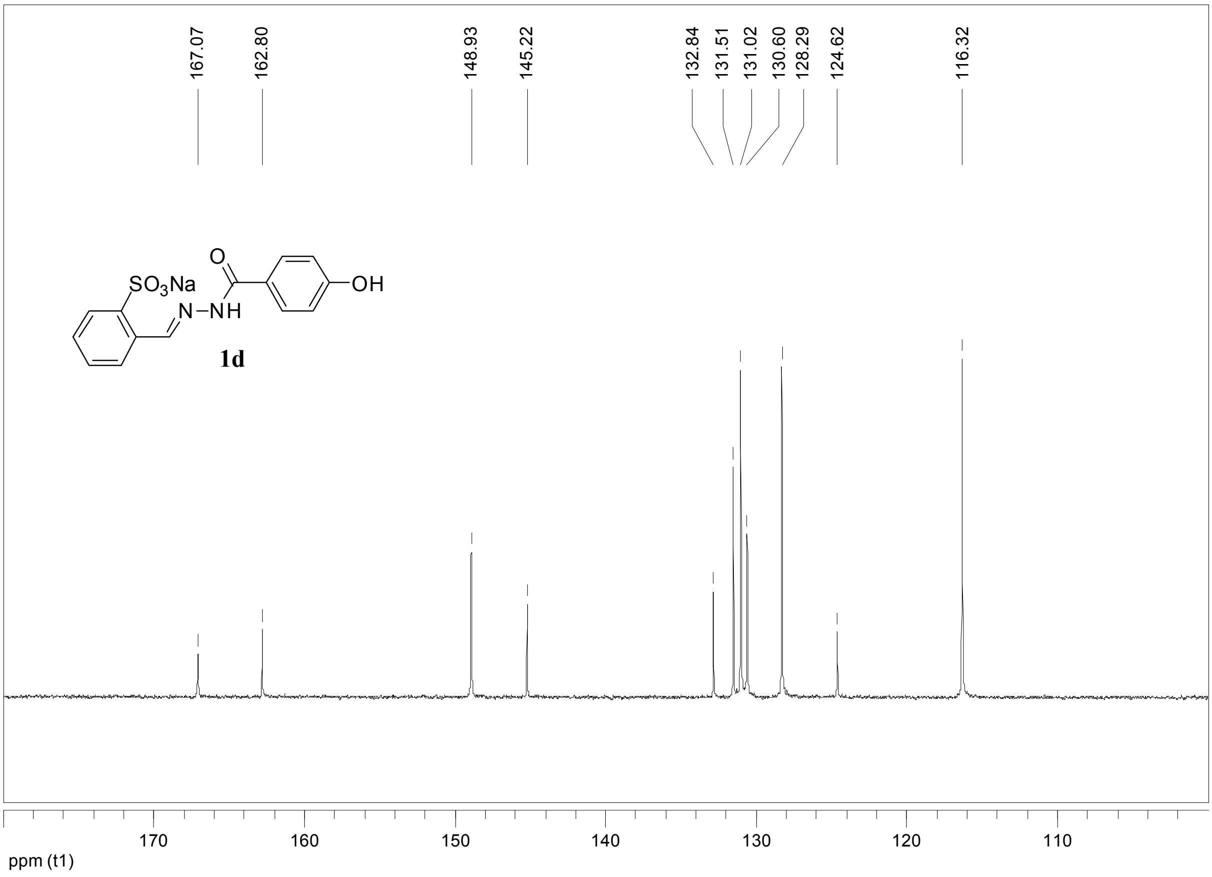

Figure S35. Expansion of ^{13}C-NMR (100 MHz, CH_3OH-d_4) spectrum of compound **1d**.

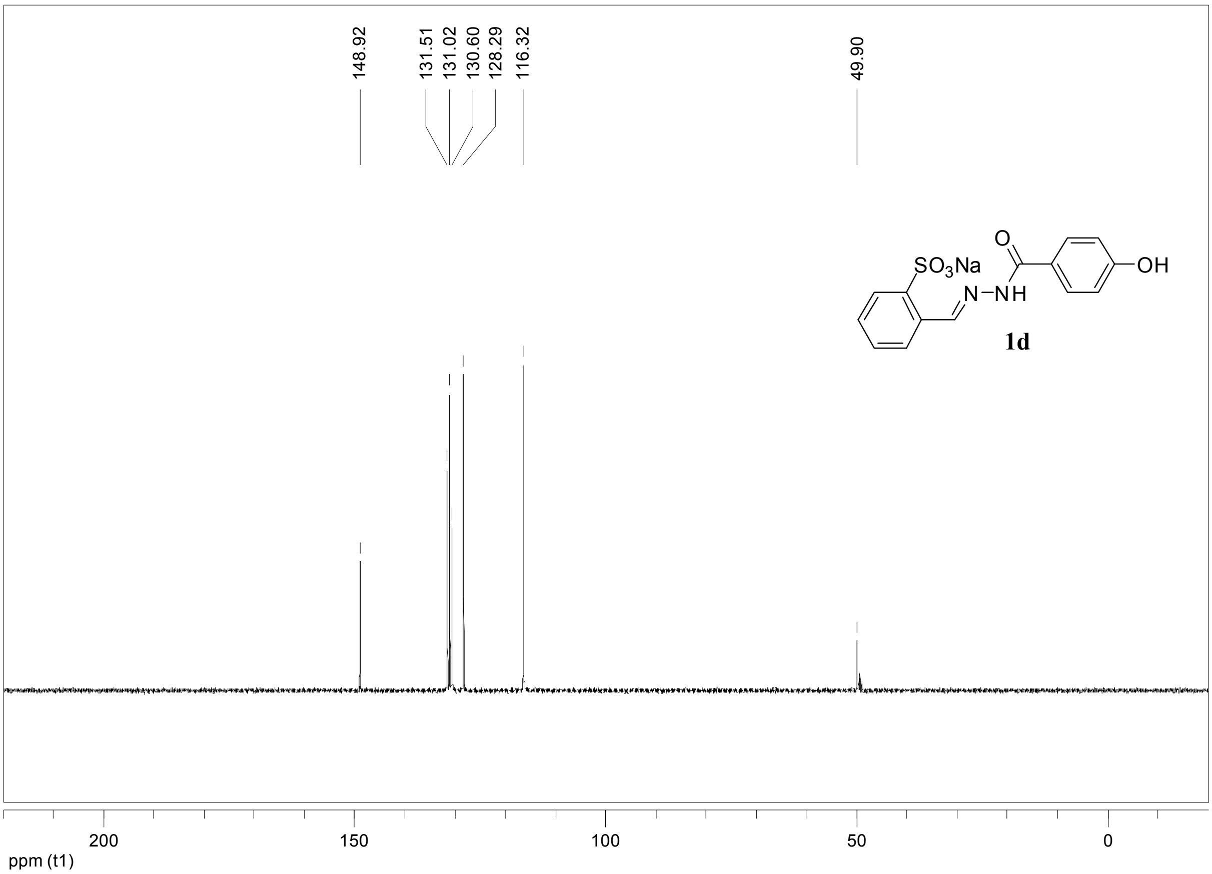

Figure S36. ^{13}C-NMR (100 MHz, CH3OH-*d*4) dept-135 experiment of compound **1d**.

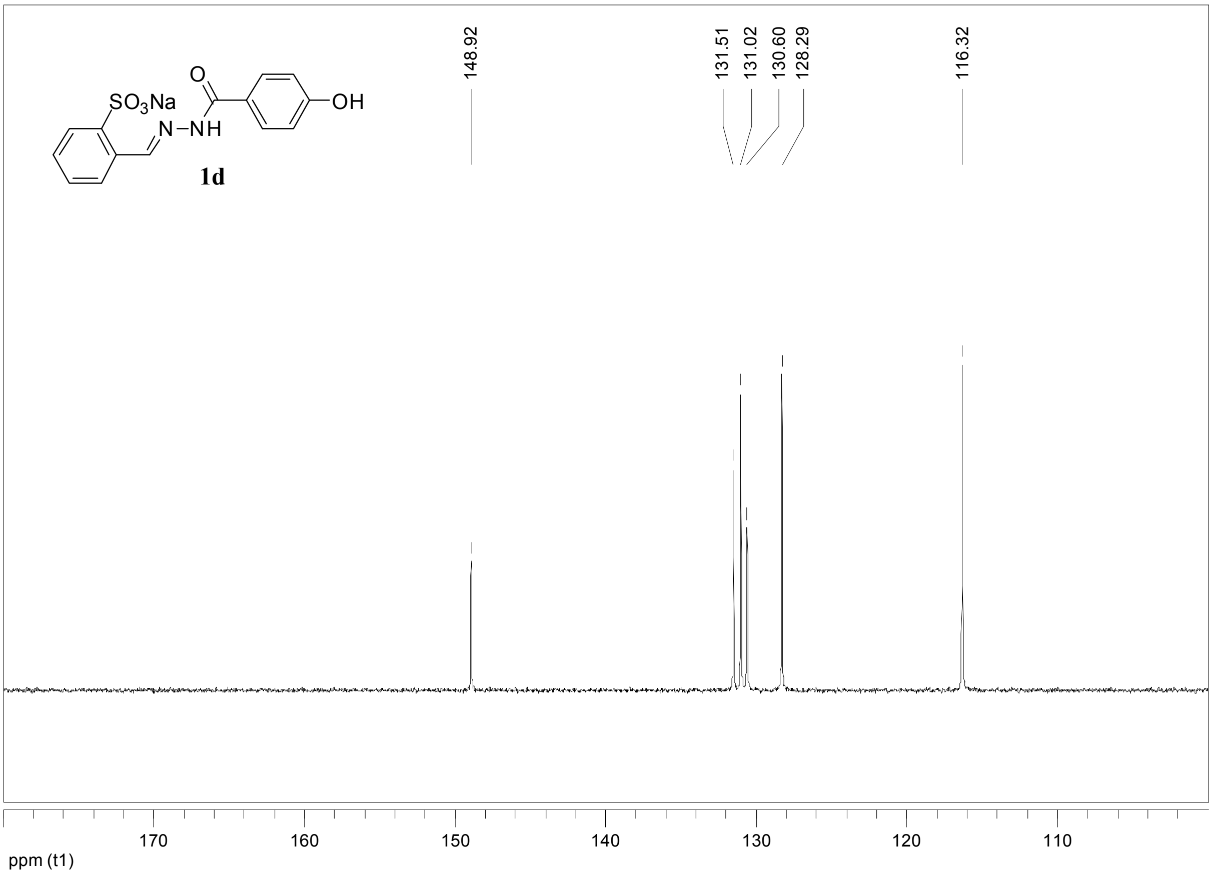

Figure S37. Expansion of ^{13}C-NMR (100 MHz, CH3OH-*d*4) dept-135 experiment of compound **1d.**

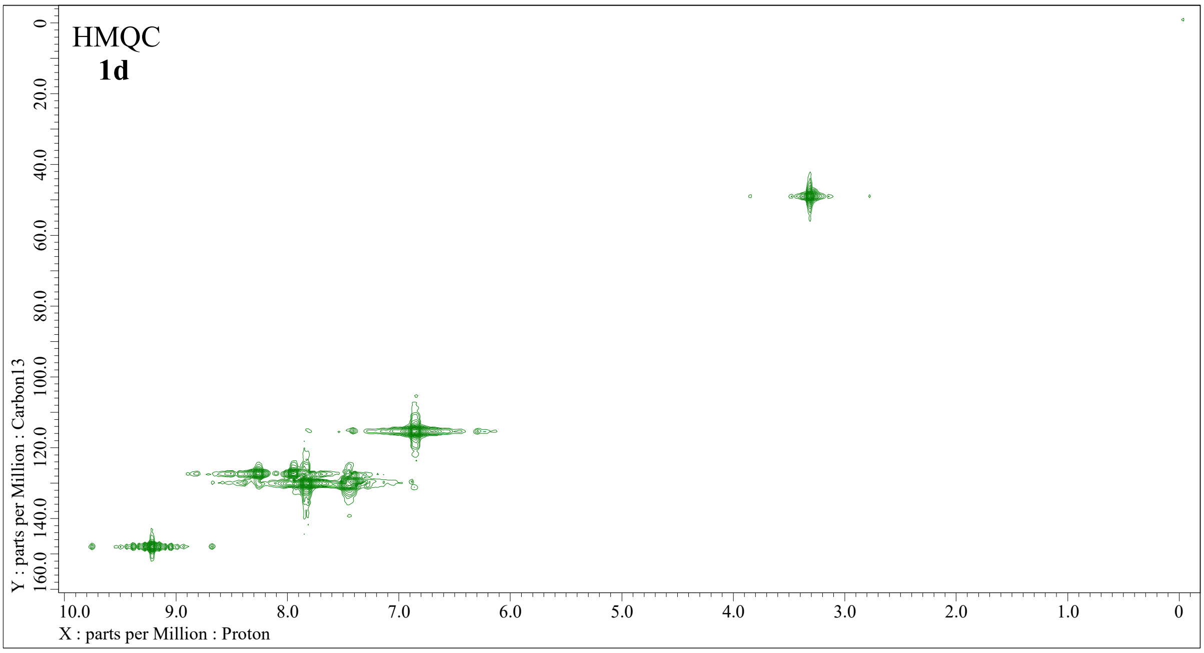

Figure S38. 2D-NMR (400 MHz, CH_3OH-*d*$_4$) HMQC experiment of sodium 2-{(*E*)-[2-(4-hydroxybenzoyl)hydrazinylidene]methyl}benzenesulfonate (**1d**).

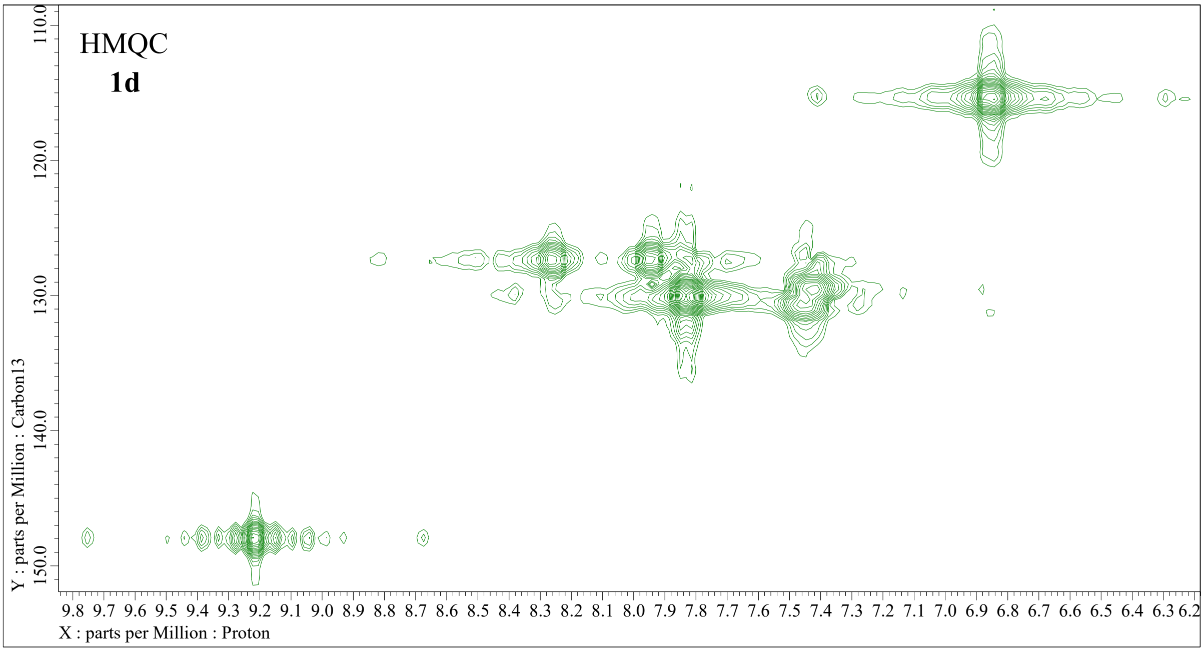

Figure S39. Expansion of 2D-NMR (400 MHz, CH_3OH-*d*$_4$) HMQC experiment of sodium 2-{(*E*)-[2-(4-hydroxybenzoyl)hydrazinylidene]methyl}benzenesulfonate (**1d**).

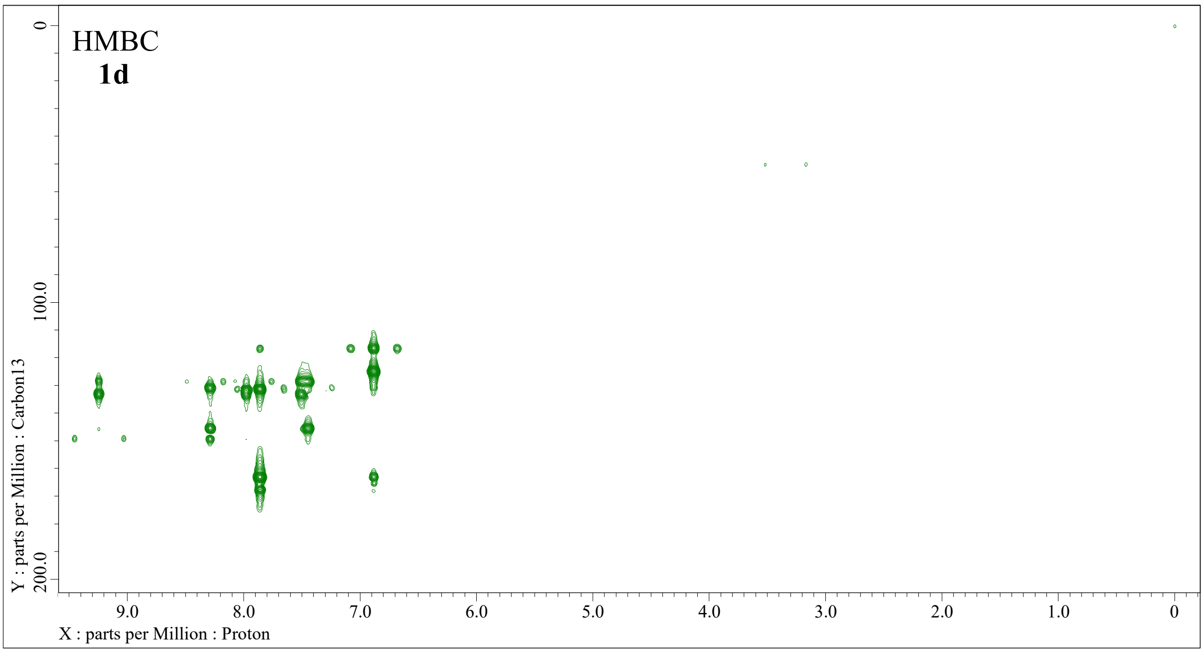

Figure S40. 2D-NMR (400 MHz, CH_3OH-d_4) HMBC experiment of sodium 2-{(*E*)-[2-(4-hydroxybenzoyl)hydrazinylidene]methyl}benzenesulfonate (**1d**).

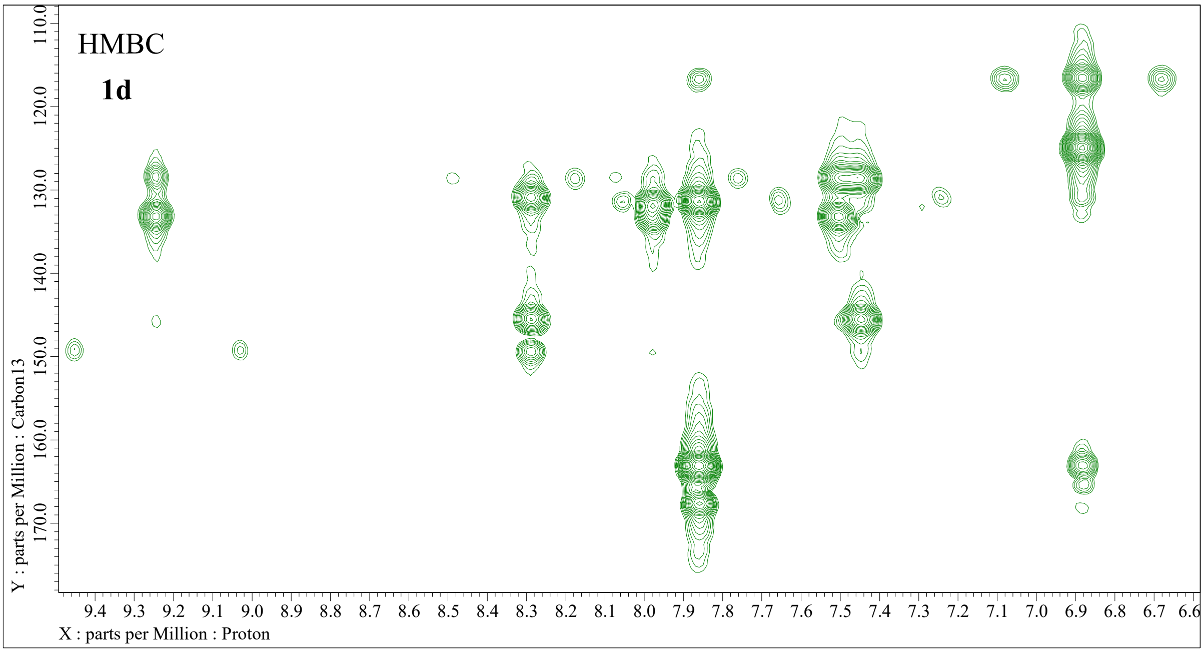

Figure S41. Expansion of 2D-NMR (400 MHz, CH_3OH-d_4) HMBC experiment of sodium 2-{(*E*)-[2-(4-hydroxybenzoyl)hydrazinylidene]methyl}benzenesulfonate (**1d**).

Complete layout correction per preceding examples noting bold numbering (and the fact the numbers are off by one) and label placement

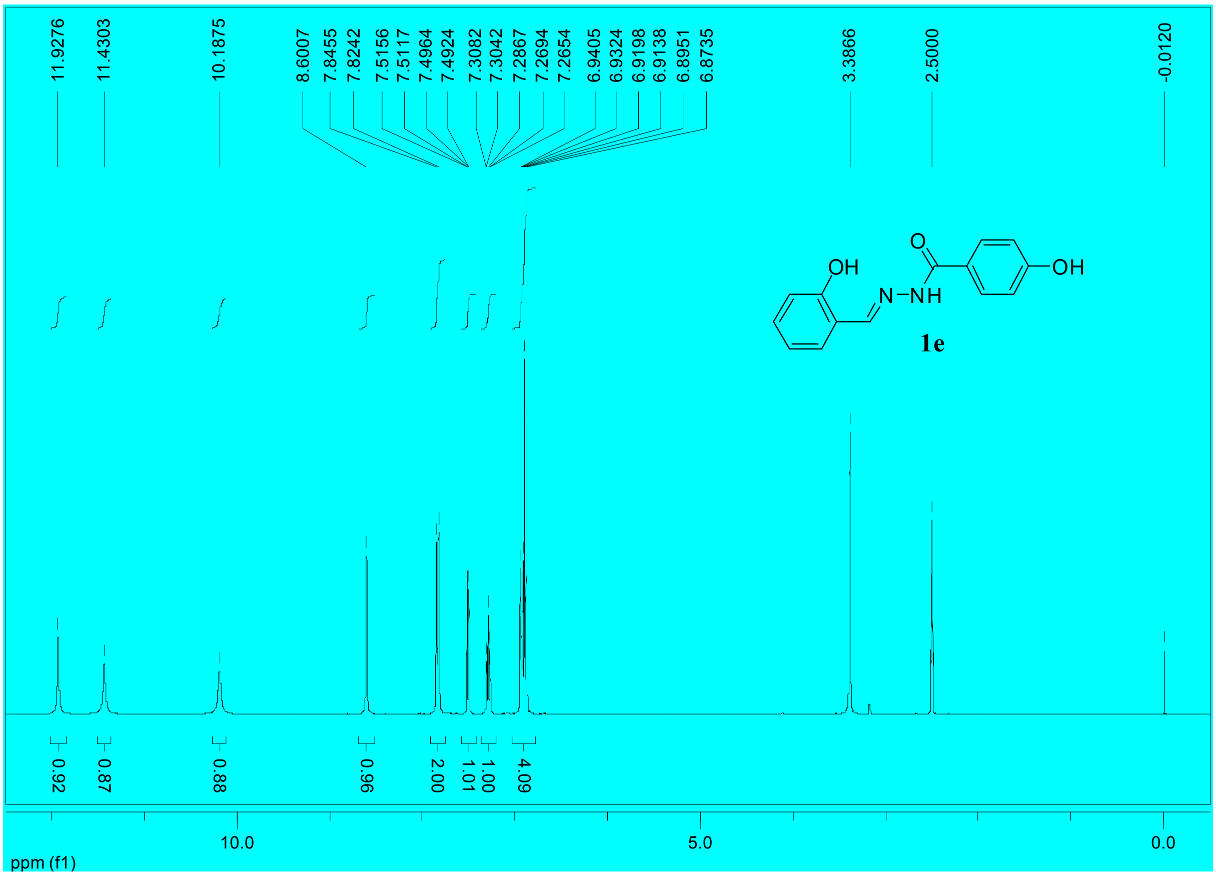

Figure S43. ^{1}H-NMR (400 MHz, DMSO-d_6) spectrum of **1e**.

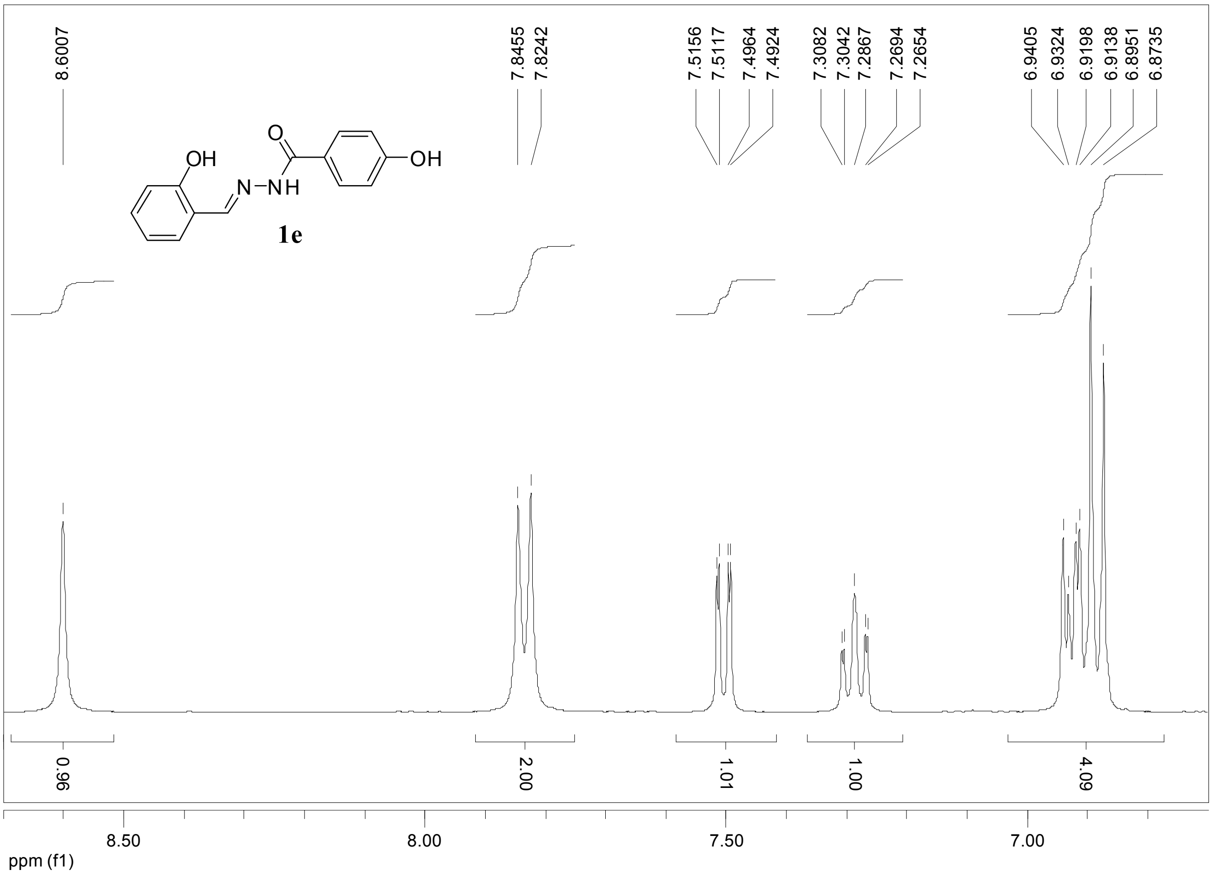

Figure S44. Expansion of ^{1}H-NMR (400 MHz, DMSO-d_6) spectrum of compound **1e**.

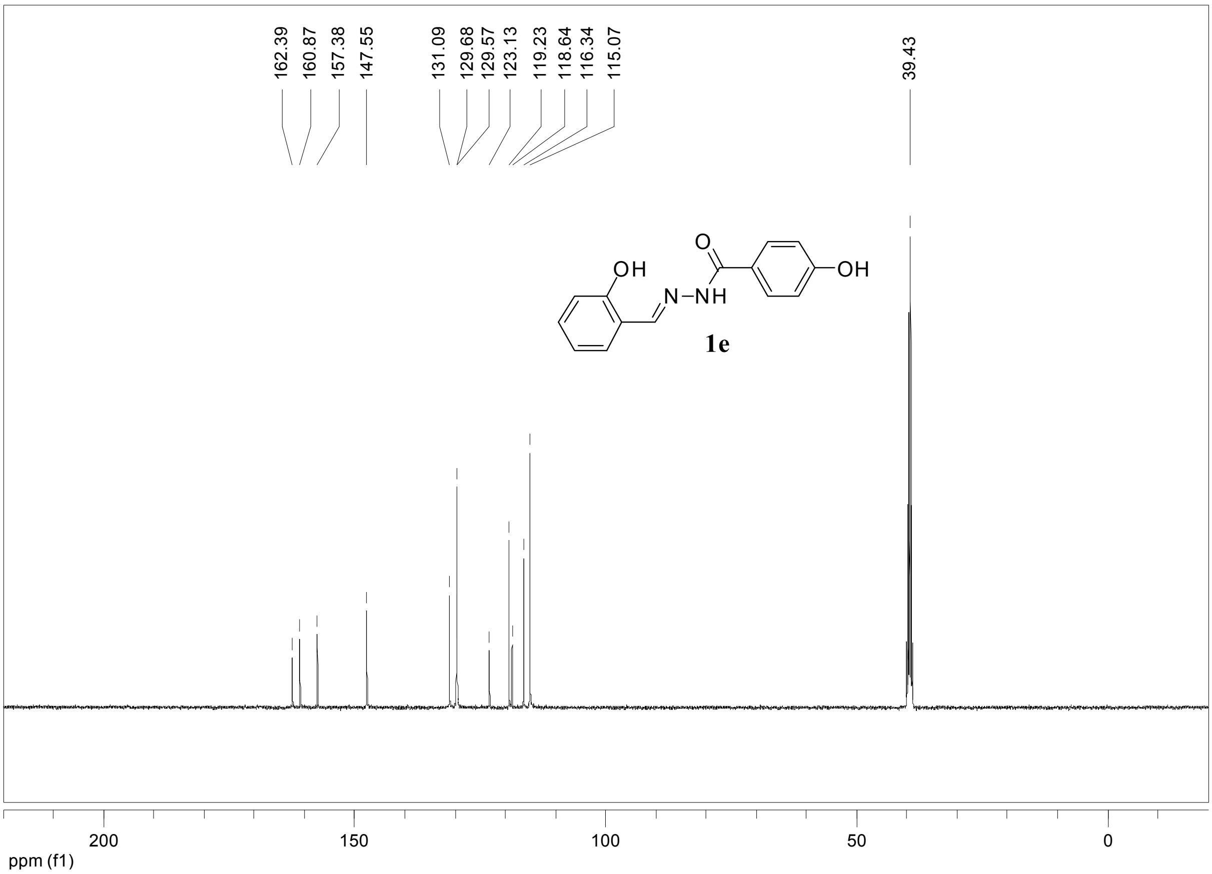

Figure S45. ^{13}C-NMR (100 MHz, DMSO-d_6) spectrum of compound **1e**.

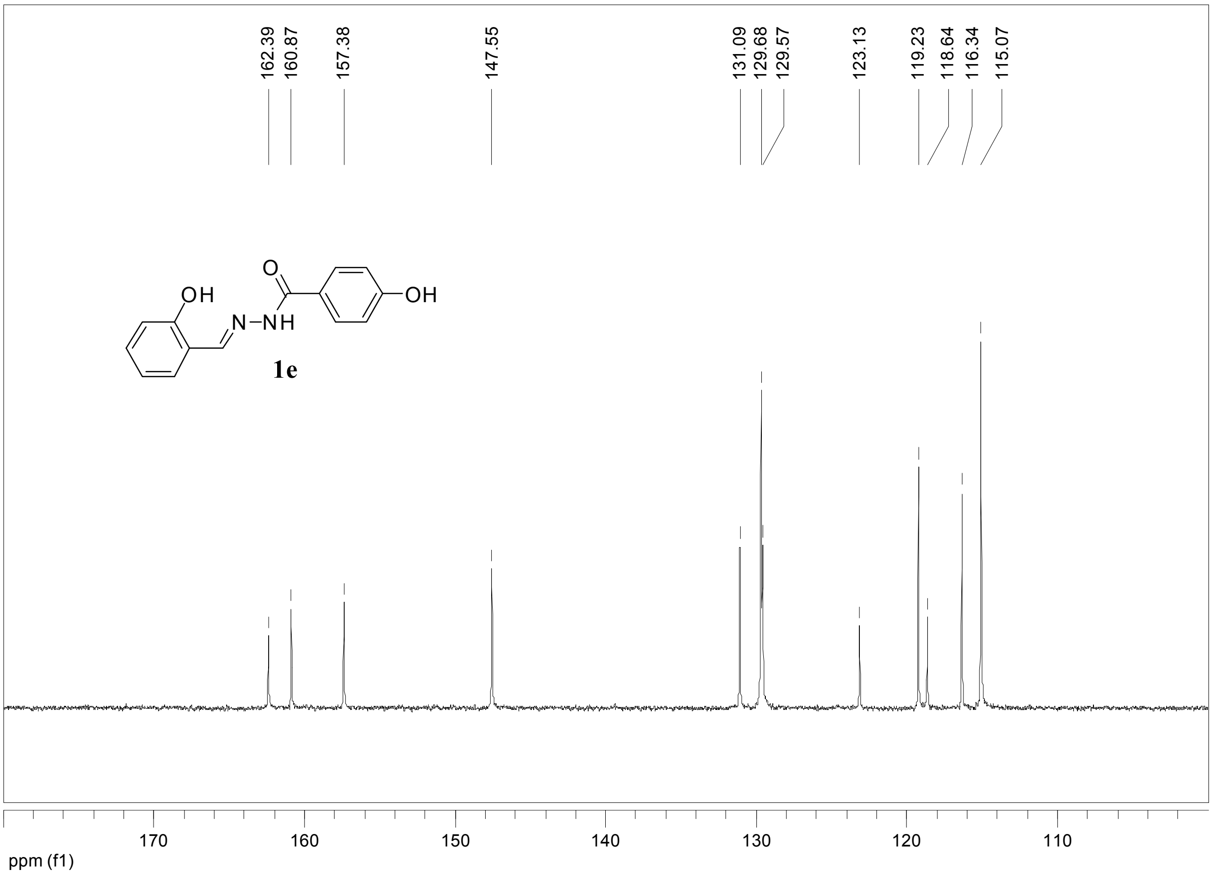

Figure S46. Expansion of ^{13}C-NMR (100 MHz, DMSO-d_6) spectrum of compound **1e**.

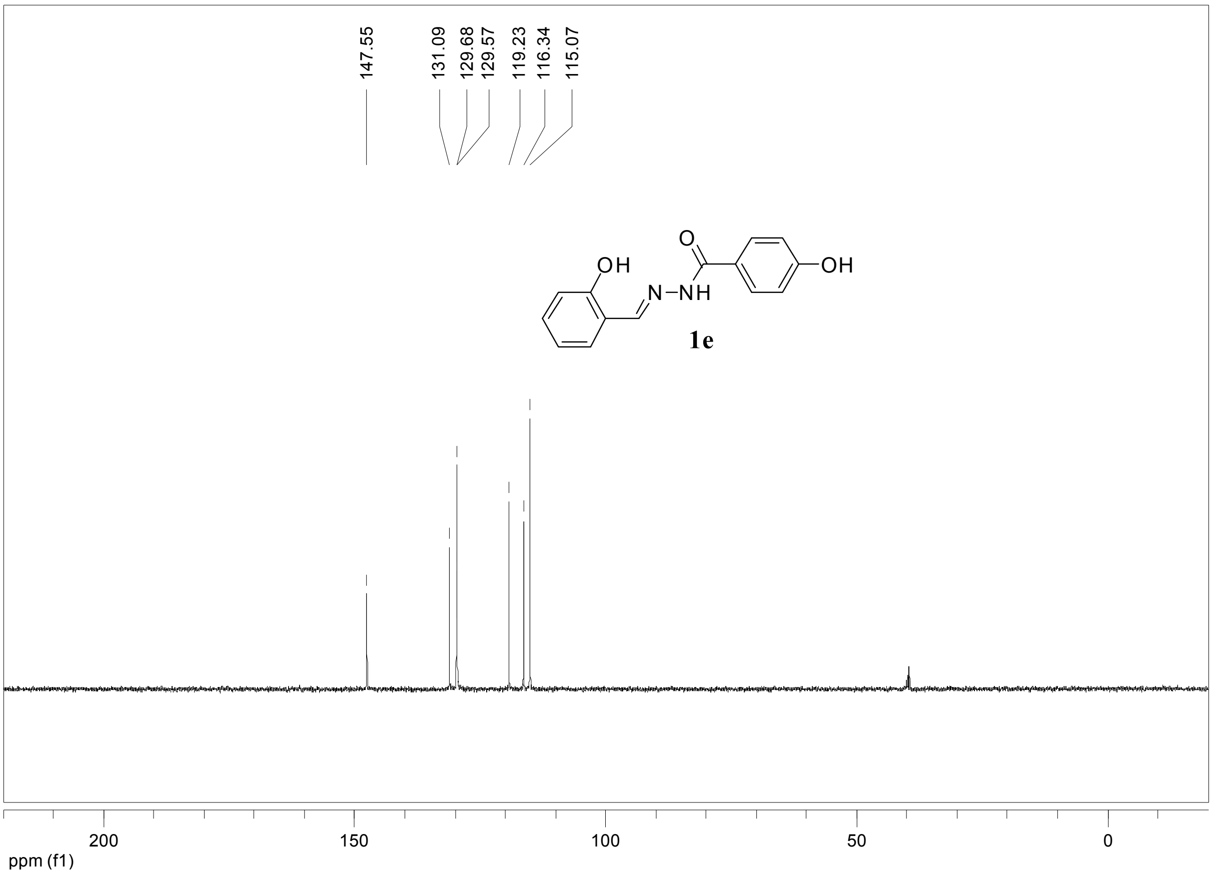

Figure S47. ^{13}C-NMR (100 MHz, DMSO-d_6) experiment dept-135 of compound **1e**.

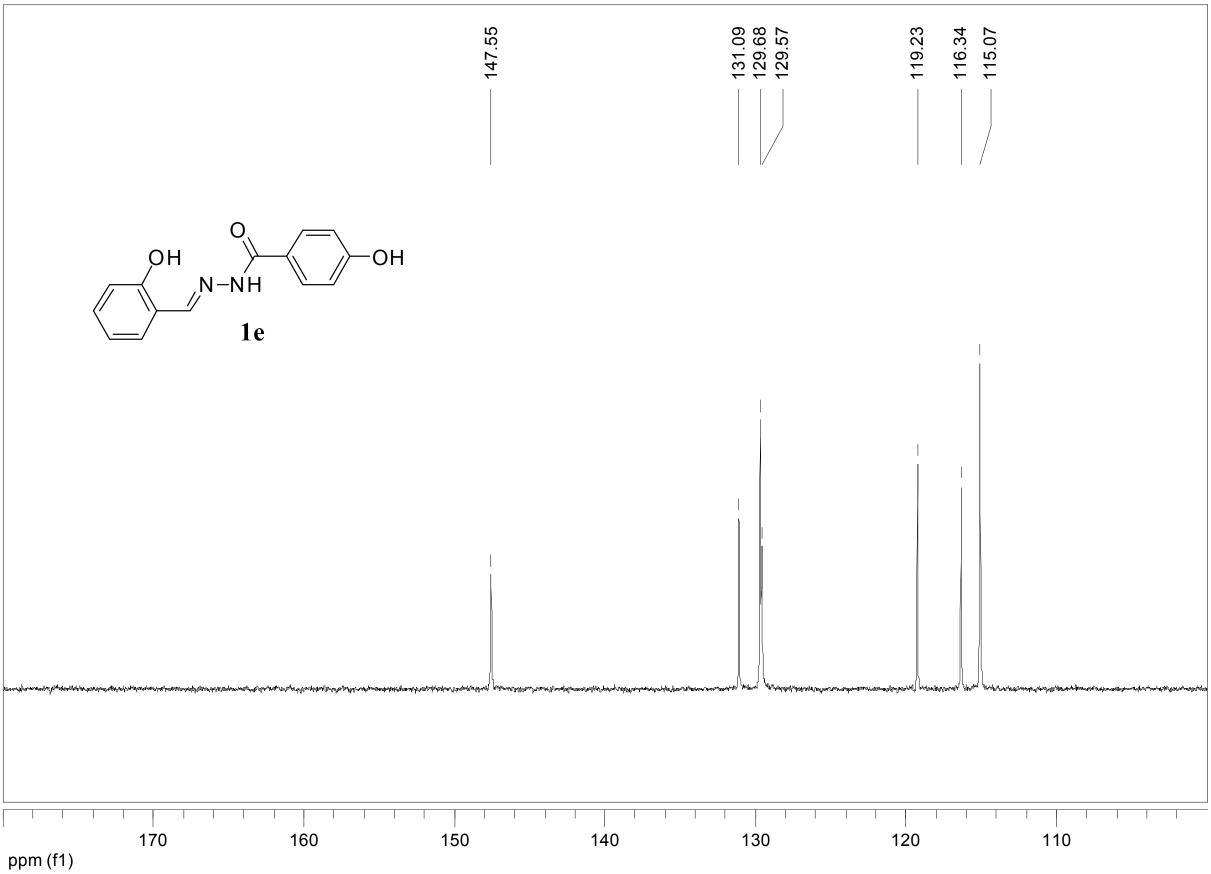

Figure S48. Expansion of ^{13}C-NMR (100 MHz, DMSO-d_6) experiment dept-135 of compound **1e**.

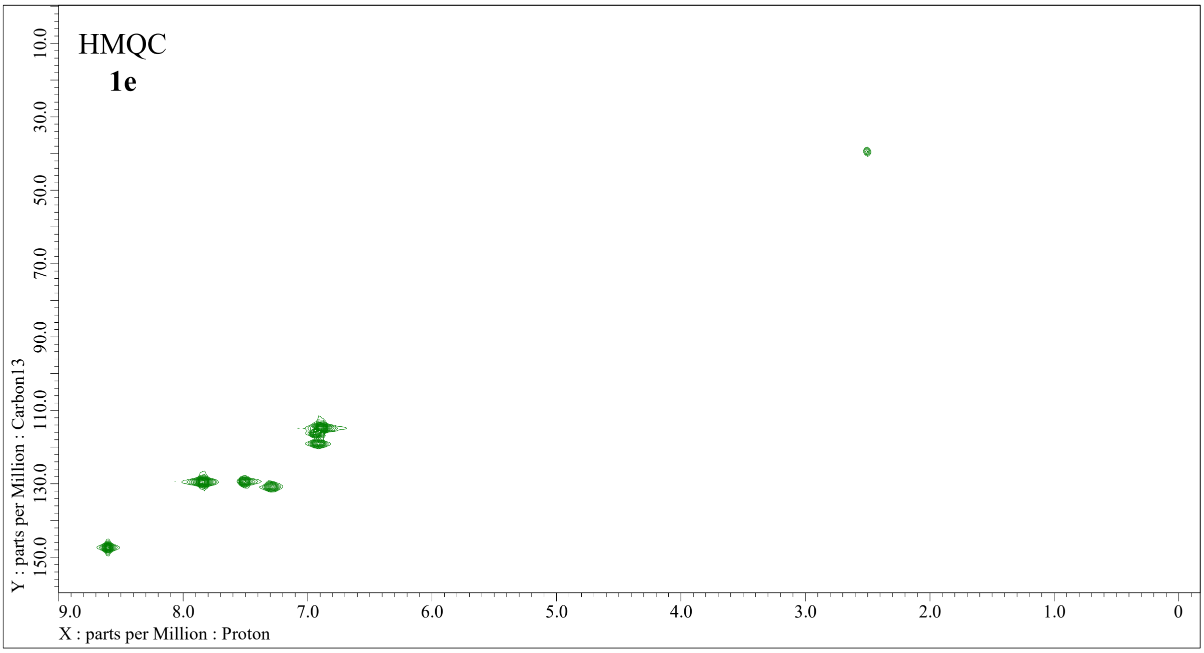

Figure S49. 2D-NMR (400 MHz, DMSO-d_6) HMQC experiment of 4-hydroxy-*N*′-[(*E*)-[2-hydroxyphenyl)methylidene]benzohydrazide (**1e**).

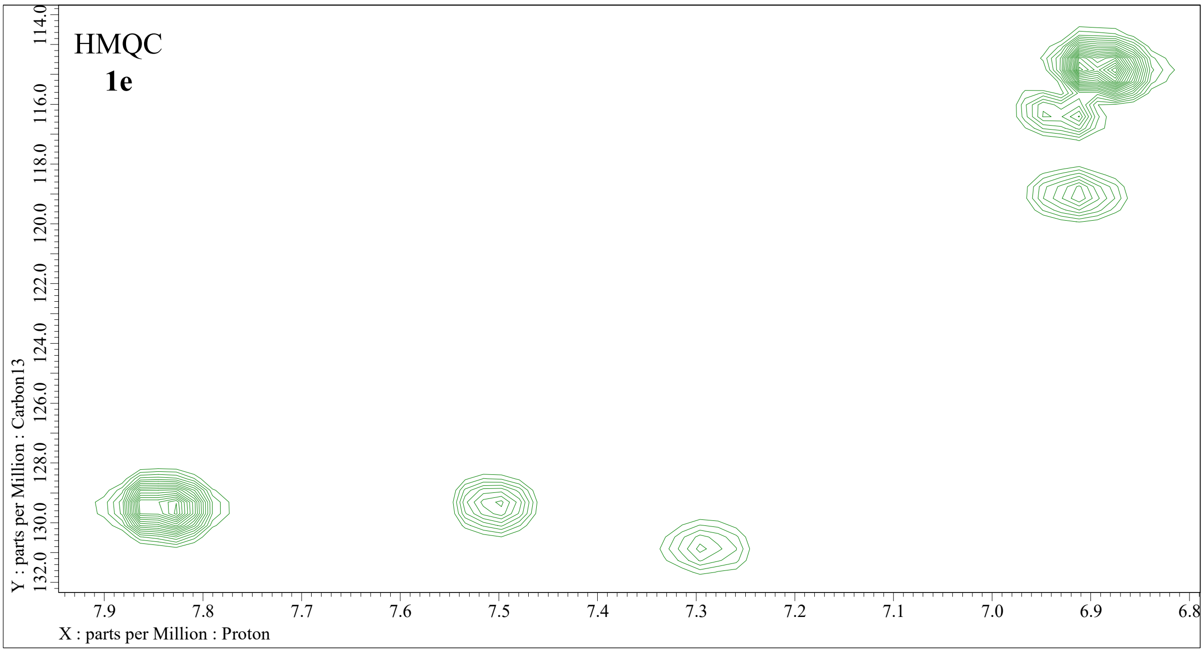

Figure S50. Expansion of 2D-NMR (400 MHz, DMSO-d_6) HMQC experiment of 4-hydroxy-*N*′-[(*E*)-[2-hydroxyphenyl)methylidene]benzohydrazide (**1e**).

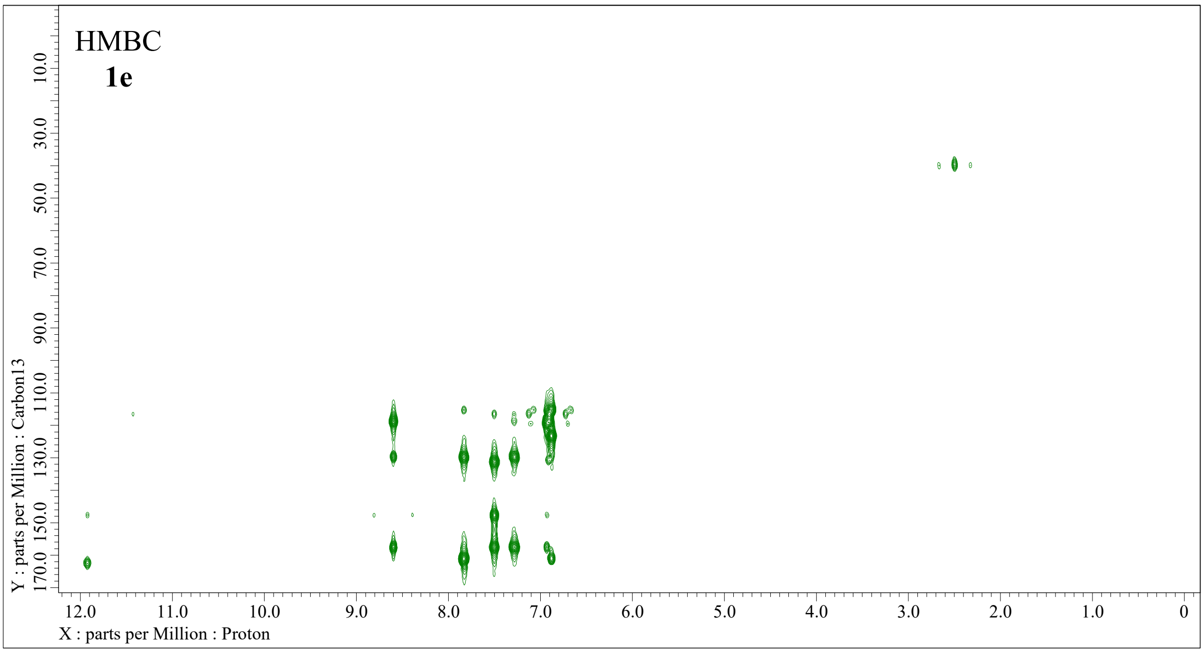

Figure S51. 2D-NMR (400 MHz, DMSO-d_6) HMBC experiment of 4-hydroxy-*N'*-[(*E*)-[2-hydroxyphenyl)methylidene]benzohydrazide (**1e**).

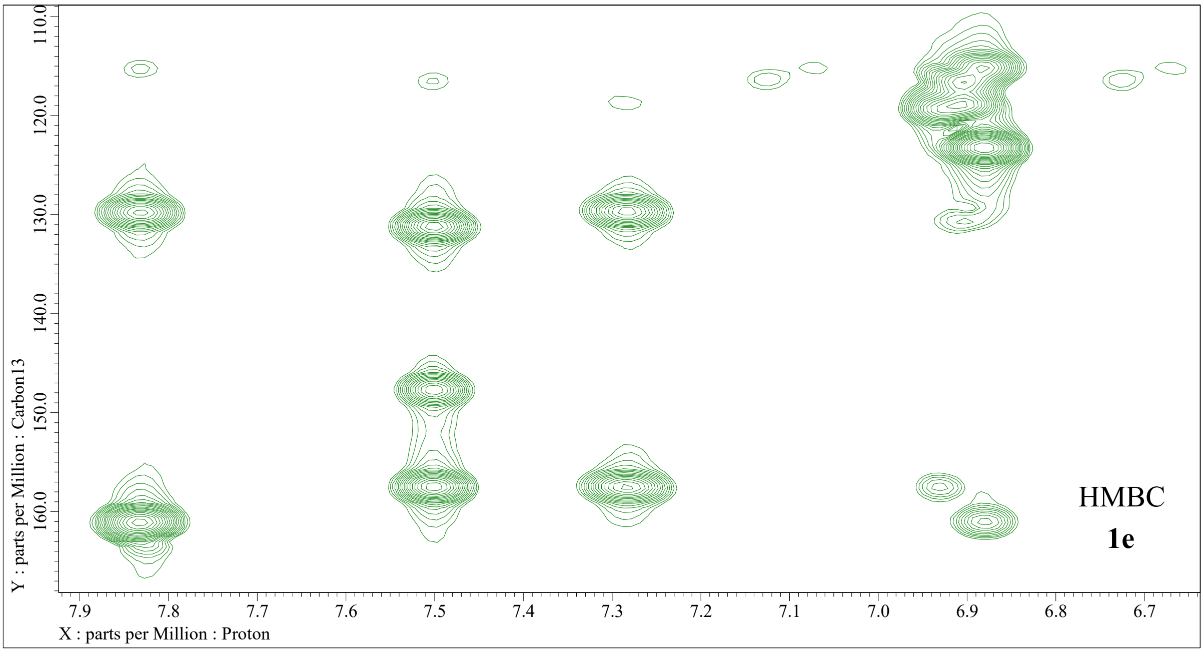

Figure S52. Expansion of 2D-NMR (400 MHz, DMSO-d_6) HMBC experiment of 4-hydroxy-*N'*-[(*E*)-[2-hydroxyphenyl)methylidene]benzohydrazide (**1e**).

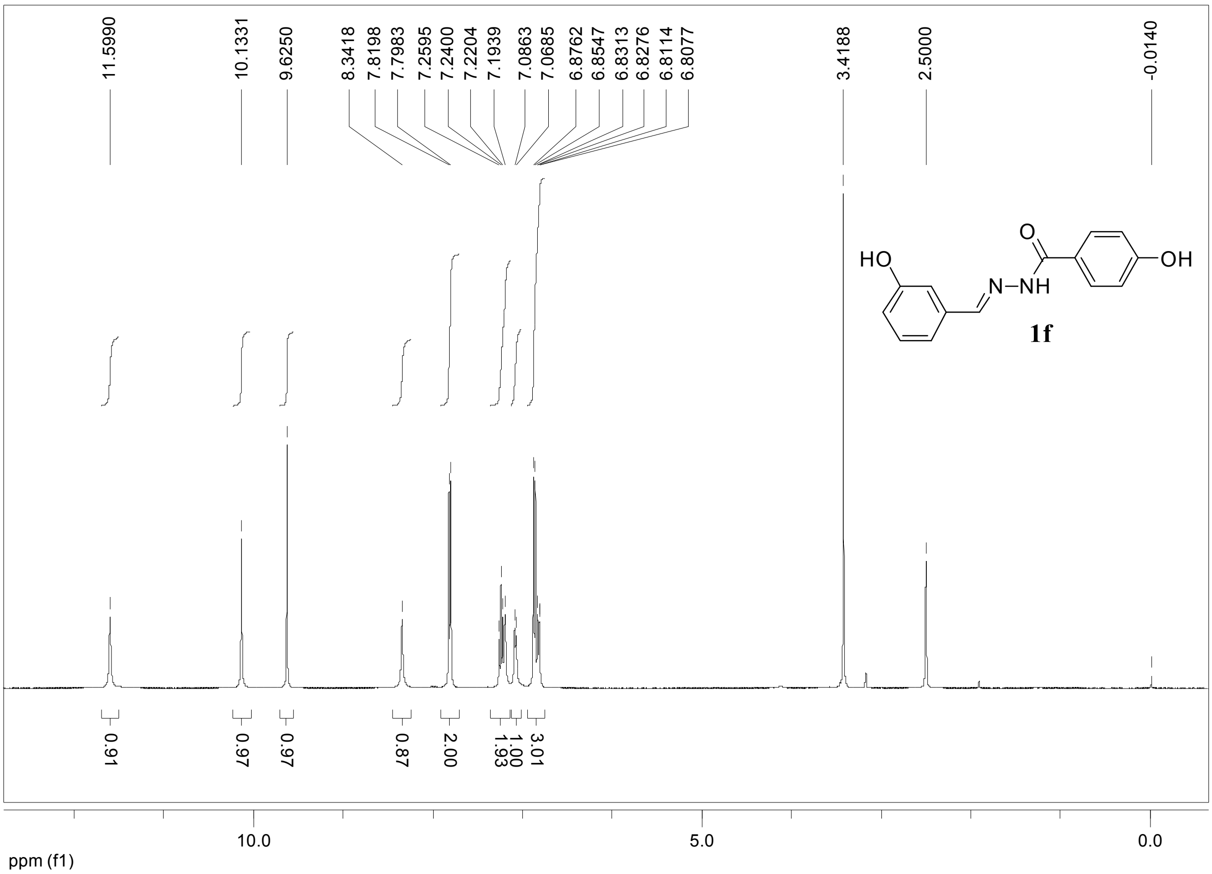

Figure S53. ^{1}H-NMR (400 MHz, DMSO-d_6) spectrum of compound **1f**.

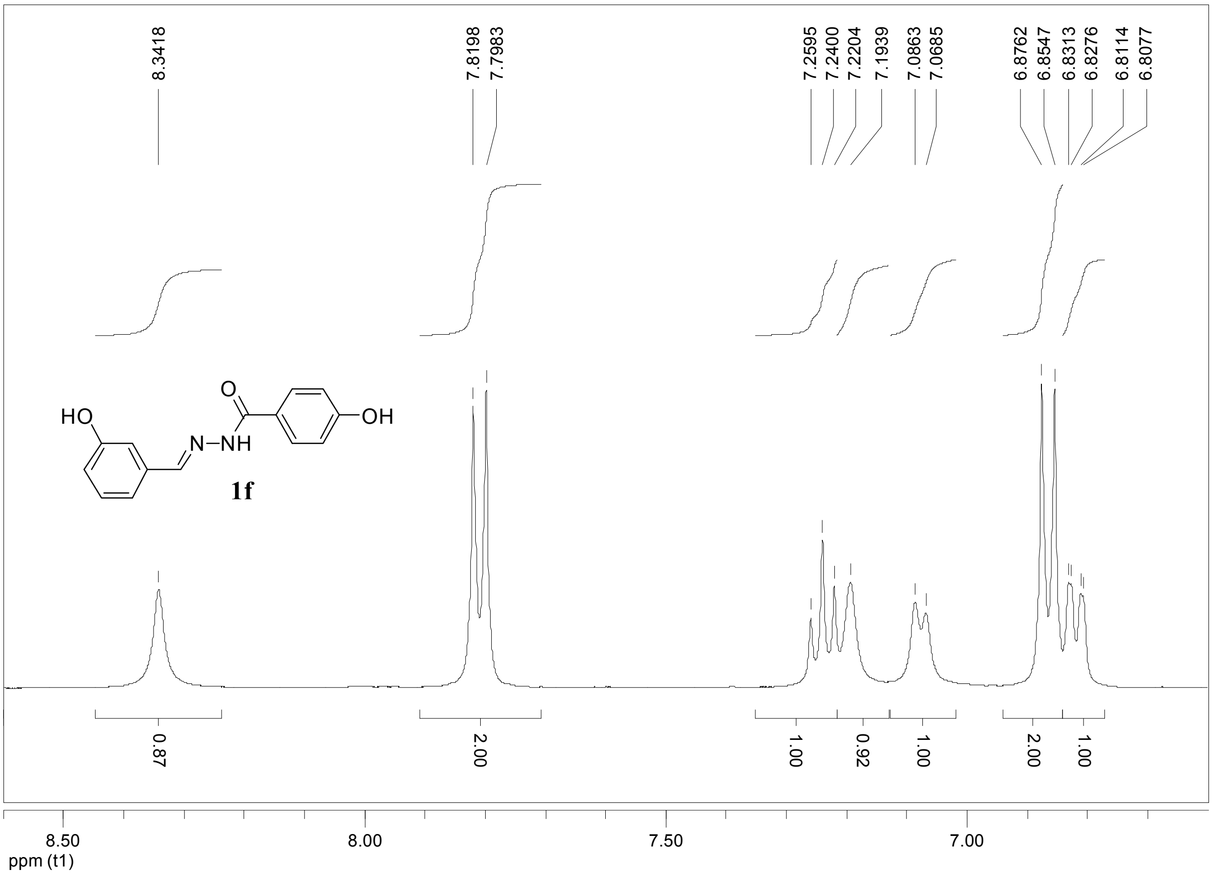

Figure S54. Expansion of ^{1}H-NMR (400 MHz, DMSO-d_6) spectrum of compound **1f**.

Molecules **2020**, *25*, 1255; doi:10.3390/molecules25051255 www.mdpi.com/journal/molecules

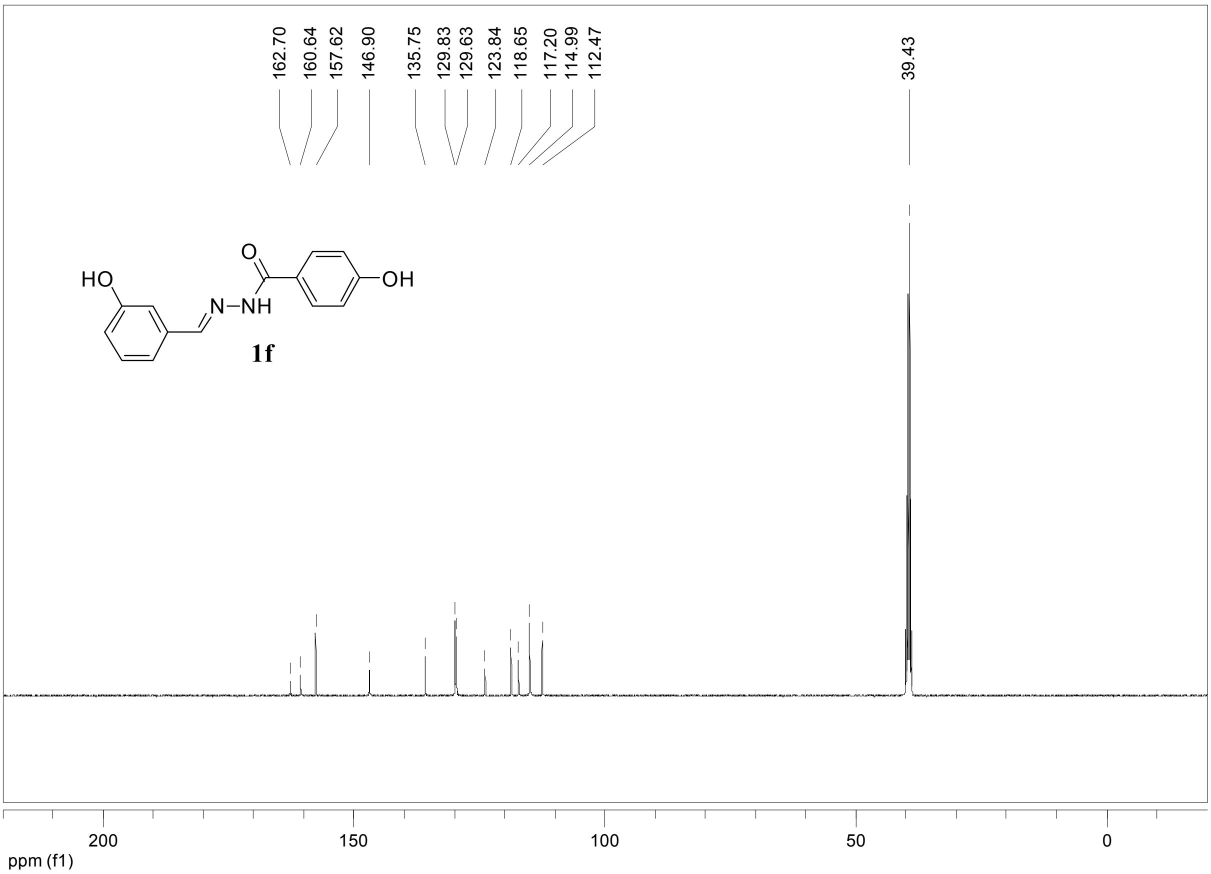

Figure S55. ^{13}C-NMR (100 MHz, DMSO-d_6) spectrum of compound **1f**.

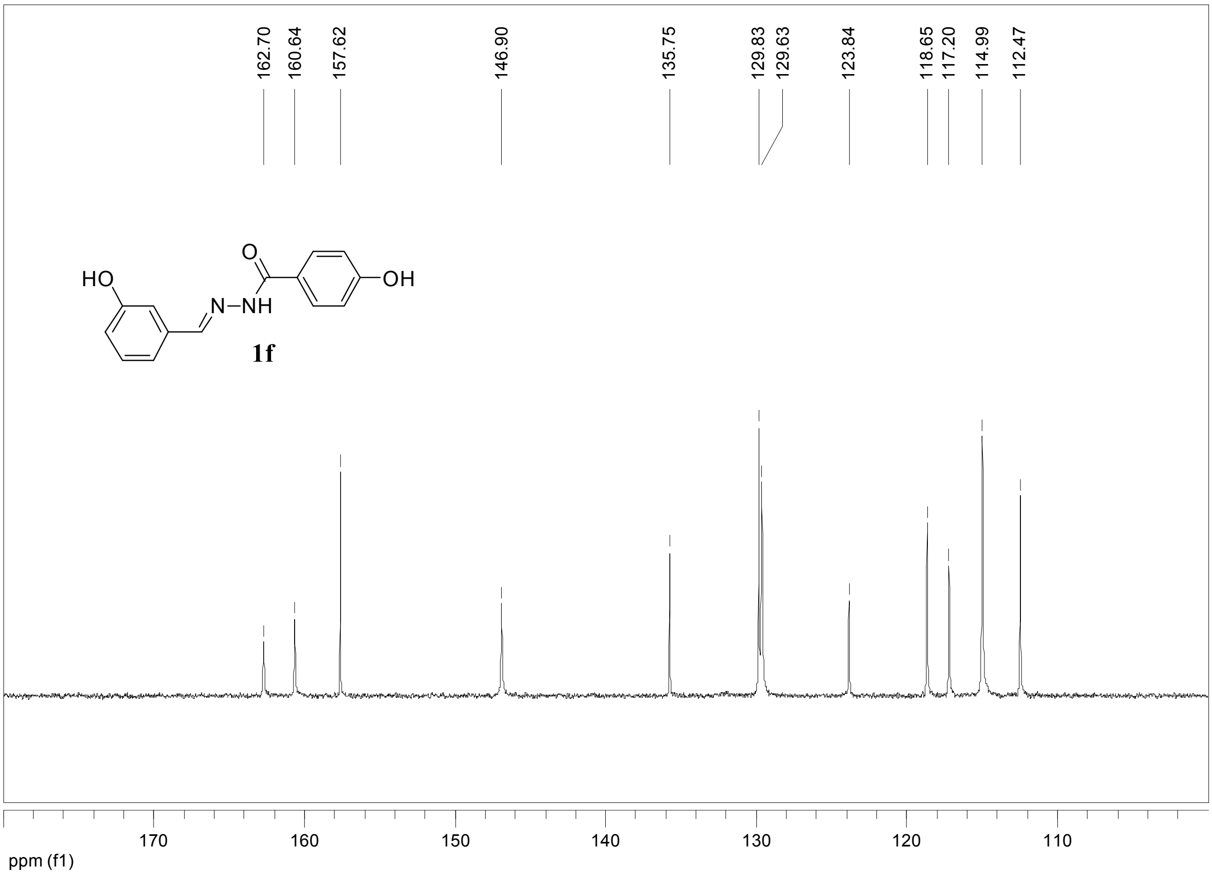

Figure S56. Expansion of ^{13}C-NMR (100 MHz, DMSO-d_6) spectrum of compound **1f**.

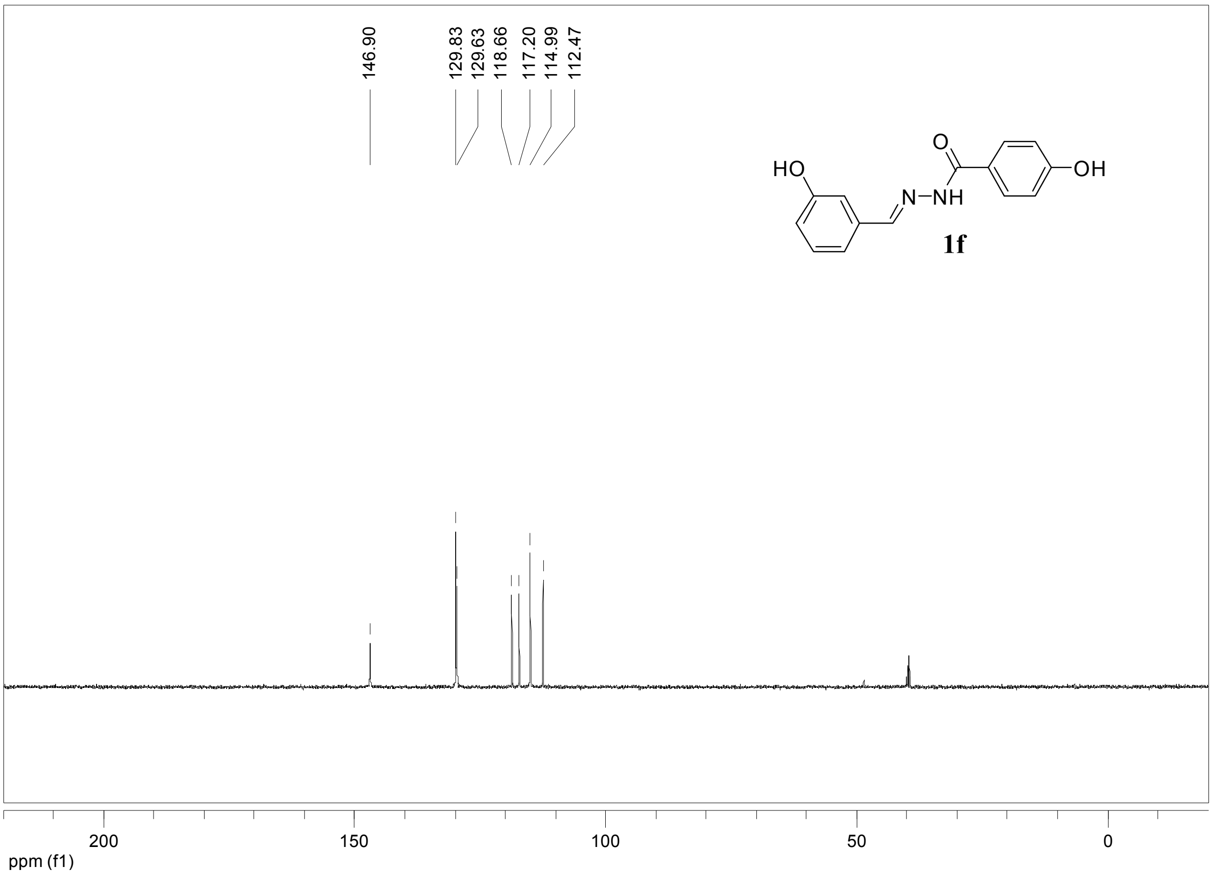

Figure S57. ^{13}C-NMR (100 MHz, DMSO-*d*$_6$) experiment dept-135 of compound **1f**.

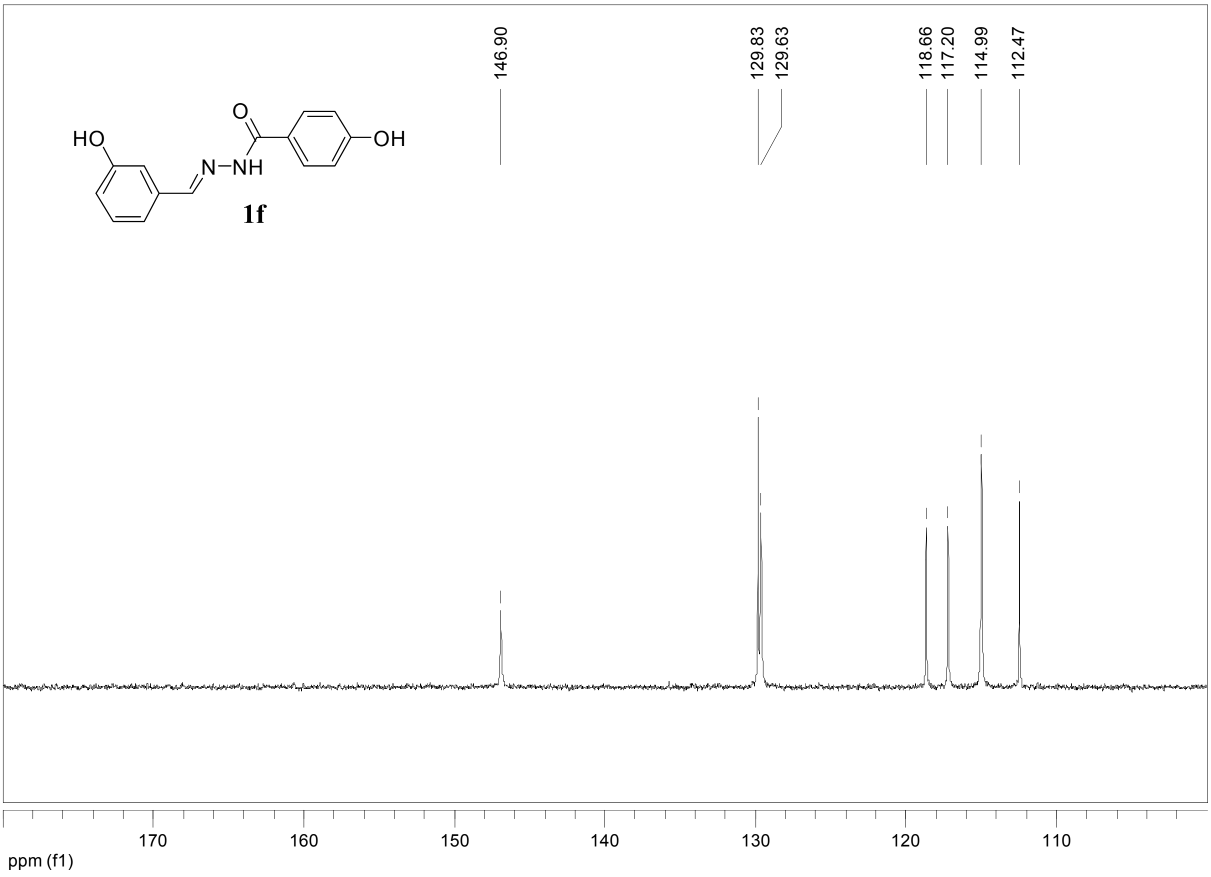

Figure S58. Expansion of ^{13}C-NMR (100 MHz, DMSO-*d*$_6$) dept-135 experiment of compound **1f**.

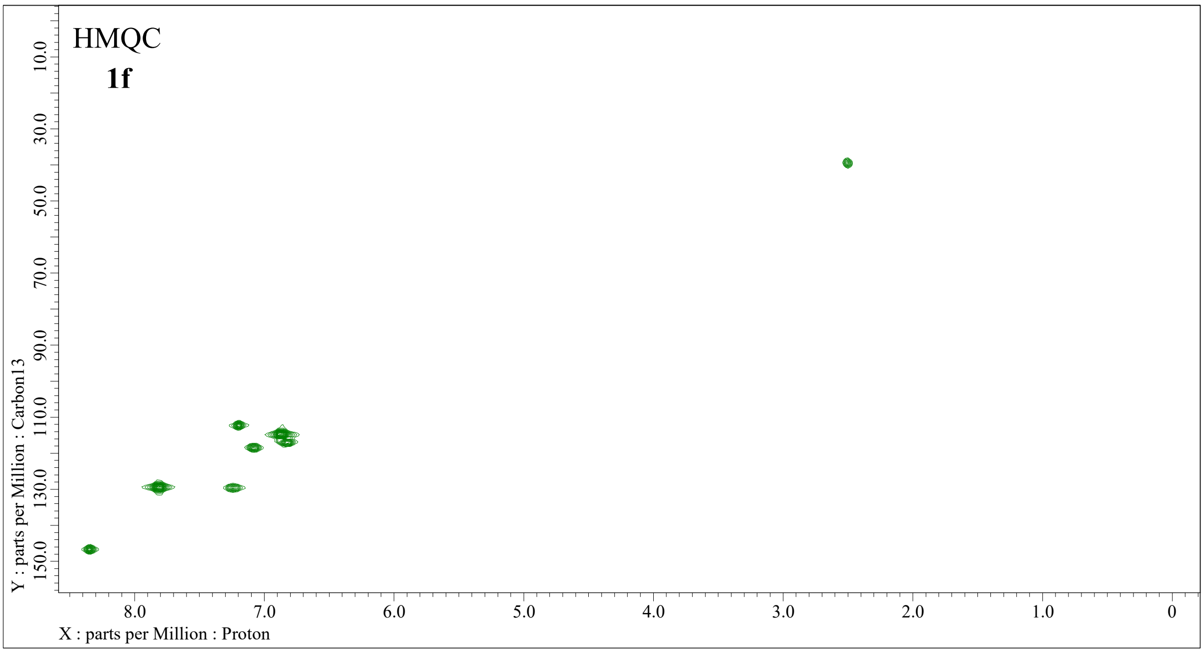

Figure S59. 2D-NMR (400 MHz, DMSO-d_6) HMQC experiment of 4-hydroxy-*N*'-[(*E*)-(3-hydroxyphenyl)methylidene]benzohydrazide (**1f**).

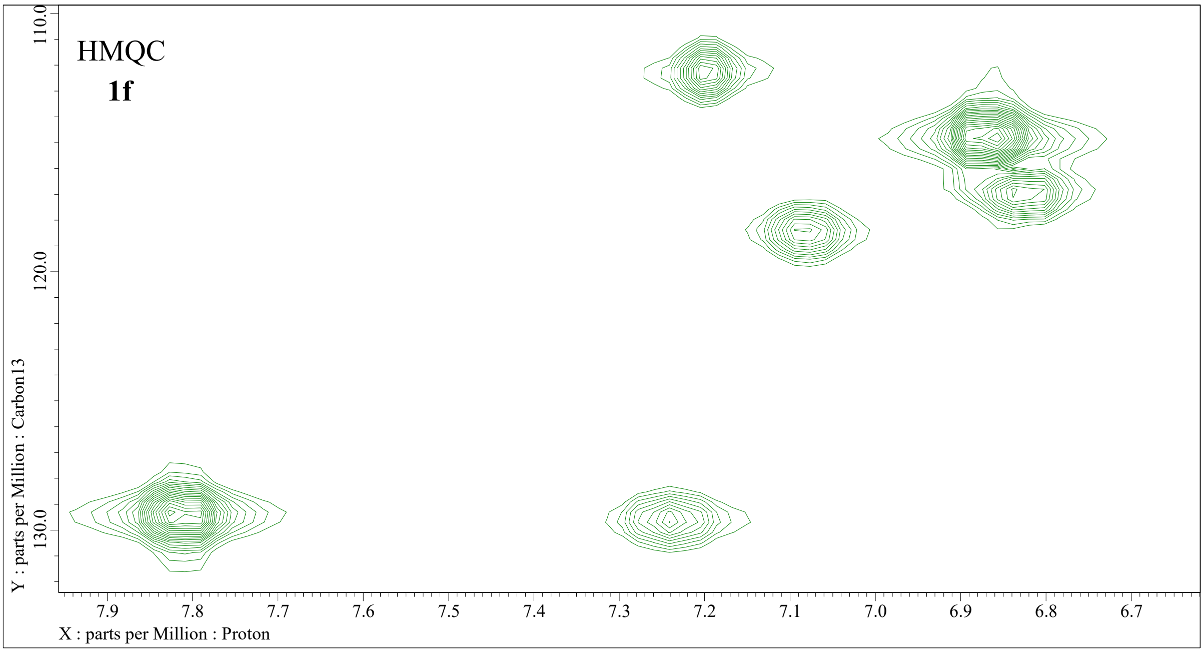

Figure S60. Expansion of 2D-NMR (400 MHz, DMSO-d_6) HMQC experiment of 4-hydroxy-*N*'-[(*E*)-(3-hydroxyphenyl)methylidene]benzohydrazide (**1f**).

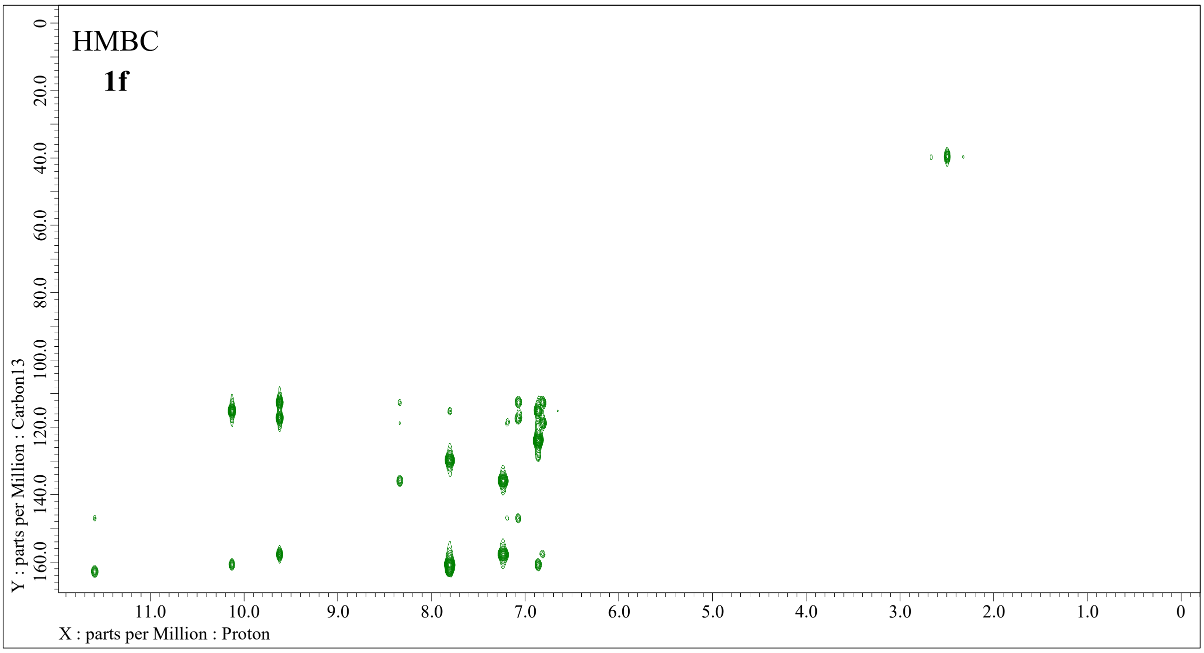

Figure S61. 2D-NMR (400 MHz, DMSO-d_6) HMBC experiment of 4-hydroxy-*N*'-[(*E*)-(3-hydroxyphenyl)methylidene]benzohydrazide (**1f**).

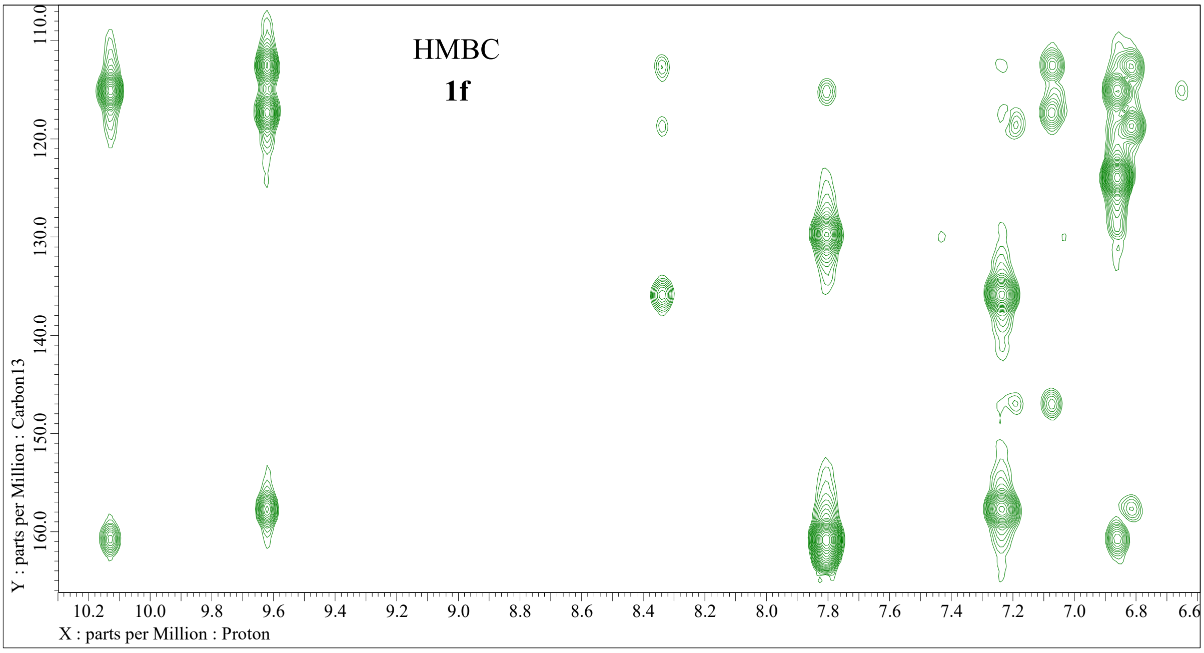

Figure S62. Expansion of 2D-NMR (400 MHz, DMSO-d_6) HMBC experiment of 4-hydroxy-*N*'-[(*E*)-(3-hydroxyphenyl)methylidene]benzohydrazide (**1f**).

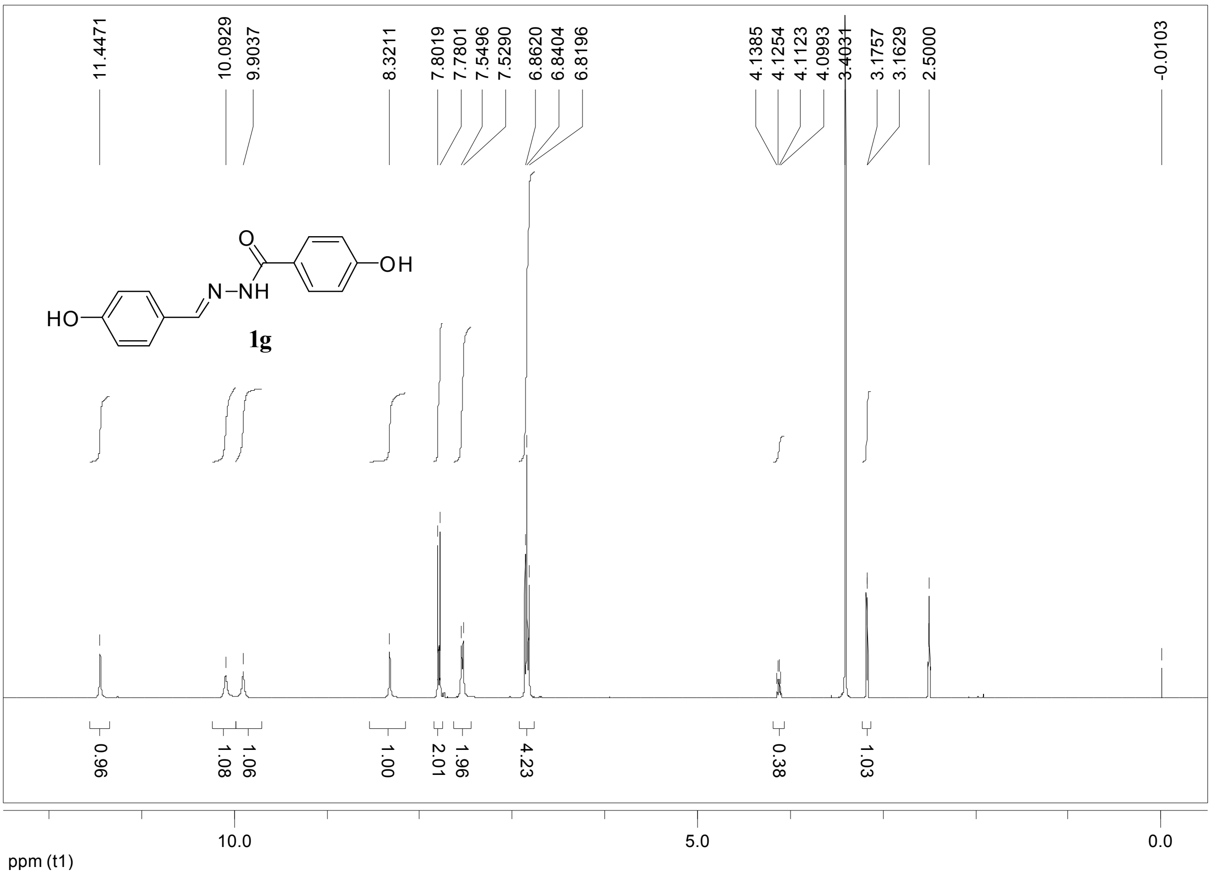

Figure S63. ^{1}H-NMR (400 MHz, DMSO-d_6) spectrum of compound **1g**.

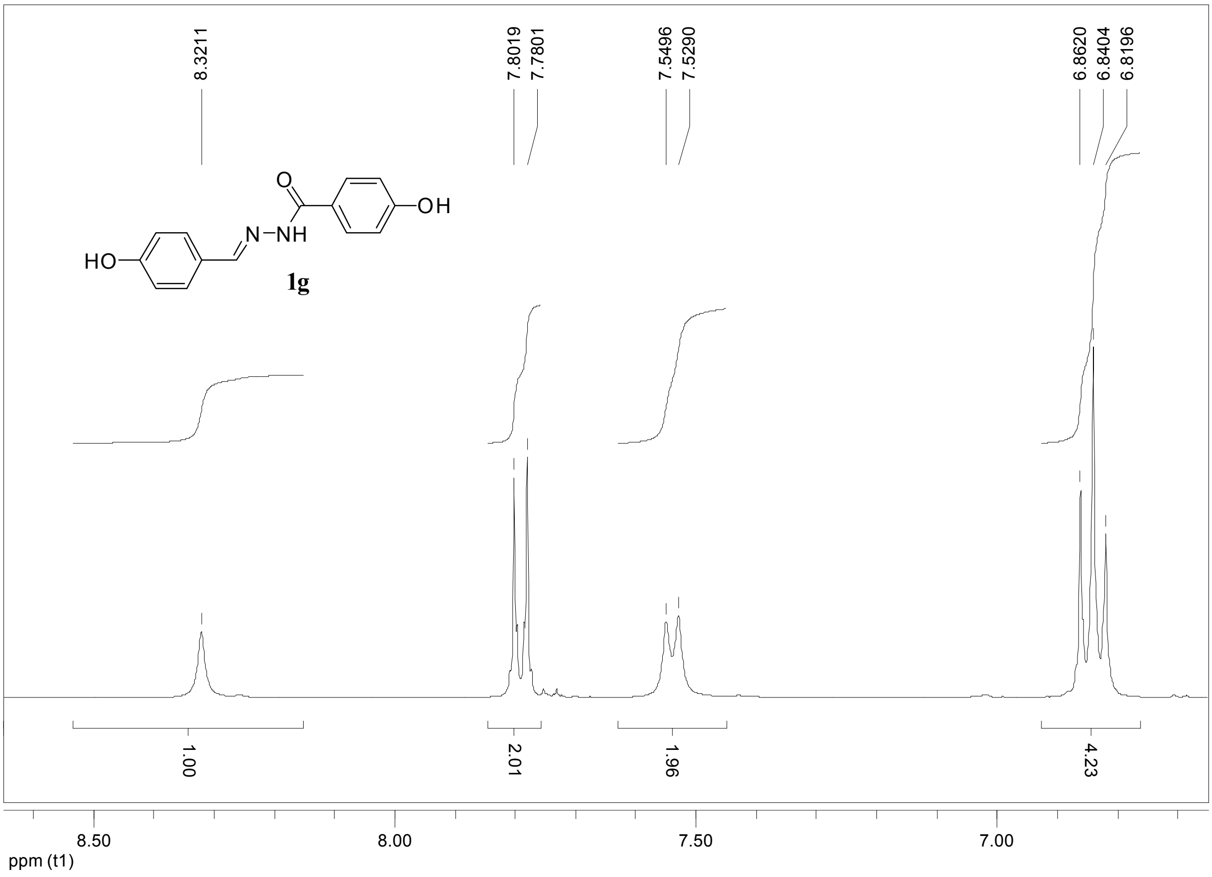

Figure S64. Expansion of ^{1}H-NMR (400 MHz, DMSO-d_6) spectrum of compound **1g**.

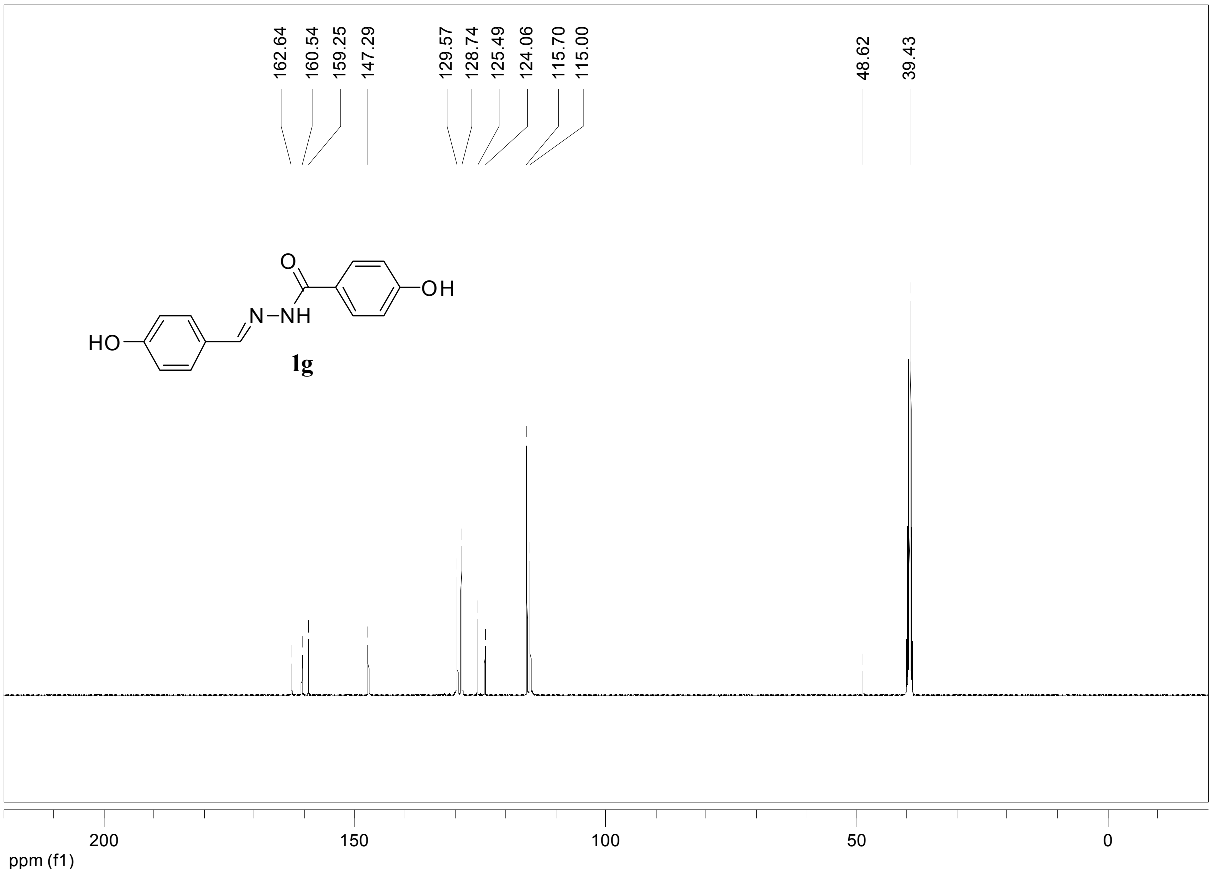

Figure S65. ^{13}C-NMR (100 MHz, DMSO-d_6) spectrum of compound **1g**.

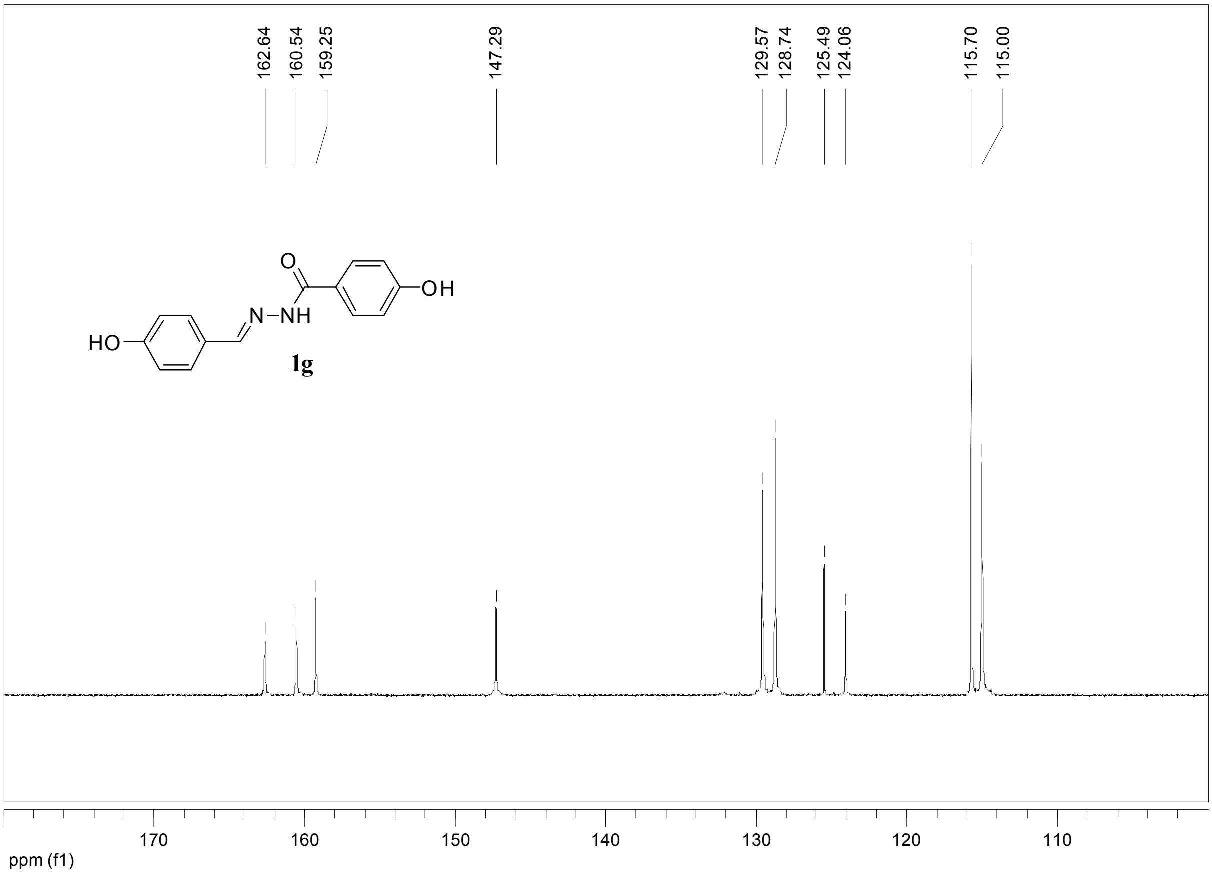

Figure S66. Expansion of ^{13}C-NMR (100 MHz, DMSO-d_6) spectrum of compound **1g**.

Molecules **2020**, *25*, 1255; doi:10.3390/molecules25051255 www.mdpi.com/journal/molecules

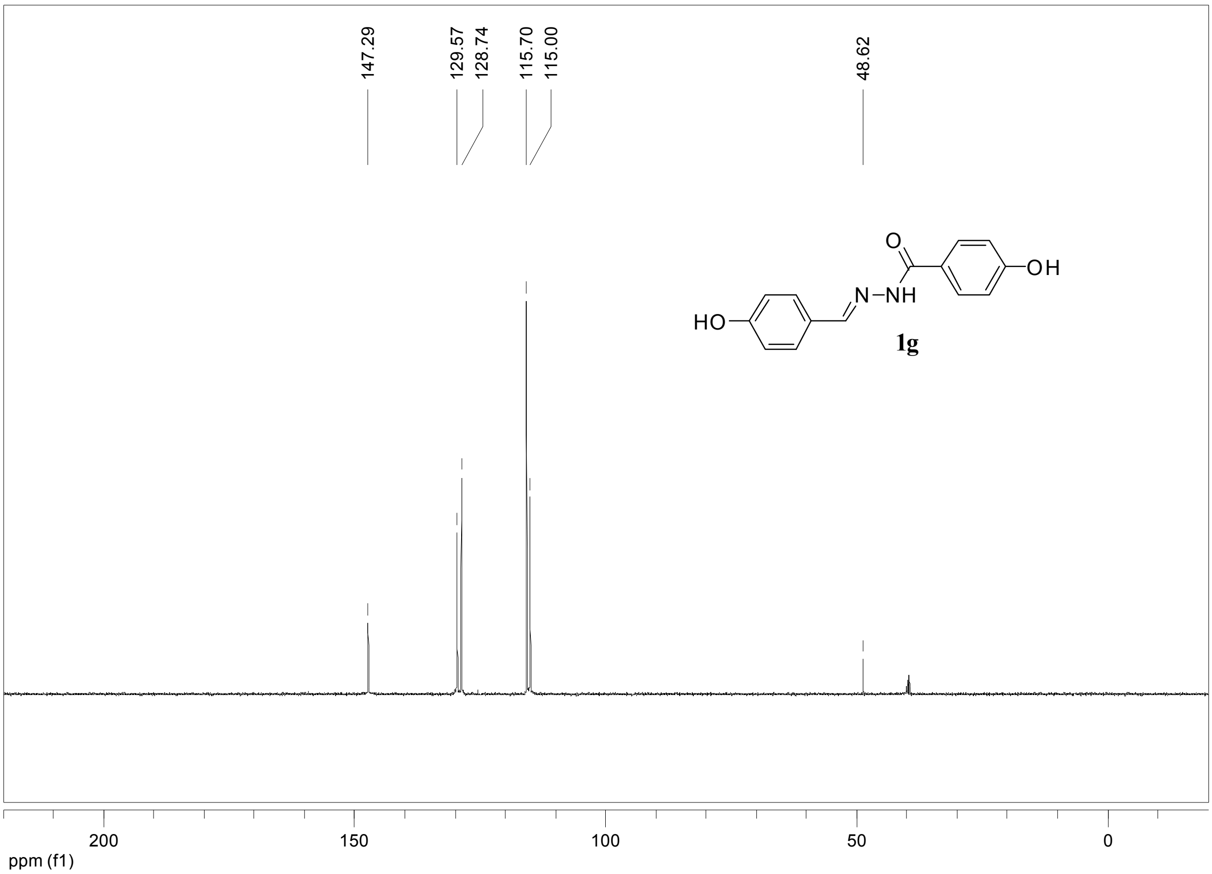

Figure S67. ^{13}C-NMR (100 MHz, DMSO-d_6) experiment dept-135 of compound **1g**.

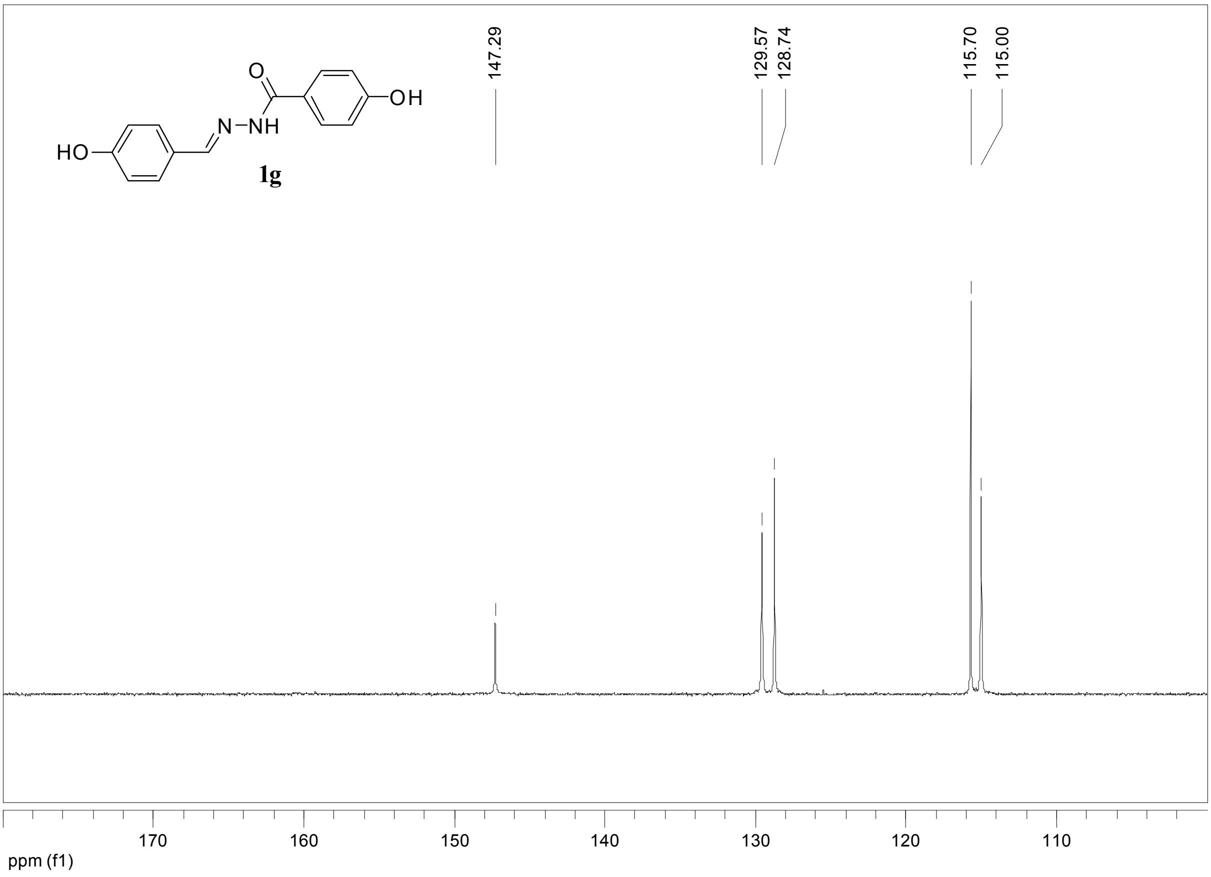

Figure S68. Expansion of ^{13}C-NMR (100 MHz, DMSO-d_6) experiment dept-135 of compound **1g**.

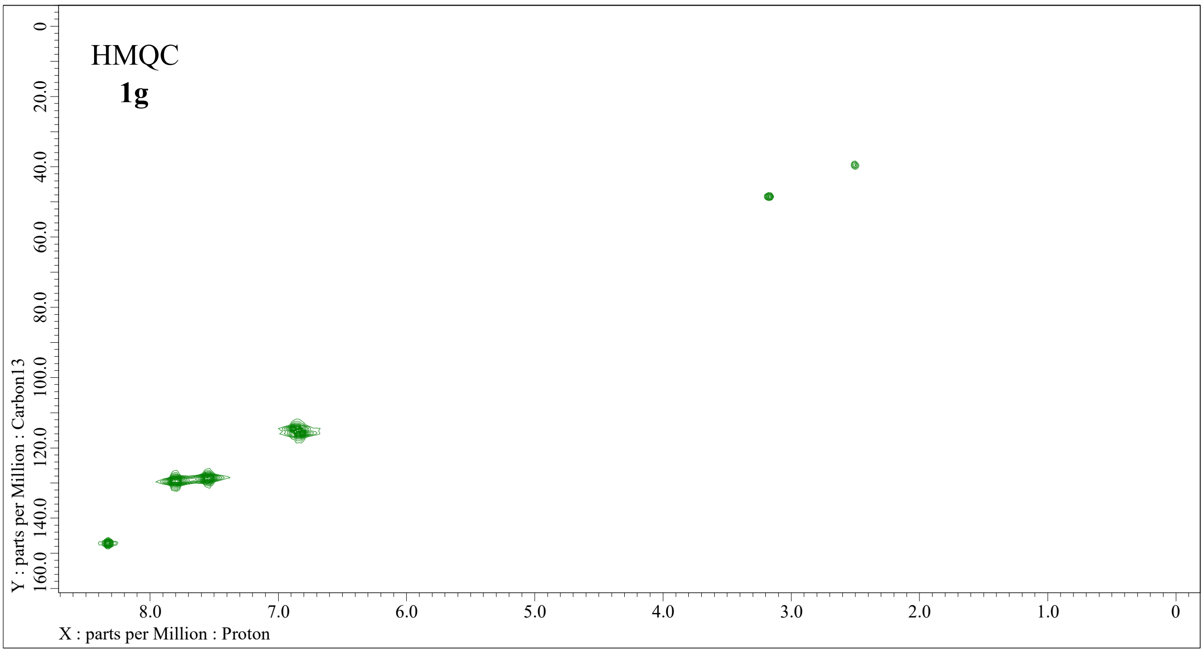

Figure S69. 2D-NMR (400 MHz, DMSO-d_6) HMQC experiment of 4-hydroxy-*N*'-[(*E*)-(4-hydroxyphenyl)methylidene]benzohydrazide (**1g**).

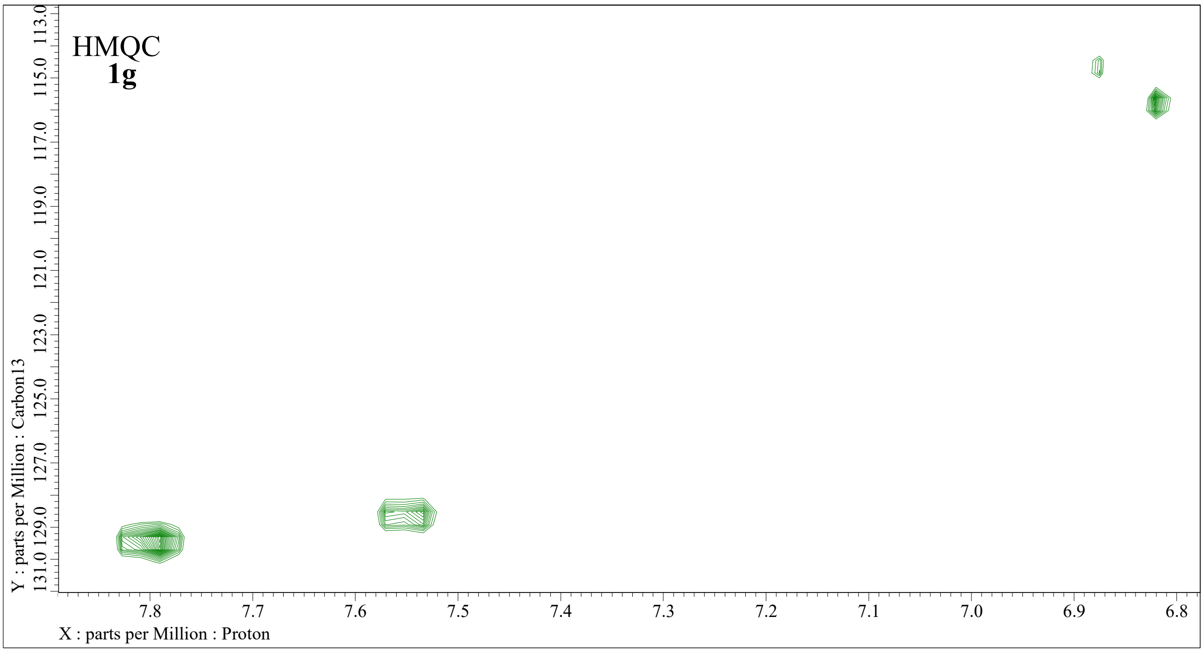

Figure S70. Expansion of 2D-NMR (400 MHz, DMSO-d_6) HMQC experiment of 4-hydroxy-*N*'-[(*E*)-(4-hydroxyphenyl)methylidene]benzohydrazide (**1g**).

Molecules **2020**, *25*, 1255; doi:10.3390/molecules25051255 www.mdpi.com/journal/molecules

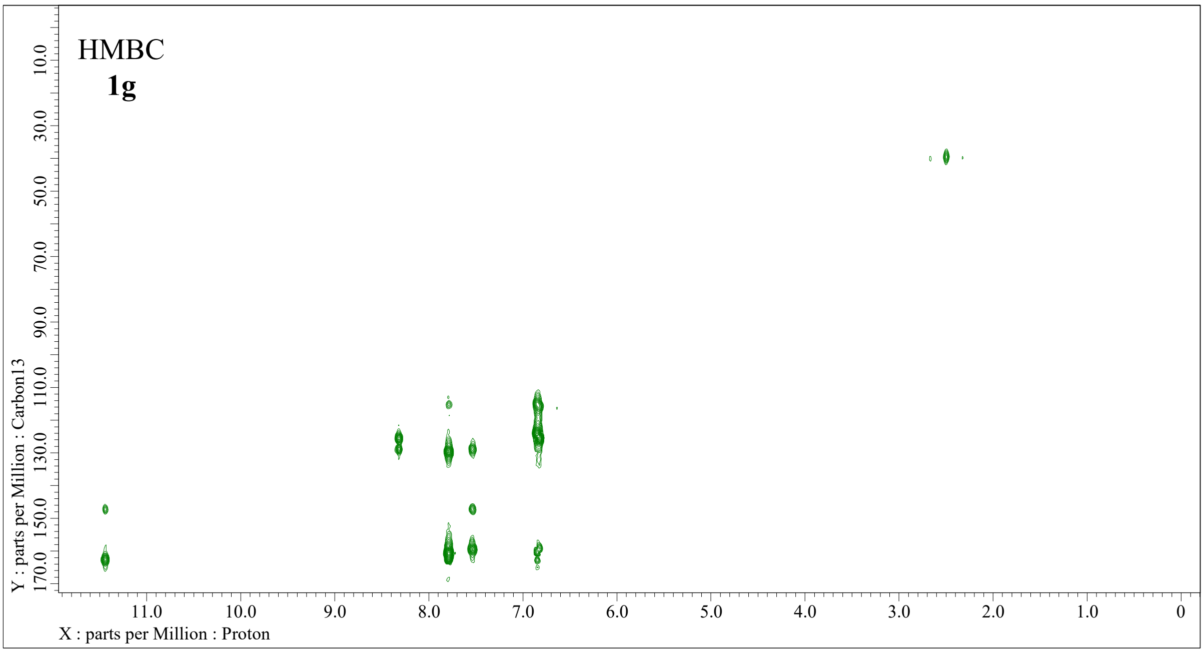

Figure S71. 2D-NMR (400 MHz, DMSO-d_6) HMBC experiment of 4-hydroxy-*N*'-[(*E*)-(4-hydroxyphenyl)methylidene]benzohydrazide (**1g**).

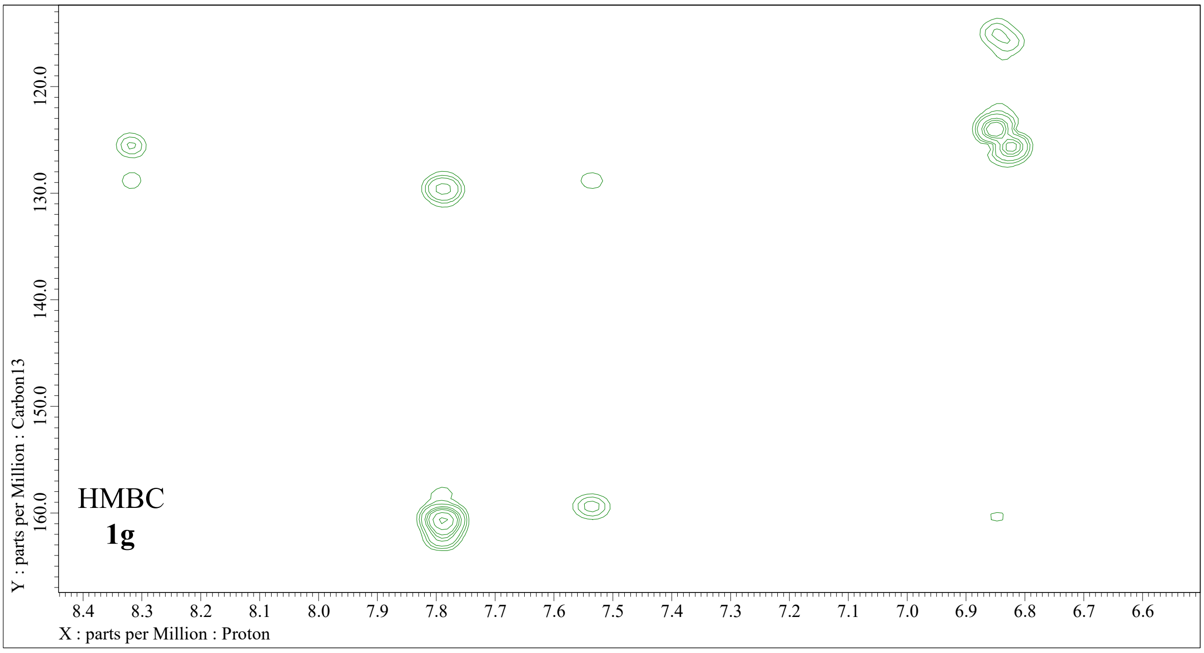

Figure S72. Expansion of 2D-NMR (400 MHz, DMSO-d_6) HMBC experiment of 4-hydroxy-*N*'-[(*E*)-(4-hydroxyphenyl)methylidene]benzohydrazide (**1g**).

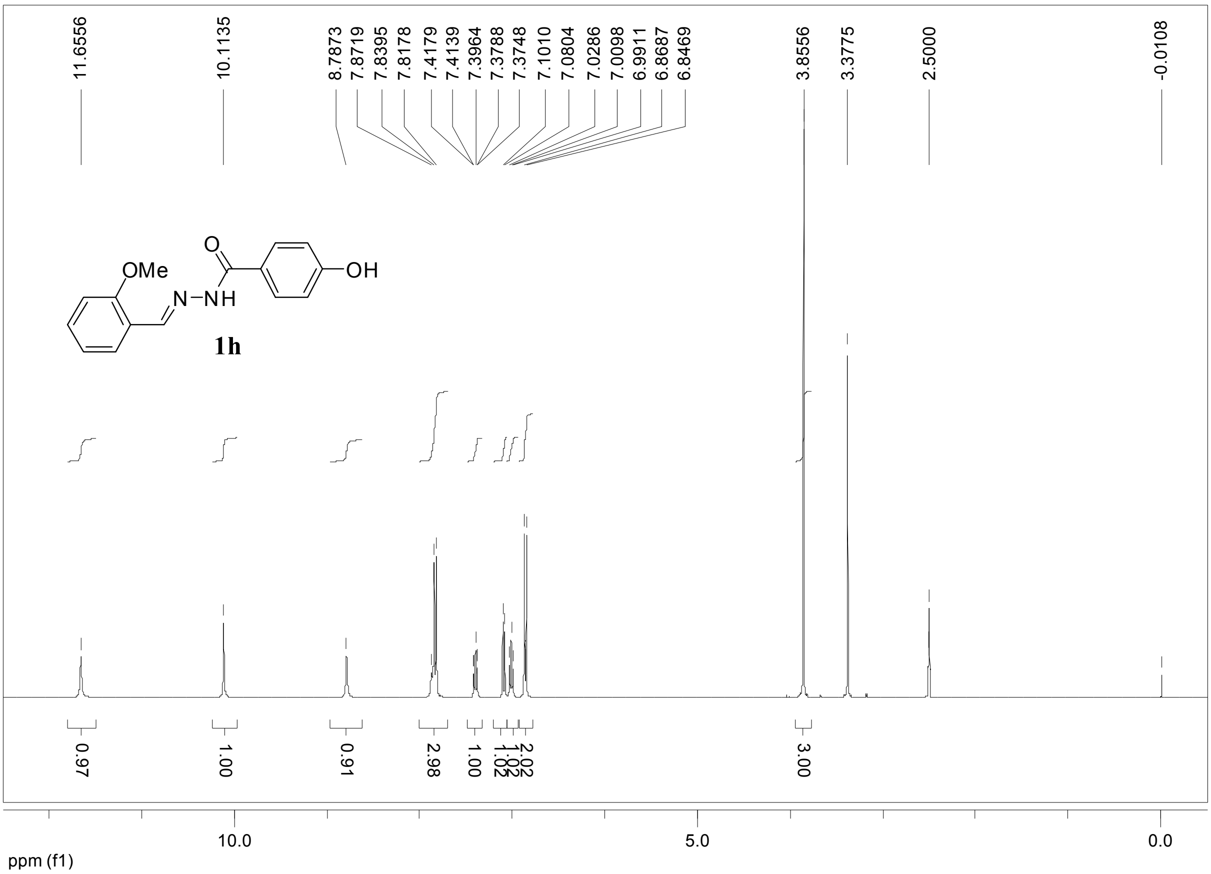

Figure S73. ^{1}H-NMR (400 MHz, DMSO-d_6) spectrum of compound **1h**.

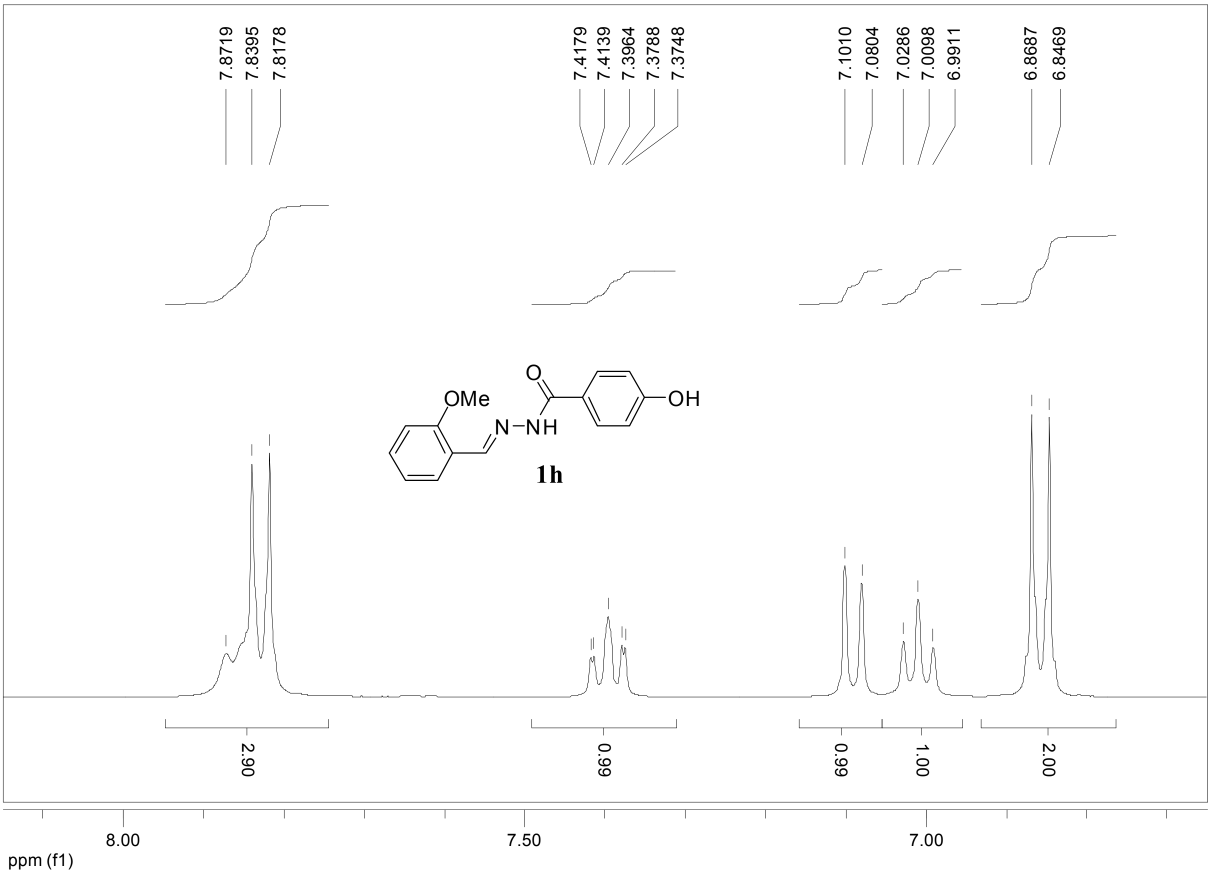

Figure S74. Expansion of ^{1}H-NMR (400 MHz, DMSO-d_6) spectrum of compound **1h**.

Molecules **2020**, *25*, 1255; doi:10.3390/molecules25051255 www.mdpi.com/journal/molecules

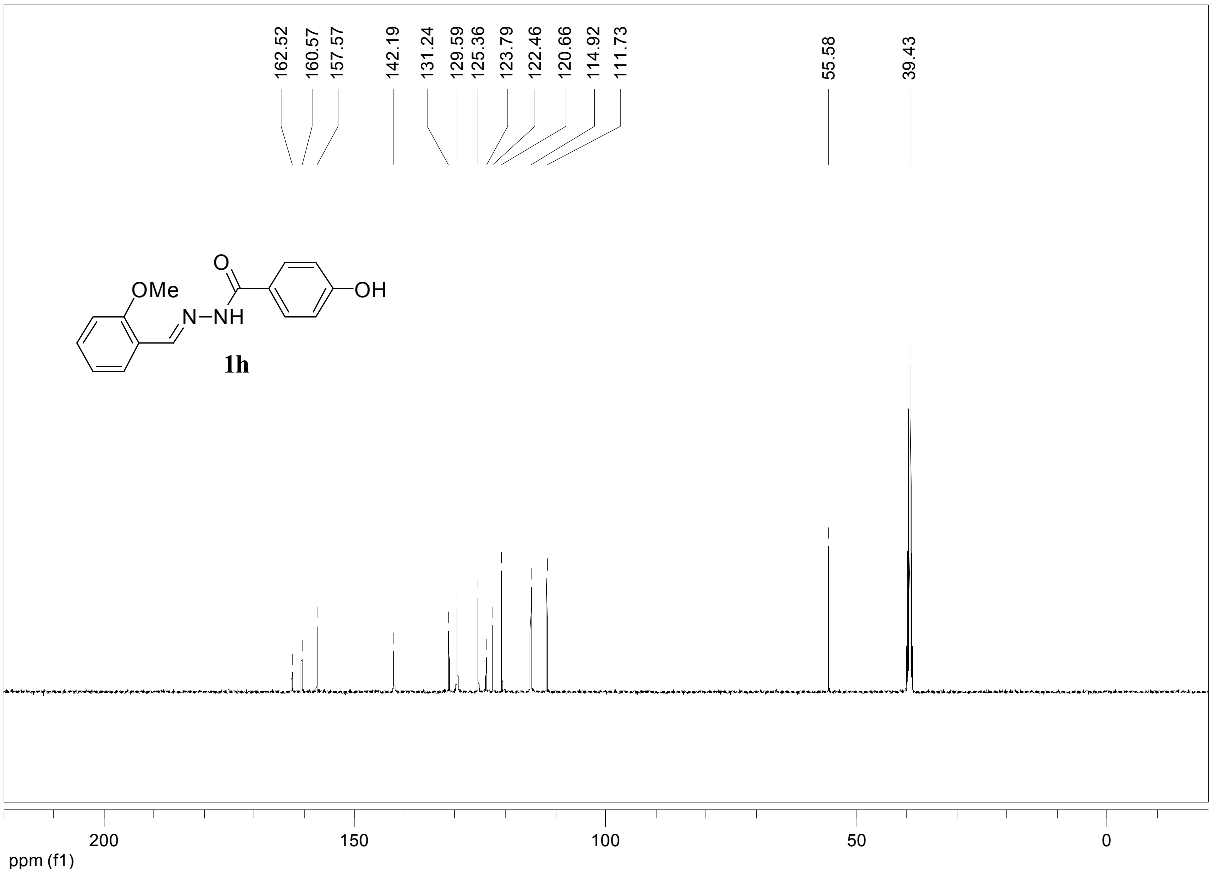

Figure S75. ^{13}C-NMR (100 MHz, DMSO-d_6) spectrum of compound **1h**.

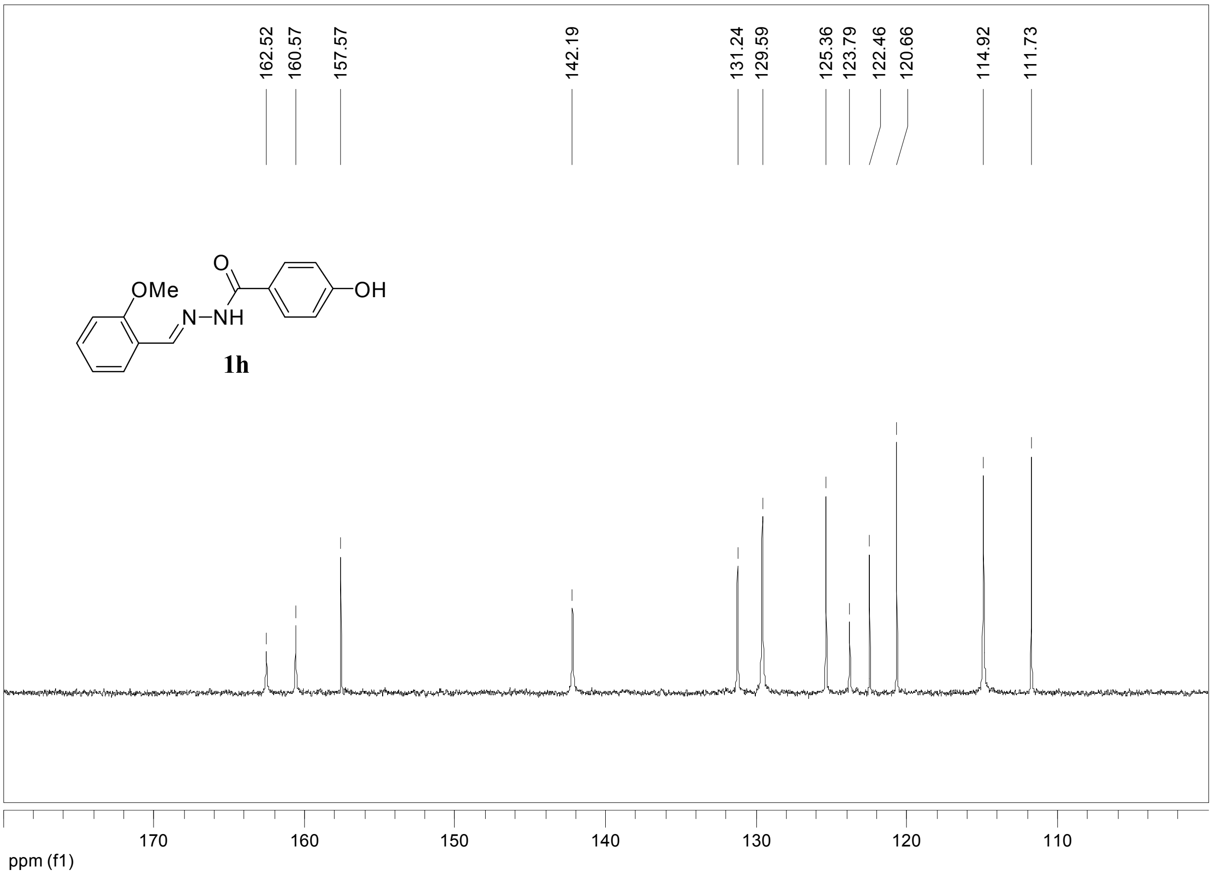

Figure S76. Expansion of ^{13}C-NMR (100 MHz, DMSO-d_6) spectrum of compound **1h**.

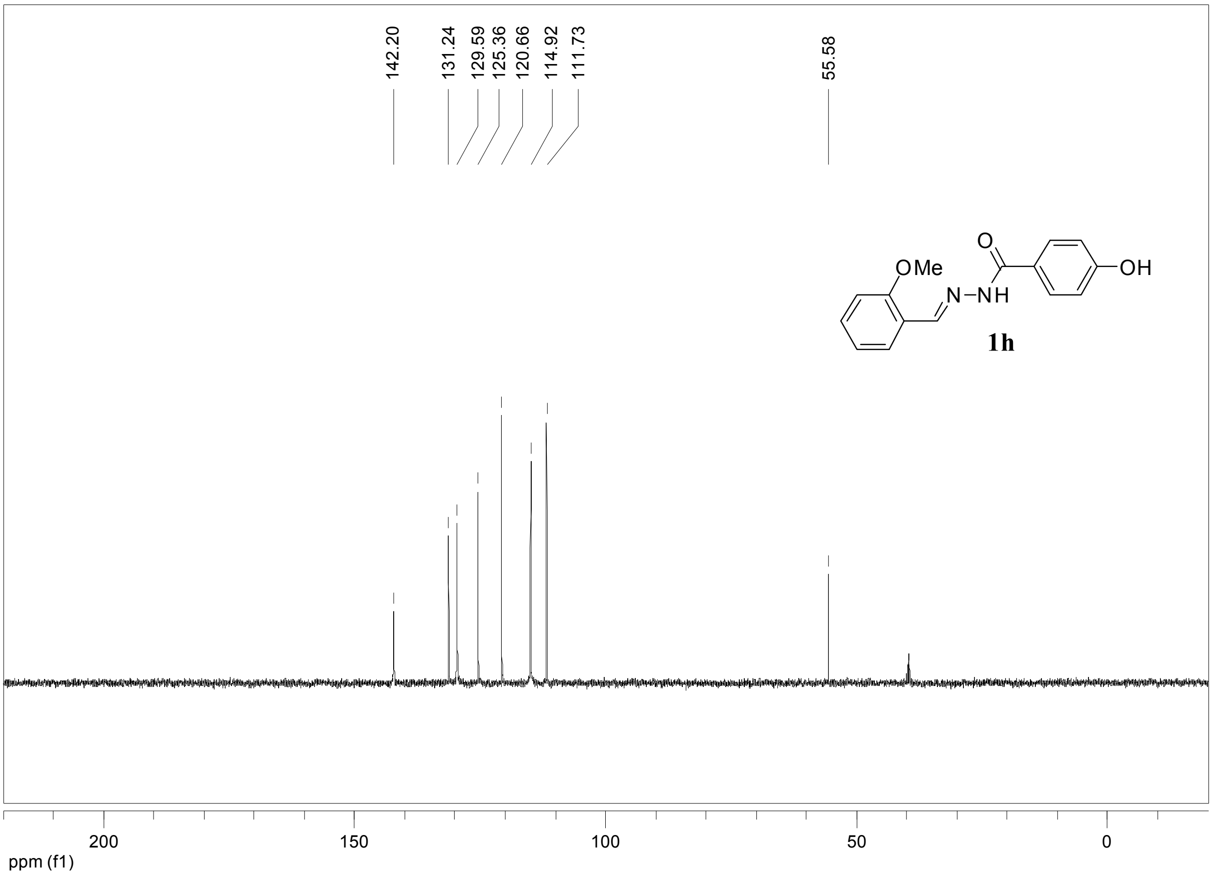

Figure S77. ^{13}C-NMR (100 MHz, DMSO-*d*$_6$) dept-135 experiment of compound **1h**.

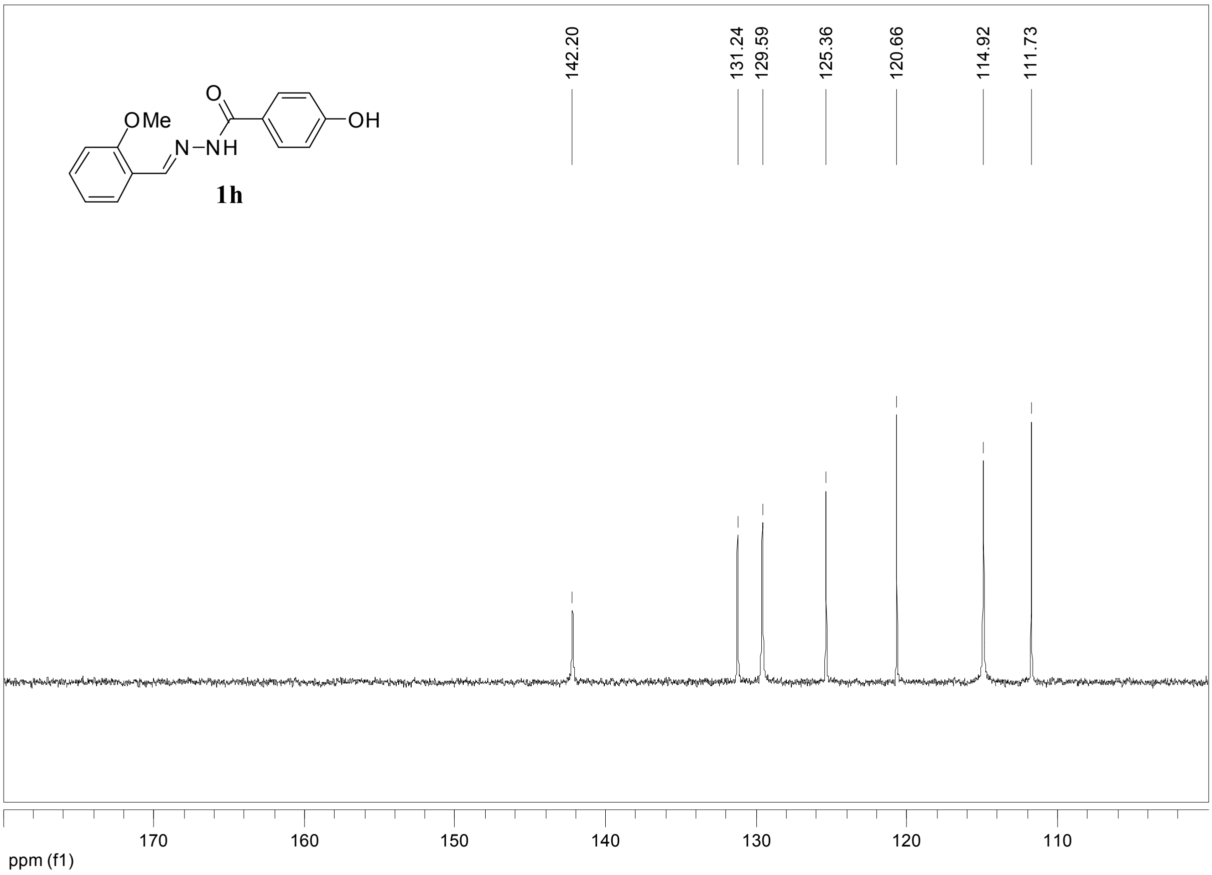

Figure S78. Expansion of ^{13}C-NMR (100 MHz, DMSO-*d*$_6$) dept-135 experiment of compound **1h**.

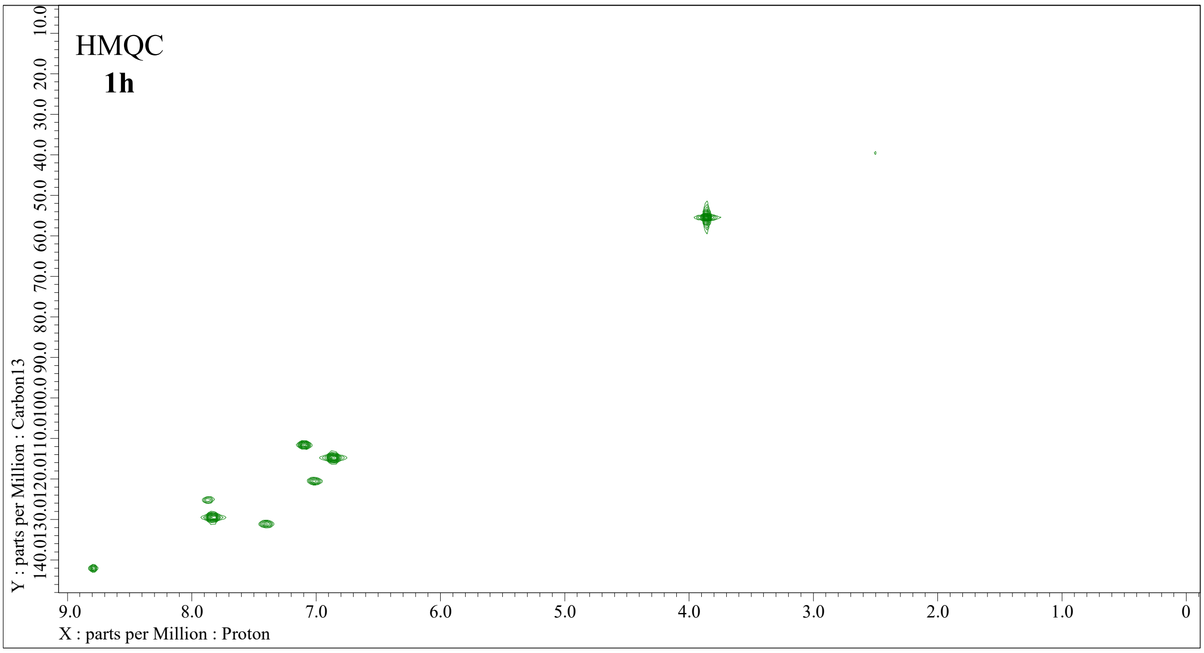

Figure S79. 2D-NMR (400 MHz, DMSO-d_6) HMQC experiment of 4-hydroxy-*N*'-[(*E*)-(2-methoxyphenyl)methylidene]benzohydrazide (**1h**).

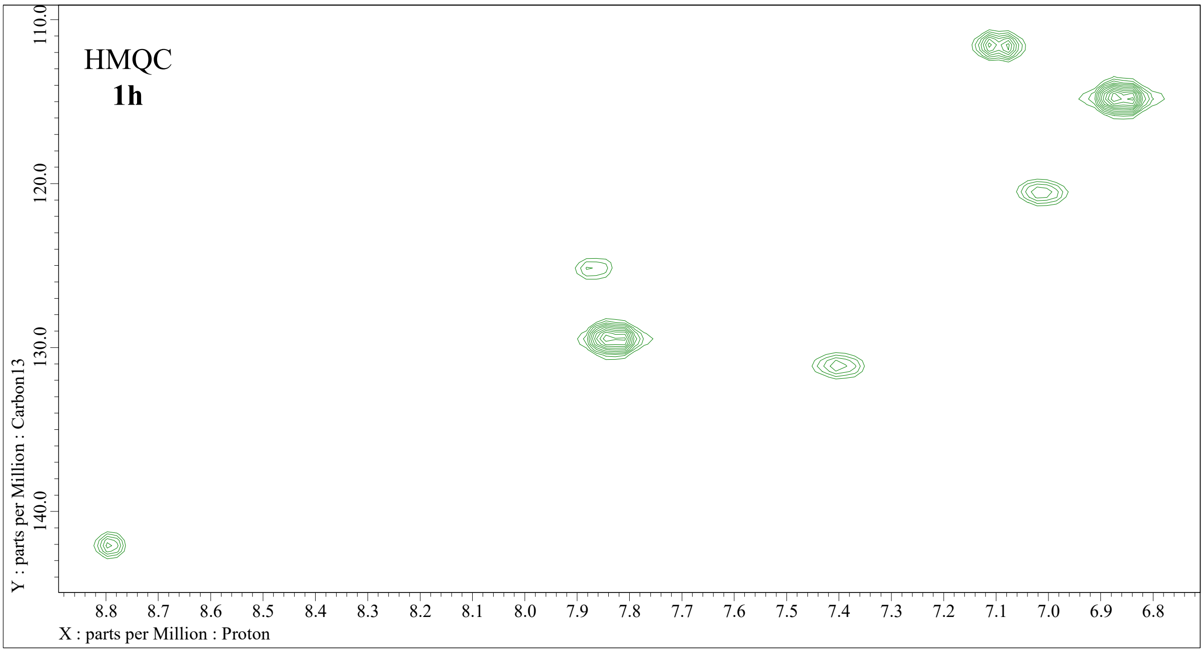

Figure S80. Expansion of 2D-NMR (400 MHz, DMSO-d_6) HMQC experiment of 4-hydroxy-*N*'-[(*E*)-(2-methoxyphenyl)methylidene]benzohydrazide (**1h**).

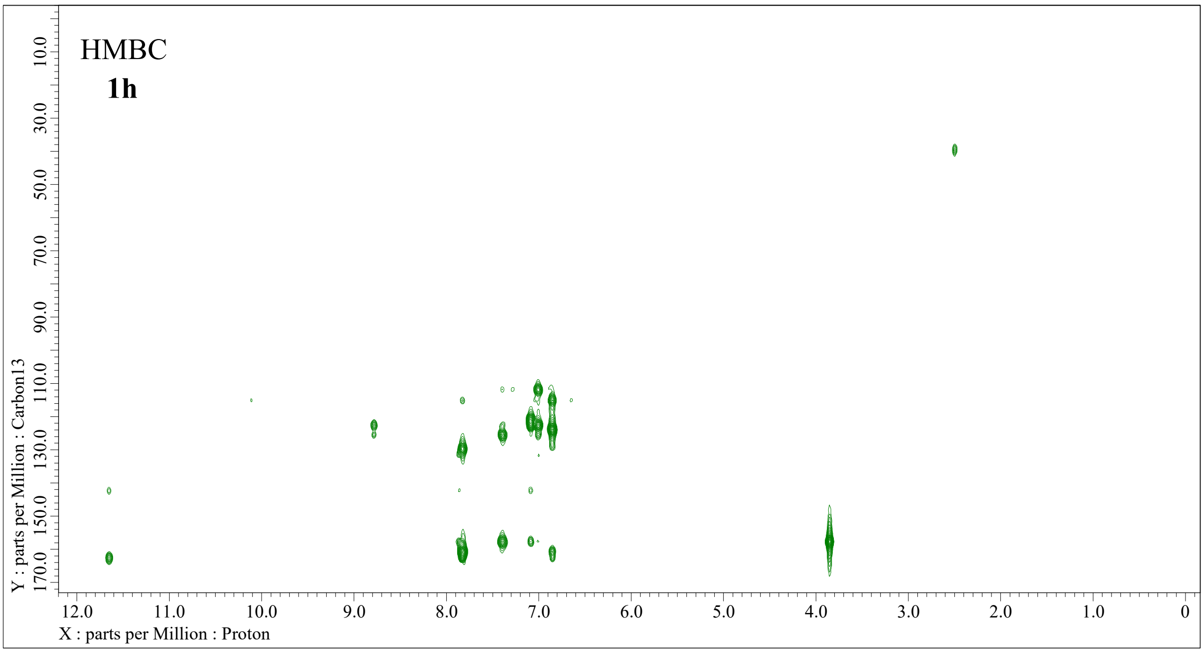

Figure S81. 2D-NMR (400 MHz, DMSO-d_6) HMBC experiment of 4-hydroxy-*N*'-[(*E*)-(2-methoxyphenyl)methylidene]benzohydrazide (**1h**).

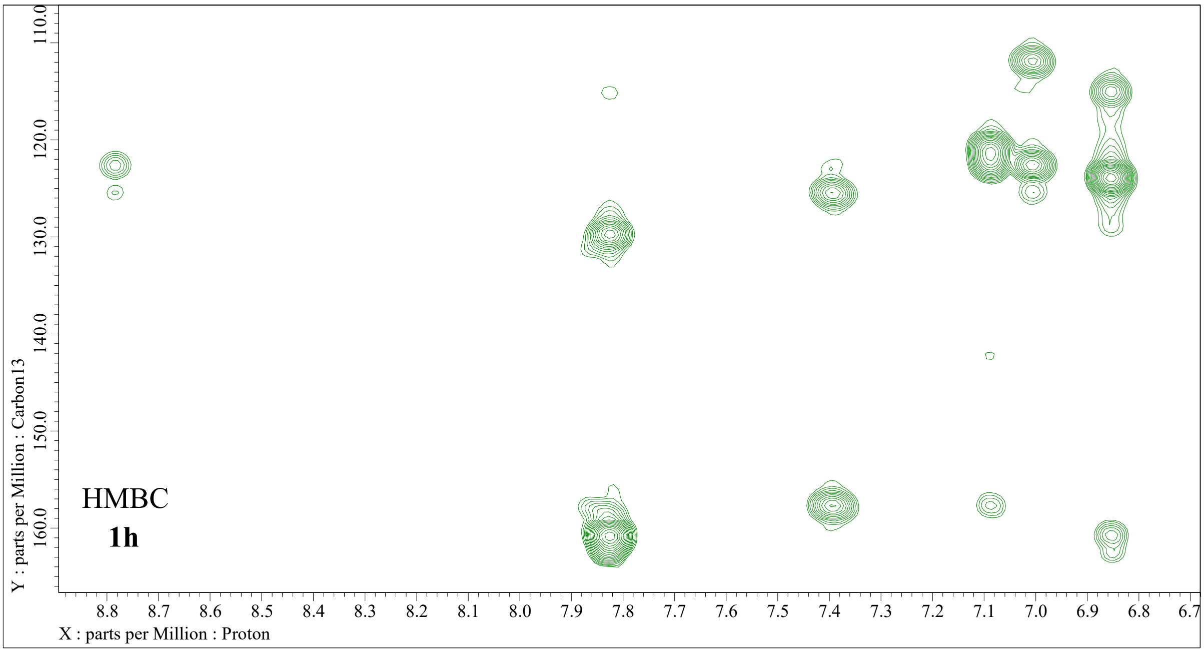

Figure S82. Expansion of 2D-NMR (400 MHz, DMSO-d_6) HMBC experiment of 4-hydroxy-*N*'-[(*E*)-(2-methoxyphenyl)methylidene]benzohydrazide (**1h**).

Molecules **2020**, *25*, 1255; doi:10.3390/molecules25051255 www.mdpi.com/journal/molecules

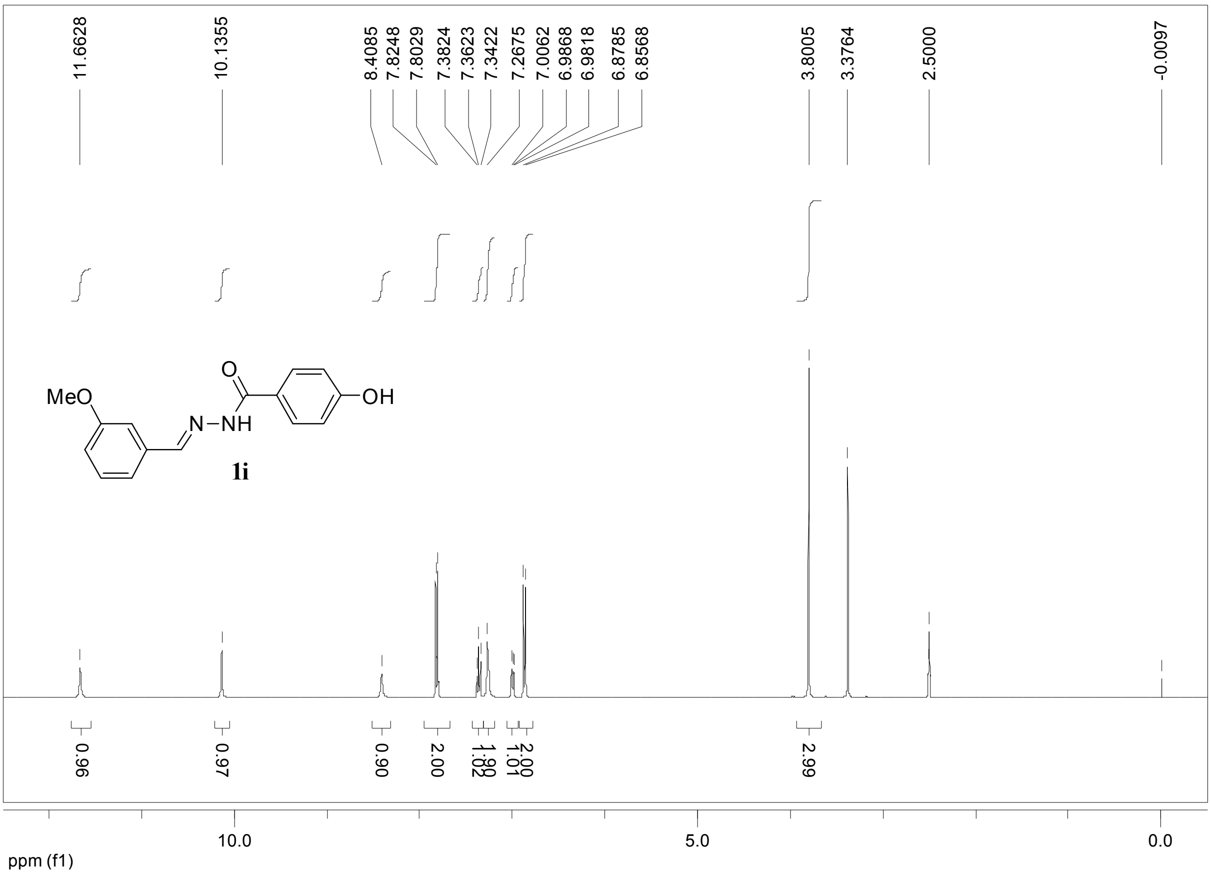

Figure S83. ^{1}H-NMR (400 MHz, DMSO-d_6) spectrum of compound **1i**.

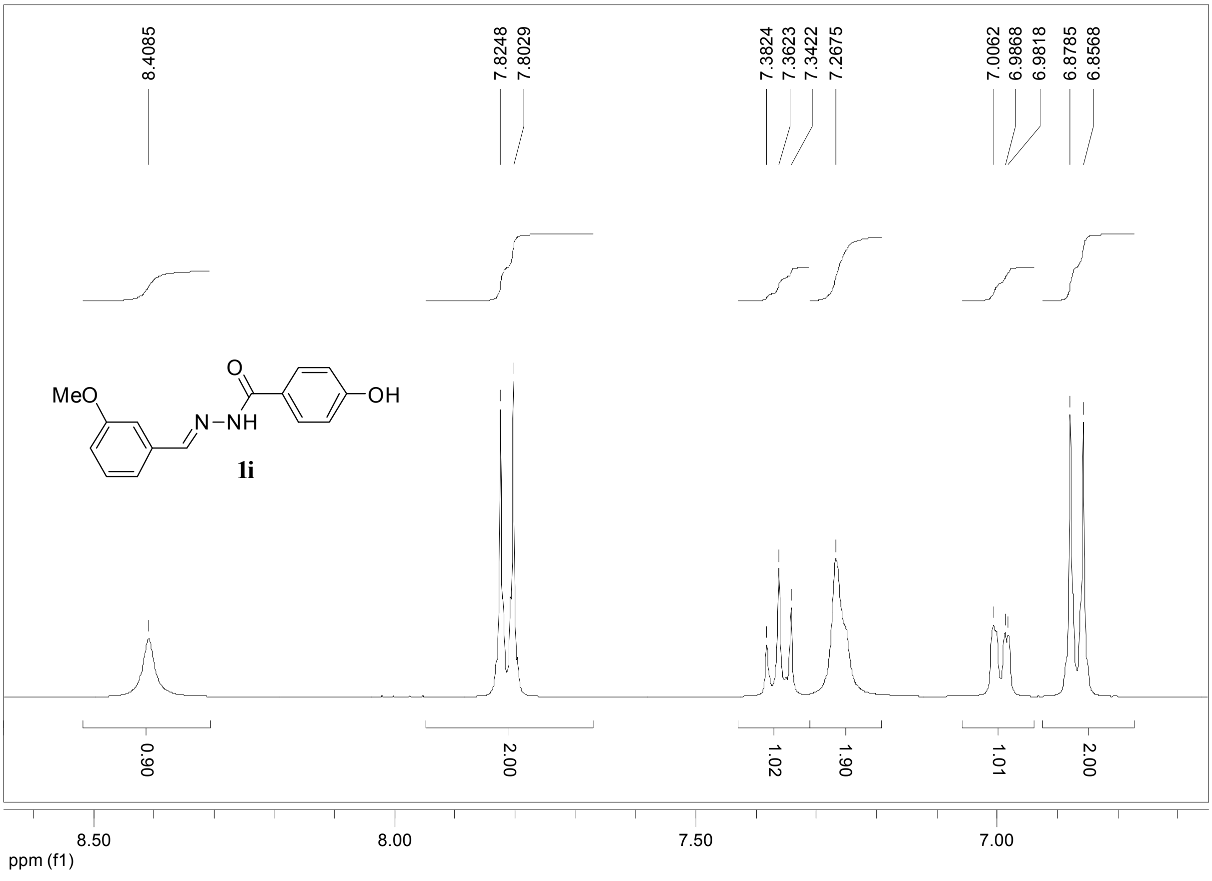

Figure S84. Expansion of ^{1}H-NMR (400 MHz, DMSO-d_6) spectrum of compound **1i**.

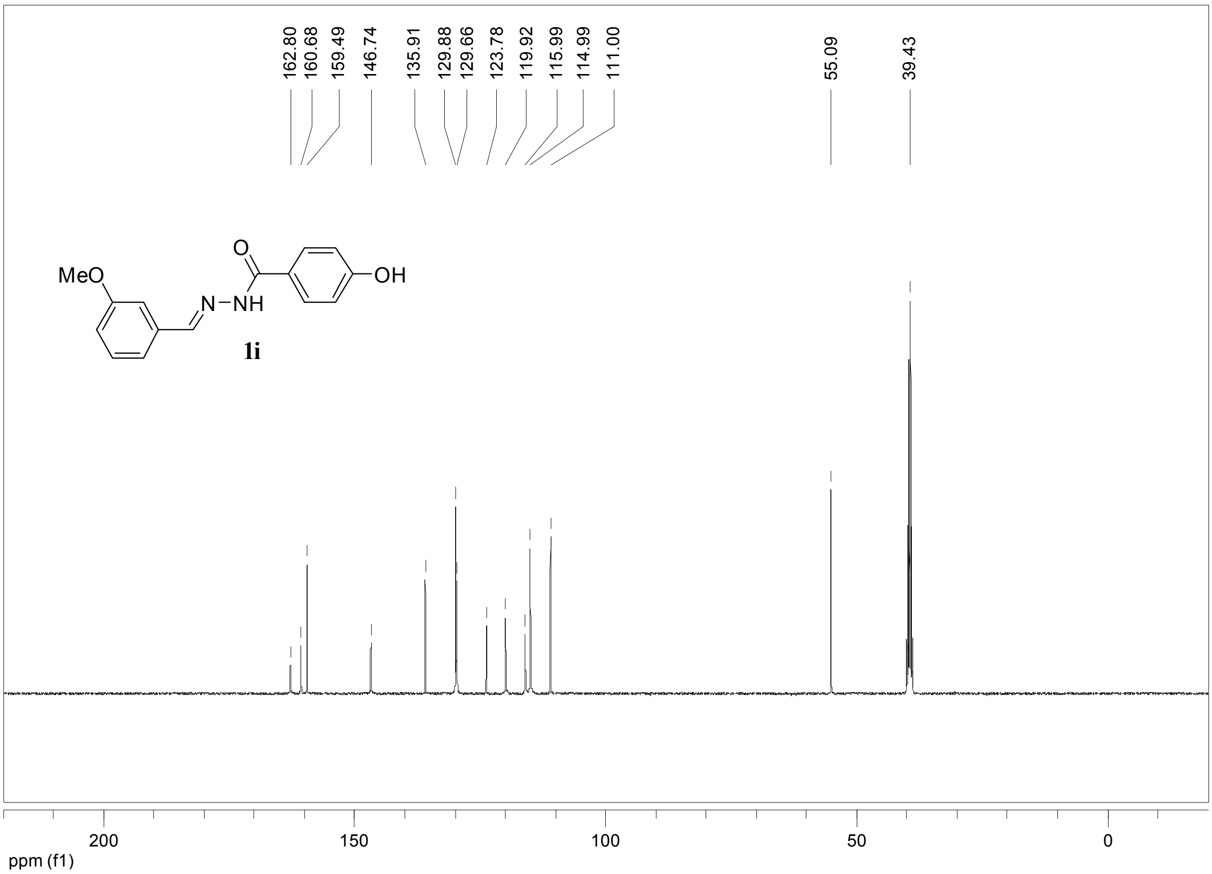

Figure S85. ^{13}C-NMR (100 MHz, DMSO-d_6) spectrum of compound **1i**.

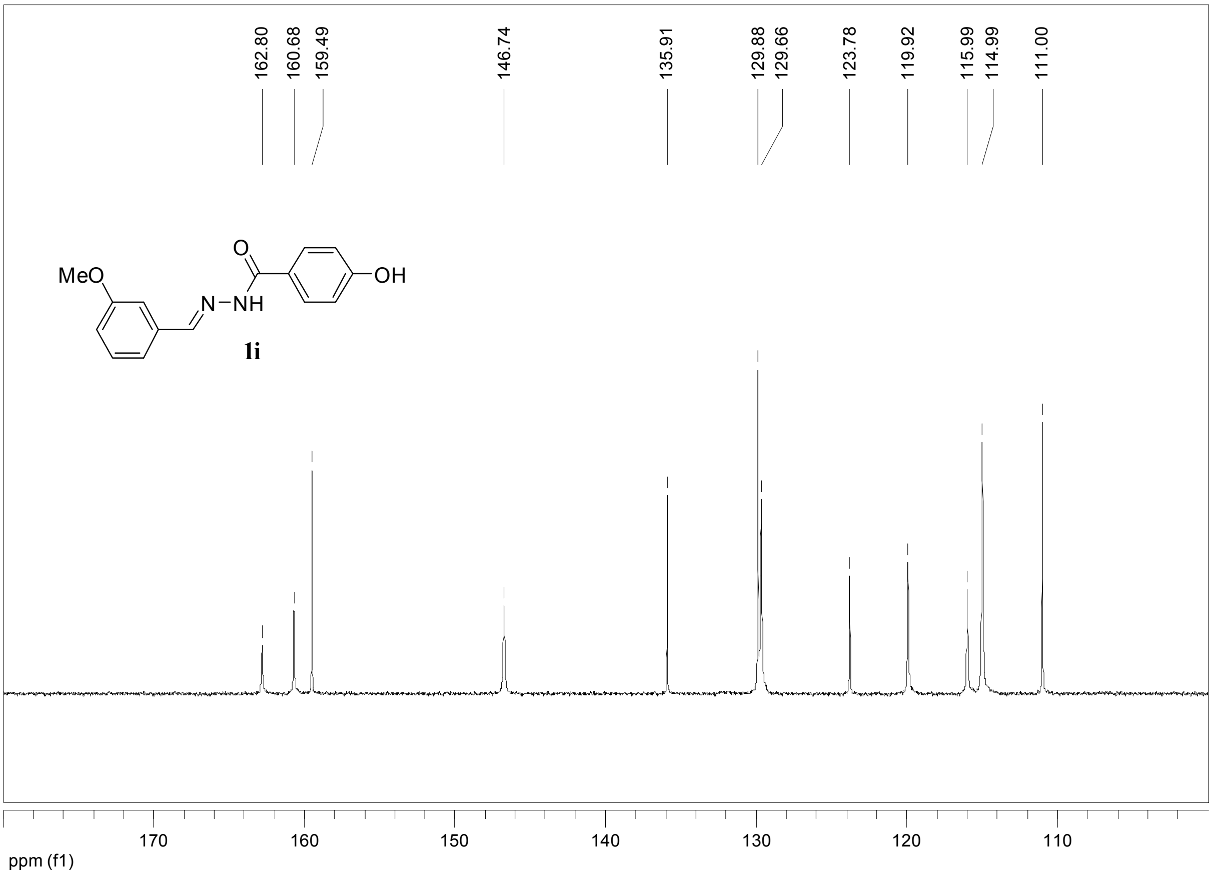

Figure S86. Expansion of ^{13}C-NMR (100 MHz, DMSO-d_6) spectrum of compound **1i**.

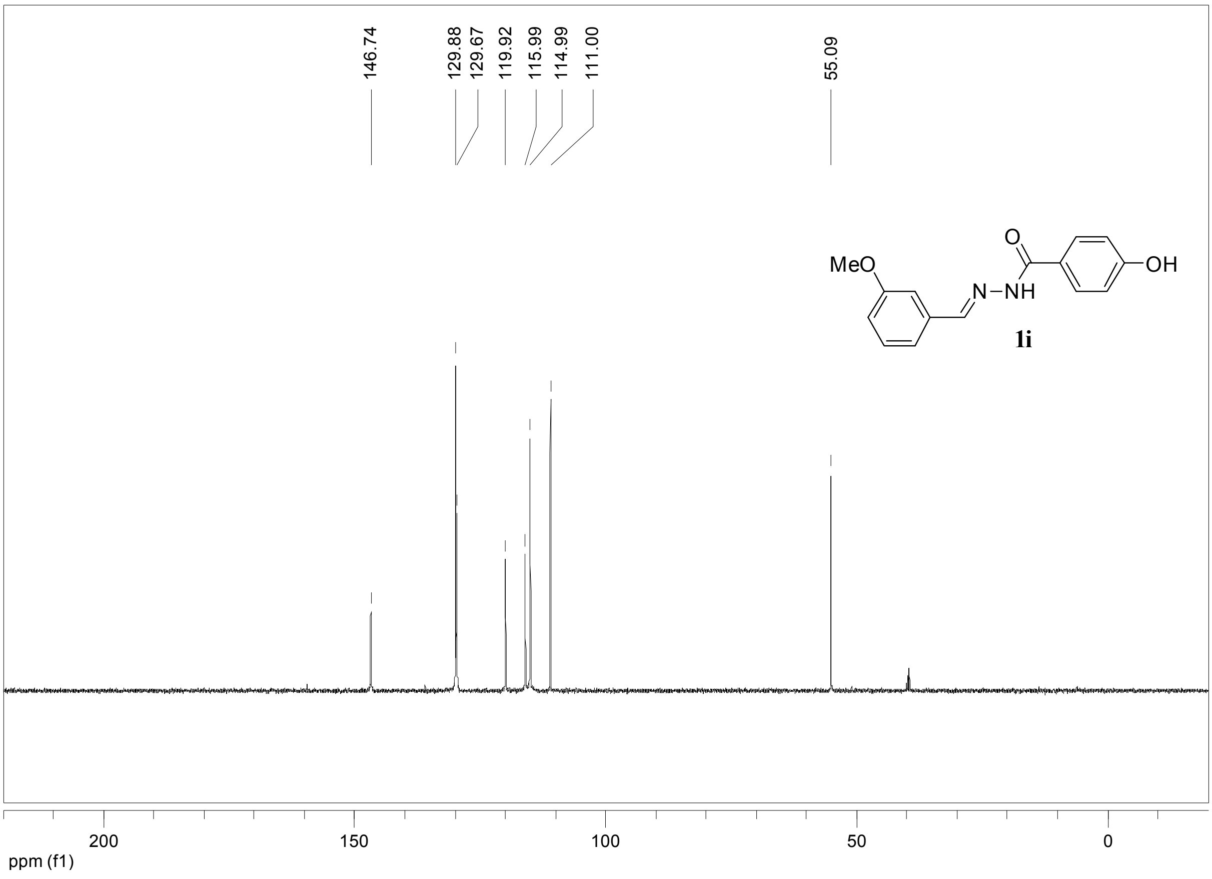

Figure S87. ^{13}C-NMR (100 MHz, DMSO-d_6) dept-135 experiment of compound **1i**.

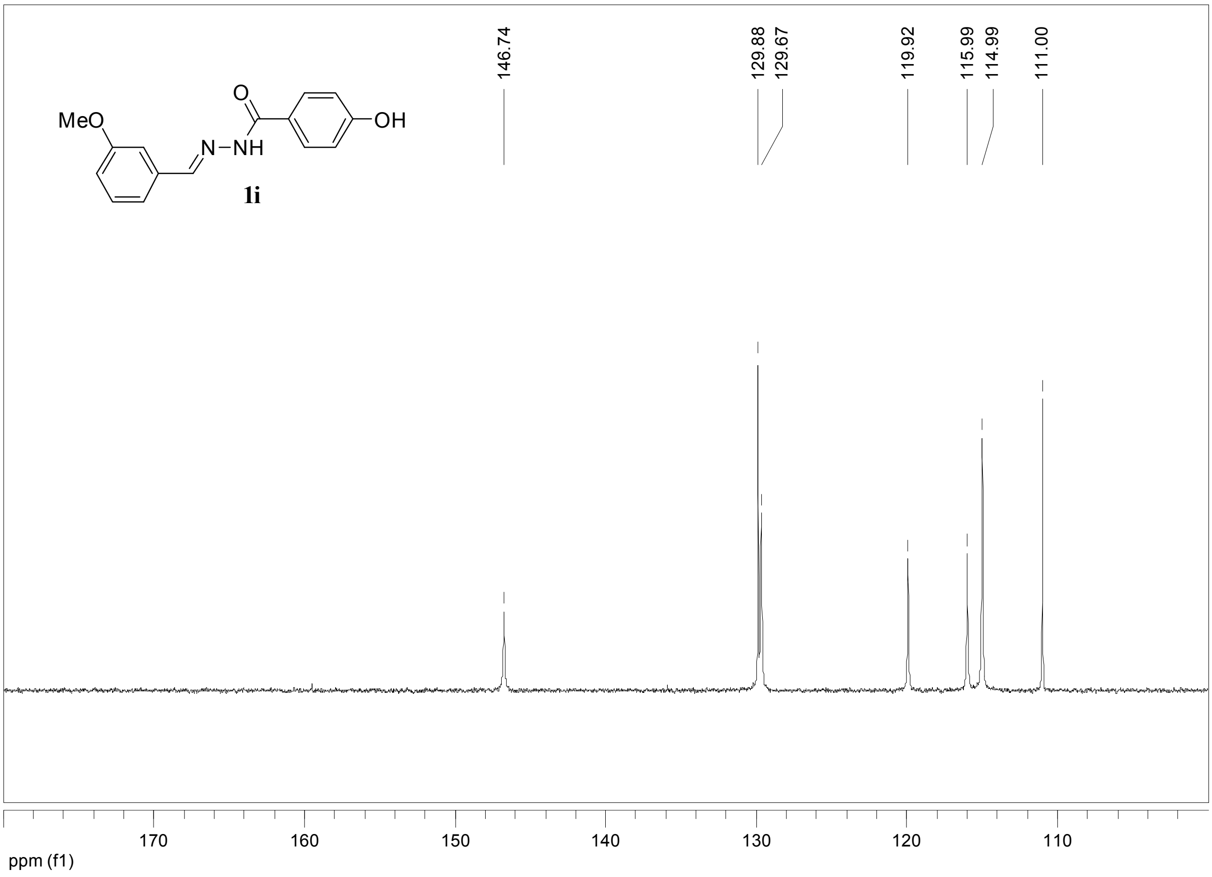

Figure S88. Expansion of ^{13}C-NMR (100 MHz, DMSO-d_6) dept-135 experiment of compound **1i**.

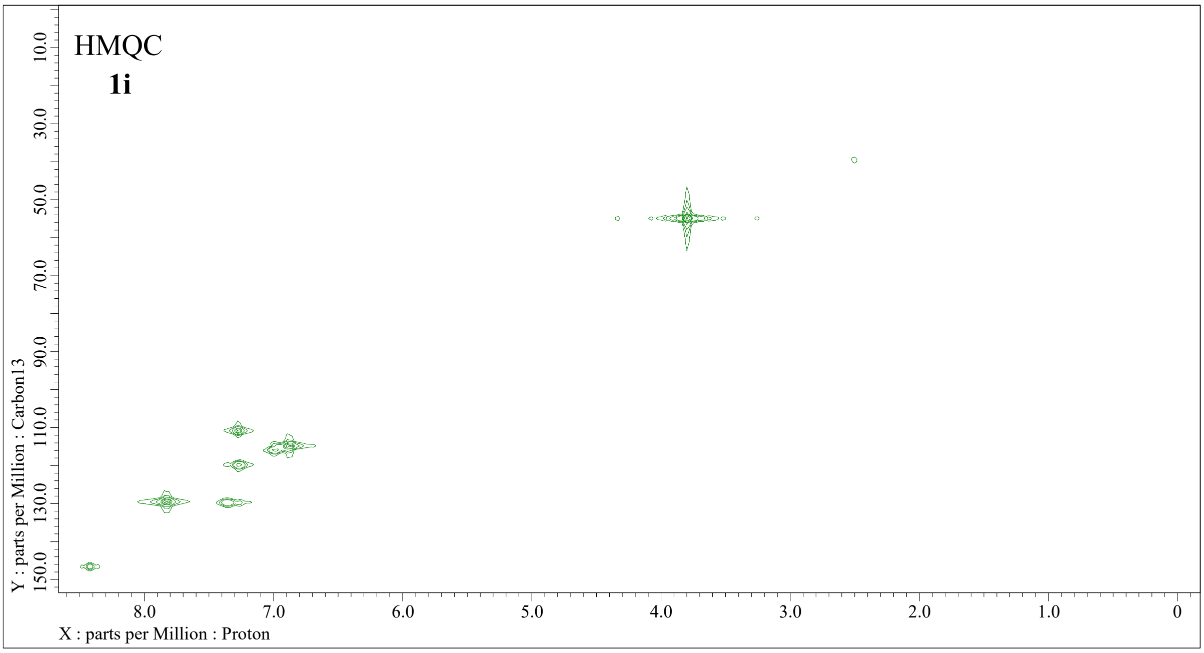

Figure S89. 2D-NMR (400 MHz, DMSO-d_6) HMQC experiment of 4-hydroxy-*N*'-[(*E*)-(3-methoxyphenyl)methylidene]benzohydrazide (**1i**).

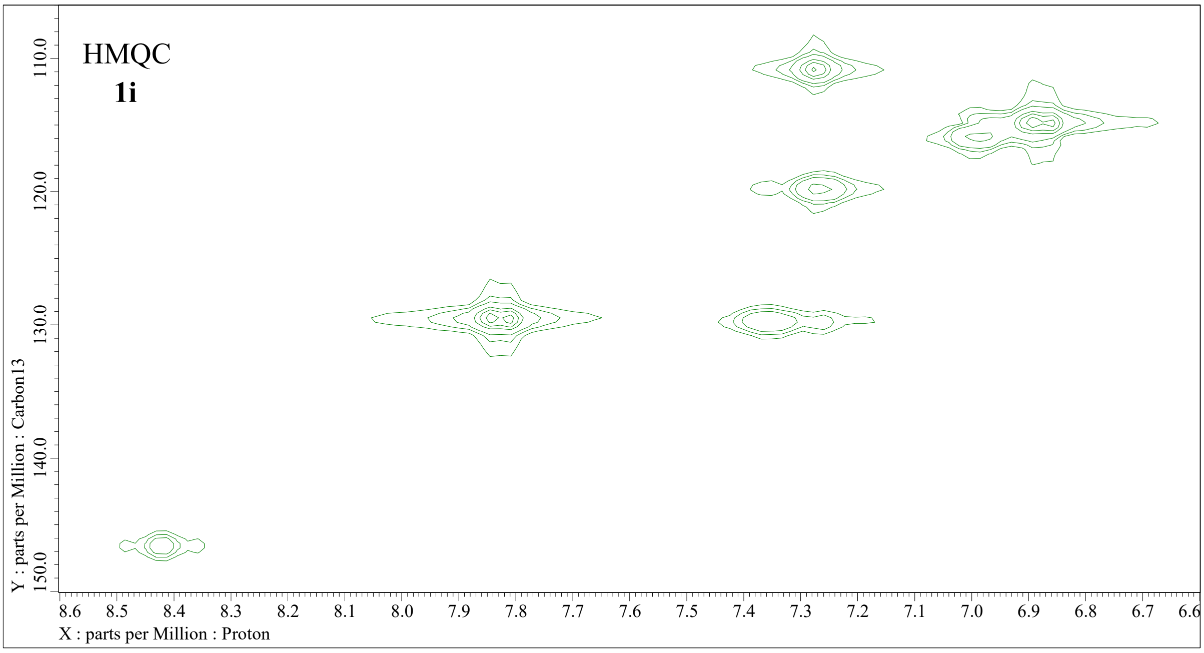

Figure S90. Expansion of 2D-NMR (400 MHz, DMSO-d_6) HMQC experiment of 4-hydroxy-*N*'-[(*E*)-(3-methoxyphenyl)methylidene]benzohydrazide (**1i**).

Molecules **2020**, *25*, 1255; doi:10.3390/molecules25051255 www.mdpi.com/journal/molecules

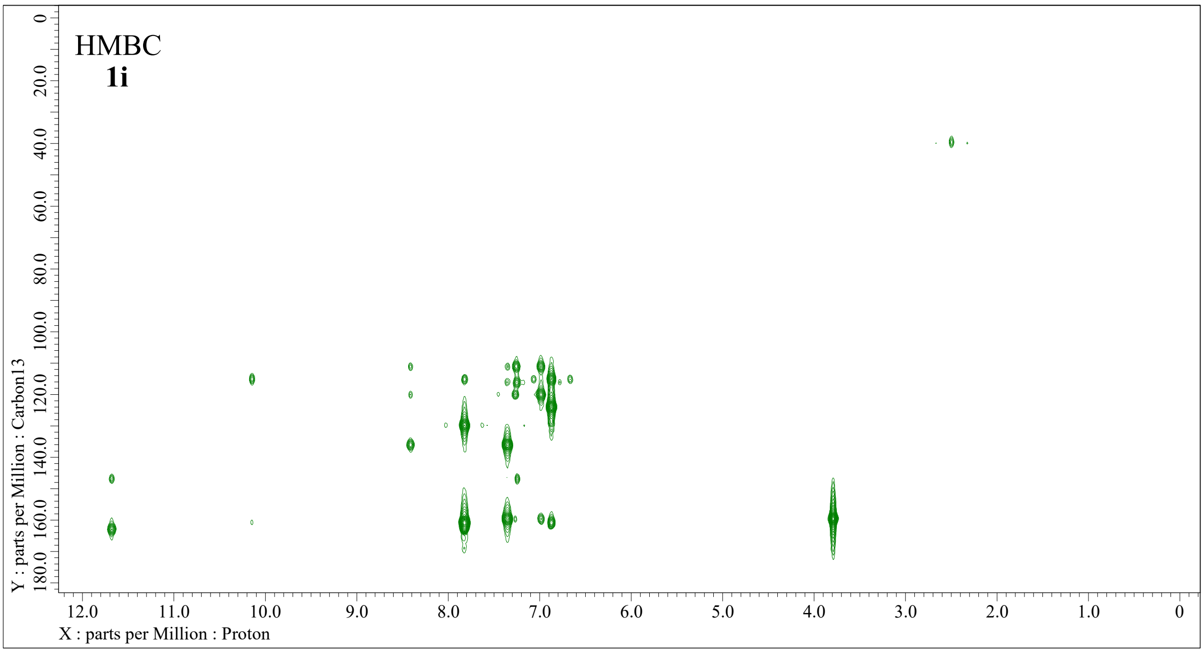

Figure S91. 2D-NMR (400 MHz, DMSO-d_6) HMBC experiment of 4-hydroxy-*N*'-[(*E*)-(3-methoxyphenyl)methylidene]benzohydrazide (**1i**).

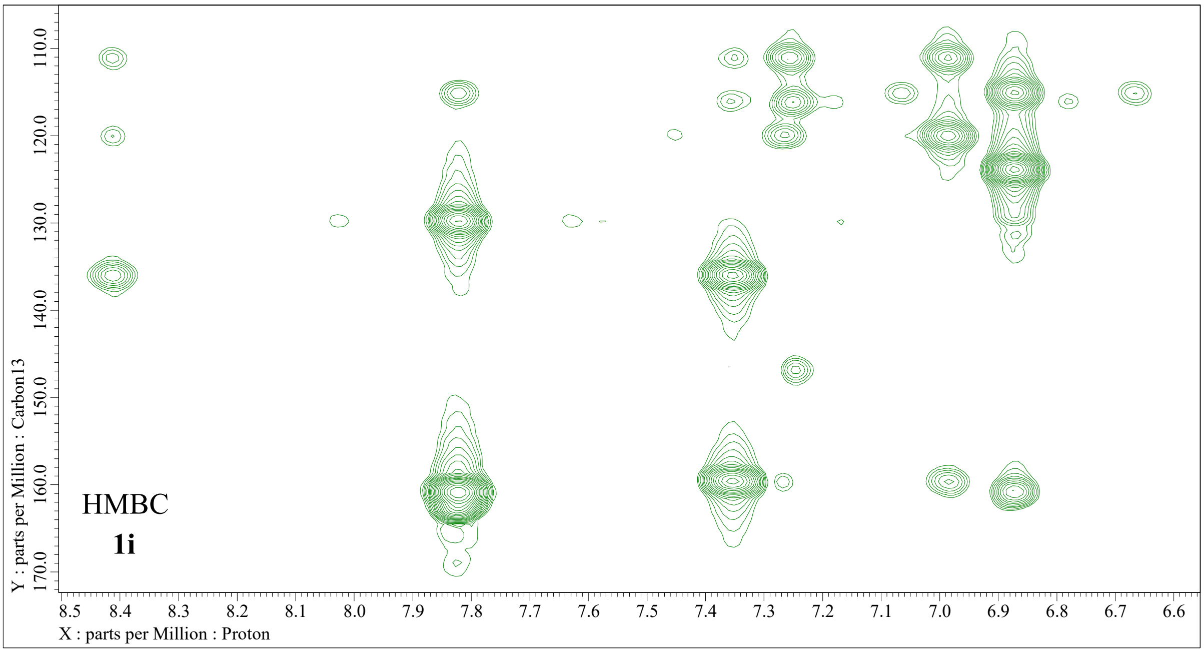

Figure S92. Expansion of 2D-NMR (400 MHz, DMSO-d_6) HMBC experiment of 4-hydroxy-*N*'-[(*E*)-(3-methoxyphenyl)methylidene]benzohydrazide (**1i**).

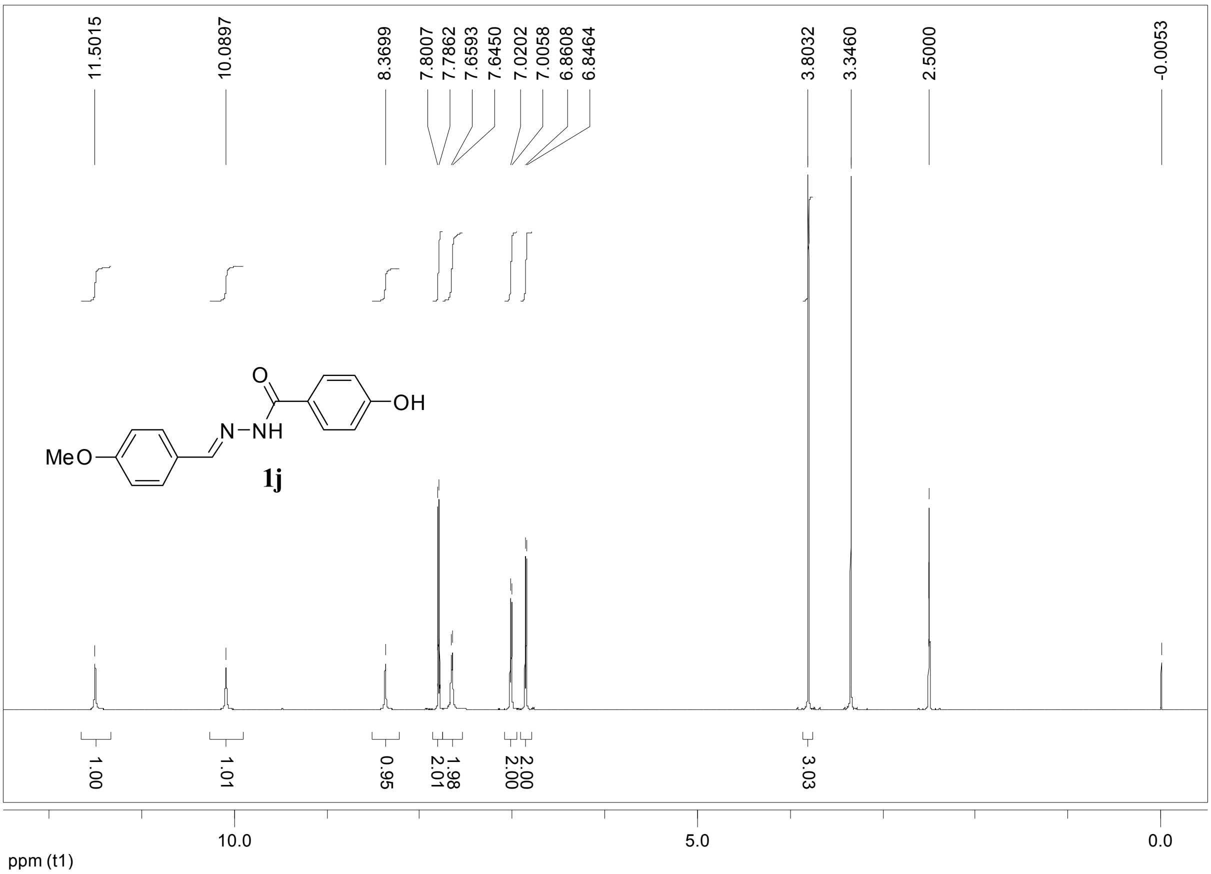

Figure S93. ¹H-NMR (600 MHz, DMSO-d_6) spectrum of compound **1j**.

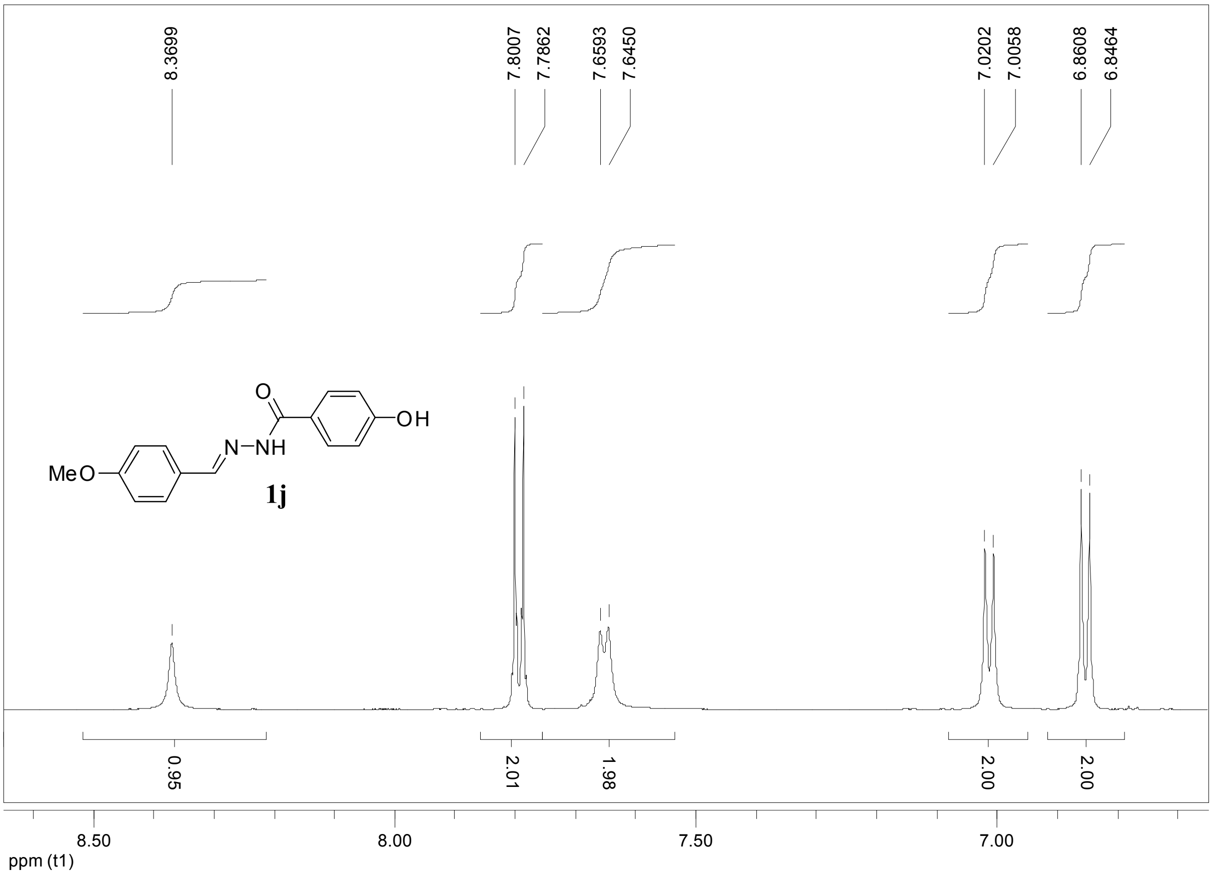

Figure S94. Expansion of ¹H-NMR (600 MHz, DMSO-d_6) spectrum of compound **1j**.

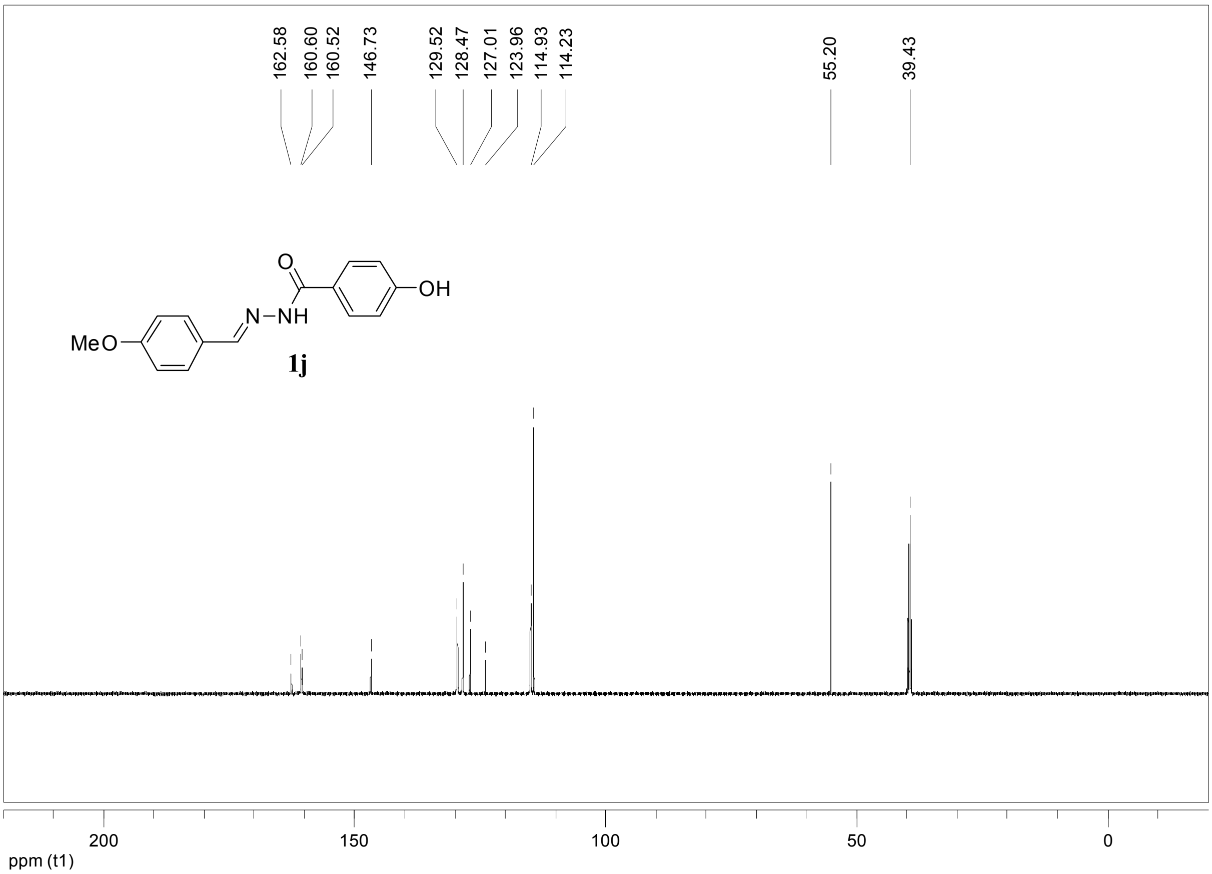

Figure S95. ^{13}C-NMR (150 MHz, DMSO-d_6) spectrum of compound **1j**.

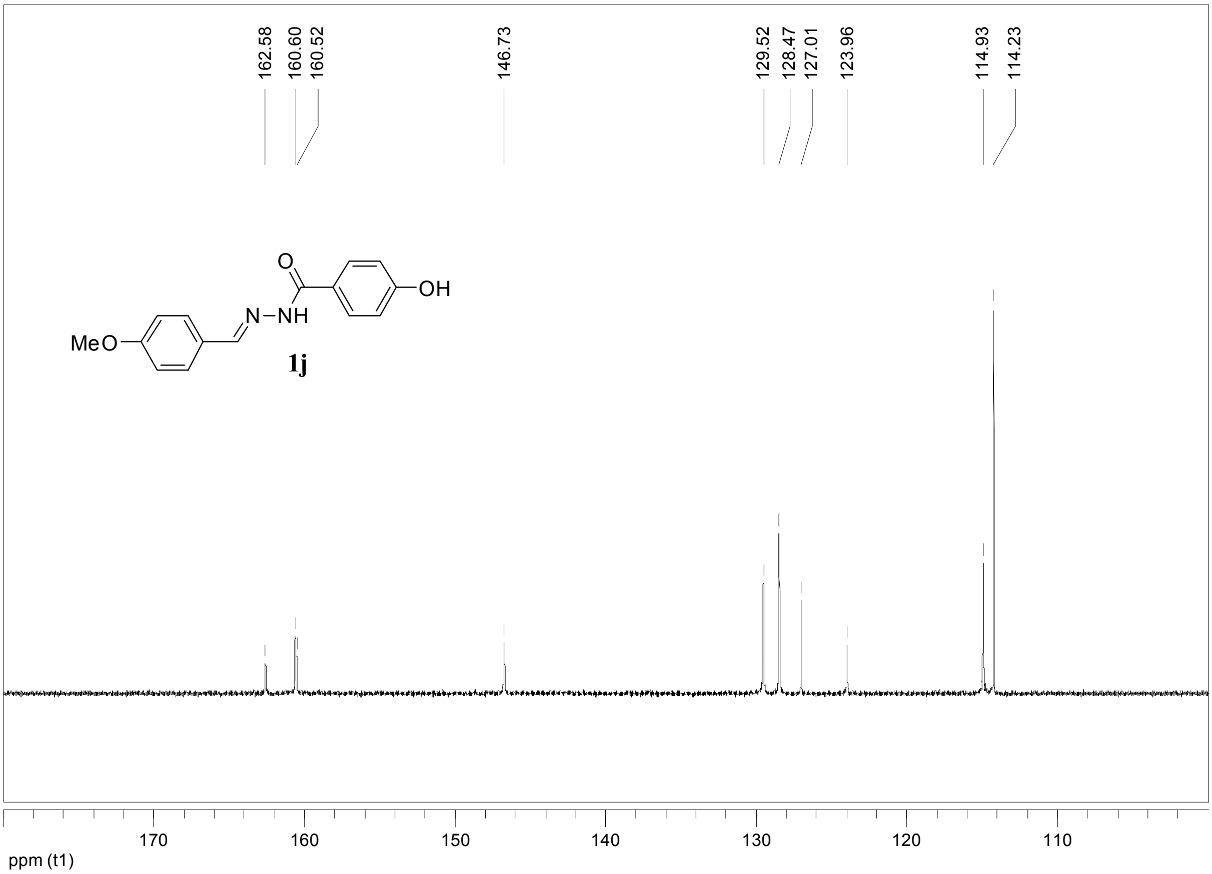

Figure S96. Expansion of ^{13}C-NMR (150 MHz, DMSO-d_6) spectrum of compound **1j**.

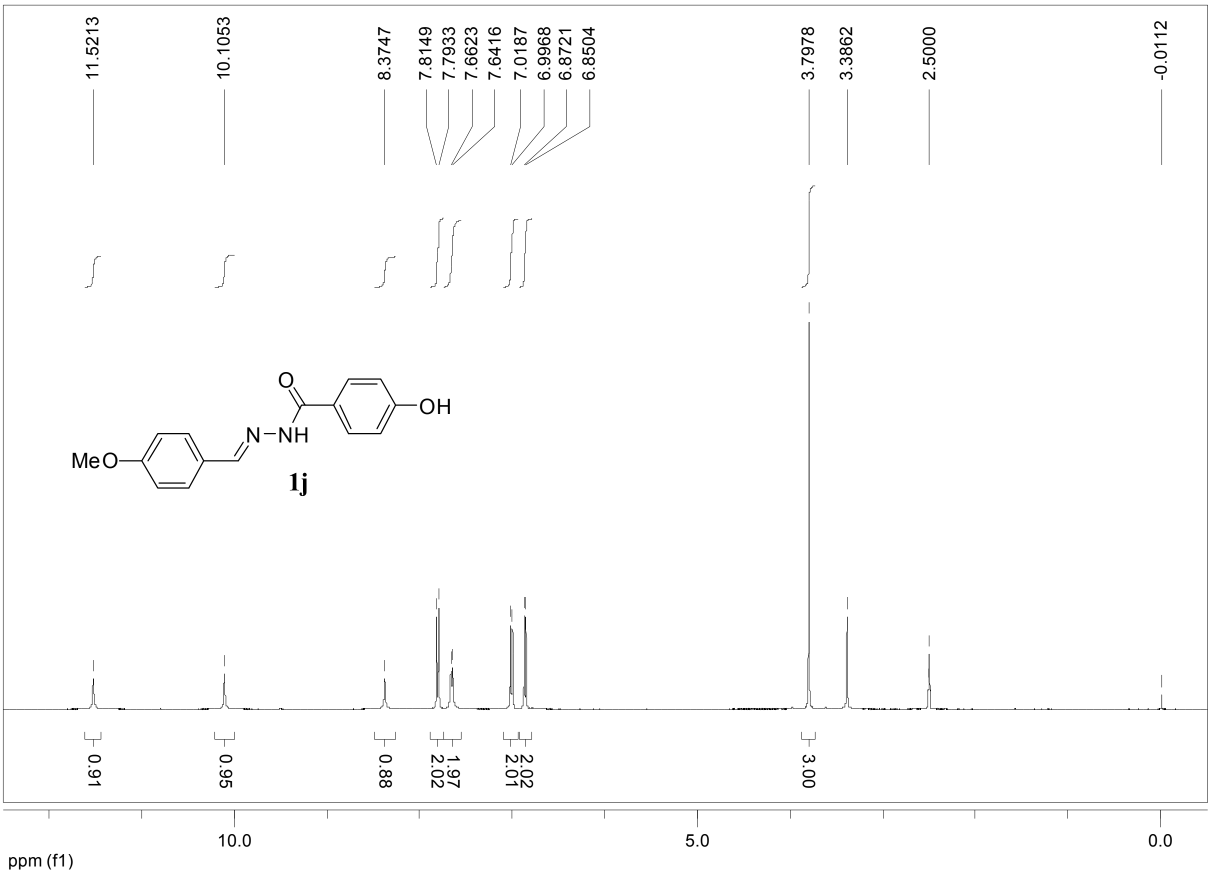

Figure S97. ^{1}H-NMR (400 MHz, DMSO-d_6) spectrum of compound **1j**.

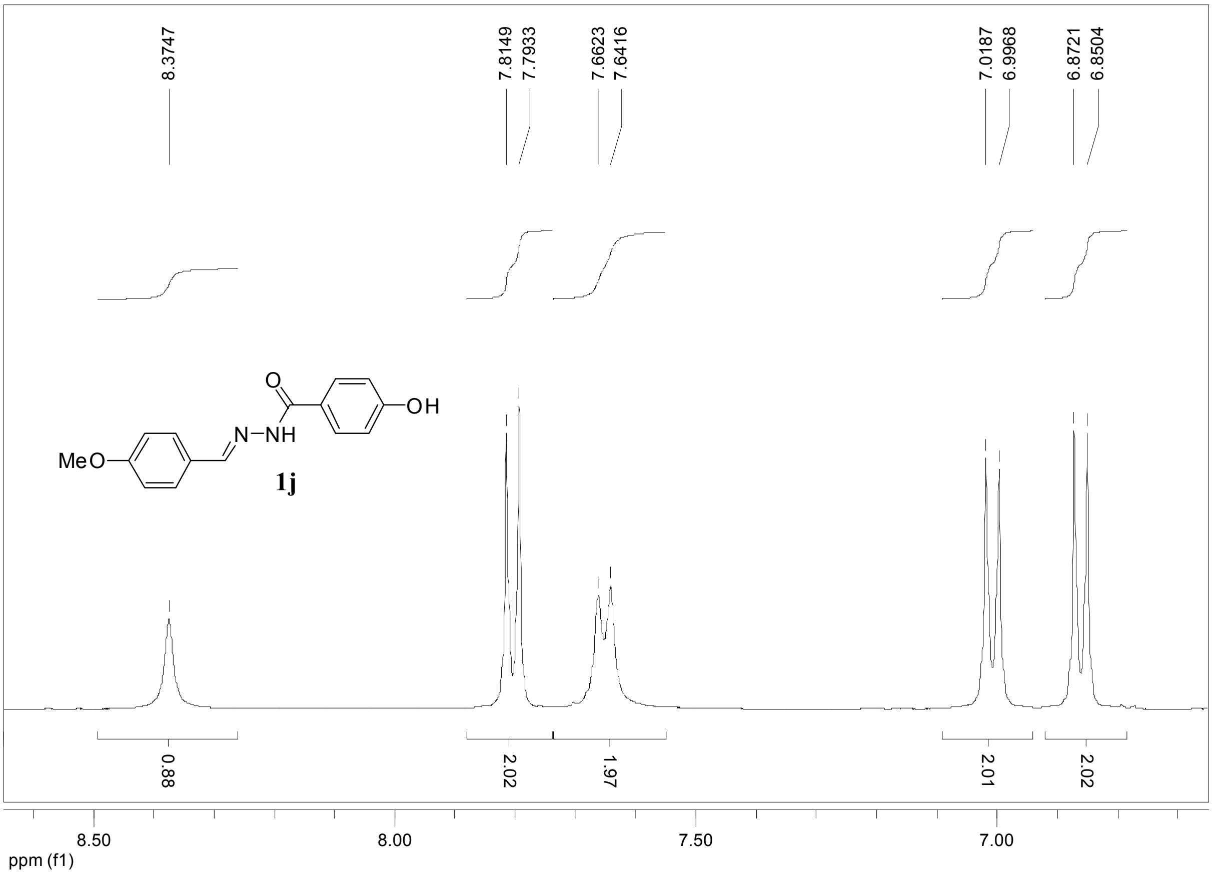

Figure S98. Expansion of ^{1}H-NMR (400 MHz, DMSO-d_6) spectrum of compound **1j**.

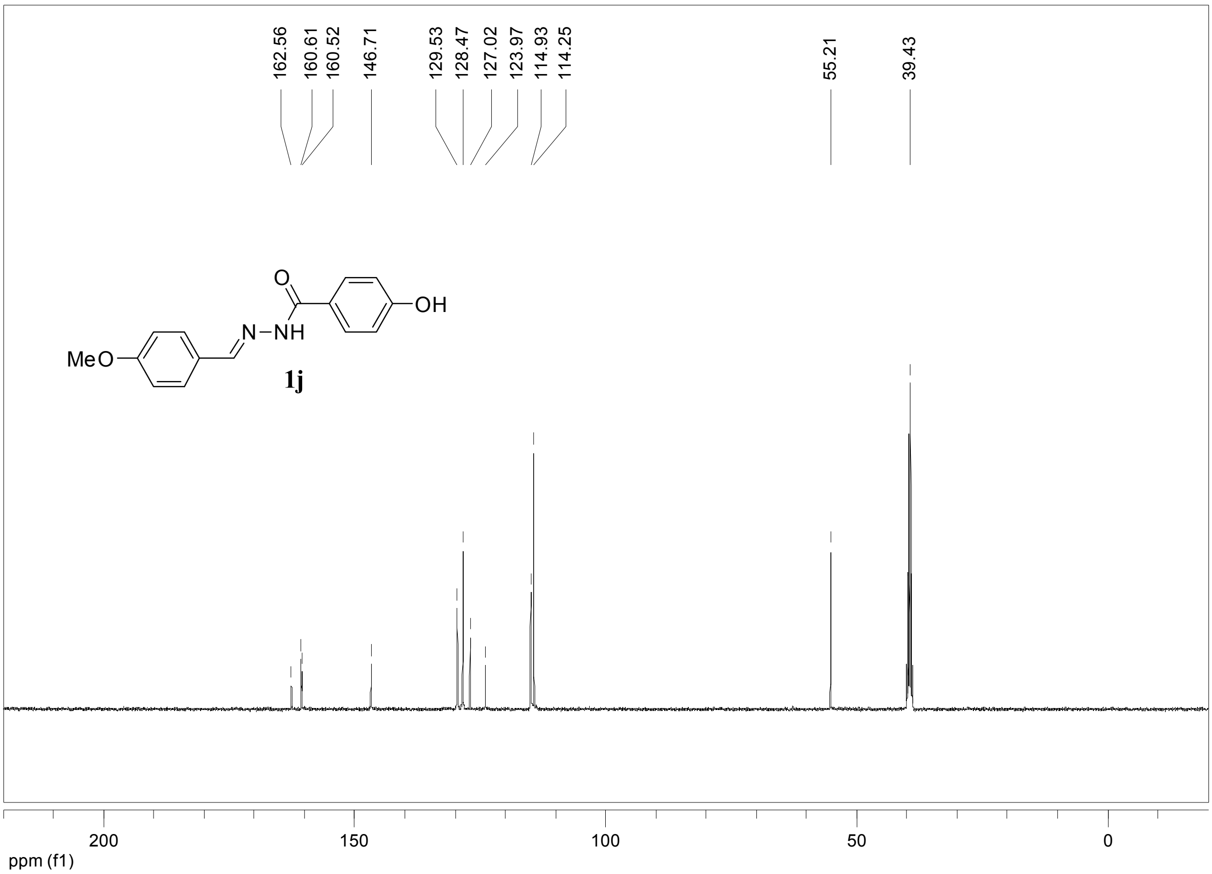

Figure S99. ^{13}C-NMR (100 MHz, DMSO-d_6) spectrum of compound **1j**.

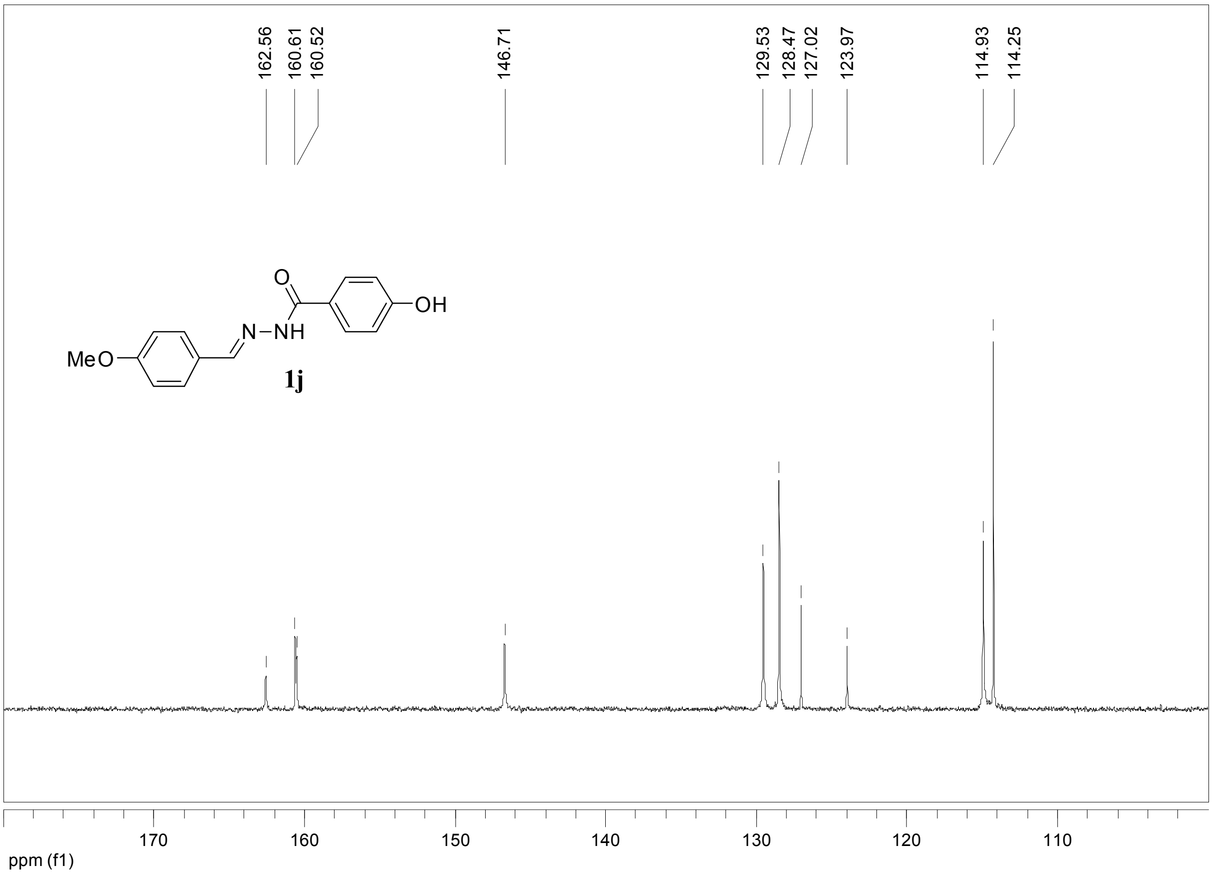

Figure S100. Expansion of ^{13}C-NMR (100 MHz, DMSO-d_6) spectrum of compound **1j**.

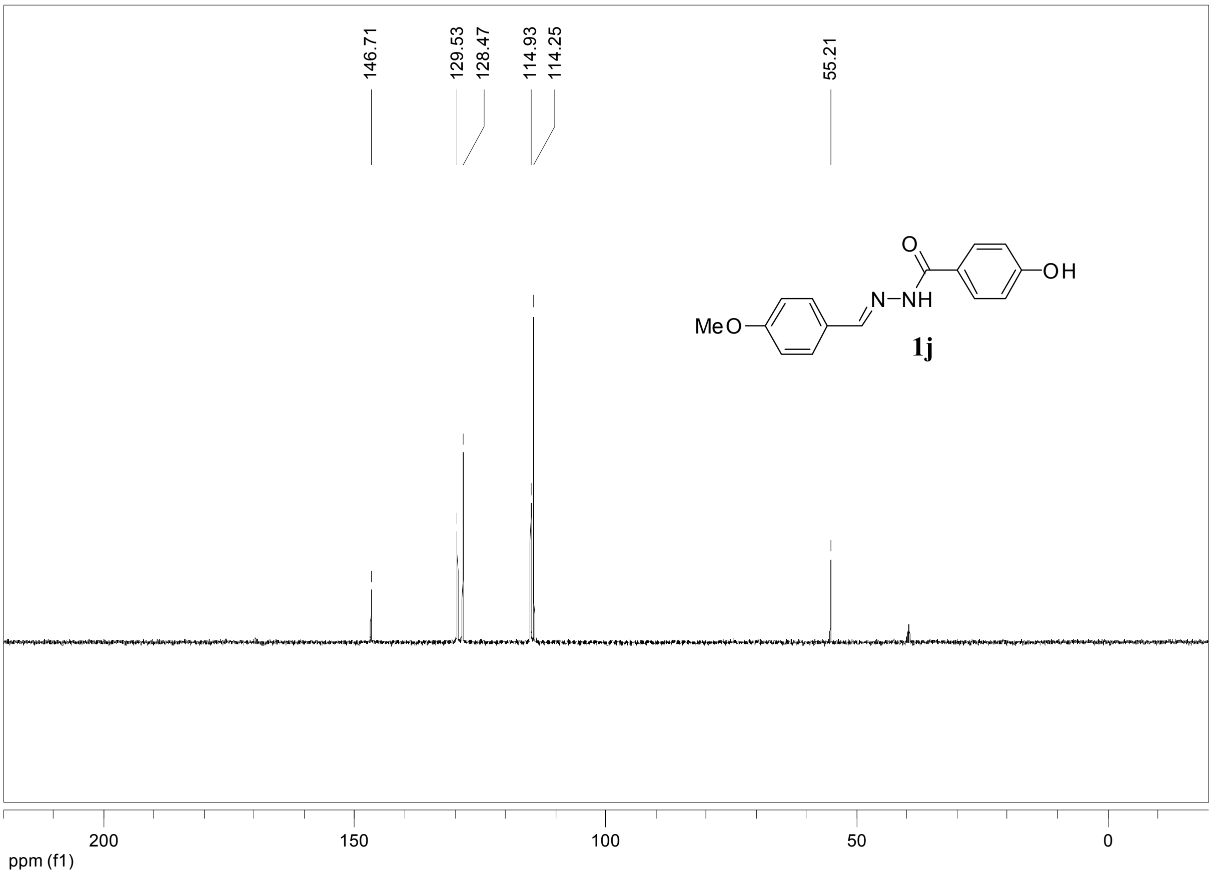

Figure S101. ¹³C-NMR (100 MHz, DMSO-d_6) dept-135 experiment of compound **1j**.

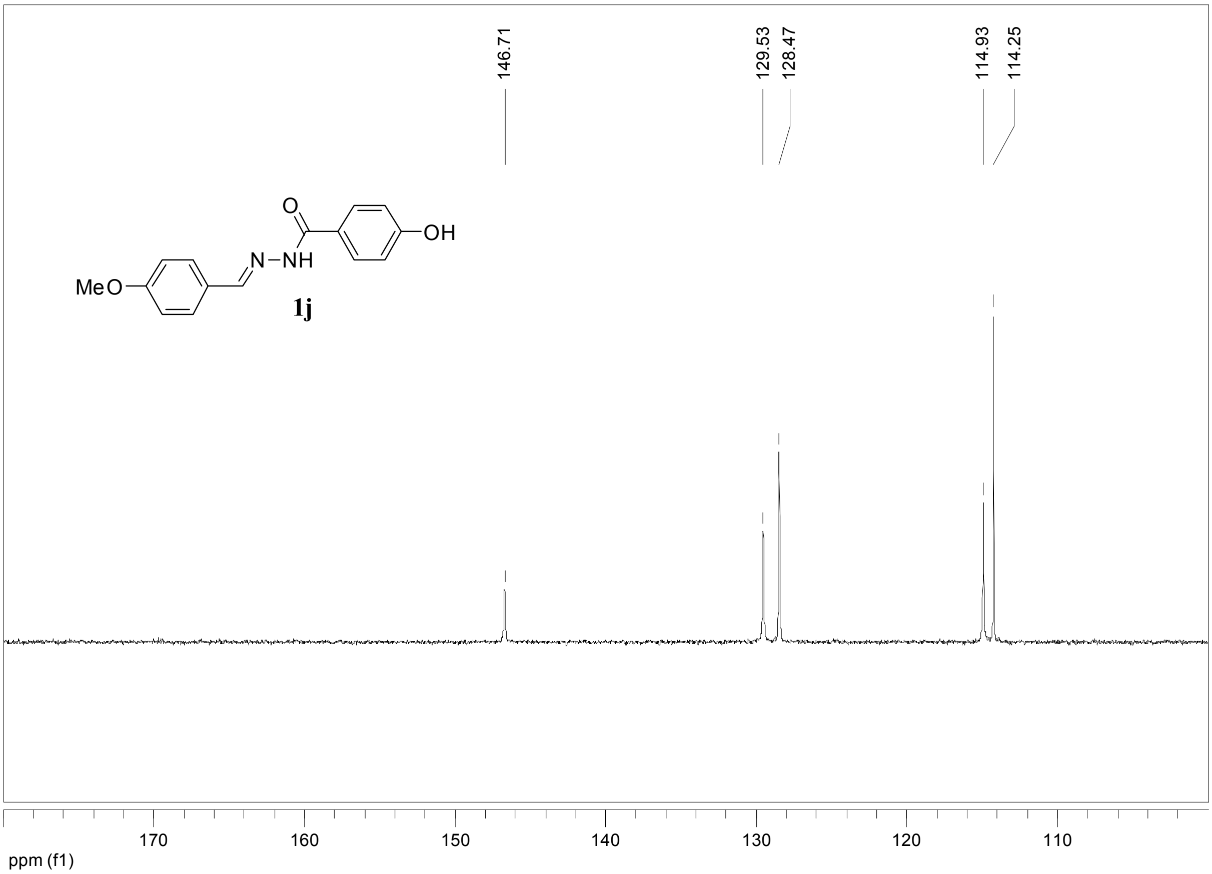

Figure S102. Expansion of ¹³C-NMR (100 MHz, DMSO-d_6) dept-135 experiment of compound **1j**.

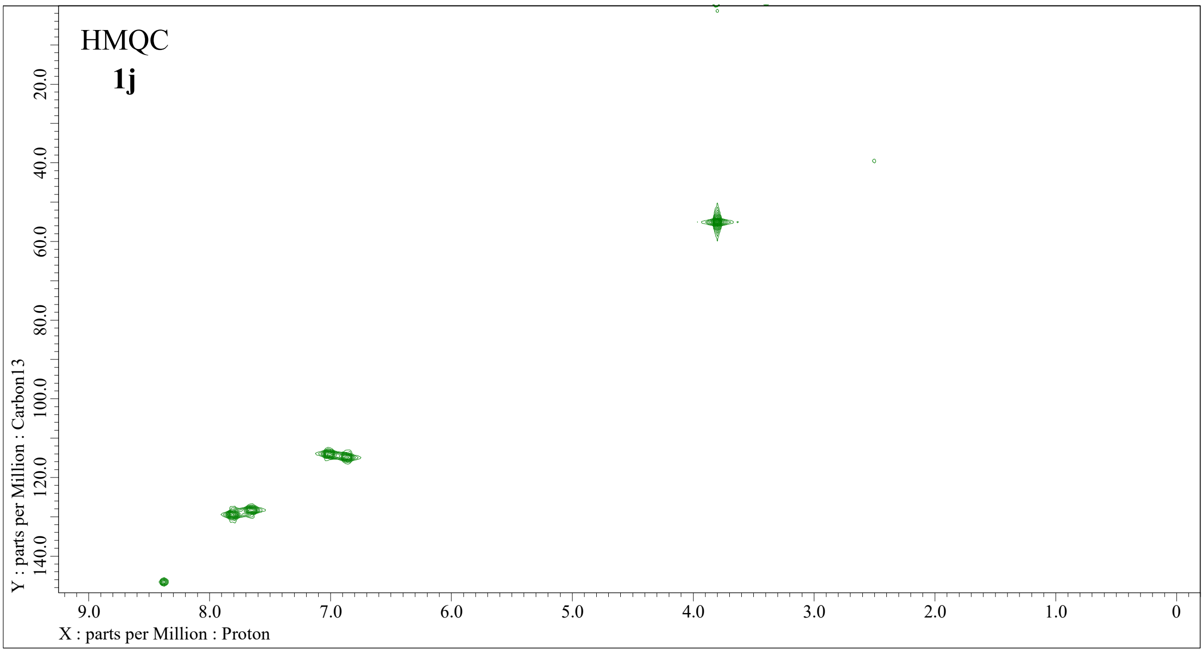

Figure S103. 2D-NMR (400 MHz, DMSO-d_6) HMQC experiment of 4-hydroxy-*N*'-[(*E*)-(4-methoxyphenyl)methylidene]benzohydrazide (**1j**).

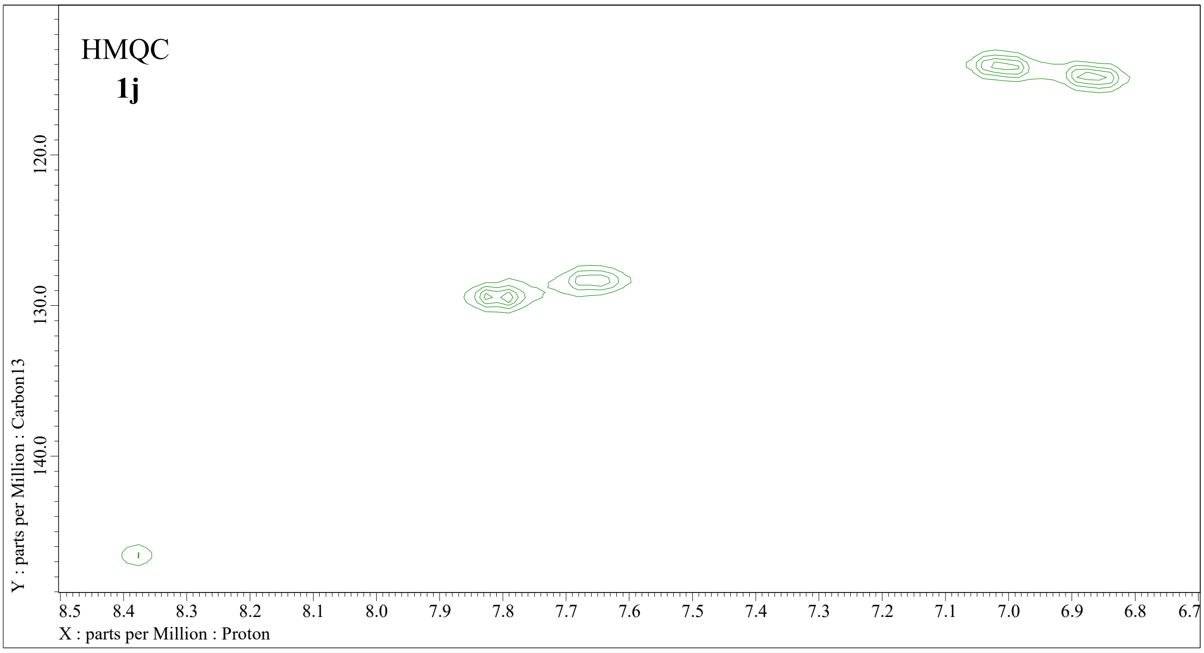

Figure S104. Expansion of 2D-NMR (400 MHz, DMSO-d_6) HMQC experiment of 4-hydroxy-*N*'-[(*E*)-(4-methoxyphenyl)methylidene]benzohydrazide (**1j**).

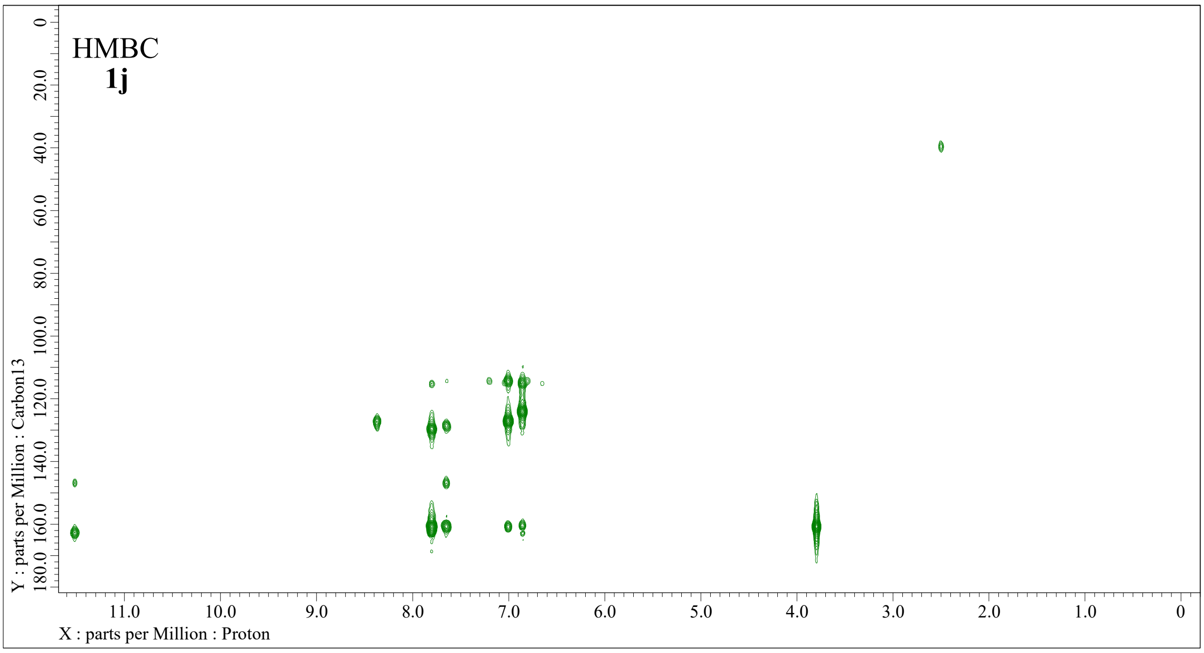

Figure S105. 2D-NMR (400 MHz, DMSO-d_6) HMBC experiment of 4-hydroxy-*N*'-[(*E*)-(4-methoxyphenyl)methylidene]benzohydrazide (**1j**).

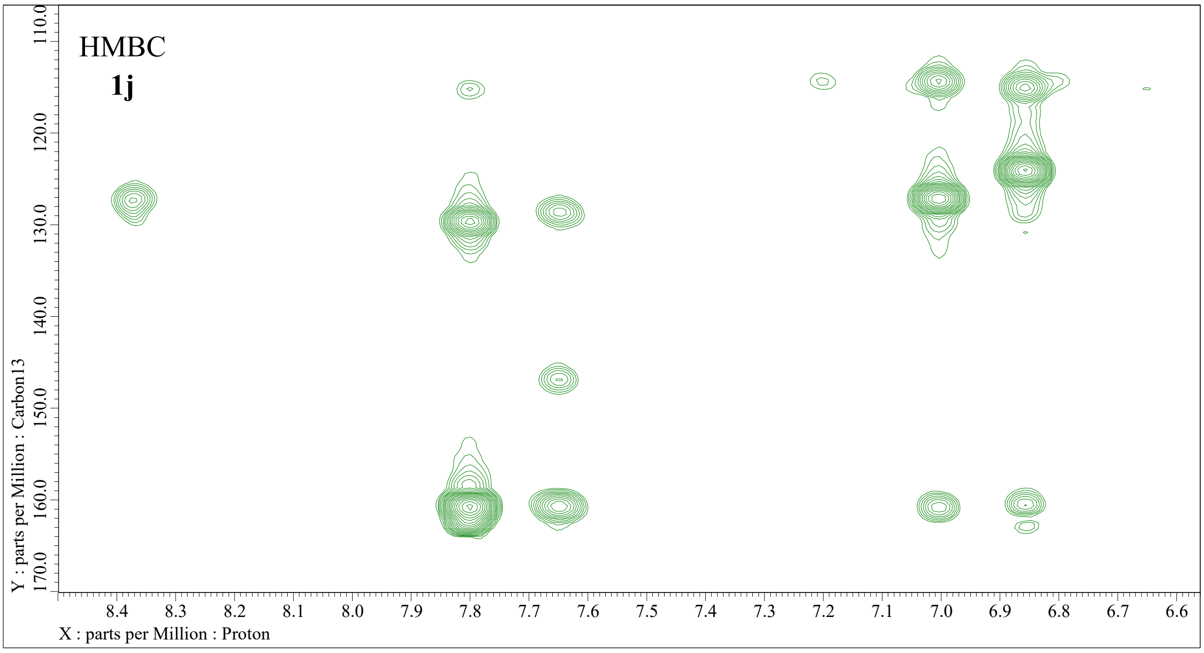

Figure S106. Expansion of 2D-NMR (400 MHz, DMSO-d_6) HMBC experiment of 4-hydroxy-*N*'-[(*E*)-(4-methoxyphenyl)methylidene]benzohydrazide (**1j**).

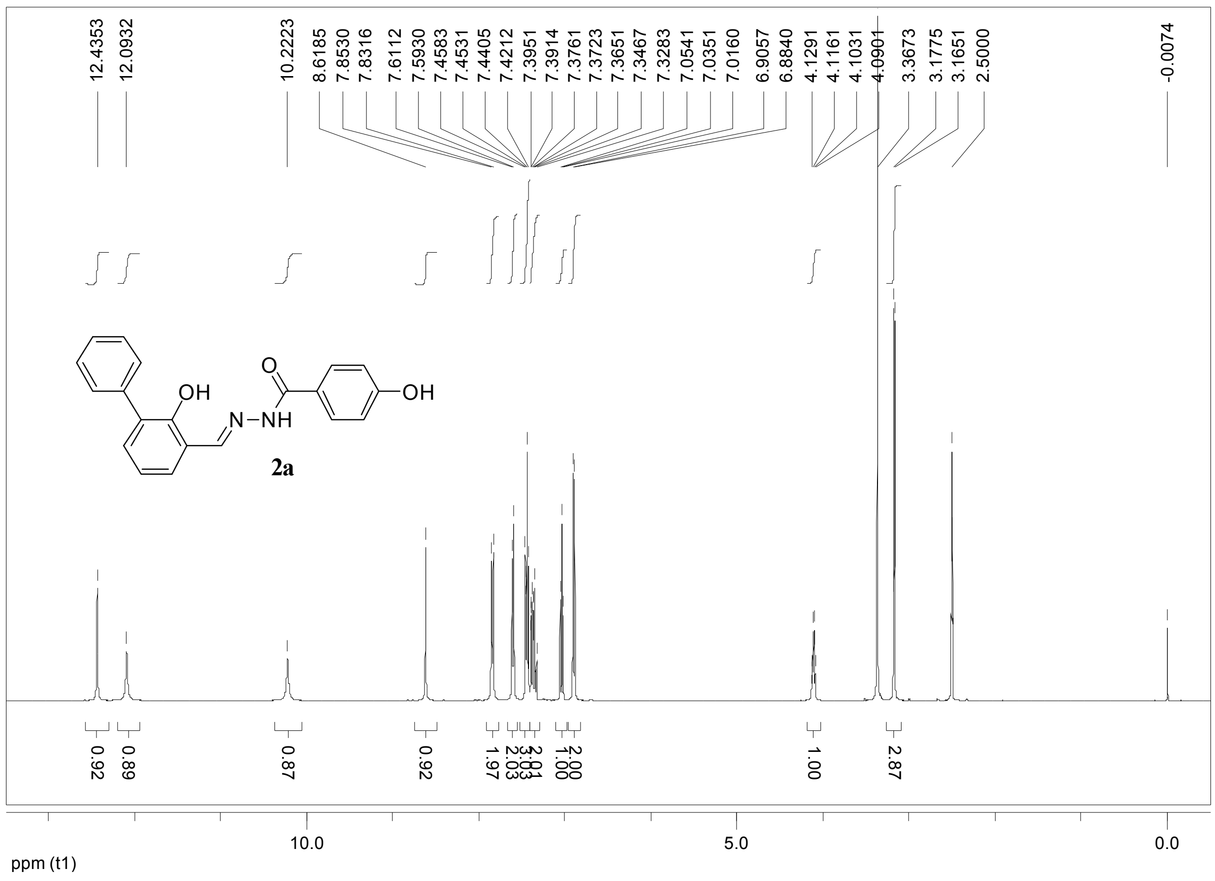

Figure S107. ¹H-NMR (400 MHz, DMSO-d_6) spectrum of compound **2a**.

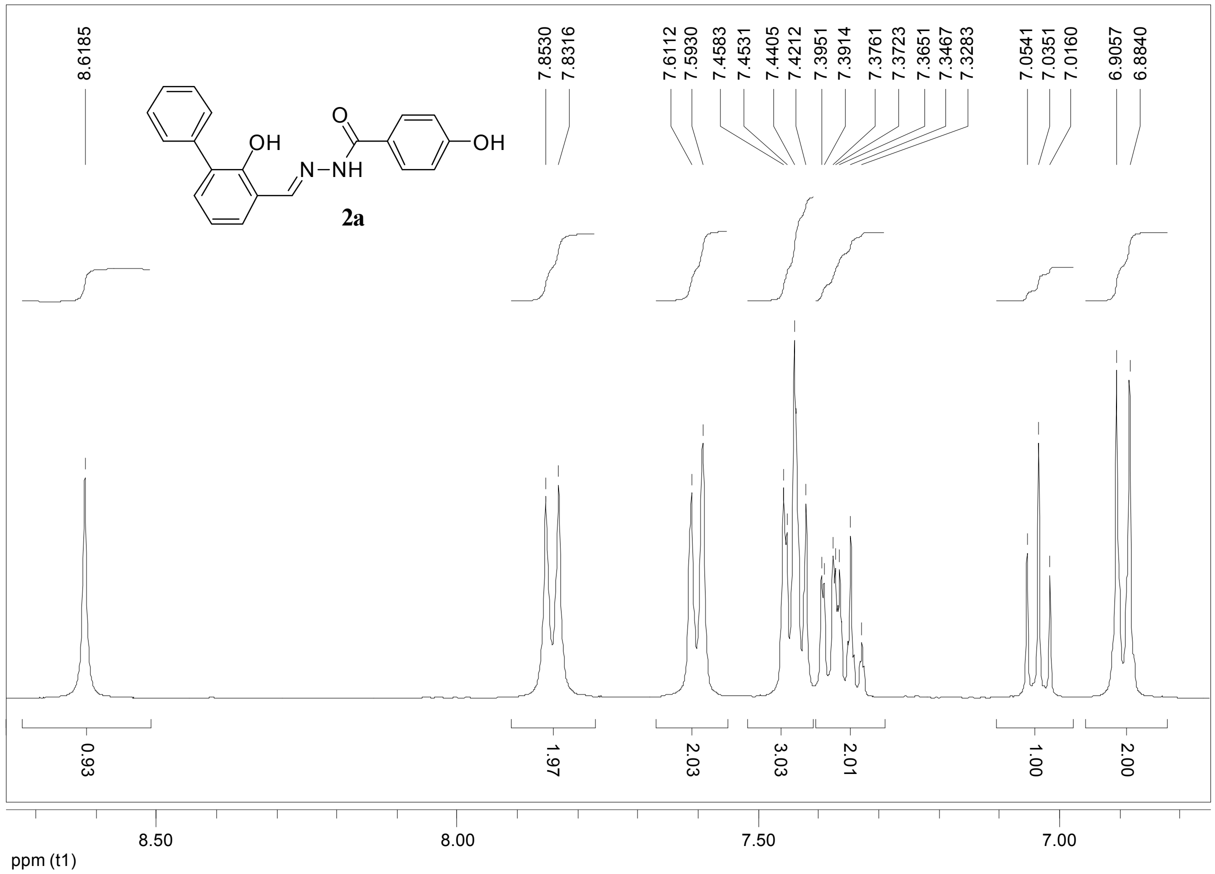

Figure S108. Expansion of ¹H-NMR (400 MHz, DMSO-d_6) spectrum of compound **2a**.

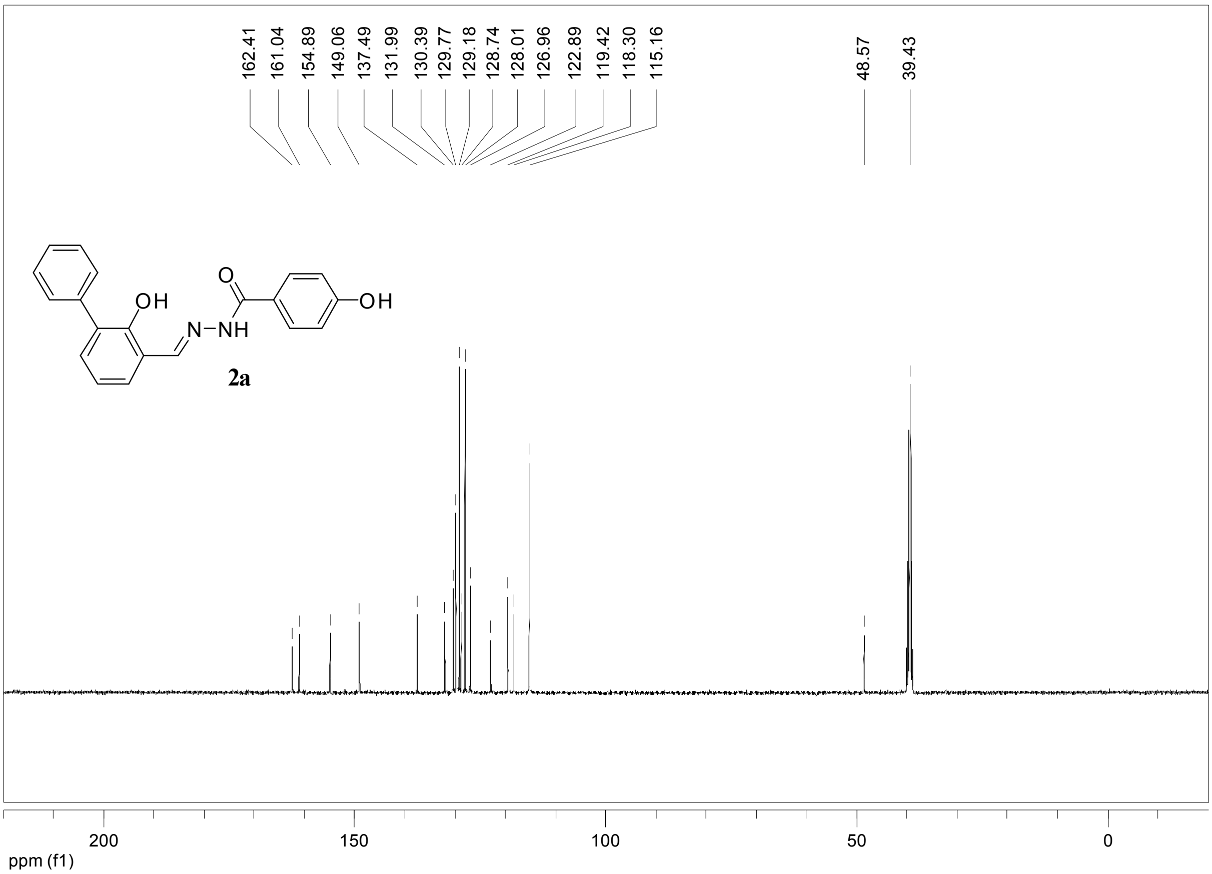

Figure S109. ^{13}C-NMR (100 MHz, DMSO-d_6) spectrum of compound **2a**.

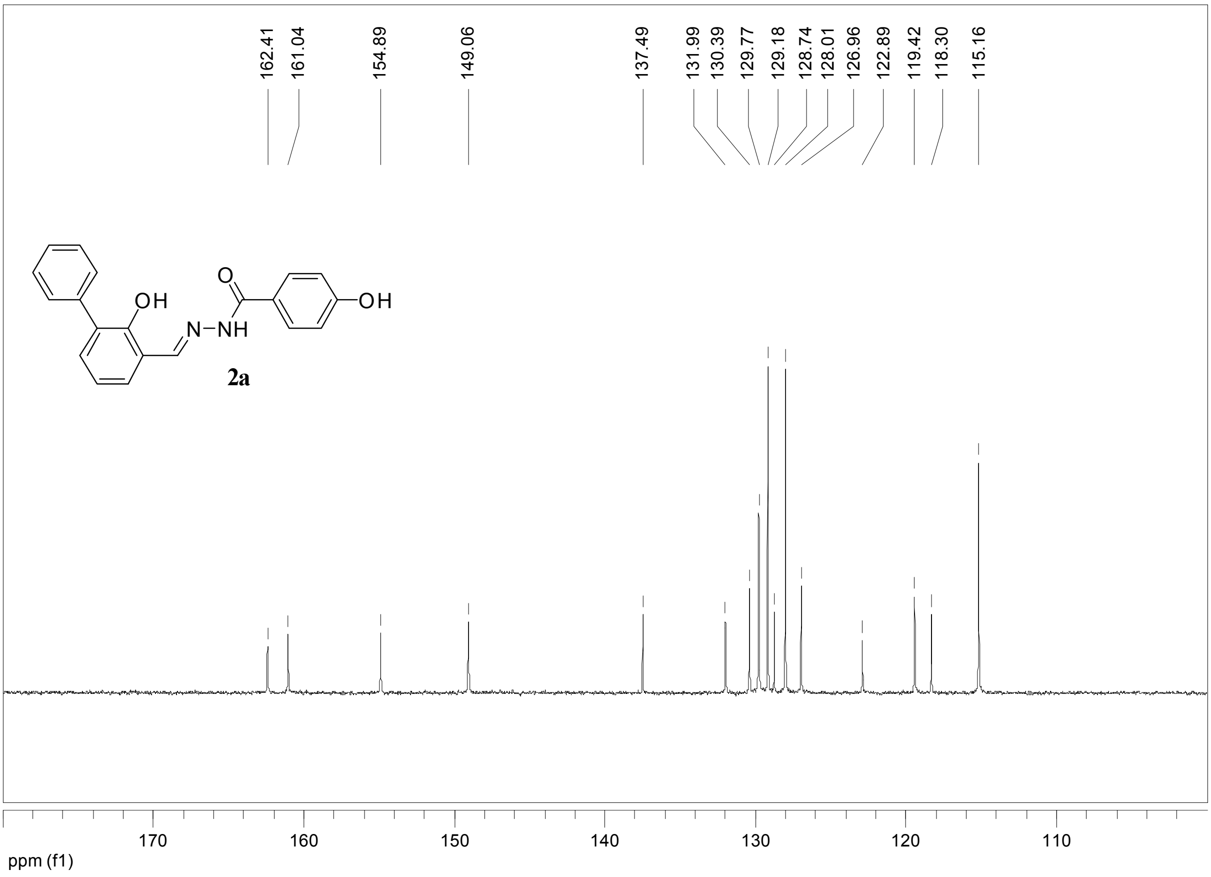

Figure S110. Expansion of ^{13}C-NMR (100 MHz, DMSO-d_6) spectrum of compound **2a**.

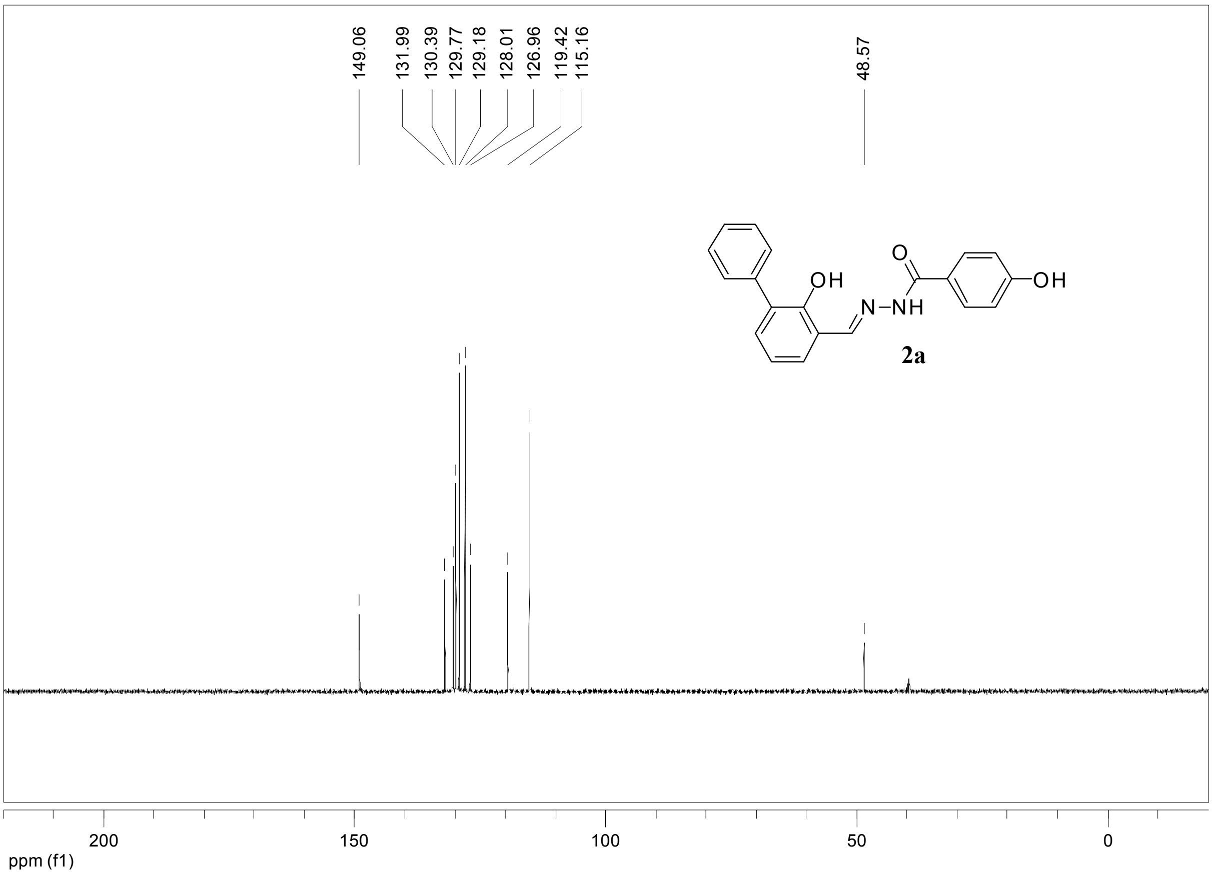

Figure S111. ^{13}C-NMR (100 MHz, DMSO-d_6) dept-135 experiment of compound **2a**.

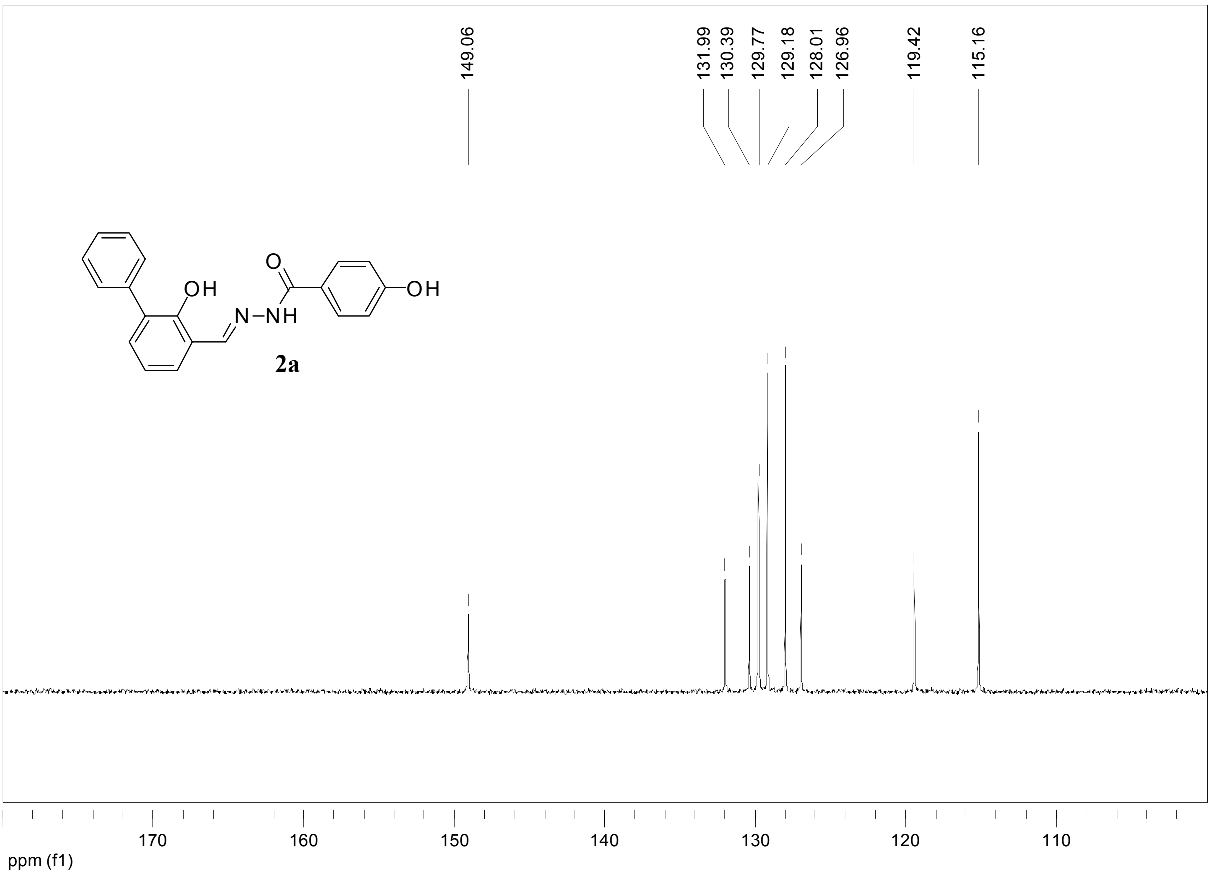

Figure S112. Expansion of ^{13}C-NMR (100 MHz, DMSO-d_6) dept-135 experiment of compound **2a**.

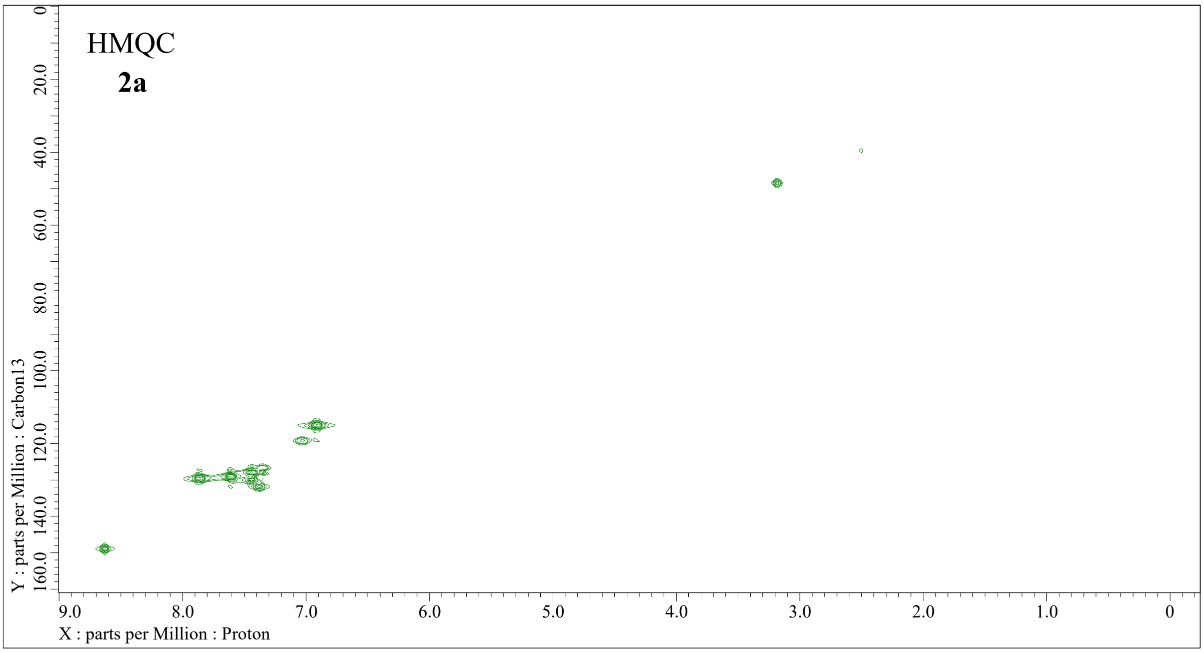

Figure S113. 2D-NMR (400 MHz, DMSO-d_6) HMQC experiment of 4-hydroxy-*N*'-[(*E*)-(2-hydroxy-3-phenyl-phenyl)methylidene]benzohydrazide (**2a**).

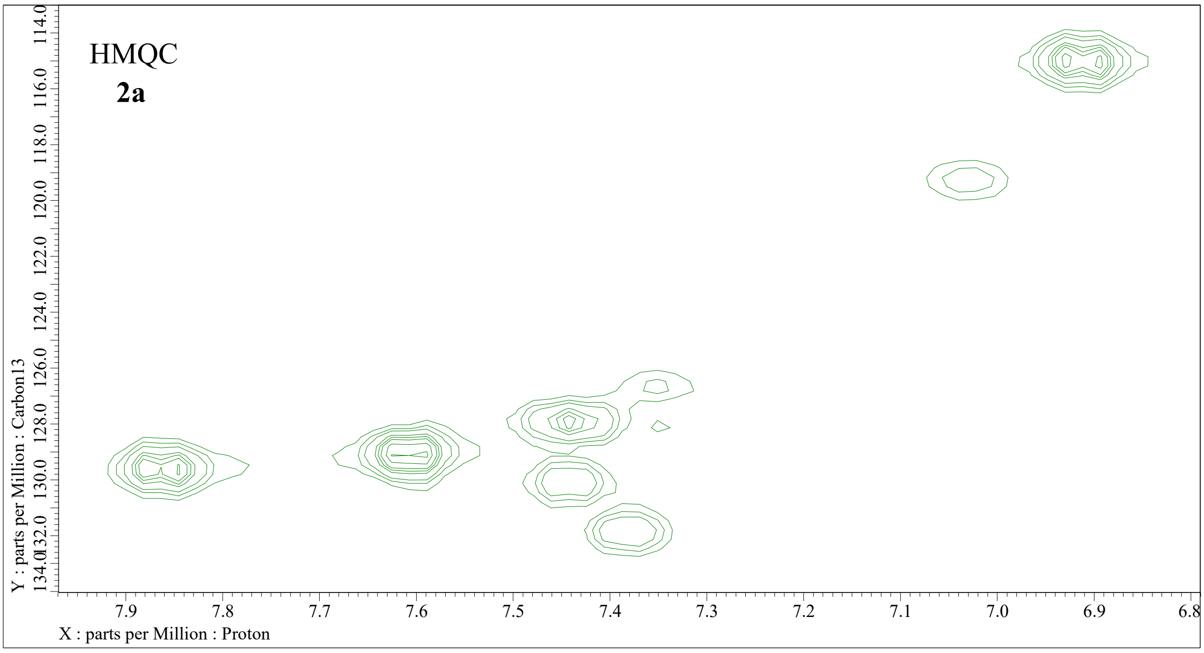

Figure S114. Expansion of 2D-NMR (400 MHz, DMSO-d_6) HMQC experiment of 4-hydroxy-*N*'-[(*E*)-(2-hydroxy-3-phenyl-phenyl)methylidene]benzohydrazide (**2a**).

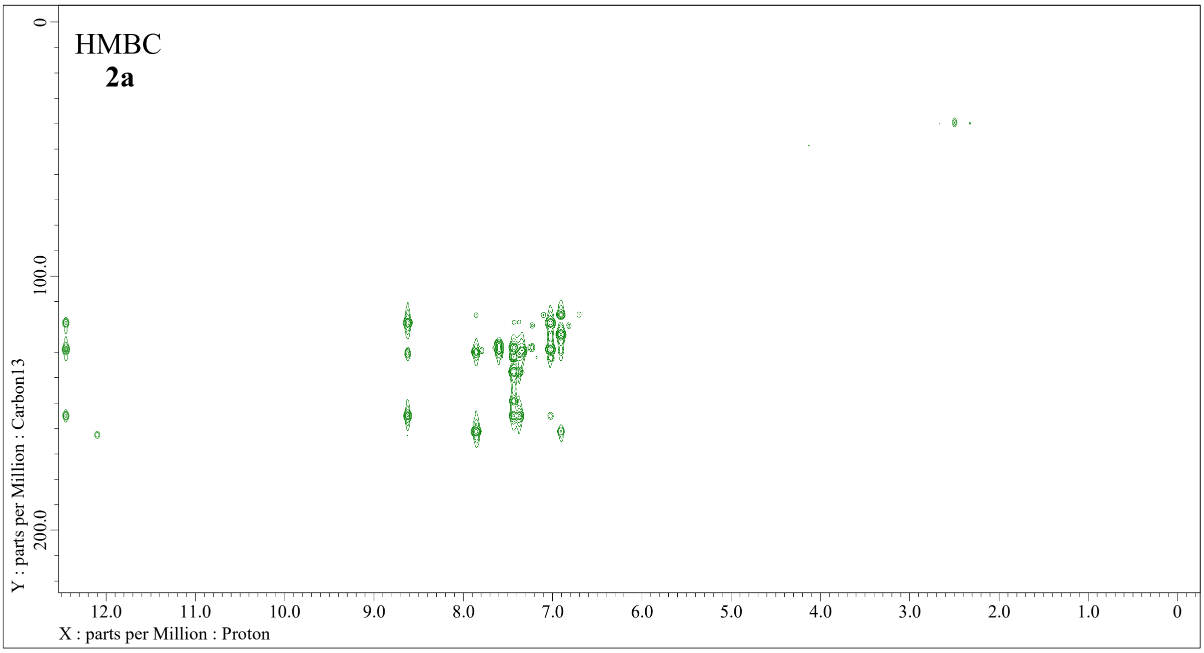

Figure S115. 2D-NMR (400 MHz, DMSO-d_6) HMBC experiment of 4-hydroxy-*N*'-[(*E*)-(2-hydroxy-3-phenyl-phenyl)methylidene]benzohydrazide (**2a**).

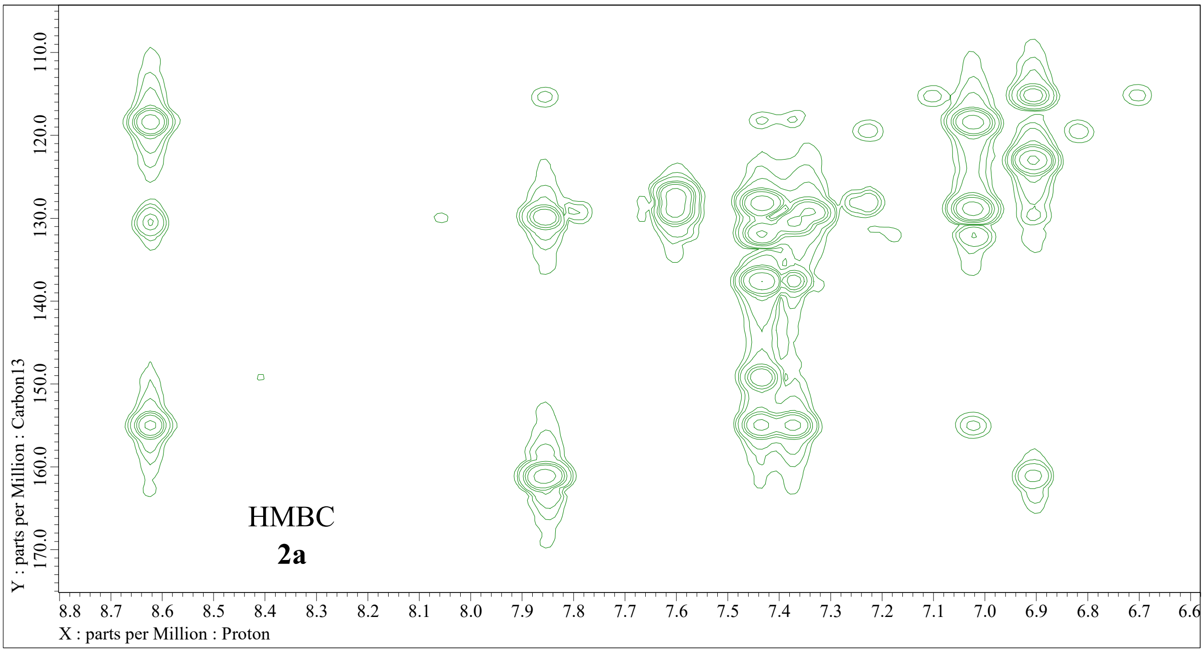

Figure S116. Expansion of 2D-NMR (400 MHz, DMSO-d_6) HMBC experiment of 4-hydroxy-*N*'-[(*E*)-(2-hydroxy-3-phenyl-phenyl)methylidene]benzohydrazide (**2a**).

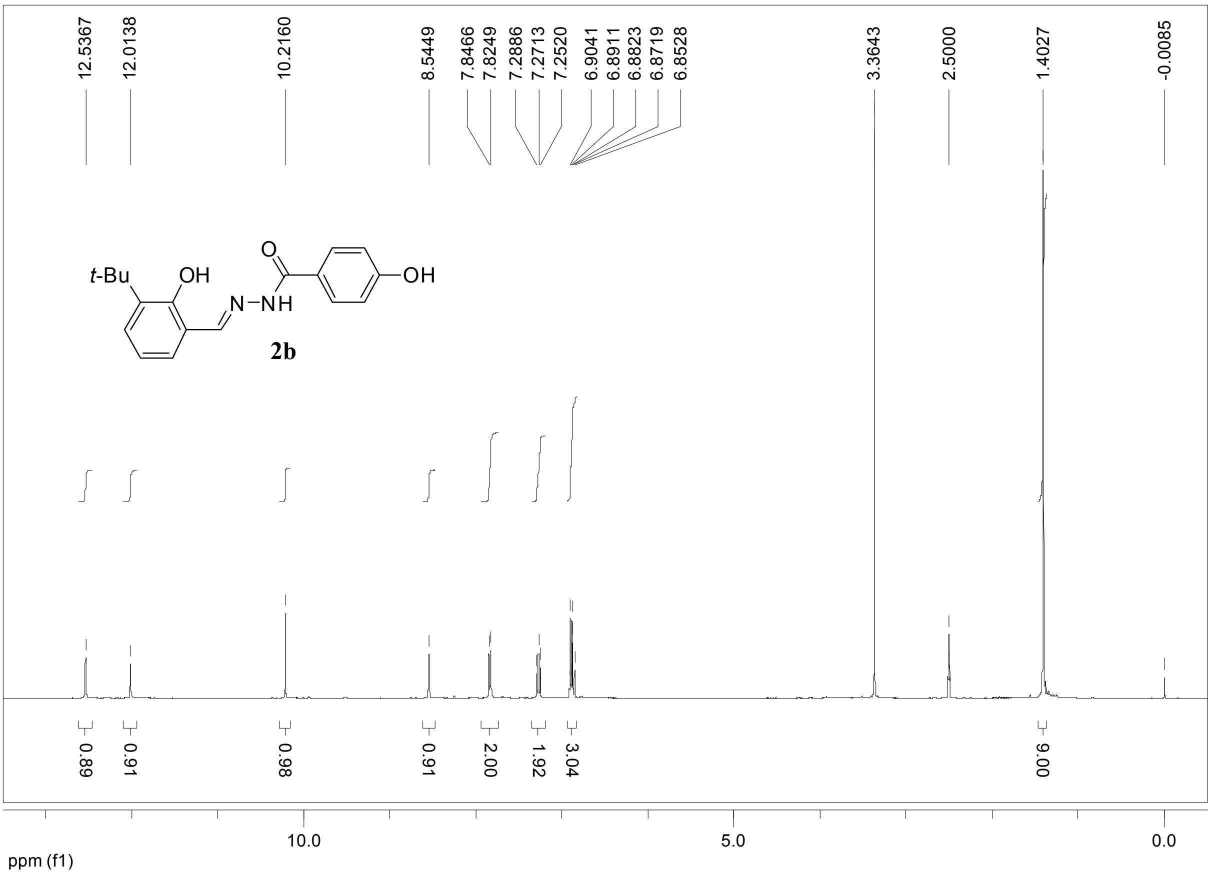

Figure S117. ¹H-NMR (400 MHz, DMSO-d_6) spectrum of compound **2b**.

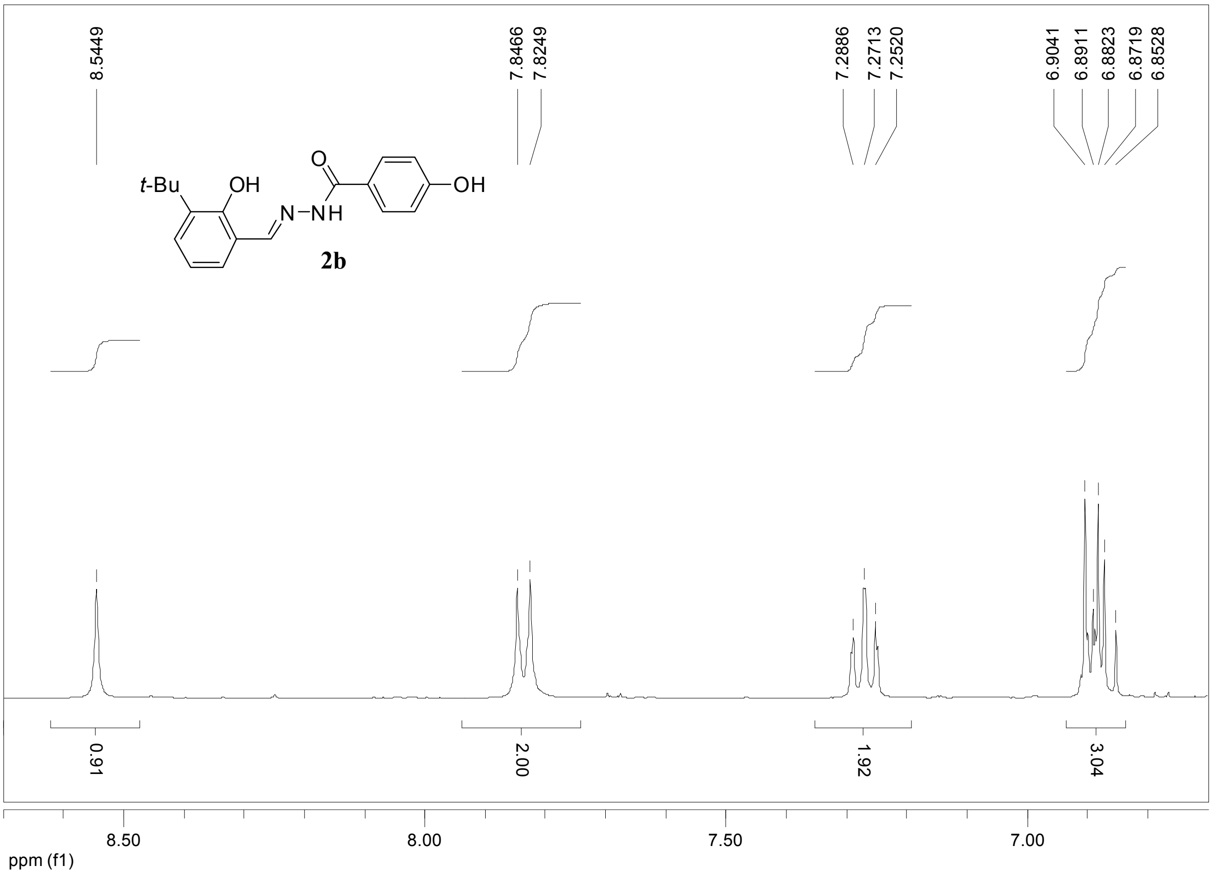

Figure S118. Expansion of ¹H-NMR (400 MHz, DMSO-d_6) spectrum of compound **2b**.

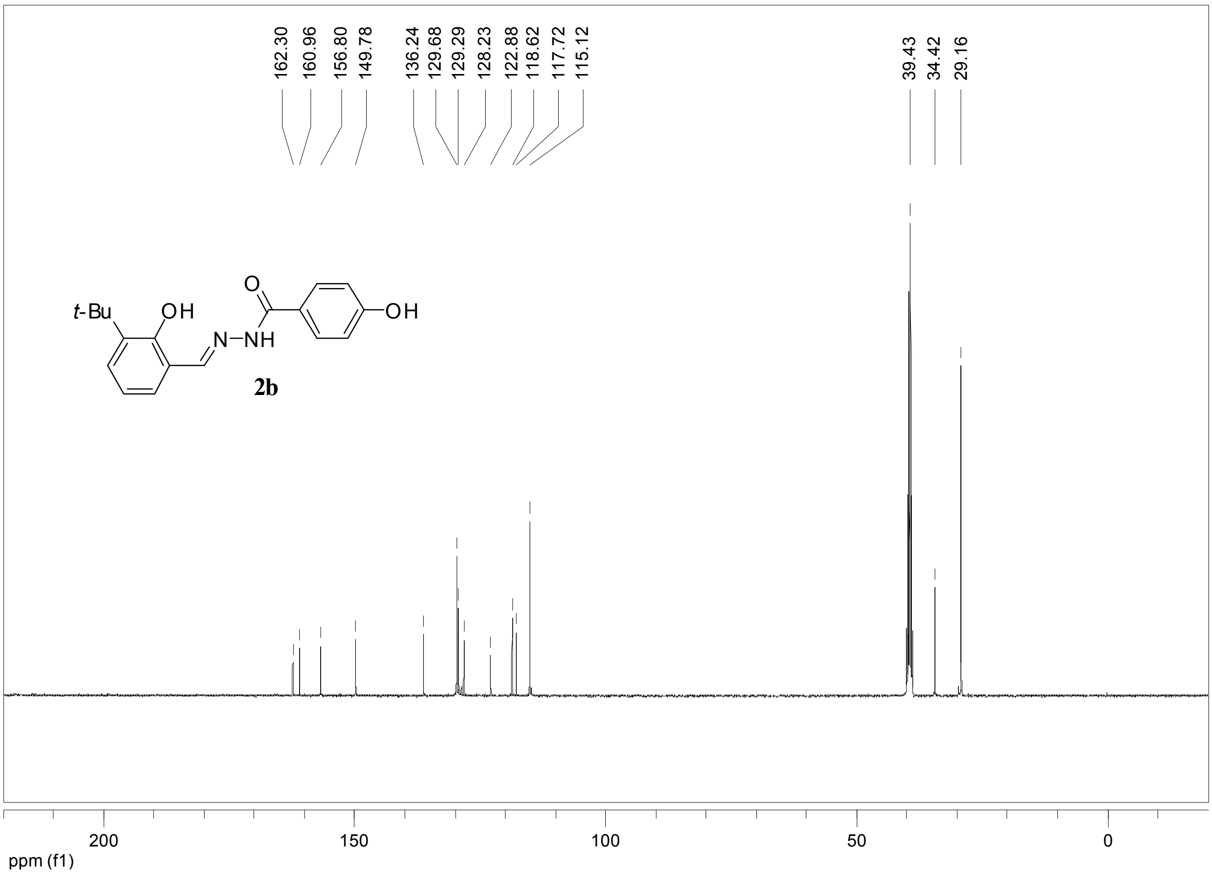

Figure S119. ^{13}C-NMR (100 MHz, DMSO-d_6) spectrum of compound **2b**.

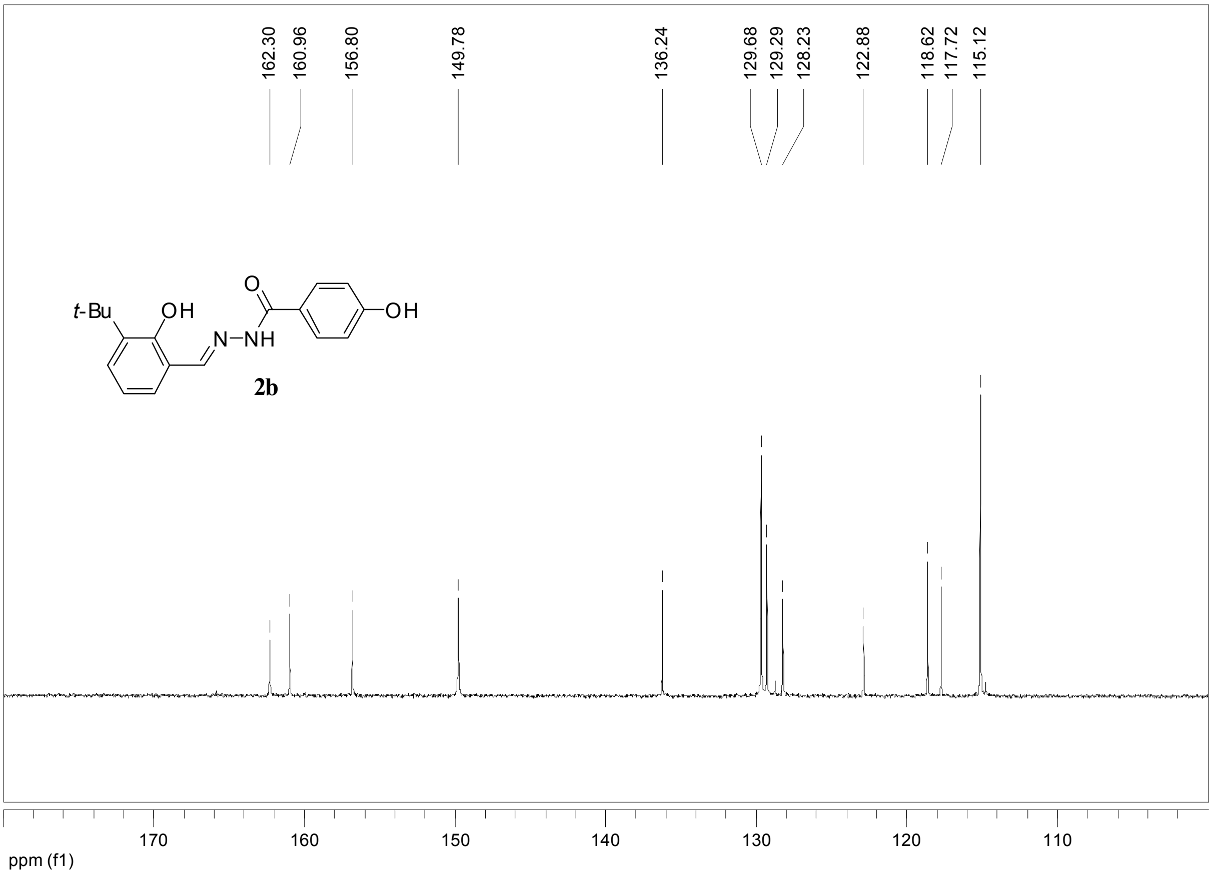

Figure S120. Expansion of ^{13}C-NMR (100 MHz, DMSO-d_6) spectrum of compound **2b**.

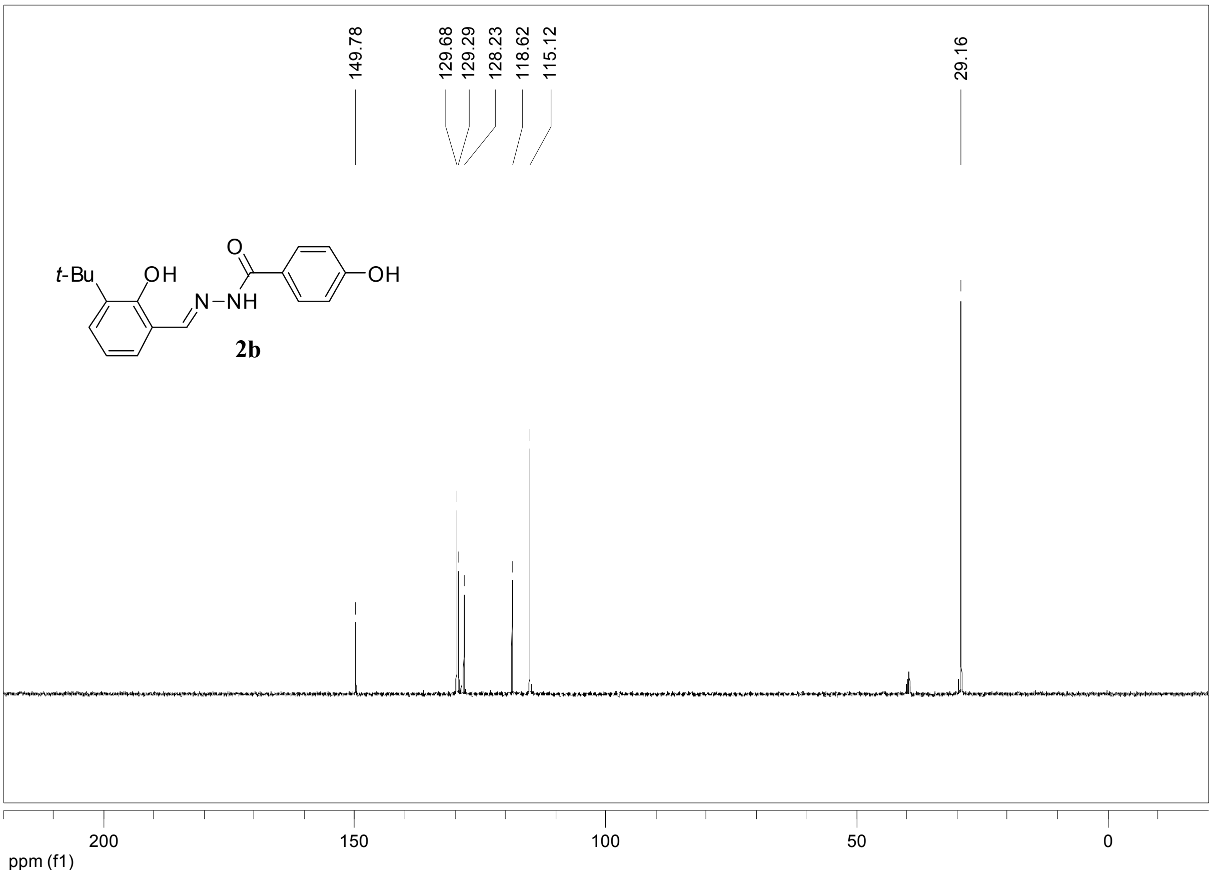

Figure S121. ^{13}C-NMR (100 MHz, DMSO-d_6) dept-135 experiment of compound **2b**.

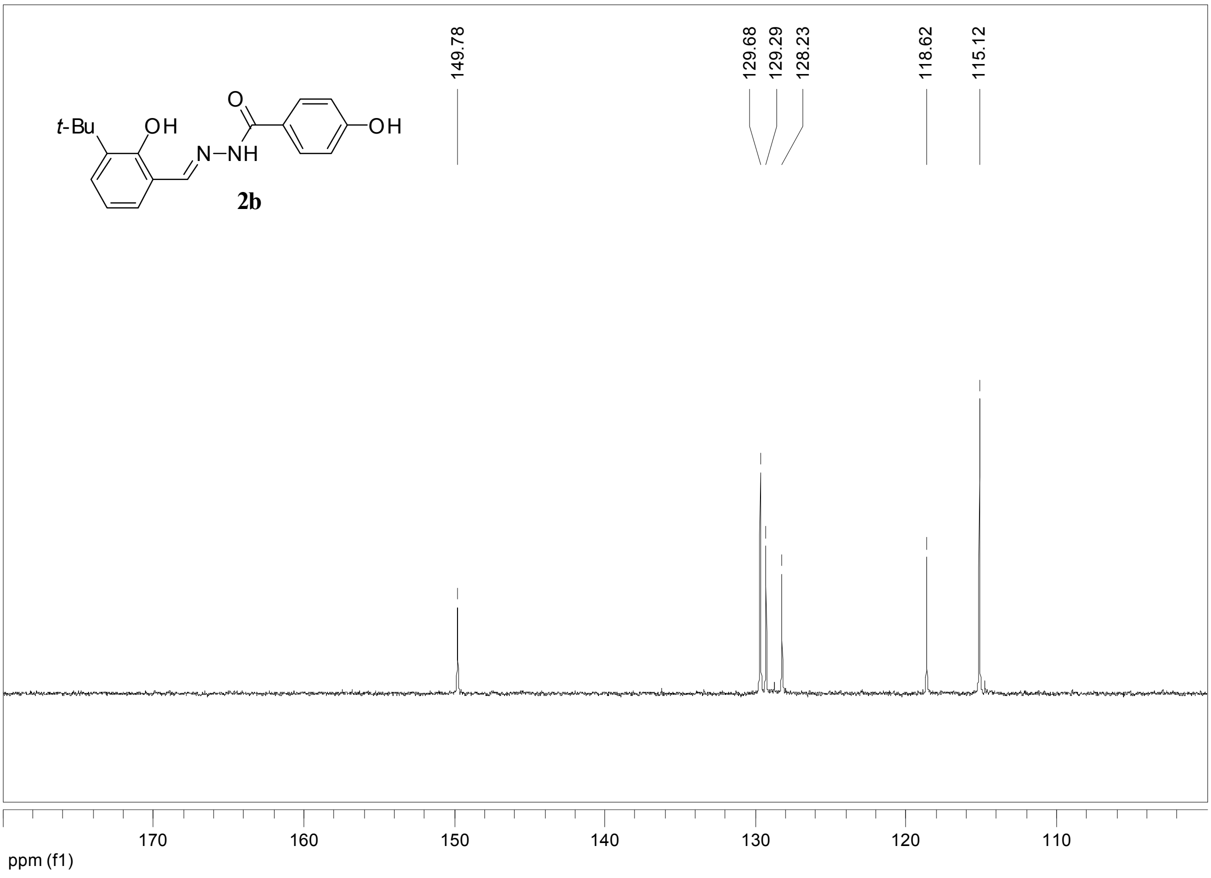

Figure S122. Expansion of ^{13}C-NMR (100 MHz, DMSO-d_6) dept-135 experiment of compound **2b**.

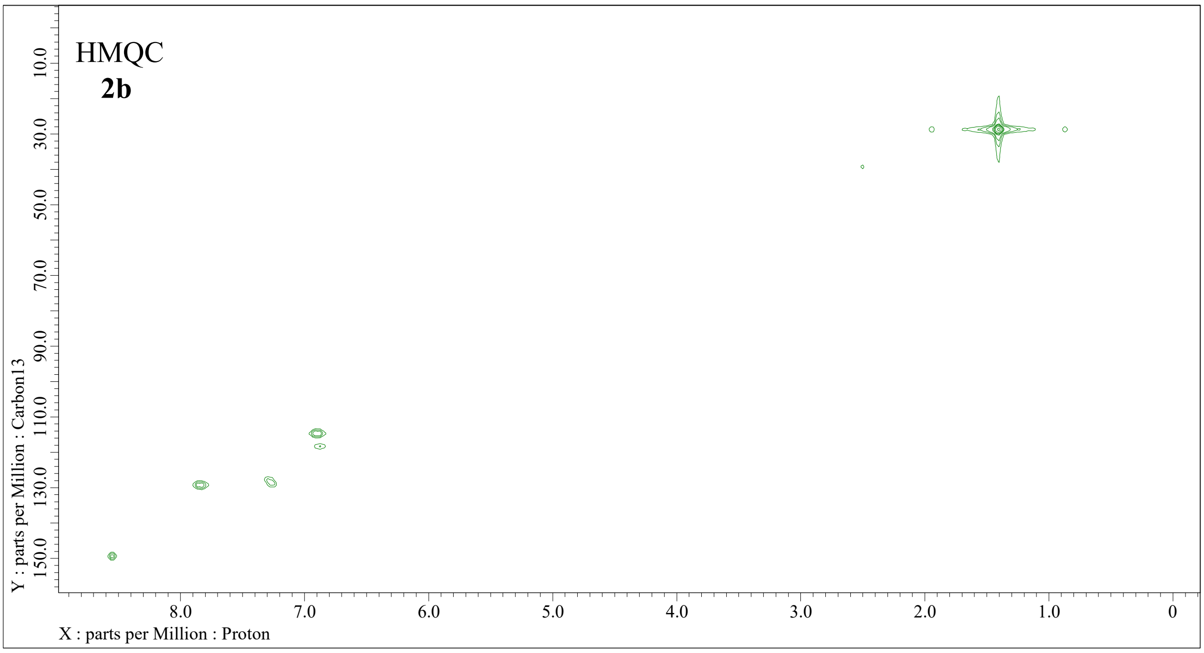

Figure S123. 2D-NMR (400 MHz, DMSO-d_6) HMQC experiment of 4-hydroxy-*N*'-[(*E*)-(3-*tert*-butyl-2-hydroxyphenyl)methylidene]benzohydrazide (**2b**).

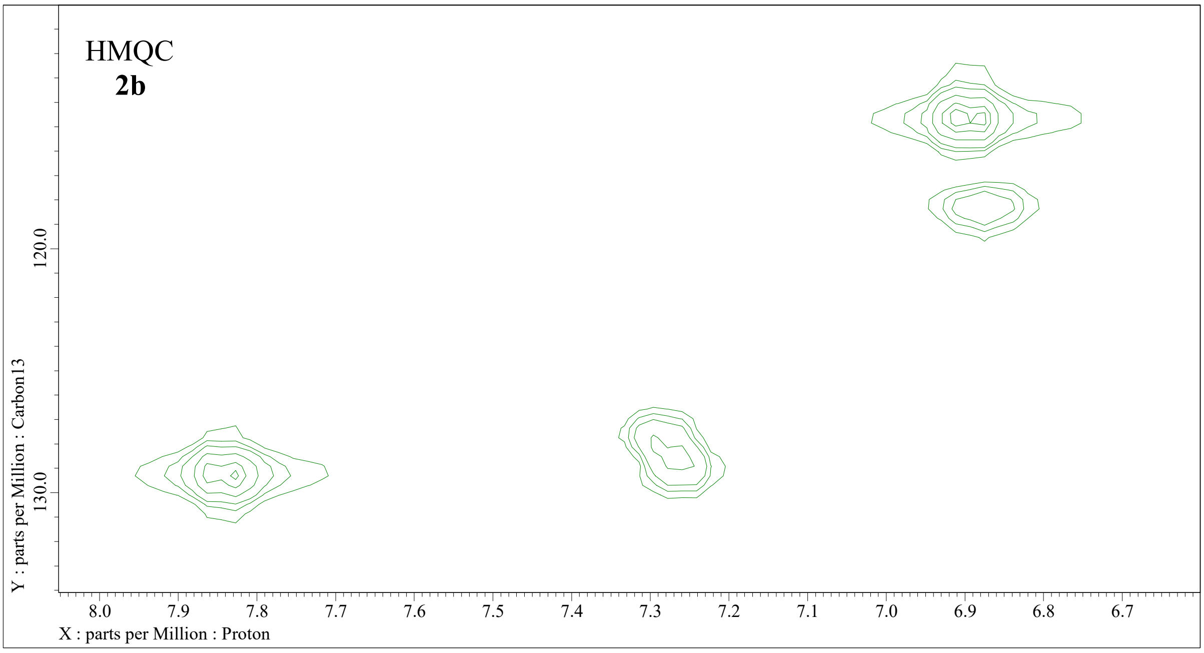

Figure S124. Expansion of 2D-NMR (400 MHz, DMSO-d_6) HMQC experiment of 4-hydroxy-*N*'-[(*E*)-(3-*tert*-butyl-2-hydroxyphenyl)methylidene]benzohydrazide (**2b**).

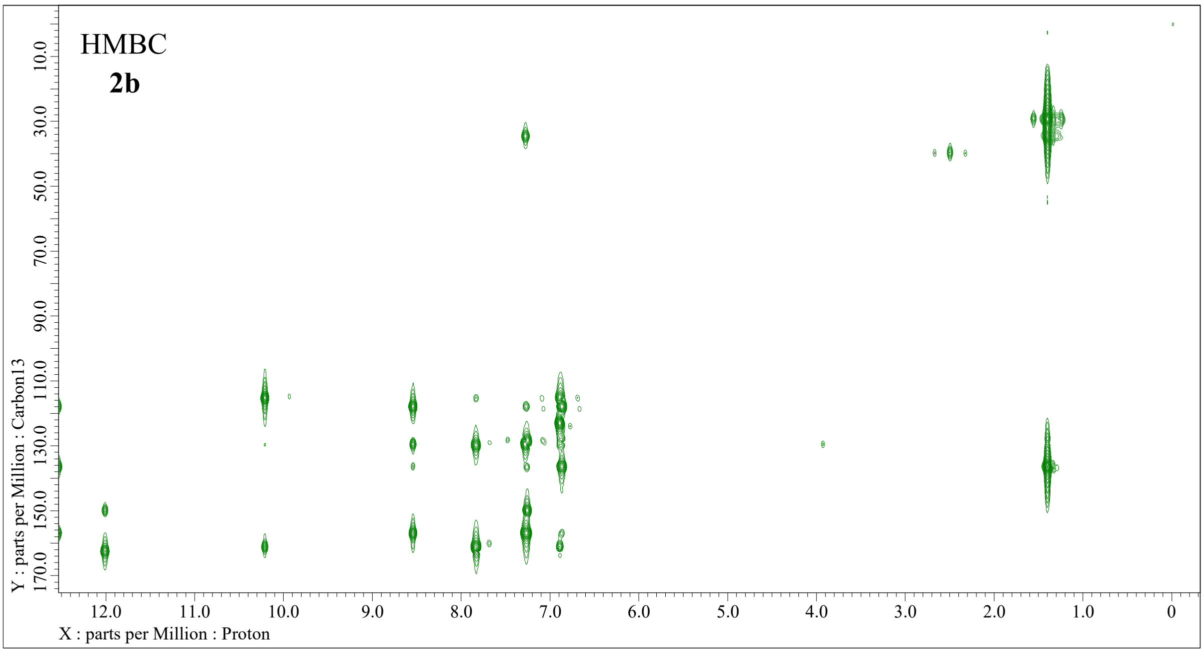

Figure S125. 2D-NMR (400 MHz, DMSO-d_6) HMBC experiment of 4-hydroxy-*N*'-[(*E*)-(3-*tert*-butyl-2-hydroxyphenyl)methylidene]benzohydrazide (**2b**).

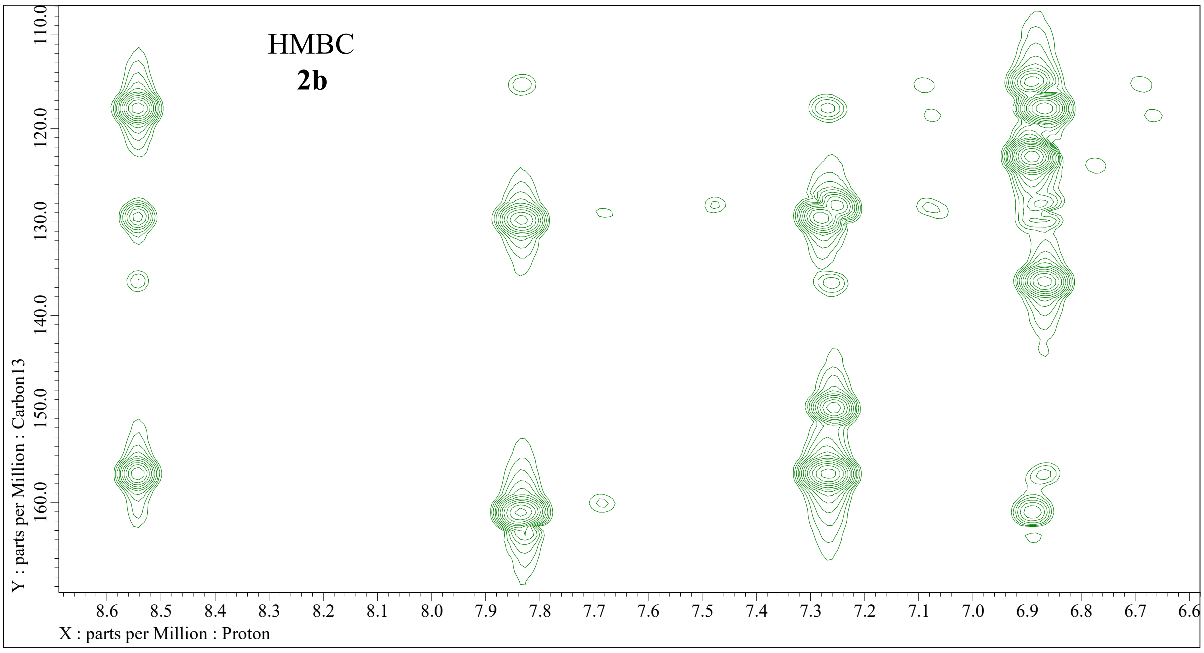

Figure S126. Expansion of 2D-NMR (400 MHz, DMSO-d_6) HMBC experiment of 4-hydroxy-*N*'-[(*E*)-(3-*tert*-butyl-2-hydroxyphenyl)methylidene]benzohydrazide (**2b**).

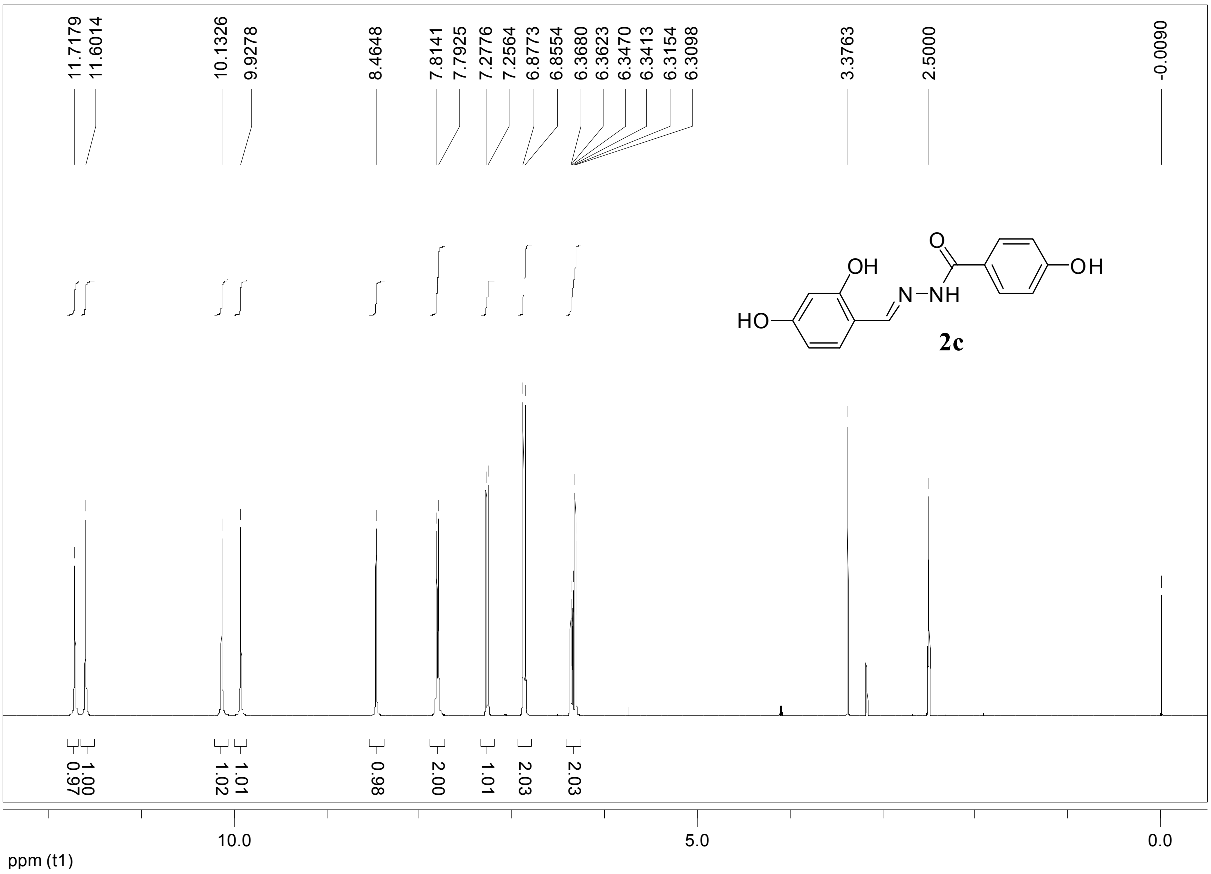

Figure S127. ^{1}H-NMR (400 MHz, DMSO-d_6) spectrum of compound **2c**.

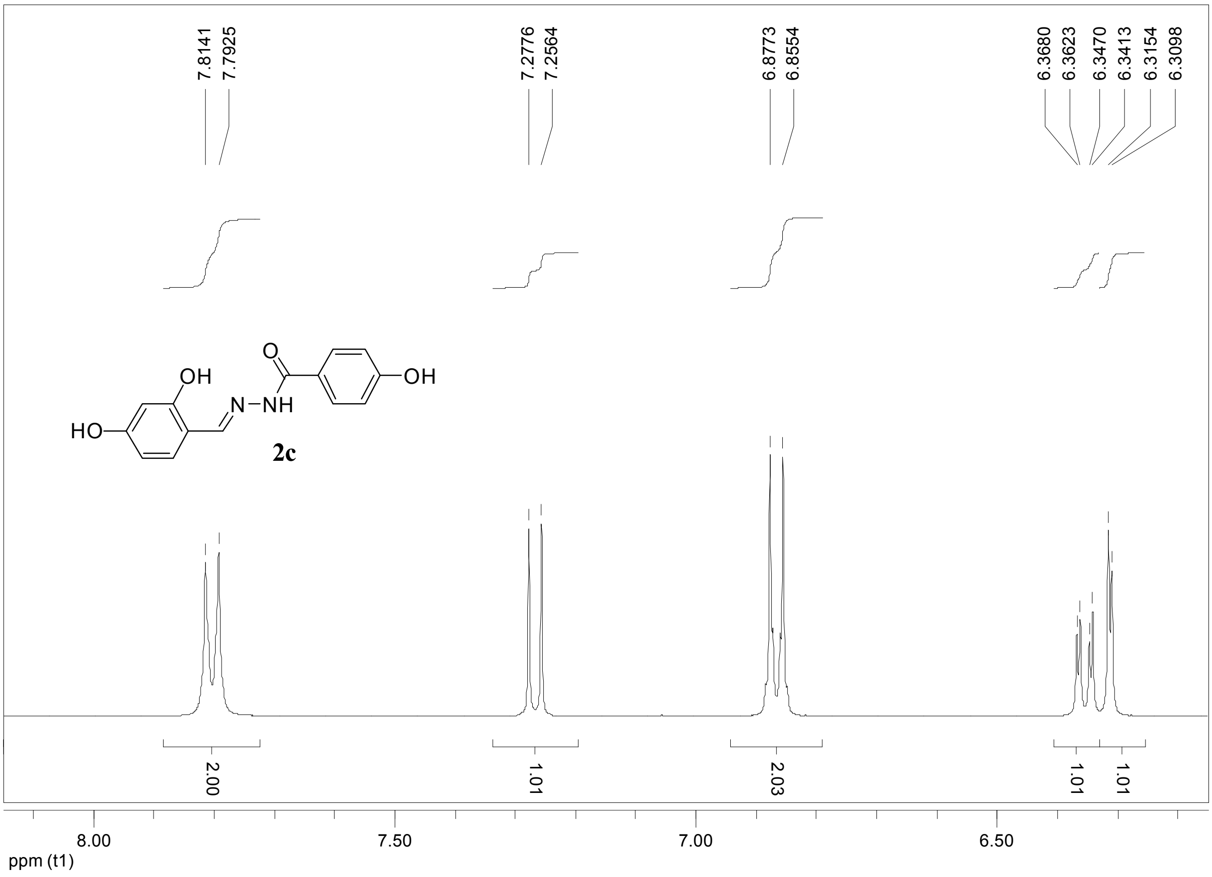

Figure S128. Expansion of ^{1}H-NMR (400 MHz, DMSO-d_6) spectrum of compound **2c**.

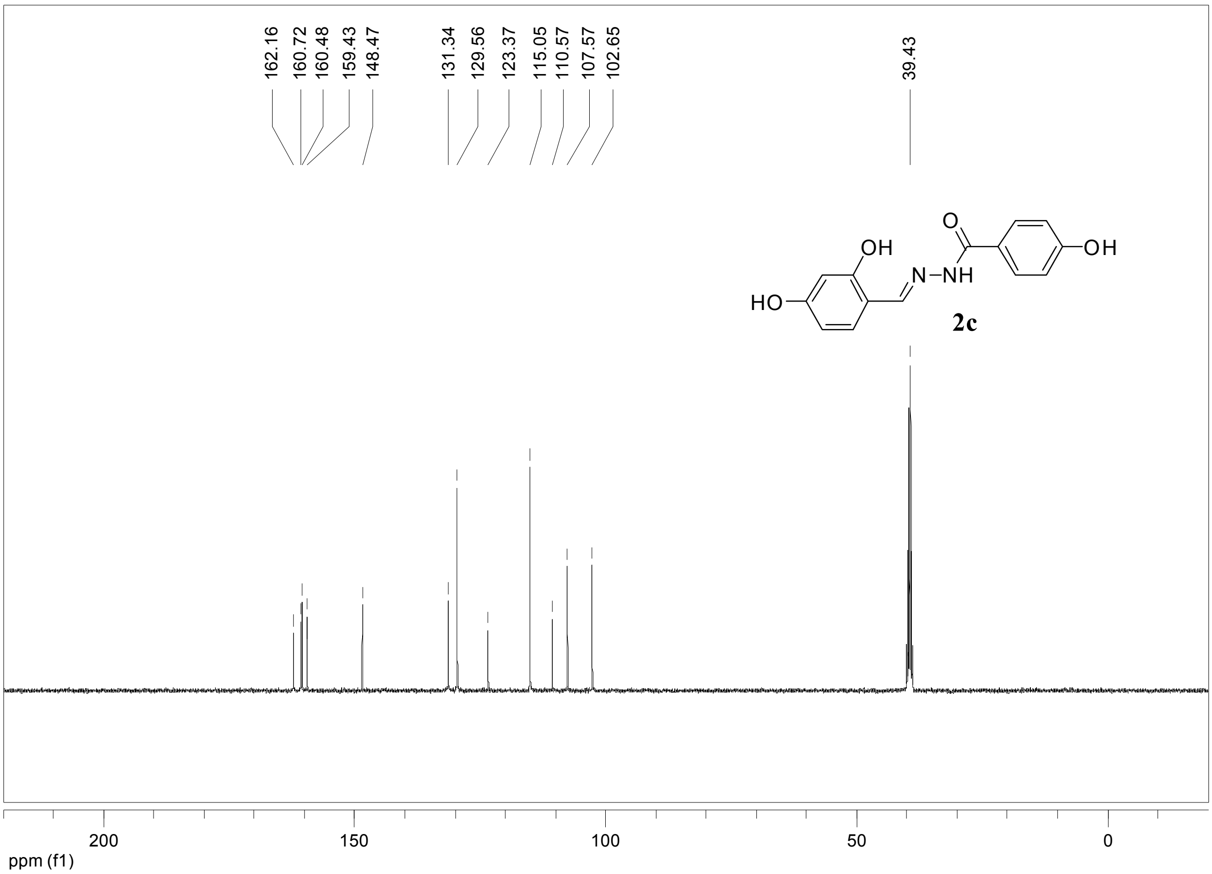

Figure S129. ^{13}C-NMR (100 MHz, DMSO-d_6) spectrum of compound **2c**.

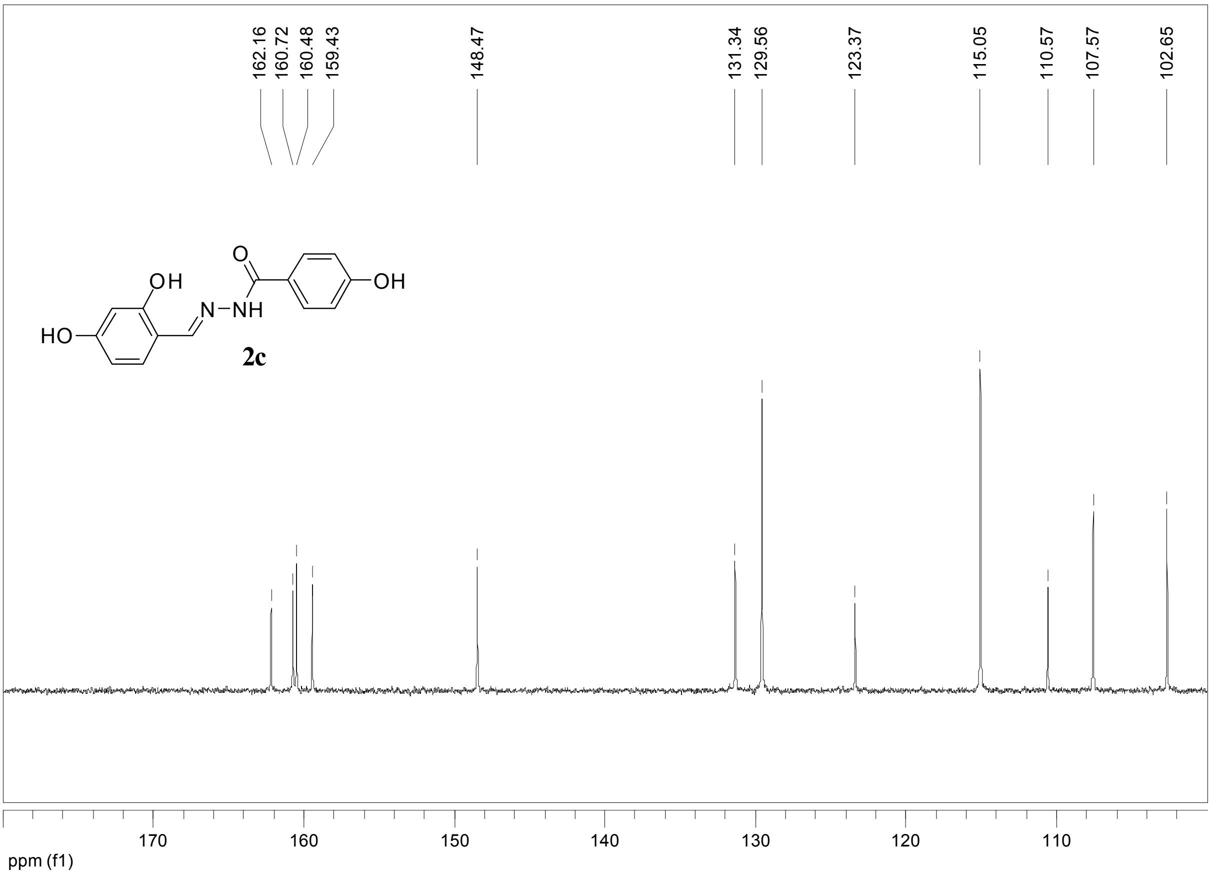

Figure S130. Expansion of ^{13}C-NMR (100 MHz, DMSO-d_6) spectrum of compound **2c**.

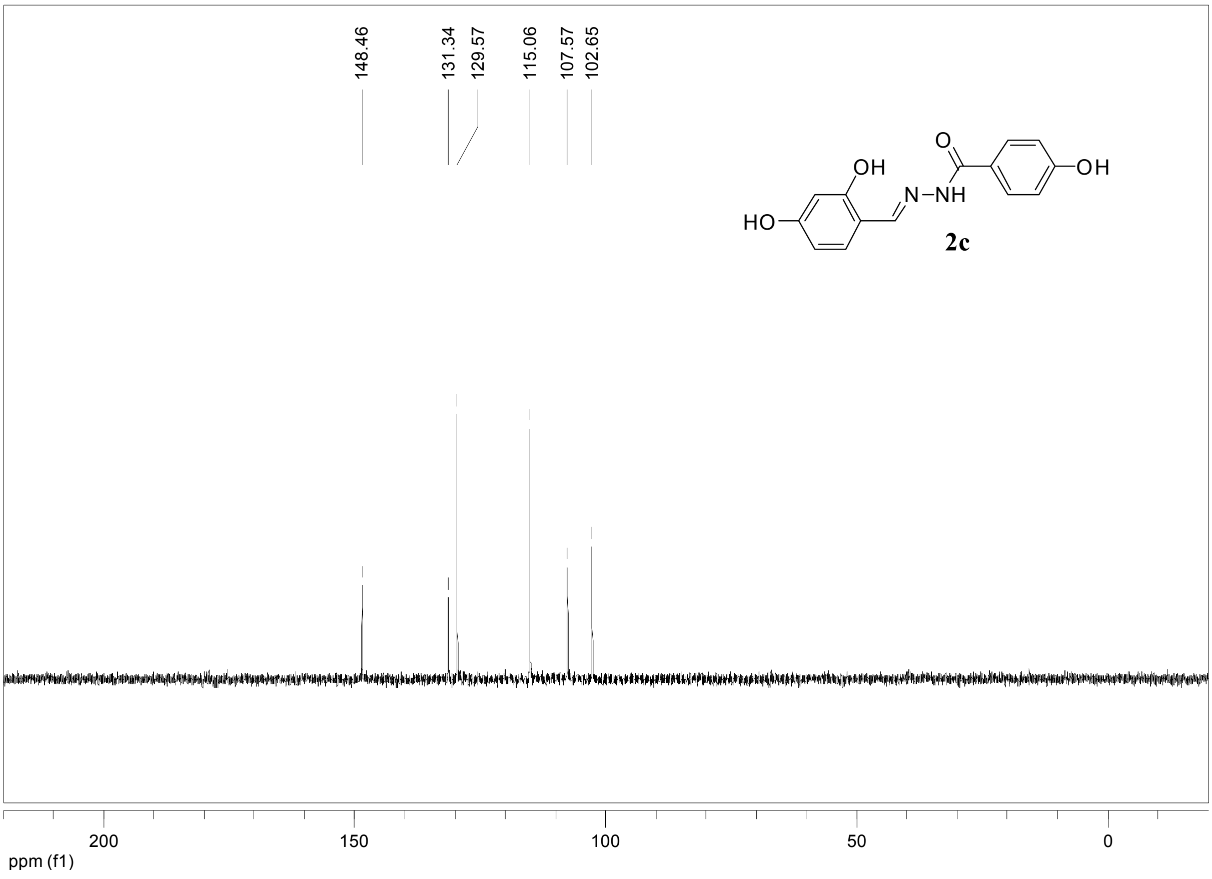

Figure S131. ^{13}C-NMR (100 MHz, DMSO-d_6) dept-135 experiment of compound **2c**.

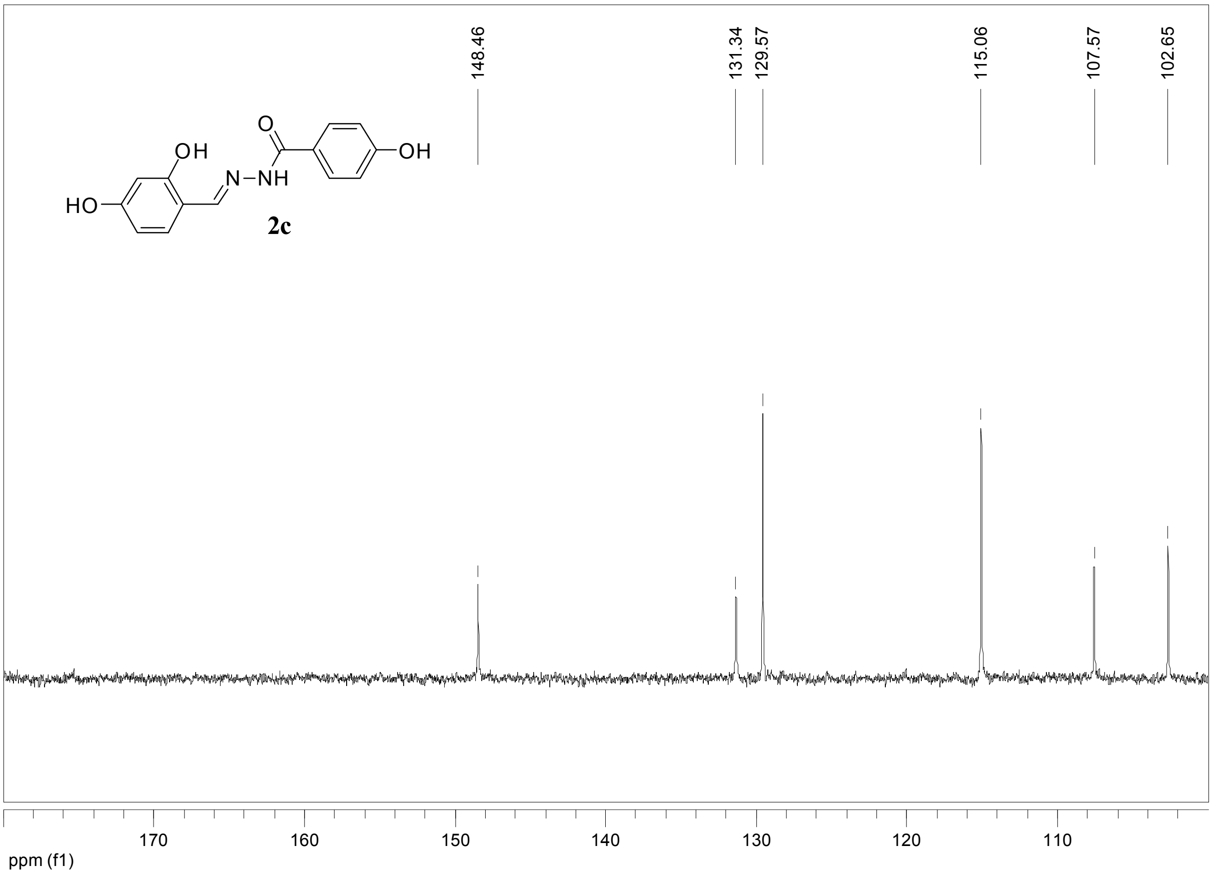

Figure S132. Expansion of ^{13}C-NMR (100 MHz, DMSO-d_6) dept-135 experiment of compound **2c**.

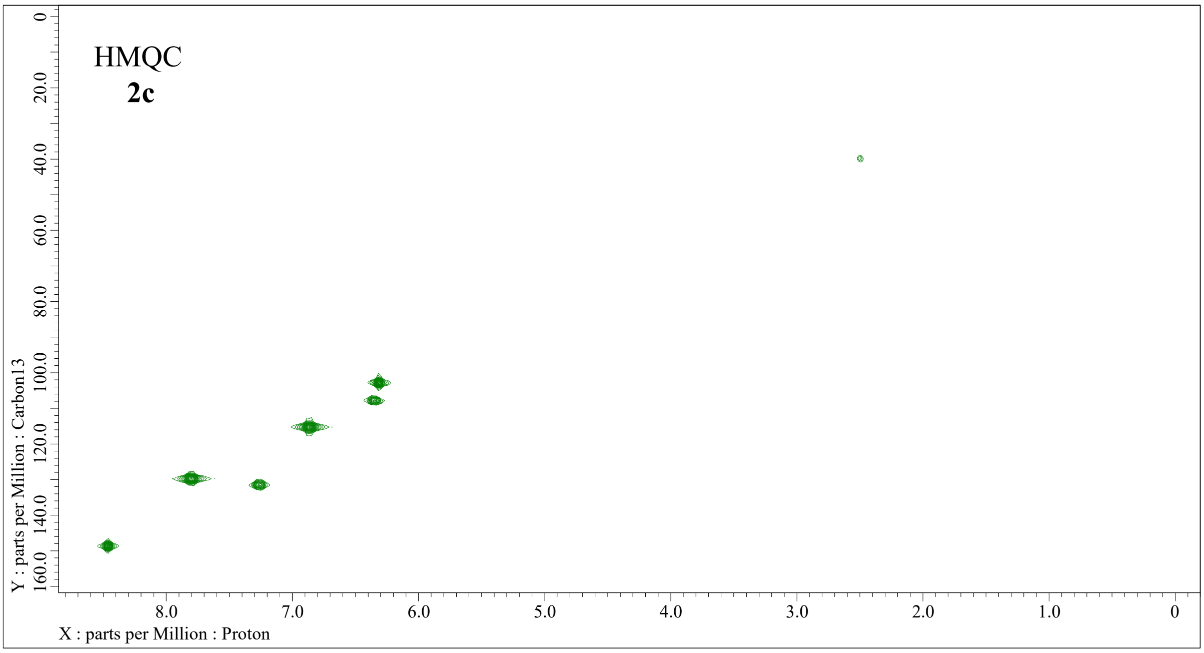

Figure S133. 2D-NMR (400 MHz, DMSO-d_6) HMQC experiment of 4-hydroxy-*N*'-[(*E*)-(2,4-dihydroxyphenyl)methylidene]benzohydrazide (**2c**).

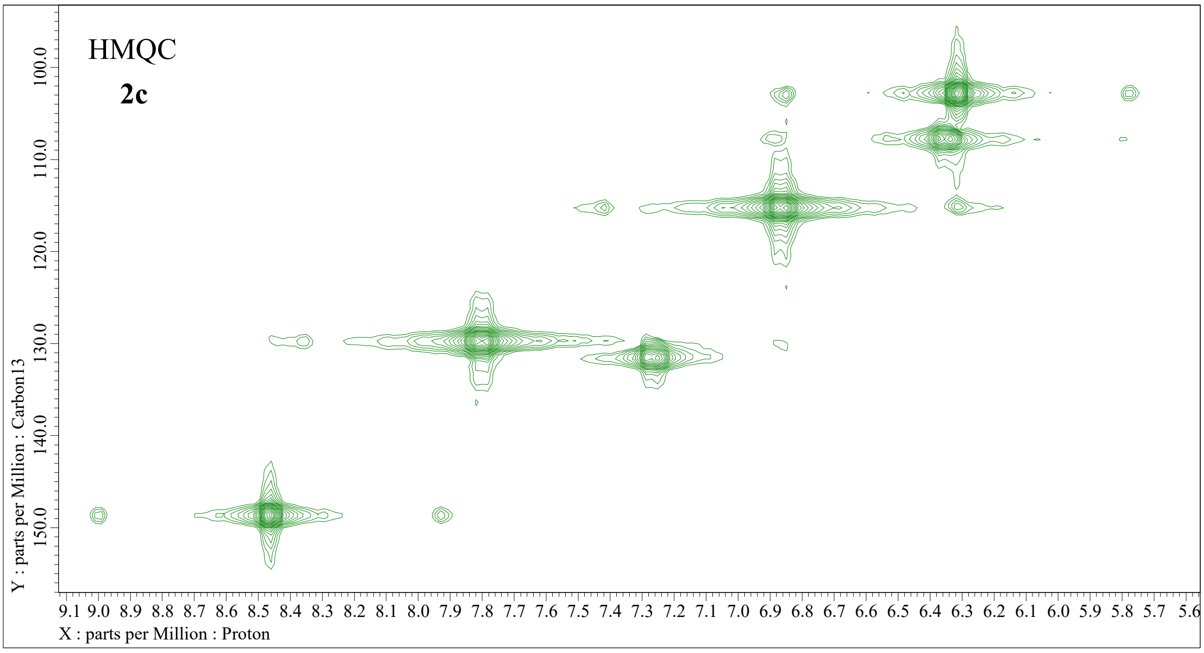

Figure S134. Expansion of 2D-NMR (400 MHz, DMSO-d_6) HMQC experiment of 4-hydroxy-*N*'-[(*E*)-(2,4-dihydroxyphenyl)methylidene]benzohydrazide (**2c**).

Molecules **2020**, *25*, 1255; doi:10.3390/molecules25051255 www.mdpi.com/journal/molecules

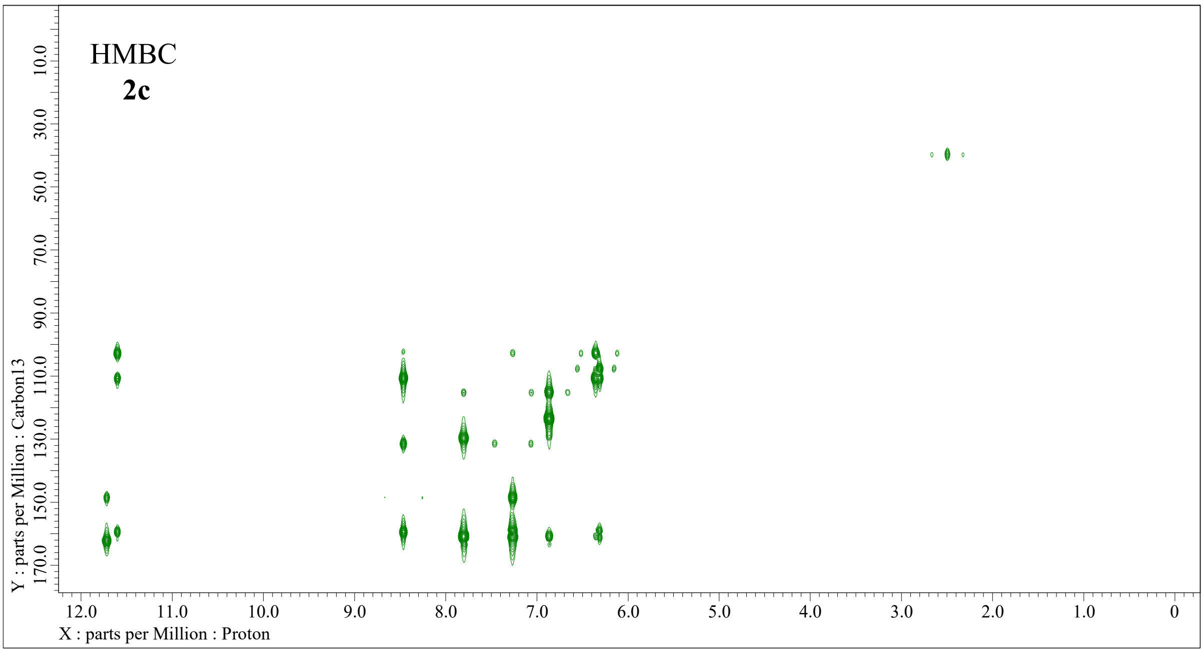

Figure S135. 2D-NMR (400 MHz, DMSO-d_6) HMBC experiment of 4-hydroxy-*N*'-[(*E*)-(2,4-dihydroxyphenyl)methylidene]benzohydrazide (**2c**).

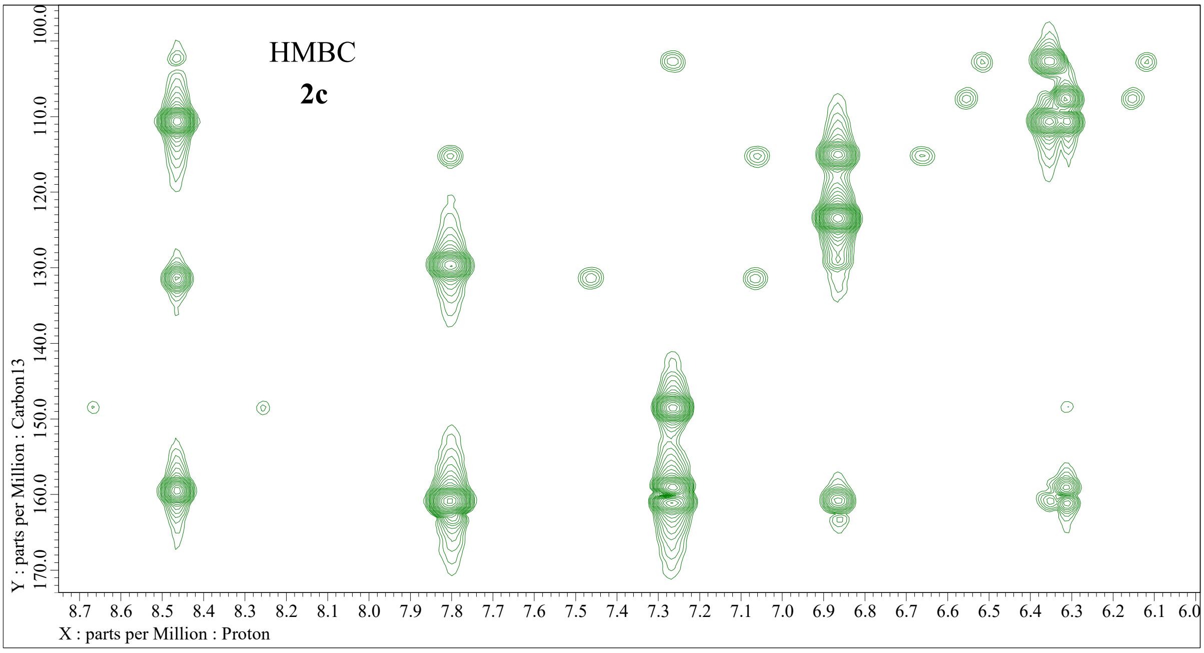

Figure S136. Expansion of 2D-NMR (400 MHz, DMSO-d_6) HMBC experiment of 4-hydroxy-*N*'-[(*E*)-(2,4-dihydroxyphenyl)methylidene]benzohydrazide (**2c**).

Molecules **2020**, *25*, 1255; doi:10.3390/molecules25051255 www.mdpi.com/journal/molecules

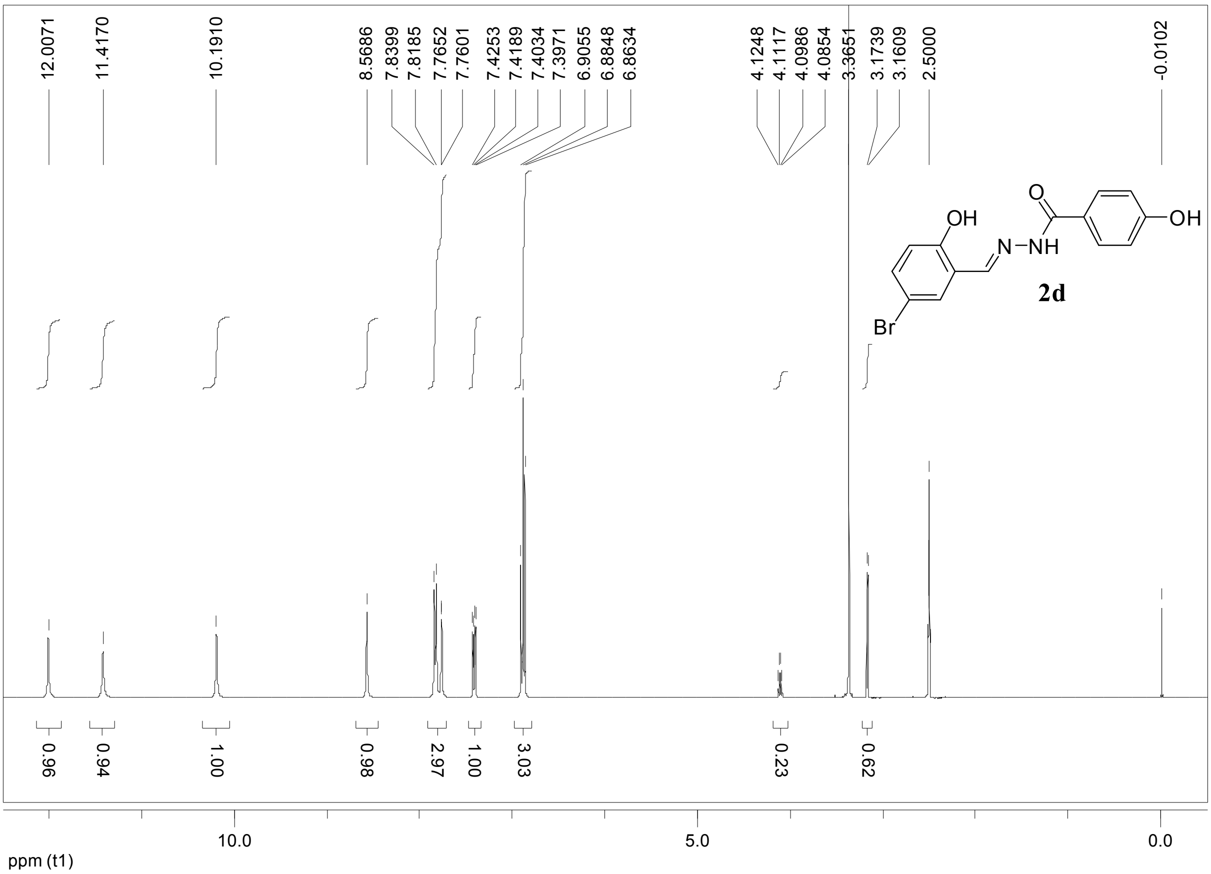

Figure S137. ^{1}H-NMR (400 MHz, DMSO-d_6) spectrum of compound **2d**.

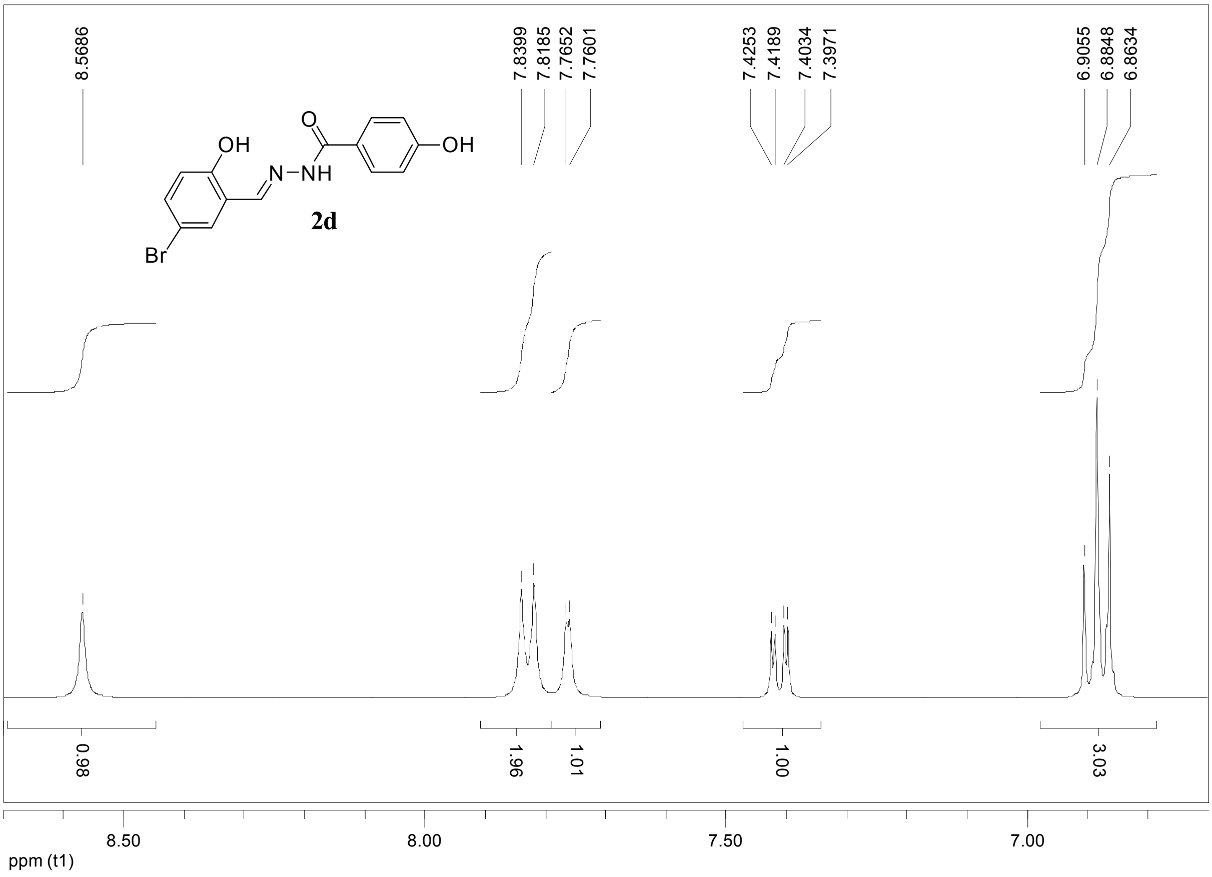

Figure S138. Expansion of ^{1}H-NMR (400 MHz, DMSO-d_6) spectrum of compound **2d**.

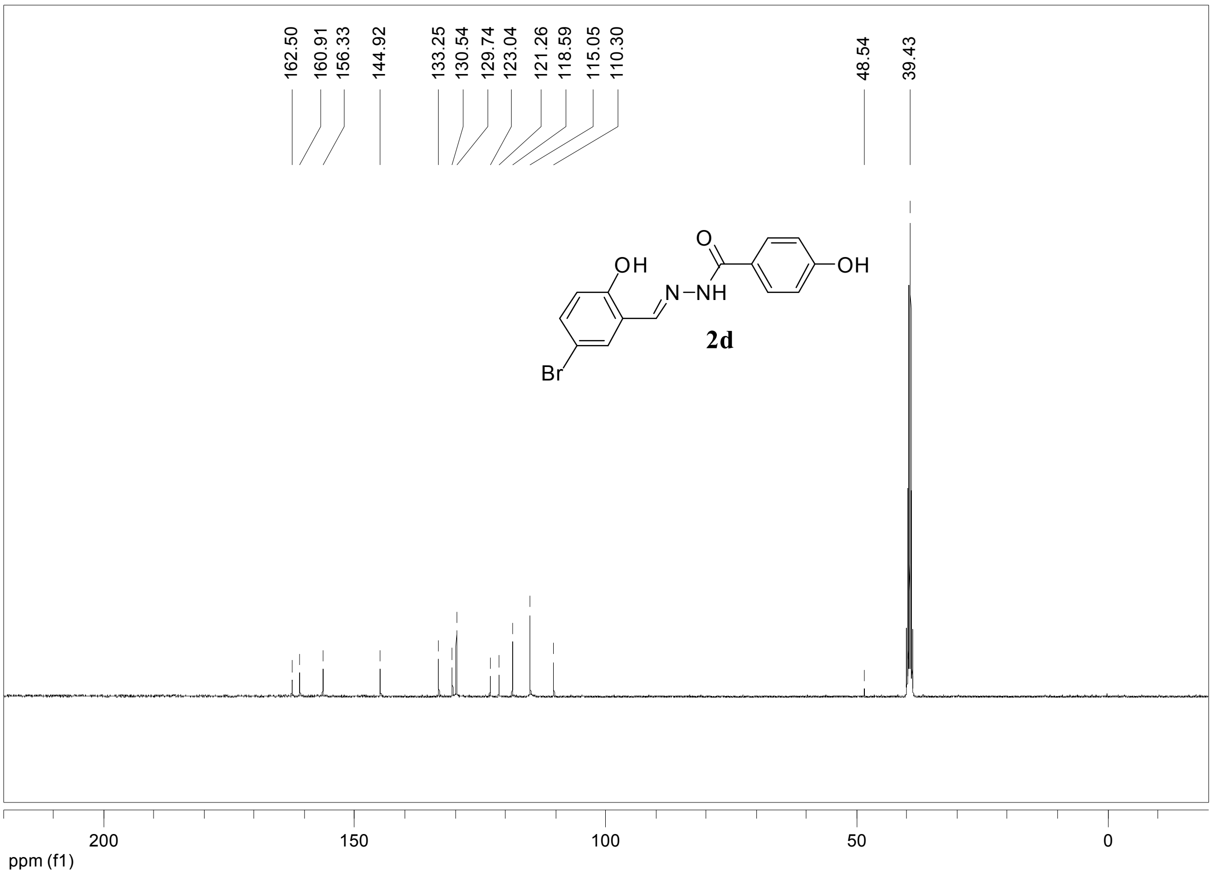

Figure S139. ^{13}C-NMR (100 MHz, DMSO-d_6) spectrum of compound **2d**.

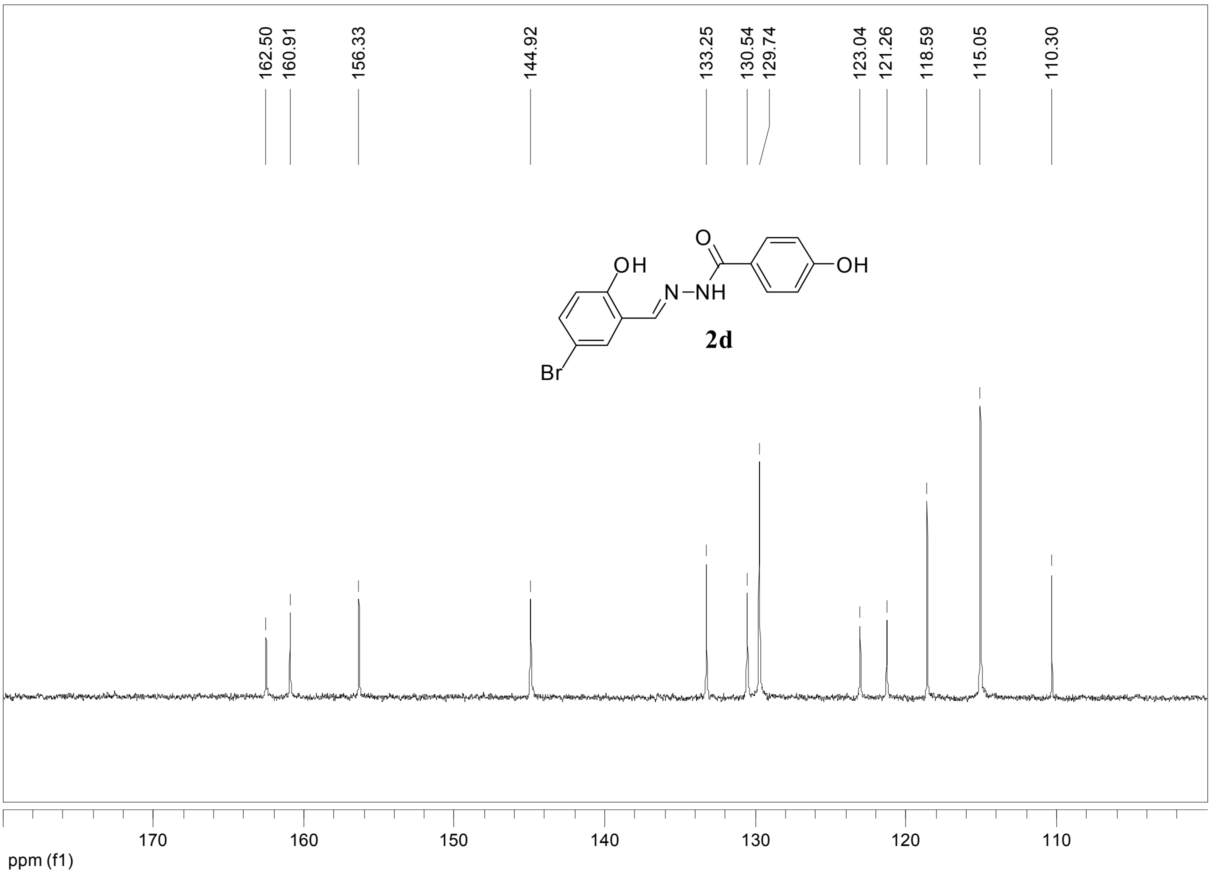

Figure S140. Expansion of ^{13}C-NMR (100 MHz, DMSO-d_6) spectrum of compound **2d**.

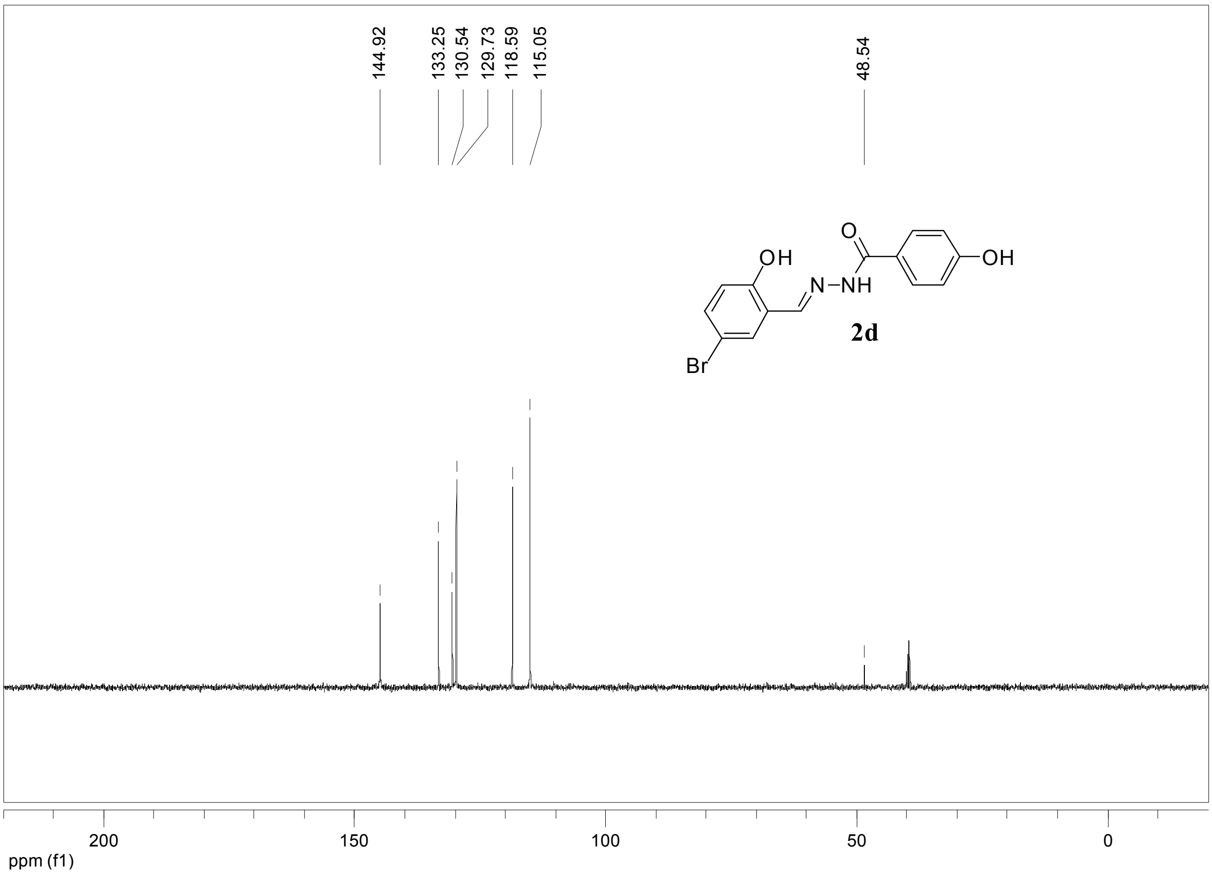

Figure S141. ^{13}C-NMR (100 MHz, DMSO-d_6) dept-135 experiment of compound **2d**.

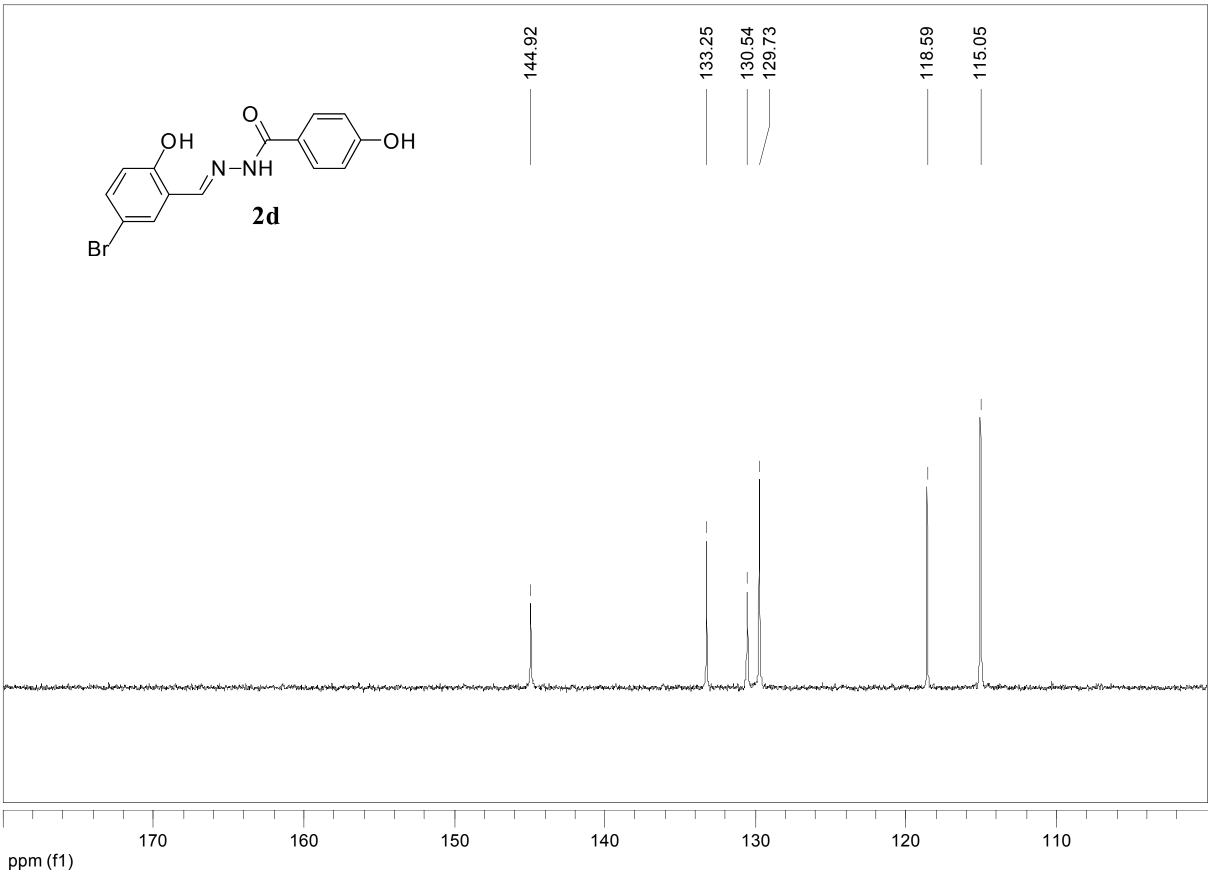

Figure S142. Expansion of ^{13}C-NMR (100 MHz, DMSO-d_6) dept-135 experiment of compound **2d**.

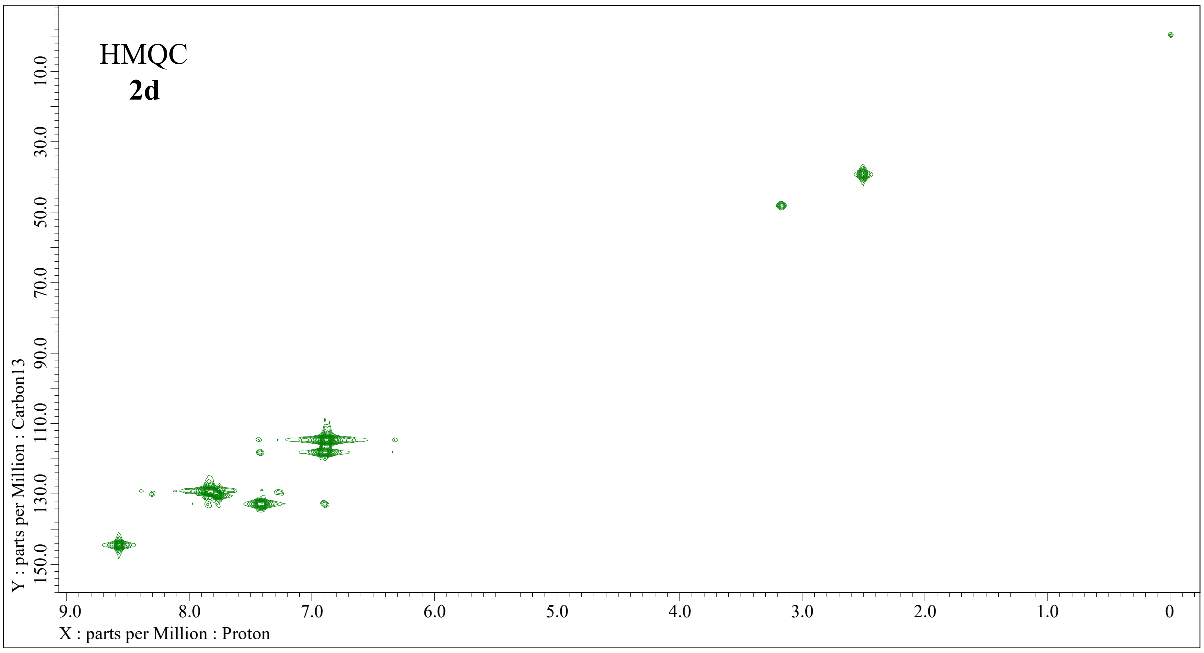

Figure S143. 2D-NMR (400 MHz, DMSO-d_6) HMQC experiment of 4-hydroxy-*N*'-[(*E*)-(5-bromo-2-hydroxyphenyl)methylidene]benzohydrazide (**2d**).

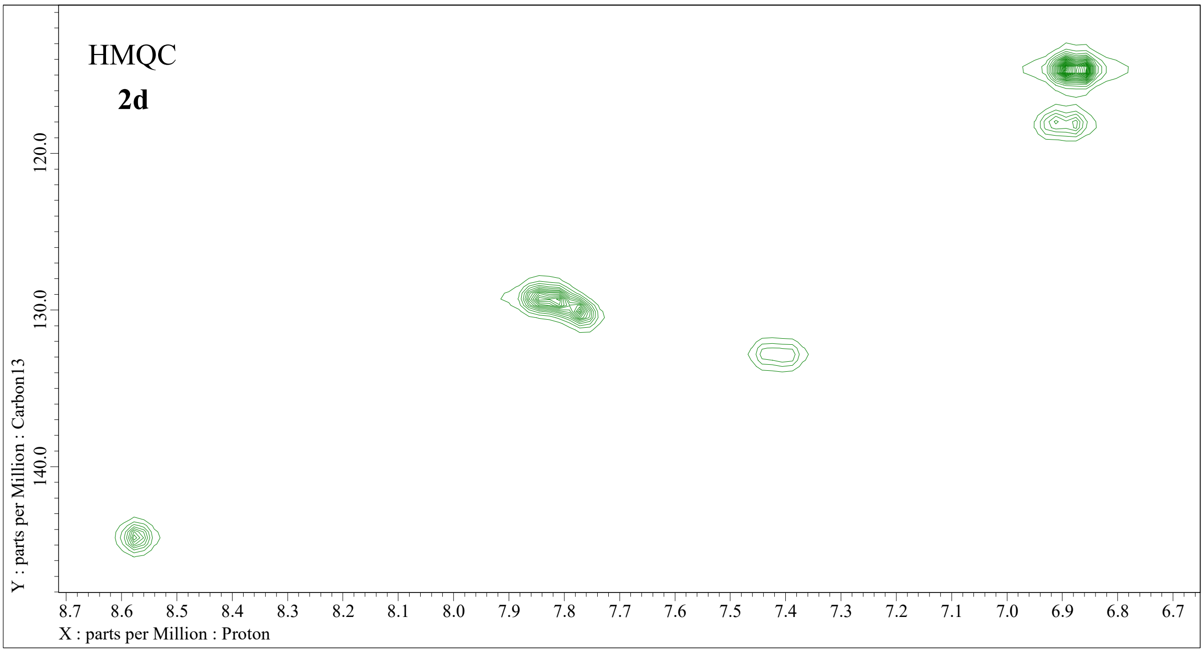

Figure S144. Expansion of 2D-NMR (400 MHz, DMSO-d_6) HMQC experiment of 4-hydroxy-*N*'-[(*E*)-(5-bromo-2-hydroxyphenyl)methylidene]benzohydrazide (**2d**).

Molecules **2020**, *25*, 1255; doi:10.3390/molecules25051255 www.mdpi.com/journal/molecules

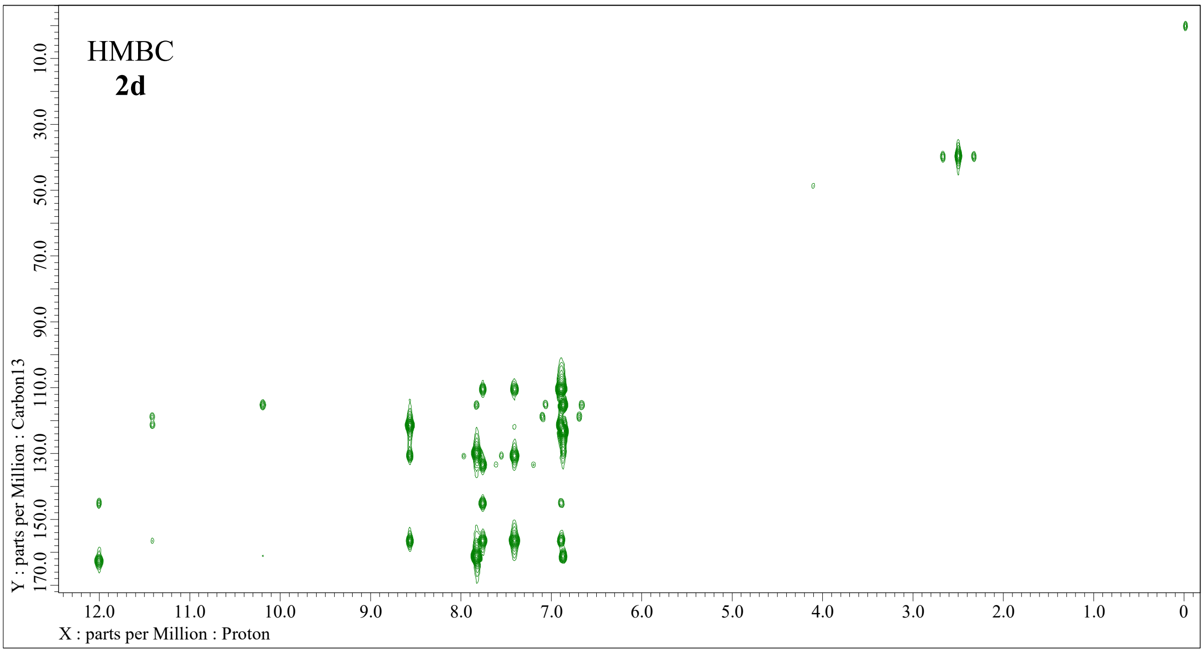

Figure S145. 2D-NMR (400 MHz, DMSO-d_6) HMBC experiment of 4-hydroxy-*N*'-[(*E*)-(5-bromo-2-hydroxyphenyl)methylidene]benzohydrazide (**2d**).

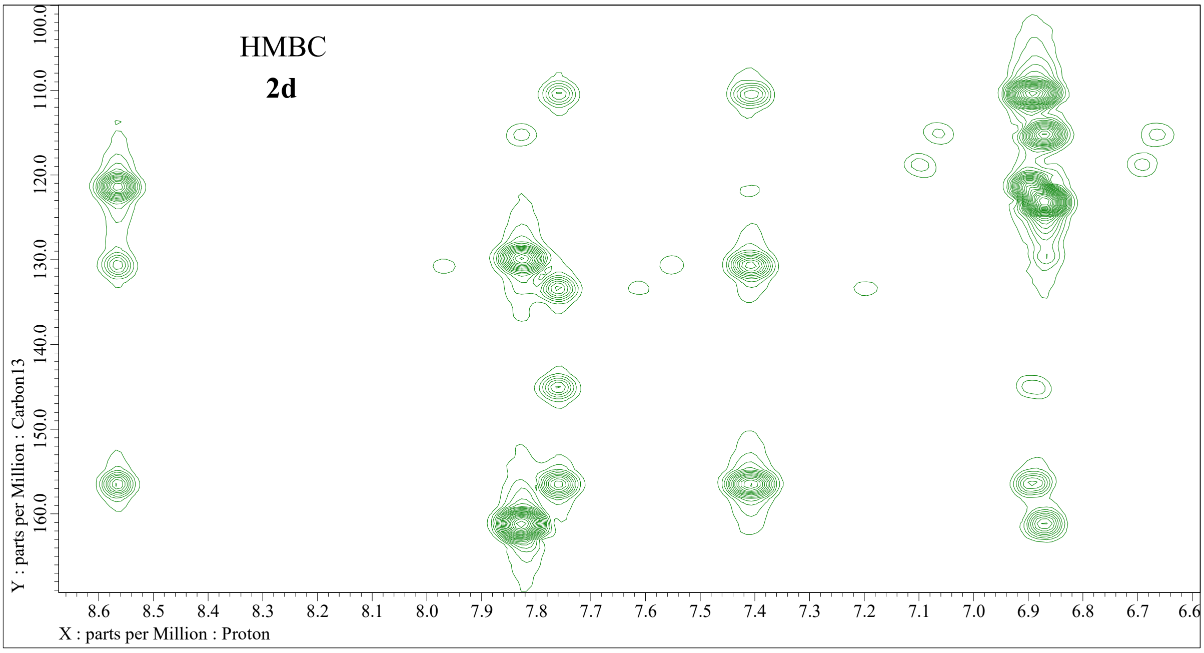

Figure S146. Expansion of 2D-NMR (400 MHz, DMSO-d_6) HMBC experiment of 4-hydroxy-*N*'-[(*E*)-(5-bromo-2-hydroxyphenyl)methylidene]benzohydrazide (**2d**).

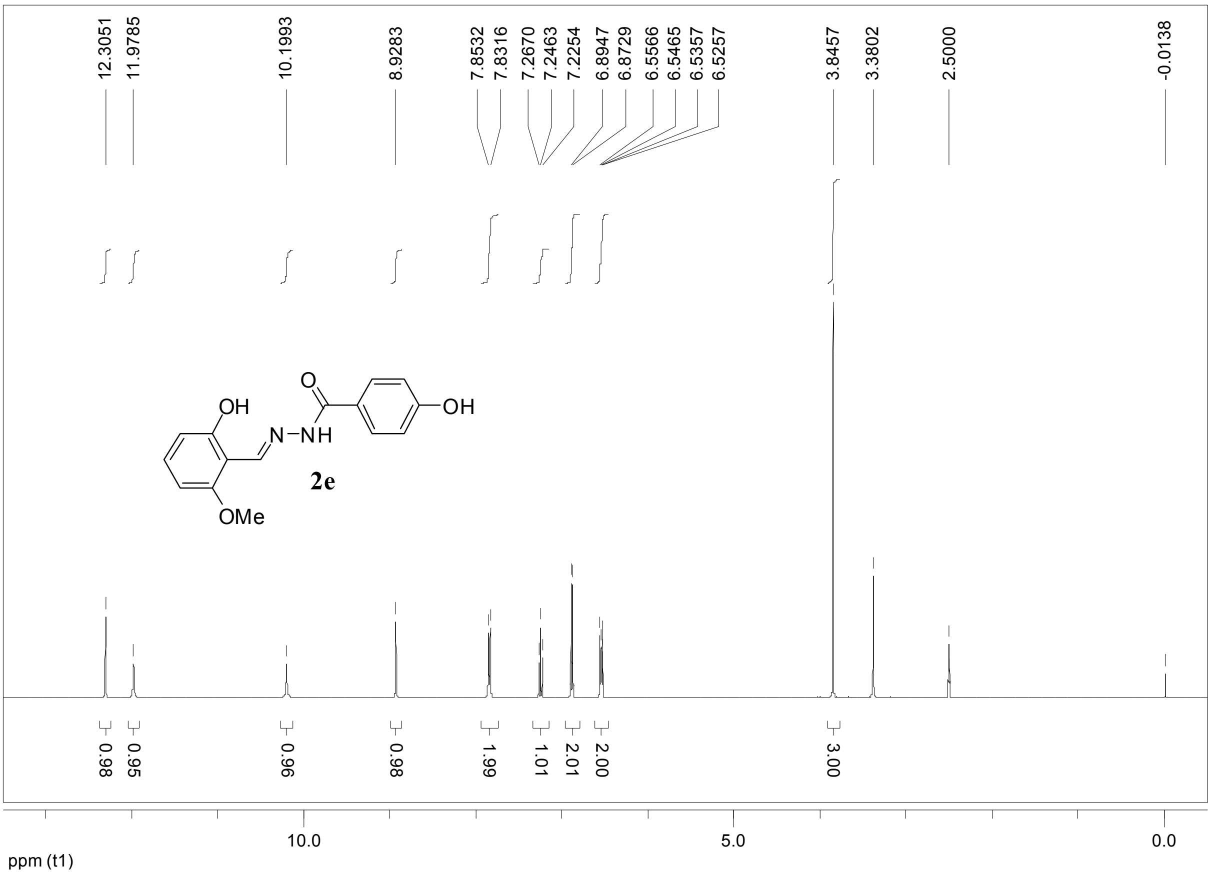

Figure S147. ^{1}H-NMR (400 MHz, DMSO-d_6) spectrum of compound **2e**.

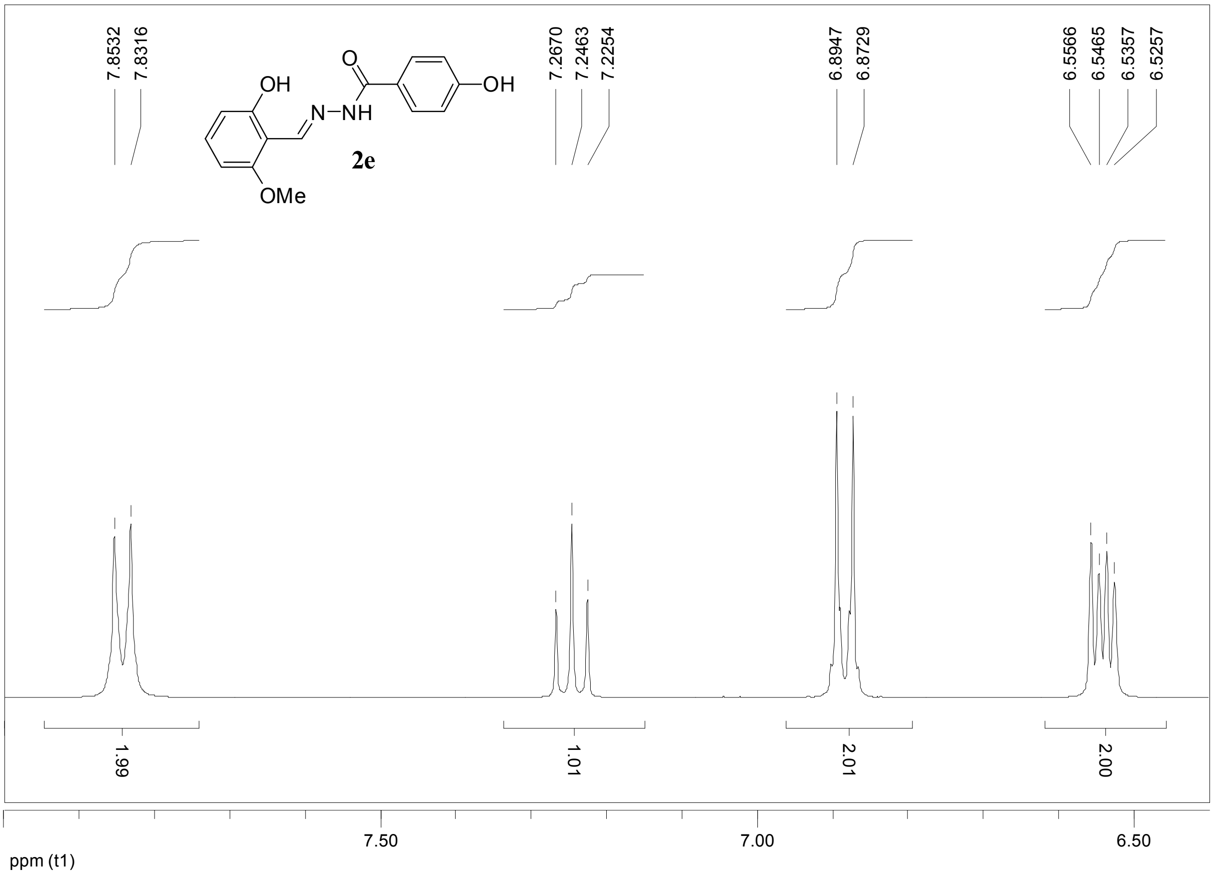

Figure S148. Expansion of ^{1}H-NMR (400 MHz, DMSO-d_6) spectrum of compound **2e**.

Molecules **2020**, *25*, 1255; doi:10.3390/molecules25051255 www.mdpi.com/journal/molecules

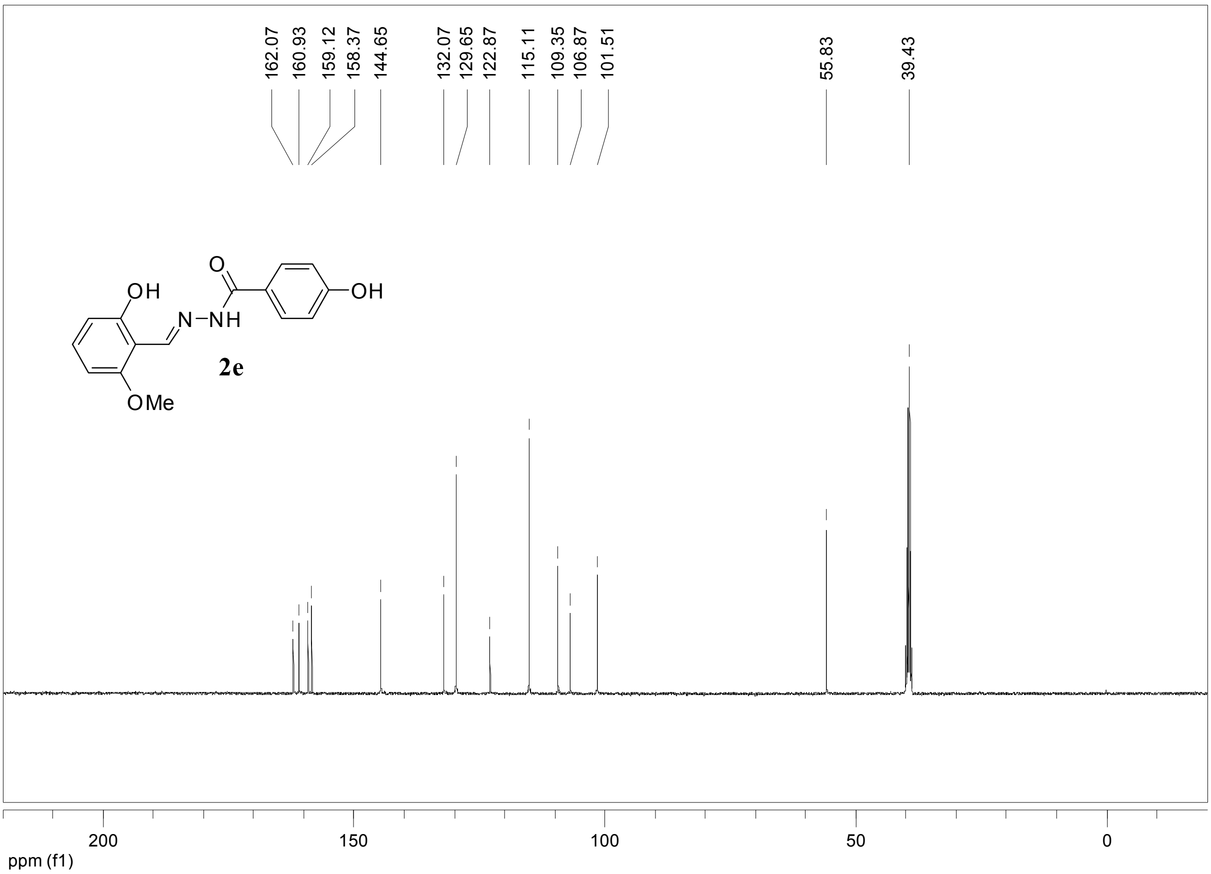

Figure S149. ^{13}C-NMR (100 MHz, DMSO-d_6) spectrum of compound **2e**.

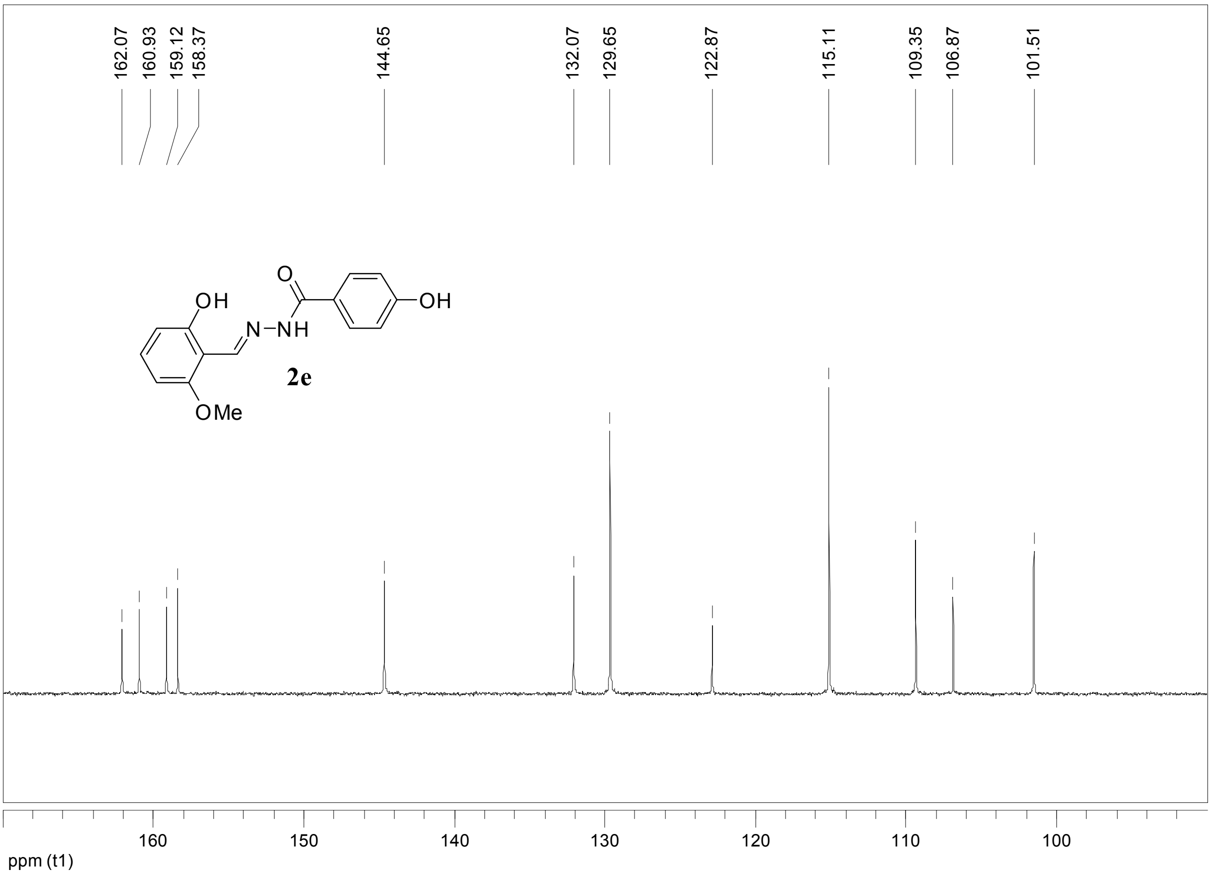

Figure S150. Expansion of ^{13}C-NMR (100 MHz, DMSO-d_6) spectrum of compound **2e**.

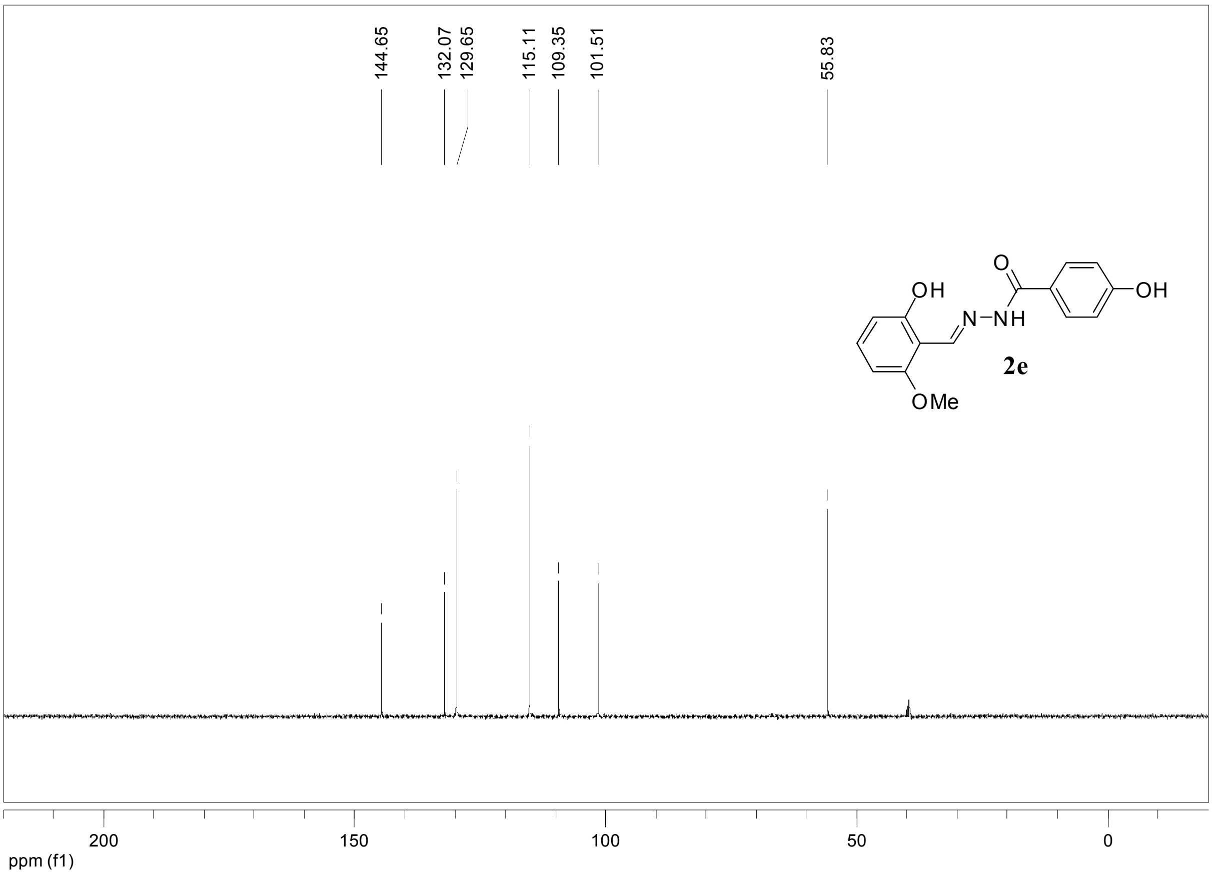

Figure S151. ^{13}C-NMR (100 MHz, DMSO-d_6) dept-135 experiment of compound **2e**.

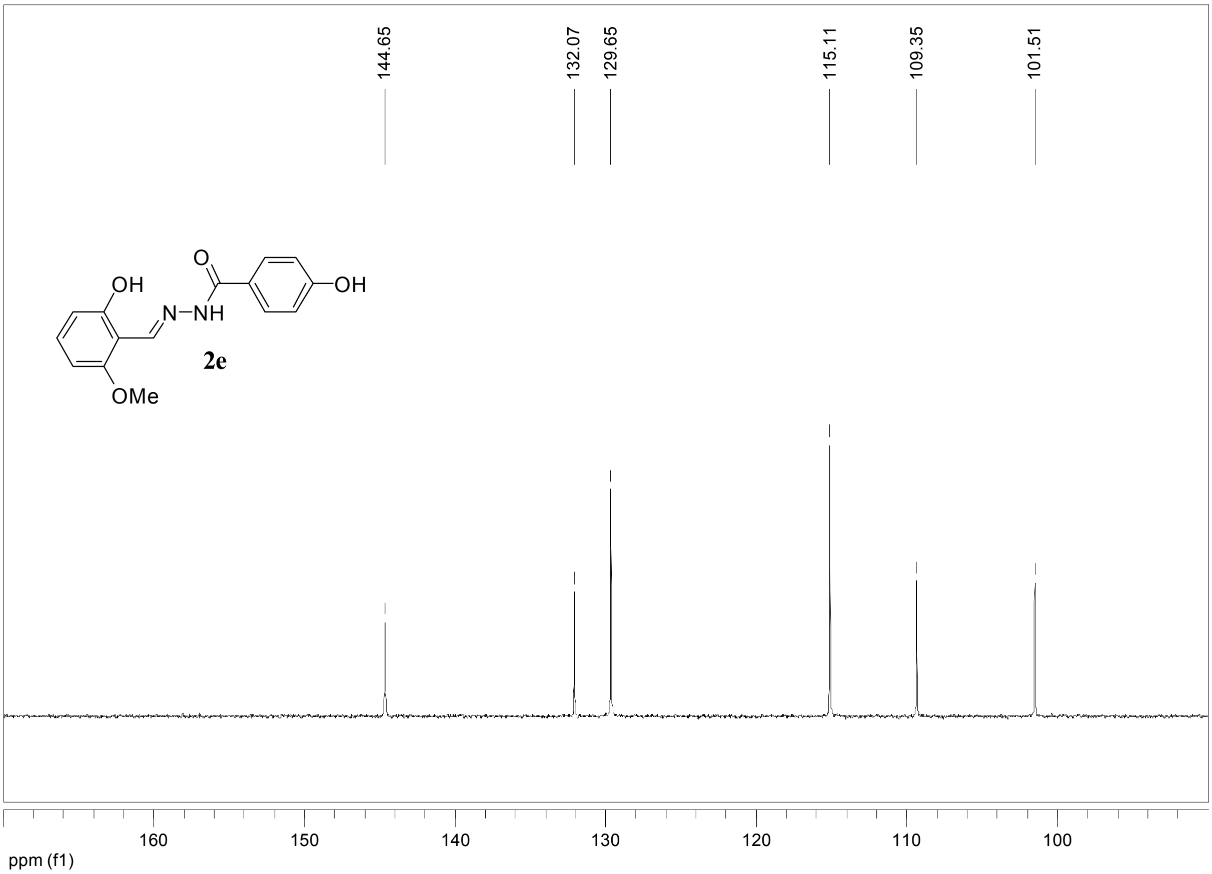

Figure S152. Expansion of ^{13}C-NMR (100 MHz, DMSO-d_6) dept-135 experiment of compound **2e**.

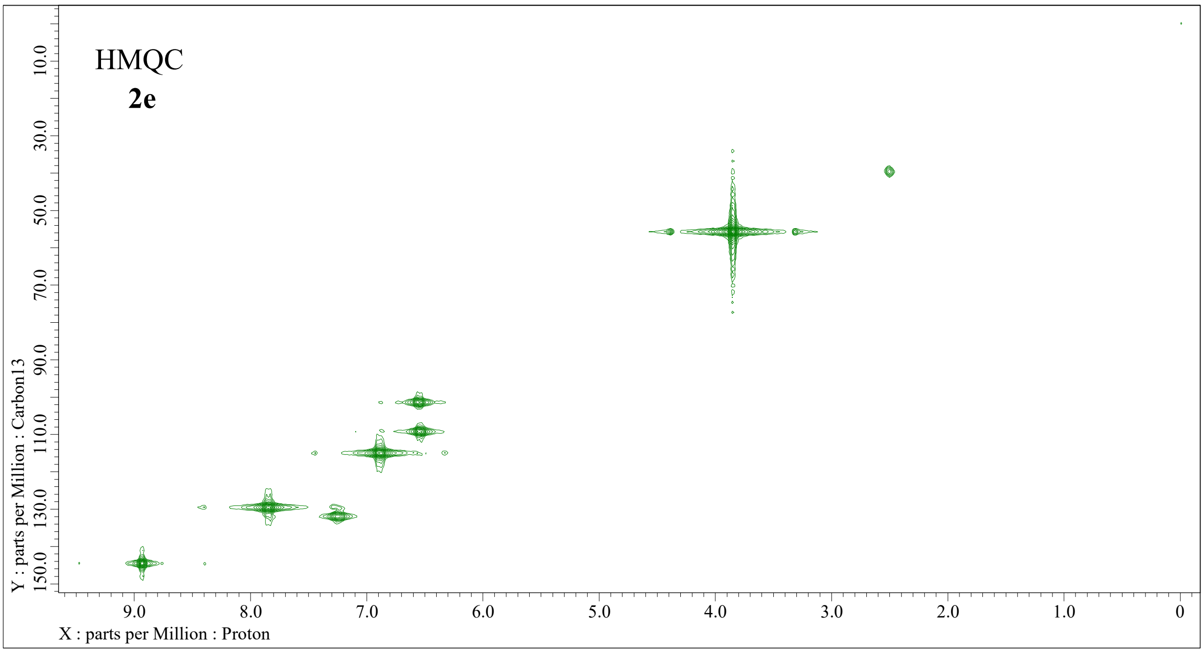

Figure S152. 2D-NMR (400 MHz, DMSO-d_6) HMQC experiment of 4-hydroxy-*N*'-[(*E*)-(6-methoxy-2-hydroxyphenyl)methylidene]benzohydrazide (**2e**).

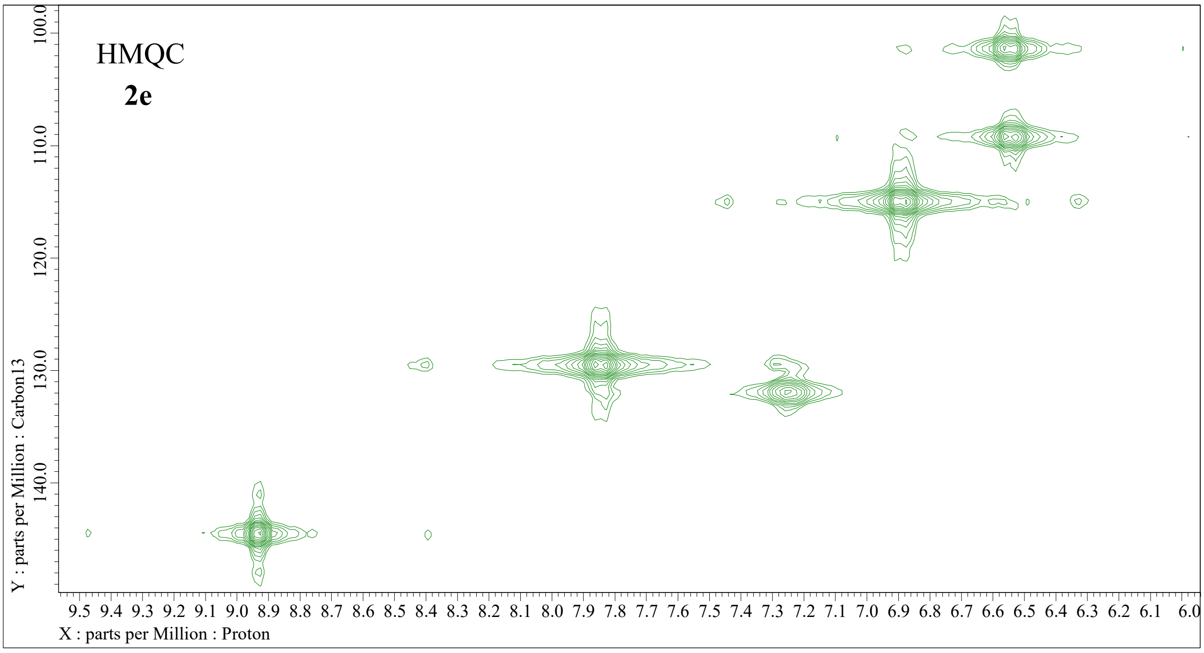

Figure S154. Expansion of 2D-NMR (400 MHz, DMSO-d_6) HMQC experiment of 4-hydroxy-*N*'-[(*E*)-(6-methoxy-2-hydroxyphenyl)methylidene]benzohydrazide (**2e**).

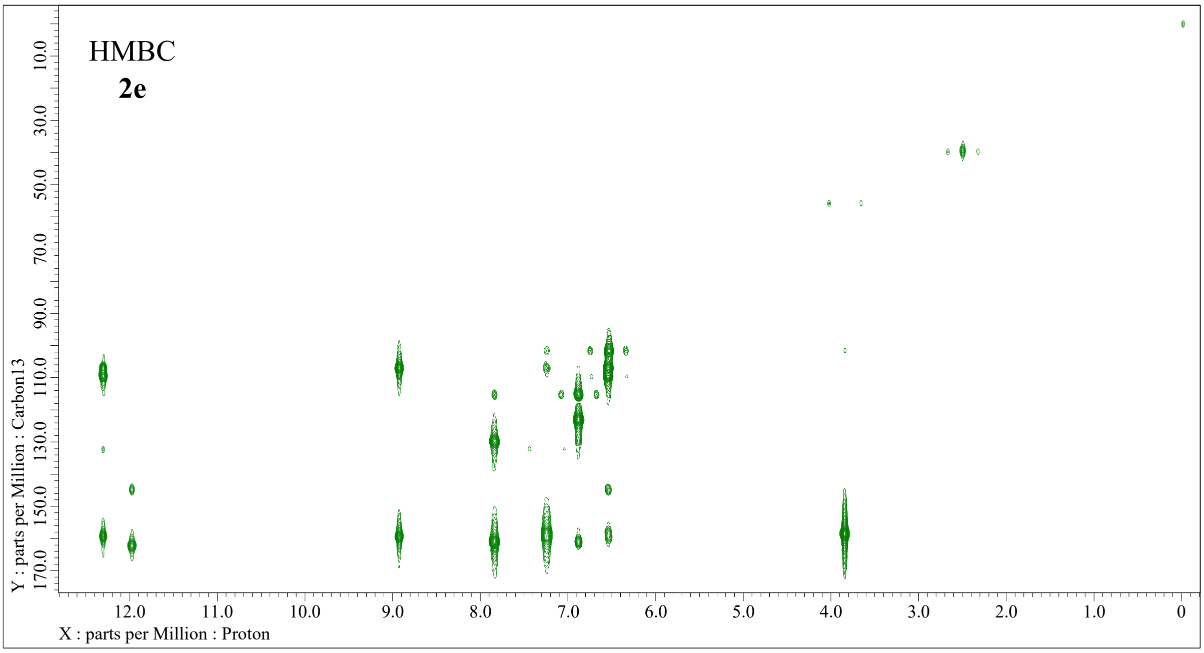

Figure S155. 2D-NMR (400 MHz, DMSO-d_6) HMBC experiment of 4-hydroxy-*N*'-[(*E*)-(6-methoxy-2-hydroxyphenyl)methylidene]benzohydrazide (**2e**).

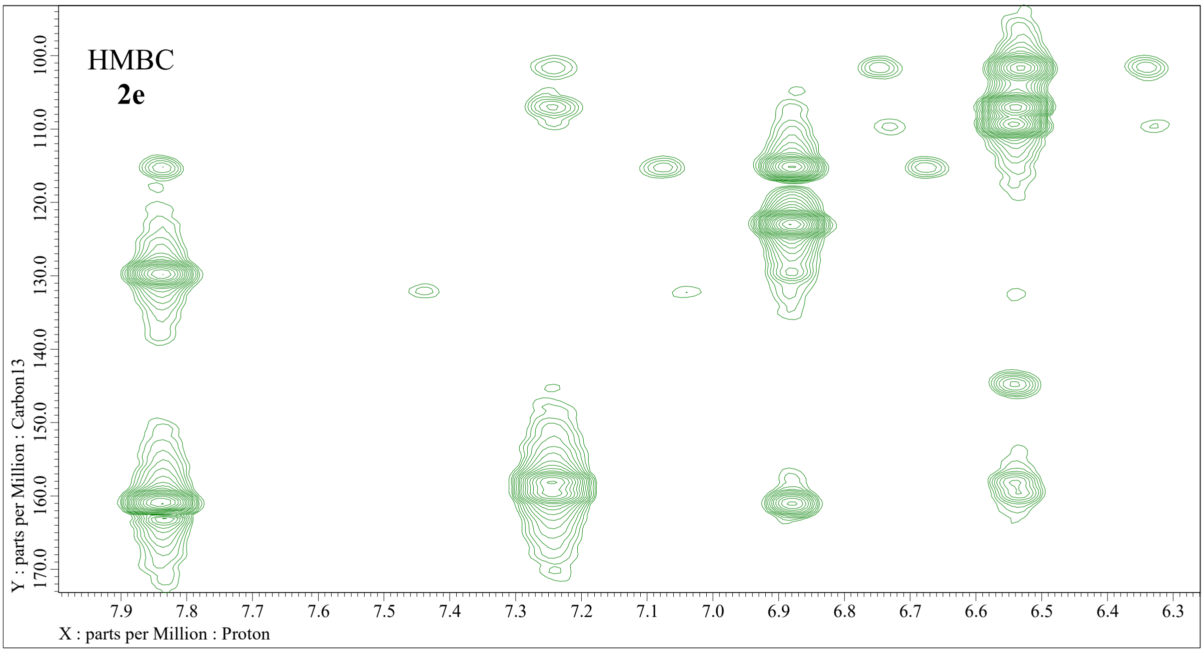

Figure S156. Expansion of 2D-NMR (400 MHz, DMSO-d_6) HMBC experiment of 4-hydroxy-*N*'-[(*E*)-(6-methoxy-2-hydroxyphenyl)methylidene]benzohydrazide (**2e**).

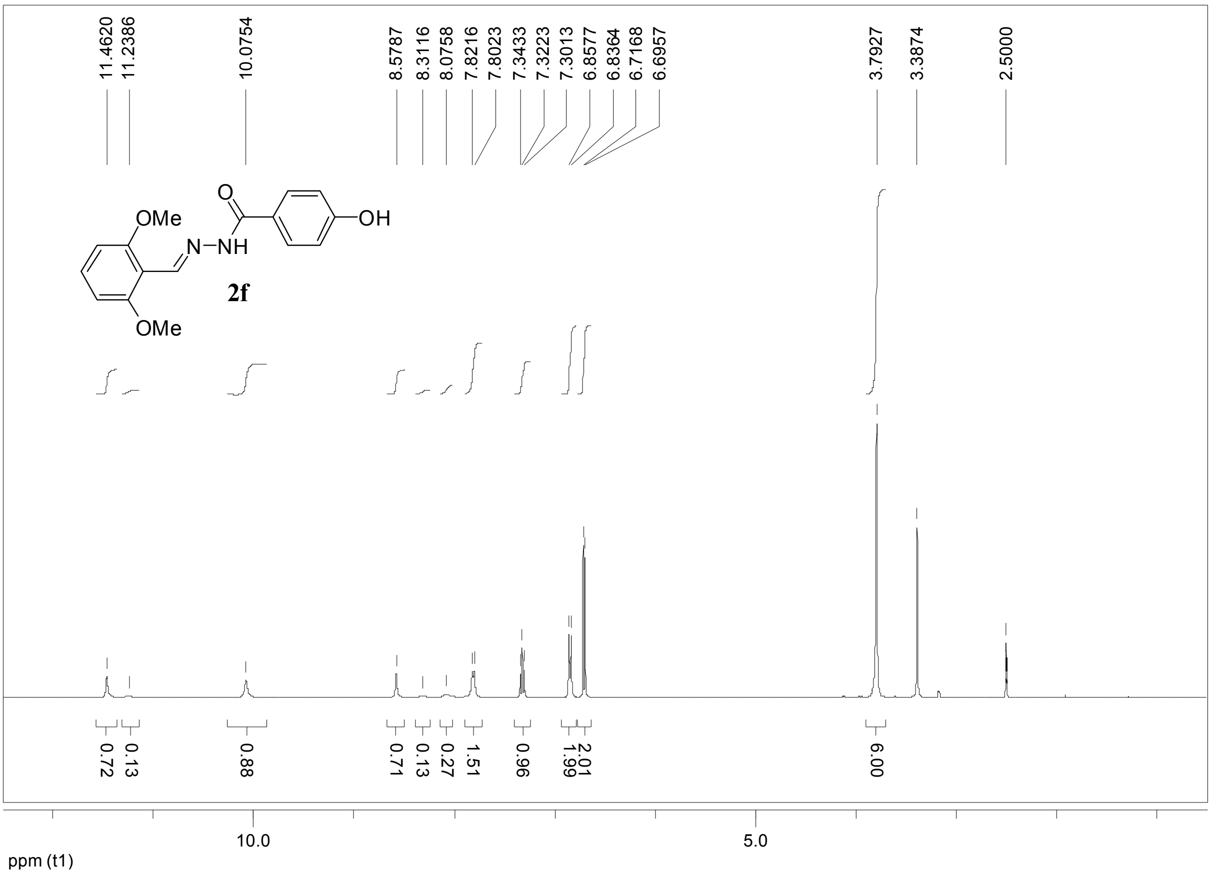

Figure S157. ^{1}H-NMR (400 MHz, DMSO-d_6) spectrum of analytical sample compound **2f**.

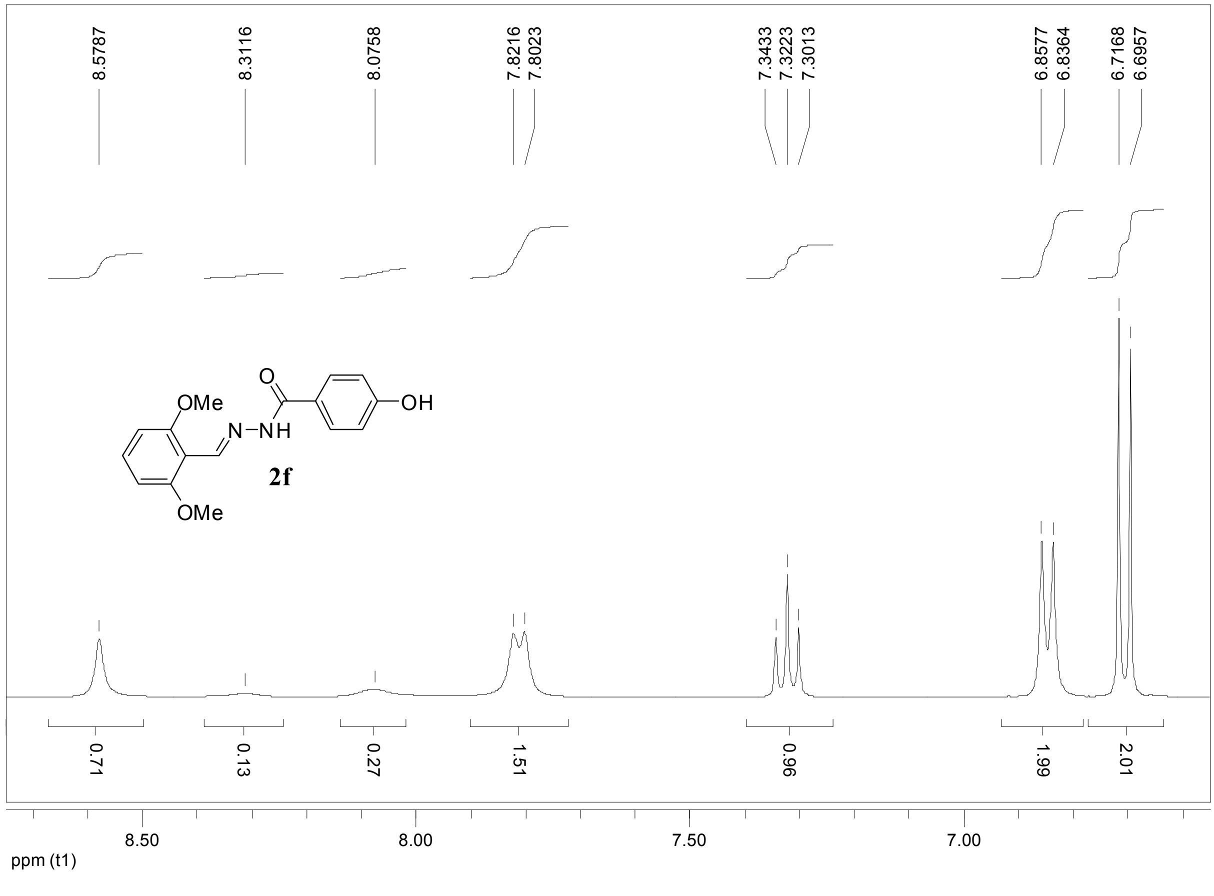

Figure S158. Expansion of ^{1}H-NMR (400 MHz, DMSO-d_6) spectrum of analytical sample compound **2f**.

Molecules **2020**, *25*, 1255; doi:10.3390/molecules25051255 www.mdpi.com/journal/molecules

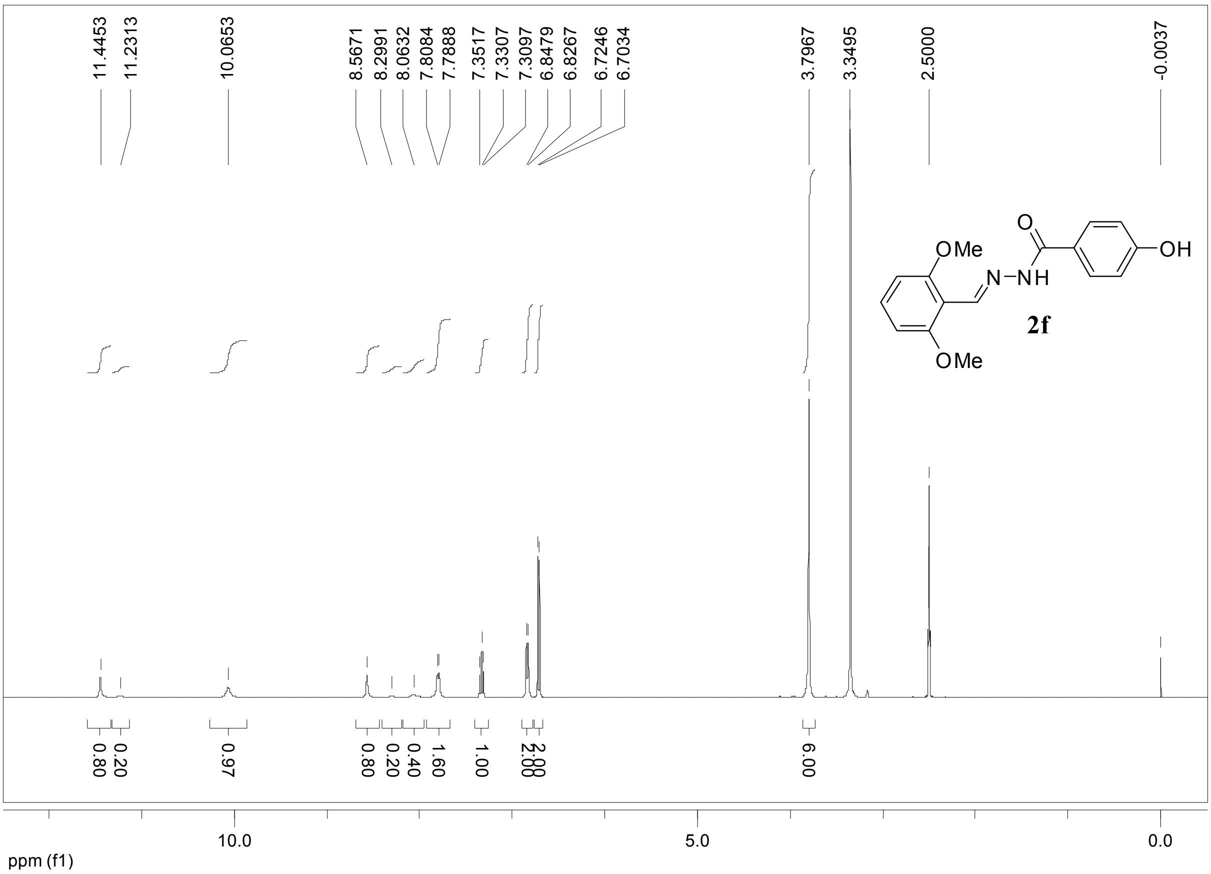

Figure S159. ^{1}H-NMR (400 MHz, DMSO-d_6) spectrum recrystallized compound **2f**.

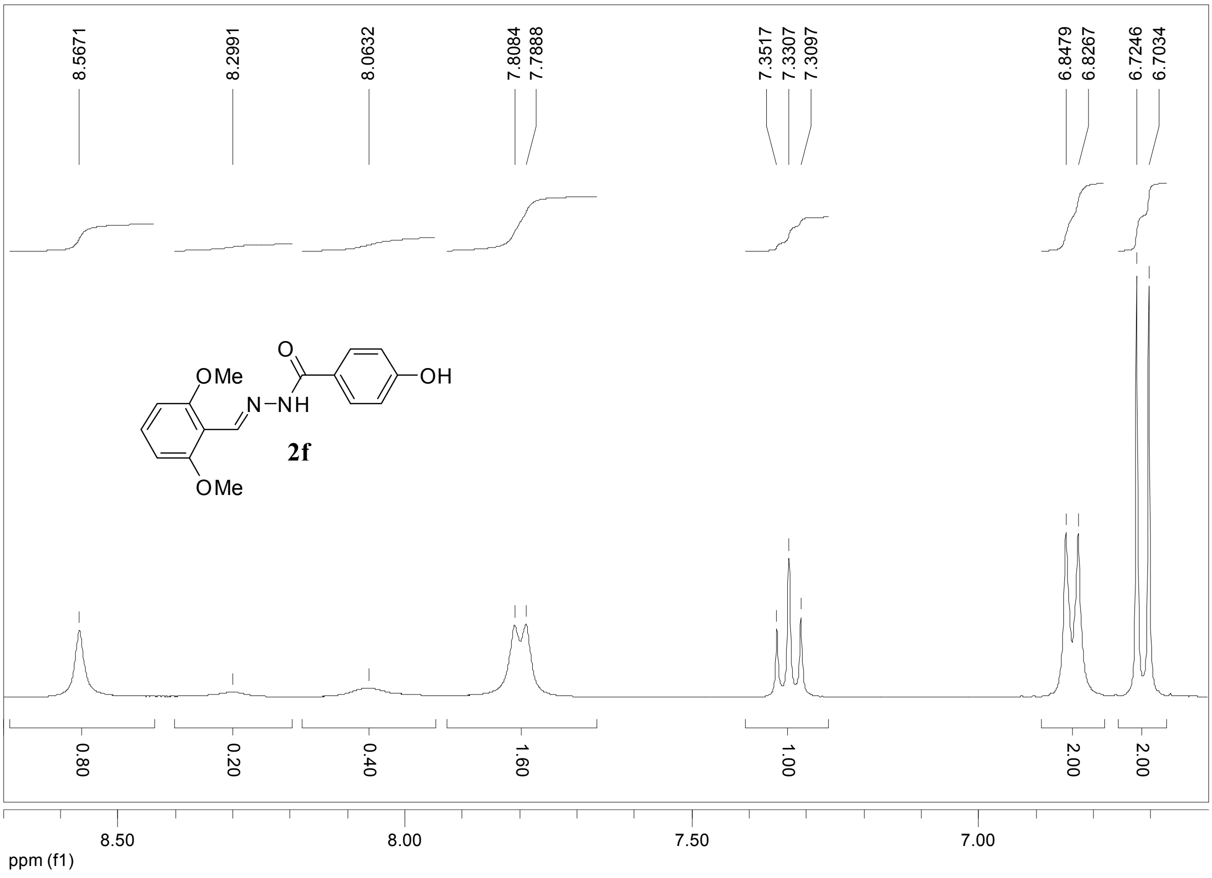

Figure S160. Expansion of ^{1}H-NMR (400 MHz, DMSO-d_6) spectrum recrystallized compound **2f**.

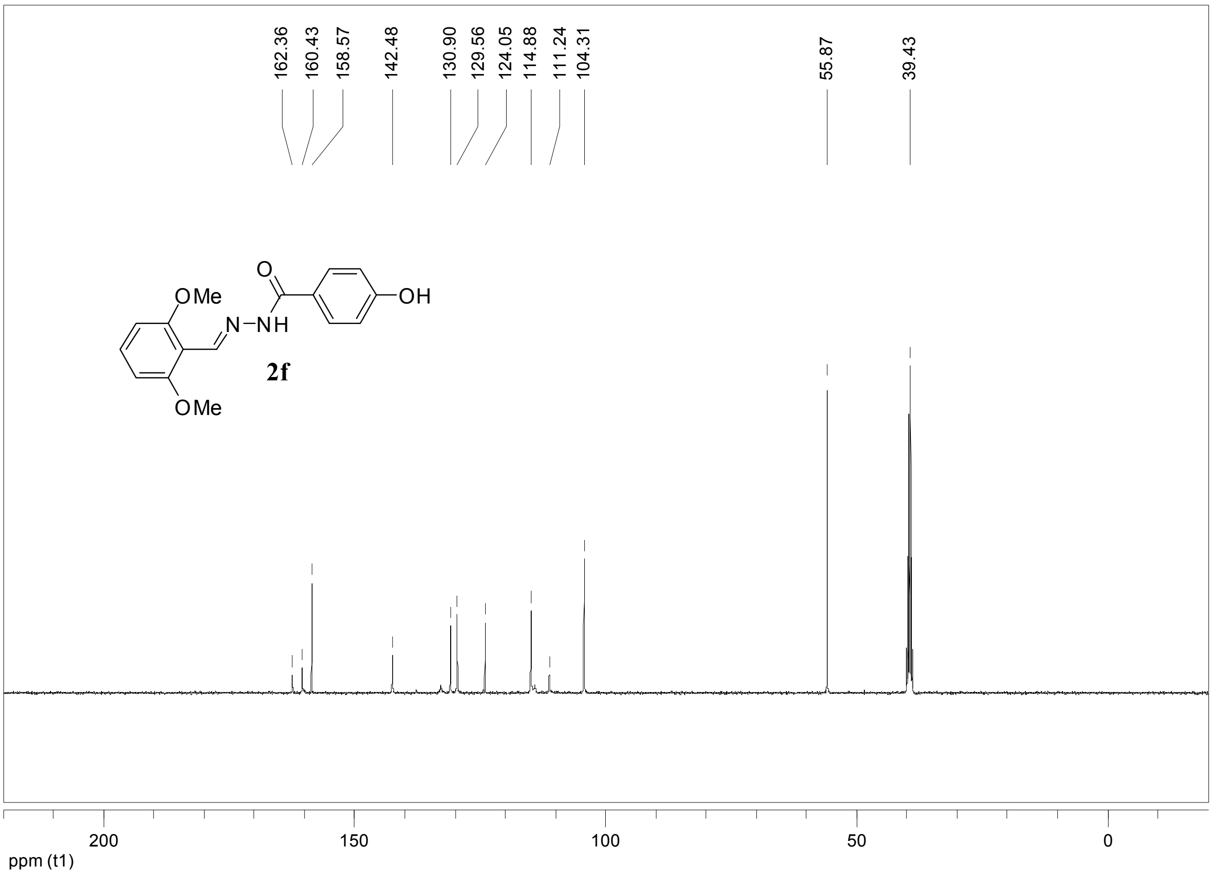

Figure S161. ^{13}C-NMR (100 MHz, DMSO-d_6) spectrum of compound **2f**.

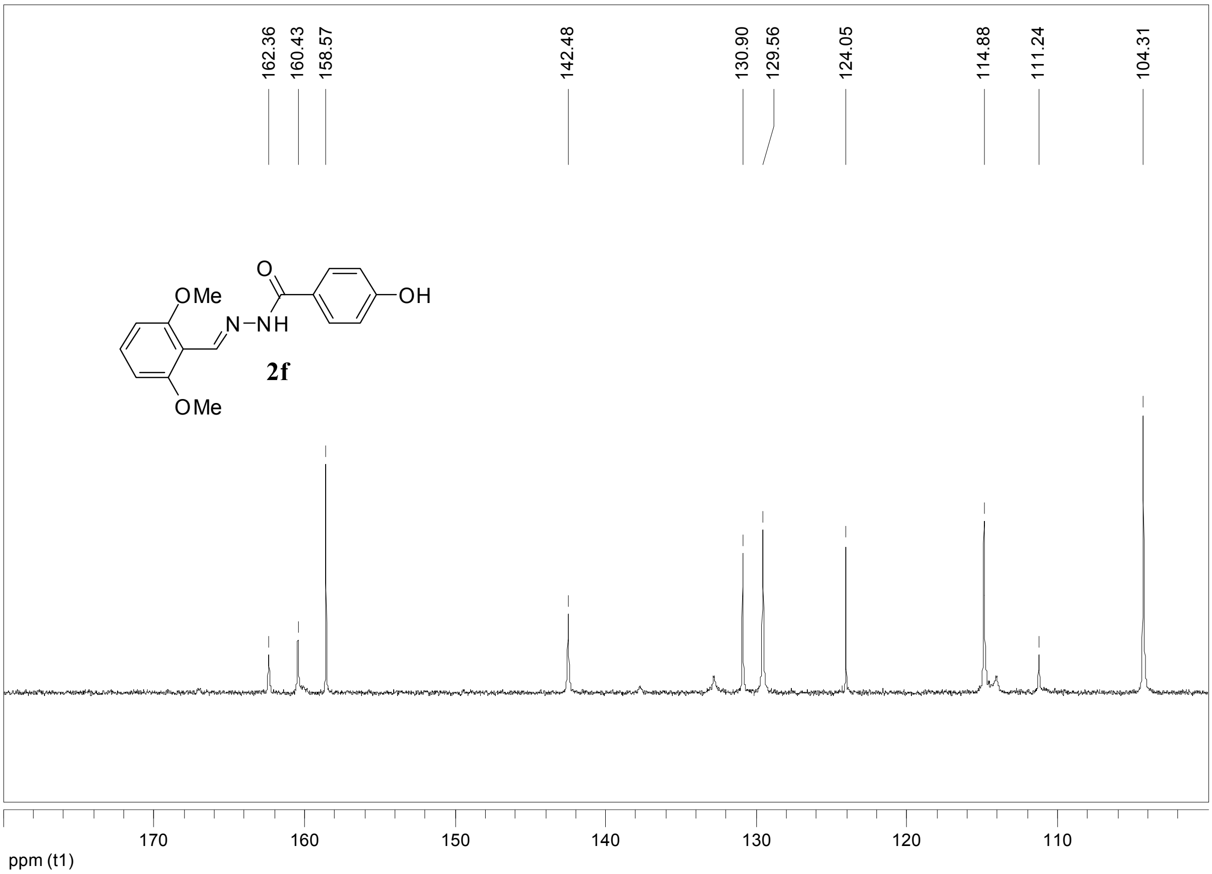

Figure S162. Expansion of ^{13}C-NMR (100 MHz, DMSO-d_6) spectrum of compound **2f**.

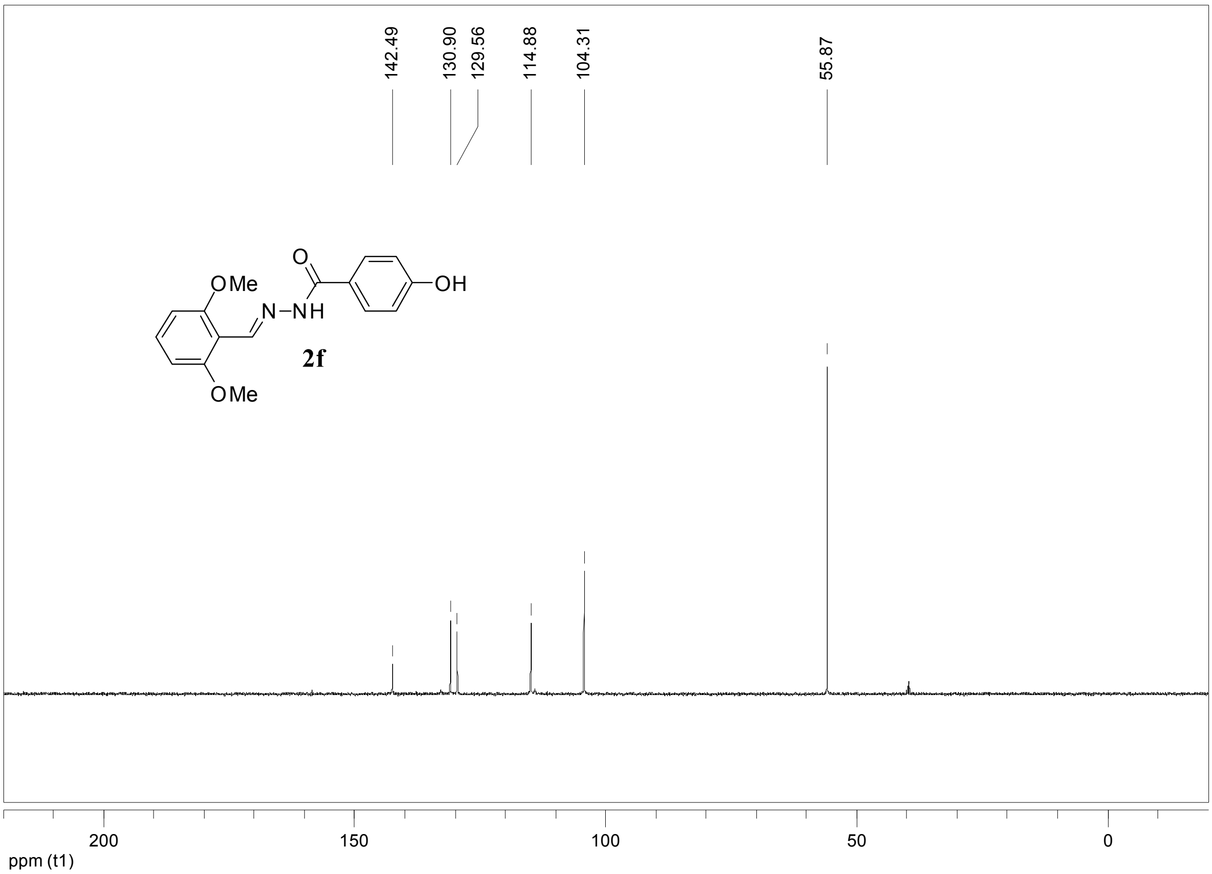

Figure S163. ^{13}C-NMR (100 MHz, DMSO-d_6) dept-135 experiment of compound **2f**.

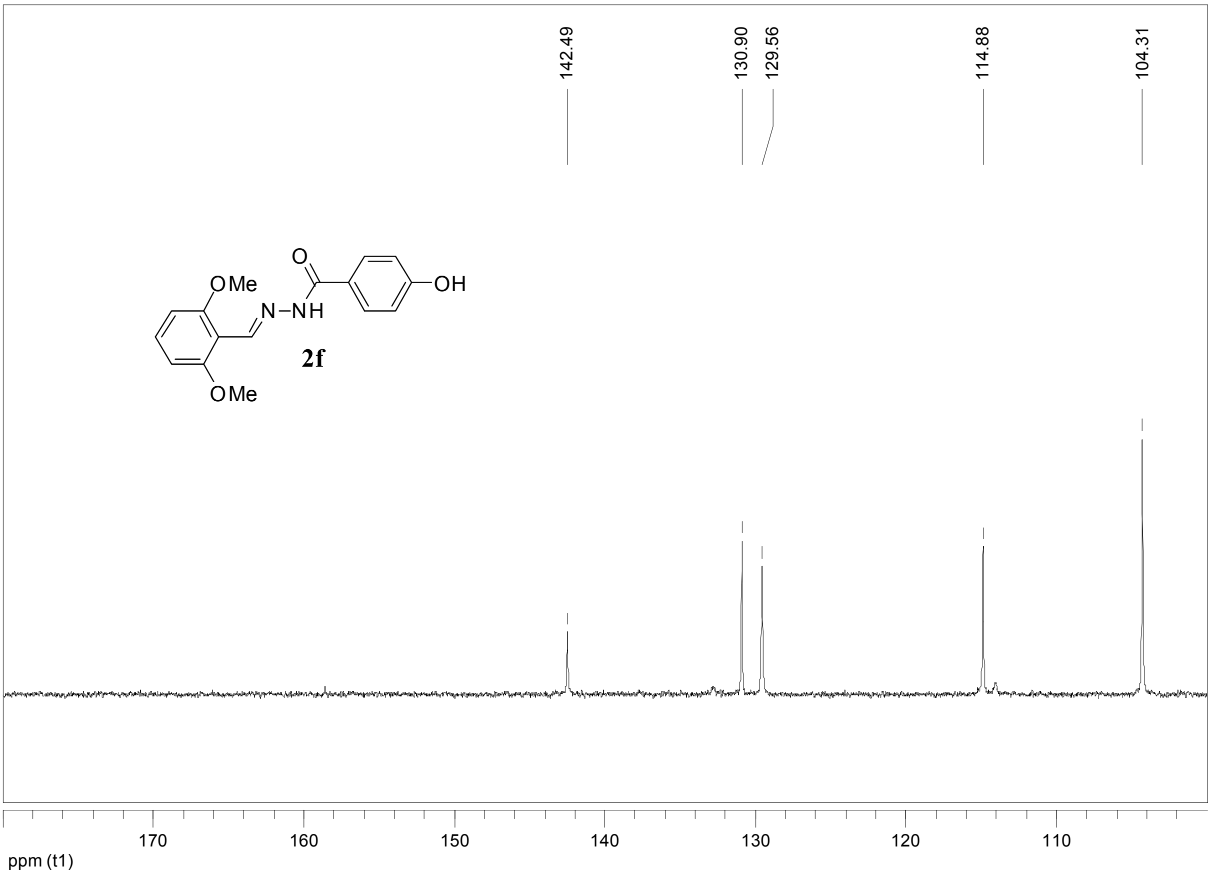

Figure S164. Expansion of ^{13}C-NMR (100 MHz, DMSO-d_6) dept-135 experiment of compound **2f**.

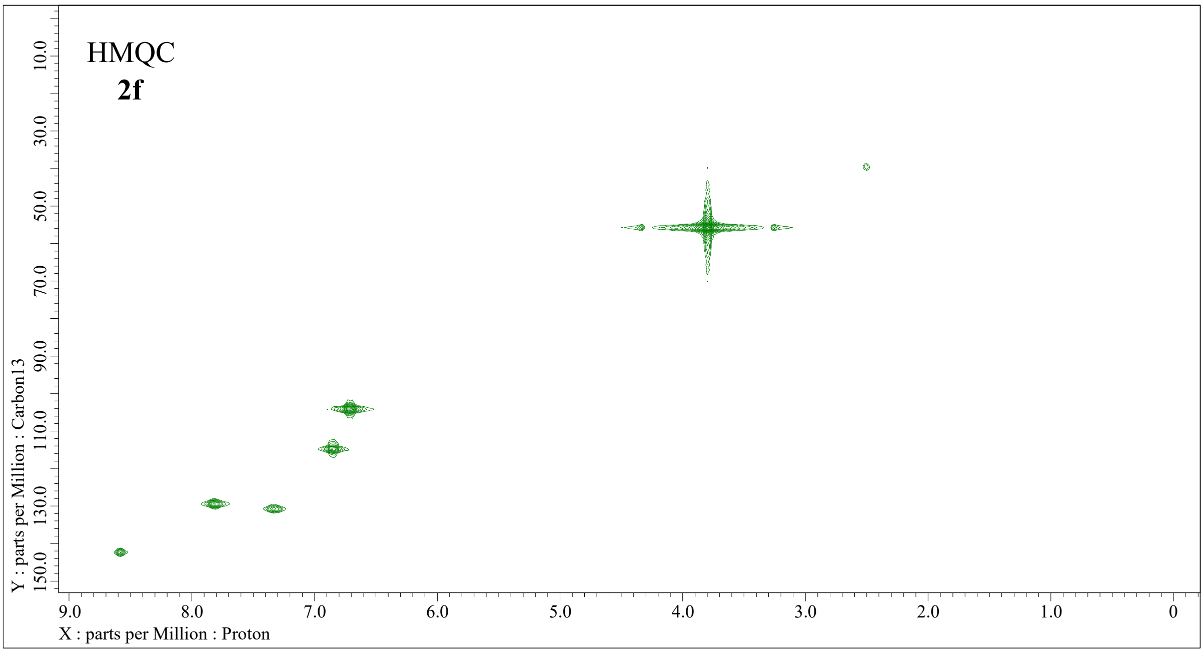

Figure S165. 2D-NMR (400 MHz, DMSO-d_6) HMQC experiment of 4-hydroxy-*N*'-[(2,6-dimethoxyphenyl)methylidene]benzohydrazide (**2f**).

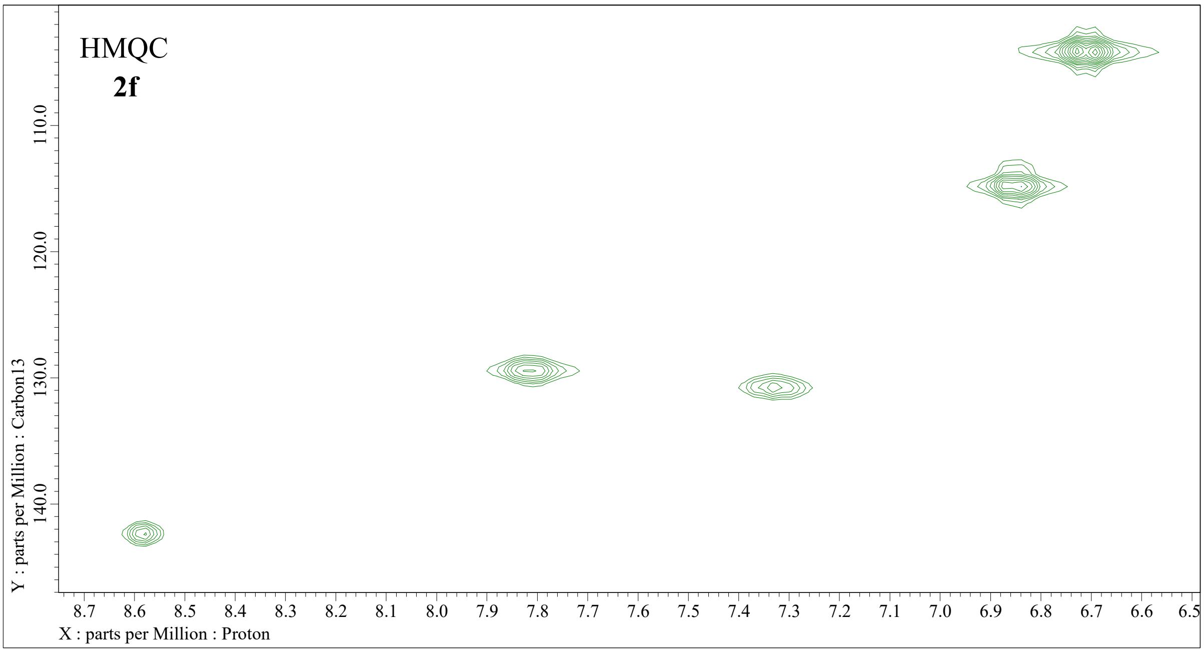

Figure S166. Expansion of 2D-NMR (400 MHz, DMSO-d_6) HMQC experiment of 4-hydroxy-*N*'-[(2,6-dimethoxyphenyl)methylidene]benzohydrazide (**2f**).

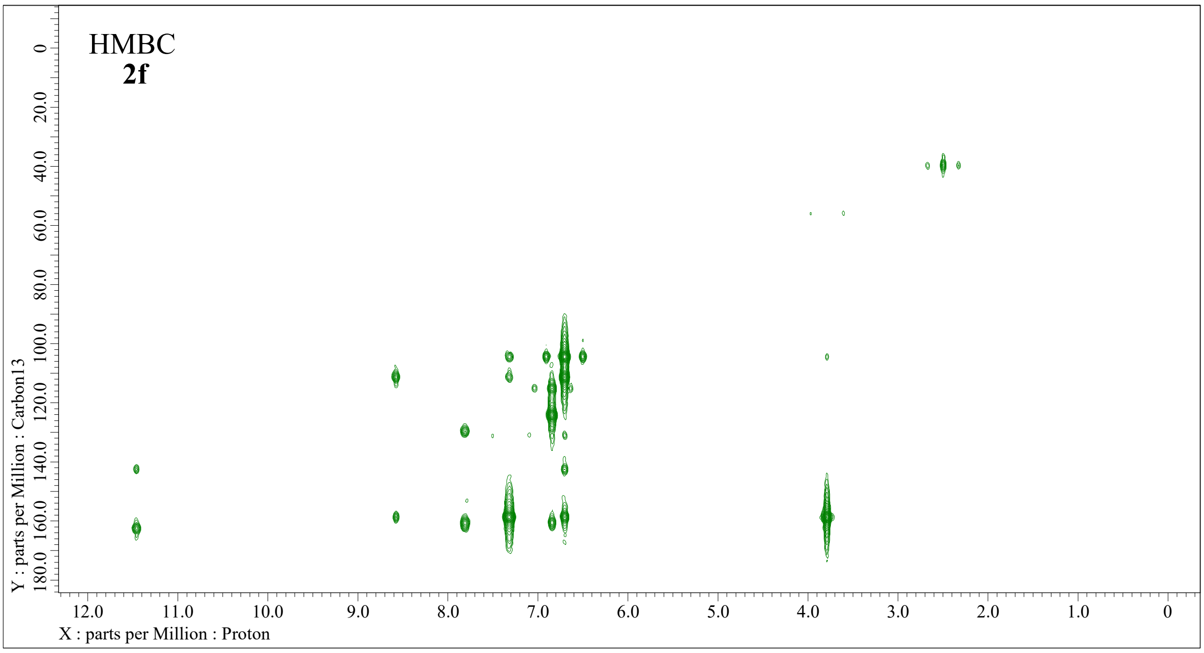

Figure S167. 2D-NMR (400 MHz, DMSO-d_6) HMBC experiment of 4-hydroxy-*N'*-[(2,6-dimethoxyphenyl)methylidene]benzohydrazide (**2f**).

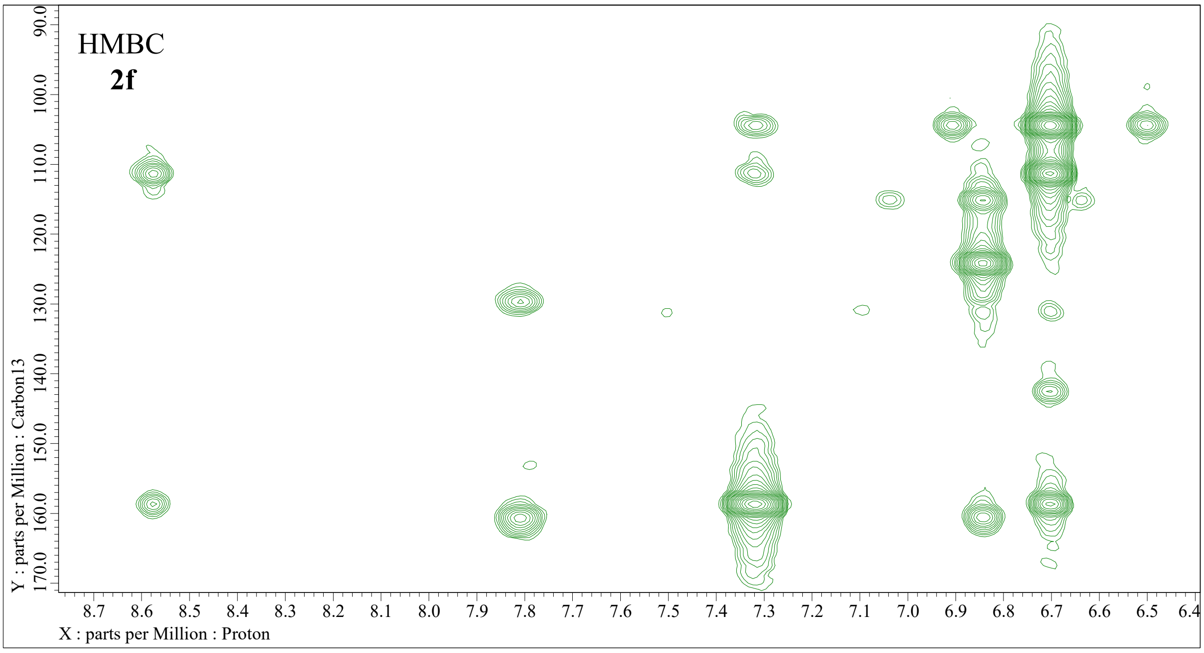

Figure S168. Expansion of 2D-NMR (400 MHz, DMSO-d_6) HMBC experiment of 4-hydroxy-*N'*-[(2,6-dimethoxyphenyl)methylidene]benzohydrazide (**2f**).

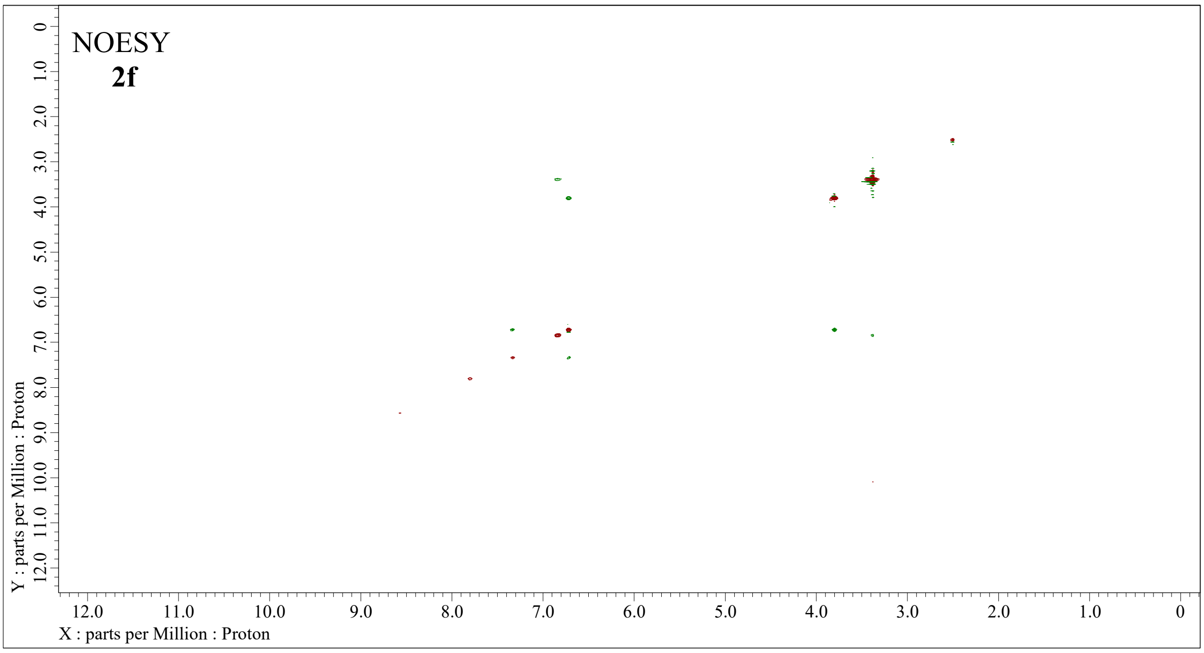

Figure S169. 2D-NMR (400 MHz, DMSO-d_6) NOESY experiment of 4-hydroxy-*N*'-[(2,6-dimethoxyphenyl)methylidene]benzohydrazide (**2f**).

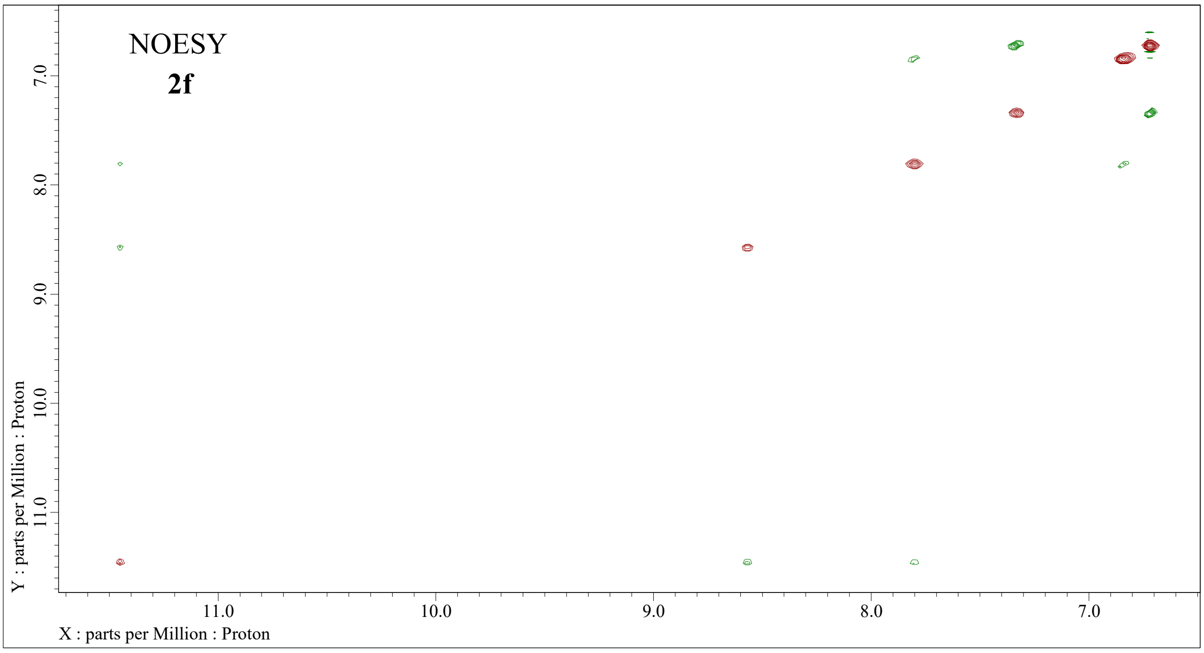

Figure S170. Expansion of 2D-NMR (400 MHz, DMSO-d_6) NOESY experiment of 4-hydroxy-*N*'-[(2,6-dimethoxyphenyl)methylidene]benzohydrazide (**2f**).

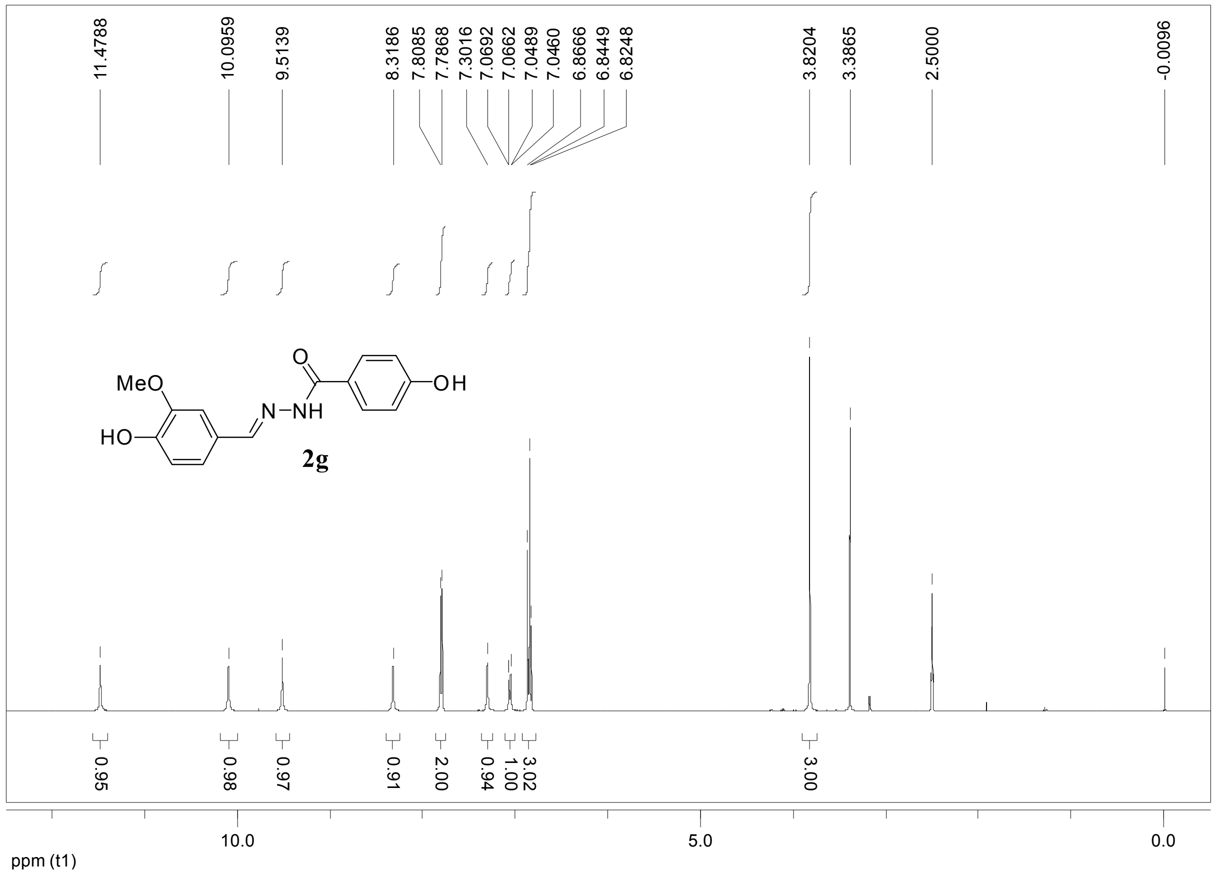

Figure S171. ^{1}H-NMR (400 MHz, DMSO-d_6) spectrum of compound **2g**.

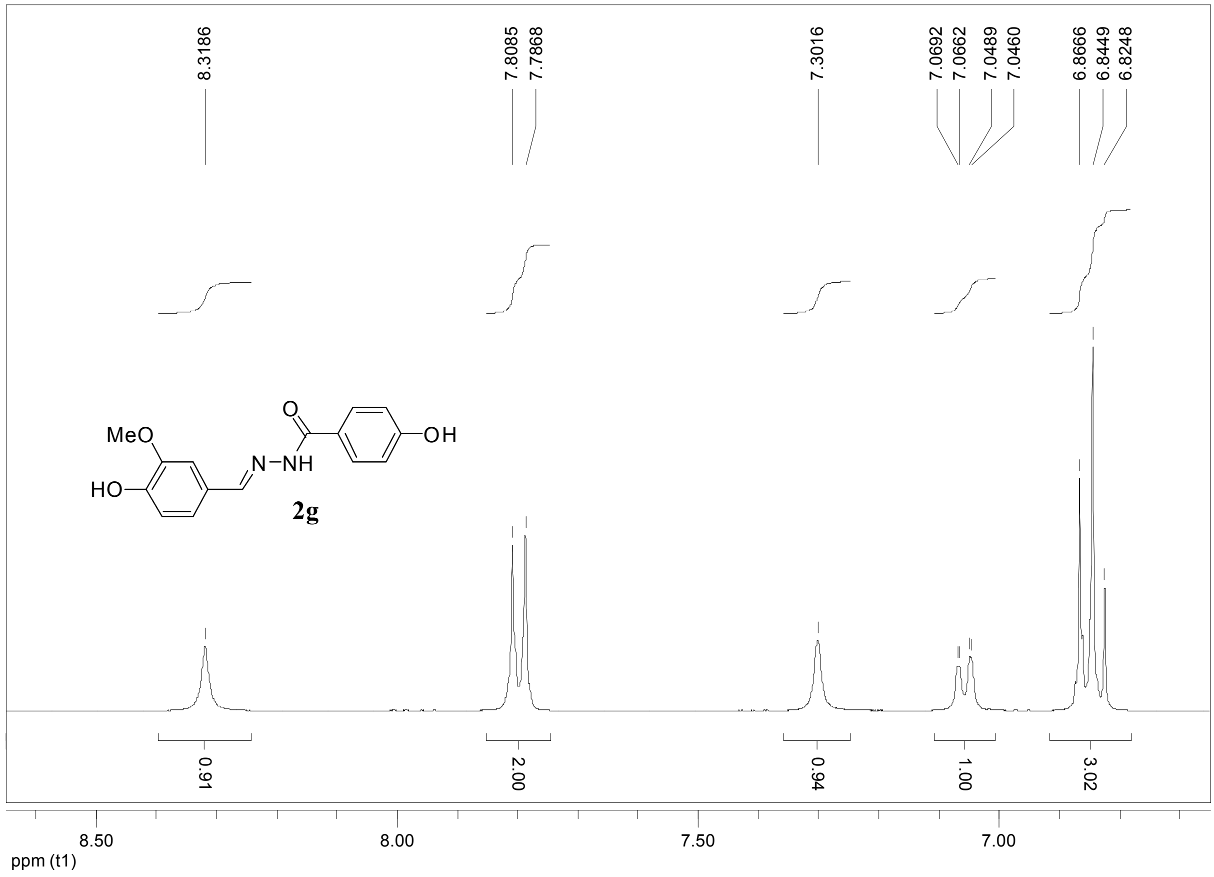

Figure S172. Expansion of ^{1}H-NMR (400 MHz, DMSO-d_6) spectrum of compound **2g**.

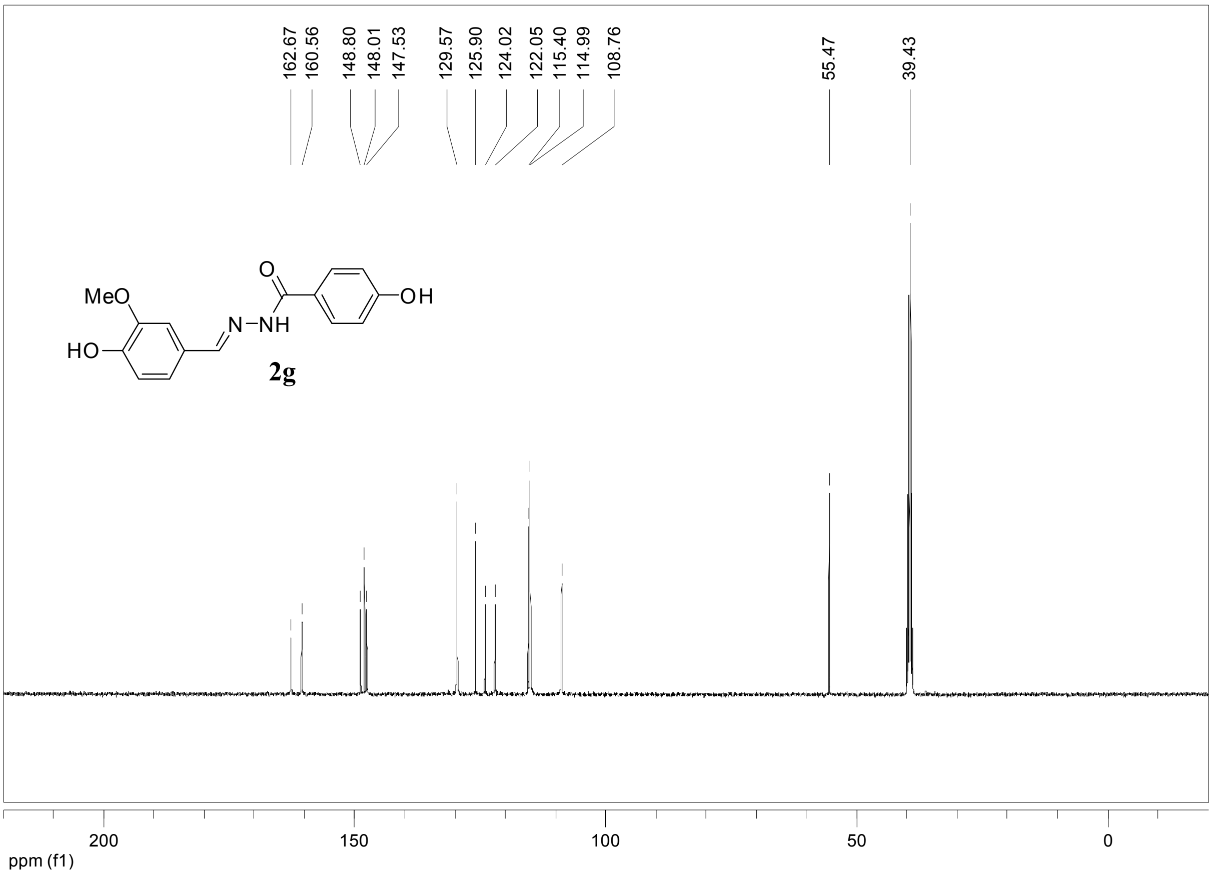

Figure S173. ^{13}C-NMR (100 MHz, DMSO-d_6) spectrum of compound **2g**.

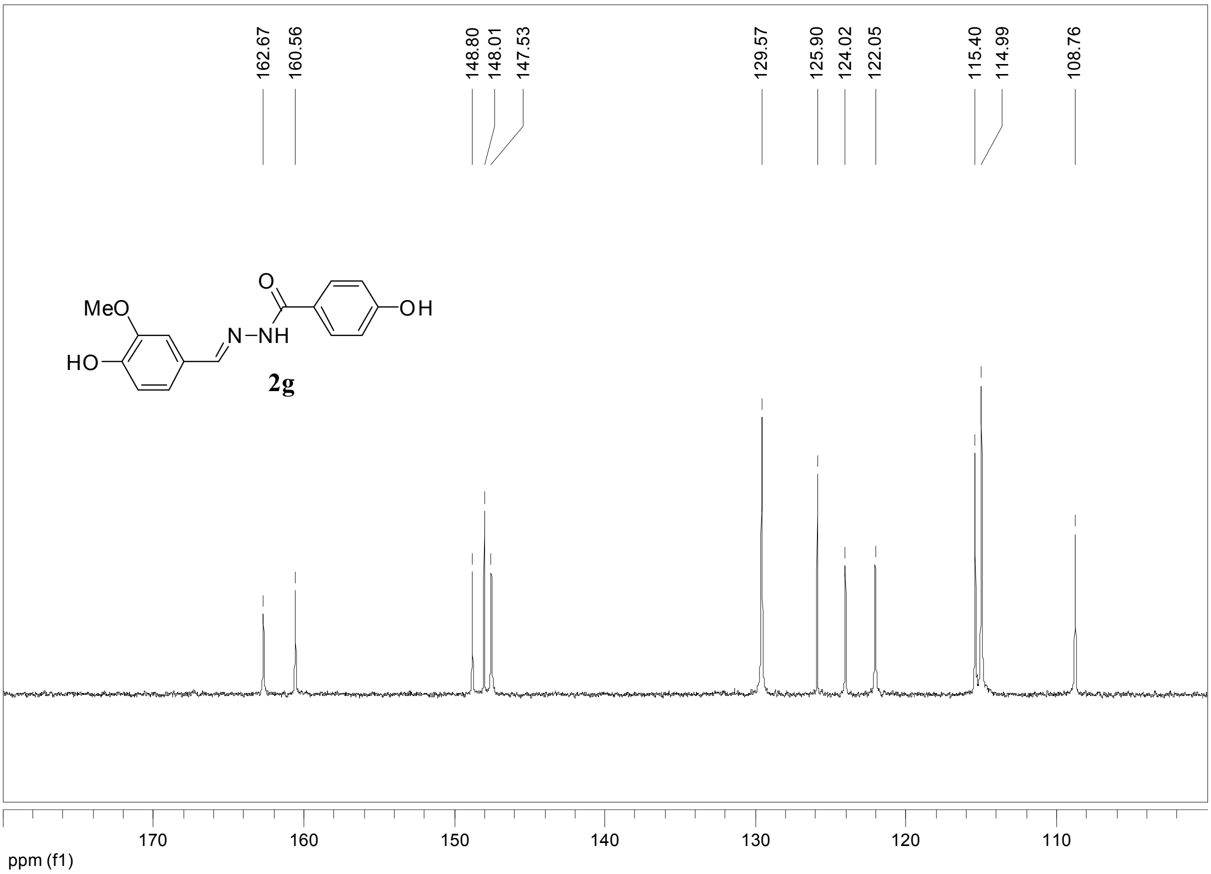

Figure S174. Expansion of ^{13}C-NMR (100 MHz, DMSO-d_6) spectrum of compound **2g**.

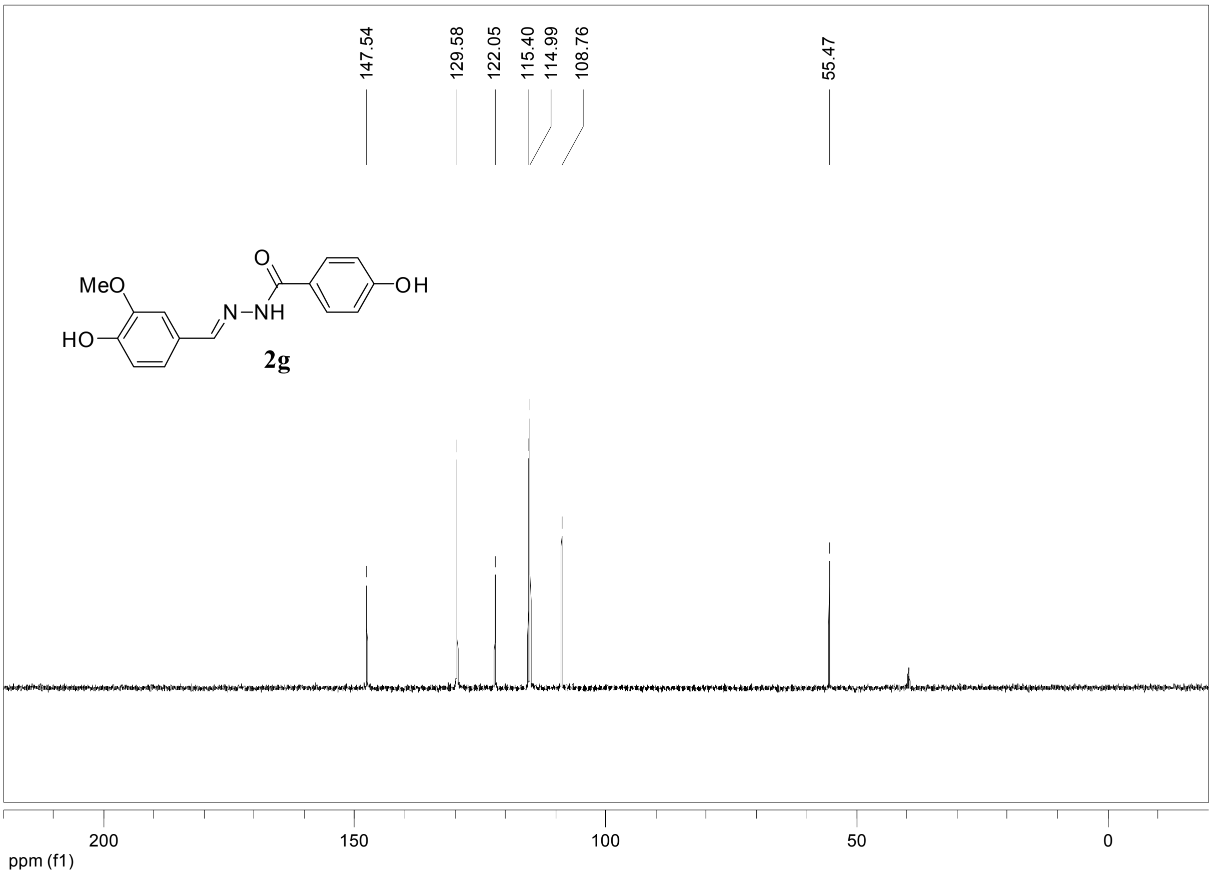

Figure S175. ^{13}C-NMR (100 MHz, DMSO-d_6) dept-135 experiment of compound **2g**.

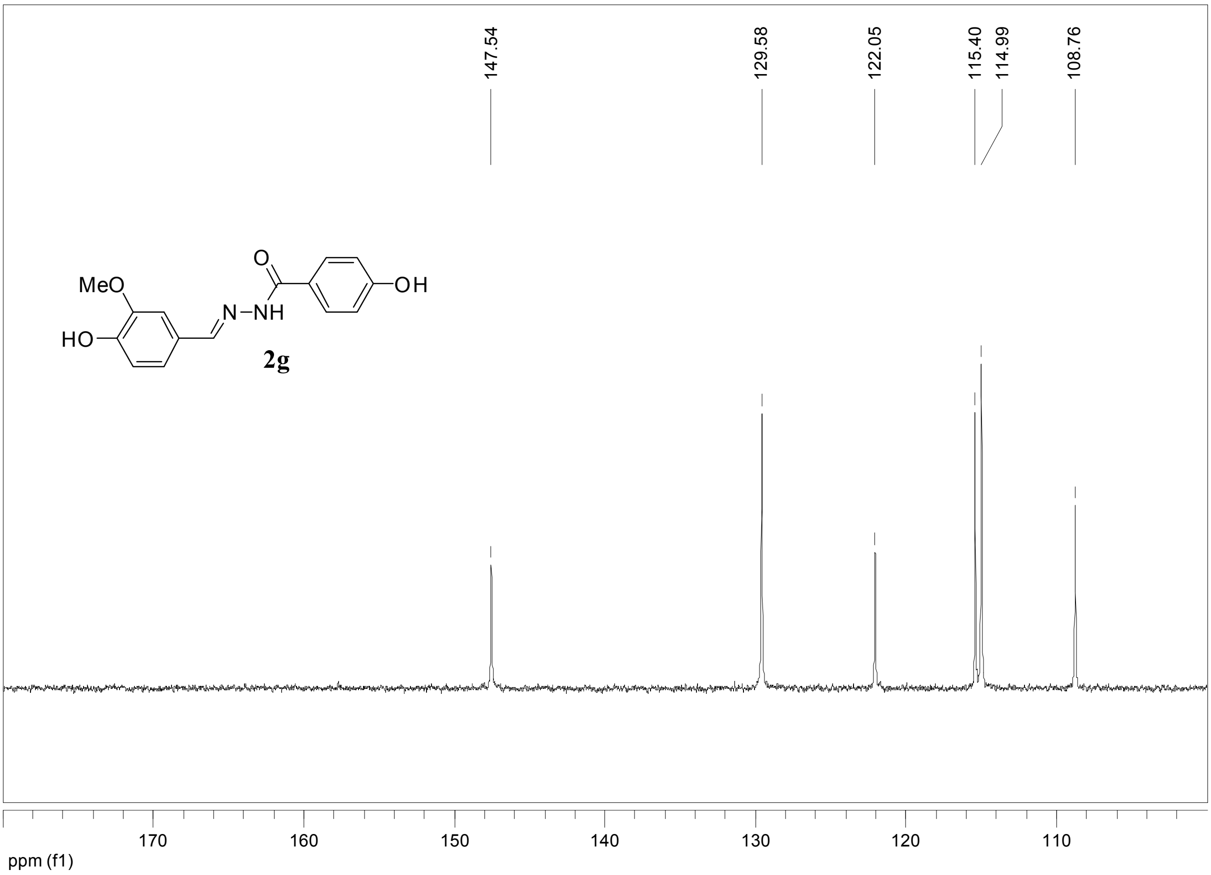

Figure S176. Expansion of ^{13}C-NMR (100 MHz, DMSO-d_6) dept-135 experiment of compound **2g**.

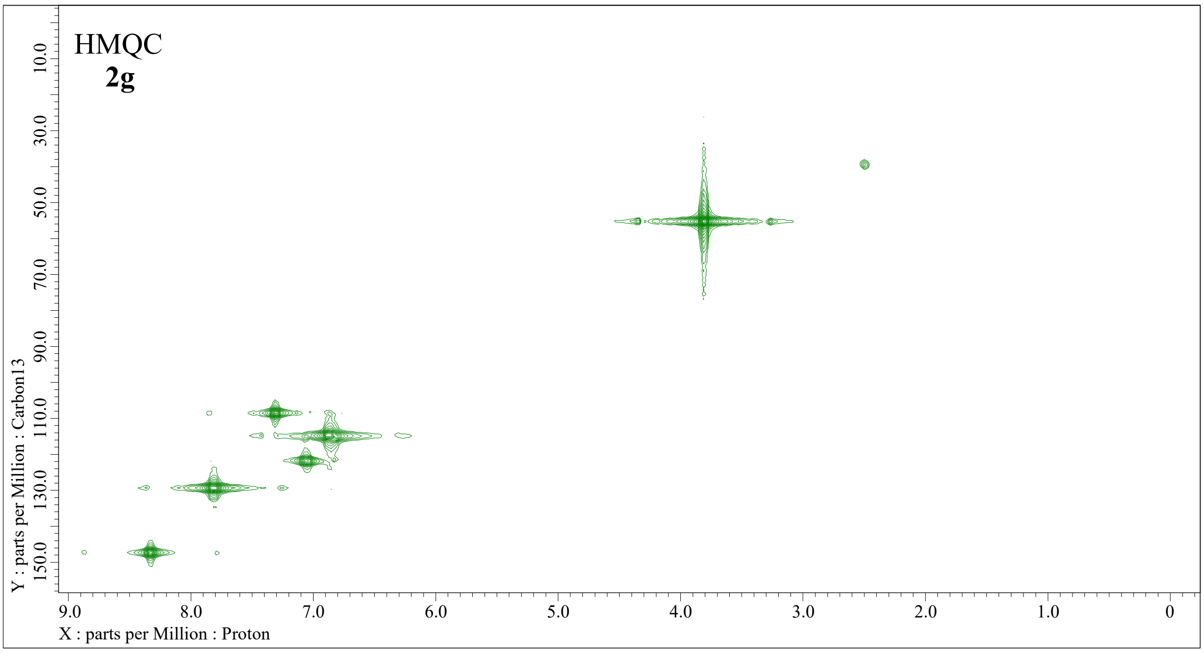

Figure S177. 2D-NMR (400 MHz, DMSO-d_6) HMQC experiment of 4-hydroxy-*N*'-[(*E*)-(4-hydroxy-3-methoxyphenyl)methylidene]benzohydrazide (**2g**).

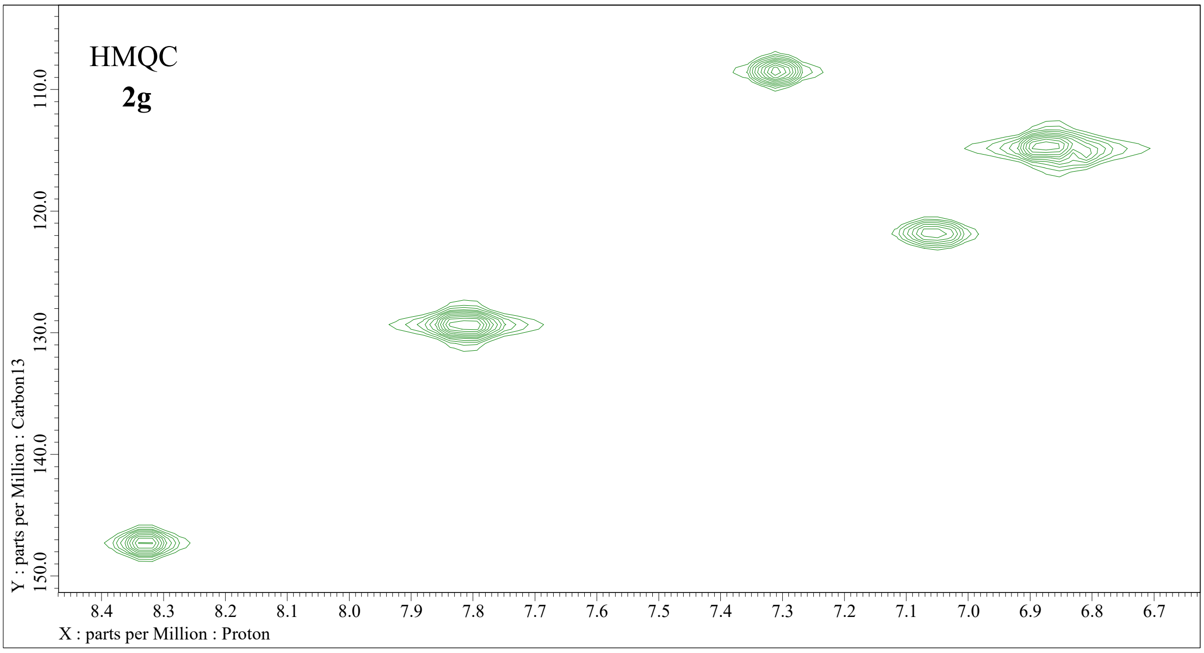

Figure S178. Expansion of 2D-NMR (400 MHz, DMSO-d_6) HMQC experiment of 4-hydroxy-*N*'-[(*E*)-(4-hydroxy-3-methoxyphenyl)methylidene]benzohydrazide (**2g**).

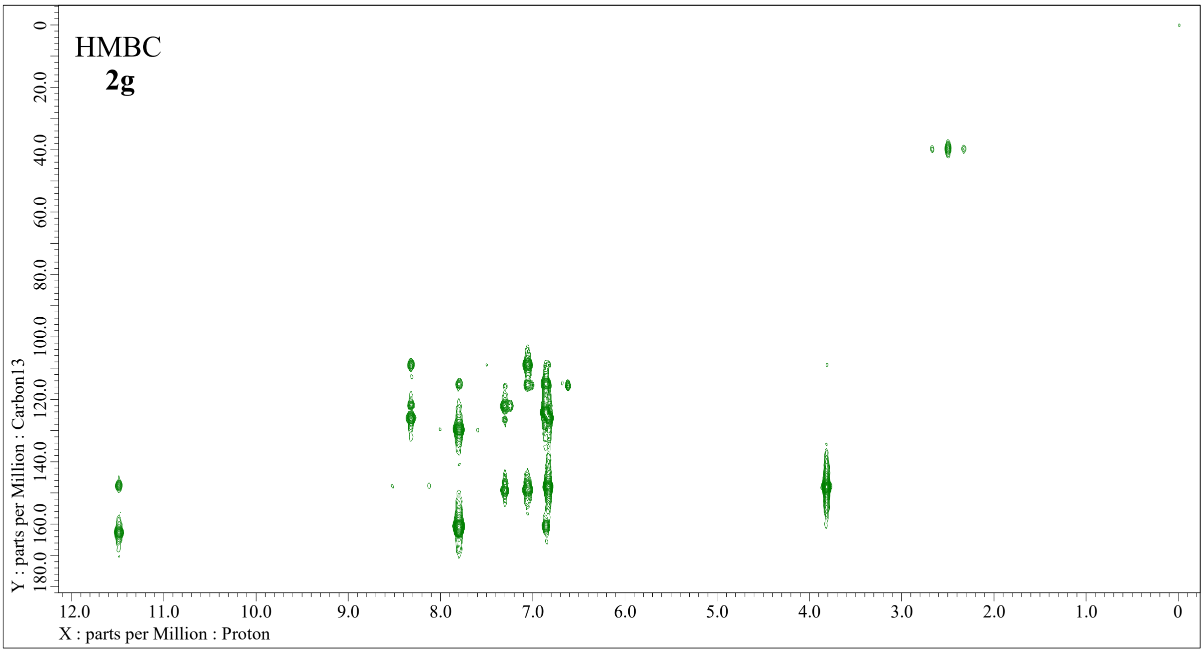

Figure S179. 2D-NMR (400 MHz, DMSO-d_6) HMBC experiment of 4-hydroxy-*N*'-[(*E*)-(4-hydroxy-3-methoxyphenyl)methylidene]benzohydrazide (**2g**).

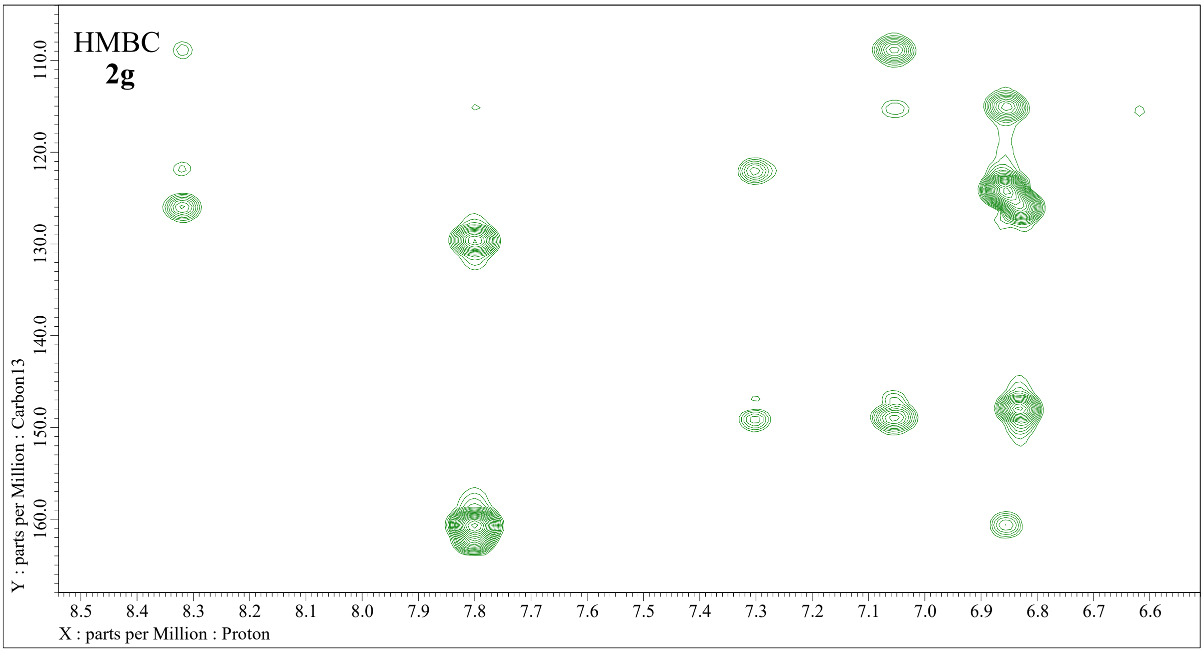

Figure S180. Expansion of 2D-NMR (400 MHz, DMSO-d_6) HMBC experiment of 4-hydroxy-*N*'-[(*E*)-(4-hydroxy-3-methoxyphenyl)methylidene]benzohydrazide (**2g**).

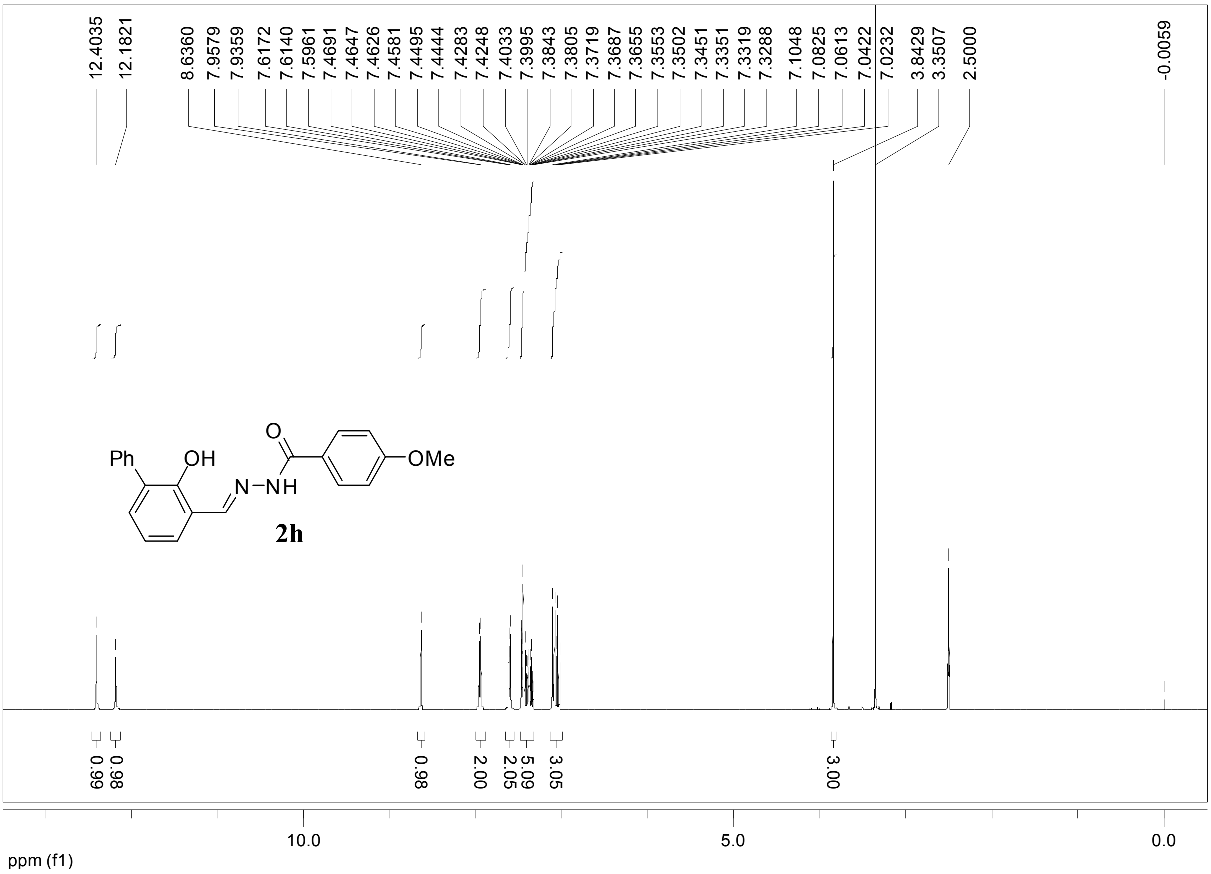

Figure S181. ¹H-NMR (400 MHz, DMSO-d_6) spectrum of compound **2h**.

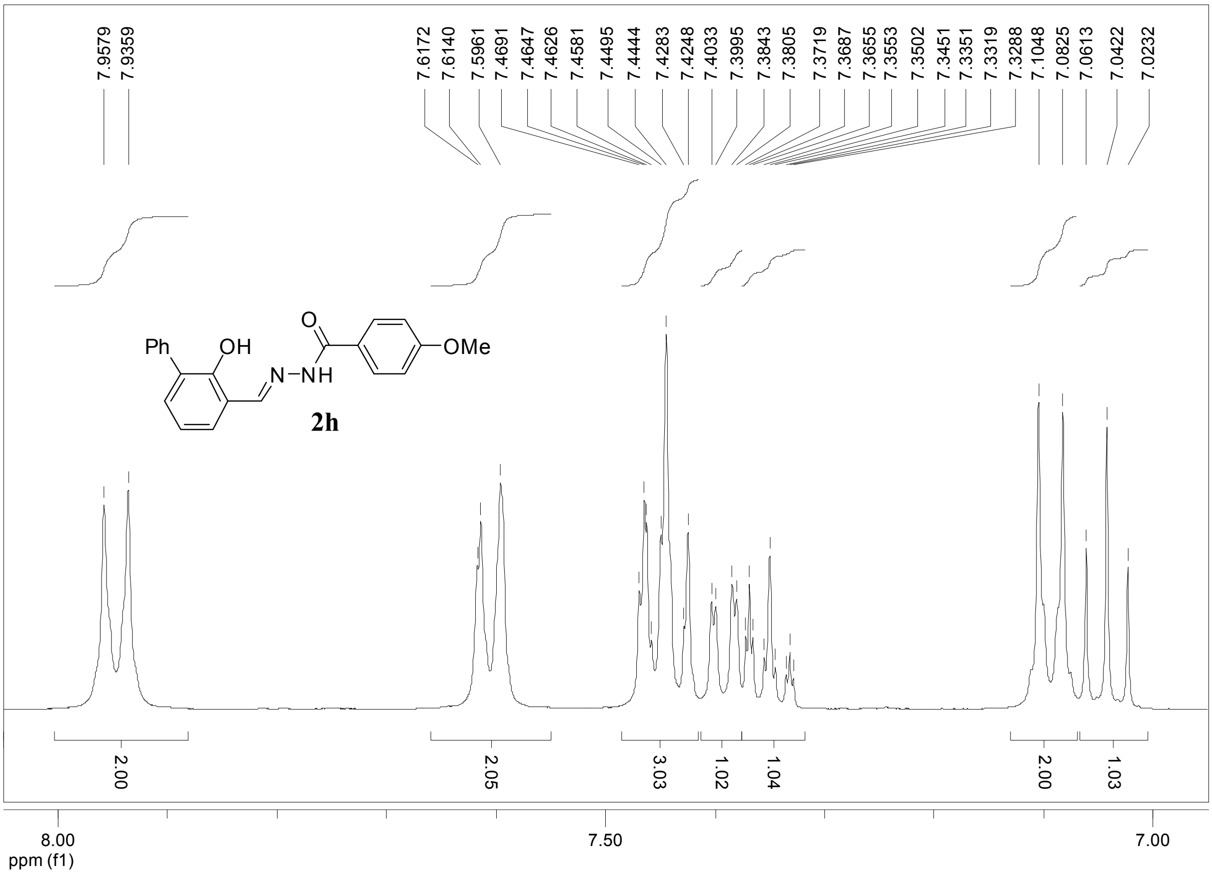

Figure S182. Expansion of ¹H-NMR (400 MHz, DMSO-d_6) spectrum of compound **2h**.

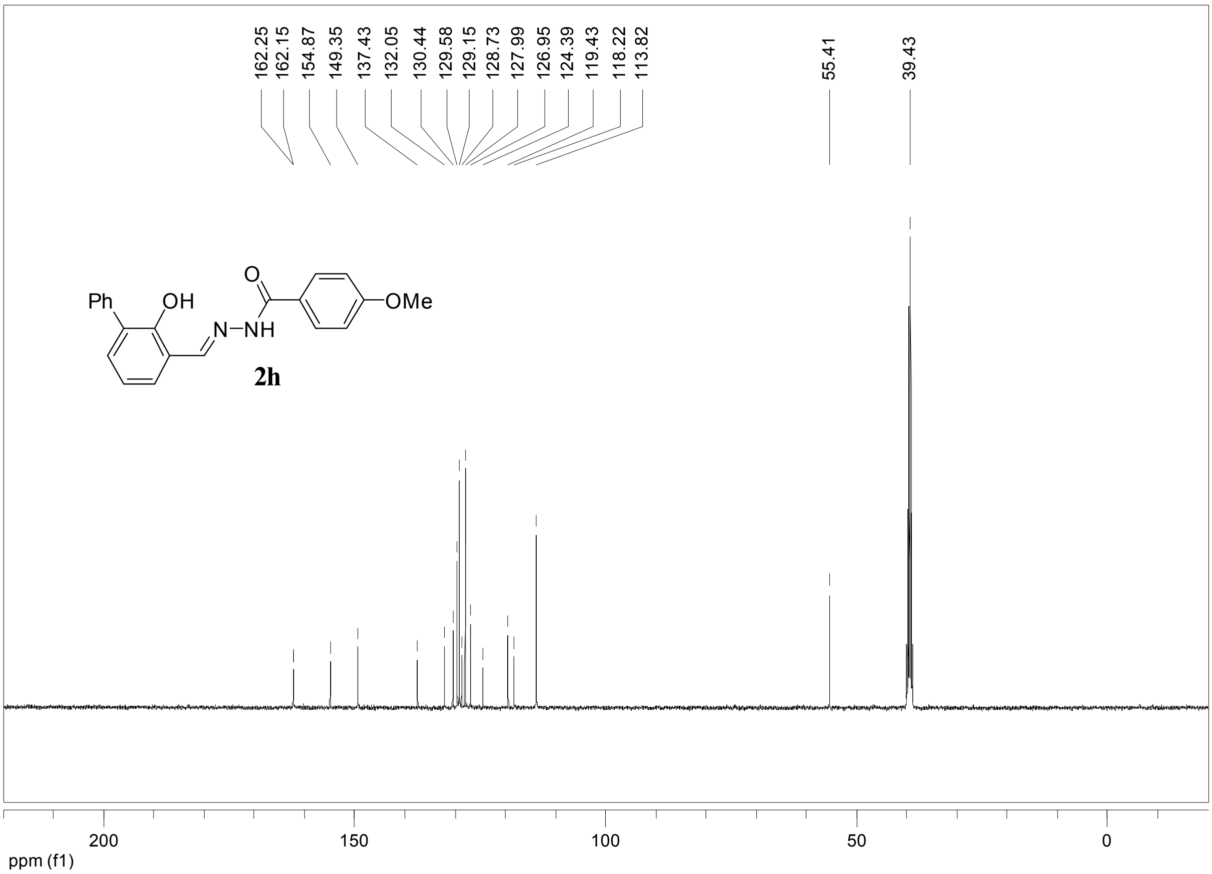

Figure S183. ^{13}C-NMR (100 MHz, DMSO-d_6) spectrum of compound **2h**.

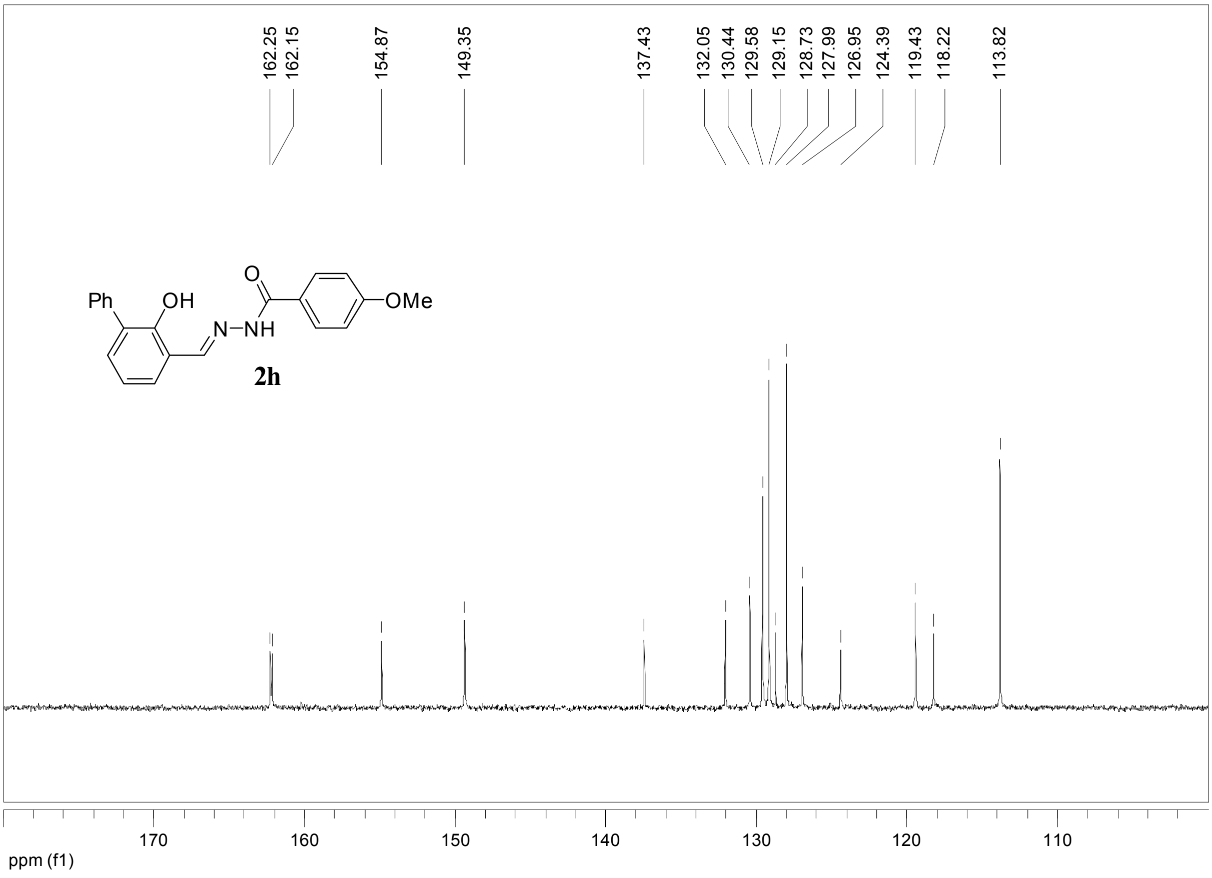

Figure S184. Expansion of ^{13}C-NMR (100 MHz, DMSO-d_6) spectrum of compound **2h**.

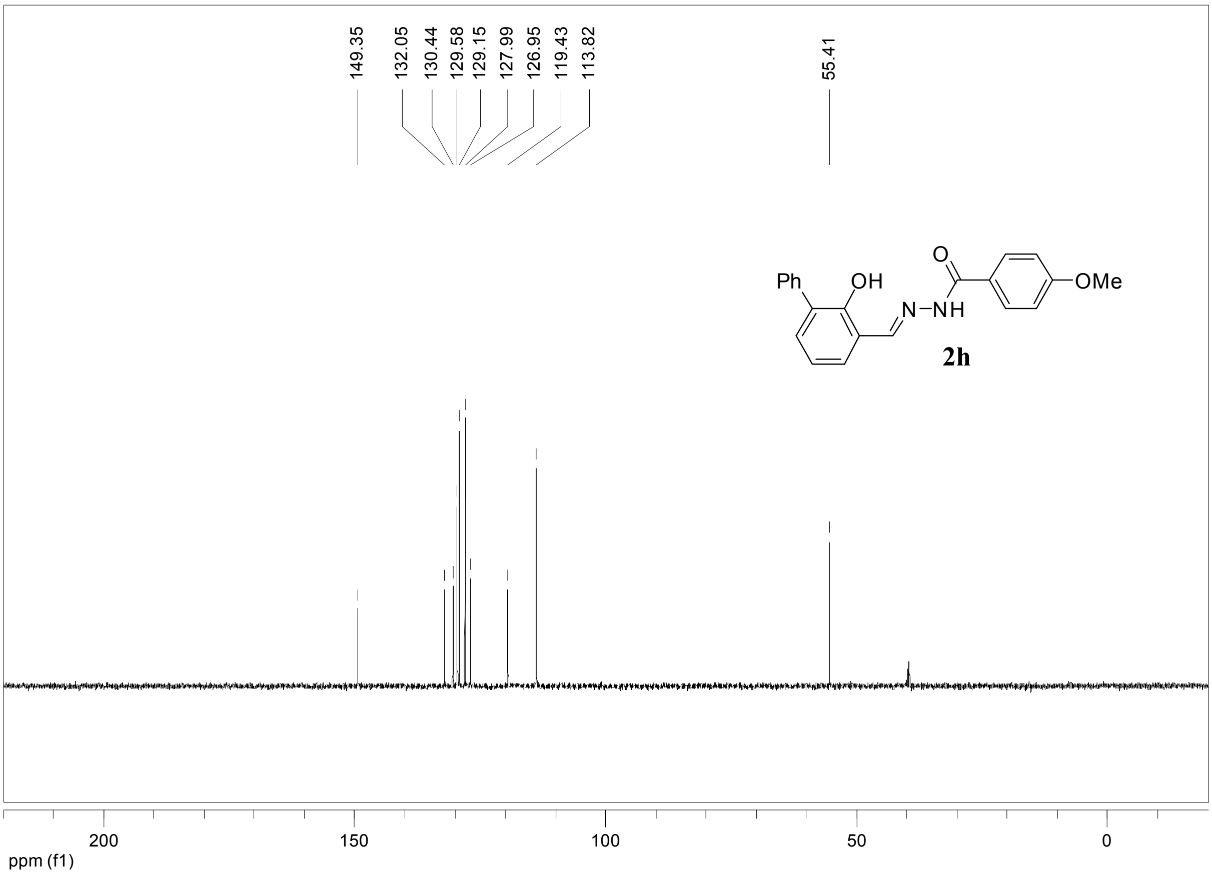

Figure S185. ^{13}C-NMR (100 MHz, DMSO-d_6) dept-135 experiment of compound **2h**.

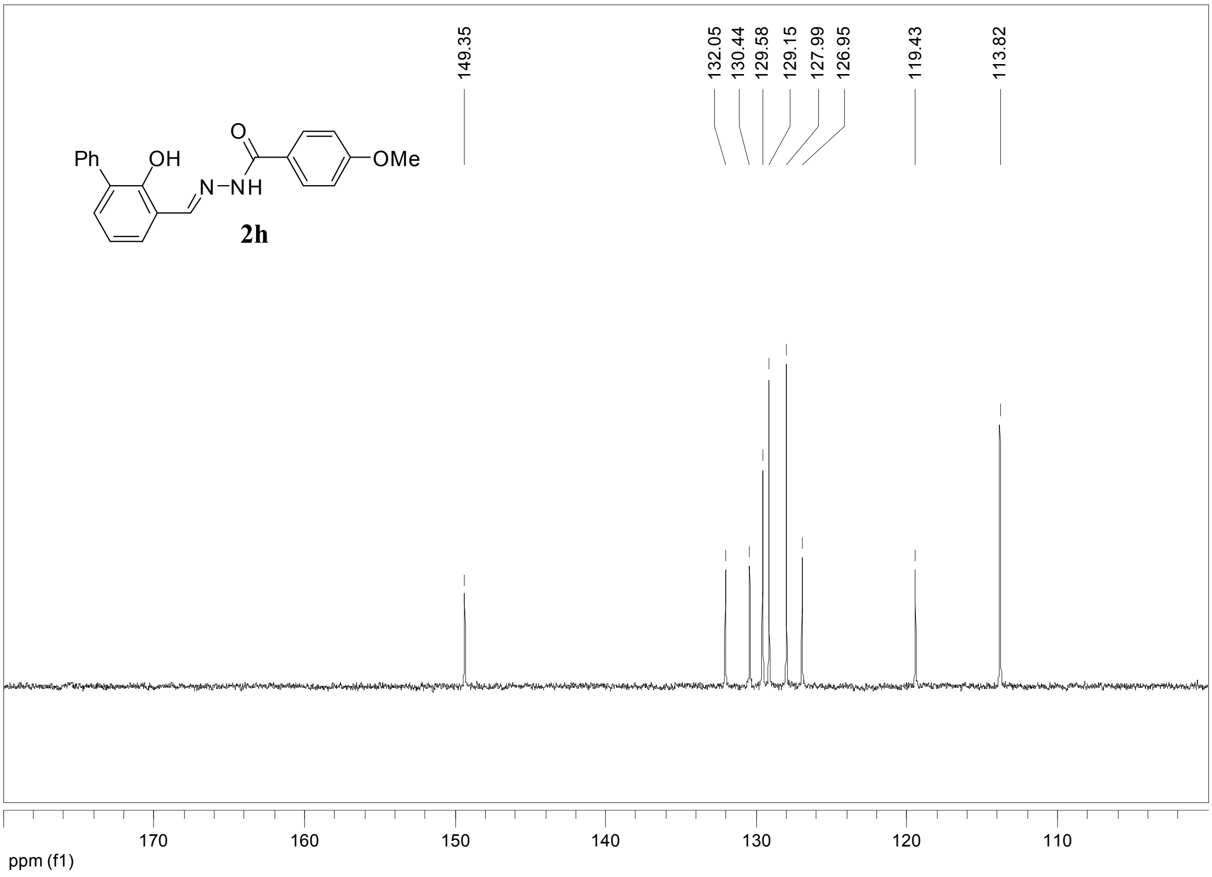

Figure S186. Expansion of ^{13}C-NMR (100 MHz, DMSO-d_6) dept-135 experiment of compound **2h**.

Molecules **2020**, *25*, 1255; doi:10.3390/molecules25051255 www.mdpi.com/journal/molecules

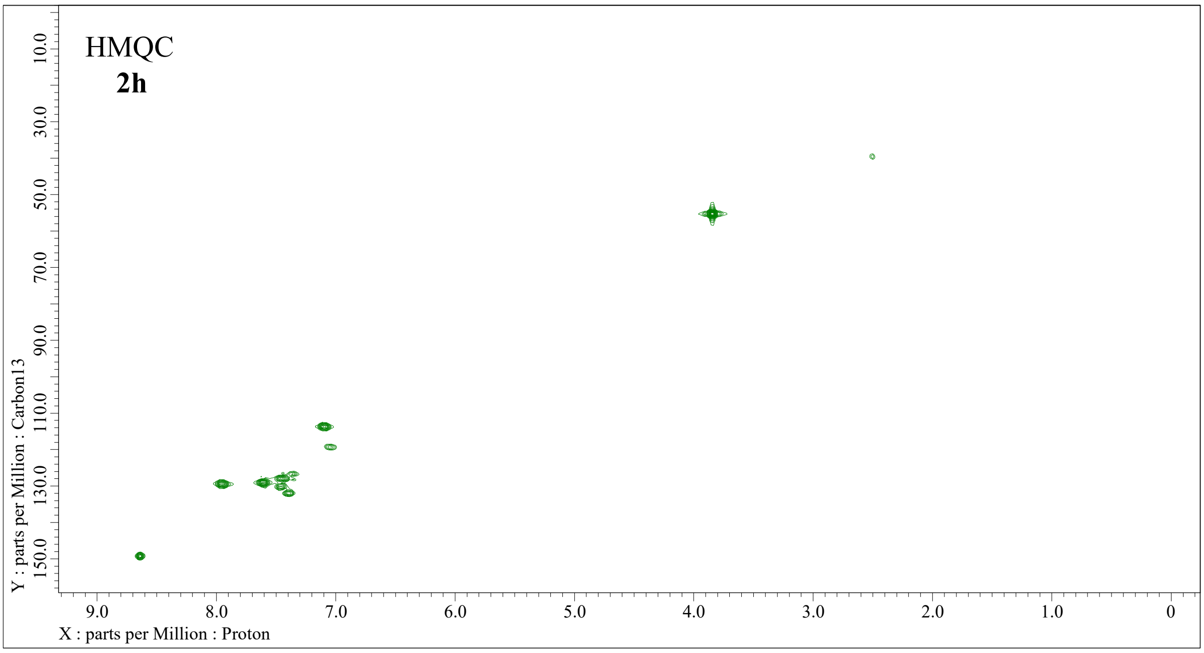

Figure S187. 2D-NMR (400 MHz, DMSO-d_6) HMQC experiment for 4-methoxy-*N*'-[(*E*)-2-hydroxy-3-phenylbenzylidene]benzohydrazide (**2h**).

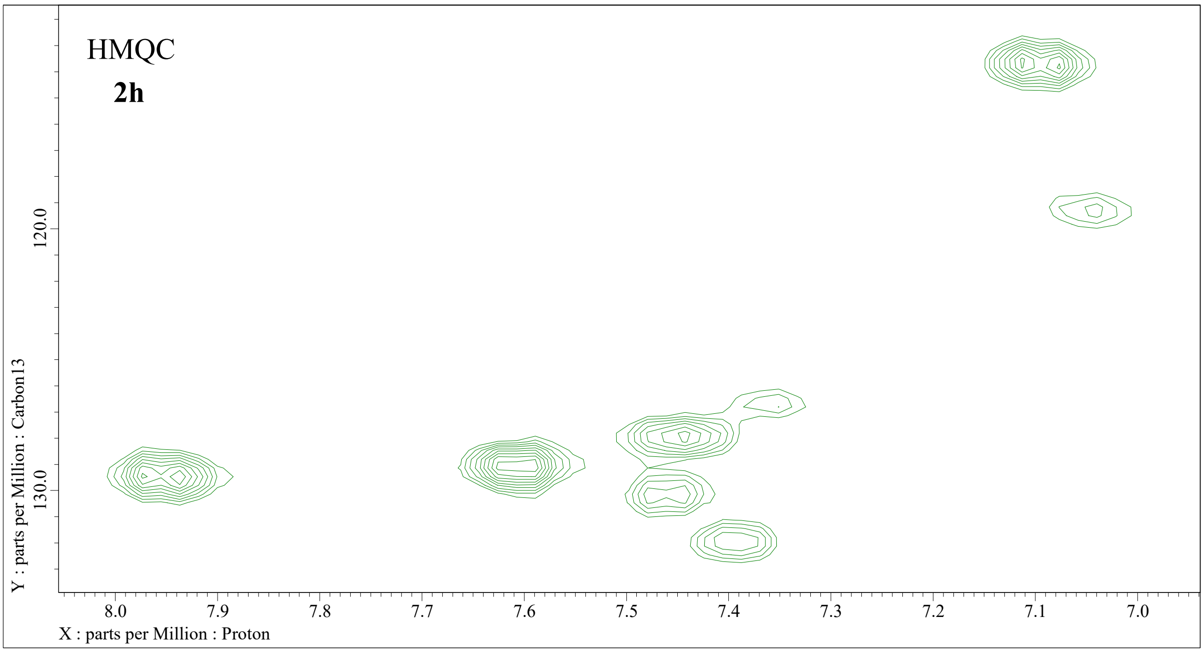

Figure S188. Expansion of 2D-NMR (400 MHz, DMSO-d_6) HMQC experiment of 4-methoxy-*N*'-[(*E*)-2-hydroxy-3-phenylbenzylidene]benzohydrazide (**2h**).

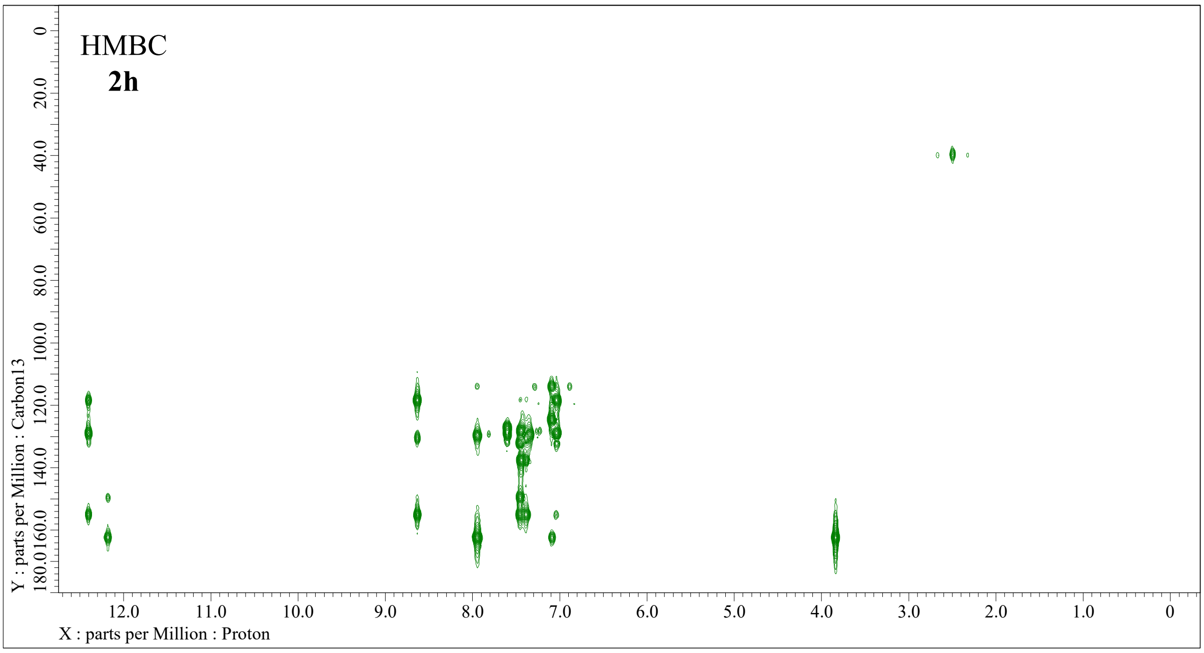

Figure S189. 2D-NMR (400 MHz, DMSO-d_6) HMBC experiment of 4-methoxy-*N*'-[(*E*)-2-hydroxy-3-phenylbenzylidene]benzohydrazide (**2h**).

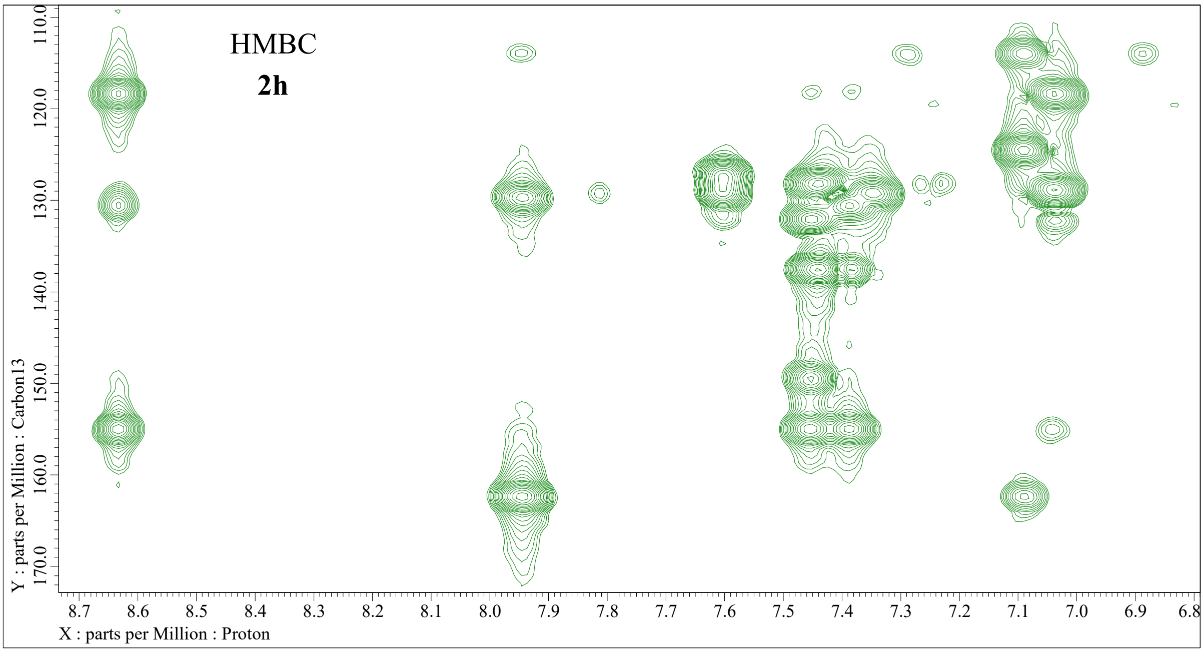

Figure S190. Expansion of 2D-NMR (400 MHz, DMSO-d_6) HMBC experiment of 4-methoxy-*N*'-[(*E*)-2-hydroxy-3-phenylbenzylidene]benzohydrazide (**2h**).

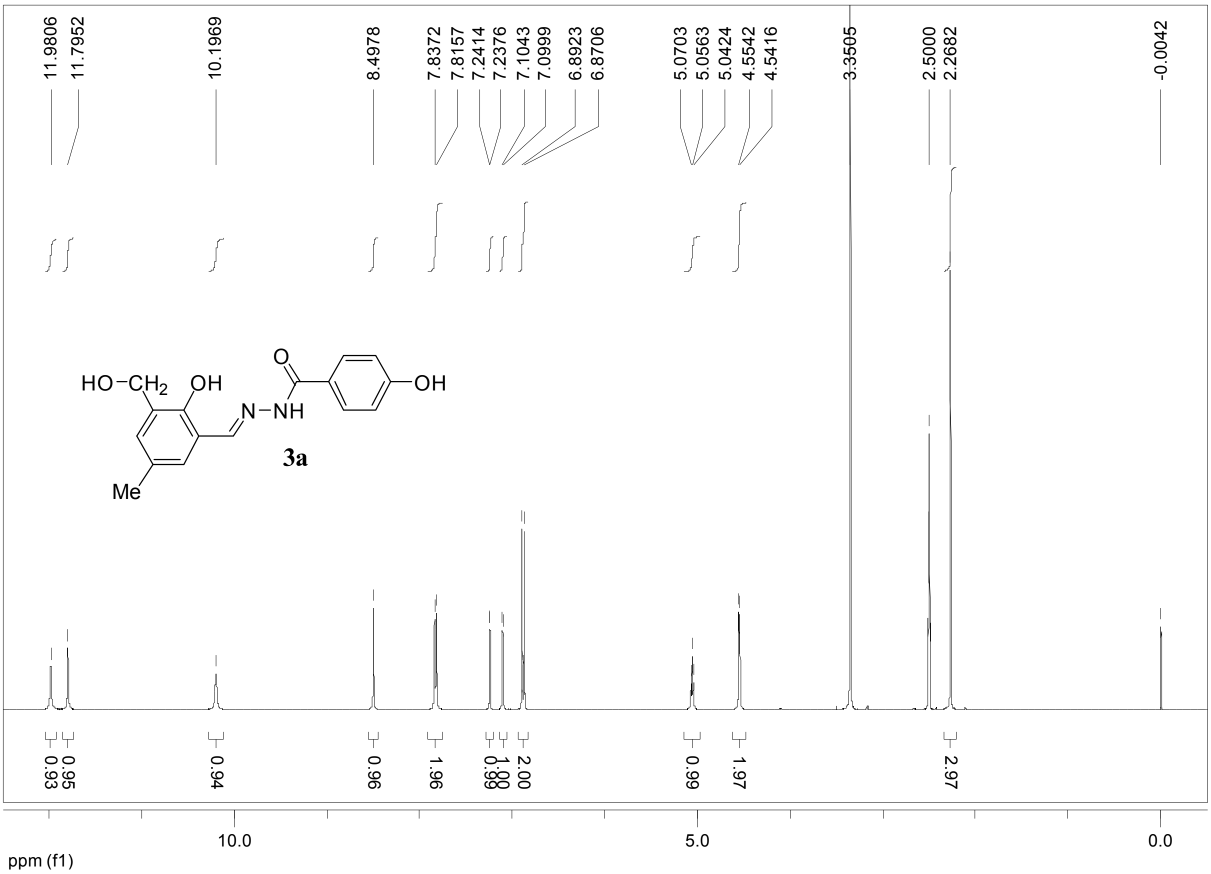

Figure S191. ^{1}H-NMR (400 MHz, DMSO-d_6) spectrum of compound **3a**.

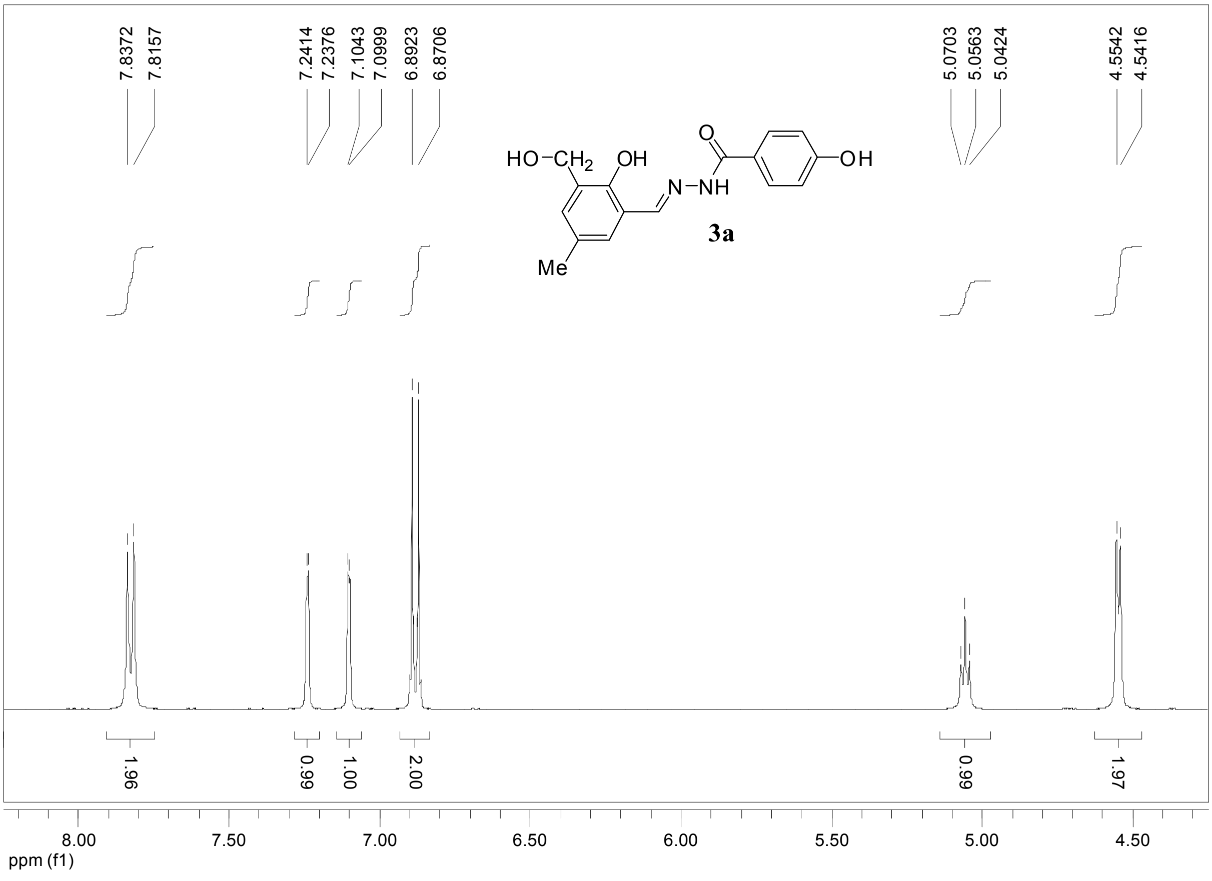

Figure S192. Expansion of ^{1}H-NMR (400 MHz, DMSO-d_6) spectrum of compound **3a**.

Molecules **2020**, *25*, 1255; doi:10.3390/molecules25051255 www.mdpi.com/journal/molecules

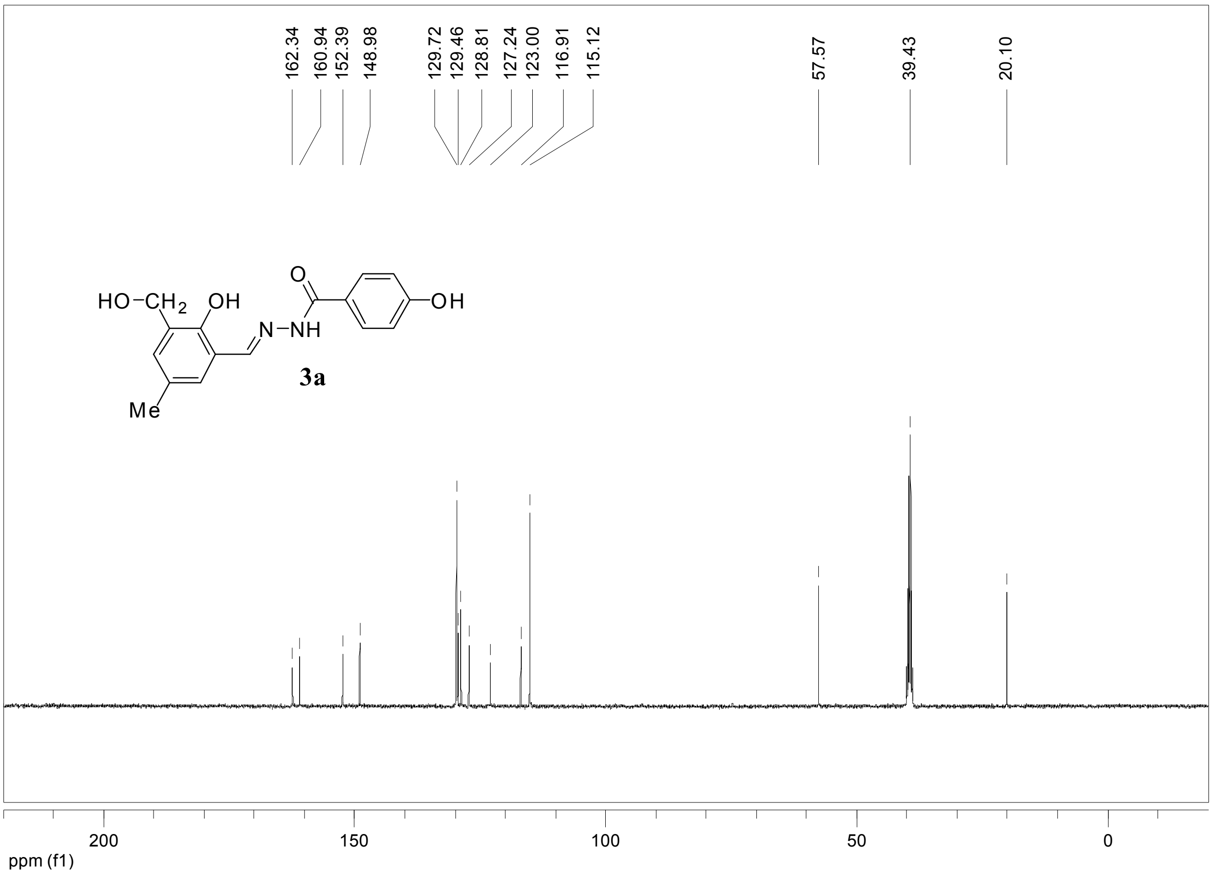

Figure S193. ^{13}C-NMR (100 MHz, DMSO-d_6) spectrum of compound **3a**.

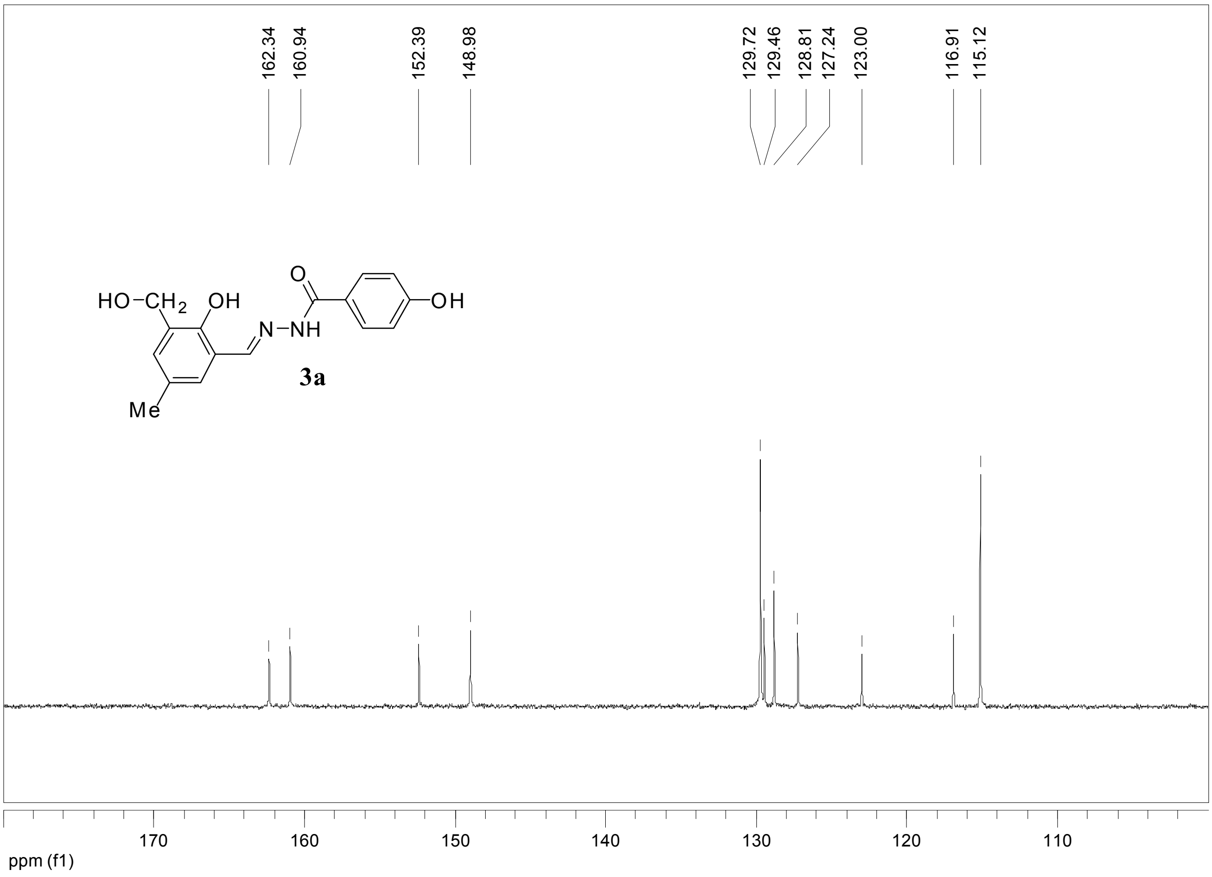

Figure S194. Expansion of ^{13}C-NMR (100 MHz, DMSO-d_6) spectrum of compound **3a**.

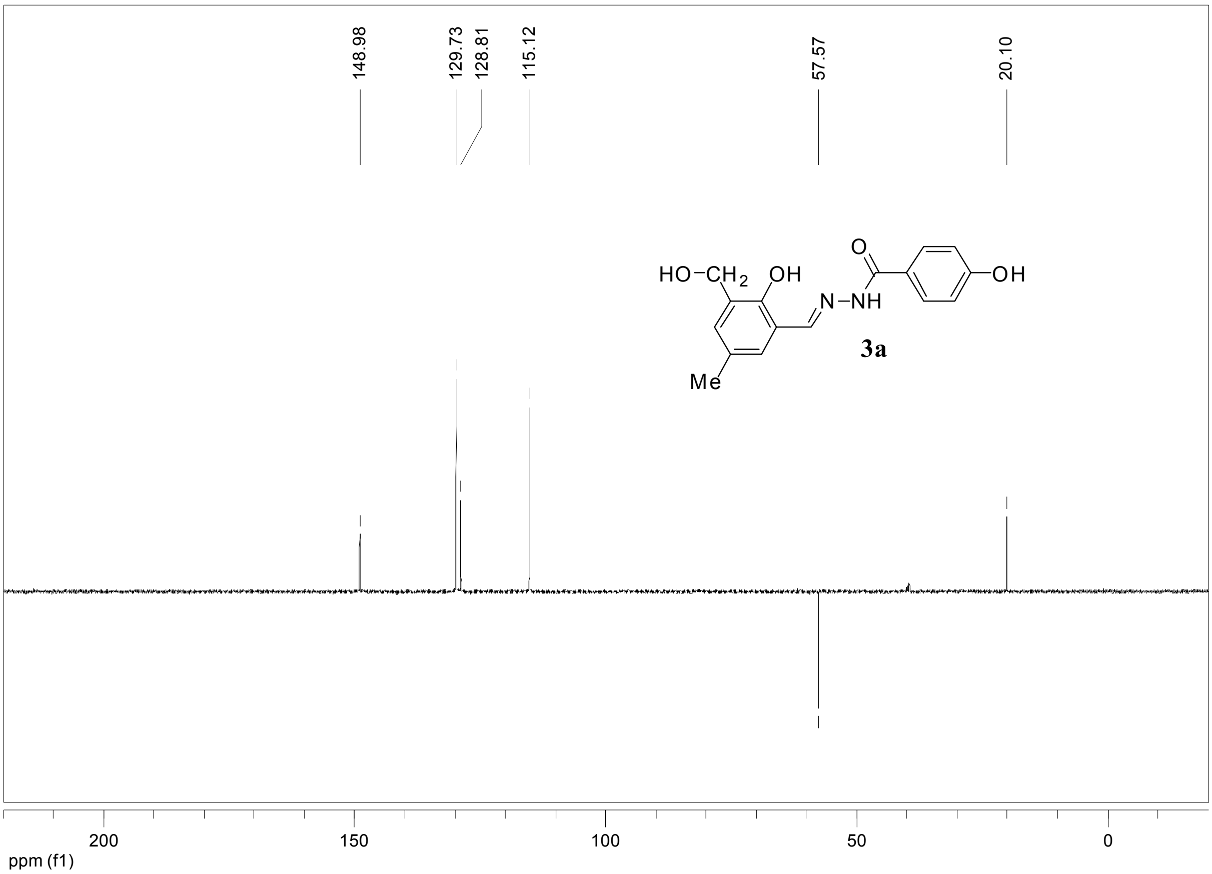

Figure S195. ^{13}C-NMR (100 MHz, DMSO-d_6) dept-135 experiment of compound **3a**.

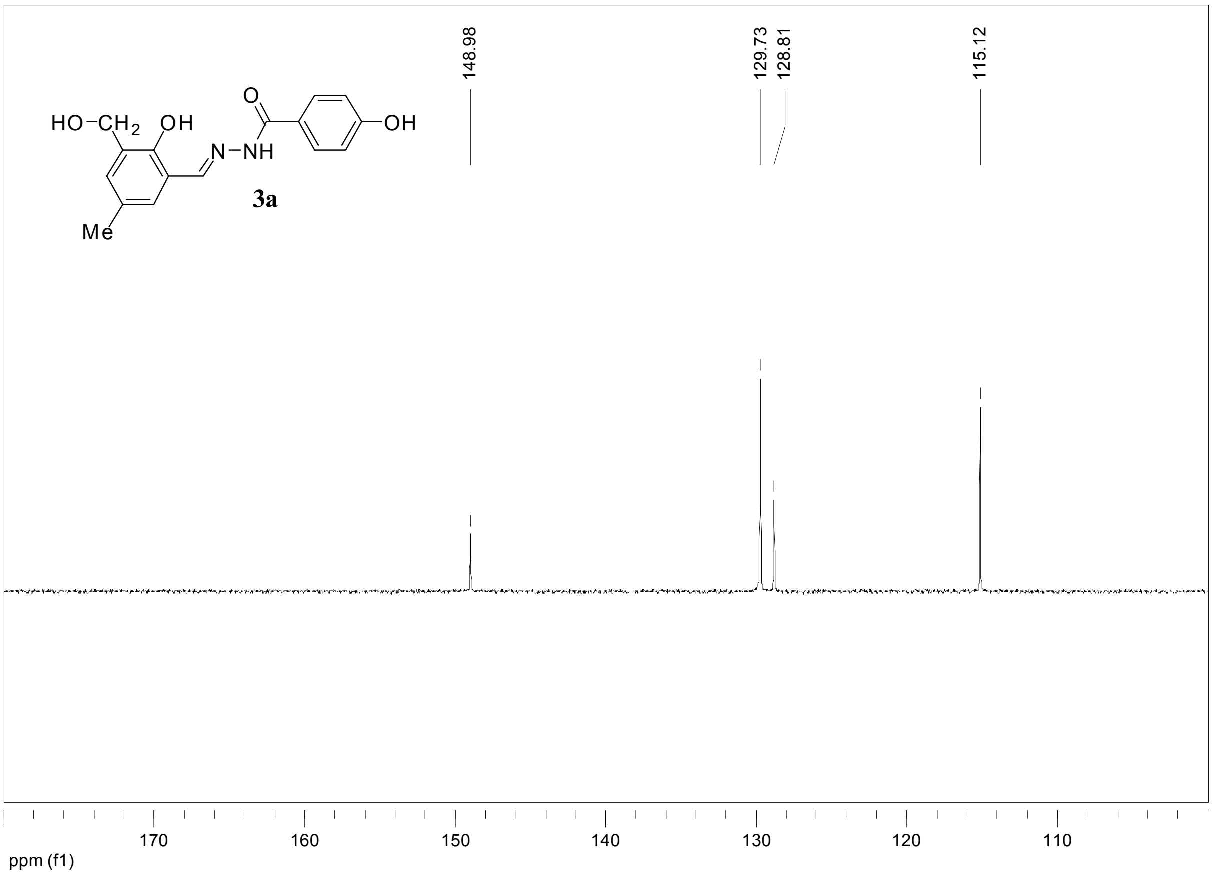

Figure S196. Expansion of ^{13}C-NMR (100 MHz, DMSO-d_6) dept-135 experiment of compound **3a**.

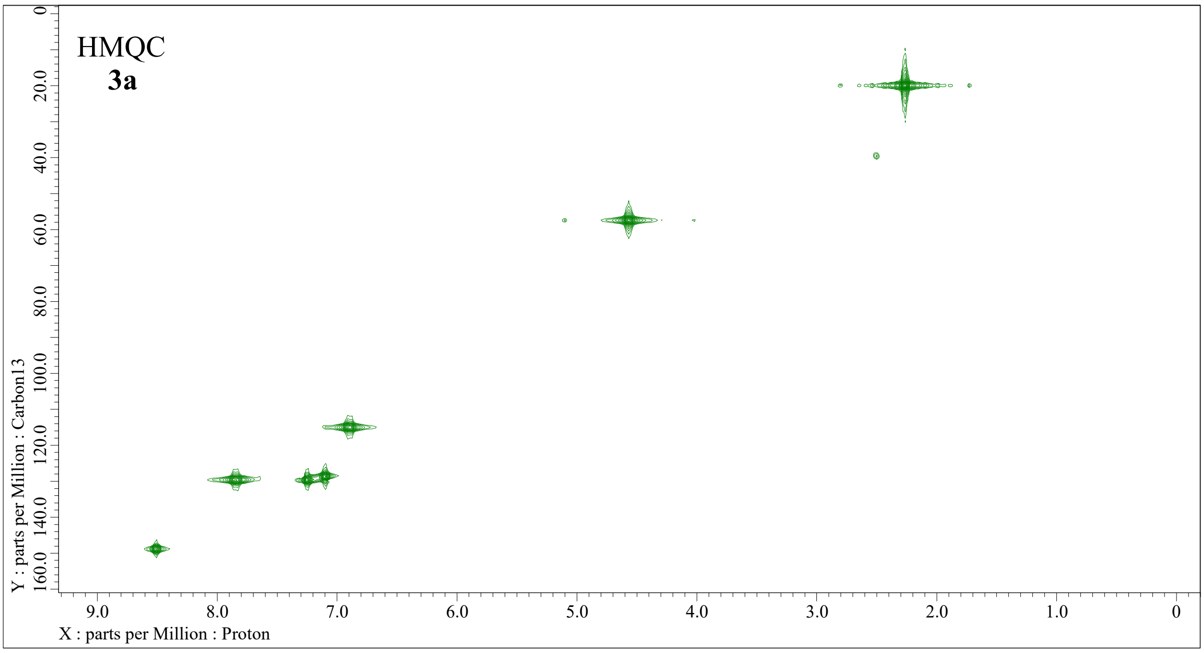

Figure S197. 2D-NMR (400 MHz, DMSO-d_6) HMQC experiment of 4-hydroxy-*N'*-[(*E*)-2-hydroxy-3-hydroxymethyl-5-methylbenzylidene]benzohydrazide (**3a**).

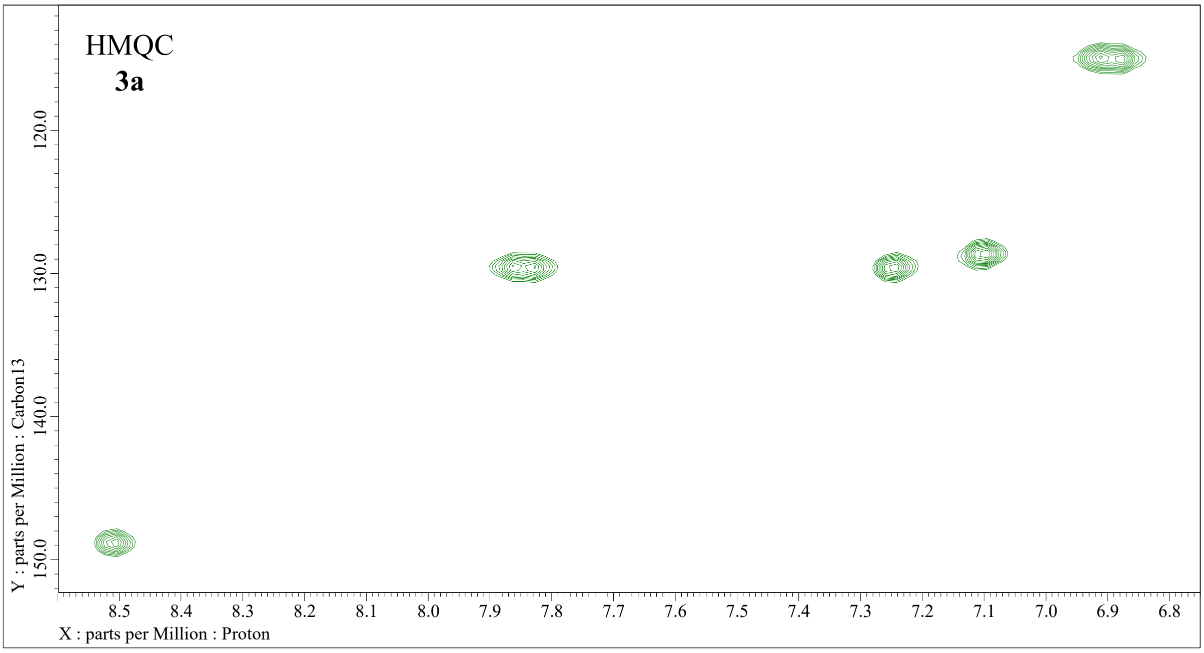

Figure S198. Expansion of 2D-NMR (400 MHz, DMSO-d_6) HMQC experiment of 4-hydroxy-*N'*-[(*E*)-2-hydroxy-3-hydroxymethyl-5-methylbenzylidene]benzohydrazide (**3a**).

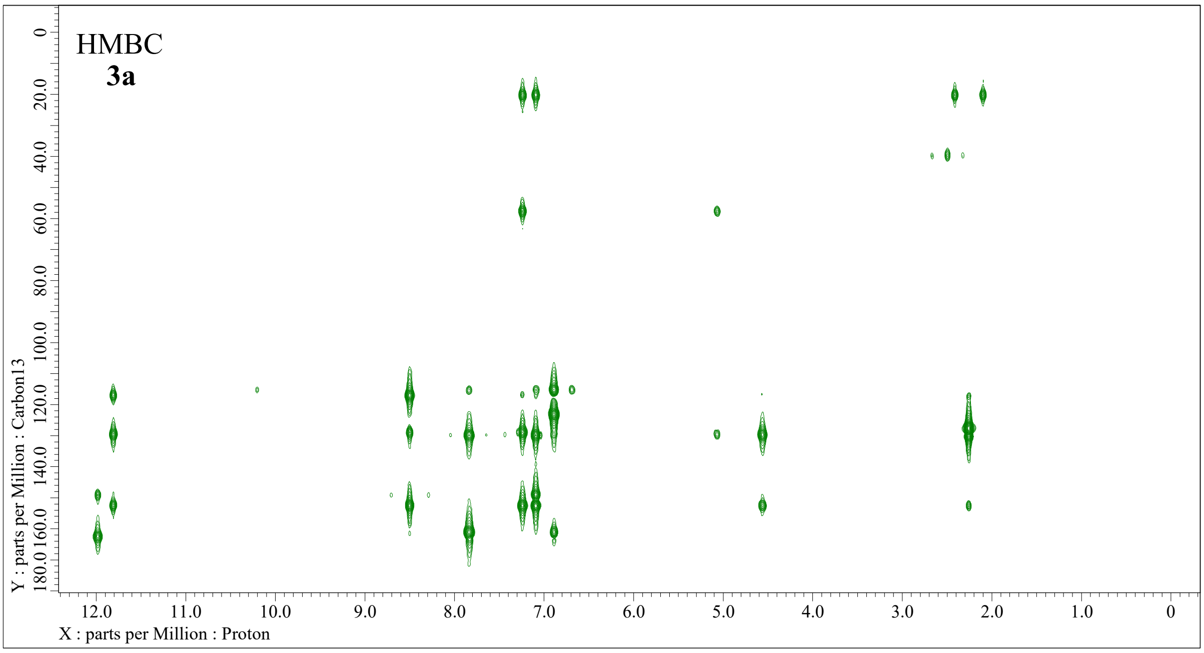

Figure S199. 2D-NMR (400 MHz, DMSO-d_6) HMBC experiment of 4-hydroxy-*N*'-[(*E*)-2-hydroxy-3-hydroxymethyl-5-methylbenzylidene]benzohydrazide (**3a**).

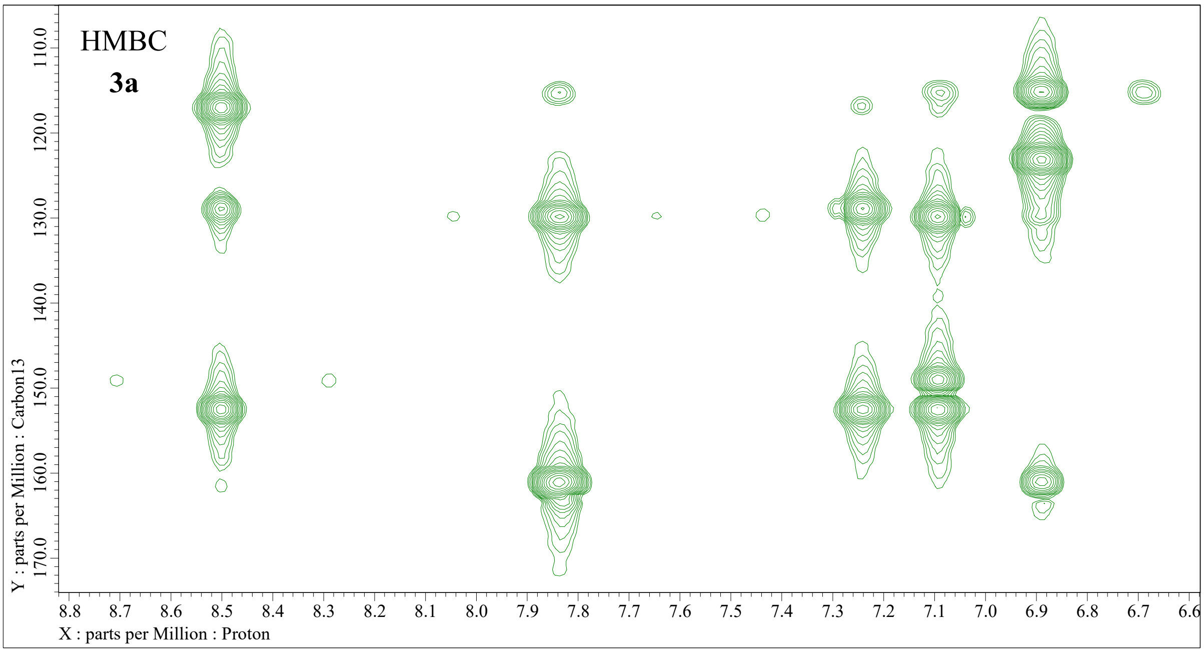

Figure S200. Expansion of 2D-NMR (400 MHz, DMSO-d_6) HMBC experiment of 4-hydroxy-*N*'-[(*E*)-2-hydroxy-3-hydroxymethyl-5-methylbenzylidene]benzohydrazide (**3a**).

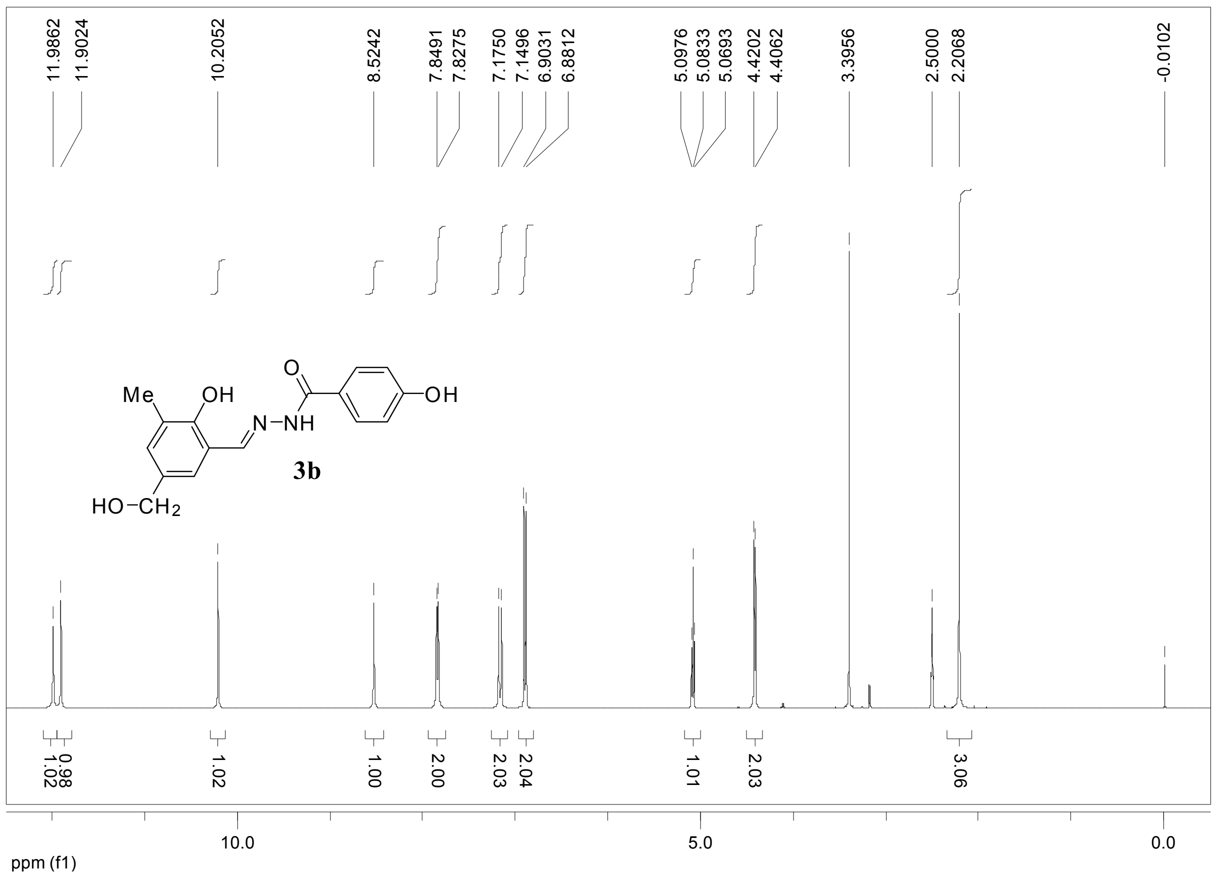

Figure S201. ^{1}H-NMR (400 MHz, DMSO-d_6) spectrum of compound **3b**.

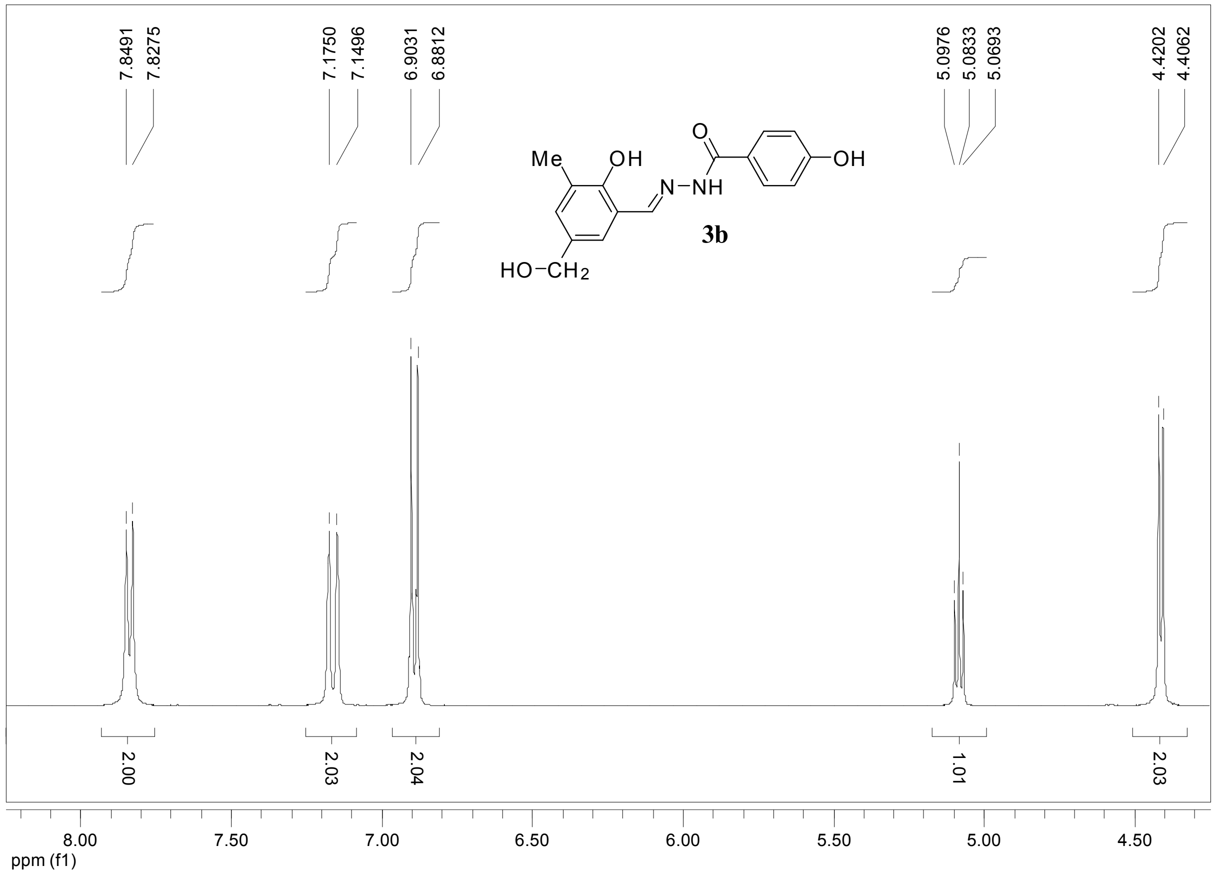

Figure S202. Expansion of ^{1}H-NMR (400 MHz, DMSO-d_6) spectrum of compound **3b**.

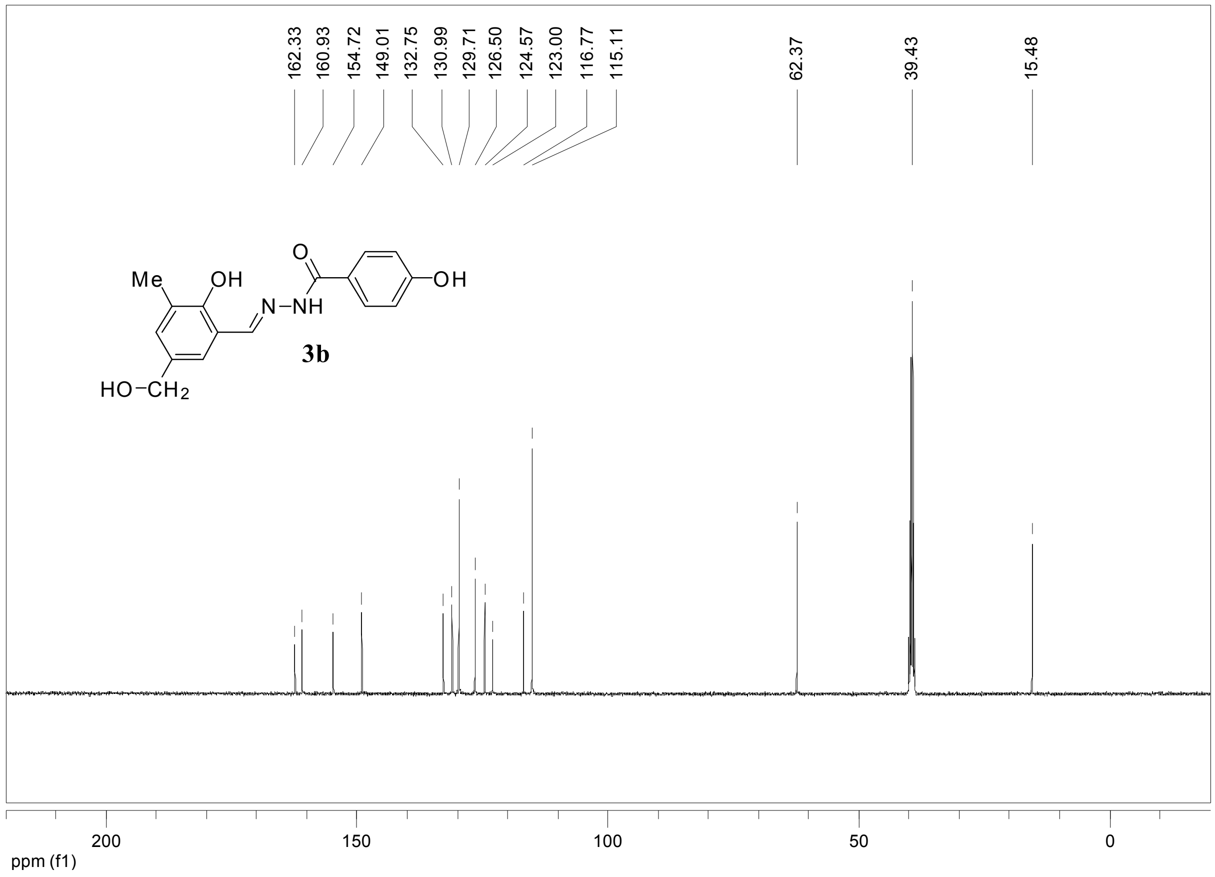

Figure S203. ^{13}C-NMR (100 MHz, DMSO-d_6) spectrum of compound **3b**.

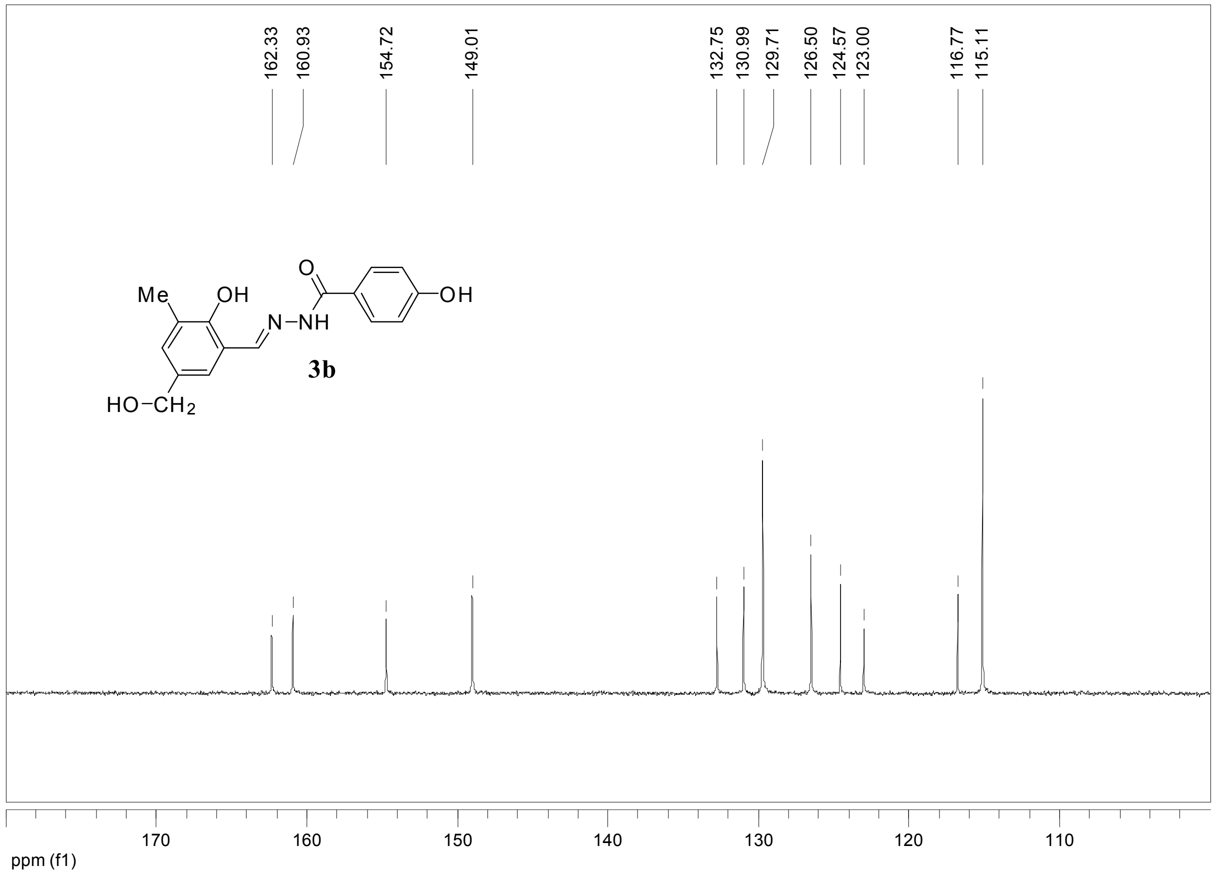

Figure S204. Expansion of ^{13}C-NMR (100 MHz, DMSO-d_6) spectrum of compound **3b**.

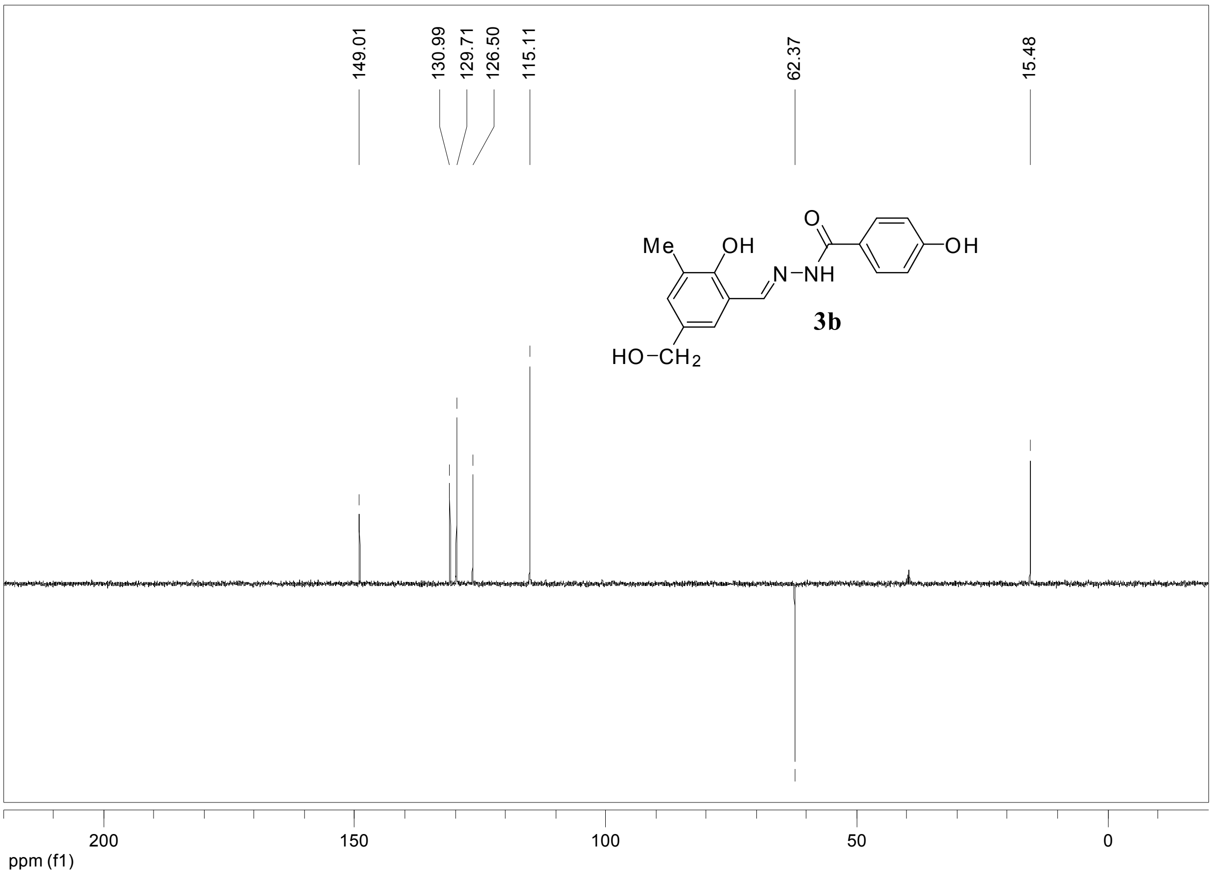

Figure S205. ^{13}C-NMR (100 MHz, DMSO-d_6) dept-135 experiment of compound **3b**.

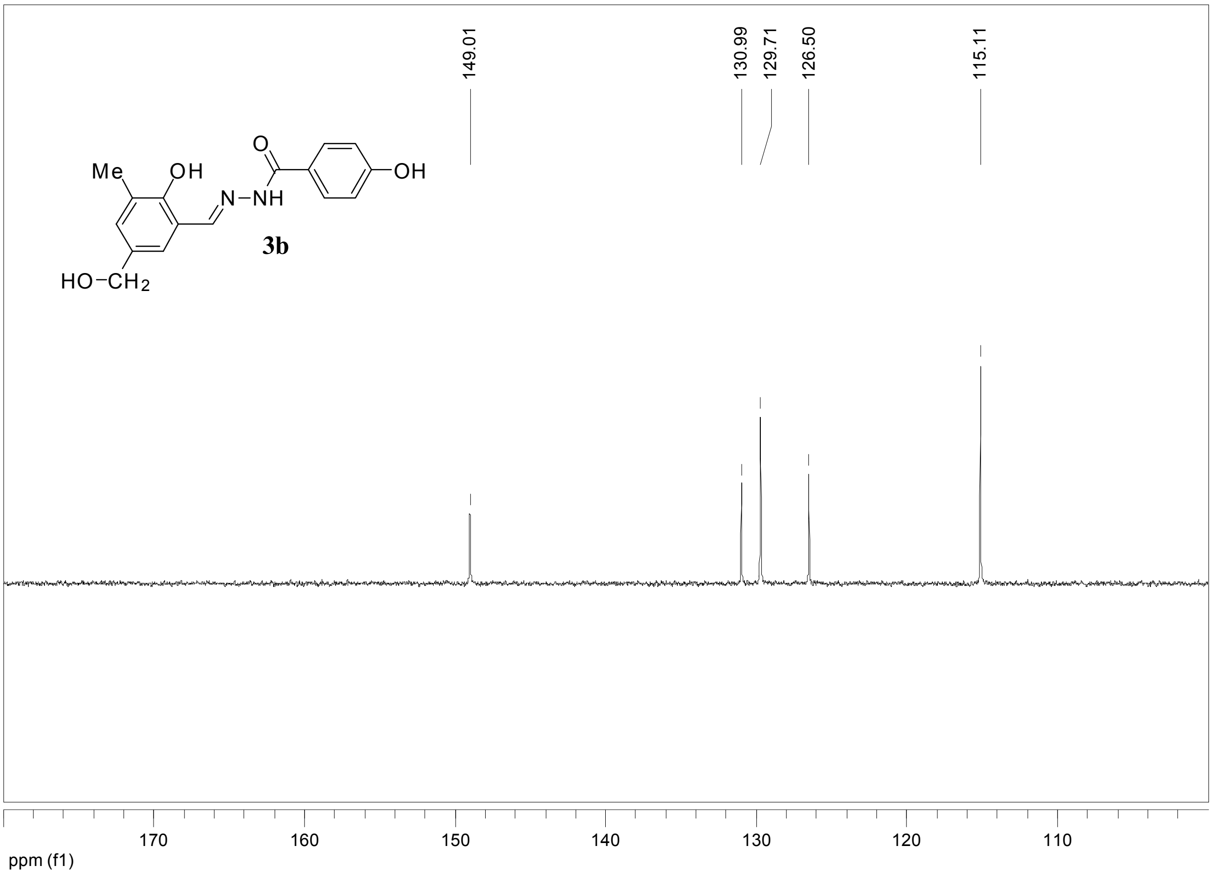

Figure S206. Expansion of ^{13}C-NMR (100 MHz, DMSO-d_6) dept-135 experiment of compound **3b**.

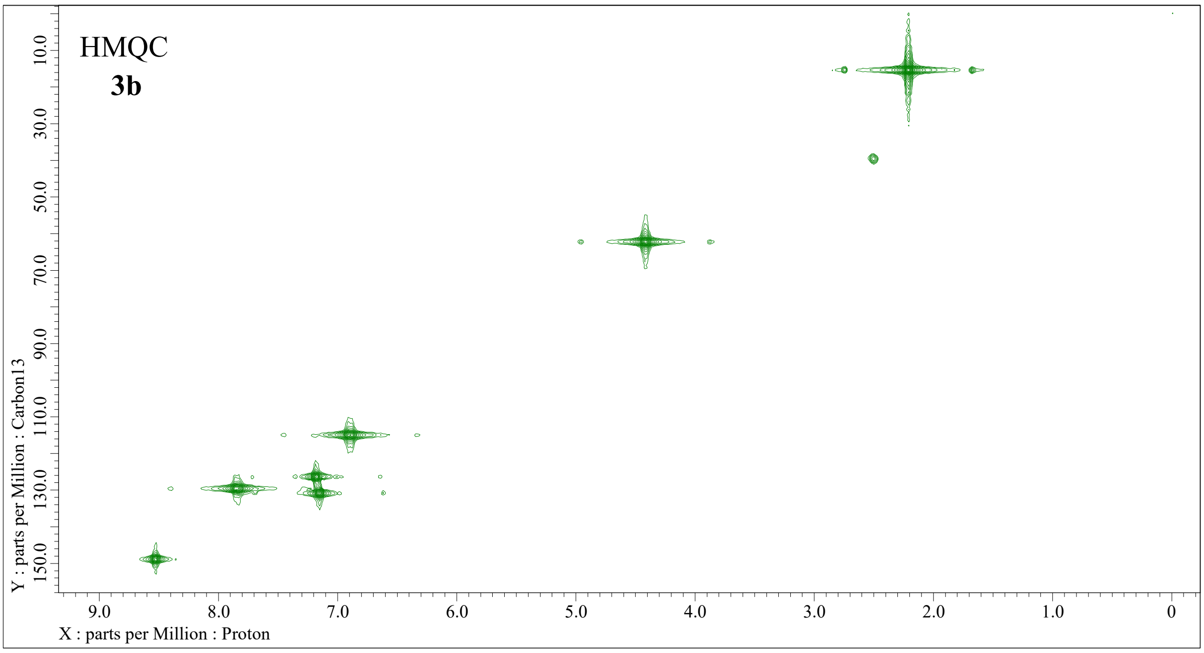

Figure S207. 2D-NMR (400 MHz, DMSO-d_6) HMQC experiment of 4-hydroxy-*N*'-[(*E*)-2-hydroxy-5-hydroxymethyl-3-methylbenzylidene]benzohydrazide (**3b**).

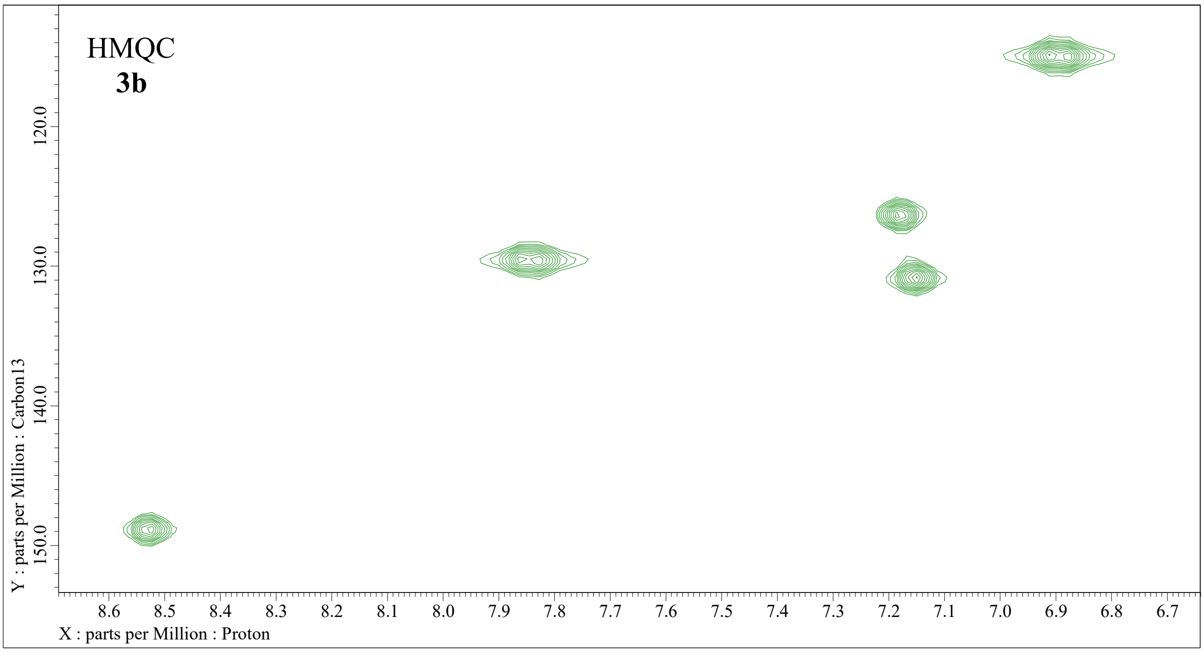

Figure S208. Expansion of 2D-NMR (400 MHz, DMSO-d_6) HMQC experiment of 4-hydroxy-*N*'-[(*E*)-2-hydroxy-5-hydroxymethyl-3-methylbenzylidene]benzohydrazide (**3b**).

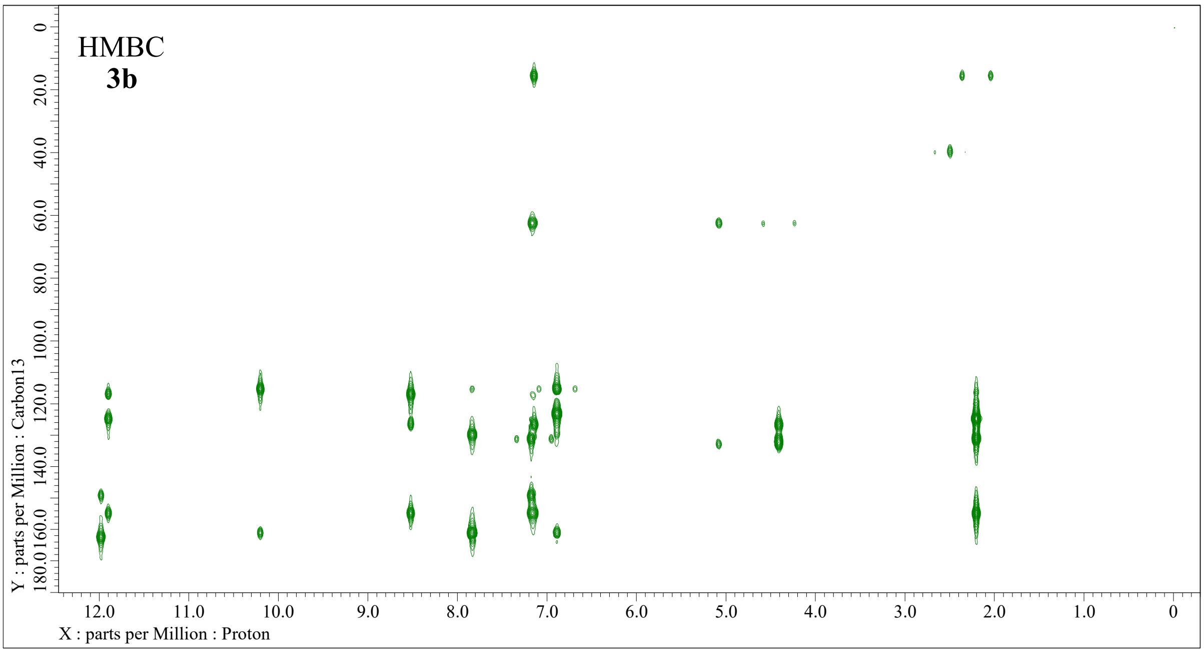

Figure S209. 2D-NMR (400 MHz, DMSO-d_6) HMBC experiment of 4-hydroxy-*N*'-[(*E*)-2-hydroxy-5-hydroxymethyl-3-methylbenzylidene]benzohydrazide (**3b**).

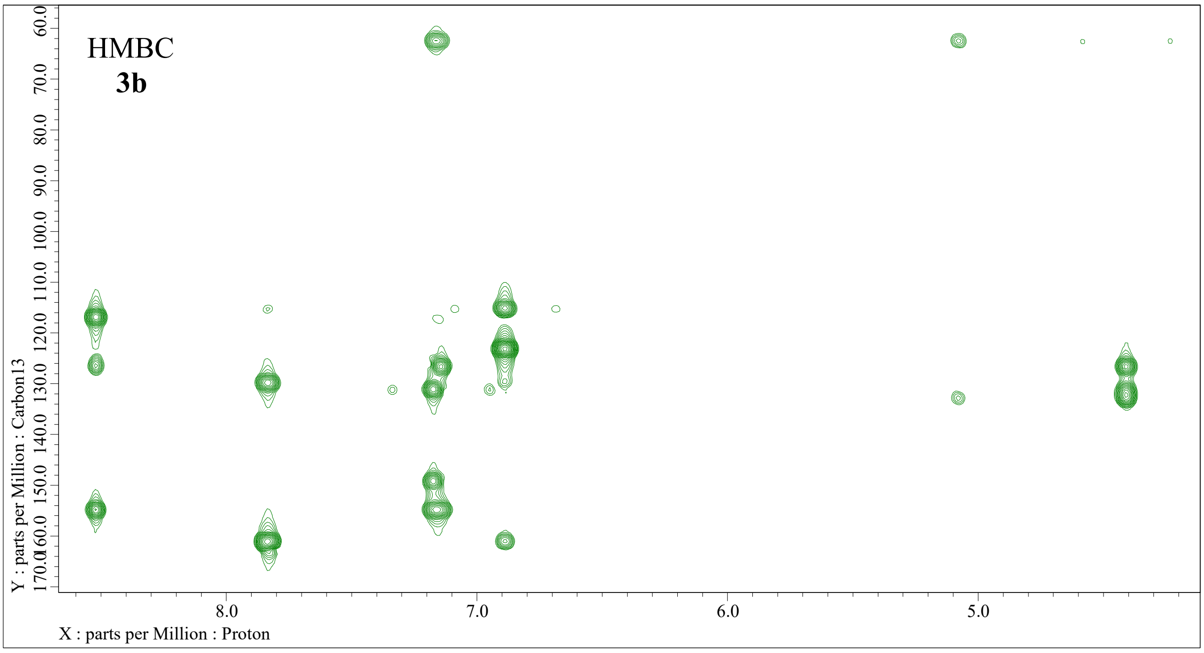

Figure S210. Expansion of 2D-NMR (400 MHz, DMSO-d_6) HMBC experiment of 4-hydroxy-*N*'-[(*E*)-2-hydroxy-5-hydroxymethyl-3-methylbenzylidene]benzohydrazide (**3b**).

Molecules **2020**, *25*, 1255; doi:10.3390/molecules25051255 www.mdpi.com/journal/molecules

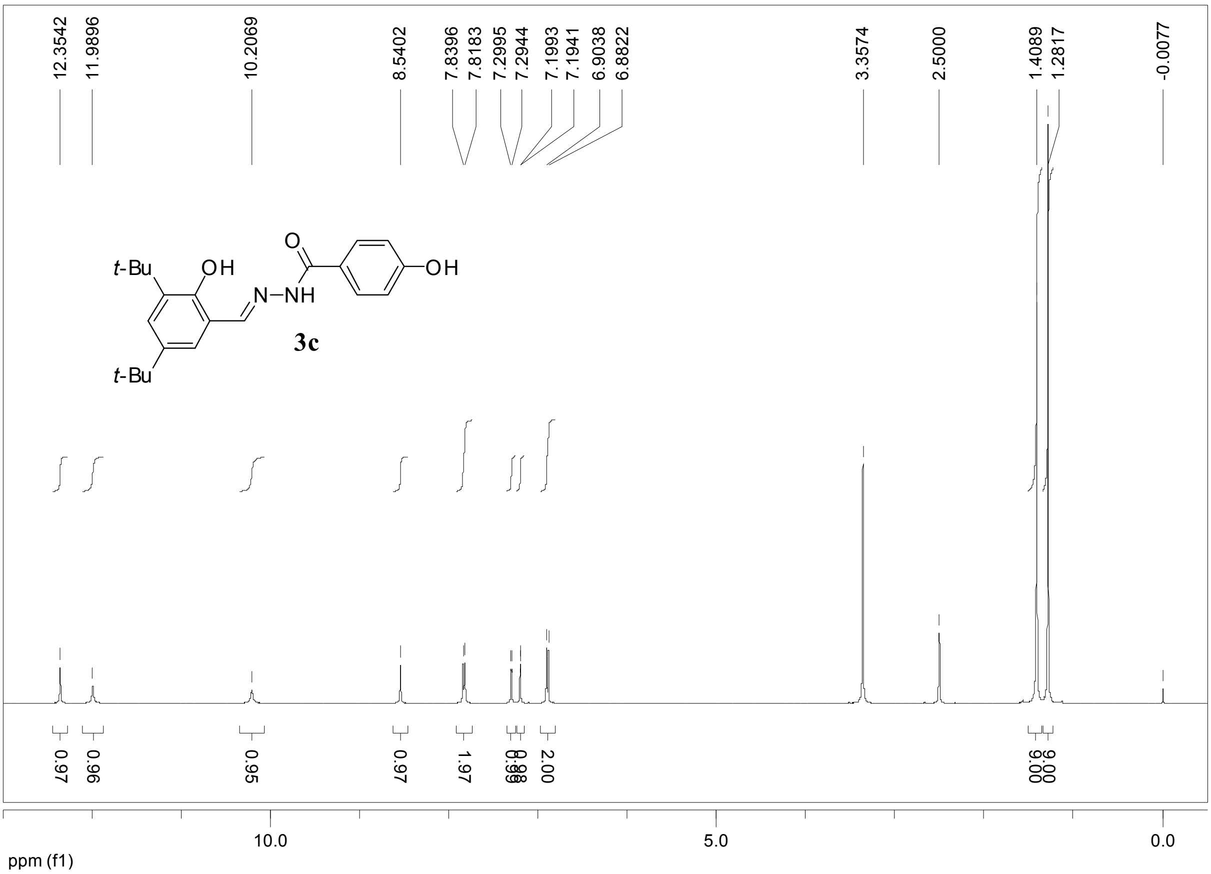

Figure S211. ^{1}H-NMR (400 MHz, DMSO-d_6) spectrum of compound **3c**.

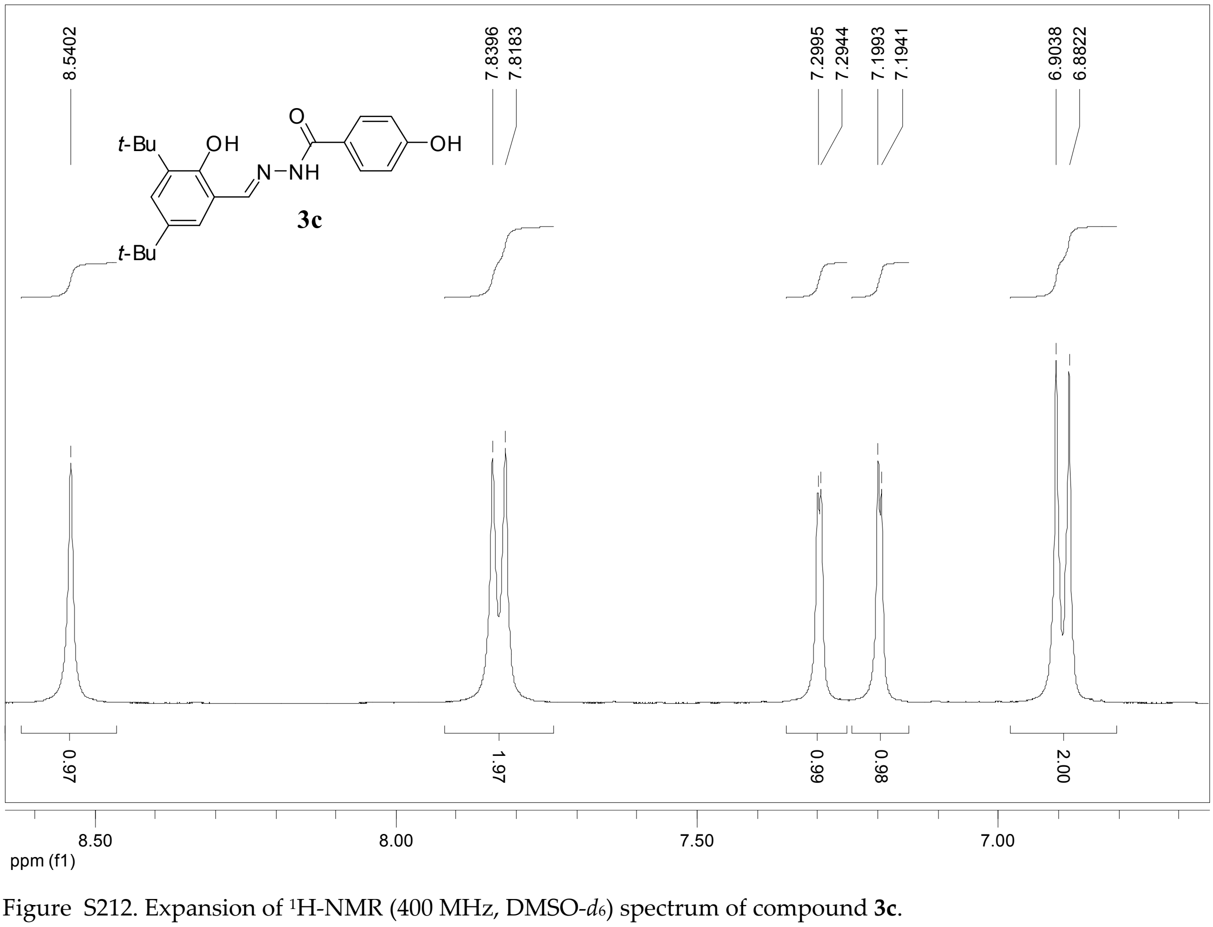

Figure S212. Expansion of ^{1}H-NMR (400 MHz, DMSO-d_6) spectrum of compound **3c**.

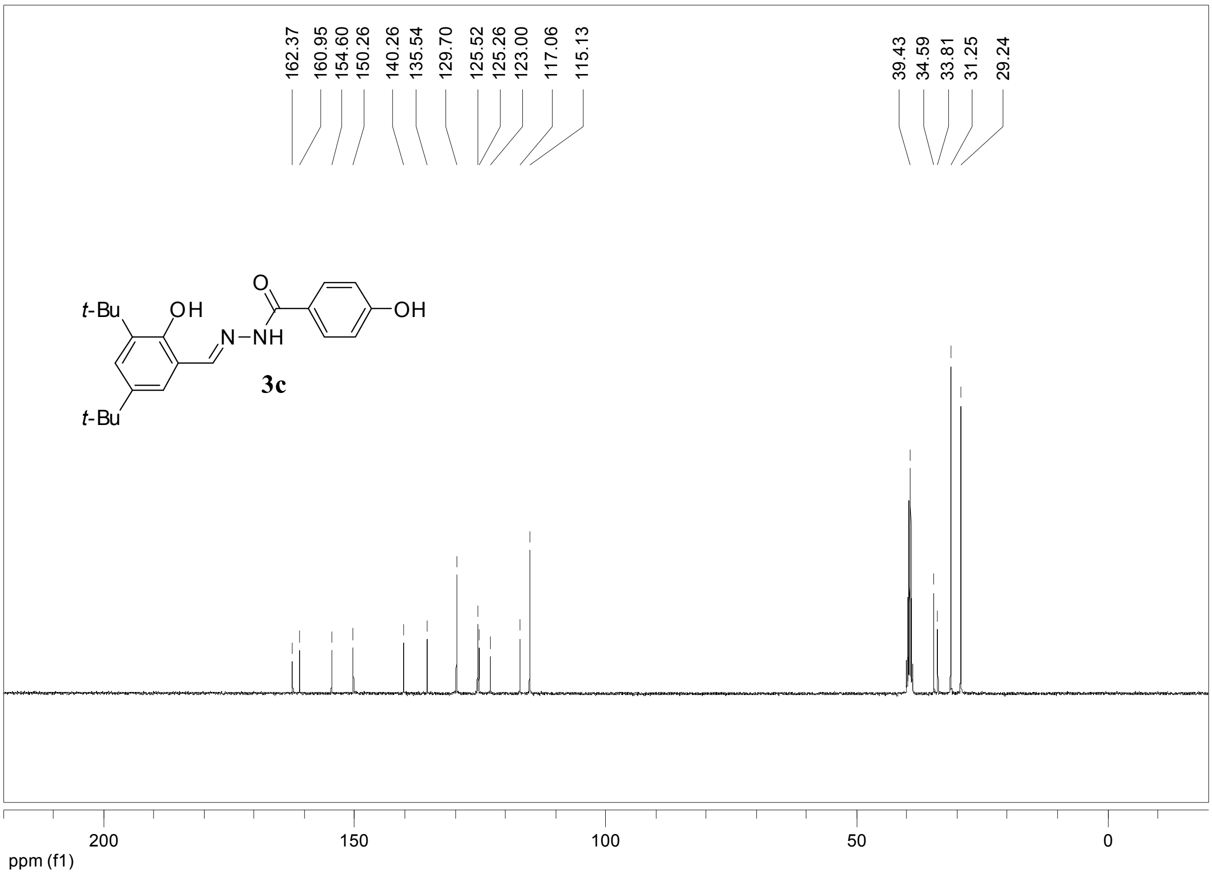

Figure S213. ^{13}C-NMR (100 MHz, DMSO-d_6) spectrum of compound **3c**.

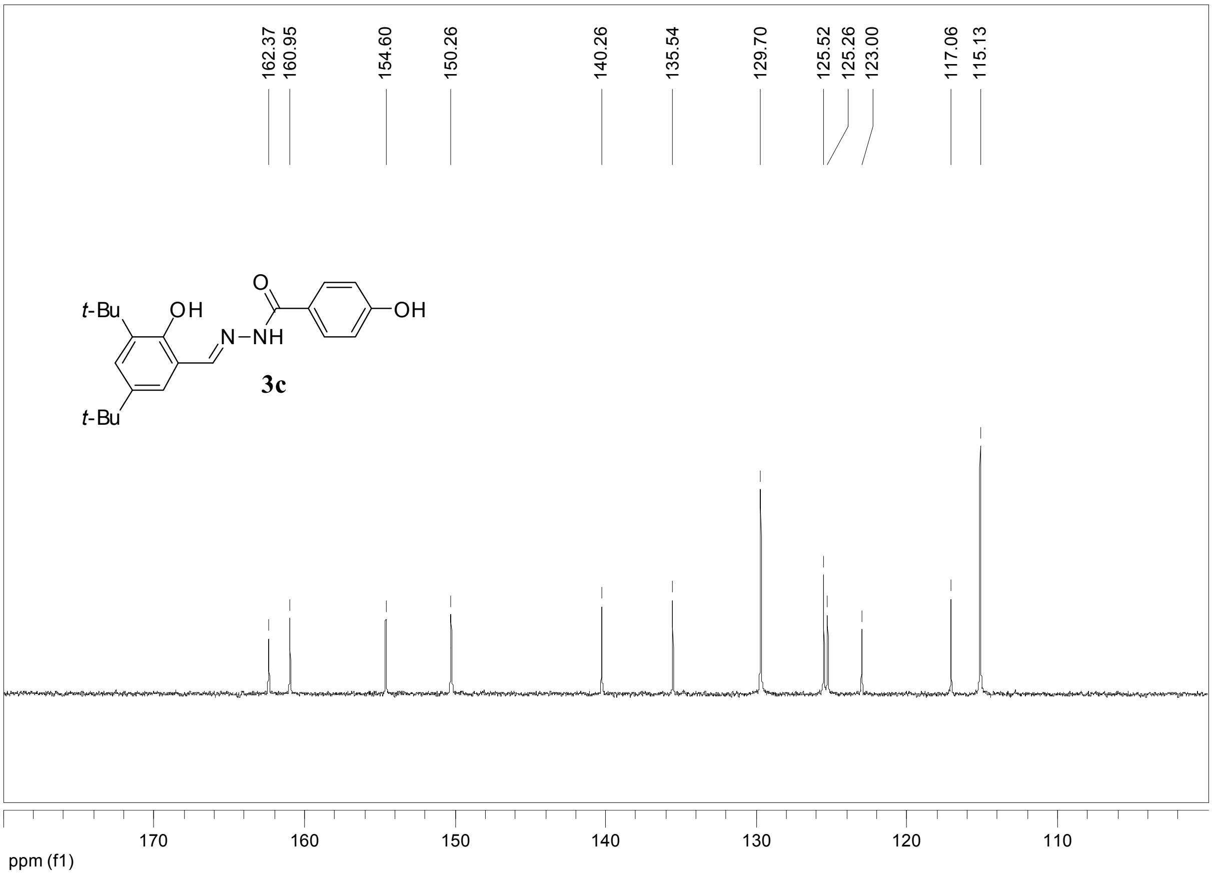

Figure S214. Expansion of ^{13}C-NMR (100 MHz, DMSO-d_6) spectrum of compound **3c**.

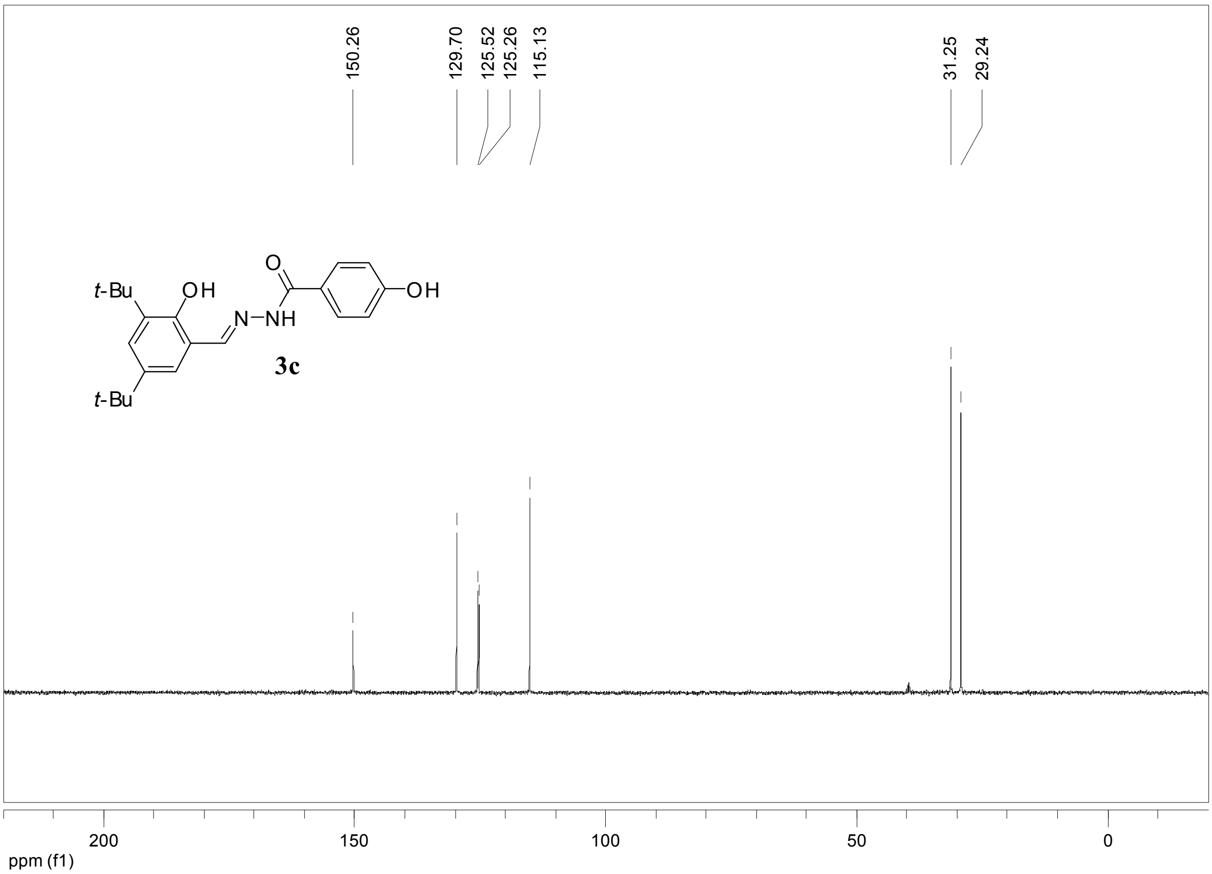

Figure S215. ^{13}C-NMR (100 MHz, DMSO-d_6) dept-135 experiment of compound **3c**.

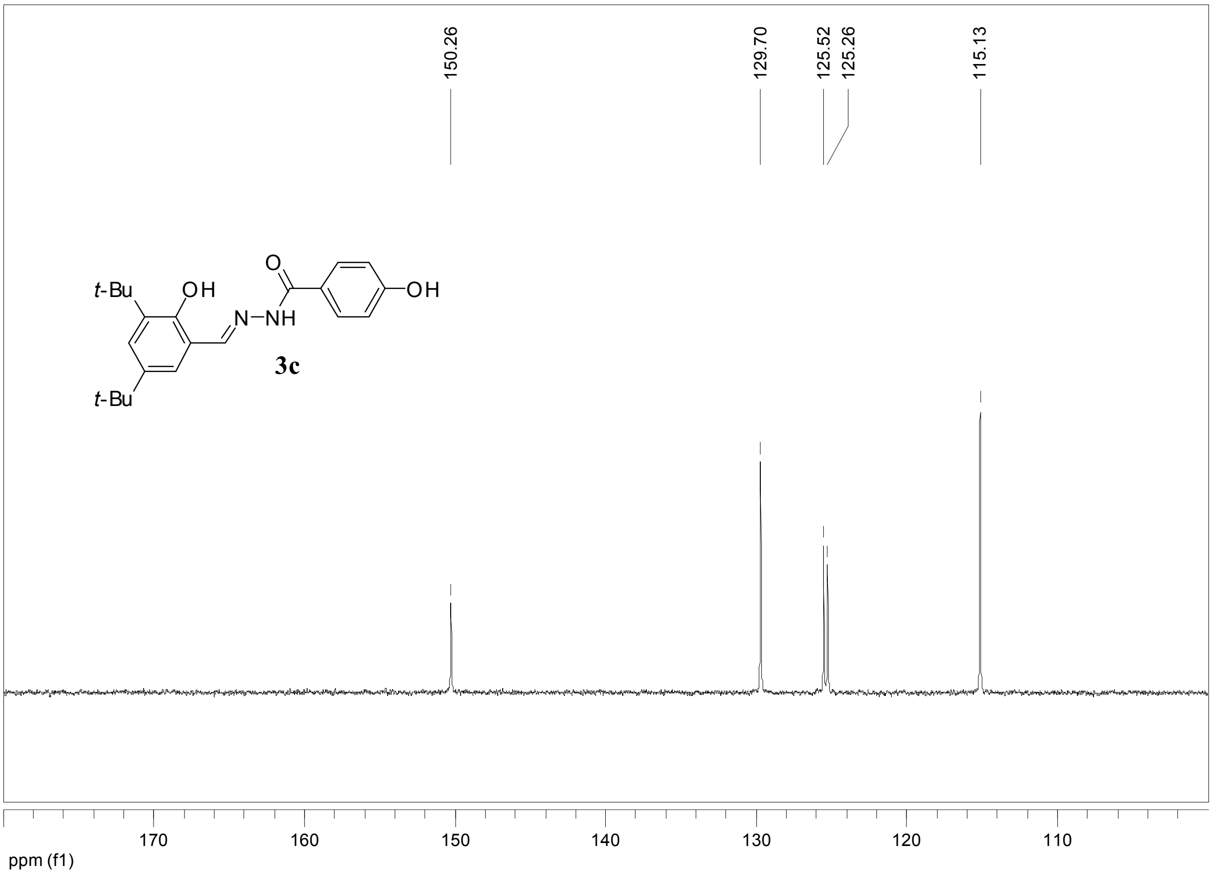

Figure S116. Expansion of ^{13}C-NMR (100 MHz, DMSO-d_6) dept-135 experiment of compound **3c**.

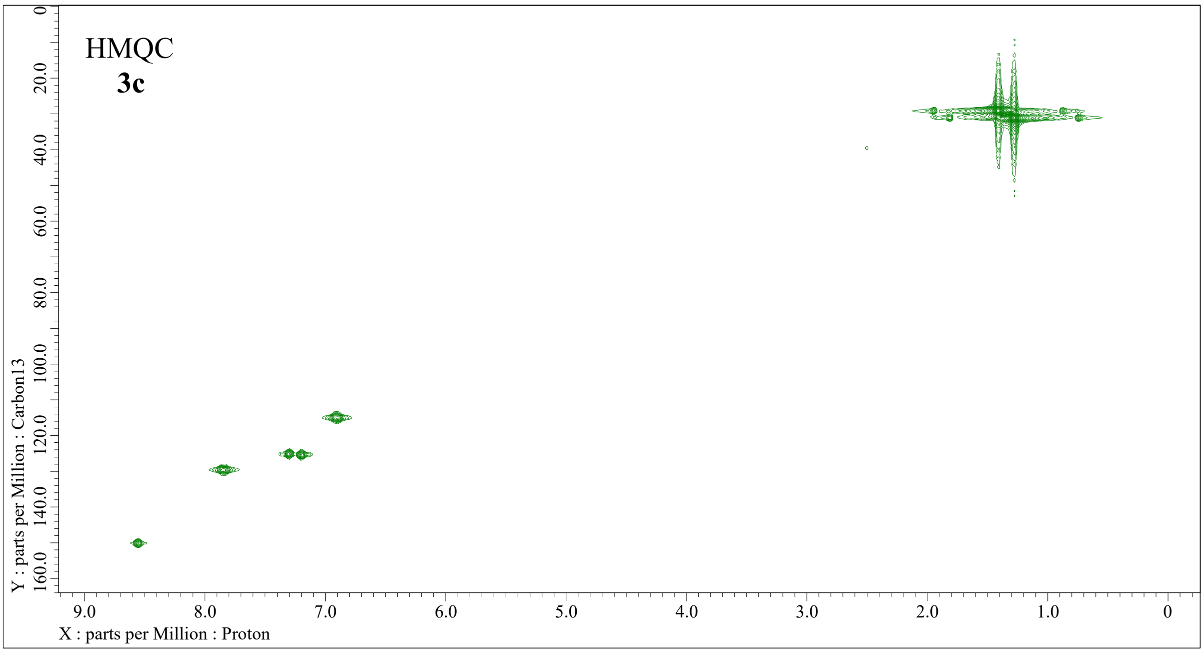

Figure S217. 2D-NMR (400 MHz, DMSO-d_6) HMQC experiment of *N*'-[(*E*)-(3,5-di-*tert*-butyl-2-hydroxyphenyl)methylidene]-4-hydroxybenzohydrazide (**3c**).

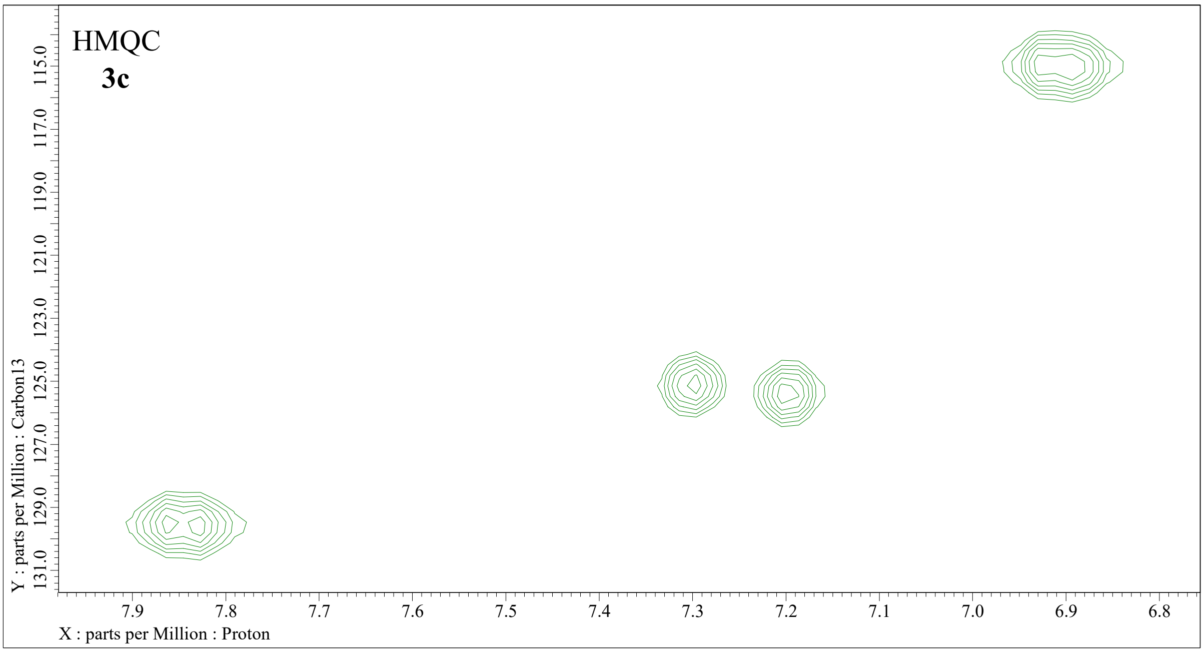

Figure S218. Expansion of 2D-NMR (400 MHz, DMSO-d_6) HMQC experiment of *N*'-[(*E*)-(3,5-di-*tert*-butyl-2-hydroxyphenyl)methylidene]-4-hydroxybenzohydrazide (**3c**).

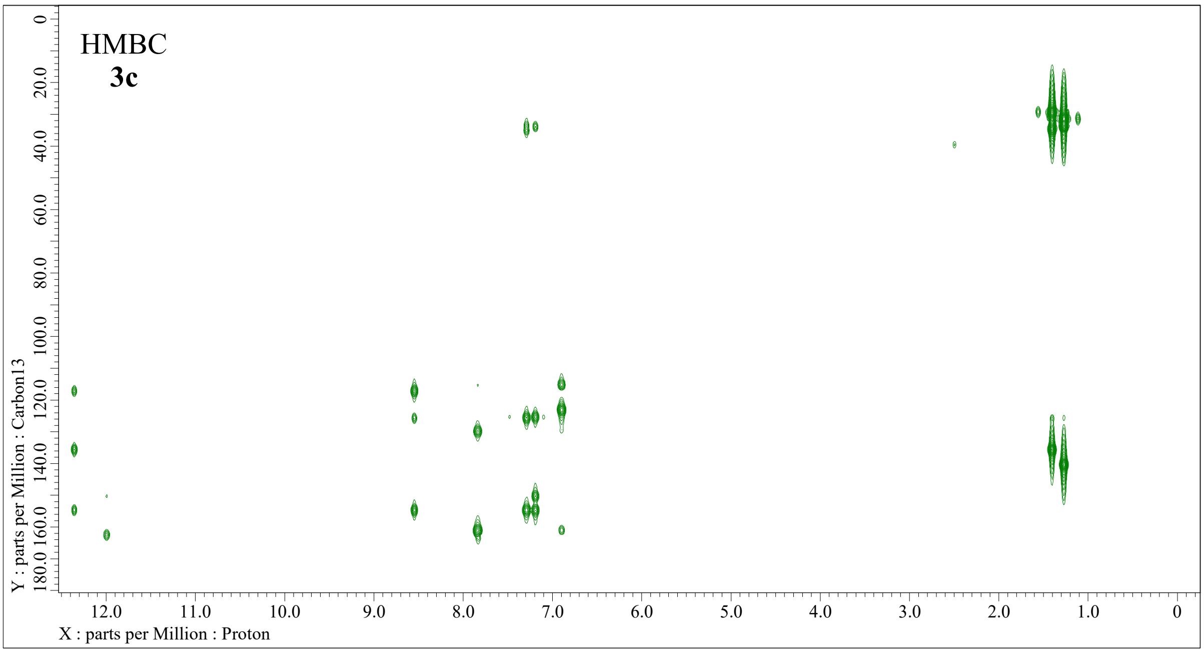

Figure S219. 2D-NMR (400 MHz, DMSO-d_6) HMBC experiment of *N*'-[(*E*)-(3,5-di-*tert*-butyl-2-hydroxyphenyl)methylidene]-4-hydroxybenzohydrazide (**3c**).

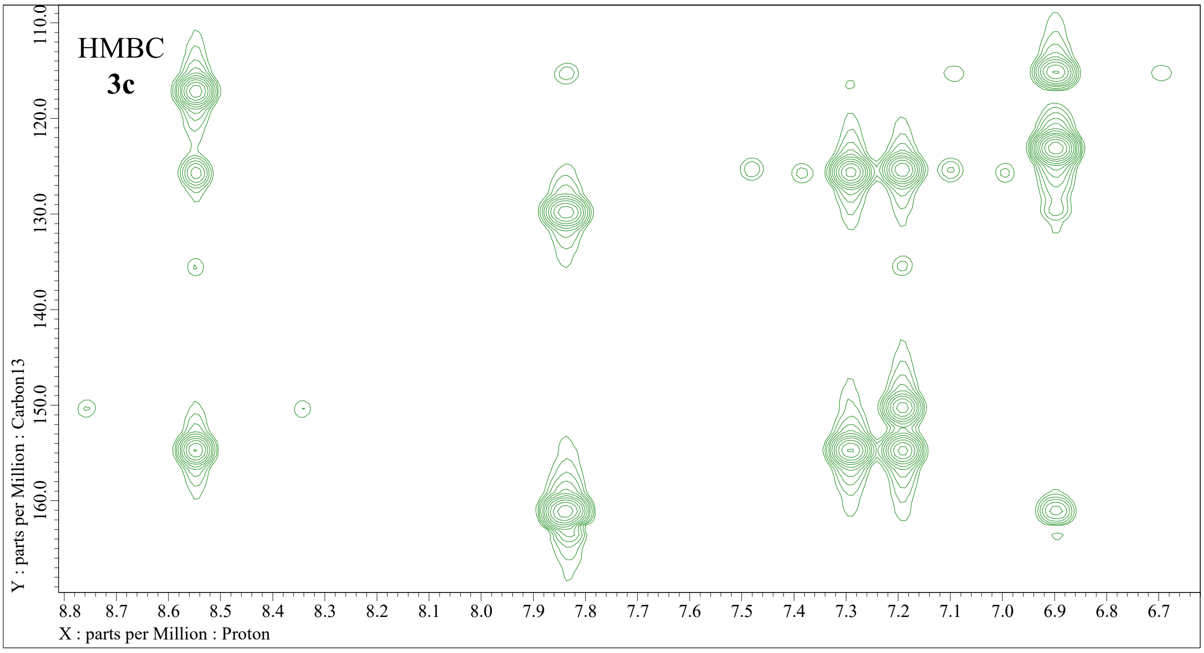

Figure S220. Expansion of 2D-NMR (400 MHz, DMSO-d_6) HMBC experiment of *N*'-[(*E*)-(3,5-di-*tert*-butyl-2-hydroxyphenyl)methylidene]-4-hydroxybenzohydrazide (**3c**).

Molecules **2020**, *25*, 1255; doi:10.3390/molecules25051255 www.mdpi.com/journal/molecules

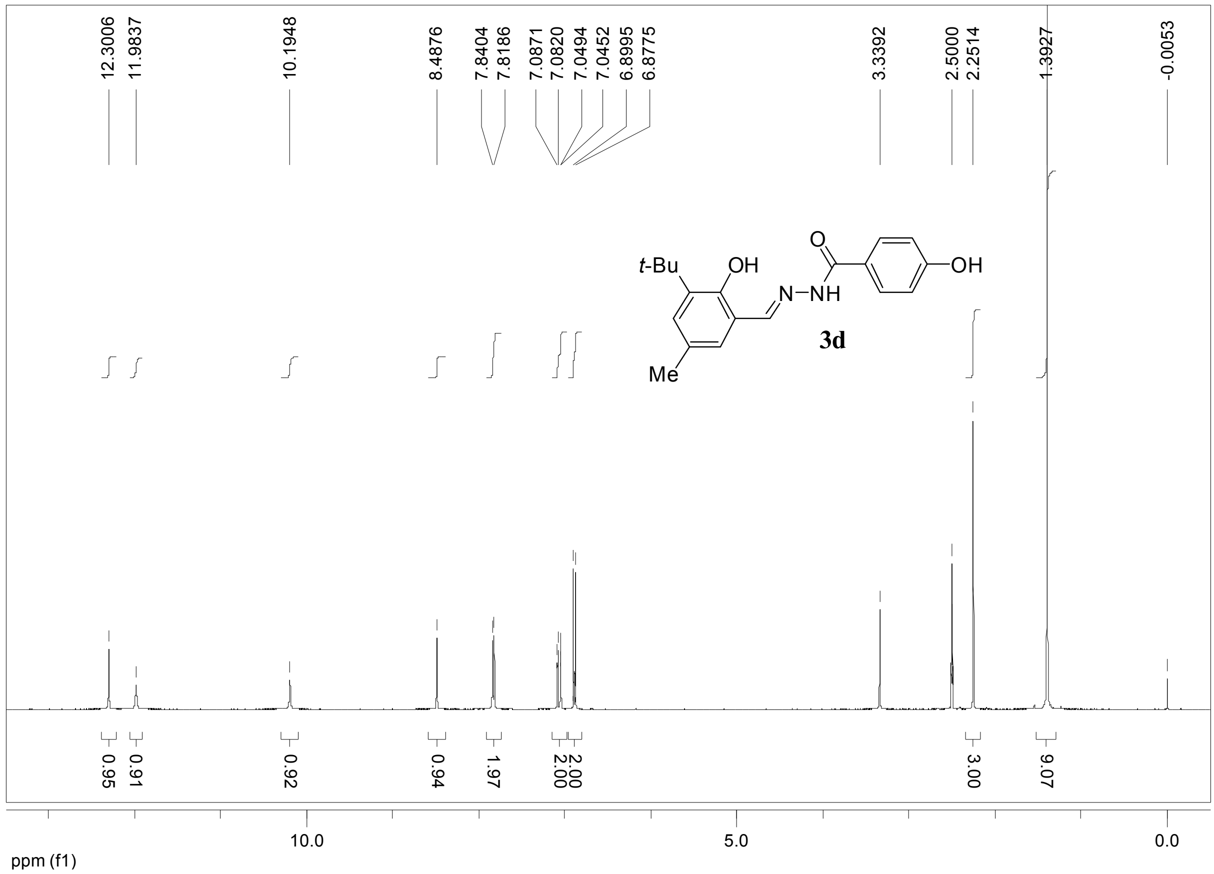

Figure S221. ¹H-NMR (400 MHz, DMSO-d_6) spectrum of compound **3d**.

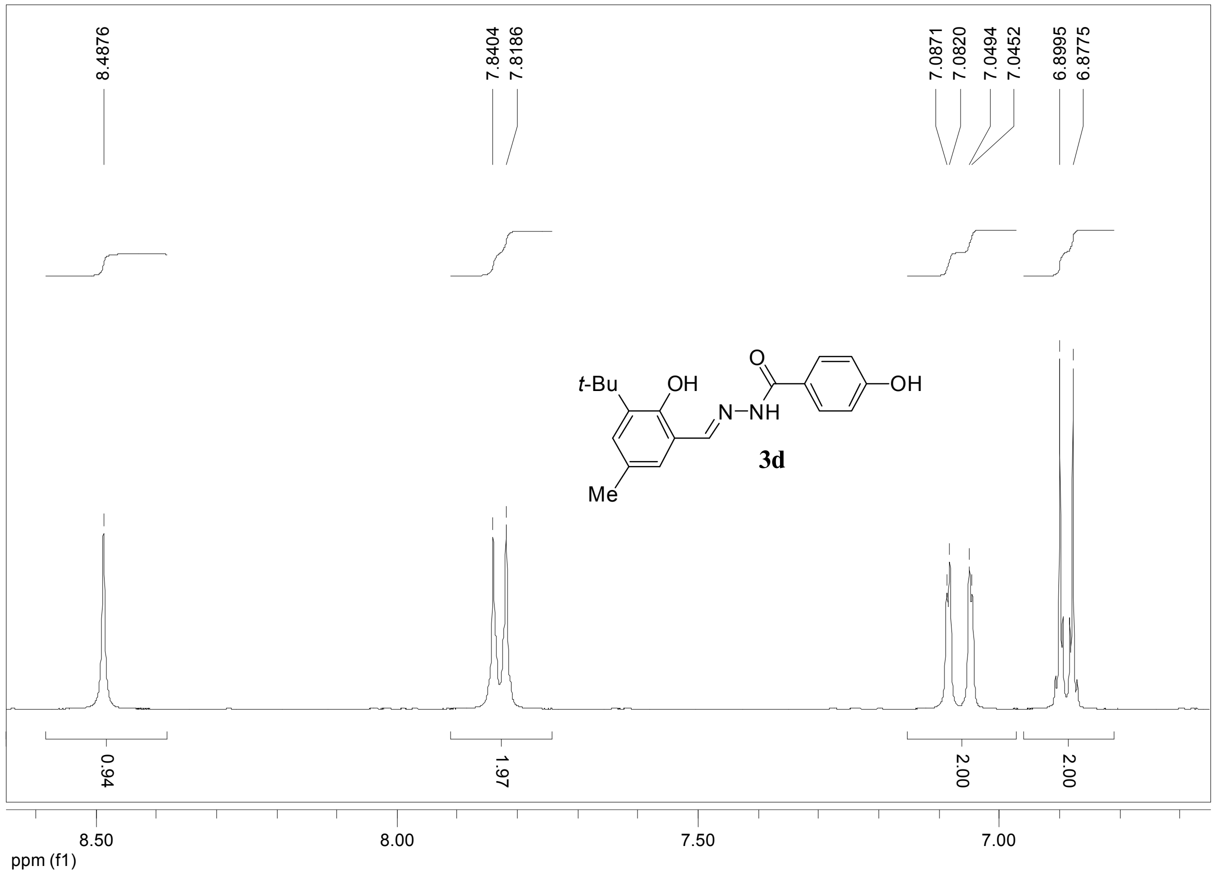

Figure S222. Expansion of ¹H-NMR (400 MHz, DMSO-d_6) spectrum of compound **3d**.

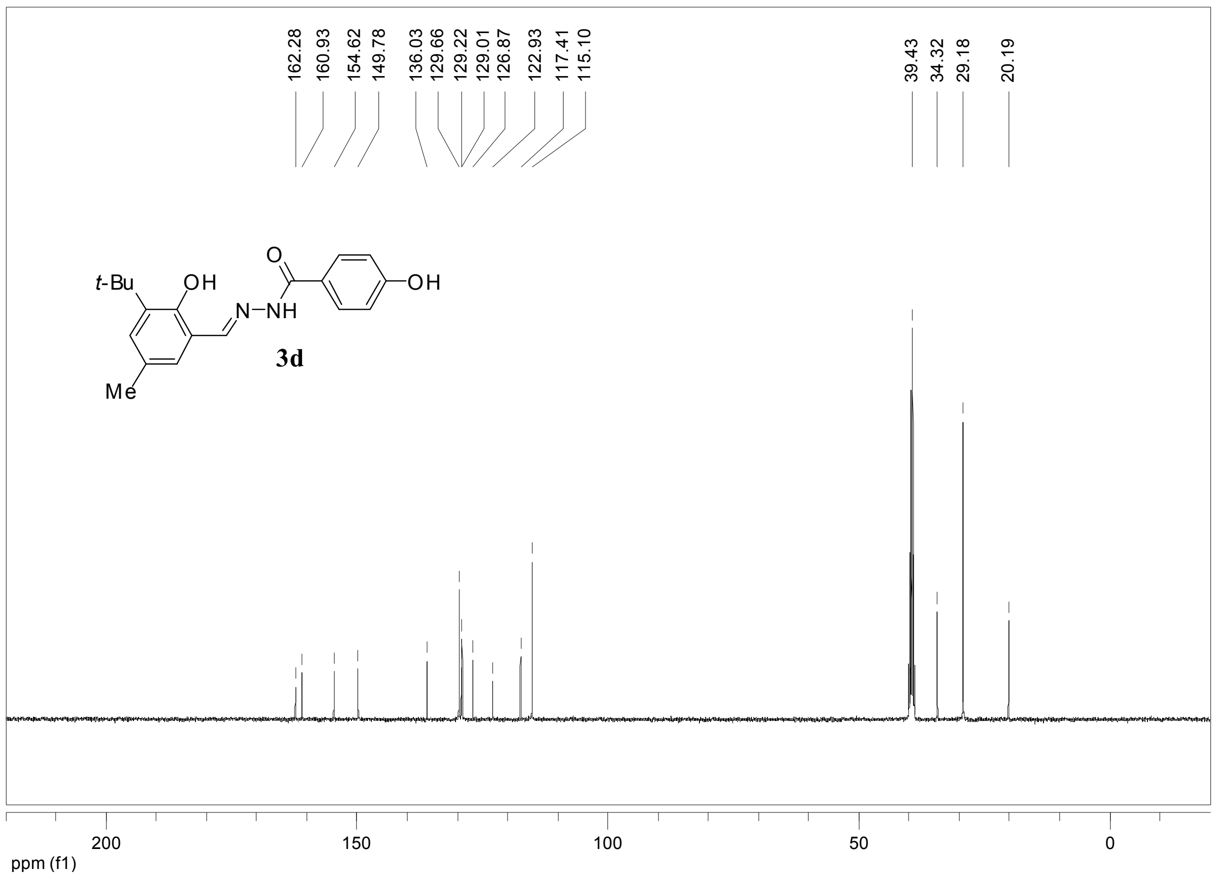

Figure S223. ^{13}C-NMR (100 MHz, DMSO-d_6) spectrum of compound **3d**.

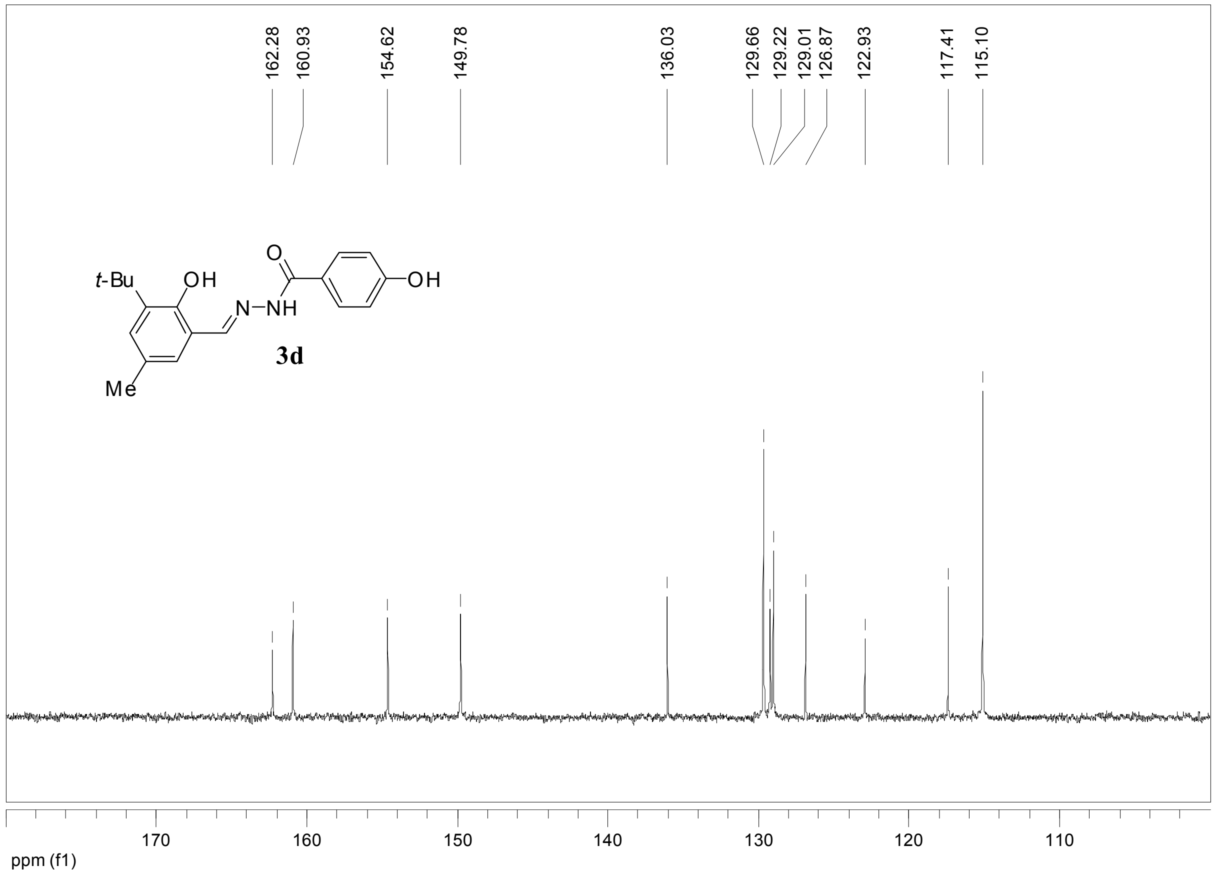

Figure S224. Expansion of ^{13}C-NMR (100 MHz, DMSO-d_6) spectrum of compound **3d**.

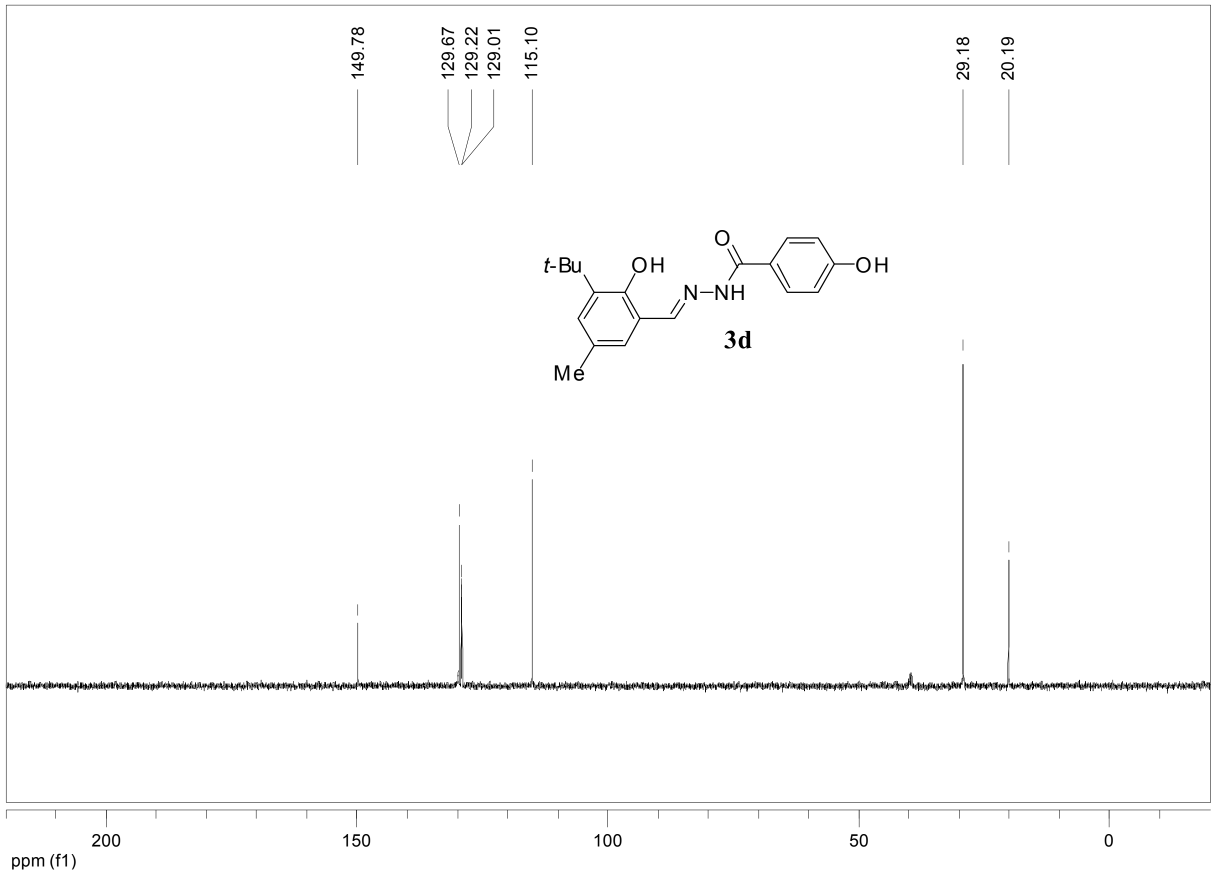

Figure S225. ^{13}C-NMR (100 MHz, DMSO-d_6) dept-135 experiment of compound **3d**.

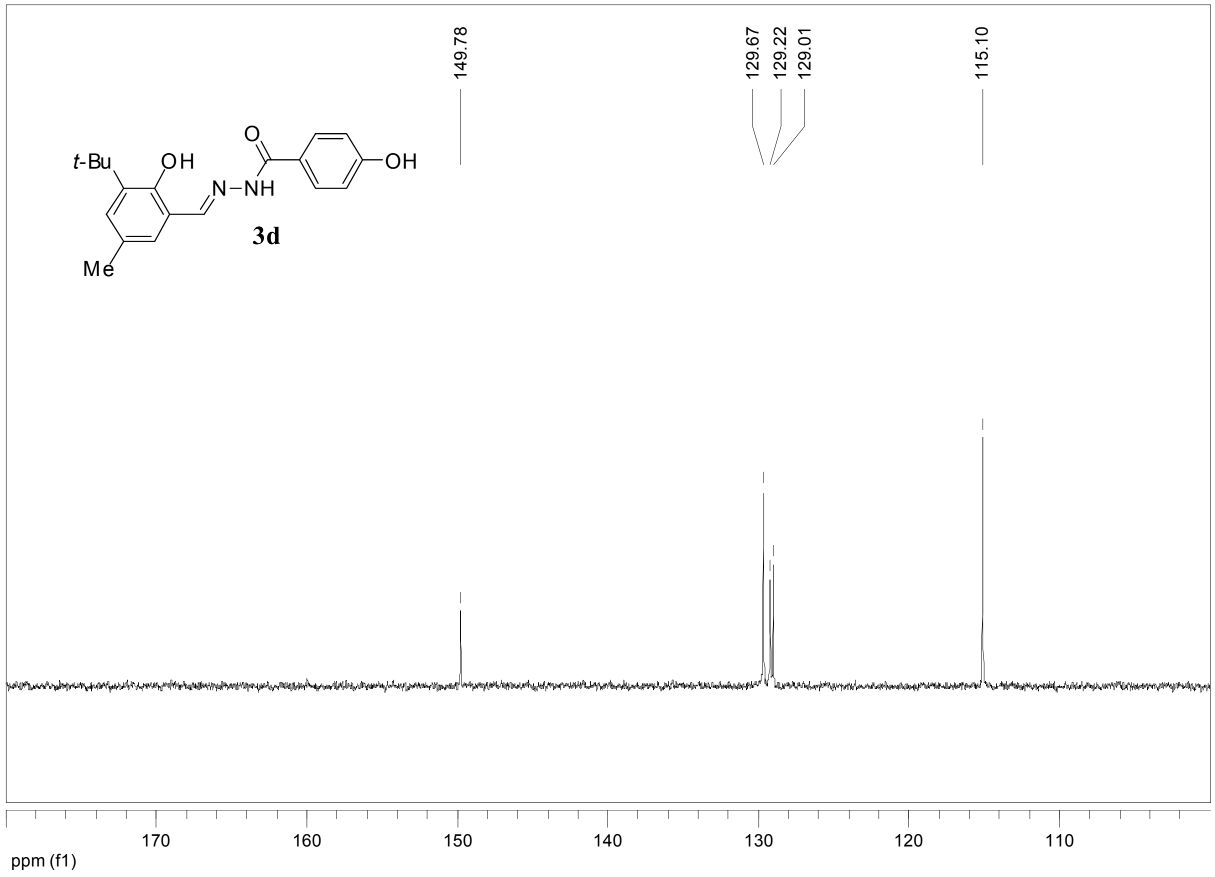

Figure S226. Expansion of ^{13}C-NMR (100 MHz, DMSO-d_6) dept-135 experiment of compound **3d**.

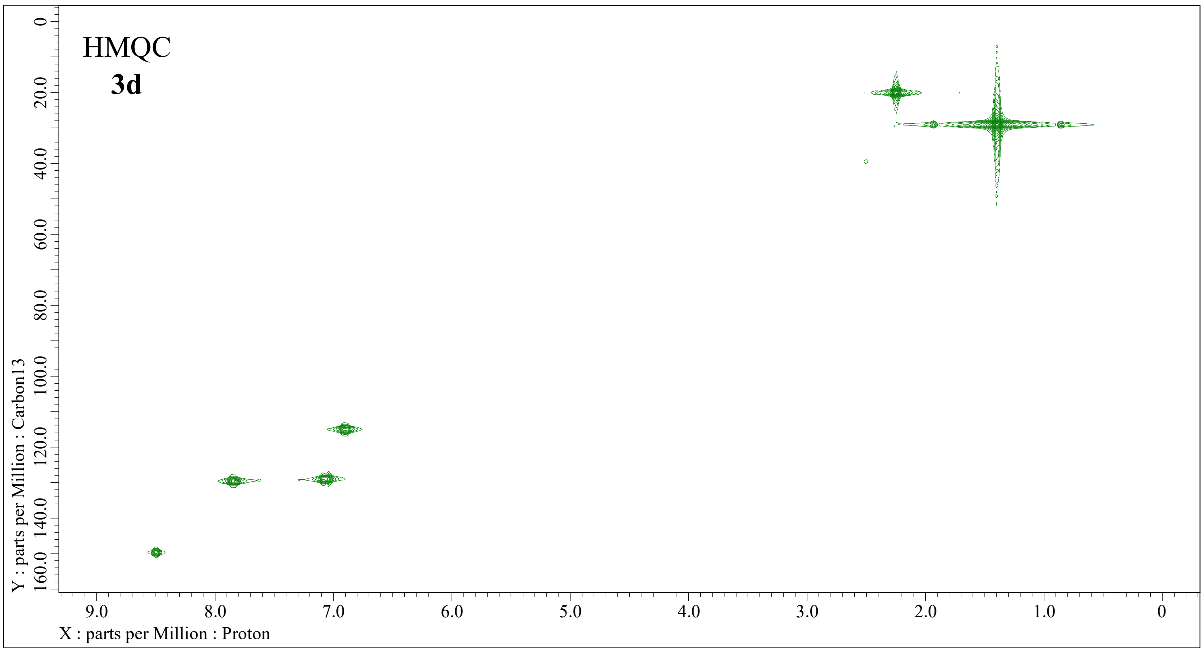

Figure S227. 2D-NMR (400 MHz, DMSO-d_6) HMQC experiment of 4-hydroxy-*N*'-[(*E*)-3-*tert*-butyl-2-hydroxy-5-methylbenzylidene]benzohydrazide (**3d**).

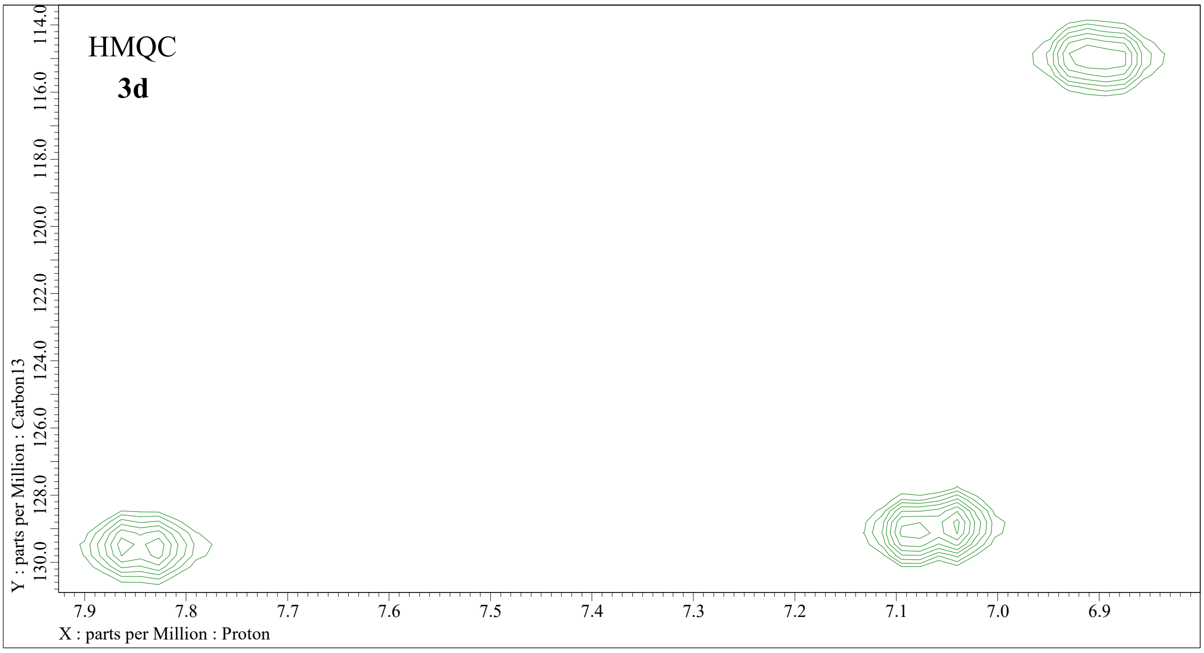

Figure S228. Expansion of 2D-NMR (400 MHz, DMSO-d_6) HMQC experiment of 4-hydroxy-*N*'-[(*E*)-3-*tert*-butyl-2-hydroxy-5-methylbenzylidene]benzohydrazide (**3d**).

Molecules **2020**, *25*, 1255; doi:10.3390/molecules25051255 www.mdpi.com/journal/molecules

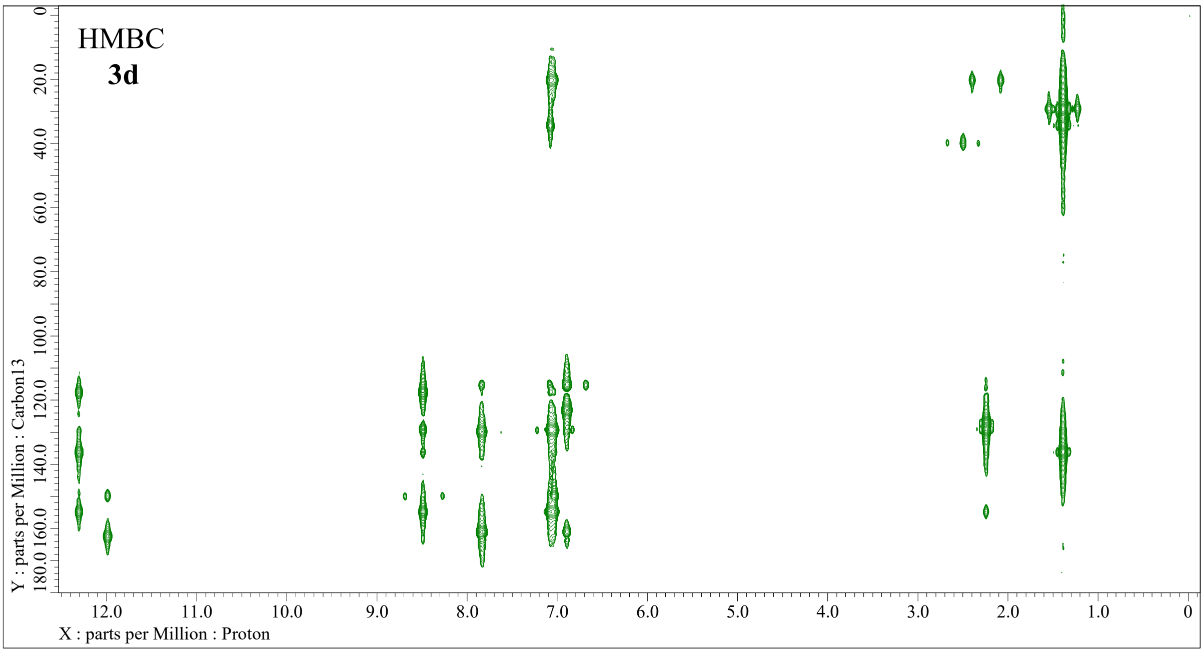

Figure S229. 2D-NMR (400 MHz, DMSO-d_6) HMBC experiment of 4-hydroxy-*N*'-[(*E*)-3-*tert*-butyl-2-hydroxy-5-methylbenzylidene]benzohydrazide (**3d**).

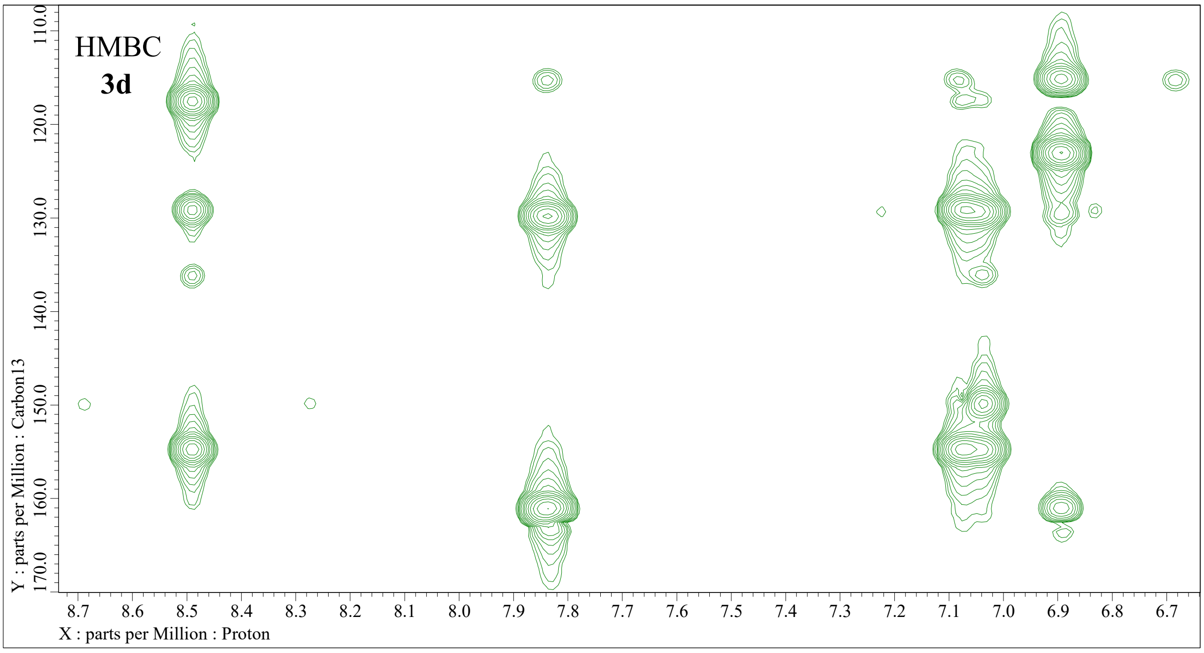

Figure S230. Expansion of 2D-NMR (400 MHz, DMSO-d_6) HMBC experiment of 4-hydroxy-*N*'-[(*E*)-3-*tert*-butyl-2-hydroxy-5-methylbenzylidene]benzohydrazide (**3d**).

Molecules **2020**, *25*, 1255; doi:10.3390/molecules25051255 www.mdpi.com/journal/molecules

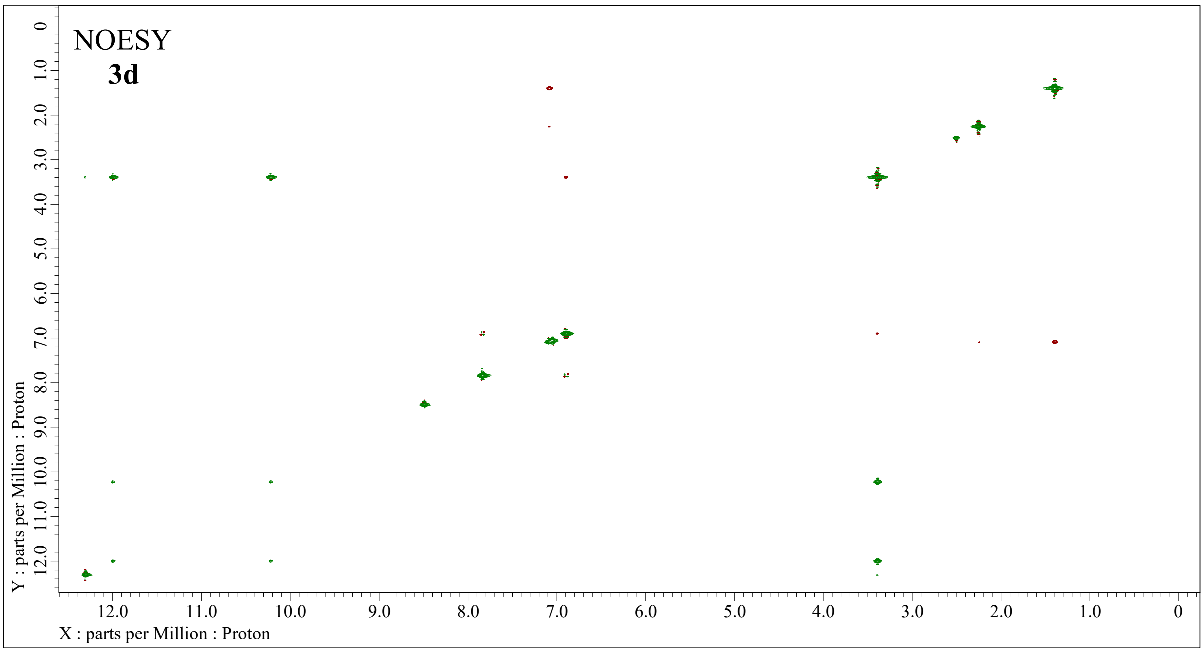

Figure S231. 2D-NMR (400 MHz, DMSO-d_6) NOESY experiment of 4-hydroxy-*N*'-[(*E*)-3-*tert*-butyl-2-hydroxy-5-methylbenzylidene]benzohydrazide (**3d**).

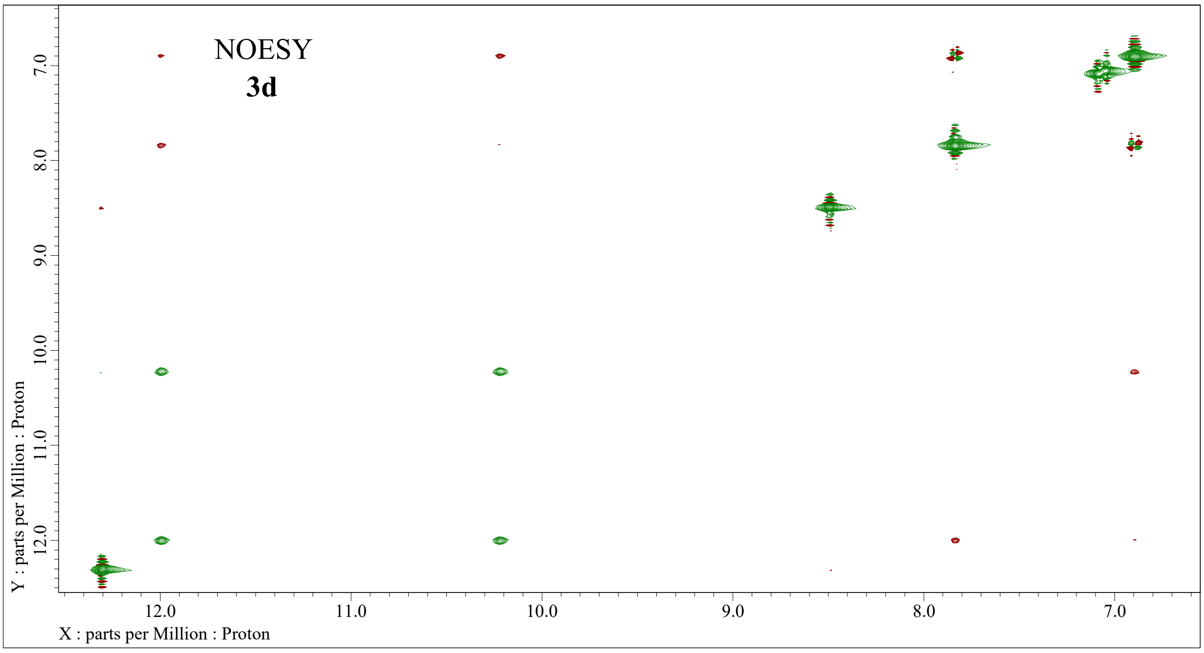

Figure S232. Expansion of 2D-NMR (400 MHz, DMSO-d_6) NOESY experiment of 4-hydroxy-*N*'-[(*E*)-3-*tert*-butyl-2-hydroxy-5-methylbenzylidene]benzohydrazide (**3d**).

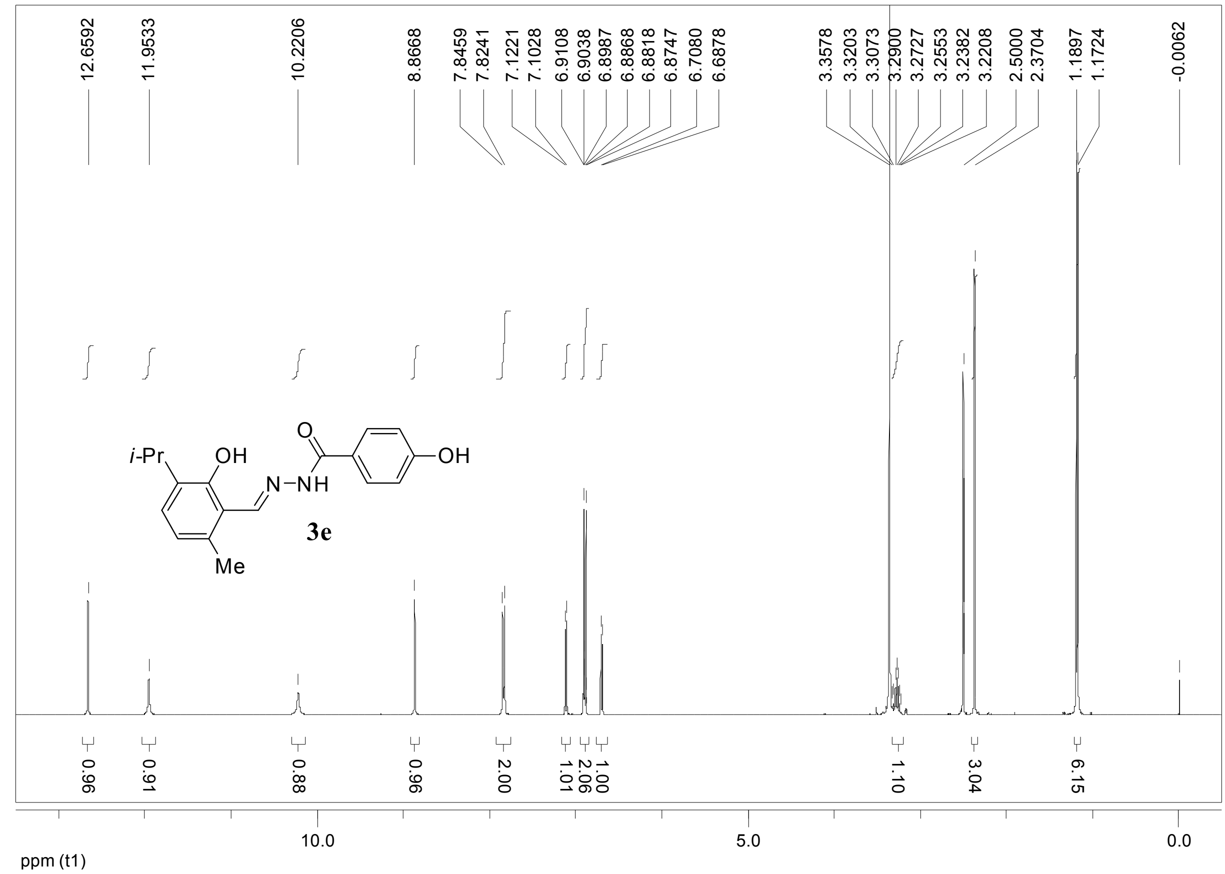

Figure S233. ^{1}H-NMR (400 MHz, DMSO-d_6) spectrum of compound **3e**.

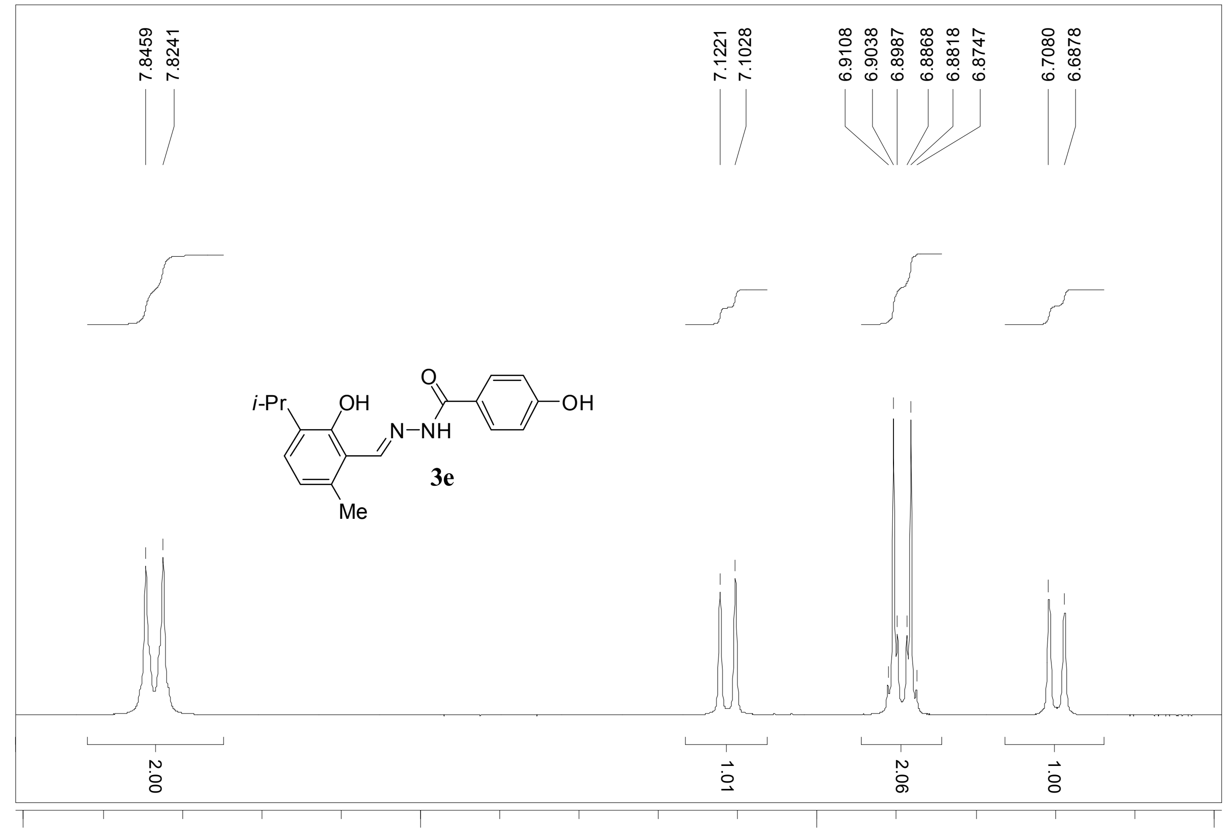

Figure S234. Expansion of ^{1}H-NMR (400 MHz, DMSO-d_6) spectrum of compound **3e**.

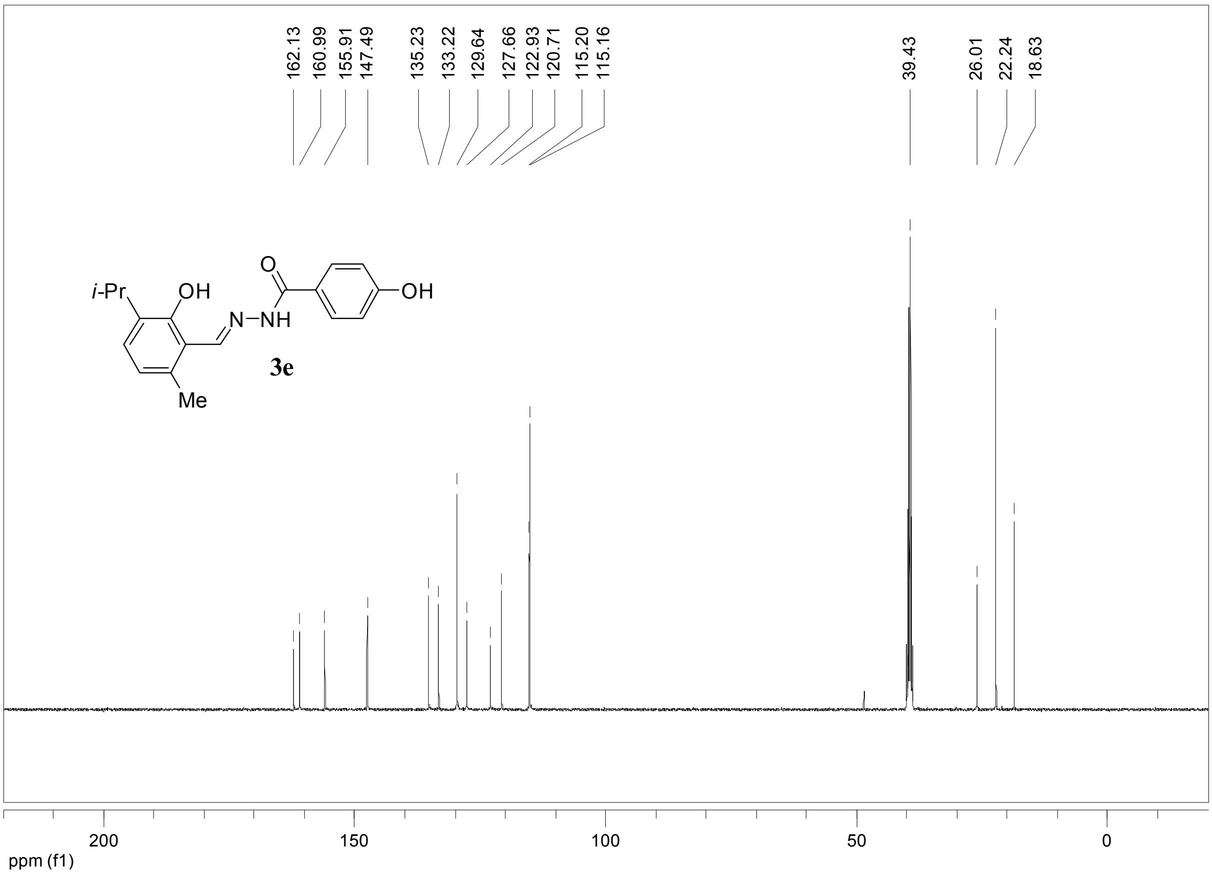

Figure S235. ^{13}C-NMR (100 MHz, DMSO-d_6) spectrum of compound **3e**.

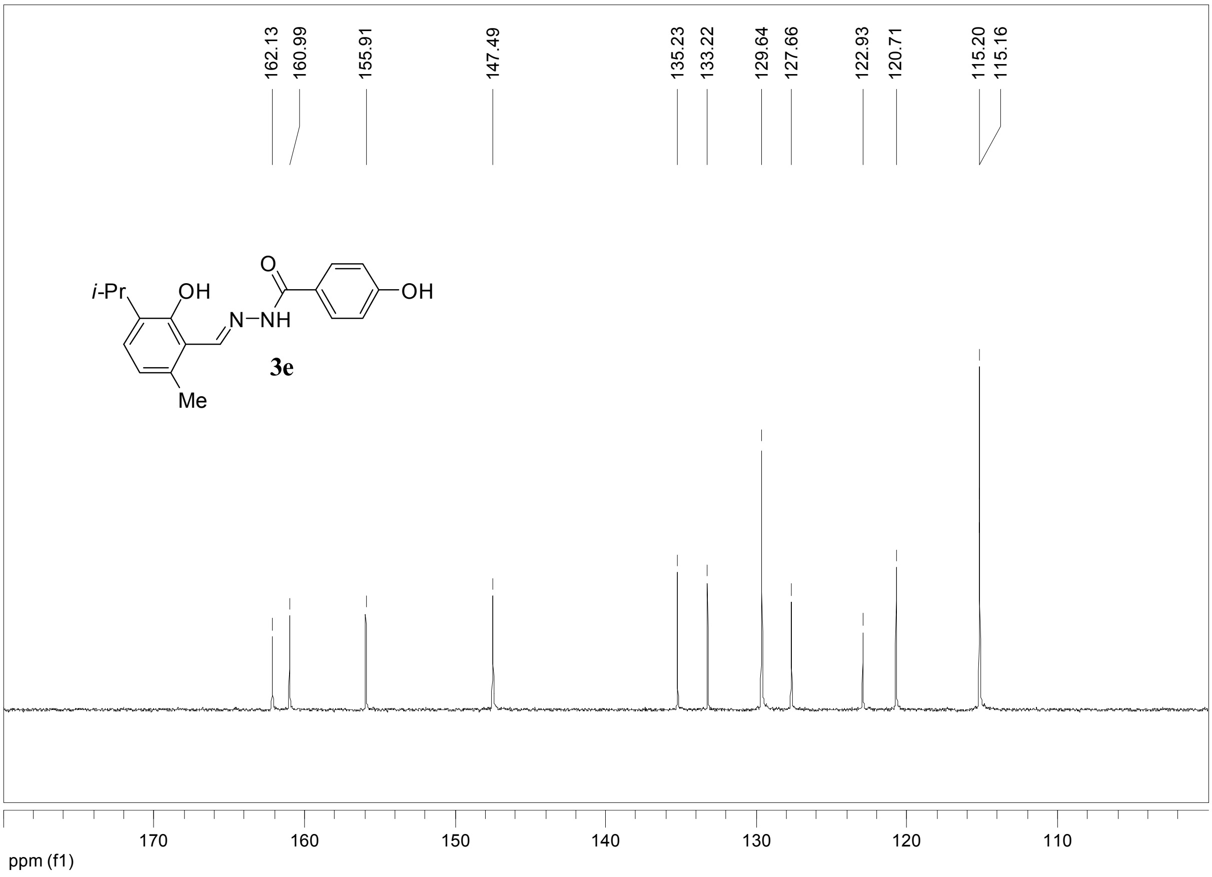

Figure S236. Expansion of ^{13}C-NMR (100 MHz, DMSO-d_6) spectrum of compound **3e**.

Molecules **2020**, *25*, 1255; doi:10.3390/molecules25051255 www.mdpi.com/journal/molecules

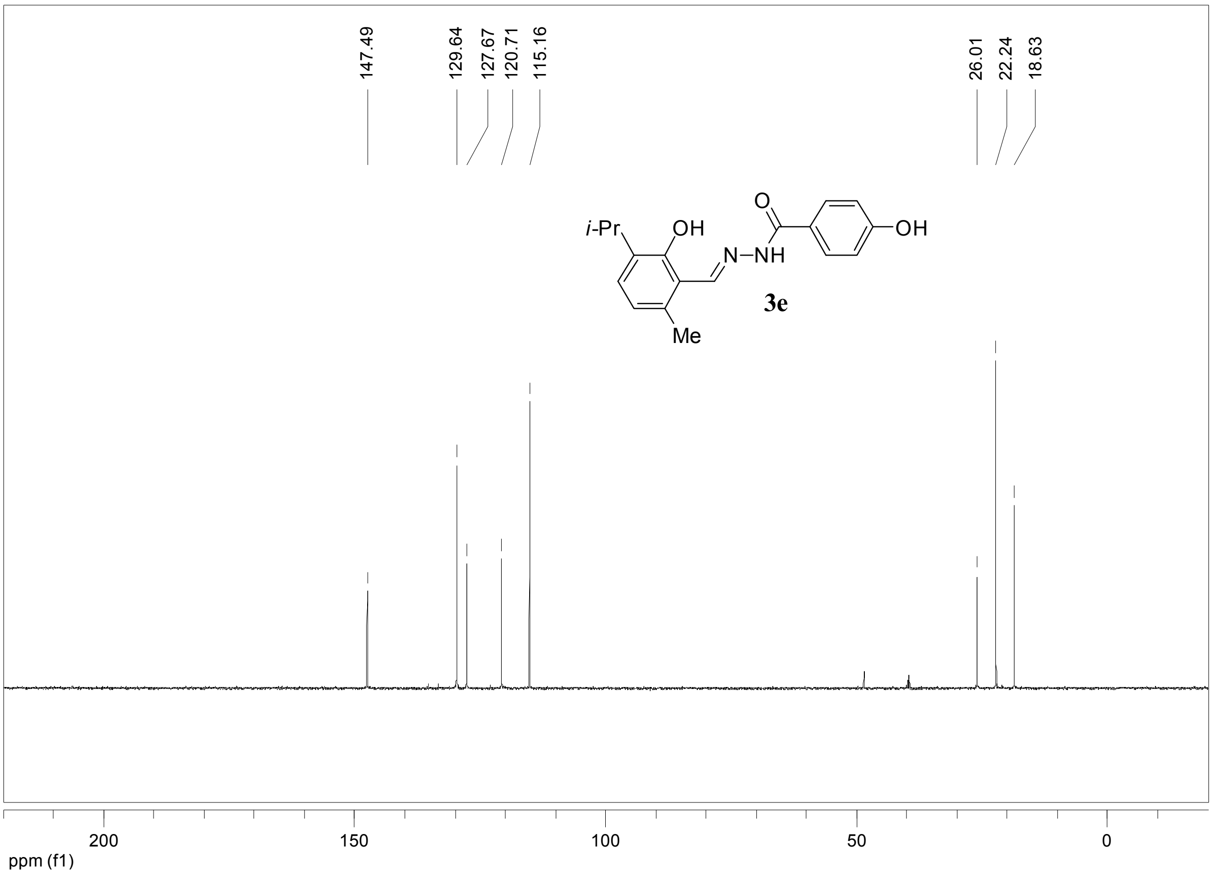

Figure S237. ^{13}C-NMR (100 MHz, DMSO-d_6) dept 135 experiment of compound **3e**.

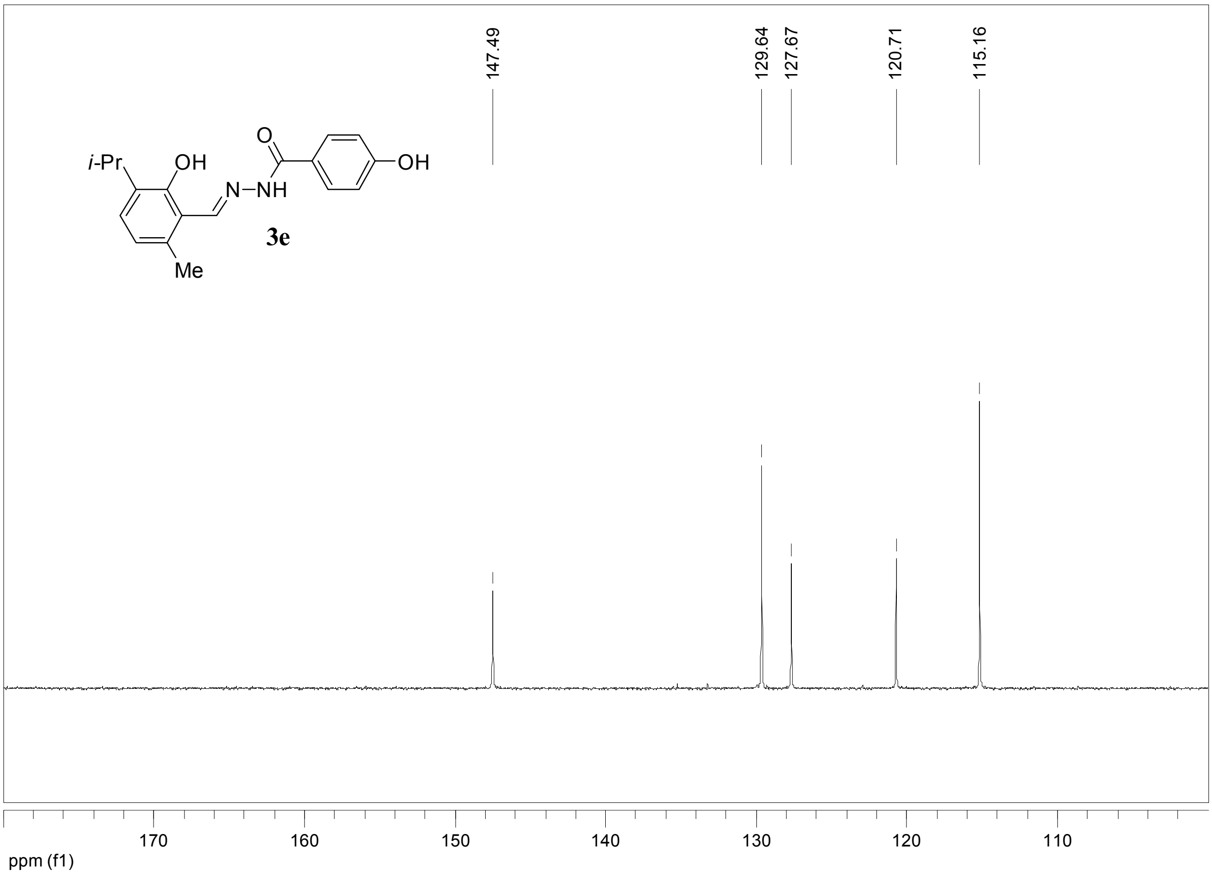

Figure S238. Expansion of ^{13}C-NMR (100 MHz, DMSO-d_6) dept 135 experiment of compound **3e**.

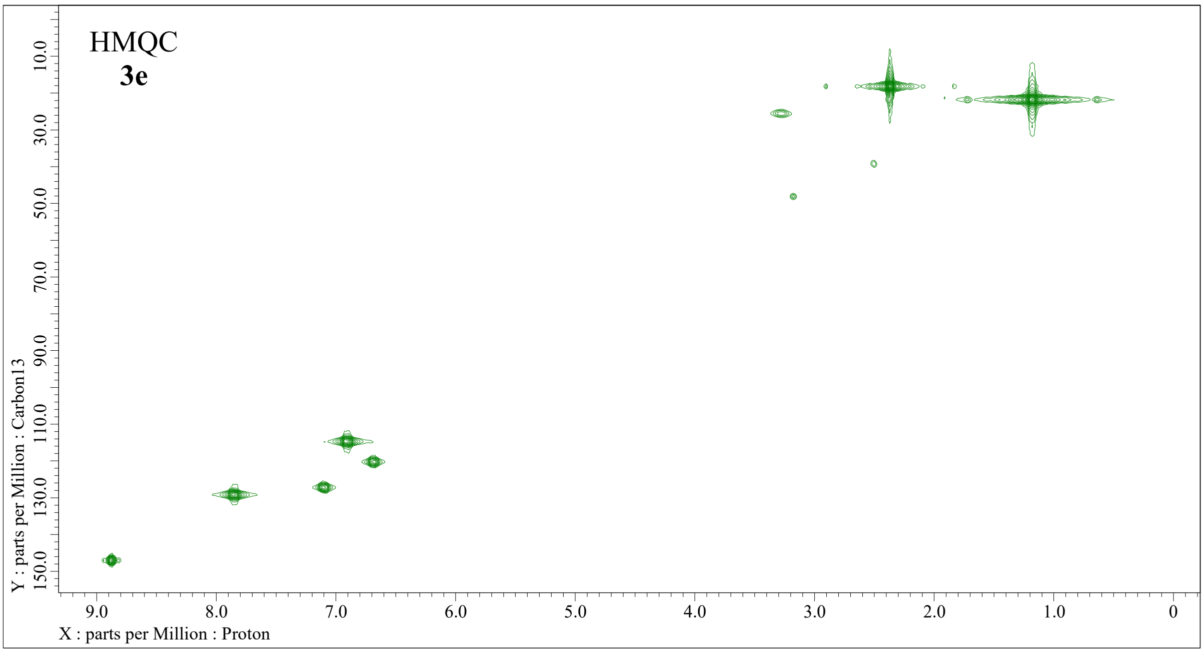

Figure S239. 2D-NMR (400 MHz, DMSO-d_6) HMQC experiment of 4-hydroxy-*N*'-[(*E*)-(2-hydroxy-3-isopropyl-5-methylphenyl)methylidene]benzohydrazide (**3e**).

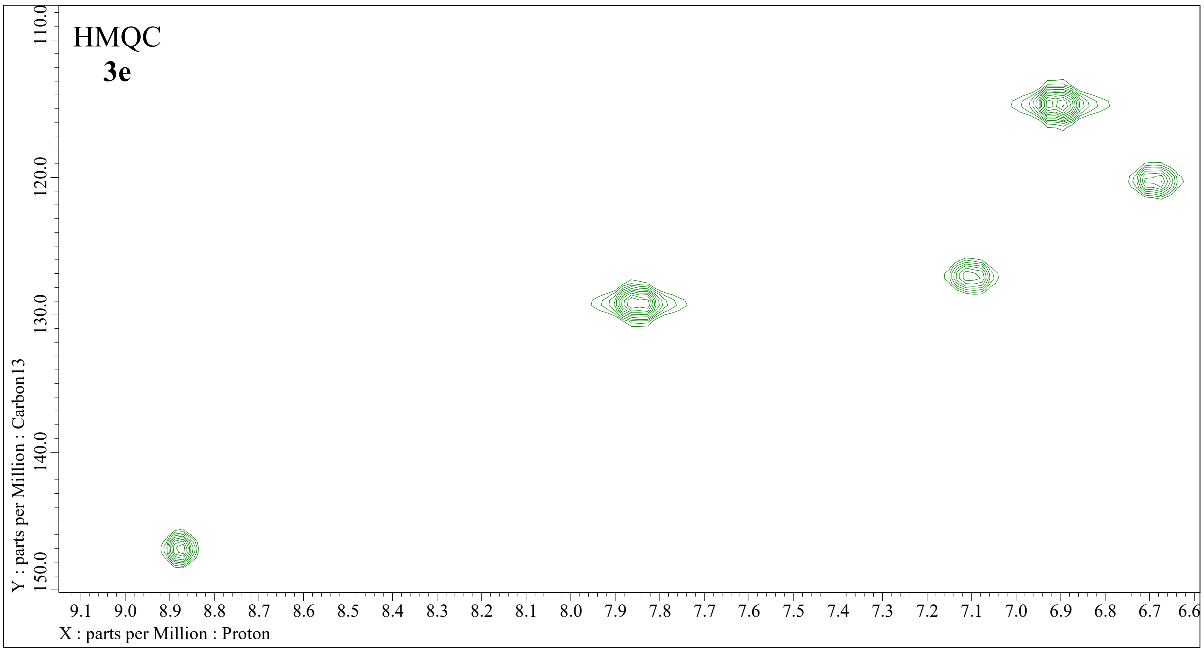

Figure S240. Expansion of 2D-NMR (400 MHz, DMSO-d_6) HMQC experiment of 4-hydroxy-*N*'-[(*E*)-(2-hydroxy-3-isopropyl-5-methylphenyl)methylidene]benzohydrazide (**3e**).

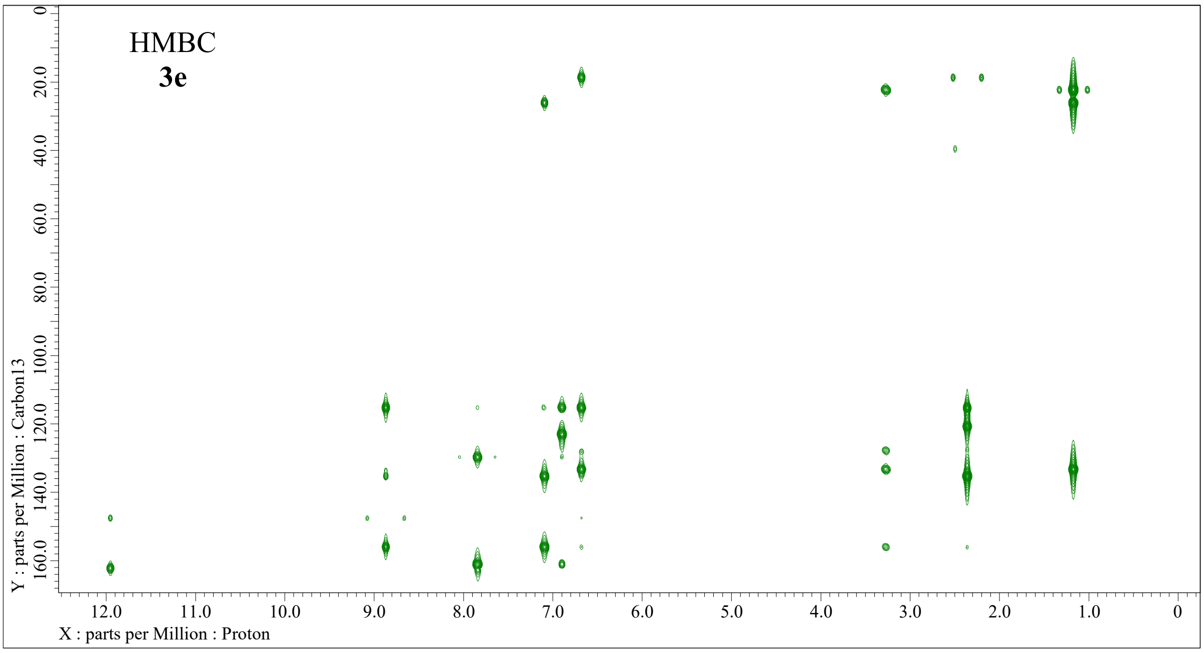

Figure S241. 2D-NMR (400 MHz, DMSO-d_6) HMBC experiment of 4-hydroxy-*N*'-[(*E*)-(2-hydroxy-3-isopropyl-5-methylphenyl)methylidene]benzohydrazide (**3e**).

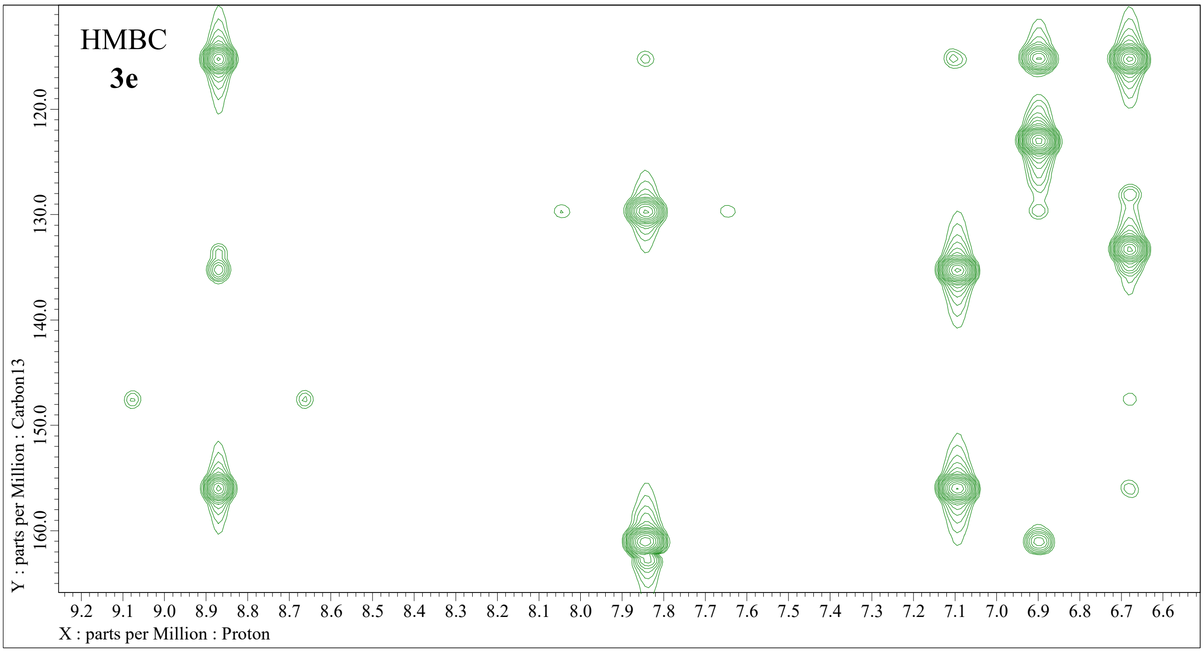

Figure S242. Expansion of 2D-NMR (400 MHz, DMSO-d_6) HMBC experiment of 4-hydroxy-*N*'-[(*E*)-(2-hydroxy-3-isopropyl-5-methylphenyl)methylidene]benzohydrazide (**3e**).

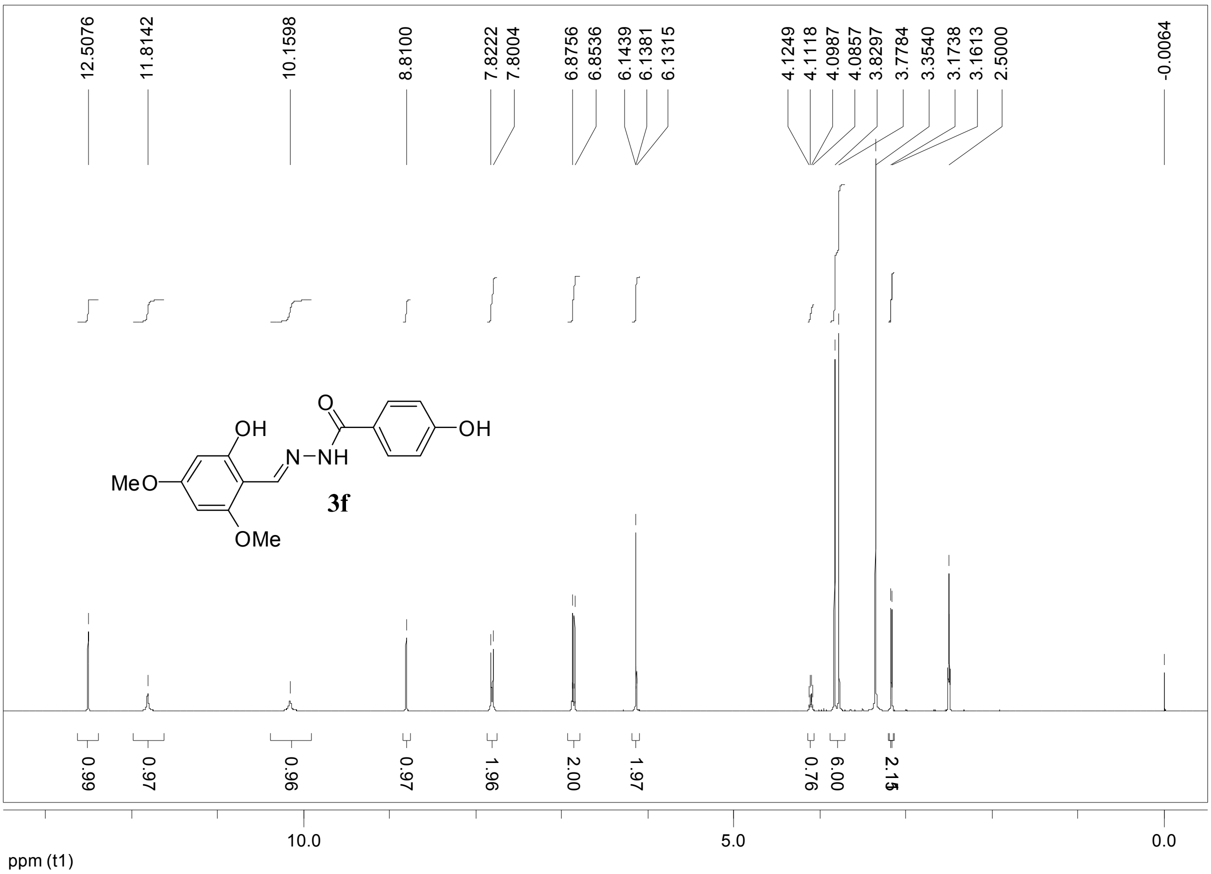

Figure S243. ¹H-NMR (400 MHz, DMSO-d_6) spectrum of compound **3f**.

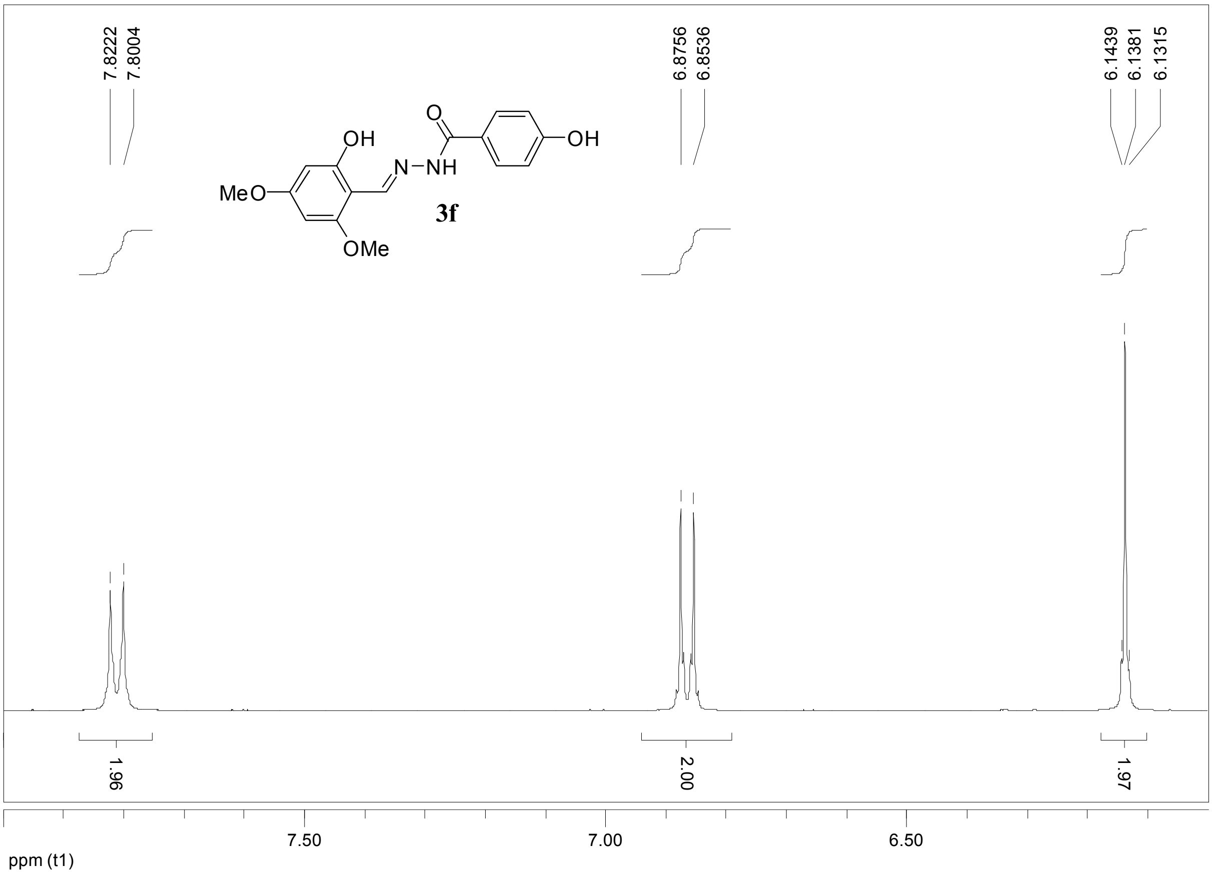

Figure S244. Expansion of ¹H-NMR (400 MHz, DMSO-d_6) spectrum of compound **3f**.

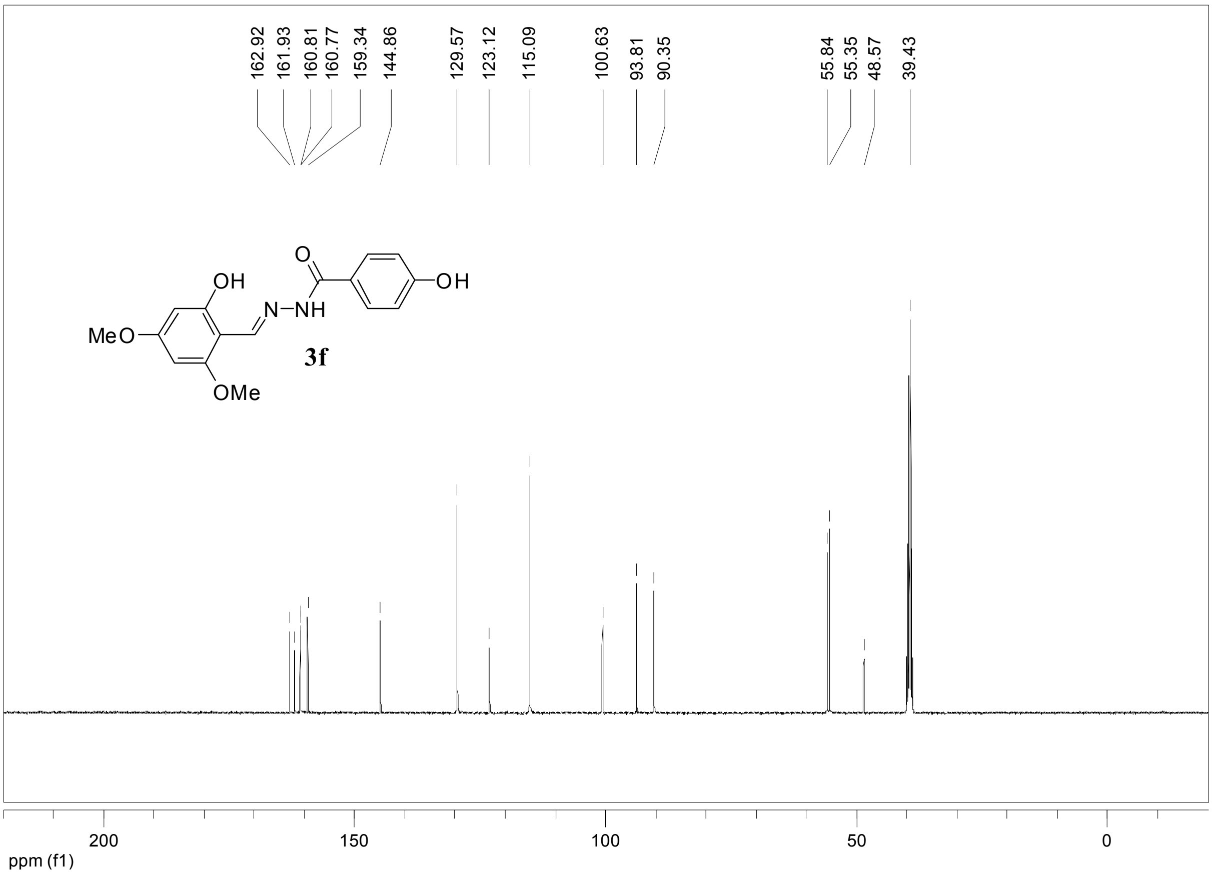

Figure S245. ^{13}C-NMR (100 MHz, DMSO-d_6) spectrum of compound **3f**.

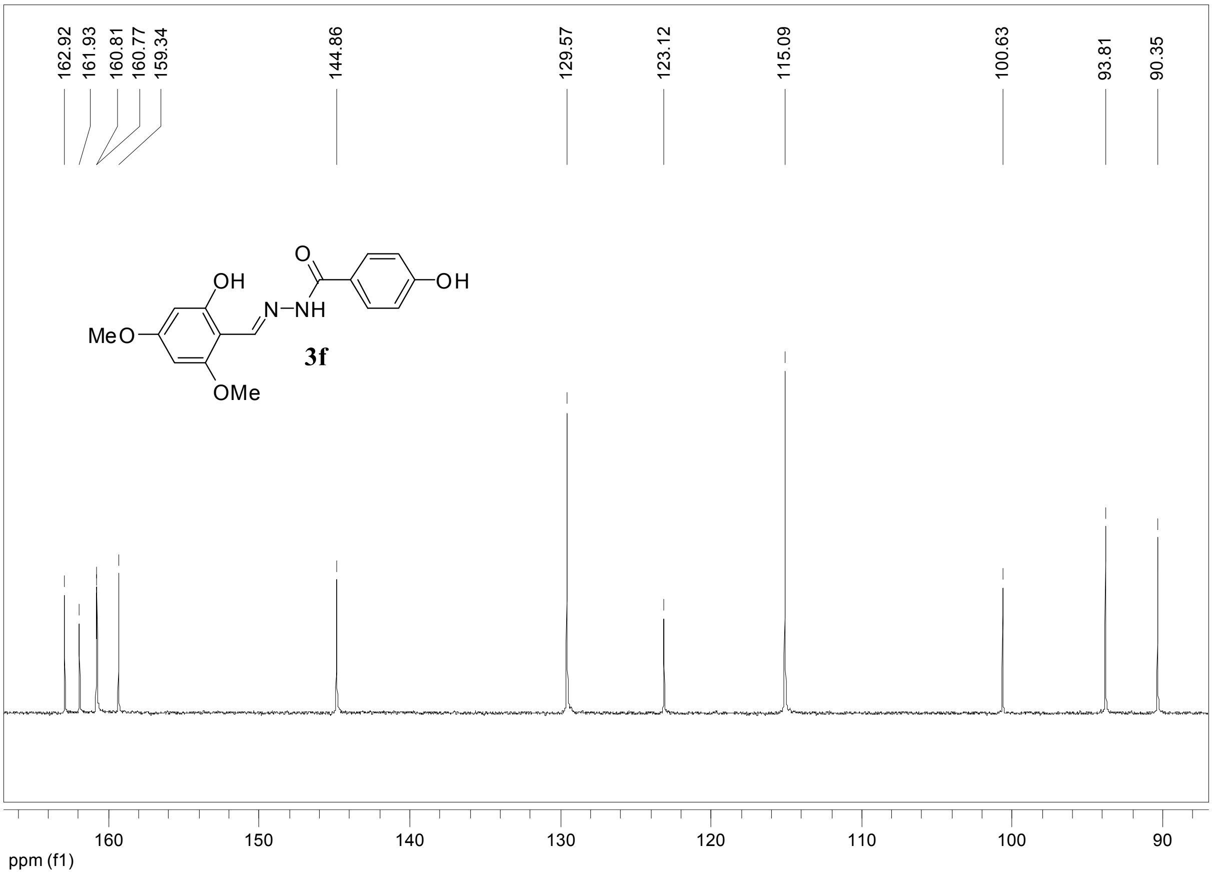

Figure S246. Expansion of ^{13}C-NMR (100 MHz, DMSO-d_6) spectrum of compound **3f**.

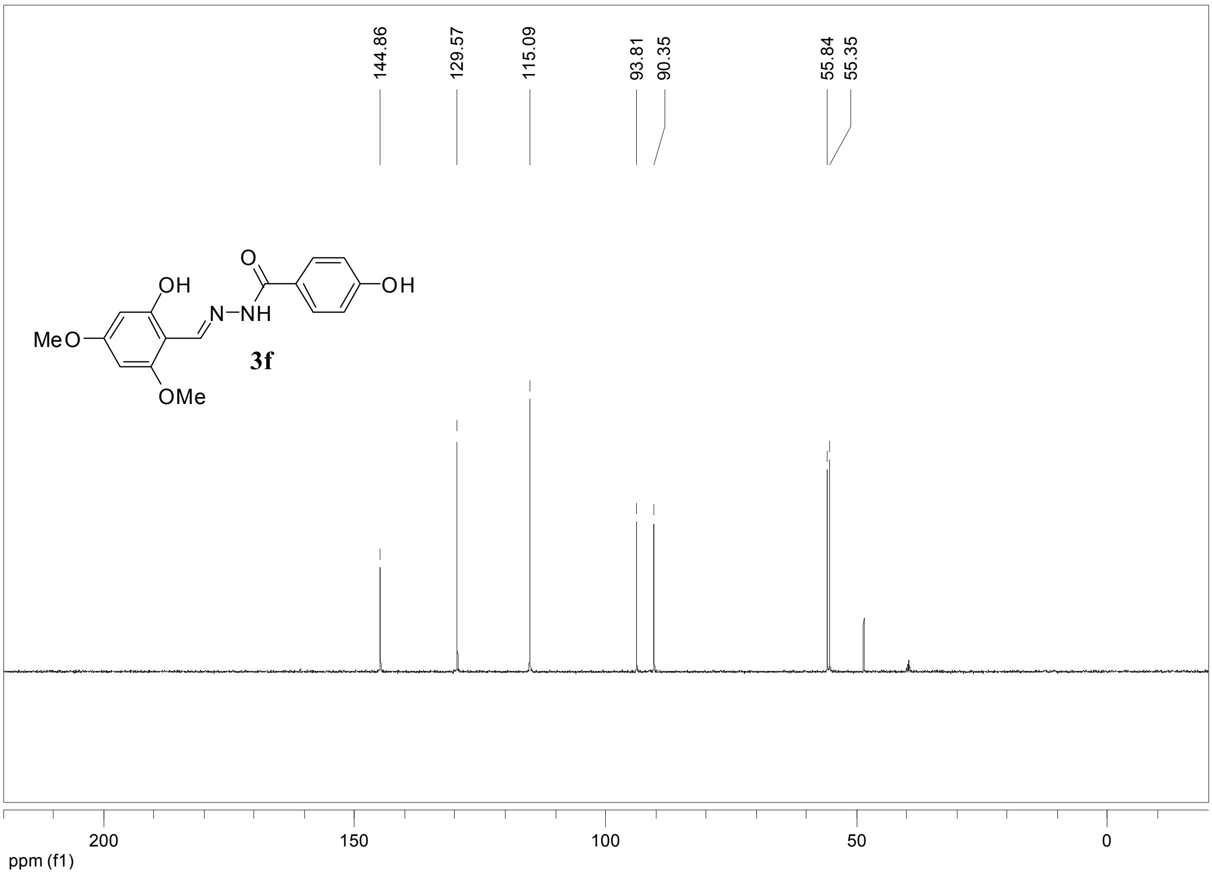

Figure S247. ^{13}C-NMR (100 MHz, DMSO-d_6) dept-135 experiment of compound **3f**.

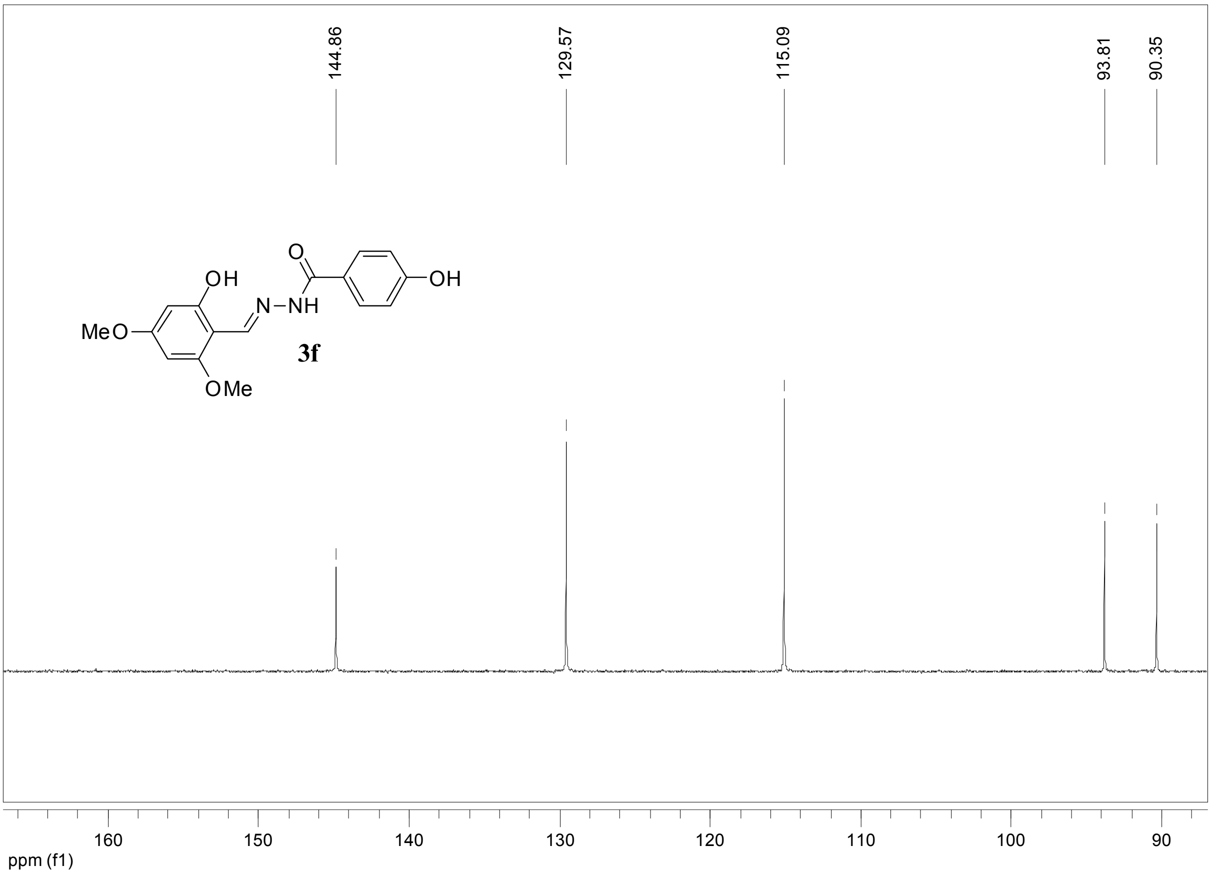

Figure S248. Expansion of ^{13}C-NMR (100 MHz, DMSO-d_6) dept-135 experiment of compound **3f**.

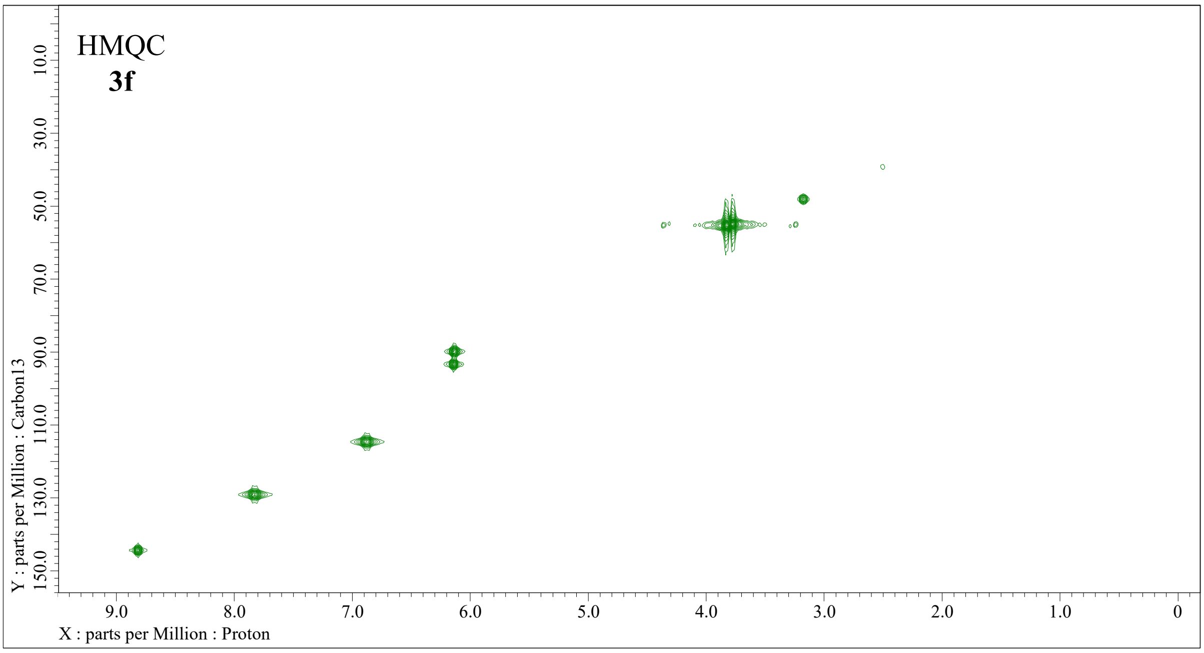

Figure S249. 2D-NMR (400 MHz, DMSO-d_6) HMQC experiment of 4-hydroxy-*N*'-[(*E*)-(2-hydroxy-4,6-dimethoxyphenyl)methylidene]benzohydrazide (**3f**).

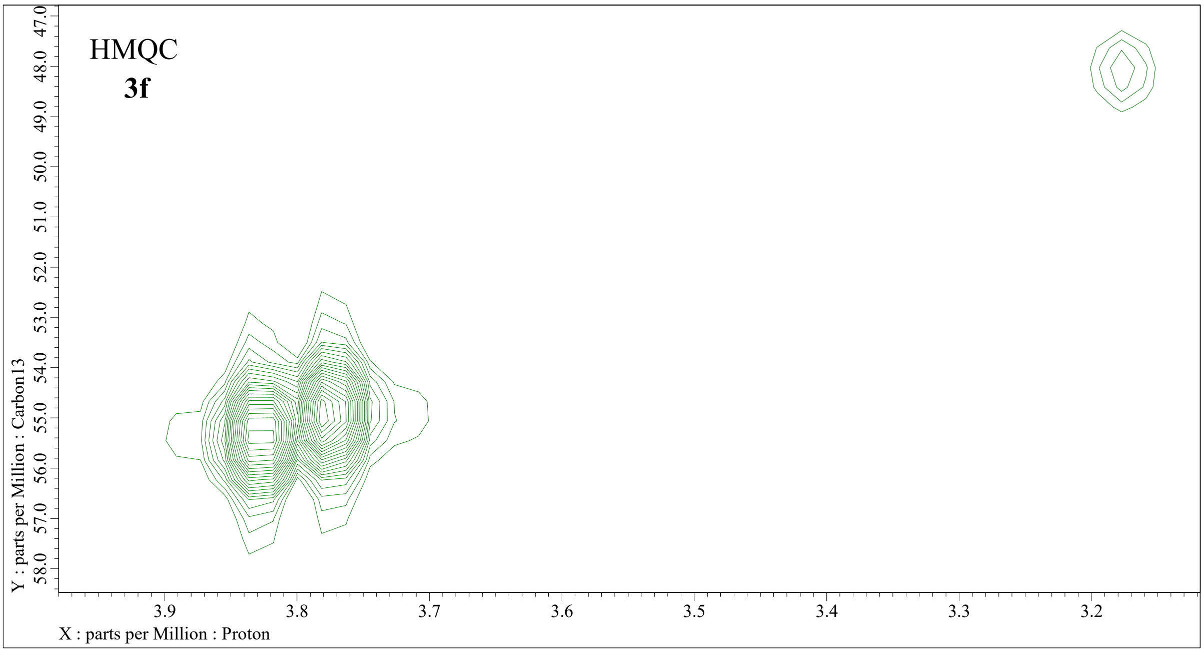

Figure S250. Expansion of 2D-NMR (400 MHz, DMSO-d_6) HMQC experiment of 4-hydroxy-*N*'-[(*E*)-(2-hydroxy-4,6-dimethoxyphenyl)methylidene]benzohydrazide (**3f**).

Molecules **2020**, *25*, 1255; doi:10.3390/molecules25051255 www.mdpi.com/journal/molecules

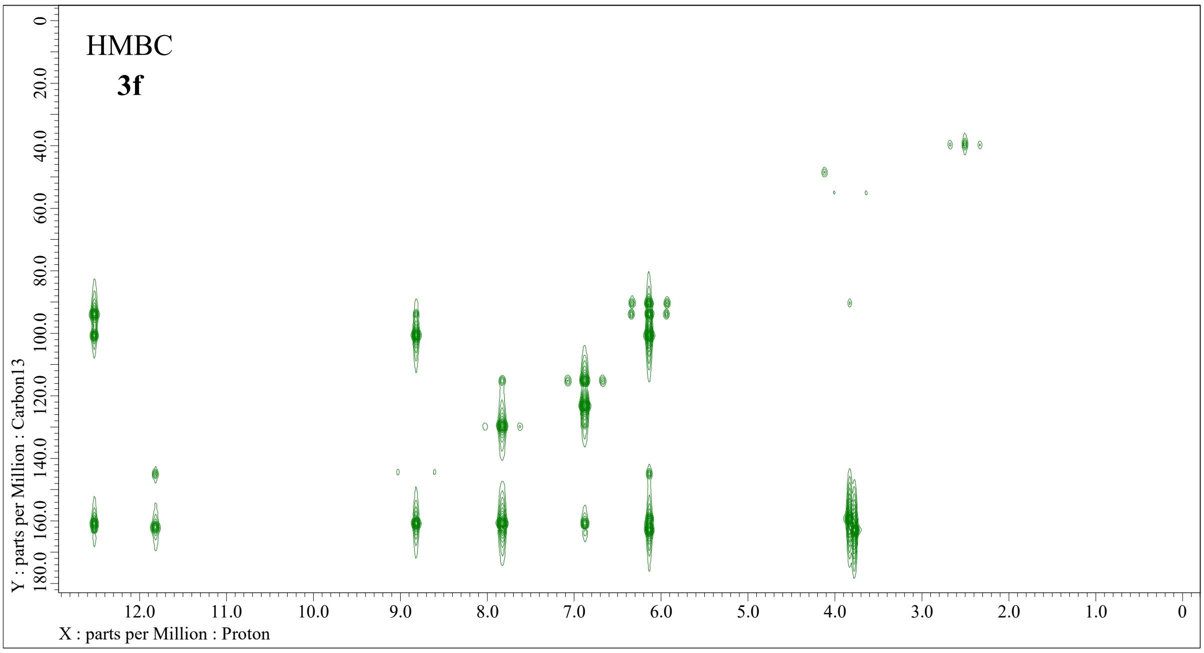

Figure S251. 2D-NMR (400 MHz, DMSO-d_6) HMBC experiment of 4-hydroxy-*N*'-[(*E*)-(2-hydroxy-4,6-dimethoxyphenyl)methylidene]benzohydrazide (**3f**).

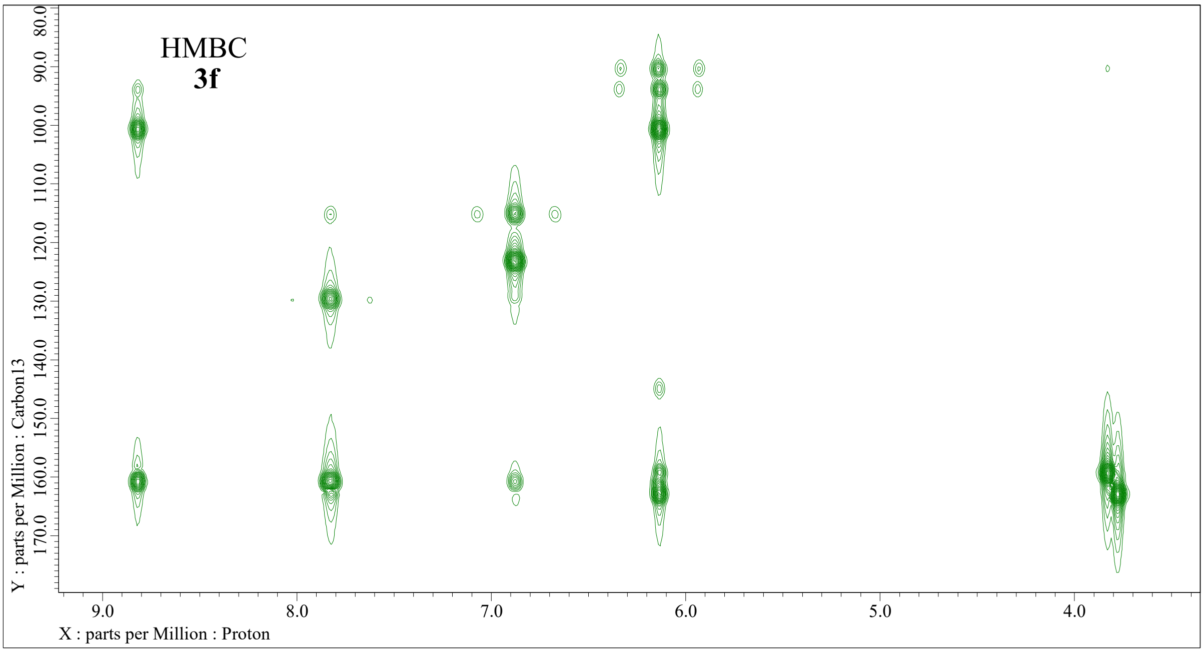

Figure S252. Expansion of 2D-NMR (400 MHz, DMSO-d_6) HMBC experiment of 4-hydroxy-*N*'-[(*E*)-(2-hydroxy-4,6-dimethoxyphenyl)methylidene]benzohydrazide (**3f**).

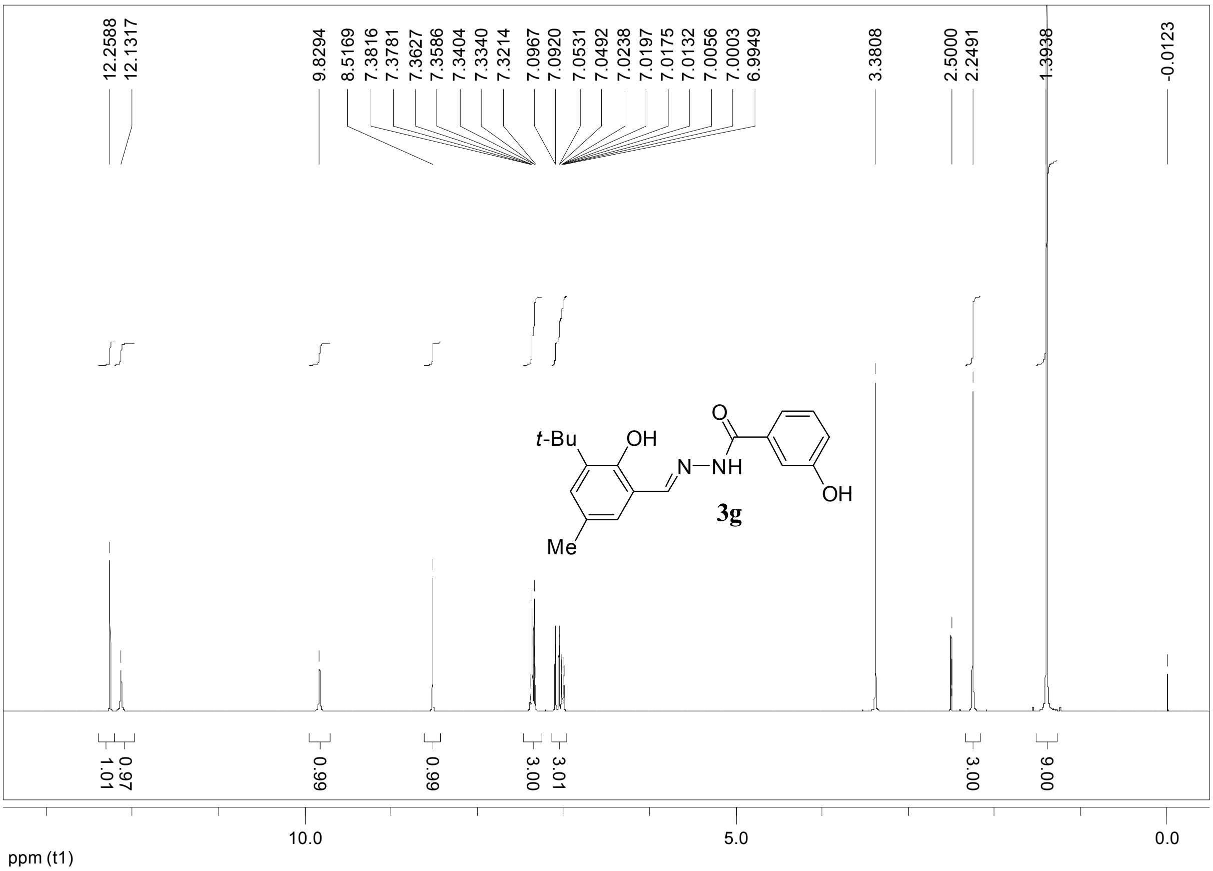

Figure S253. ^{1}H-NMR (400 MHz, DMSO-d_6) spectrum of **3g**.

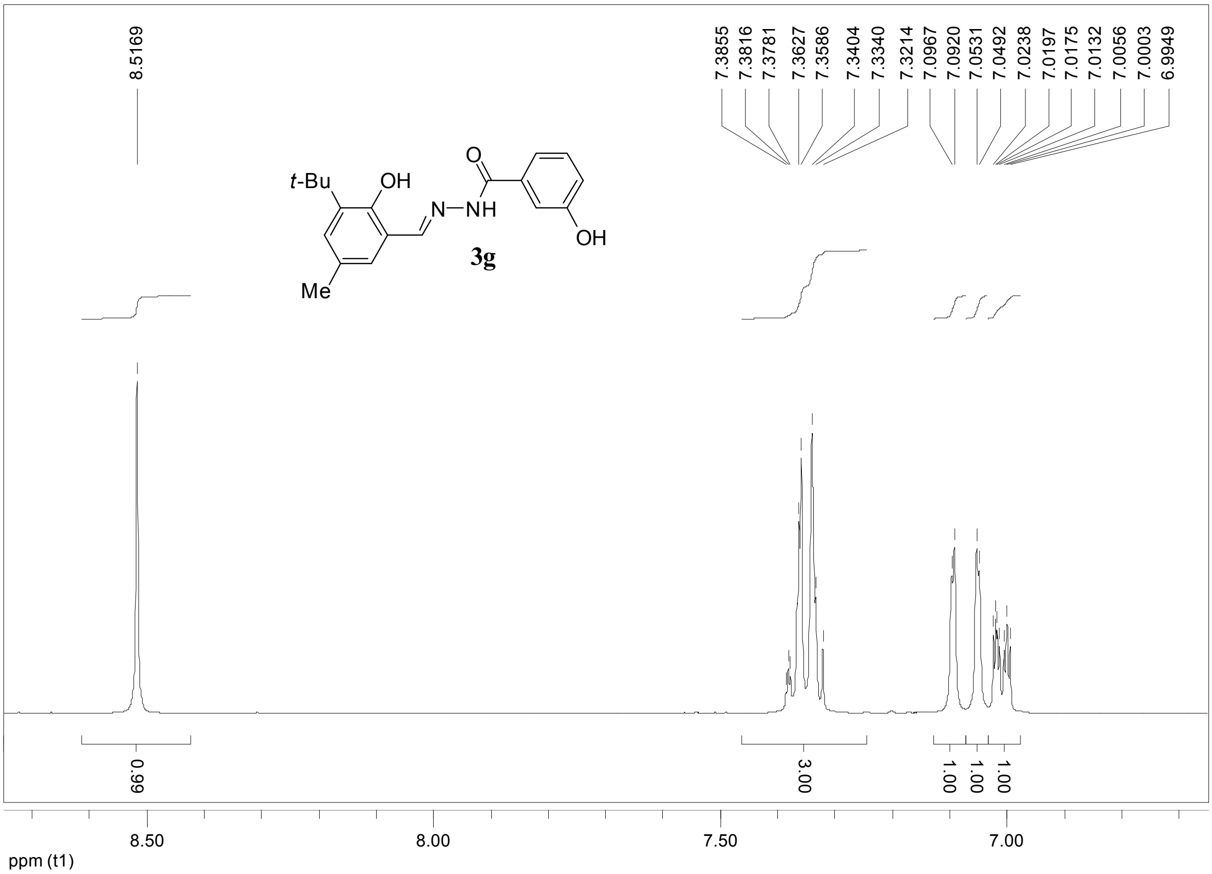

Figure S254. Expansion of ^{1}H-NMR (400 MHz, DMSO-d_6) spectrum of **3g**.

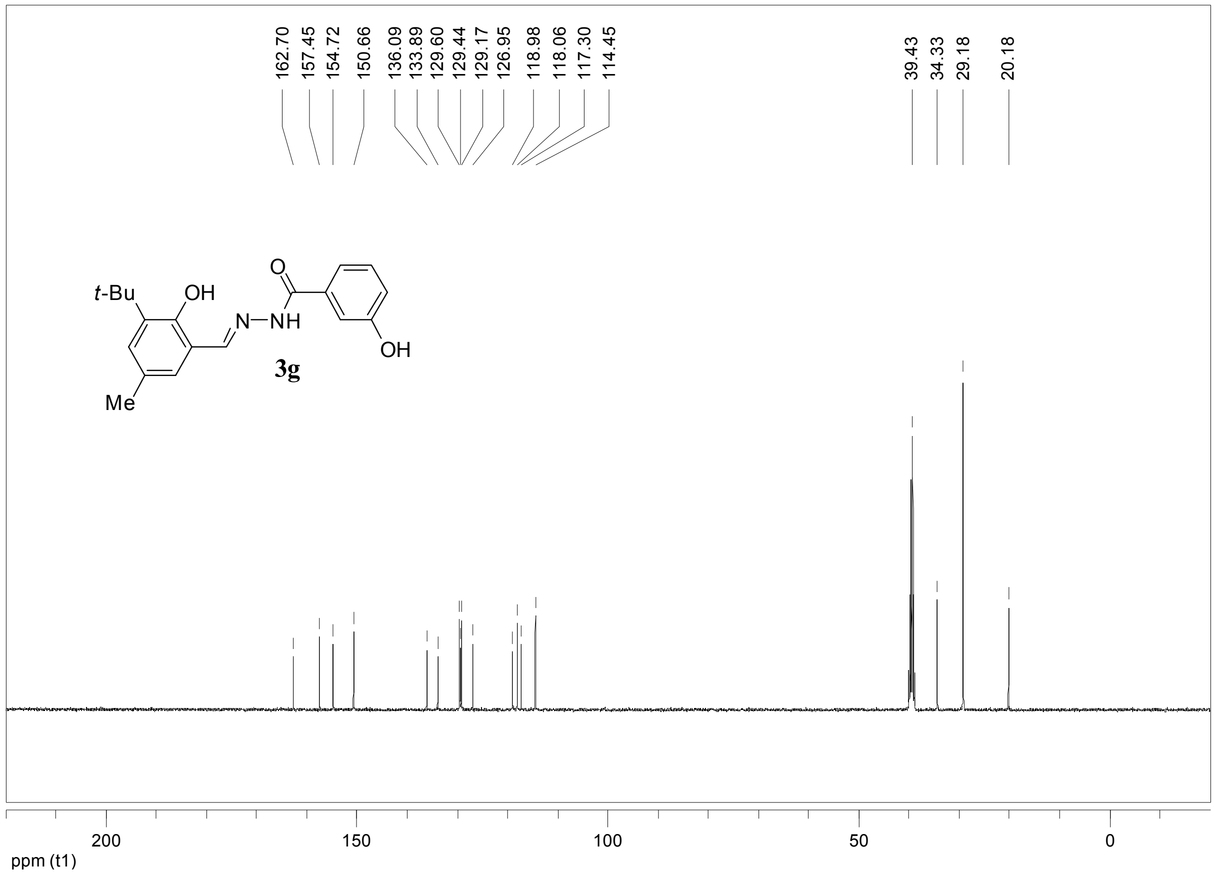

Figure S255. ^{13}C-NMR (100 MHz, DMSO-d_6) spectrum of **3g**.

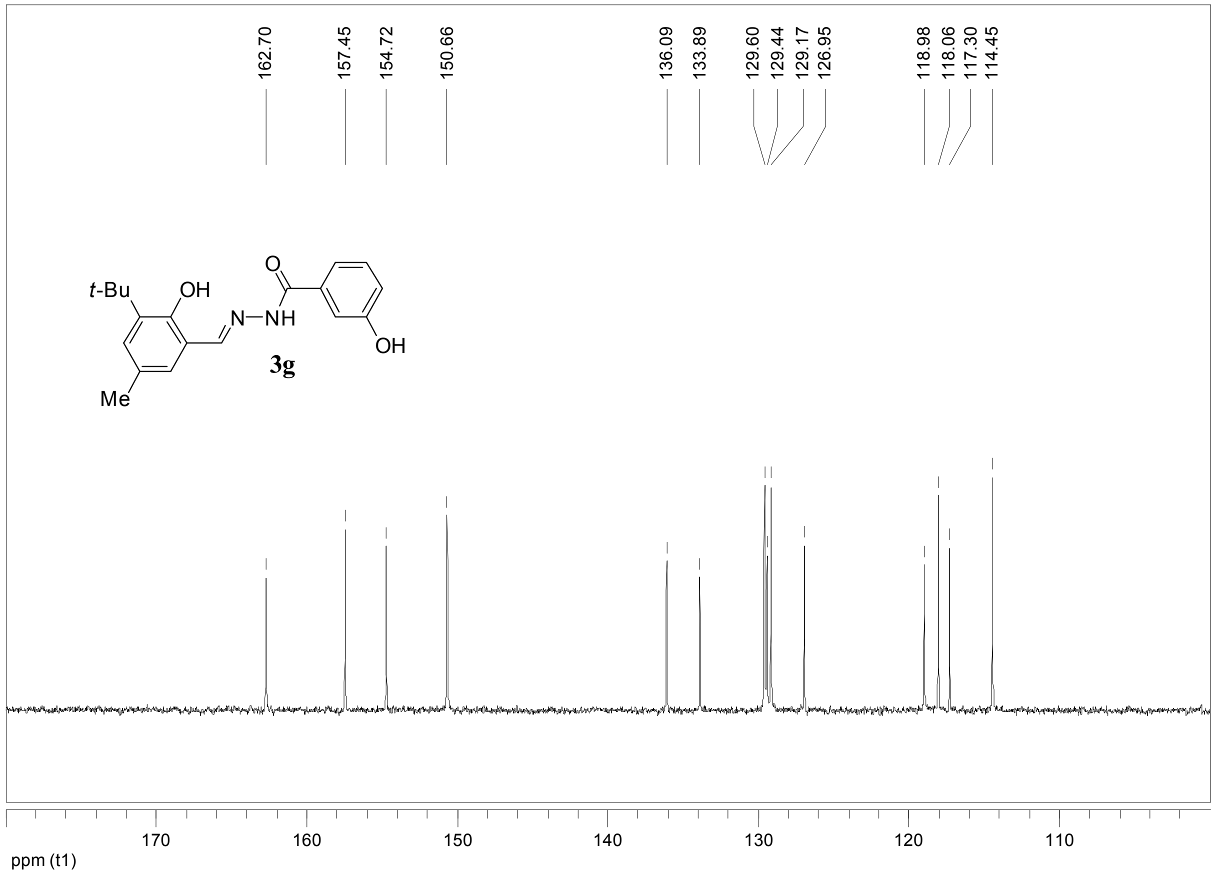

Figure S256. Expansion of ^{13}C-NMR (100 MHz, DMSO-d_6) spectrum of **3g**.

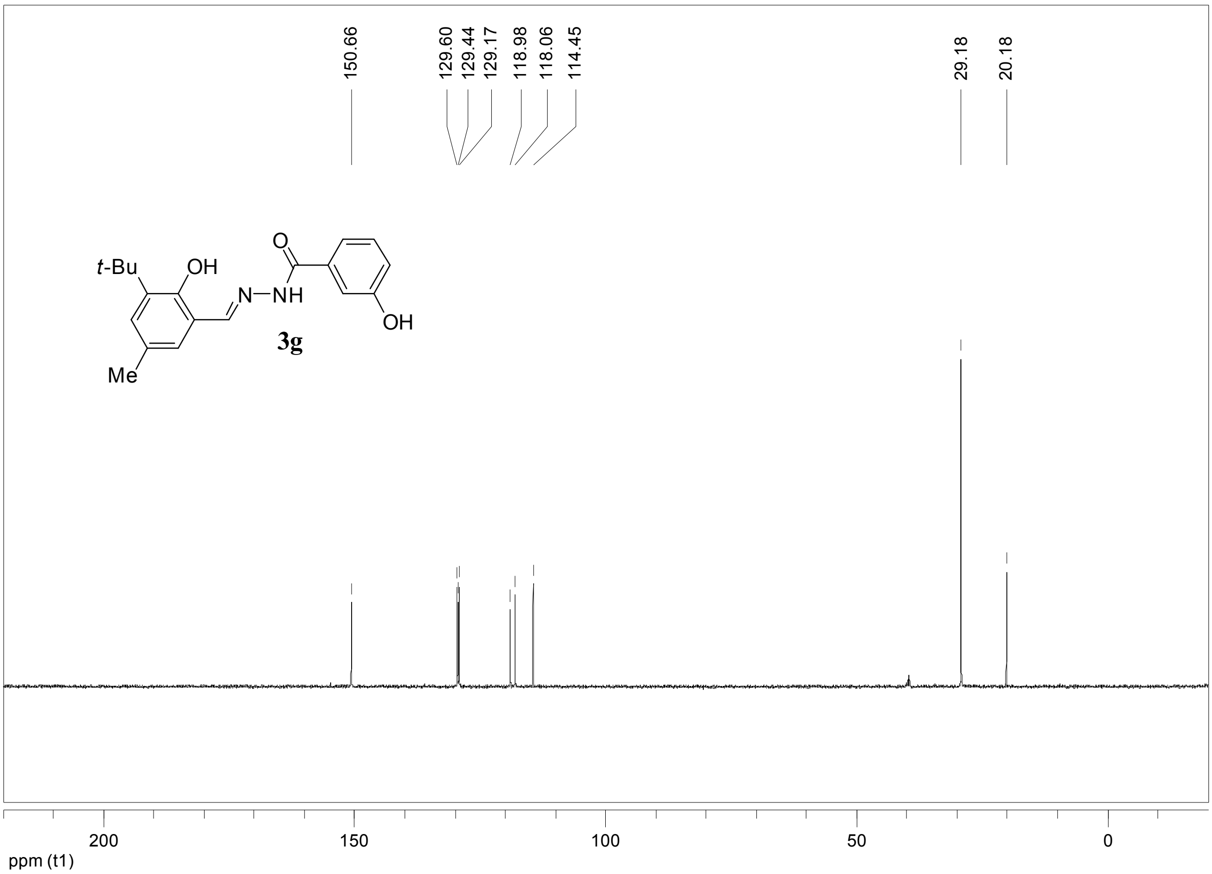

Figure S257. ^{13}C-NMR (100 MHz, DMSO-d_6) dept-135 experiment of **3g**.

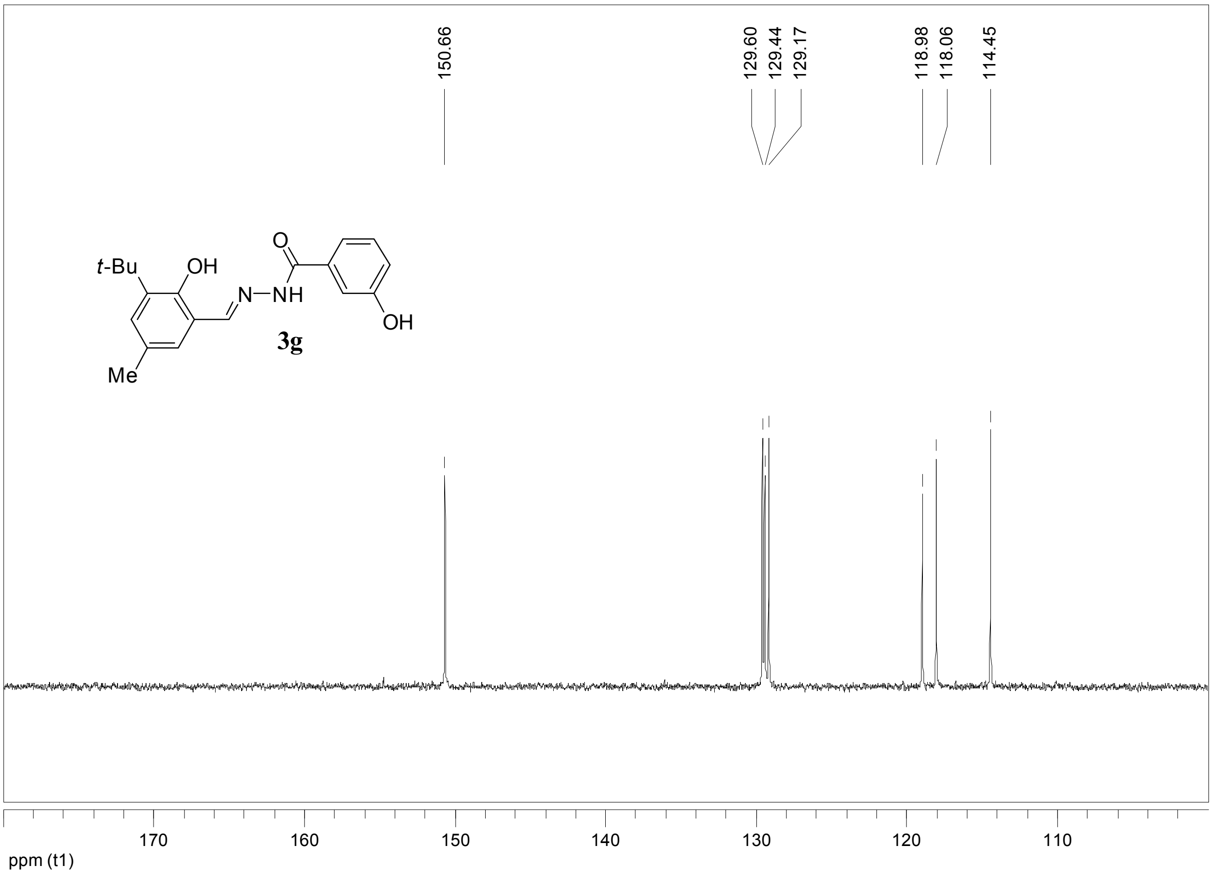

Figure S258. Expansion of ^{13}C-NMR (100 MHz, DMSO-d_6) dept-135 experiment of **3g**.

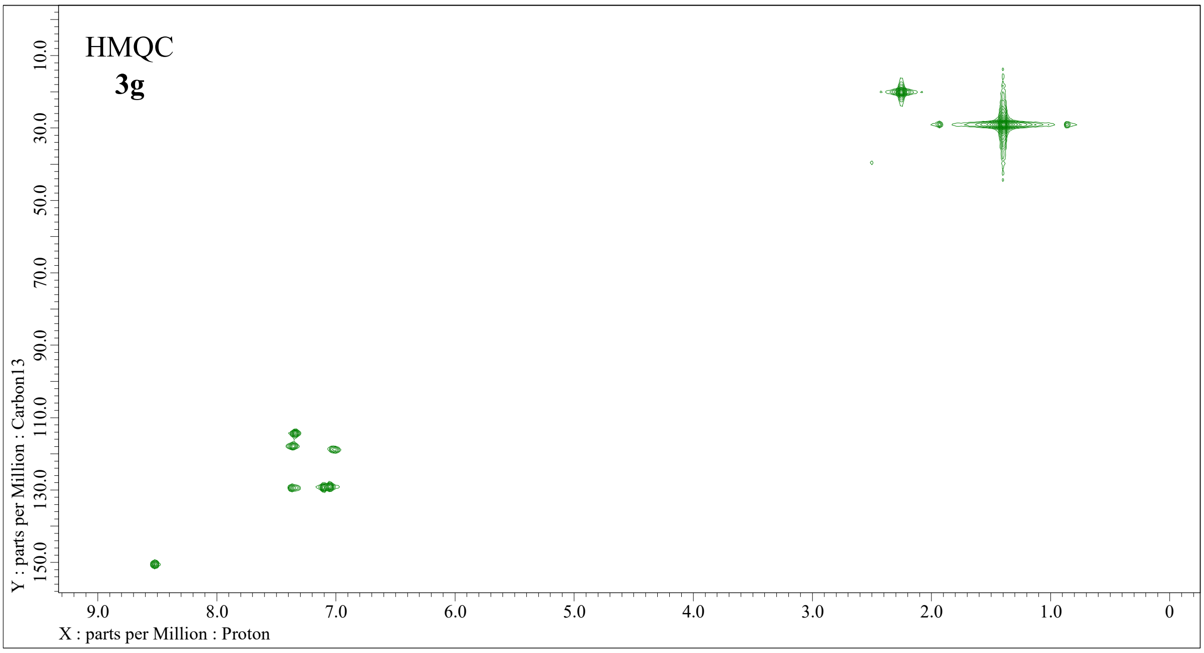

Figure S259. 2D-NMR (400 MHz, DMSO-*d*6) HMQC experiment of 3-hydroxy-*N*'-[(*E*)-(3-*tert*-butyl-2-hydroxy-5-methylphenyl)methylidene]benzohydrazide (**3g**).

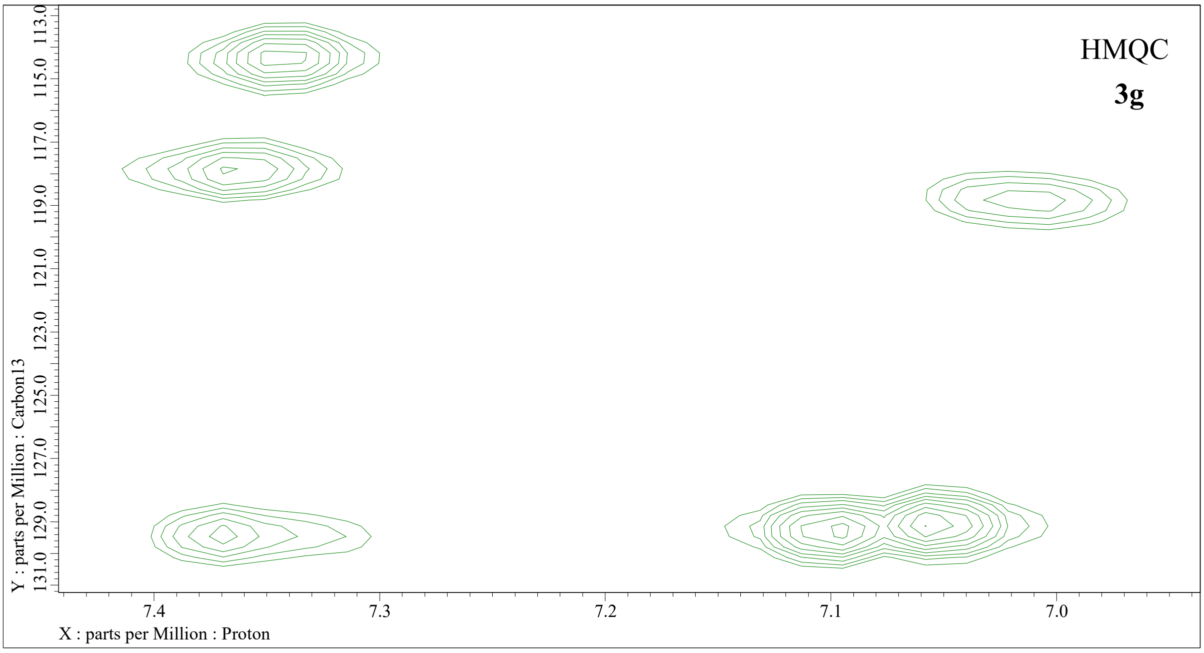

Figure S260. Expansion of 2D-NMR (400 MHz, DMSO-*d*6) HMQC experiment of 3-hydroxy-*N*'-[(*E*)-(3-*tert*-butyl-2-hydroxy-5-methylphenyl)methylidene]benzohydrazide (**3g**).

Molecules **2020**, *25*, 1255; doi:10.3390/molecules25051255 www.mdpi.com/journal/molecules

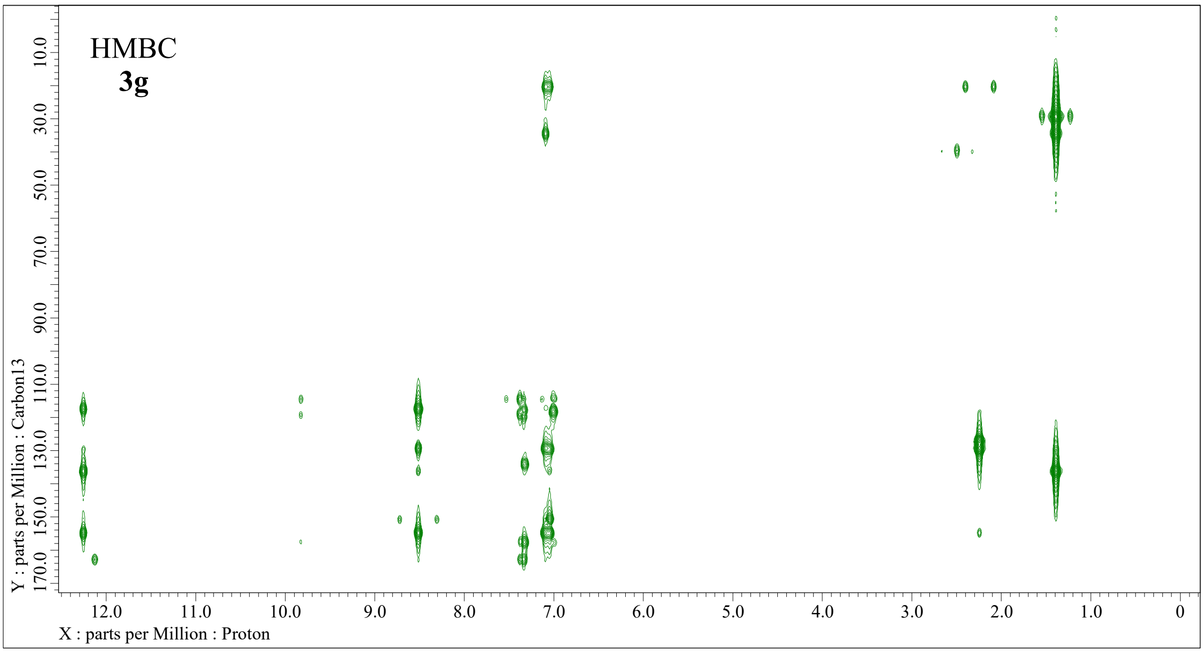

Figure S261. 2D-NMR (400 MHz, DMSO-d_6) HMBC experiment of 3-hydroxy-*N*'-[(*E*)-(3-*tert*-butyl-2-hydroxy-5-methylphenyl)methylidene]benzohydrazide (**3g**).

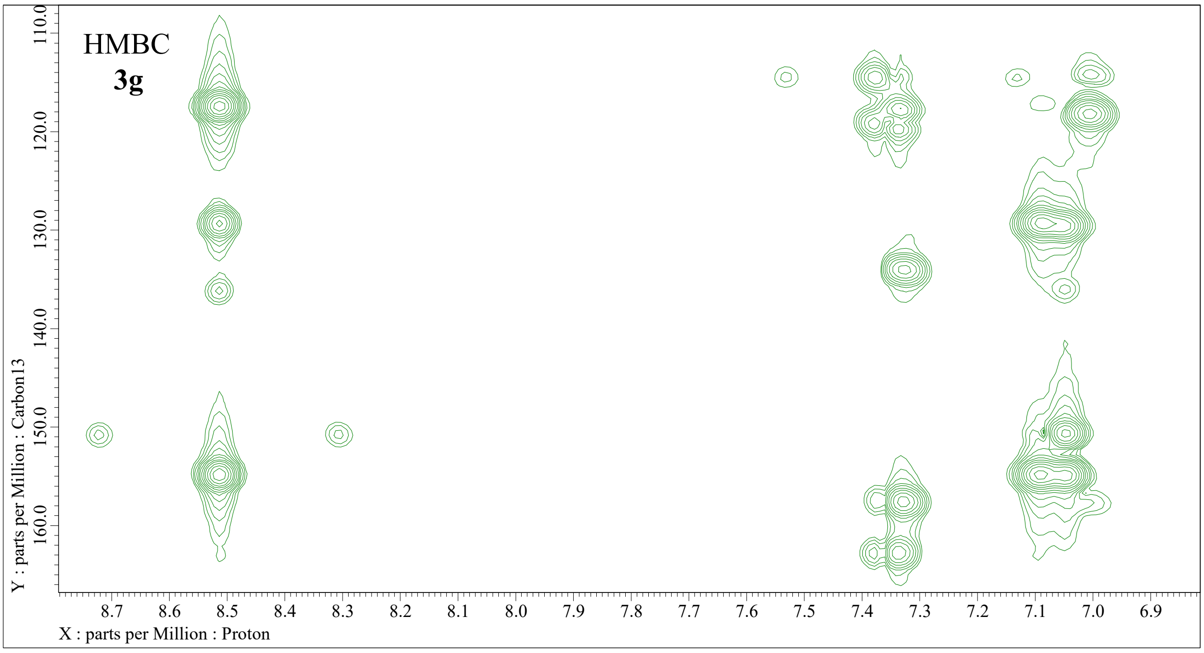

Figure S262. Expansion of 2D-NMR (400 MHz, DMSO-d_6) HMBC experiment of 3-hydroxy-*N*'-[(*E*)-(3-*tert*-butyl-2-hydroxy-5-methylphenyl)methylidene]benzohydrazide (**3g**).

Molecules **2020**, *25*, 1255; doi:10.3390/molecules25051255 www.mdpi.com/journal/molecules

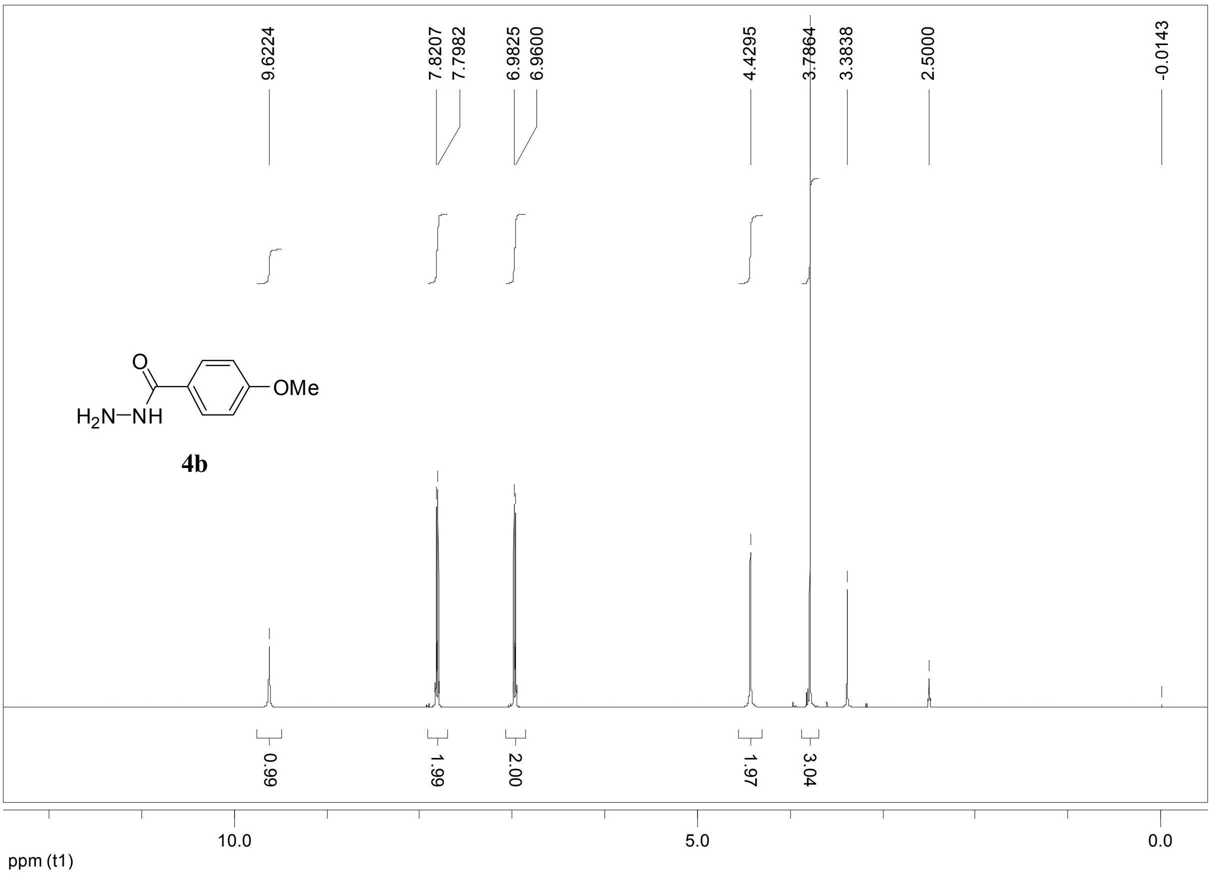

Figure S263. ¹H-NMR (400 MHz, DMSO-d_6) spectrum of 4-methoxybenzohydrazide (**4b**).

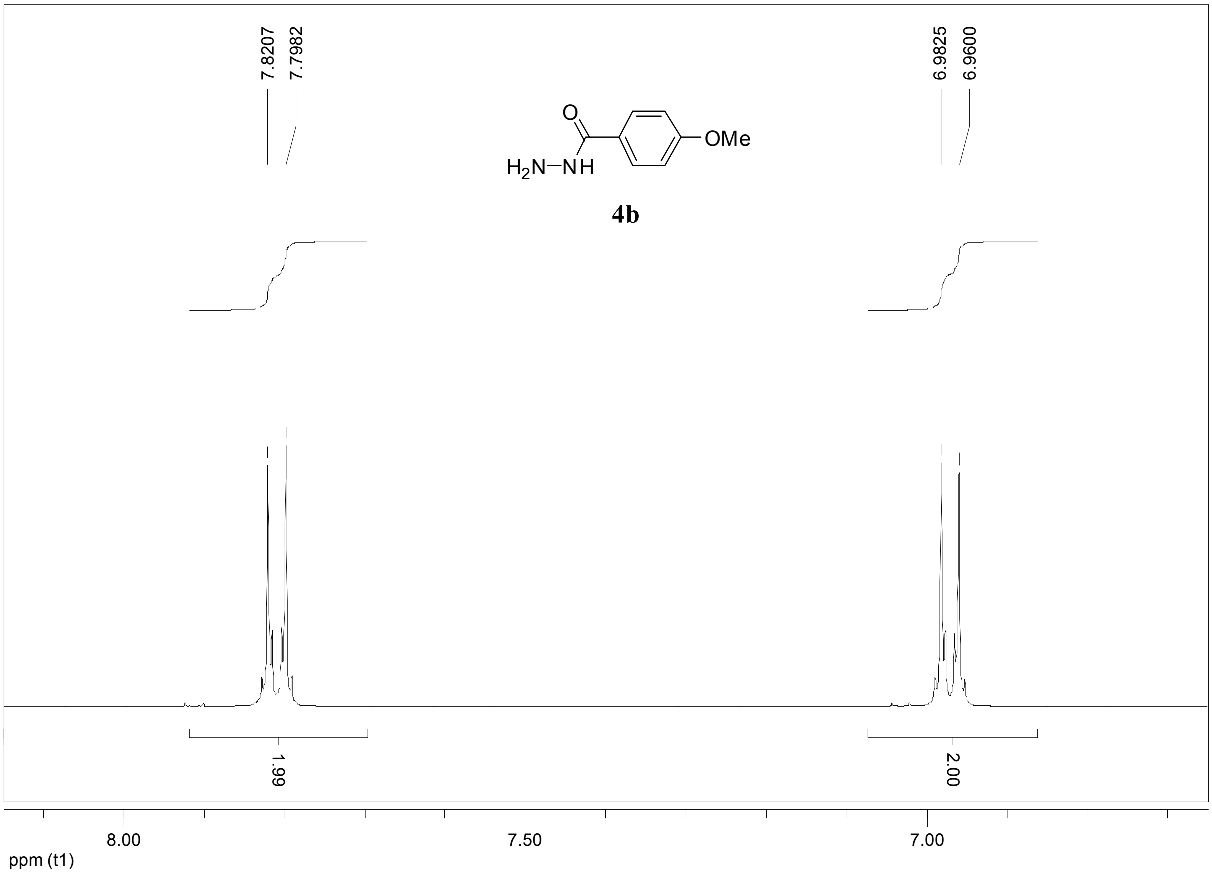

Figure S264. Expansion of ¹H-NMR (400 MHz, DMSO-d_6) spectrum of 4-methoxybenzohydrazide (**4b**).

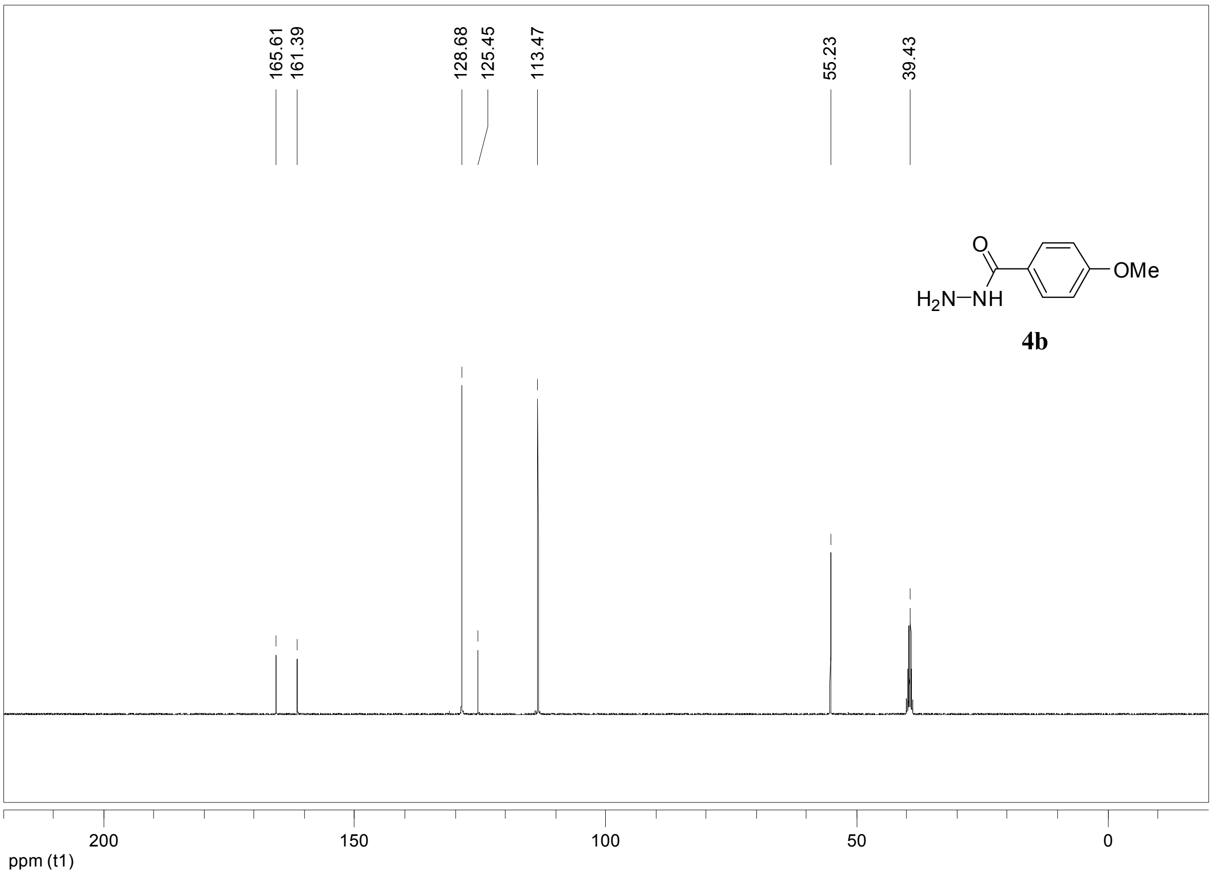

Figure S265. ^{13}C-NMR (100 MHz, DMSO-d_6) spectrum of 4-methoxybenzohydrazide (**4b**).

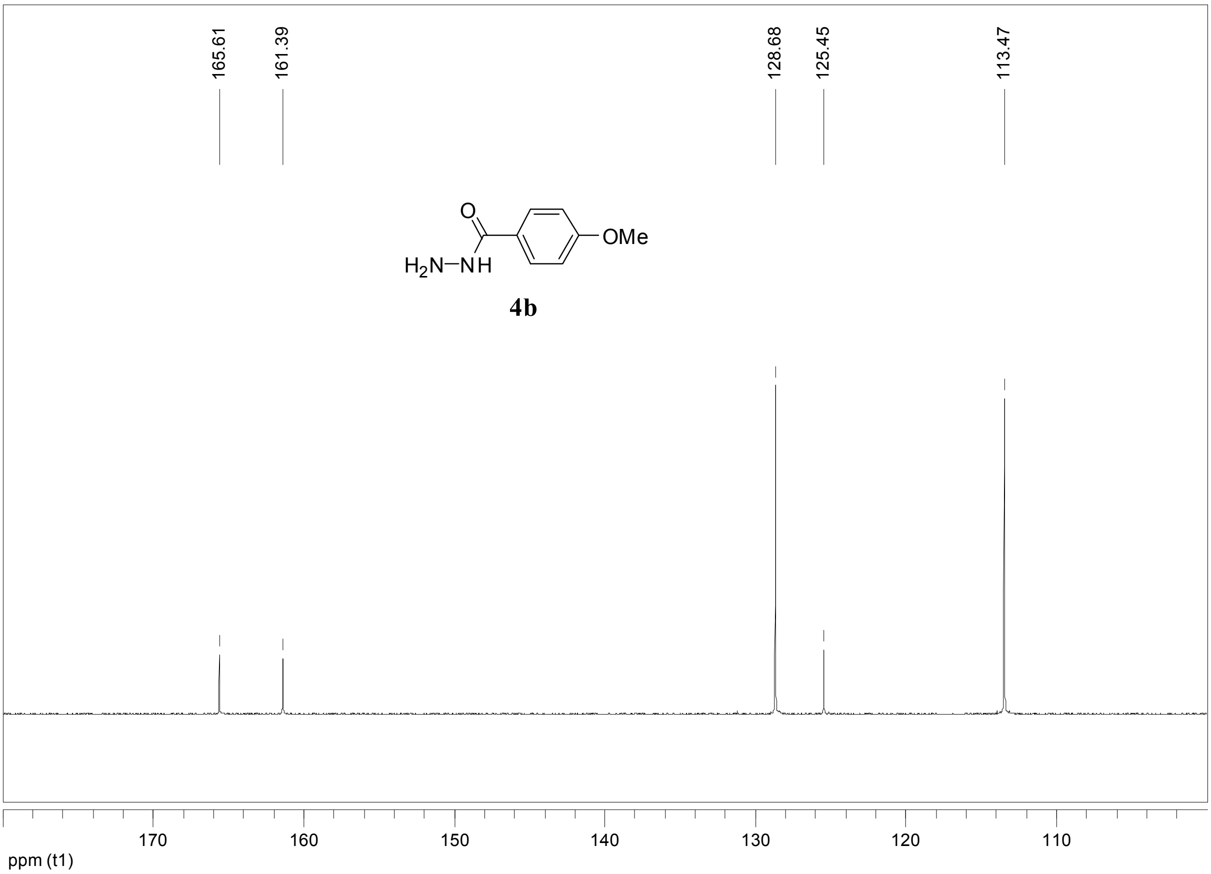

Figure S266. Expansion of ^{13}C-NMR (100 MHz, DMSO-d_6) spectrum of methoxybenzohydrazide **4b**.

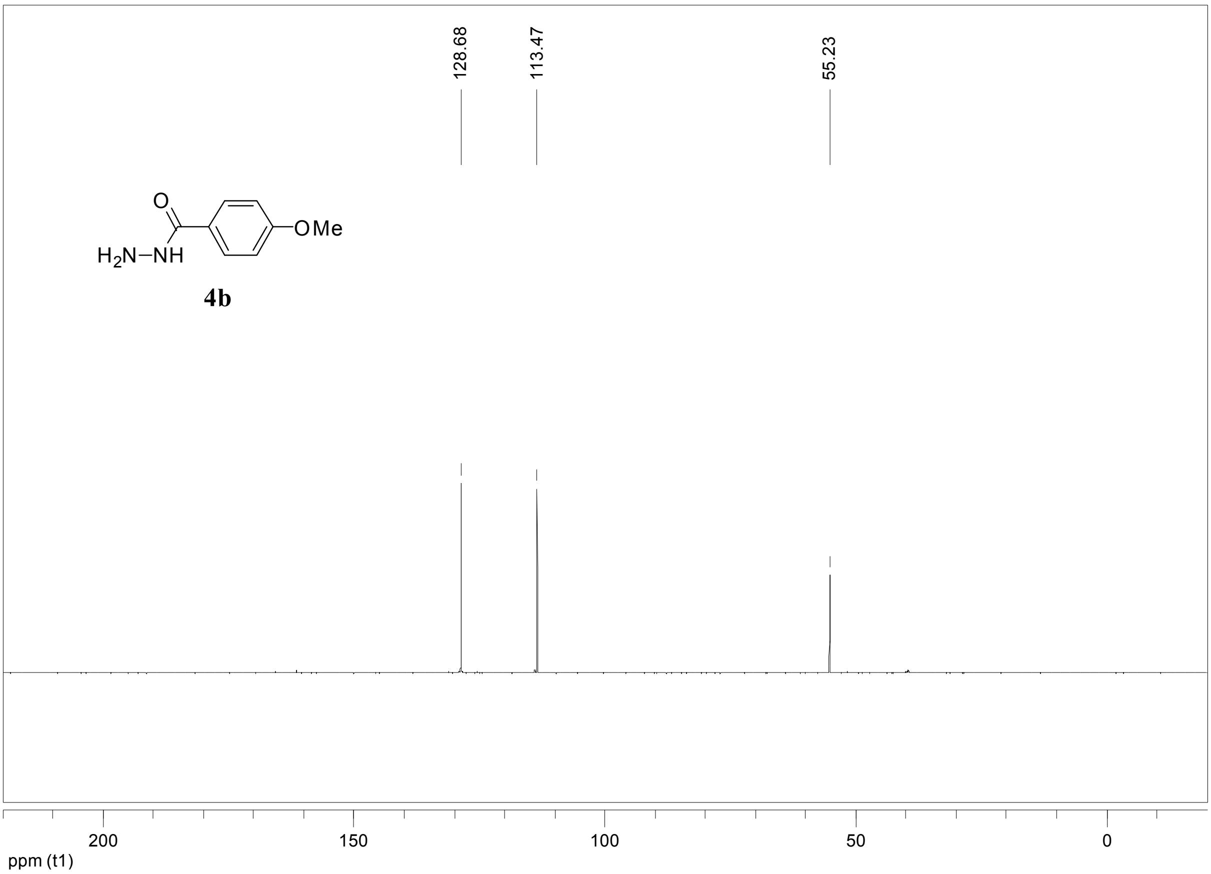

Figure S267. ^{13}C-NMR (100 MHz, DMSO-d_6) dept-135 experiment of 4-methoxybenzohydrazide (**4b**).

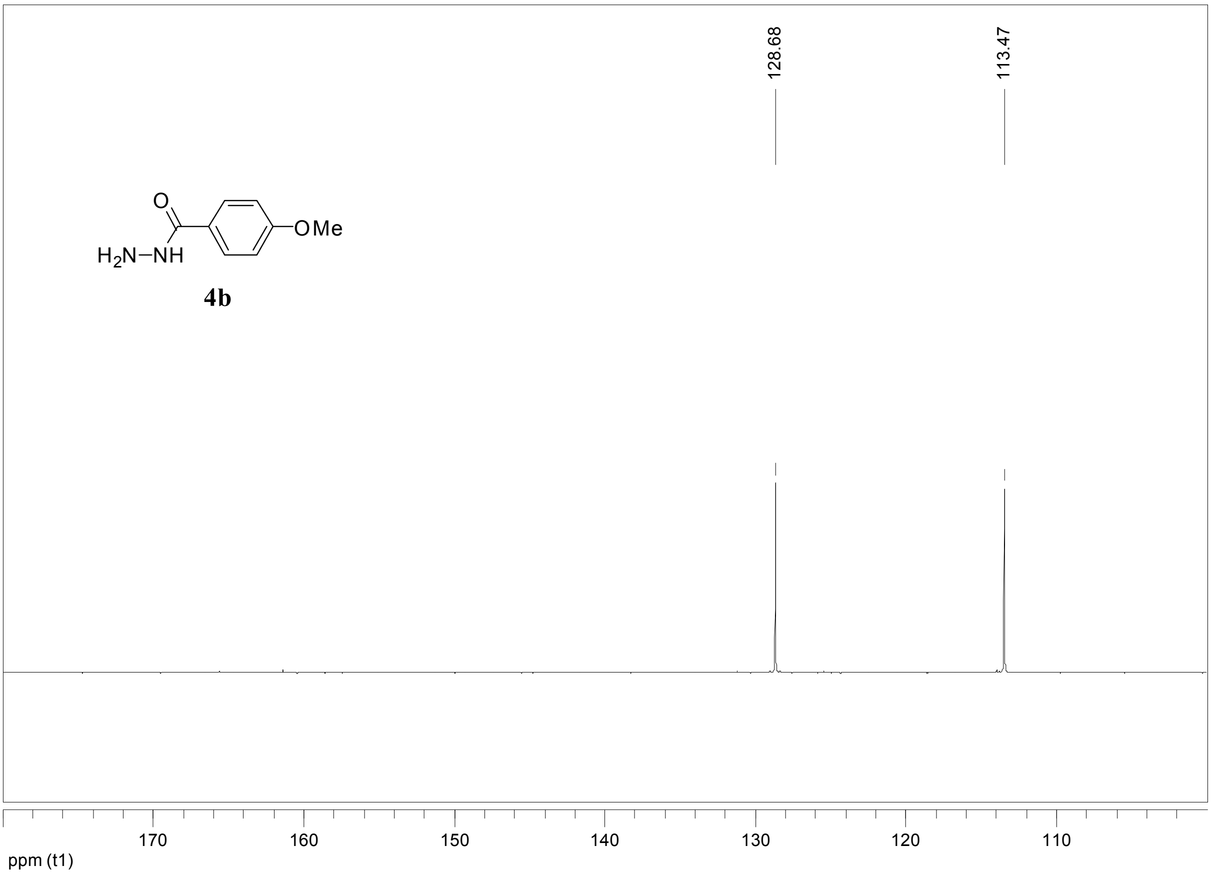

Figure S268. Expansion of ^{13}C-NMR (100 MHz, DMSO-d_6) dept-135 experiment of benzohydrazide **4b**.

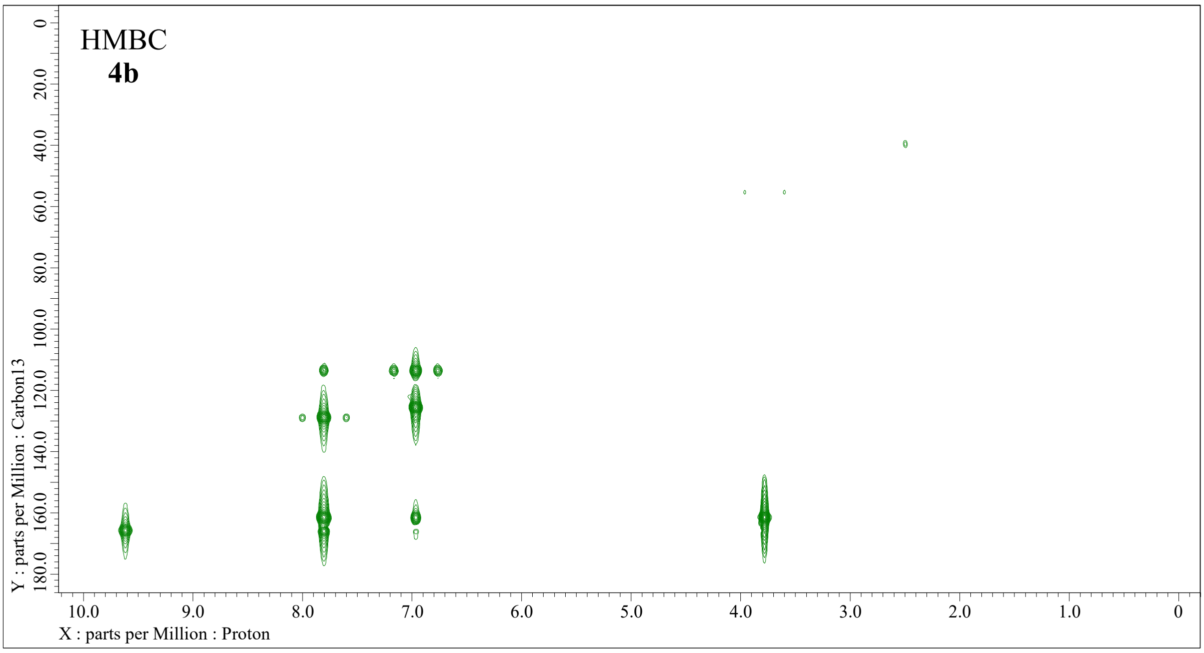

Figure S269. 2D-NMR (400 MHz, DMSO-d_6) HMBC experiment of 4-methoxybenzohydrazide (**4b**).

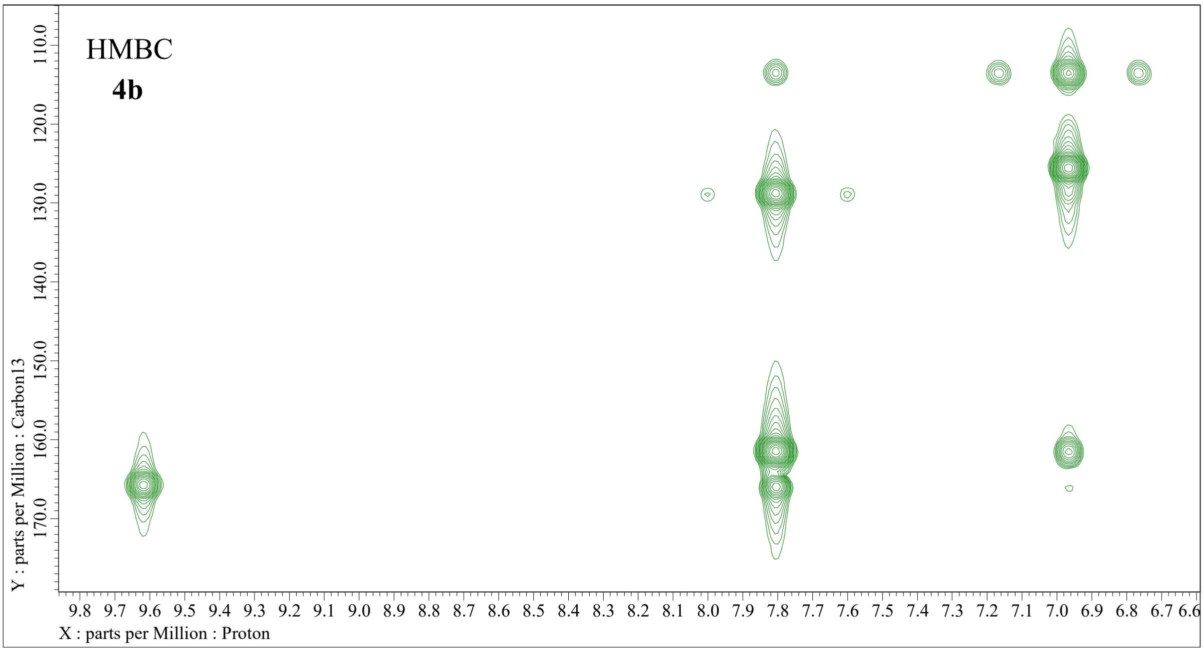

Figure S270. Expansion of 2D-NMR (400 MHz, DMSO-d_6) HMBC experiment of 4-methoxybenzohydrazide (**4b**).

Molecules **2020**, *25*, 1255; doi:10.3390/molecules25051255 www.mdpi.com/journal/molecules

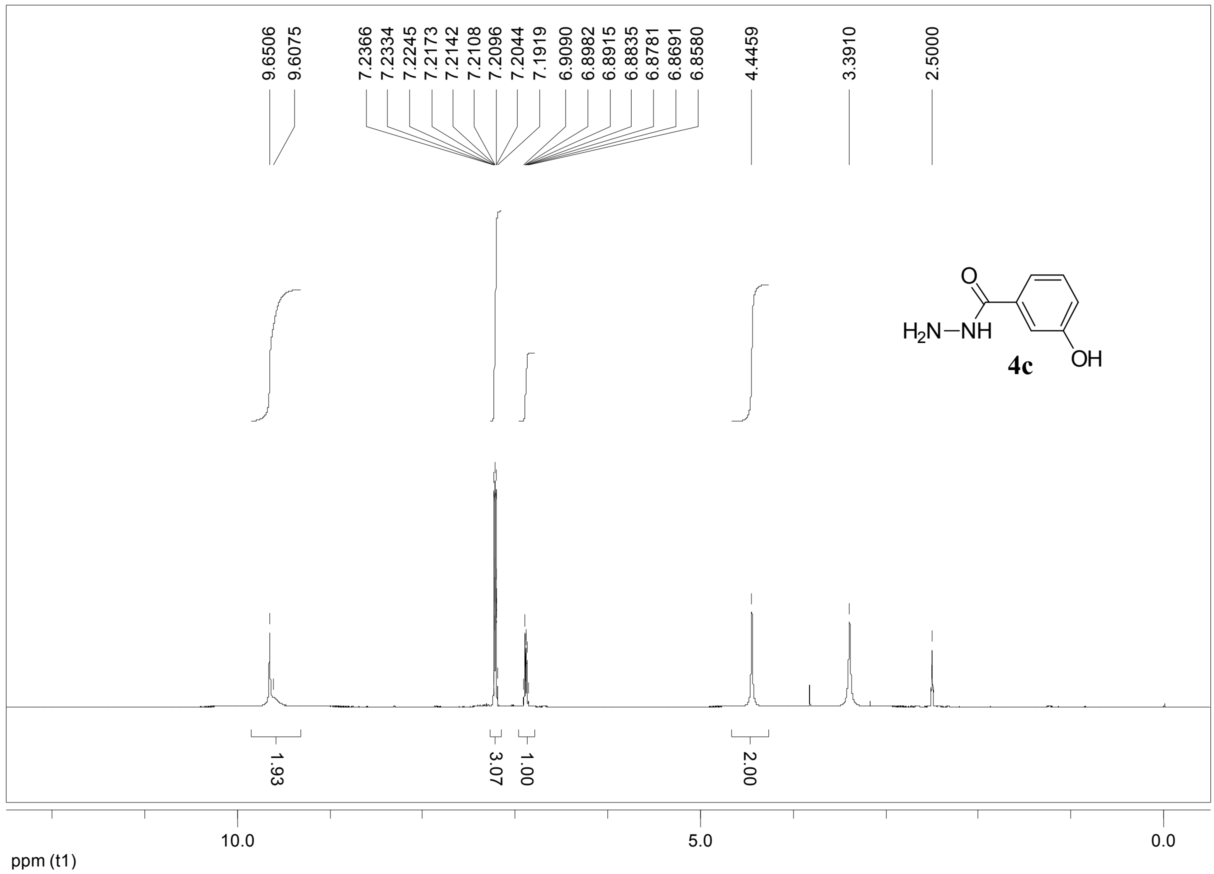

Figure S271. ^{1}H-NMR (400 MHz, DMSO-d_6) spectrum of 3-hydroxybenzohydrazide (**4c**).

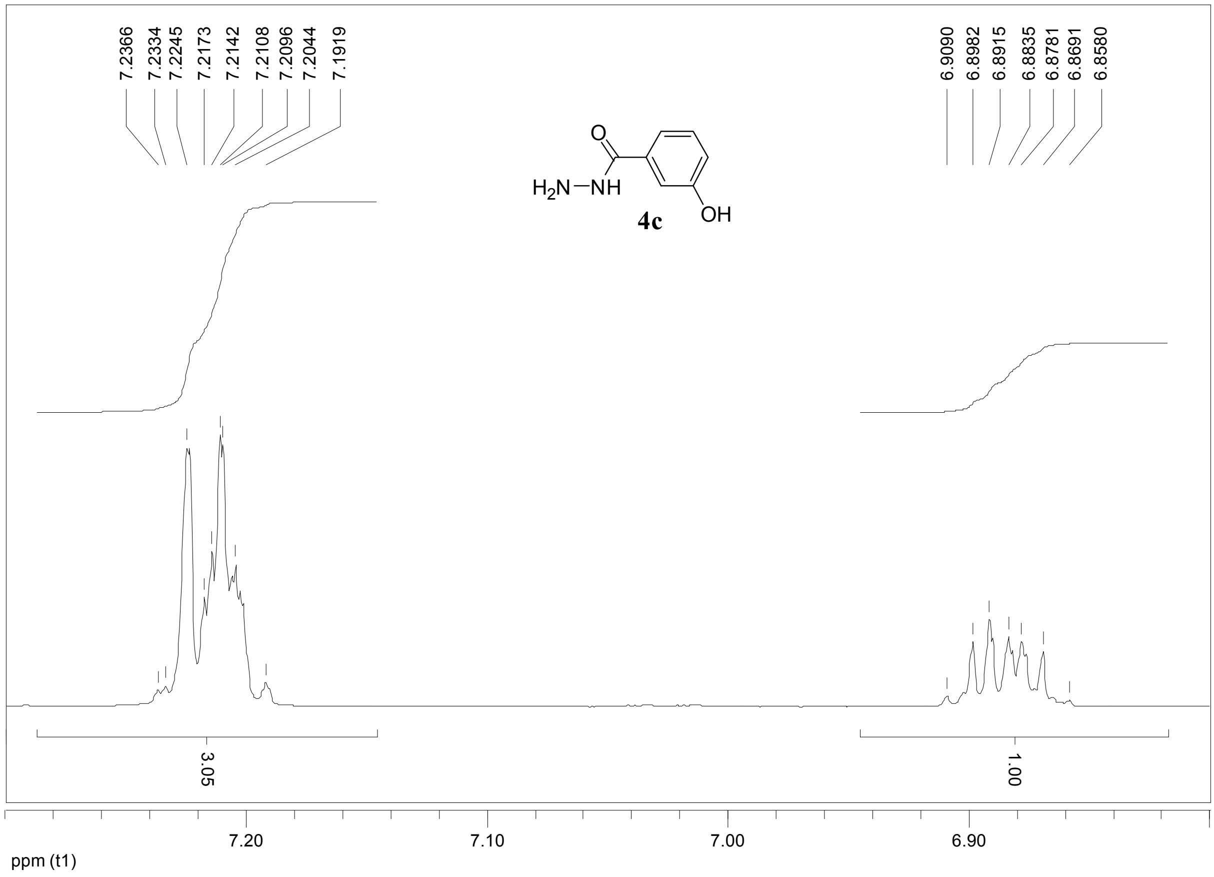

Figure S272. Expansion of ^{1}H-NMR (400 MHz, DMSO-d_6) spectrum of 3-hydroxybenzohydrazide (**4c**).

Molecules **2020**, *25*, 1255; doi:10.3390/molecules25051255 www.mdpi.com/journal/molecules

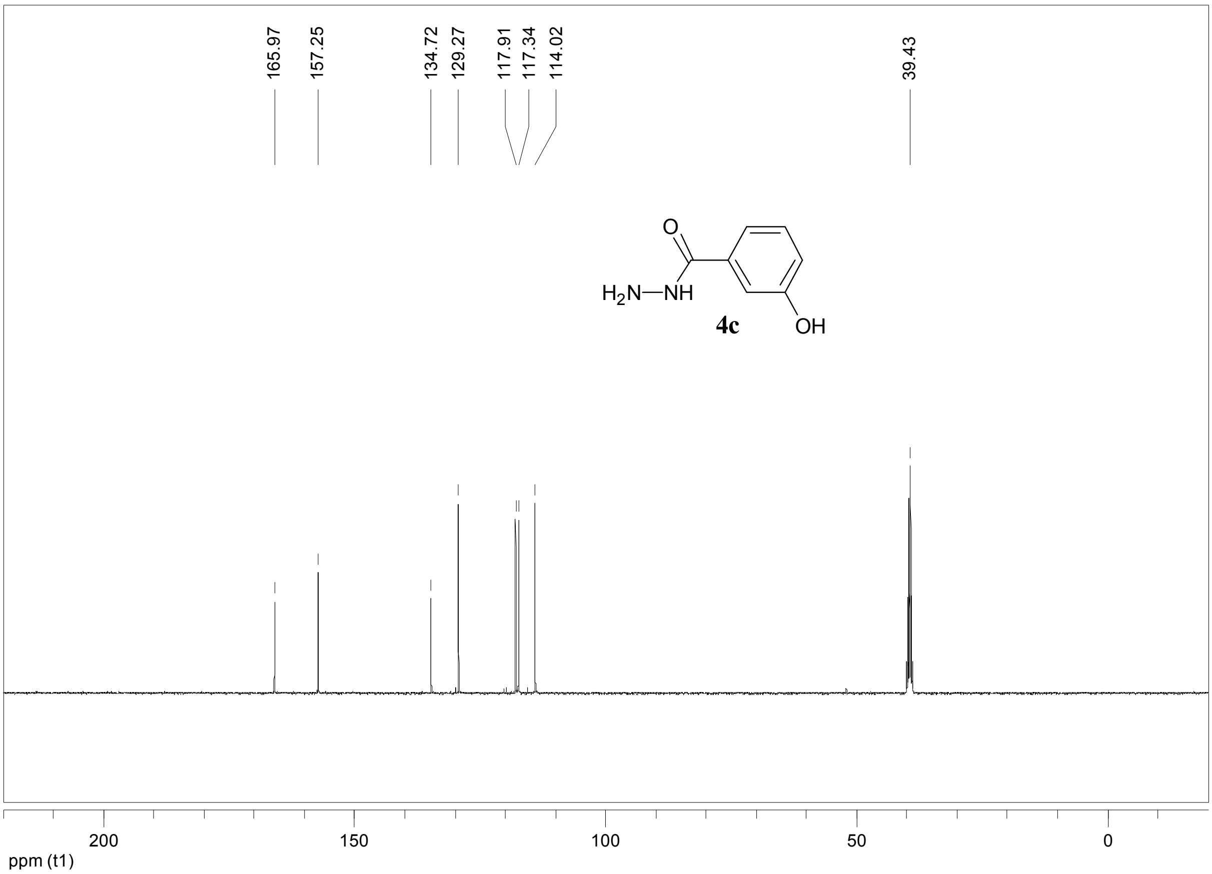

Figure S273. ^{13}C-NMR (100 MHz, DMSO-d_6) spectrum of 3-hydroxybenzohydrazide (**4c**).

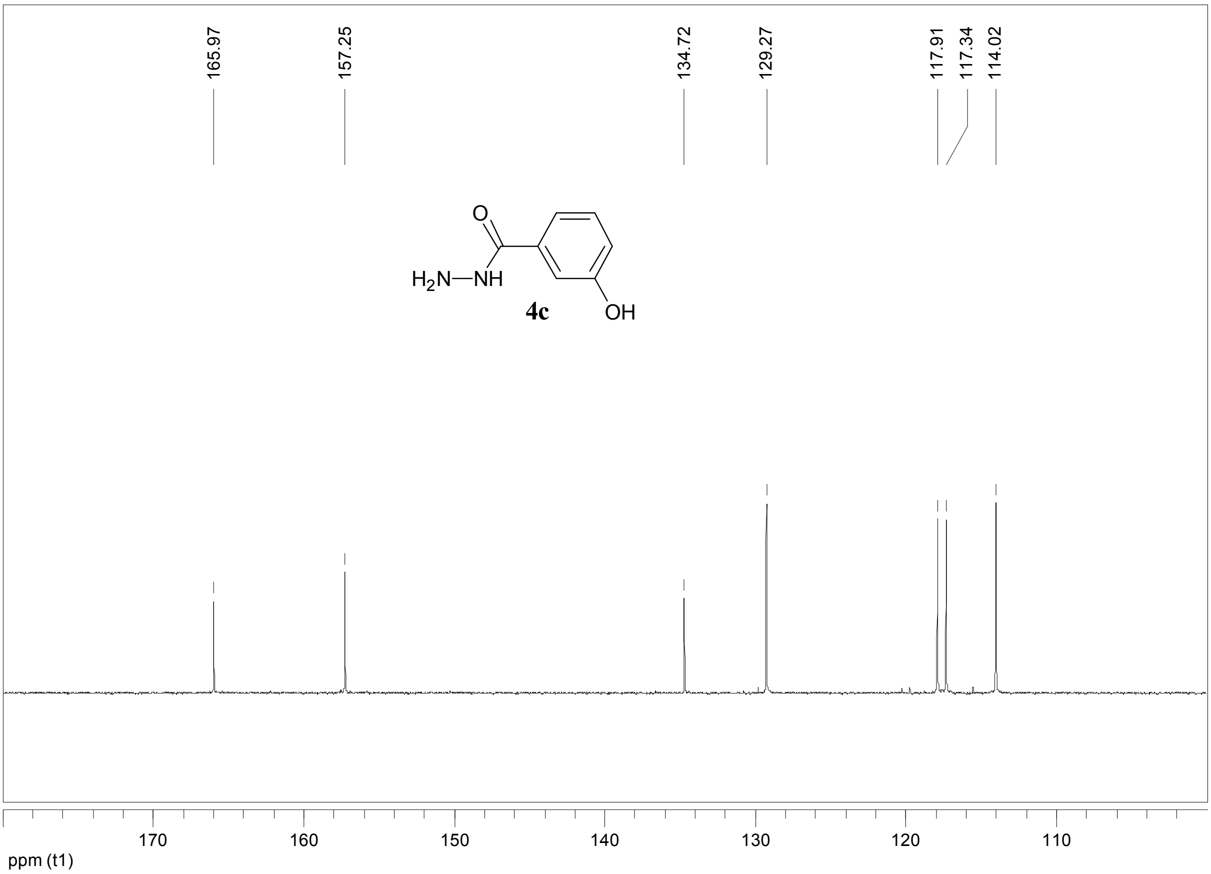

Figure S274. Expansion of ^{13}C-NMR (100 MHz, DMSO-d_6) spectrum of 3-hydroxybenzohydrazide (**4c**).

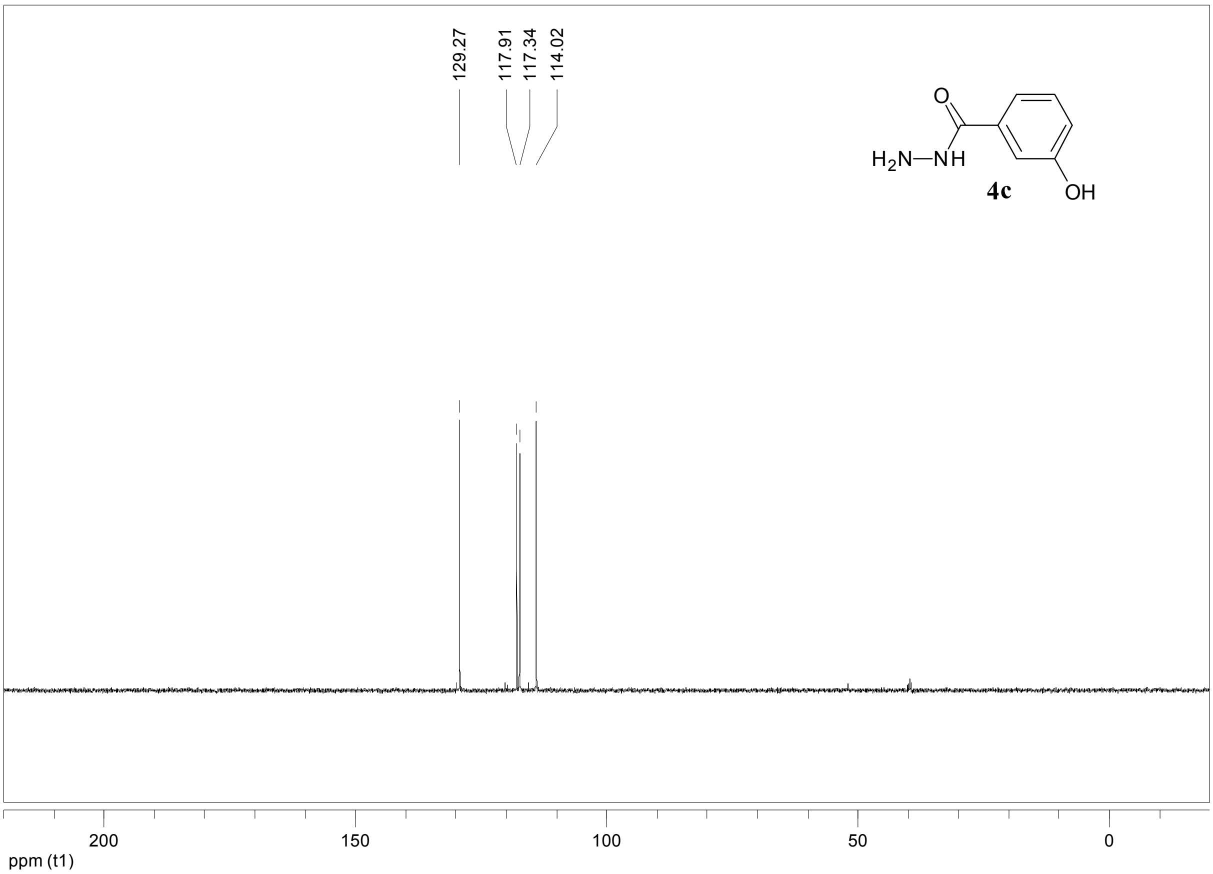

Figure S275. ^{13}C-NMR (100 MHz, DMSO-d_6) dept-135 experiment of 3-hydroxybenzohydrazide (**4c**).

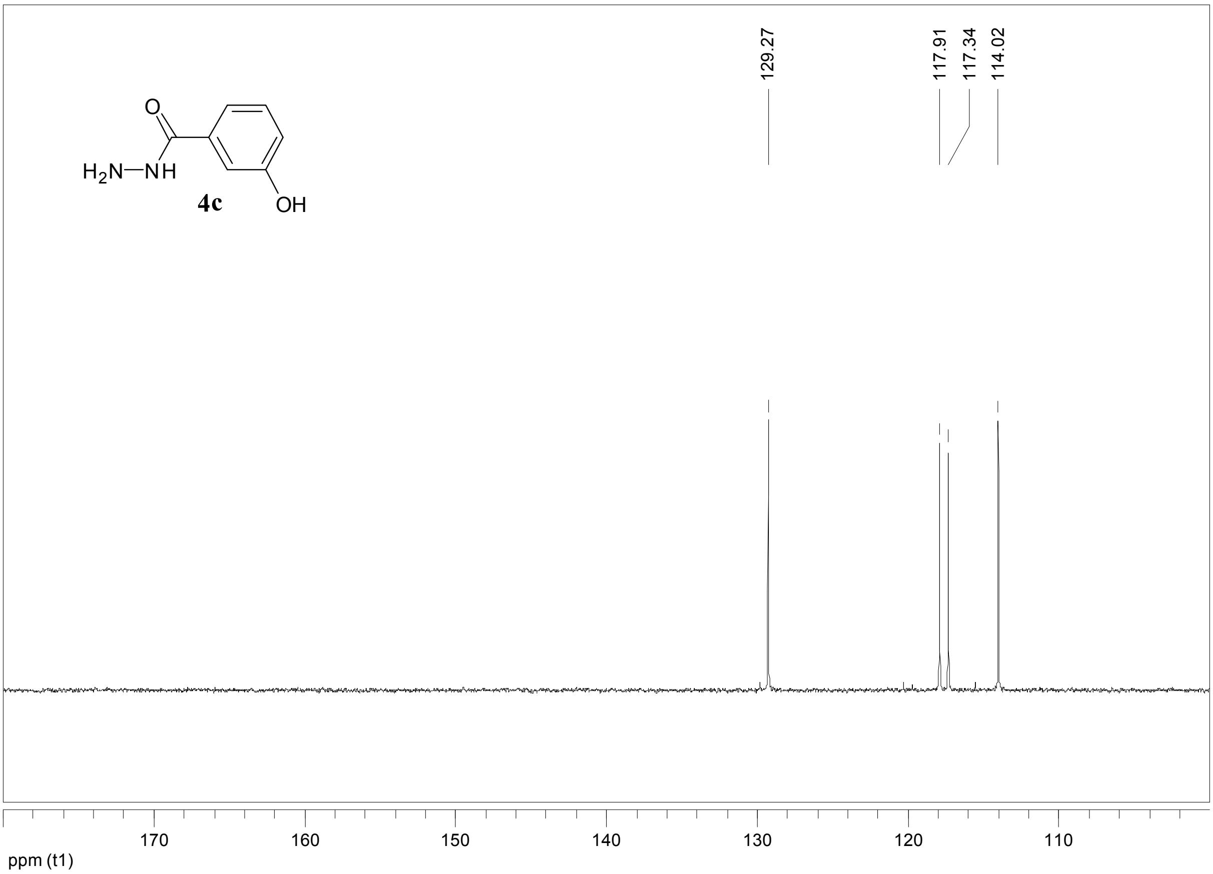

Figure S276. Expansion of ^{13}C-NMR (100 MHz, DMSO-d_6) dept-135 experiment of benzohydrazide **4c**.

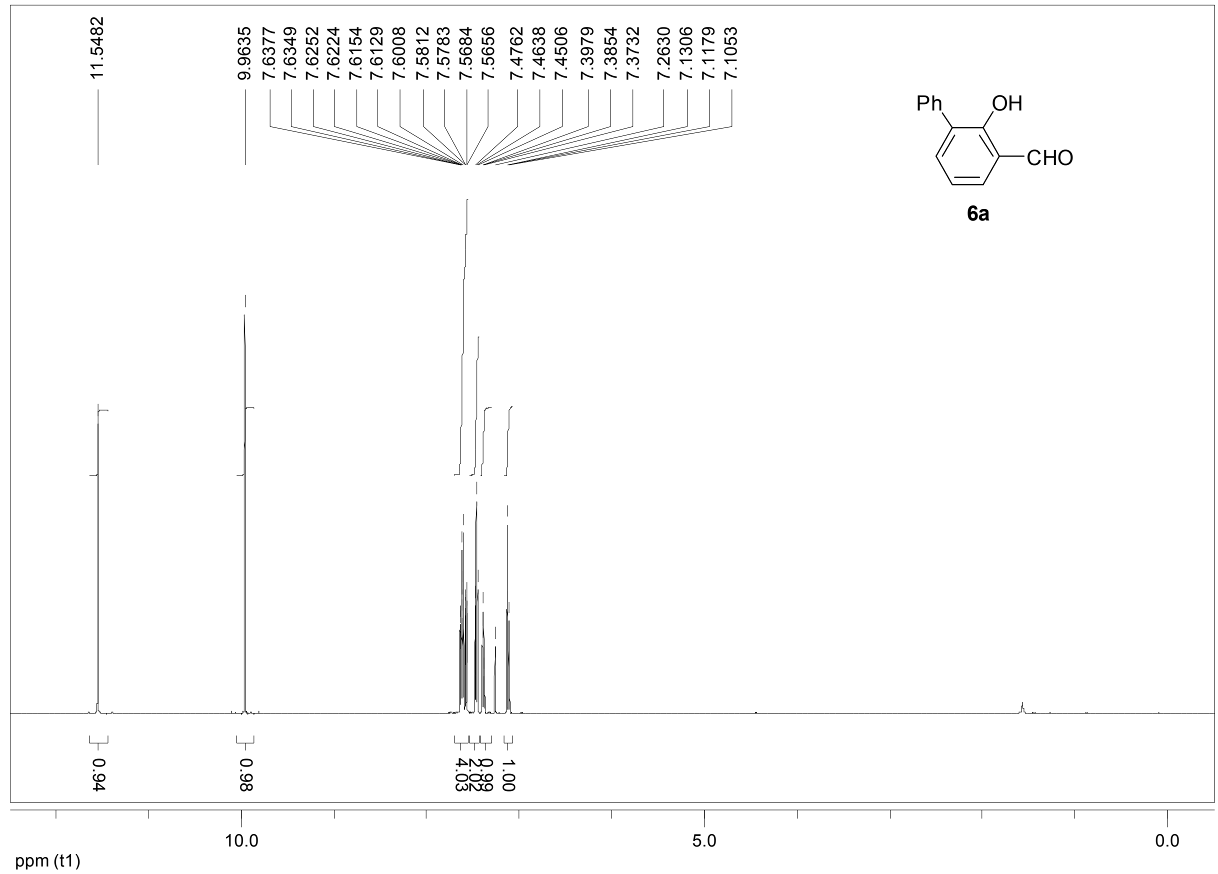

Figure S277. ^{1}H-NMR (600 MHz, $CDCl_3$) spectrum of 3-phenyl-salicylic aldehyde (**6a**).

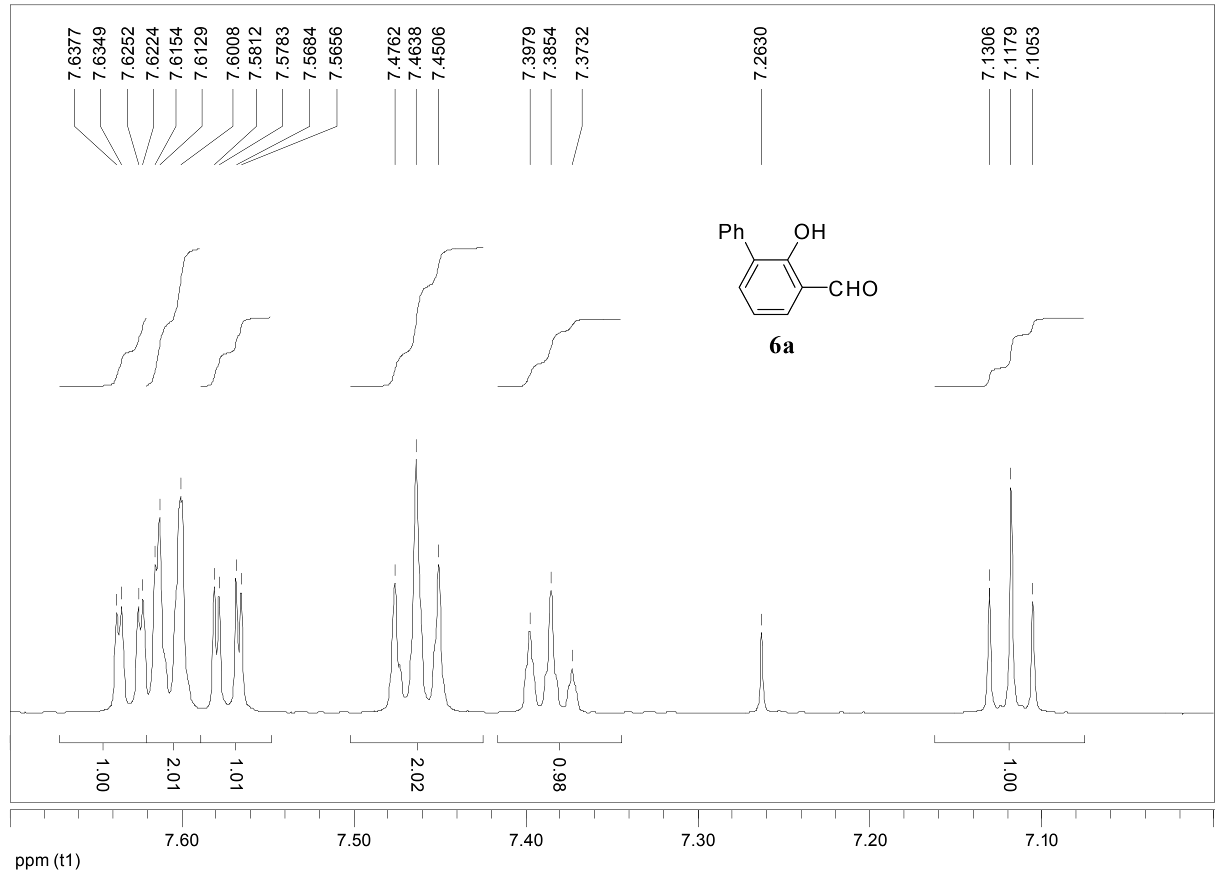

Figure S278. Expansion of ^{1}H-NMR (600 MHz, $CDCl_3$) spectrum of 3-phenyl-salicylic aldehyde (**6a**).

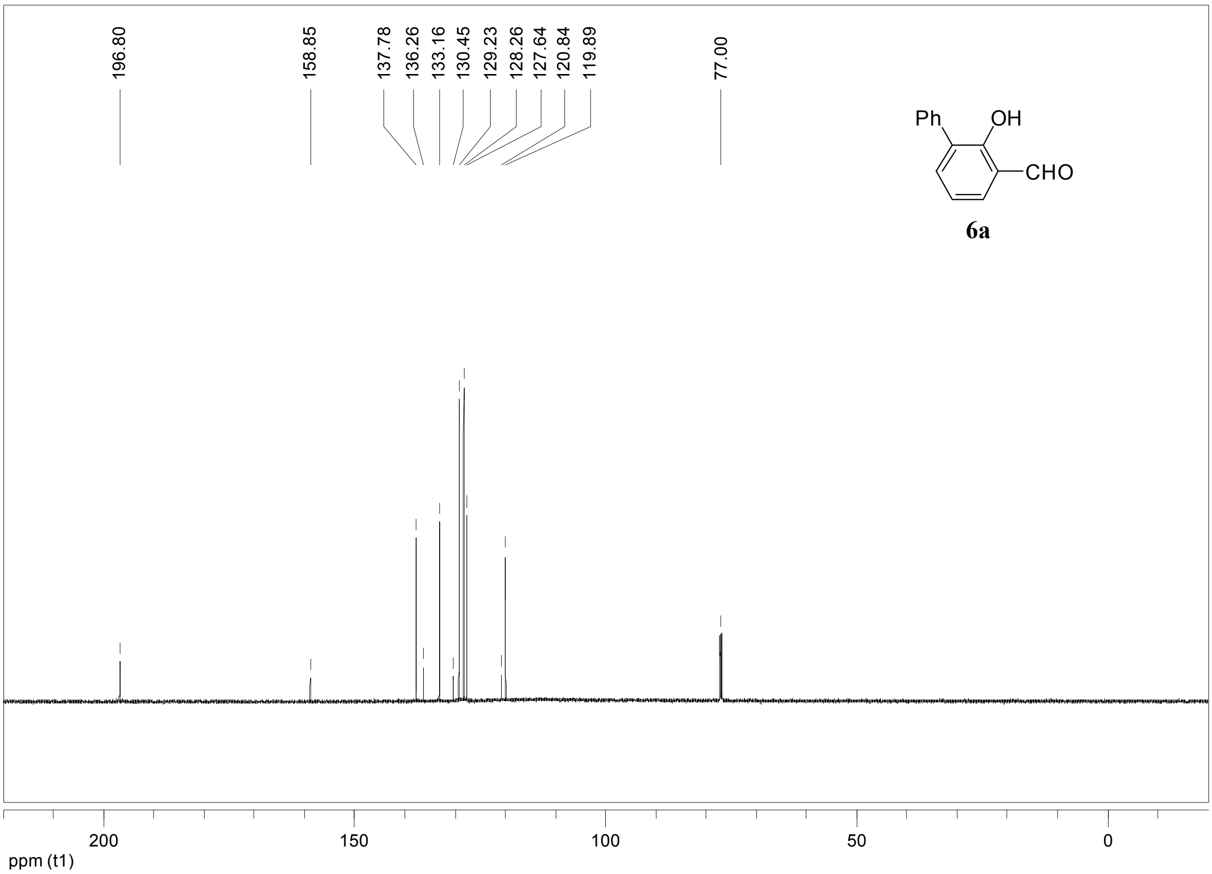

Figure S279. ^{13}C-NMR (150 MHz, $CDCl_3$) spectrum of 3-phenyl-salicylic aldehyde (**6a**).

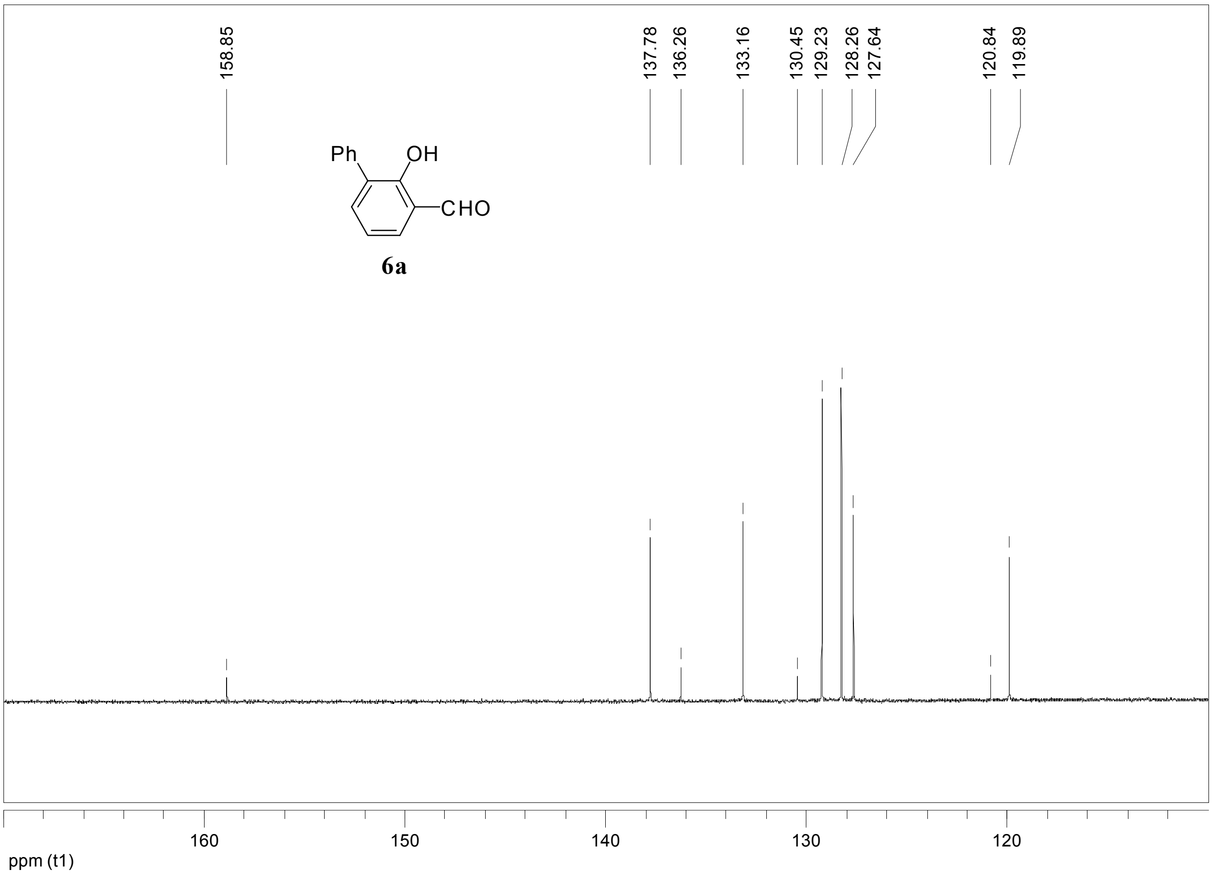

Figure S280. Expansion of ^{13}C-NMR (150 MHz, $CDCl_3$) spectrum of 3-phenyl-salicylic aldehyde (**6a**).

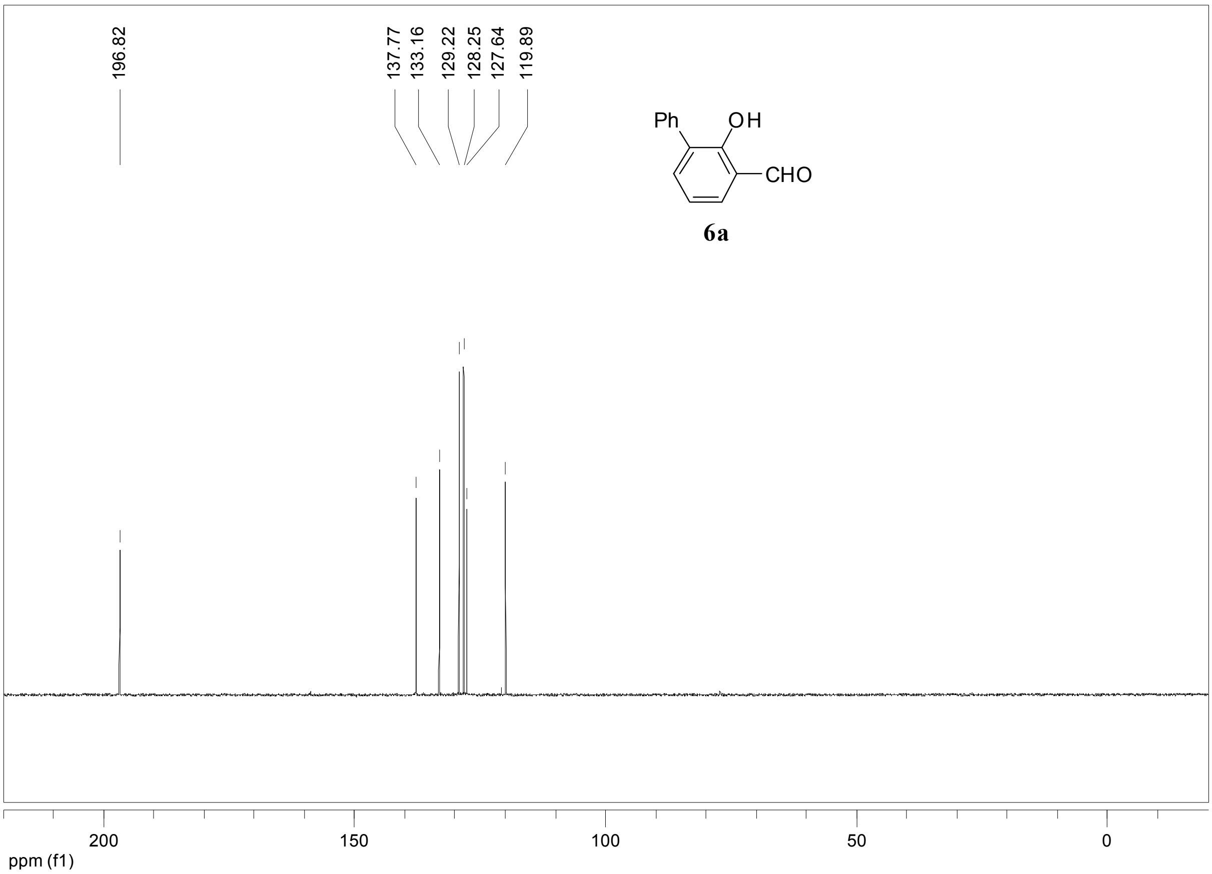

Figure S281. ^{13}C-NMR (100 MHz, $CDCl_3$) dept-135 experiment of 3-phenyl-salicylic aldehyde (**6a**).

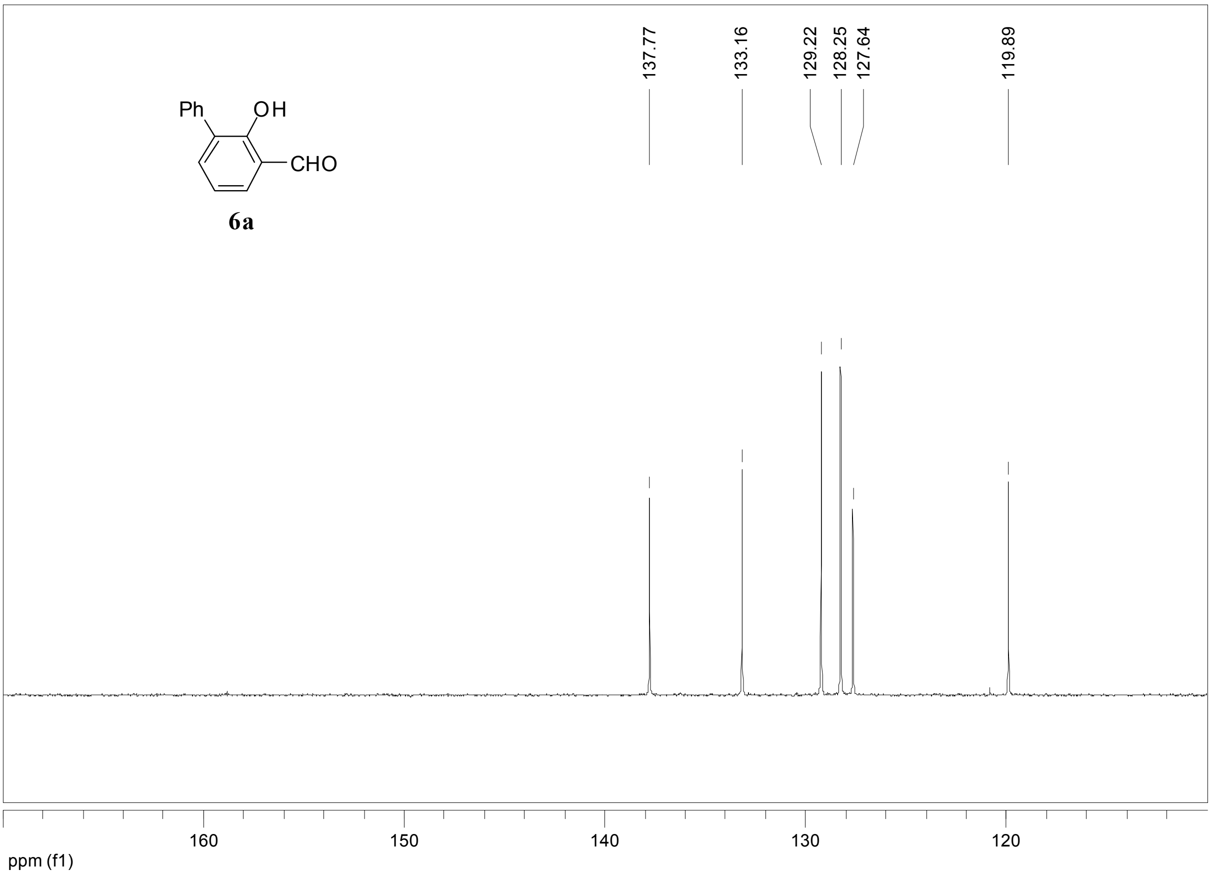

Figure S282. Expansion of ^{13}C-NMR (100 MHz, $CDCl_3$) dept-135 experiment of salicylic aldehyde **6a**.

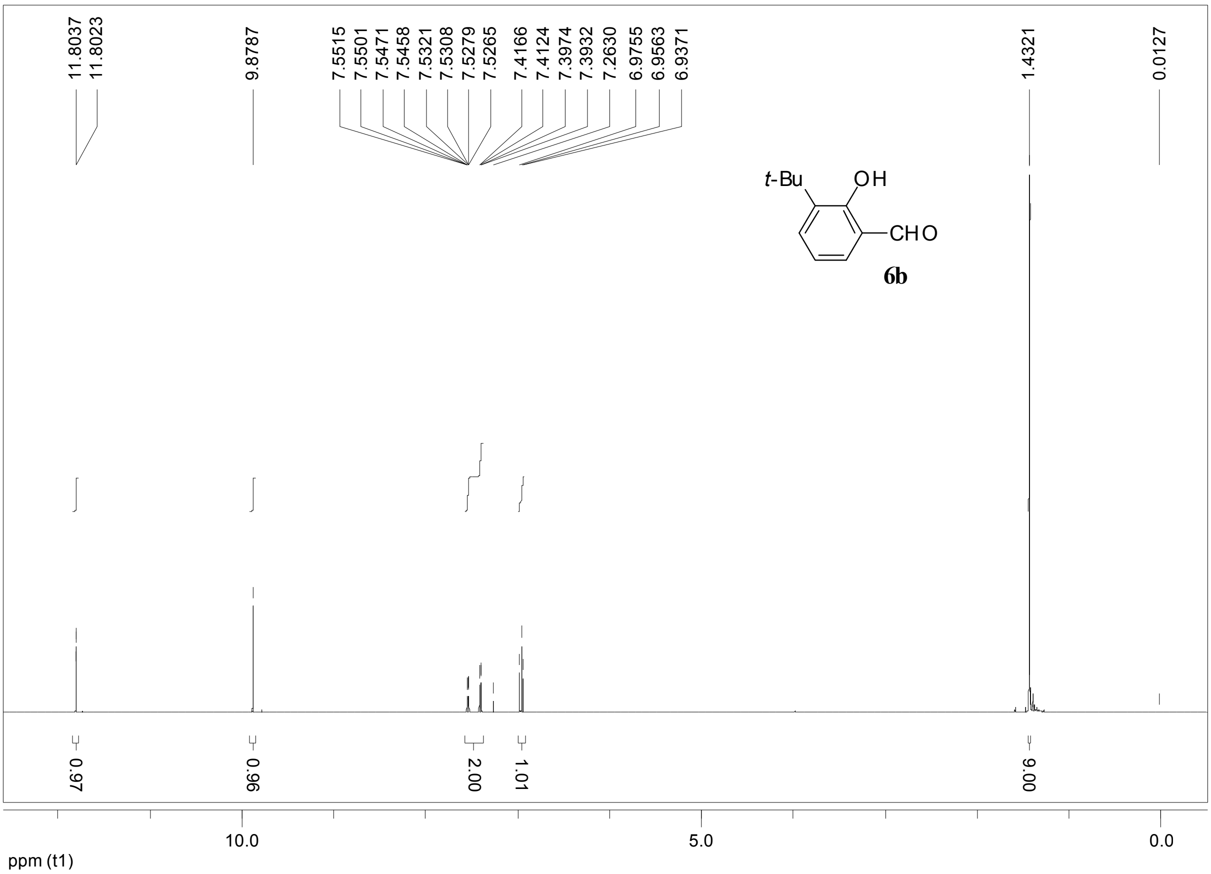

Figure S283. ^{1}H-NMR (400 MHz, $CDCl_3$) spectrum of 3-*tert*-butyl-salicylic aldehyde (**6b**).

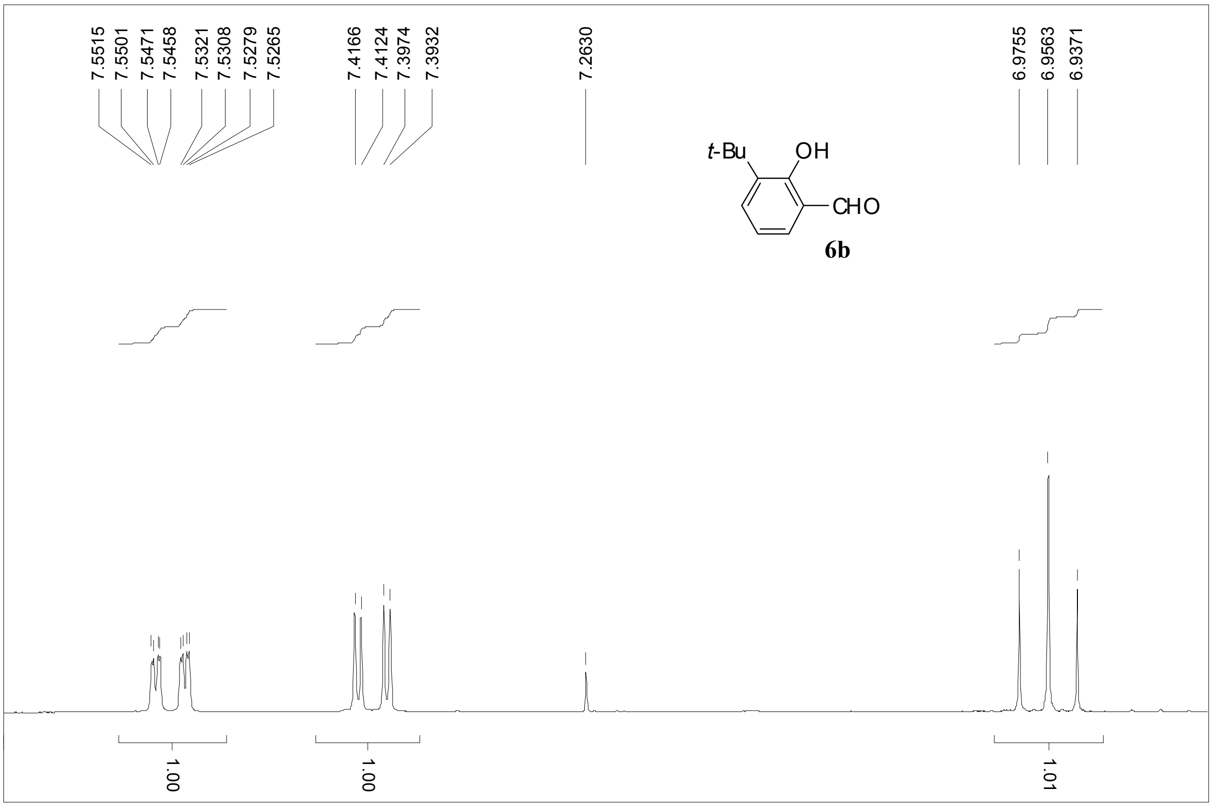

Figure S284. Expansion of ^{1}H-NMR (400 MHz, $CDCl_3$) spectrum of 3-*tert*-butyl-salicylic aldehyde (**6b**).

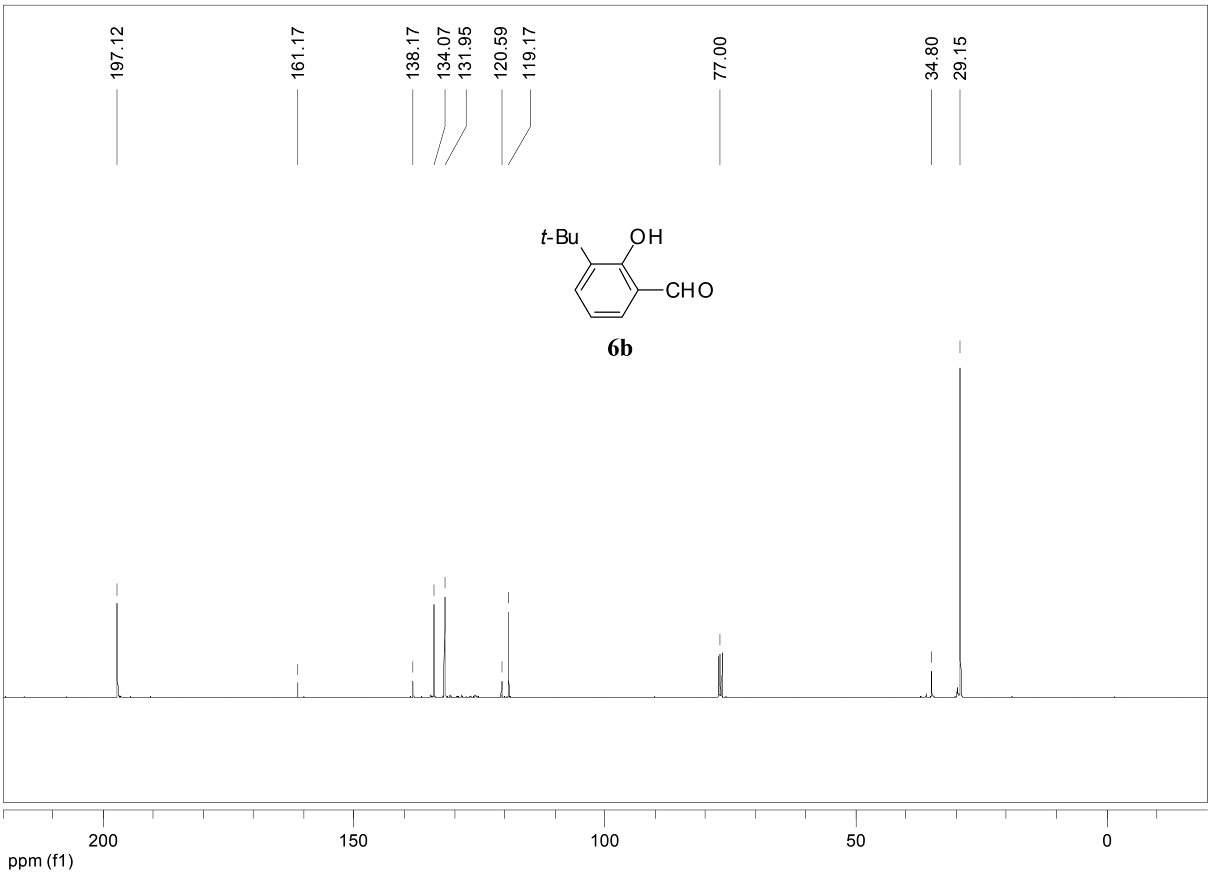

Figure S285. ^{13}C-NMR (100 MHz, $CDCl_3$) spectrum of 3-*tert*-butyl-salicylic aldehyde (**6b**).

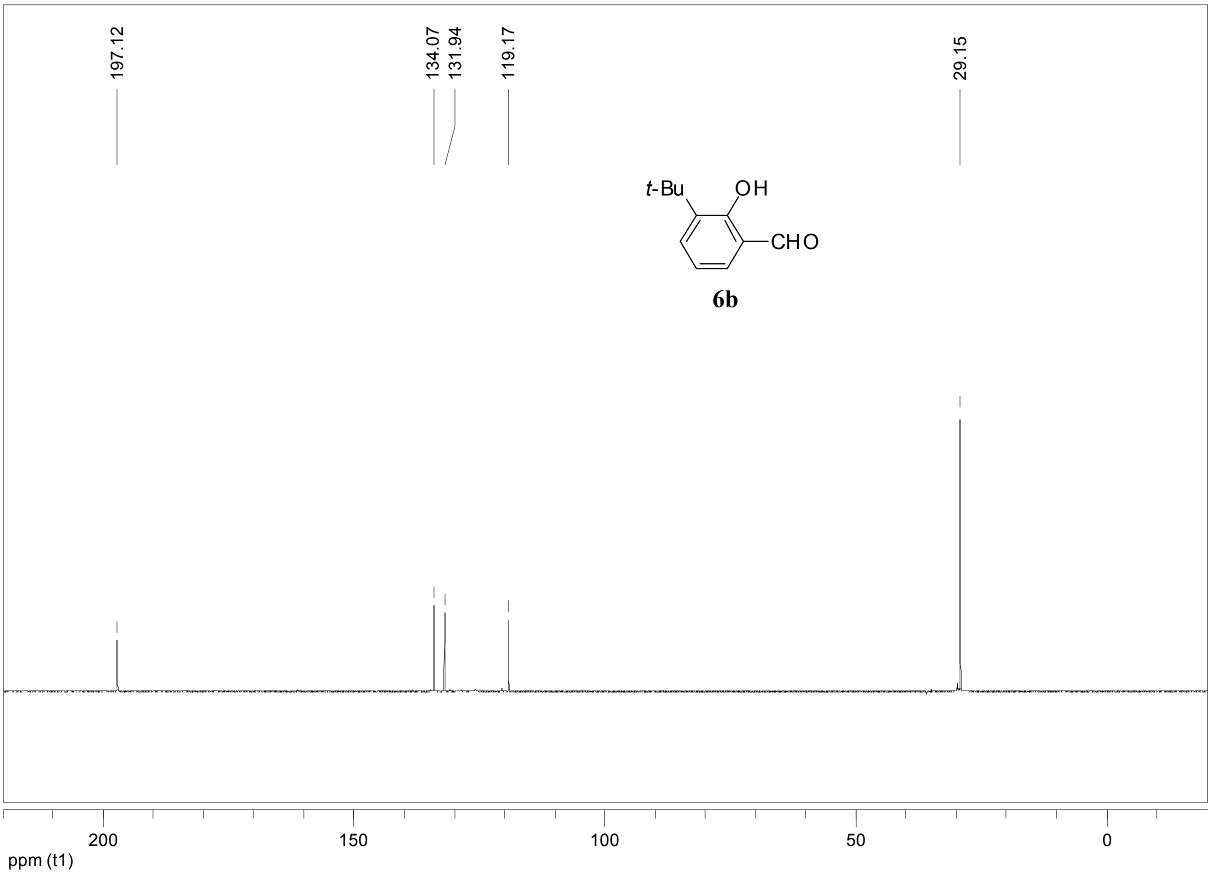

Figure S286. ^{13}C-NMR (100 MHz, $CDCl_3$) dept 135 experiment of 3-*tert*-butyl-salicylic aldehyde (**6b**).

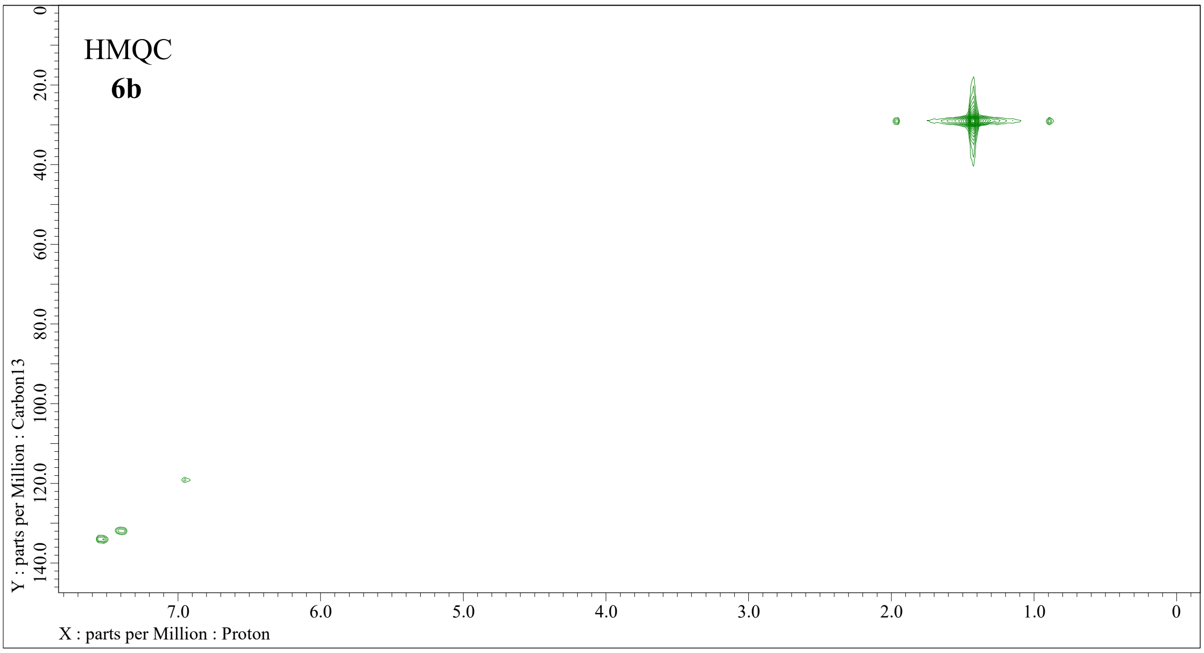

Figure S287. 2D-NMR (400 MHz, $CDCl_3$) HMQC experiment of 3-*tert*-butyl-salicylic aldehyde (**6b**).

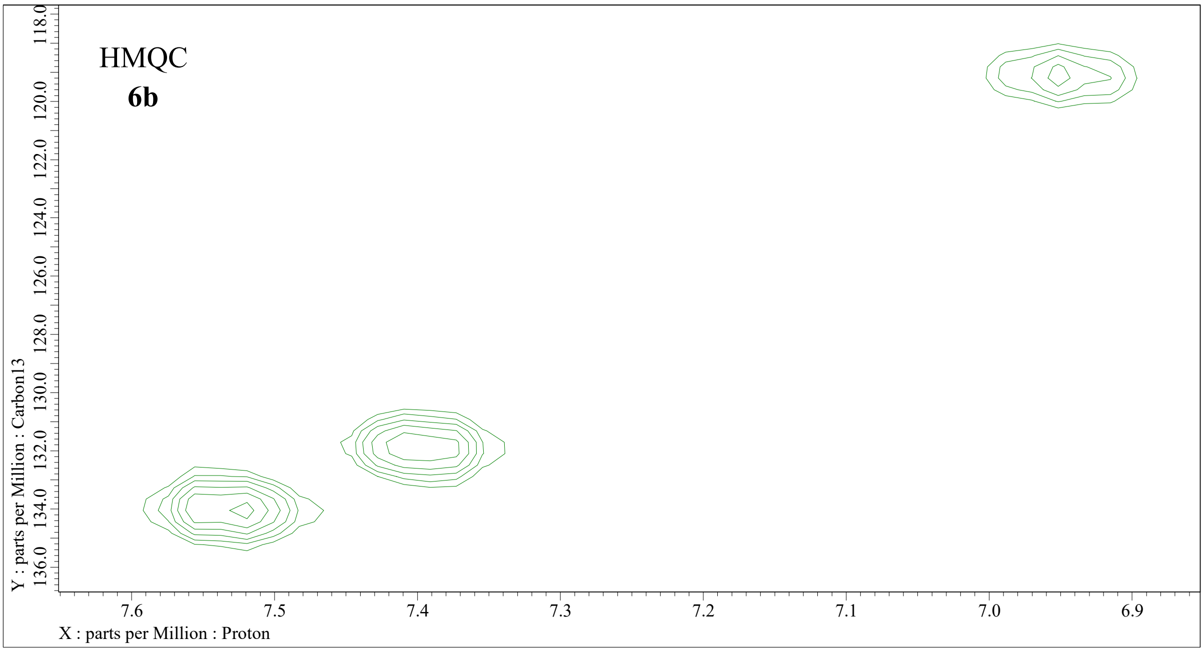

Figure S288. Expansion of 2D-NMR (400 MHz, $CDCl_3$) HMQC experiment of 3-*tert*-butyl-salicylic aldehyde (**6b**).

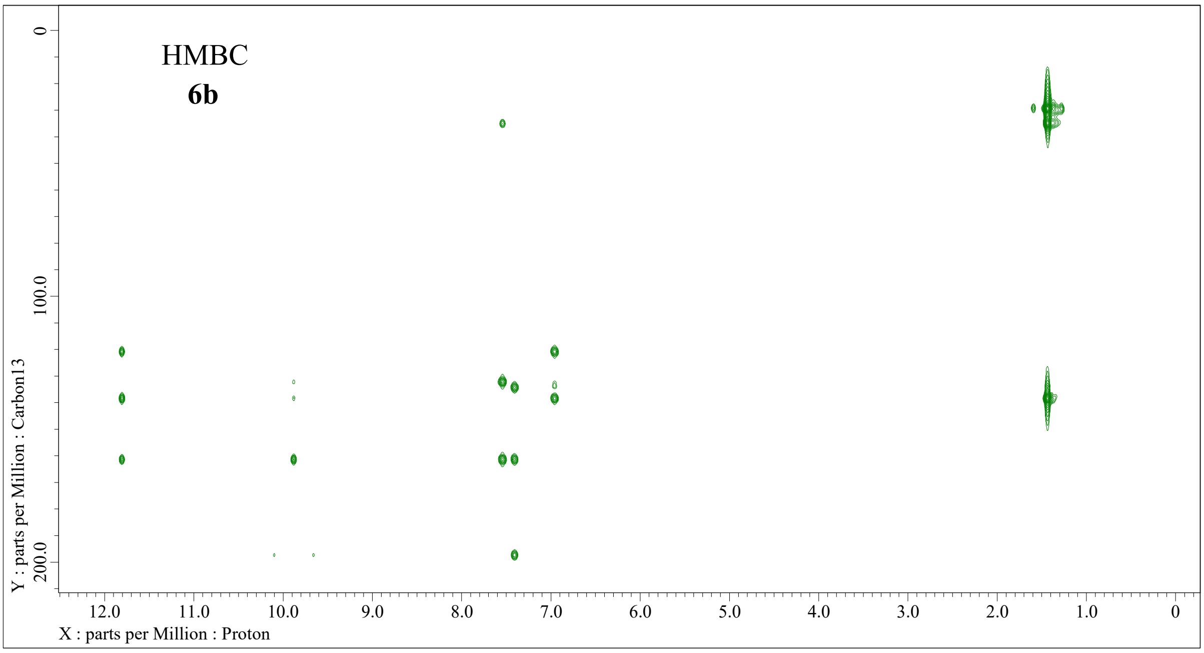

Figure S289. 2D-NMR (400 MHz, $CDCl_3$) HMBC experiment of 3-*tert*-butyl-salicylic aldehyde (**6b**).

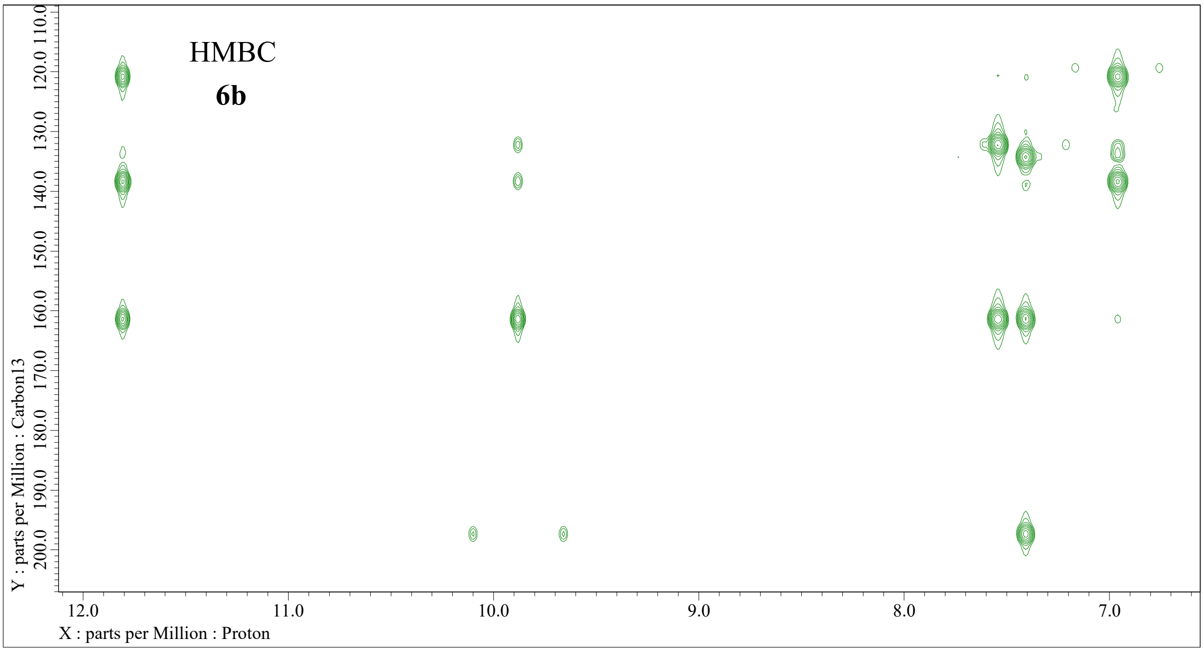

Figure S290. Expansion of 2D-NMR (400 MHz, $CDCl_3$) HMBC experiment of 3-*tert*-butyl-salicylic aldehyde (**6b**).

Molecules **2020**, *25*, 1255; doi:10.3390/molecules25051255 www.mdpi.com/journal/molecules

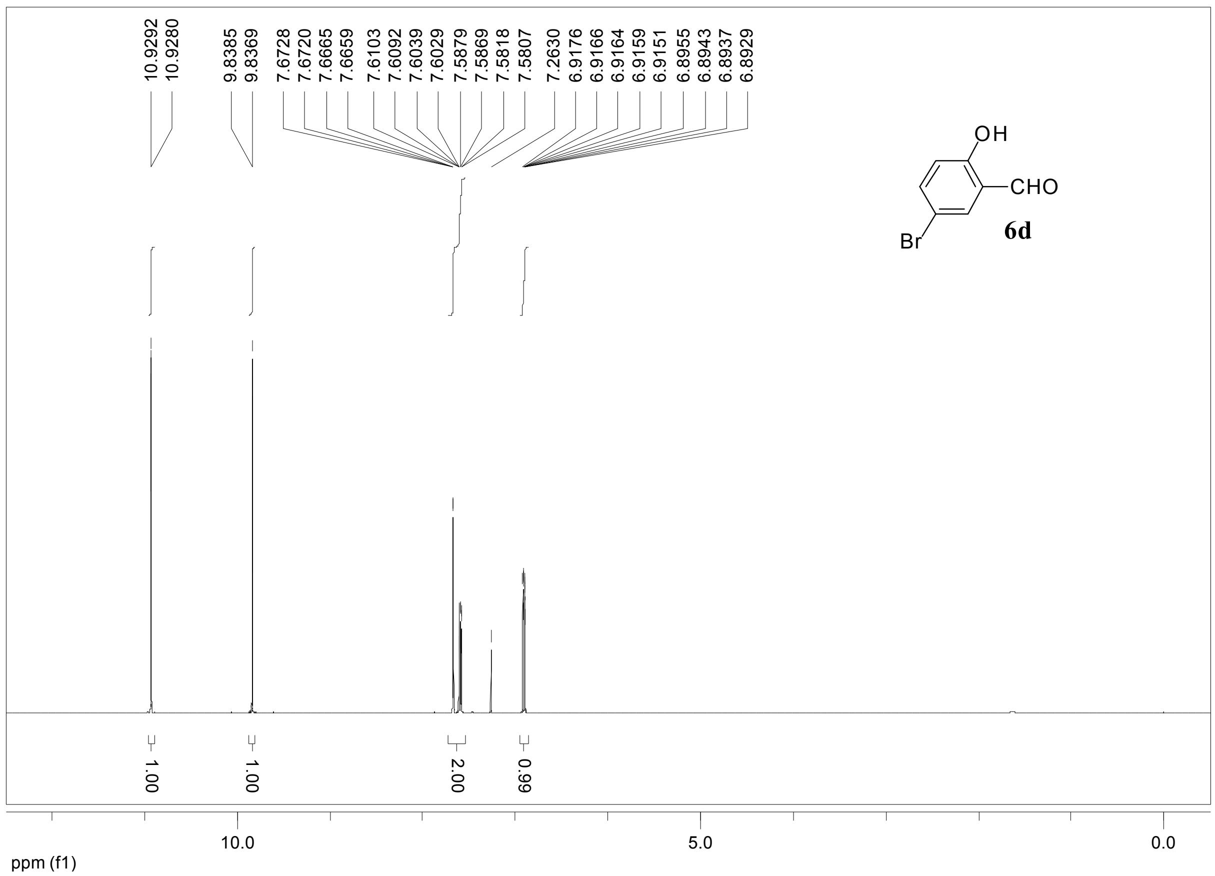

Figure S291. ¹H-NMR (400 MHz, $CDCl_3$) spectrum of 5-bromosalicylaldehyde (**6d**).

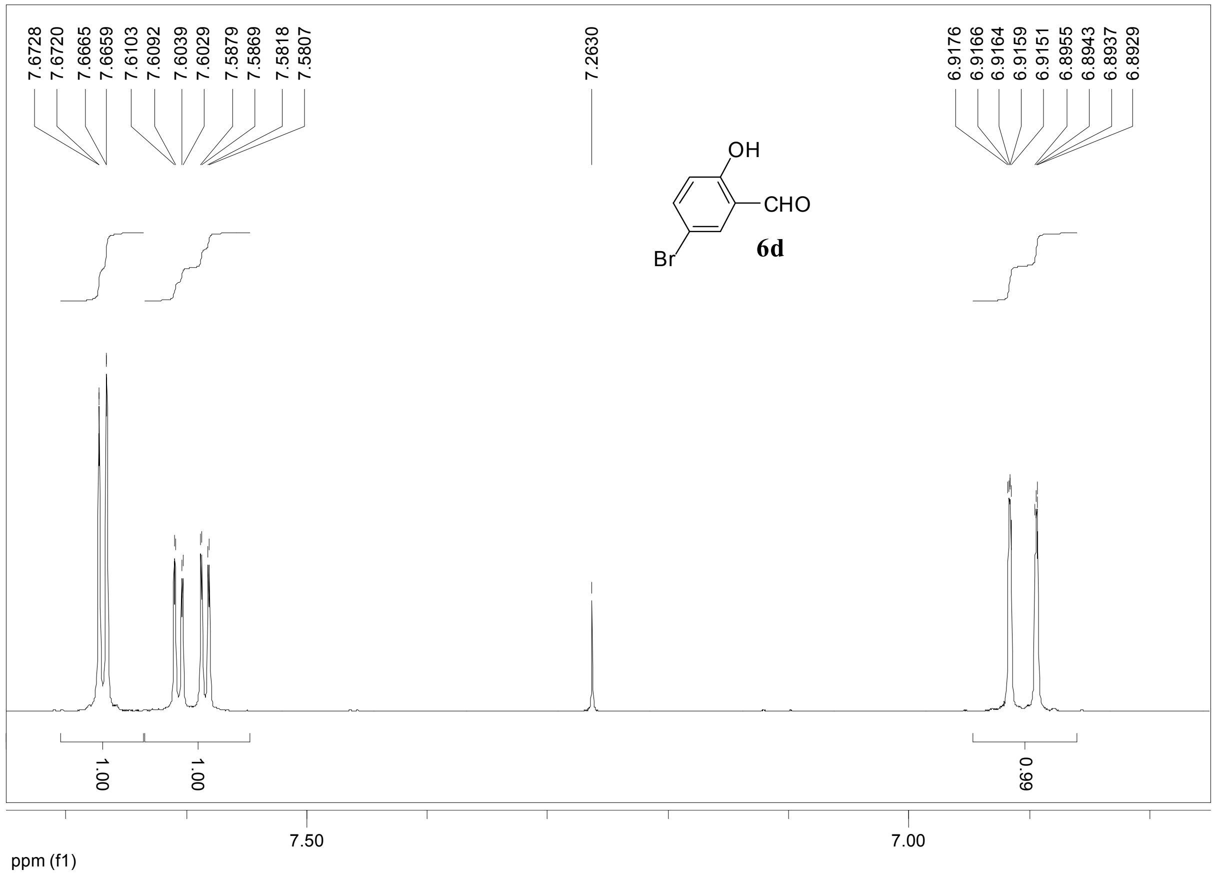

Figure S292. Expansion of ¹H-NMR (400 MHz, DMSO-*d*6) spectrum of 5-bromosalicylaldehyde (**6d**).

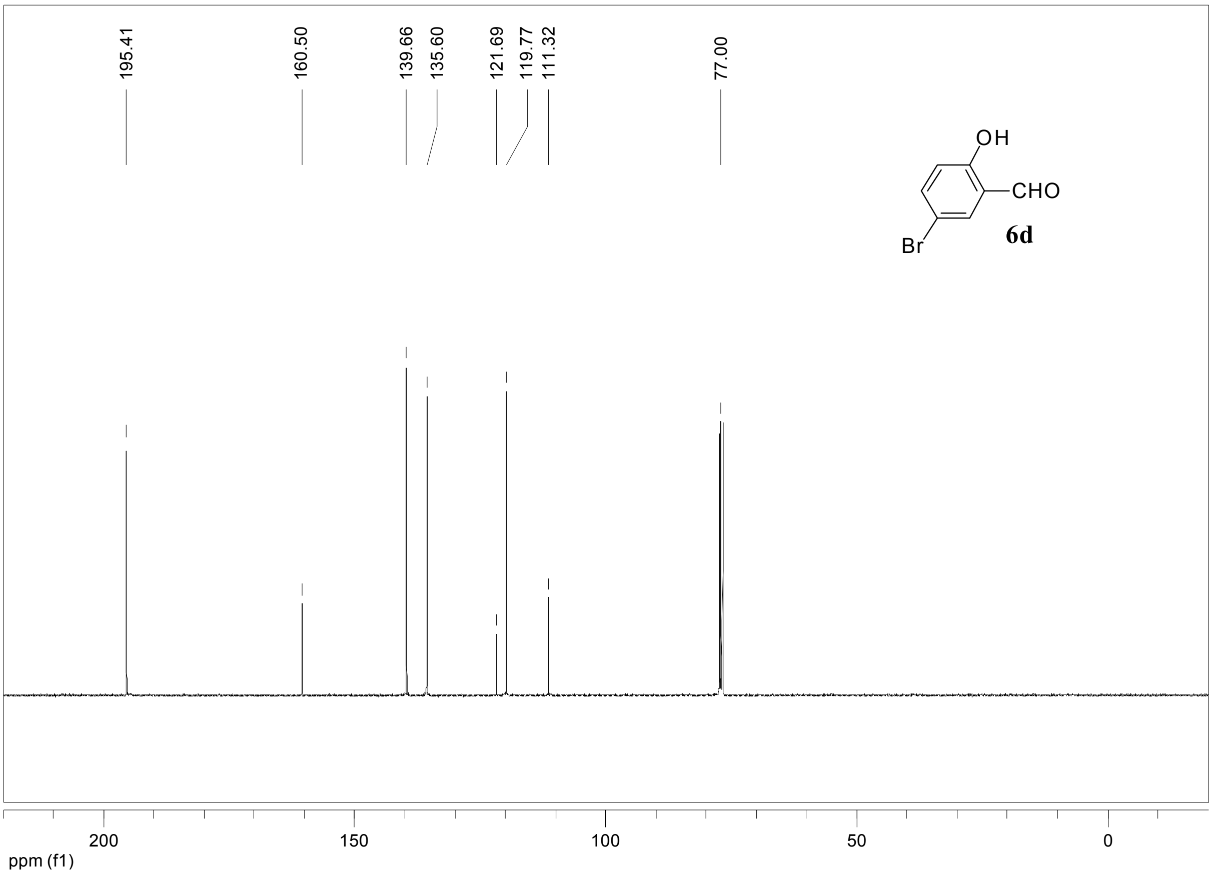

Figure S293. ^{13}C-NMR (100 MHz, DMSO-d_6) spectrum of 5-bromosalicylaldehyde (**6d**).

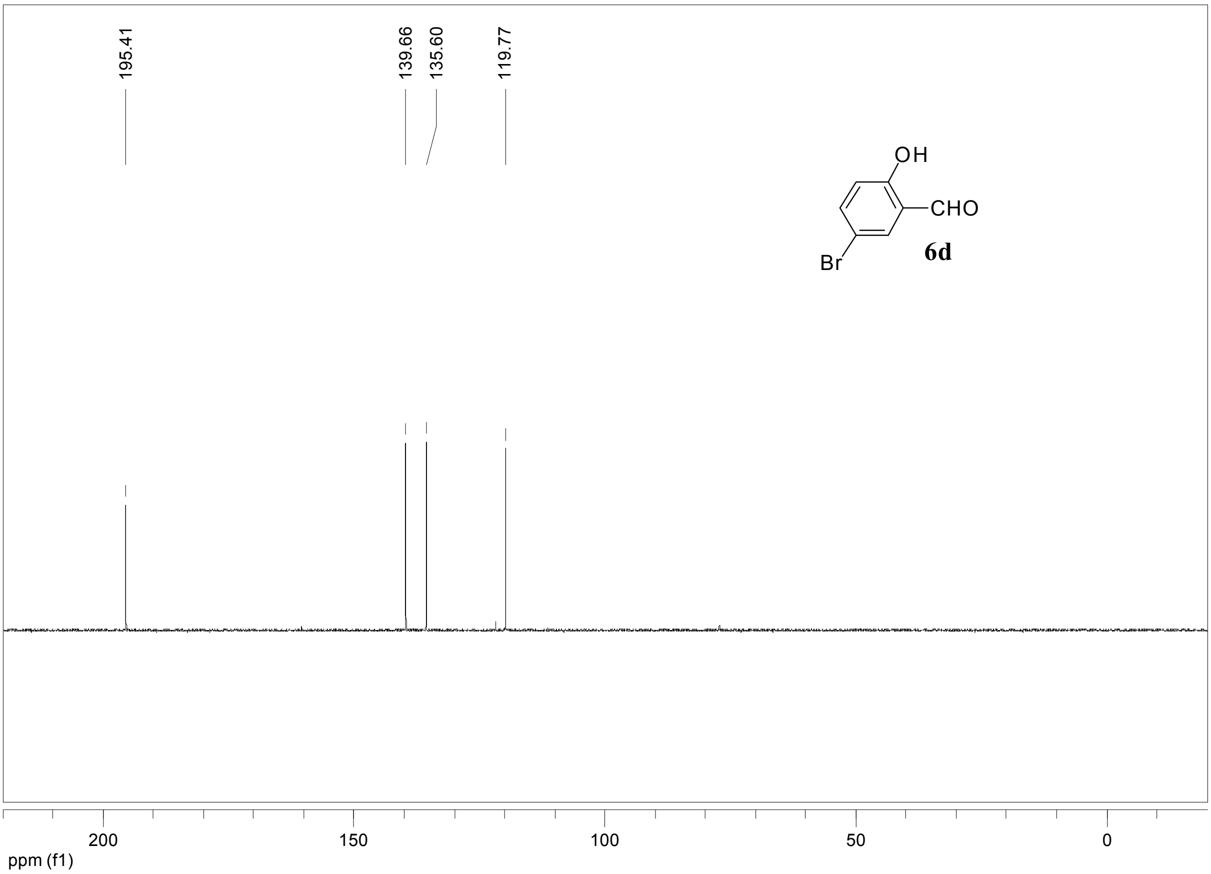

Figure S294. ^{13}C-NMR (100 MHz, DMSO-d_6) dept-135 experiment of 5-bromosalicylaldehyde (**6d**).

Molecules **2020**, *25*, 1255; doi:10.3390/molecules25051255 www.mdpi.com/journal/molecules

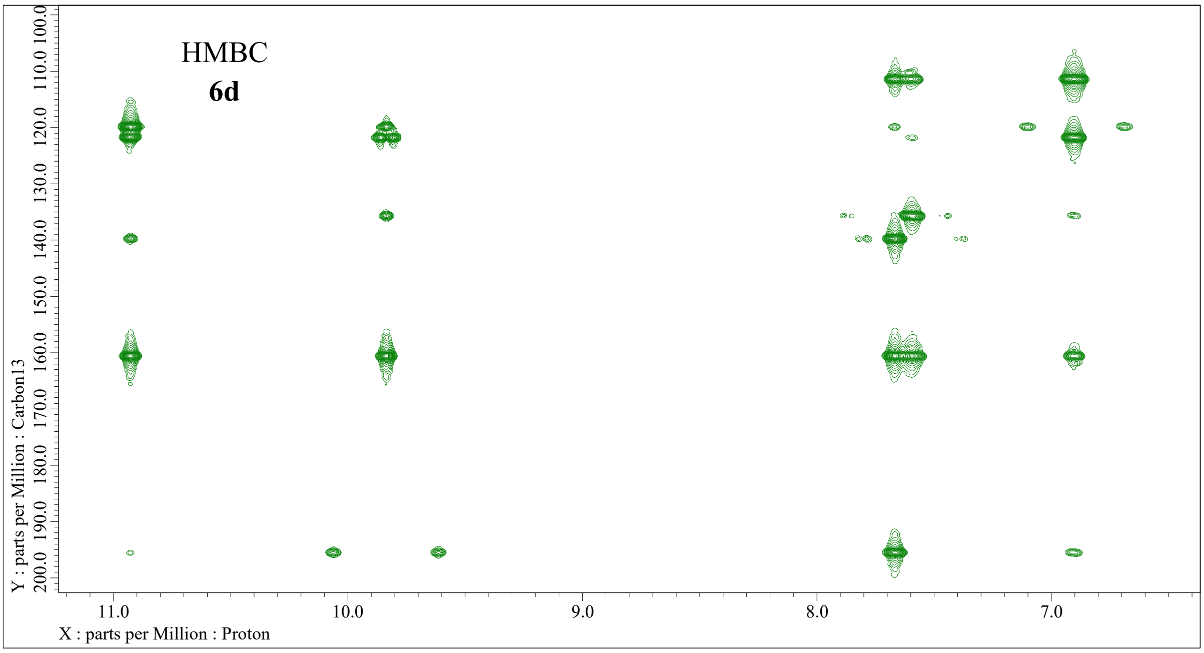

Figure S295. 2D-NMR (400 MHz, DMSO-d_6) HMBC experiment of 5-bromosalicylaldehyde (**6d**).

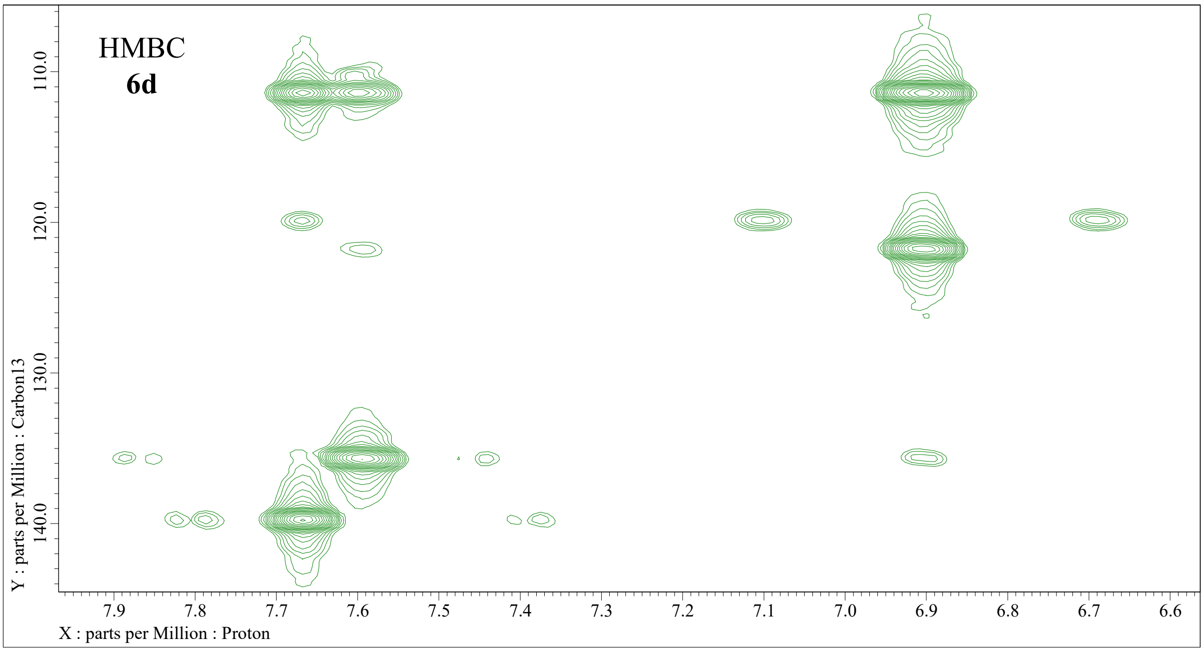

Figure S296. Expansion of 2D-NMR (400 MHz, DMSO-d_6) HMBC experiment of 5-bromosalicylaldehyde (**6d**).

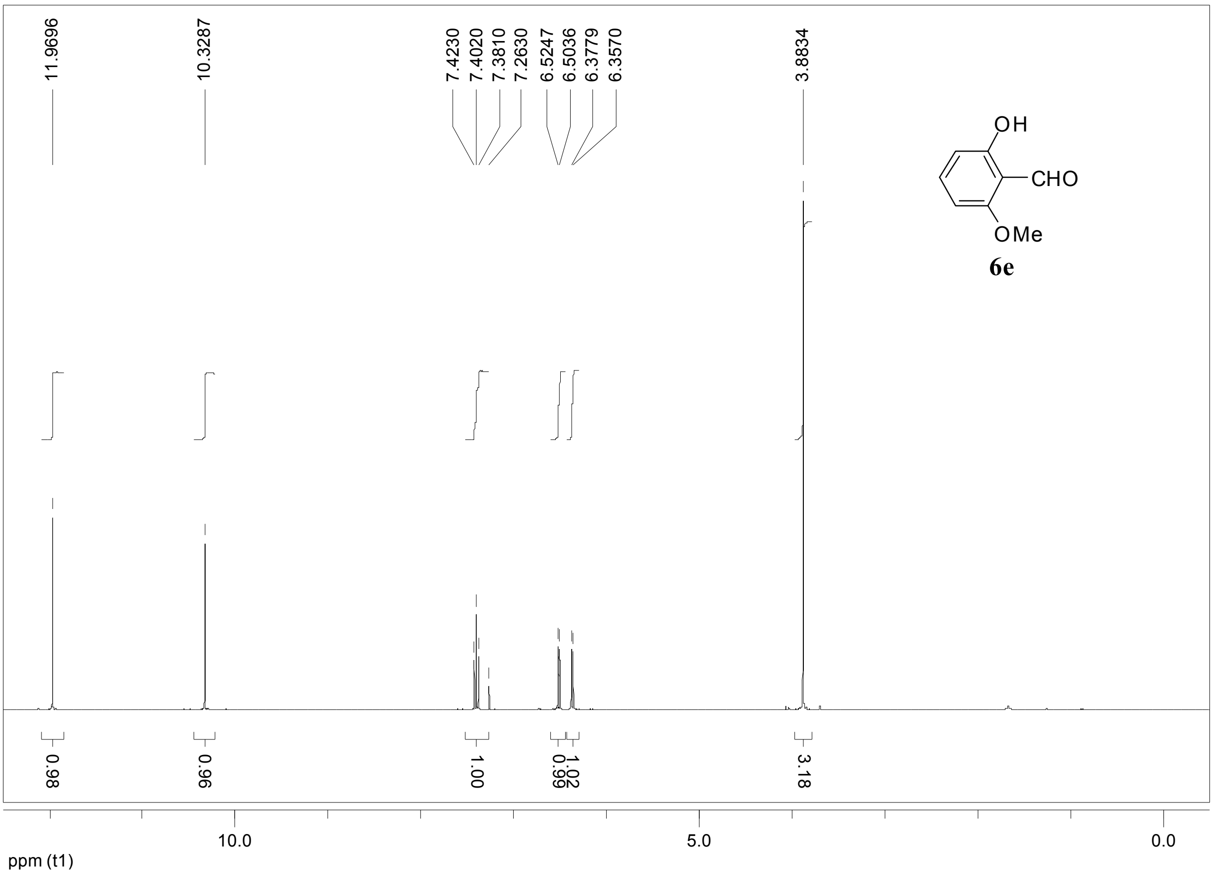

Figure S297. ^{1}H-NMR (400 MHz, $CDCl_3$) spectrum of 6-methoxy-salicylic aldehyde (**6e**).

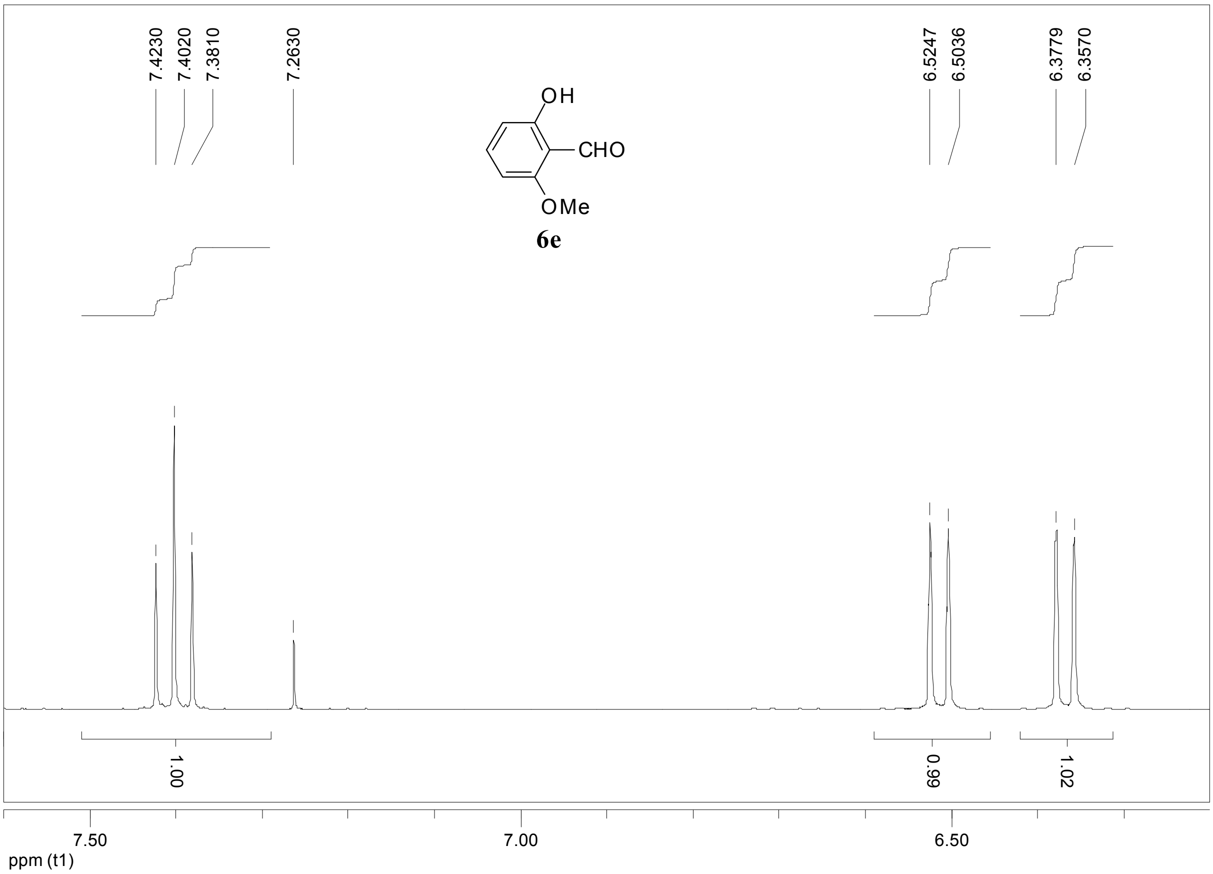

Figure S298. Expansion of ^{1}H-NMR (400 MHz, $CDCl_3$) spectrum of 6-methoxy-salicylic aldehyde (**6e**).

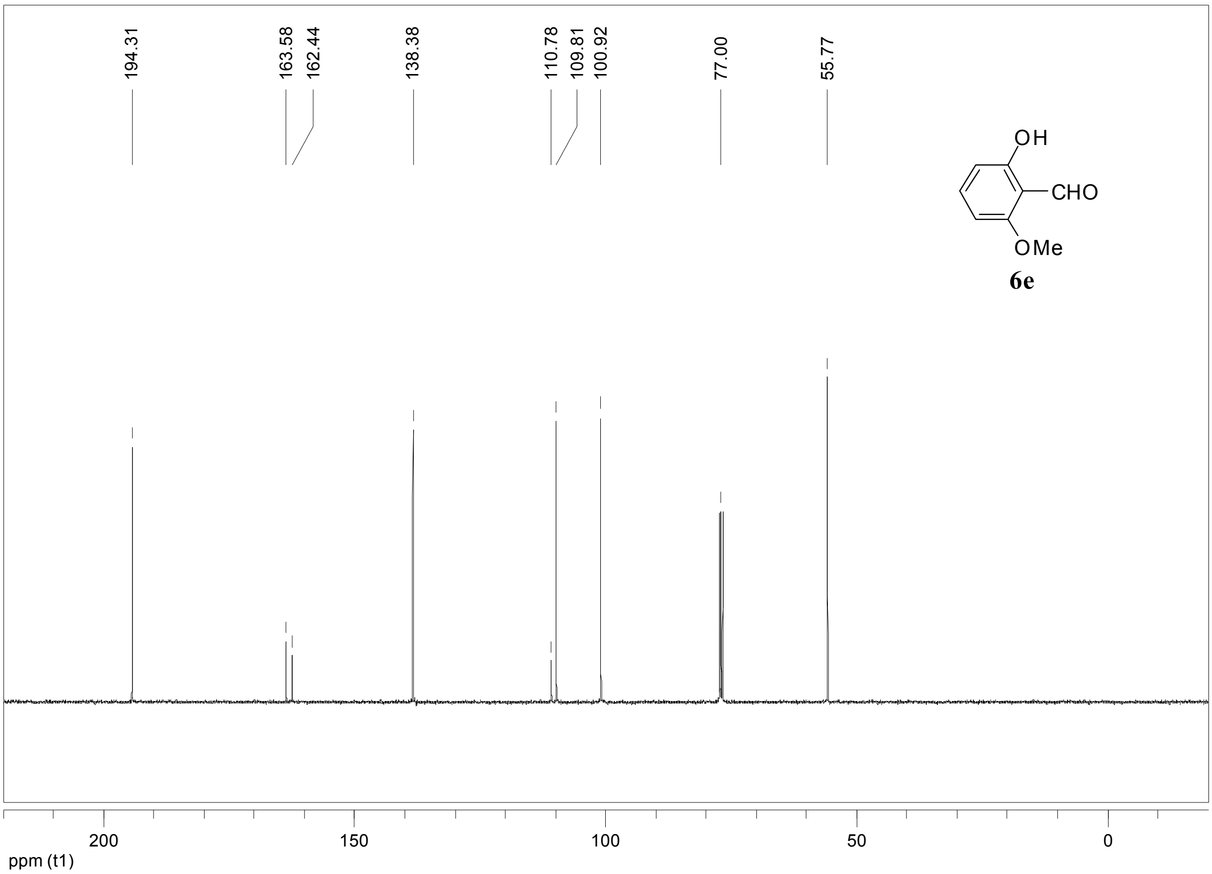

Figure S299. ^{13}C-NMR (100 MHz, $CDCl_3$) spectrum of 6-methoxy-salicylic aldehyde (**6e**).

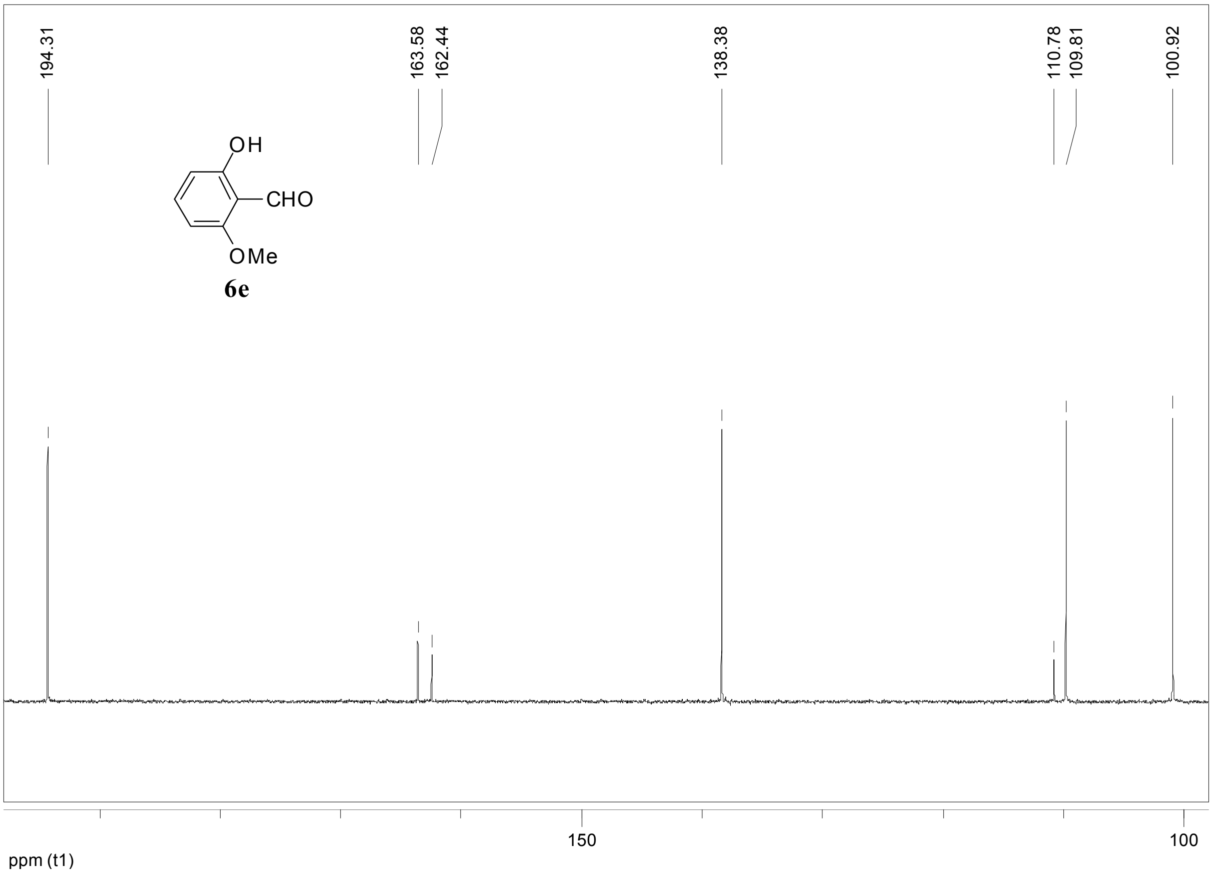

Figure S300. Expansion of ^{13}C-NMR (100 MHz, $CDCl_3$) spectrum of 6-methoxy-salicylic aldehyde (**6e**).

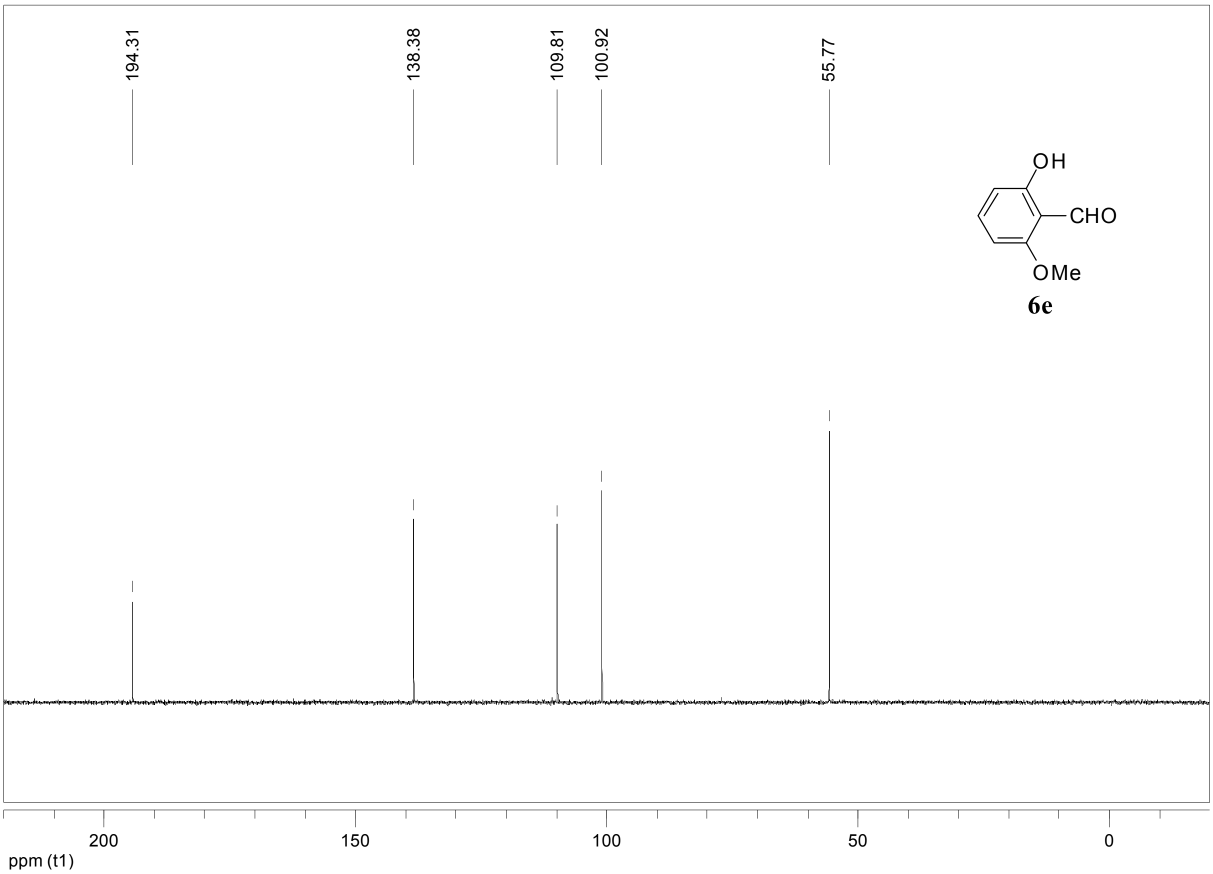

Figure S301. ^{13}C-NMR (100 MHz, $CDCl_3$) dept-135 experiment of 6-methoxy-salicylic aldehyde (**6e**).

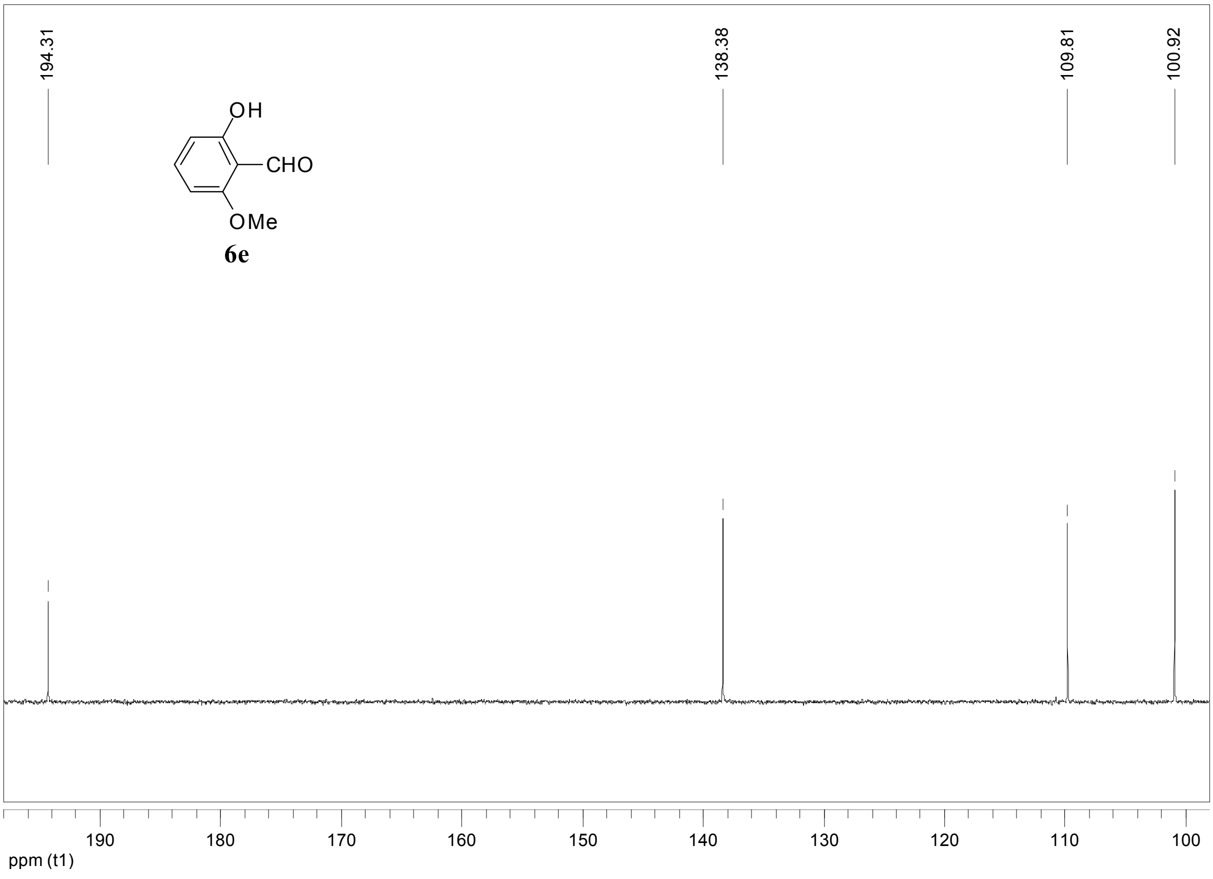

Figure S302. Expansion of ^{13}C-NMR (100 MHz, $CDCl_3$) dept-135 experiment of salicylic aldehyde **6e**.

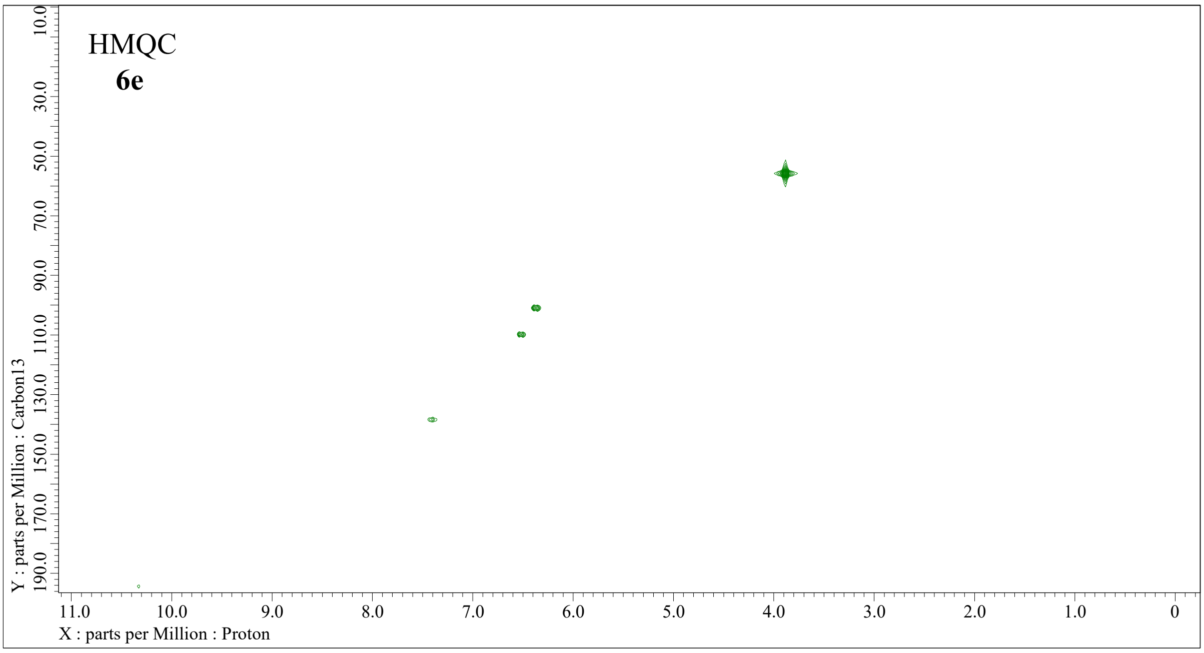

Figure S303. 2D-NMR (400 MHz, $CDCl_3$) HMQC experiment of 6-methoxy-salicylic aldehyde (**6e**).

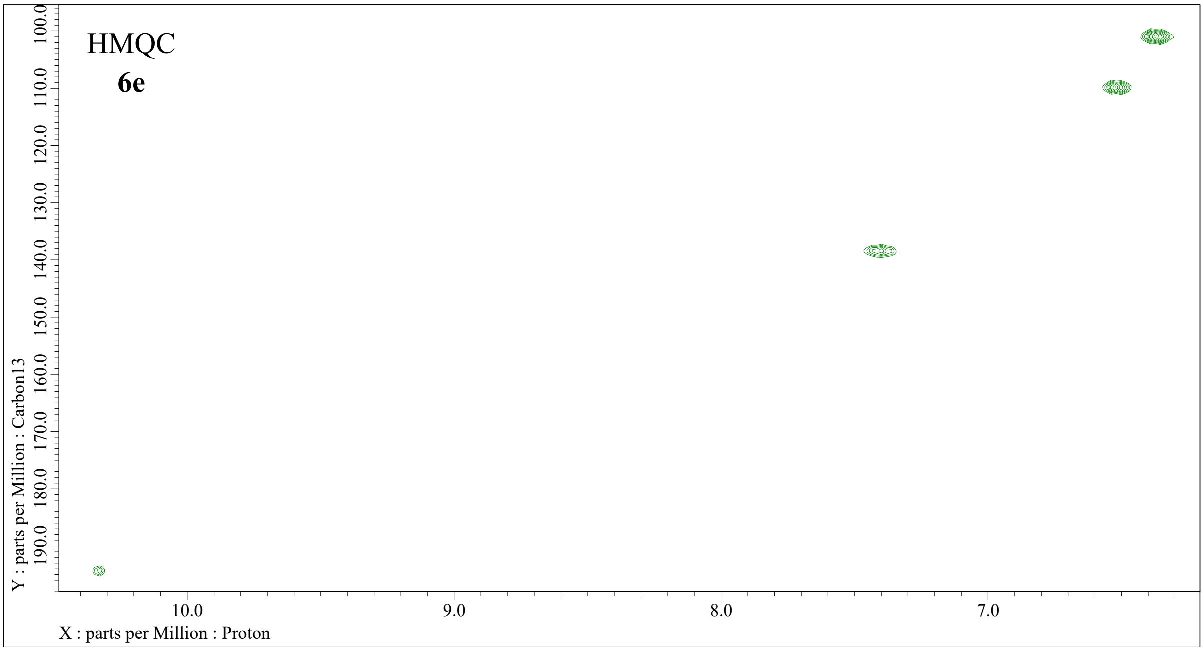

Figure S304. Expansion of 2D-NMR (400 MHz, $CDCl_3$) HMQC experiment of 6-methoxy-salicylic aldehyde (**6e**).

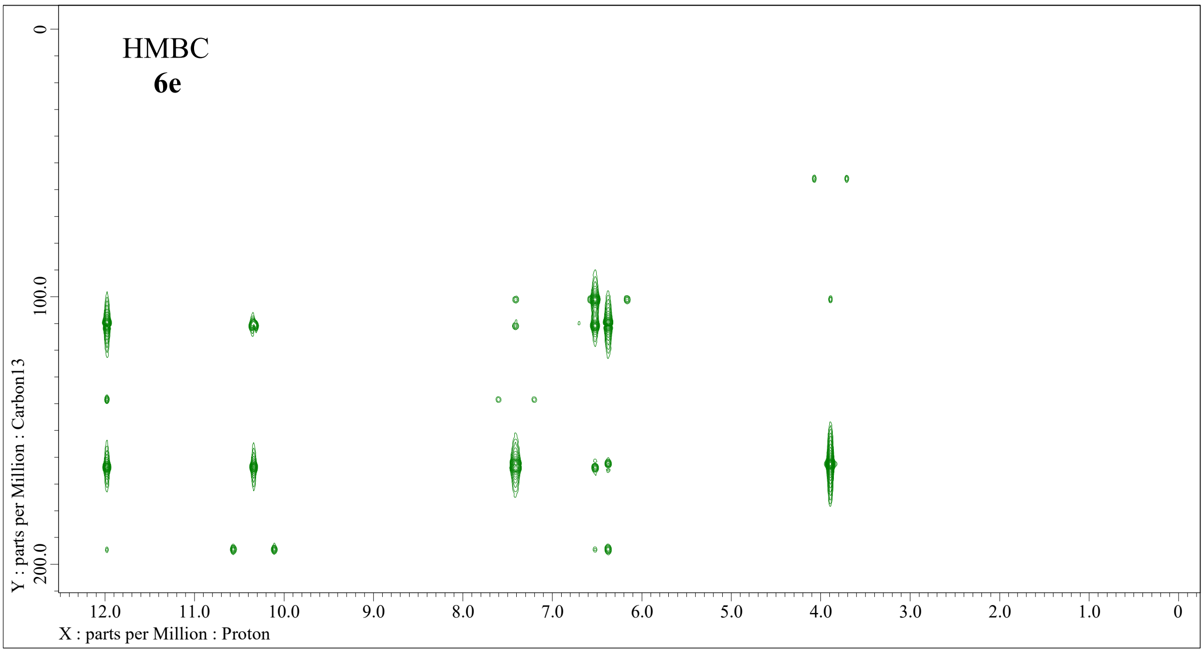

Figure S305. 2D-NMR (400 MHz, $CDCl_3$) HMBC experiment of 6-methoxy-salicylic aldehyde (**6e**).

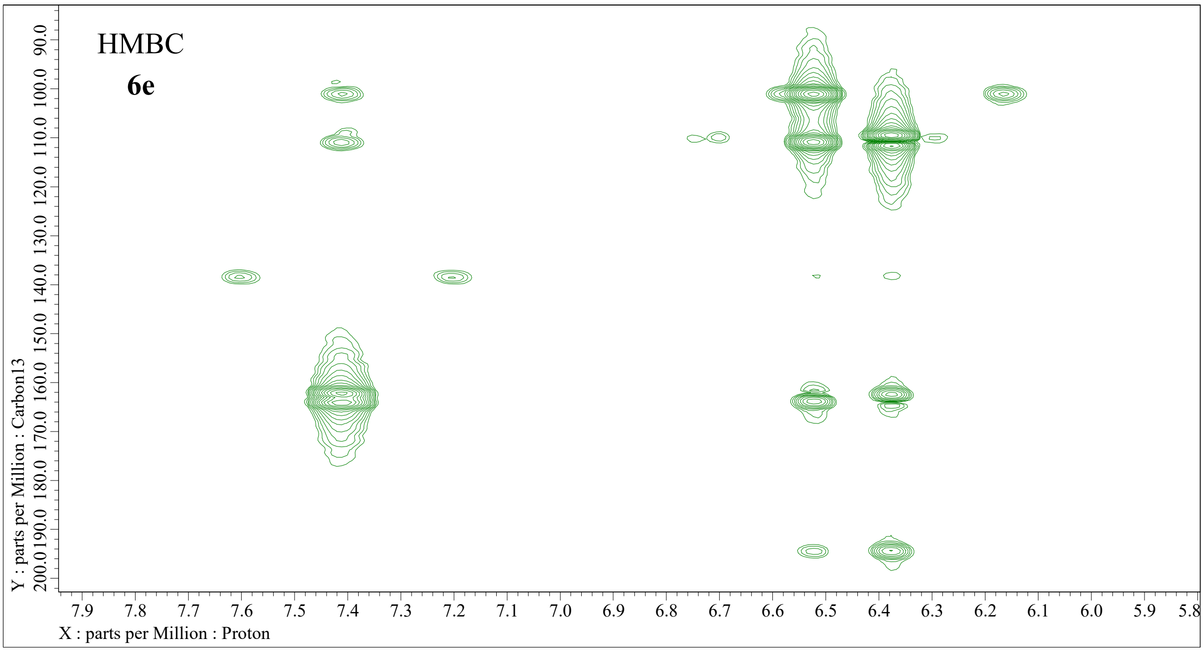

Figure S306. Expansion of 2D-NMR (400 MHz, $CDCl_3$) HMBC experiment of 6-methoxy-salicylic aldehyde (**6e**).

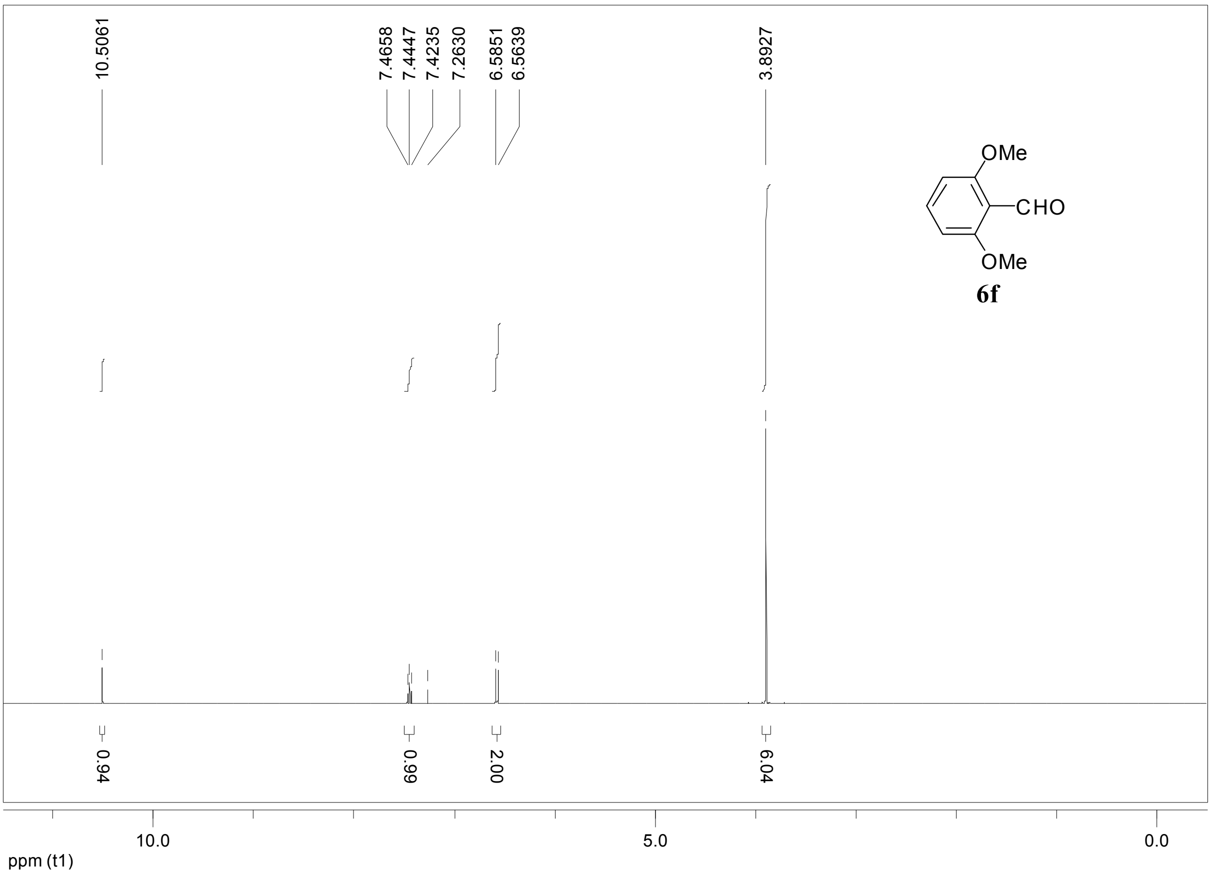

Figure S307. ^{1}H-NMR (400 MHz, $CDCl_3$) spectrum of 2,6-dimethoxybenzaldehyde (**6f**).

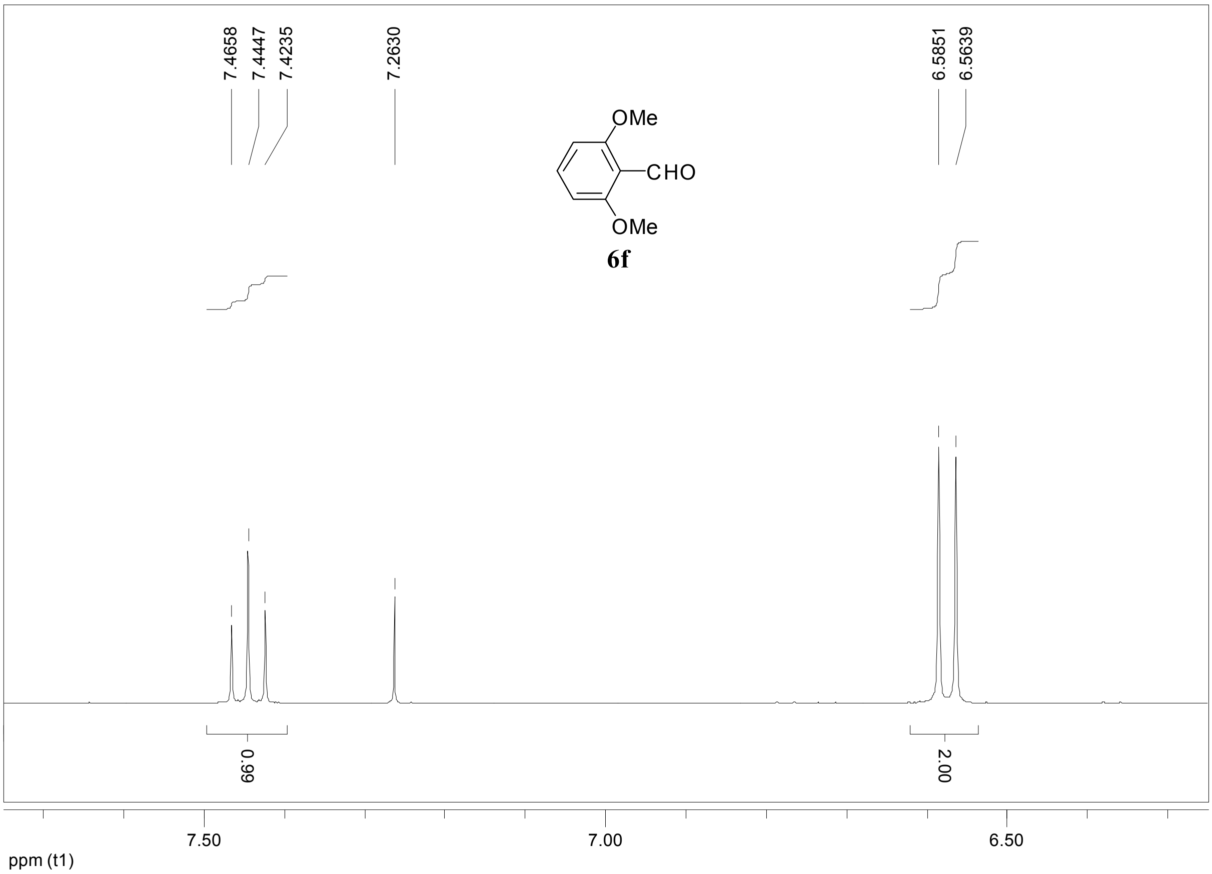

Figure S308. Expansion of ^{1}H-NMR (400 MHz, $CDCl_3$) spectrum of 2,6-dimethoxybenzaldehyde (**6f**).

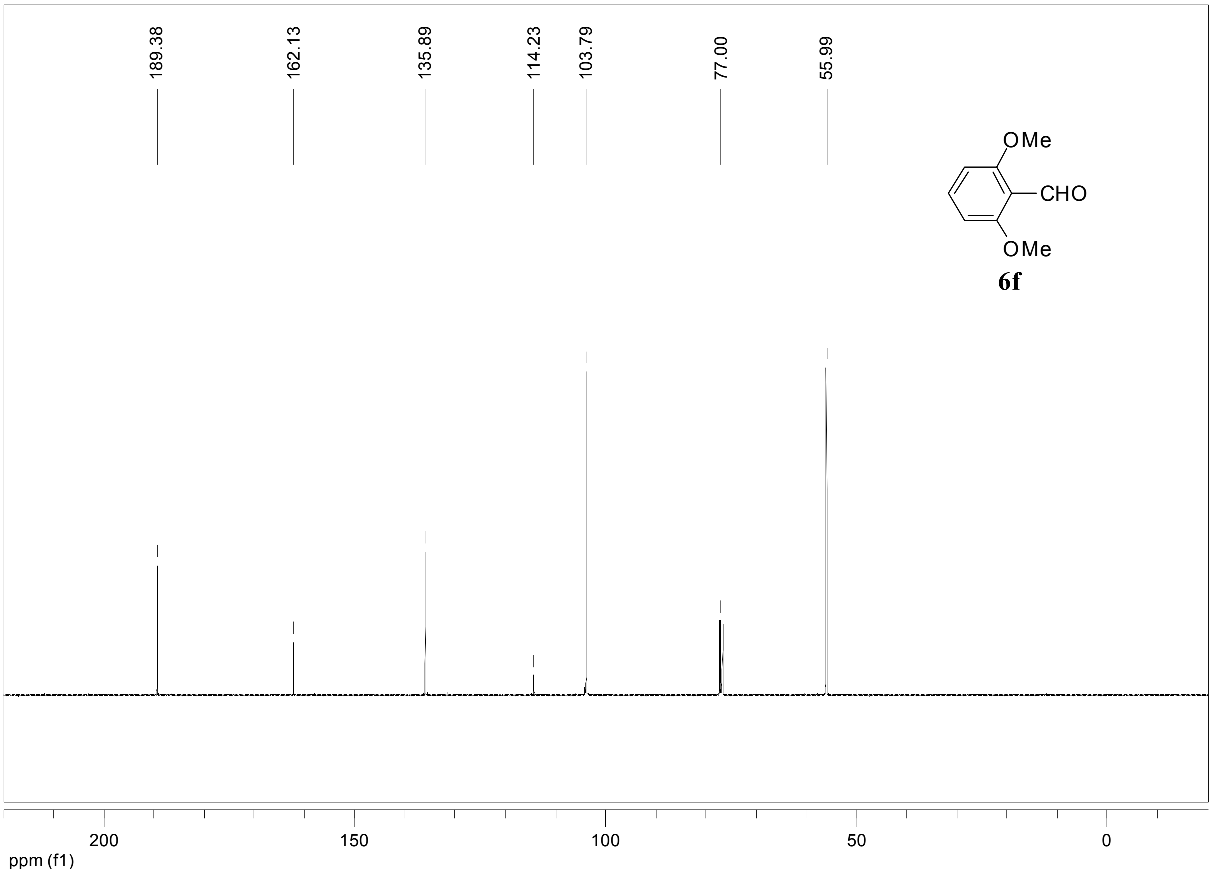

Figure S309. ^{13}C-NMR (100 MHz, $CDCl_3$) spectrum of 2,6-dimethoxybenzaldehyde (**6f**).

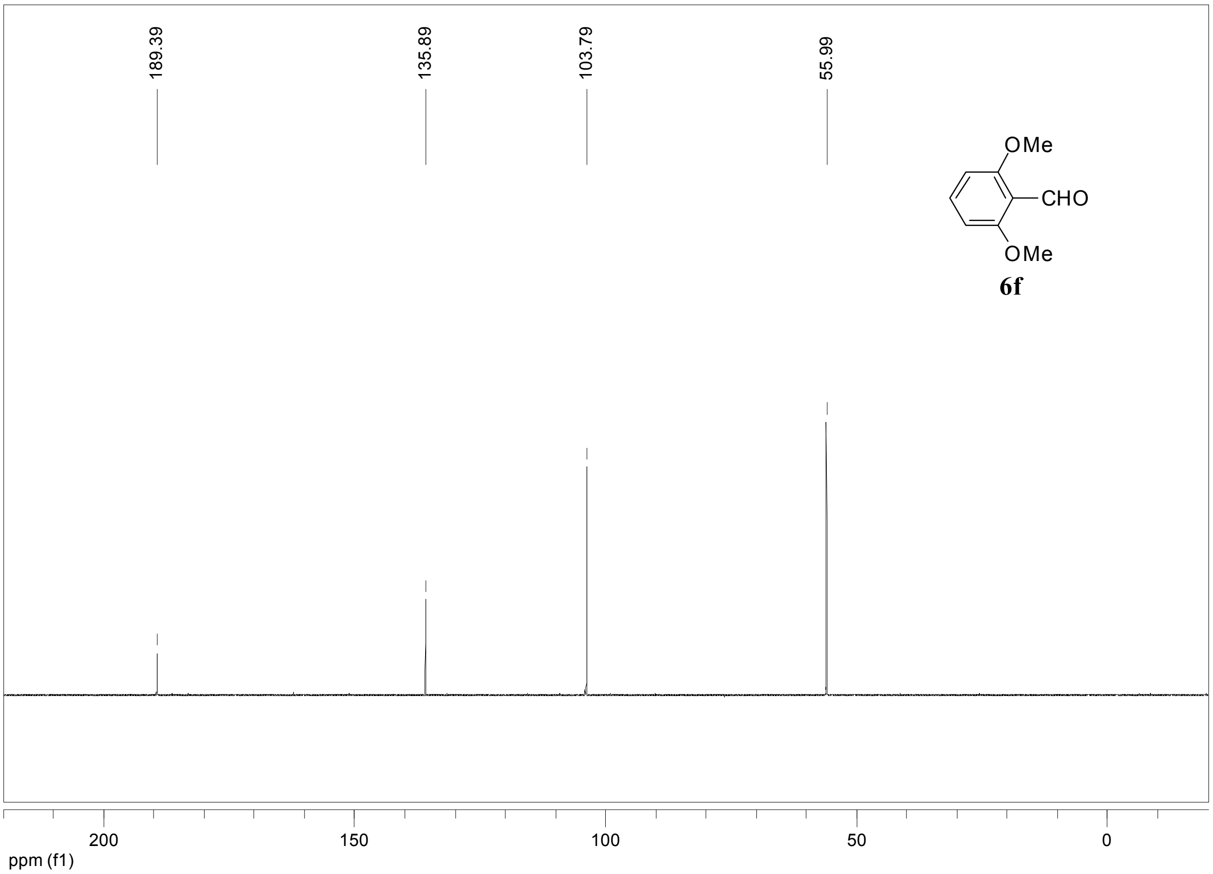

Figure S310. ^{13}C-NMR (100 MHz, $CDCl_3$) dept-135 experiment of 2,6-dimethoxybenzaldehyde (**6f**).

Molecules **2020**, *25*, 1255; doi:10.3390/molecules25051255 www.mdpi.com/journal/molecules

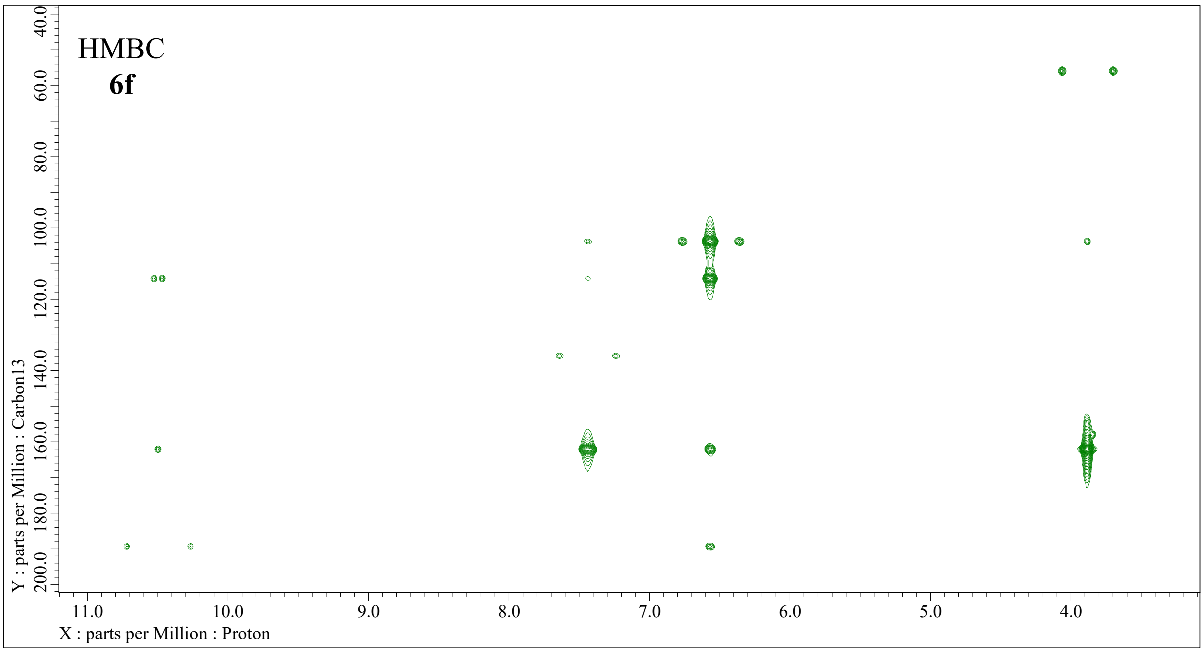

Figure S311. 2D-NMR (400 MHz, $CDCl_3$) HMBC experiment of 2,6-dimethoxybenzaldehyde (**6f**).

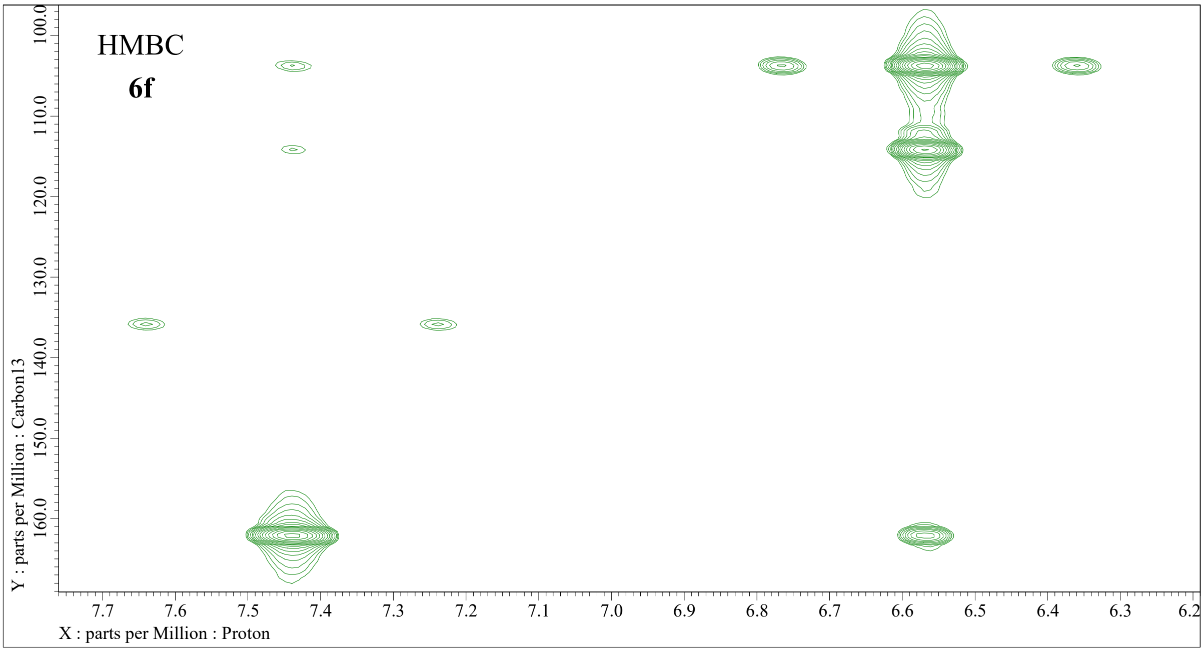

Figure S312. Expansion of 2D-NMR (400 MHz, $CDCl_3$) HMBC experiment of 2,6-dimethoxybenzaldehyde (**6f**).

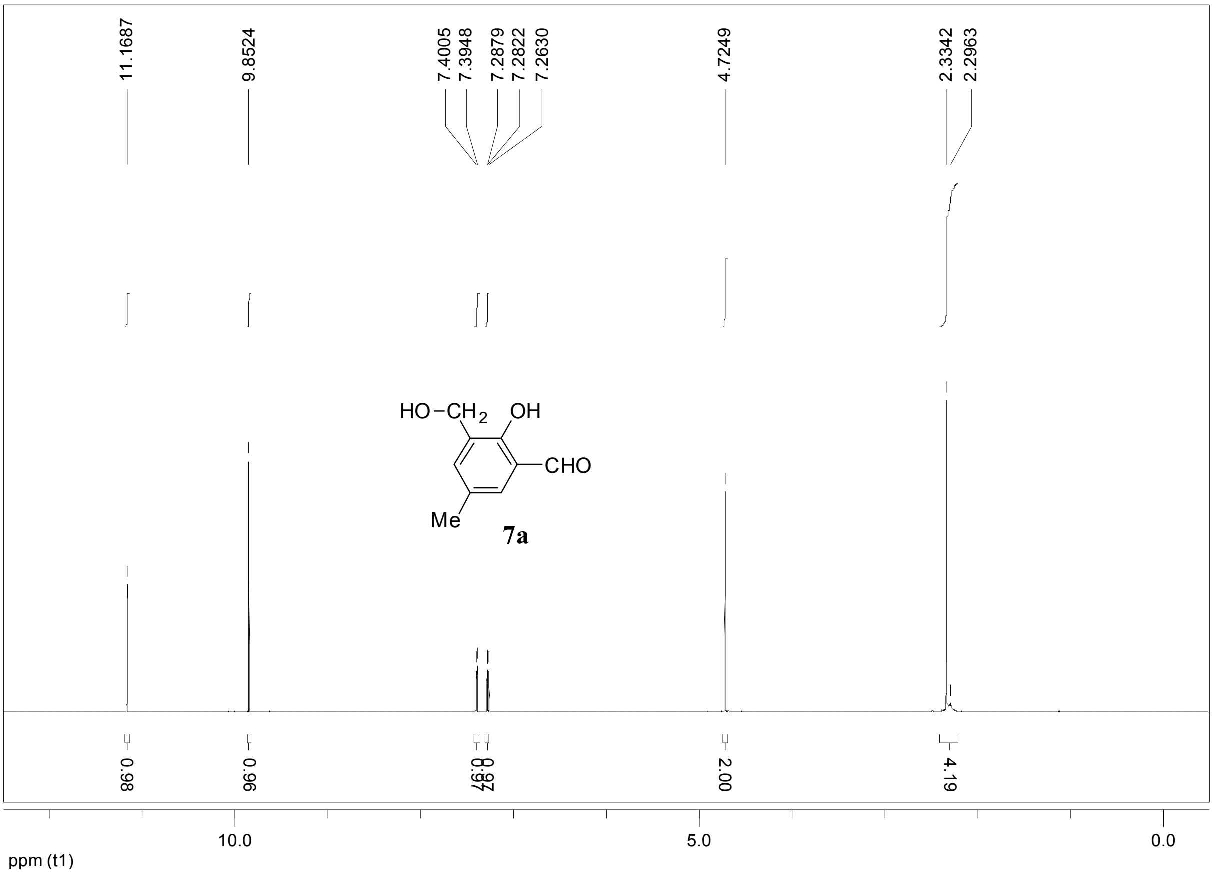

Figure S313. ^{1}H-NMR (400 MHz, $CDCl_3$) spectrum of hydroxymethyl-5-methyl-salicylic aldehyde **7a**.

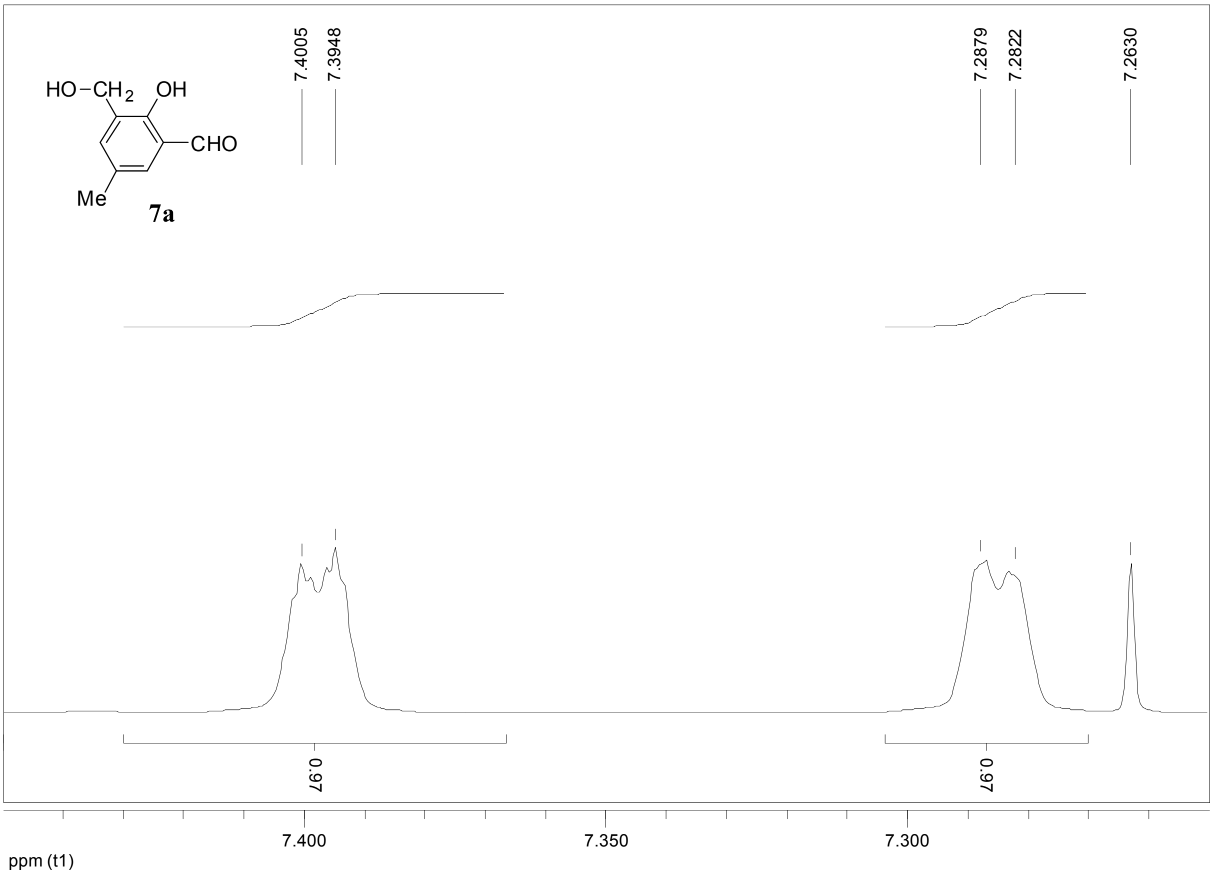

Figure S314. Expansion of ^{1}H-NMR (400 MHz, $CDCl_3$) spectrum of salicylic aldehyde **7a**.

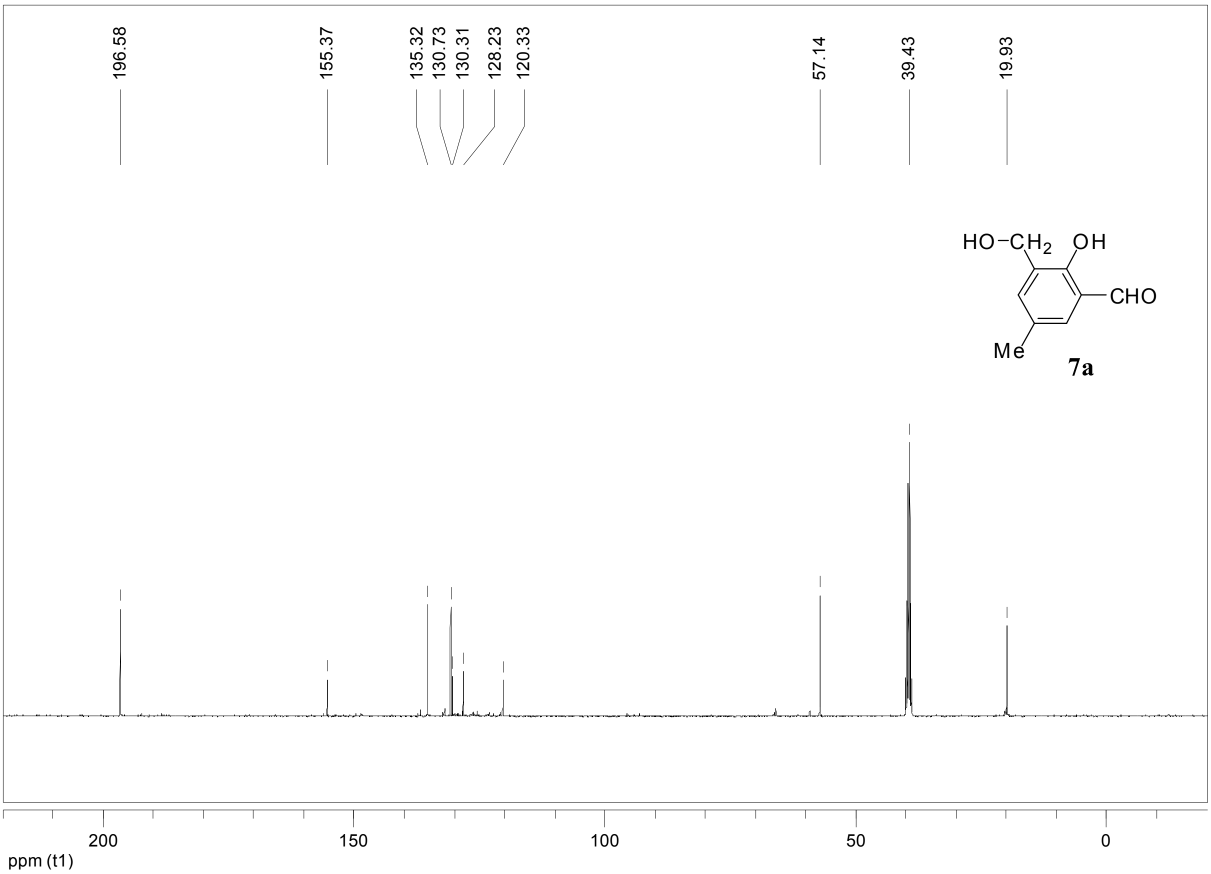

Figure S315. ^{13}C-NMR (100 MHz, DMSO-d_6) spectrum of 3-hydroxymethyl-salicylic aldehyde **7a**.

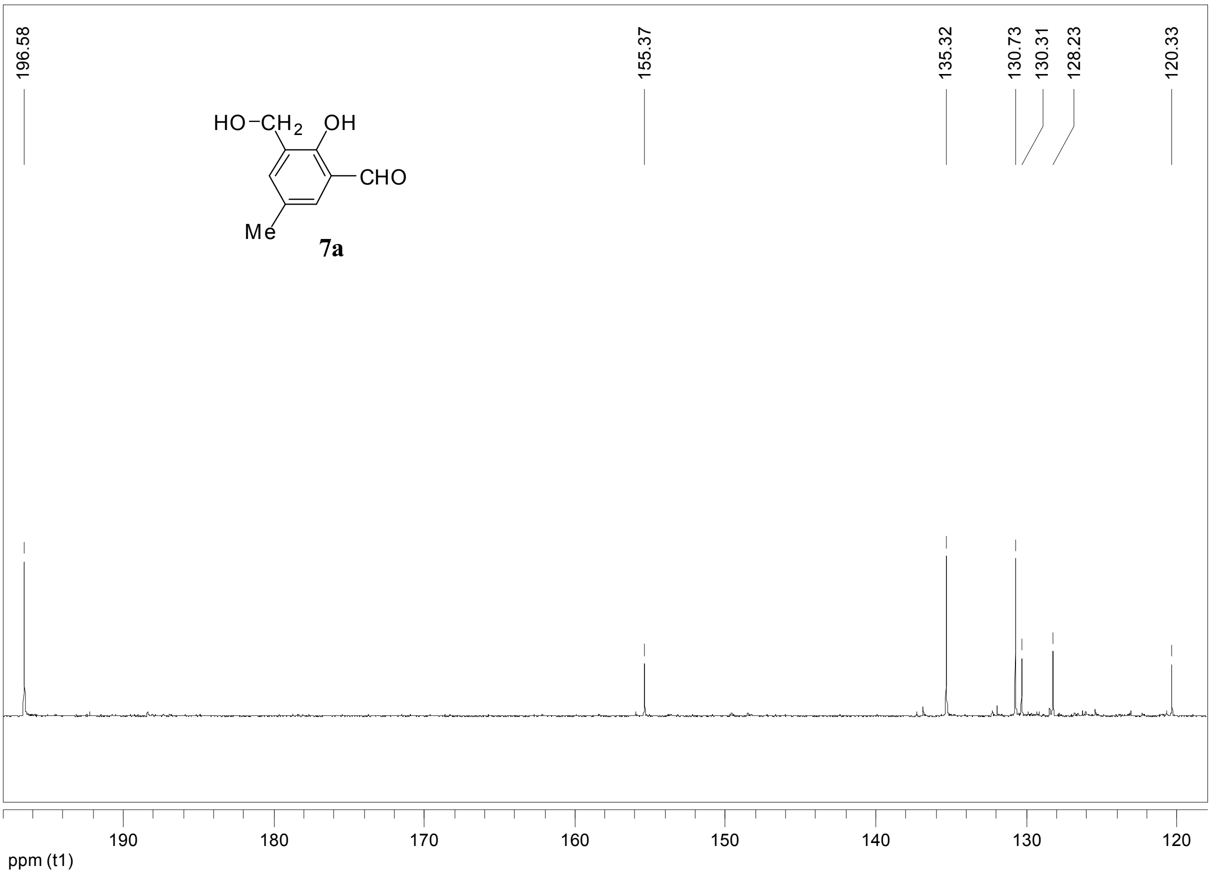

Figure S316. Expansion of ^{13}C-NMR (100 MHz, DMSO-d_6) spectrum of 5-methyl-salicylic aldehyde **7a**.

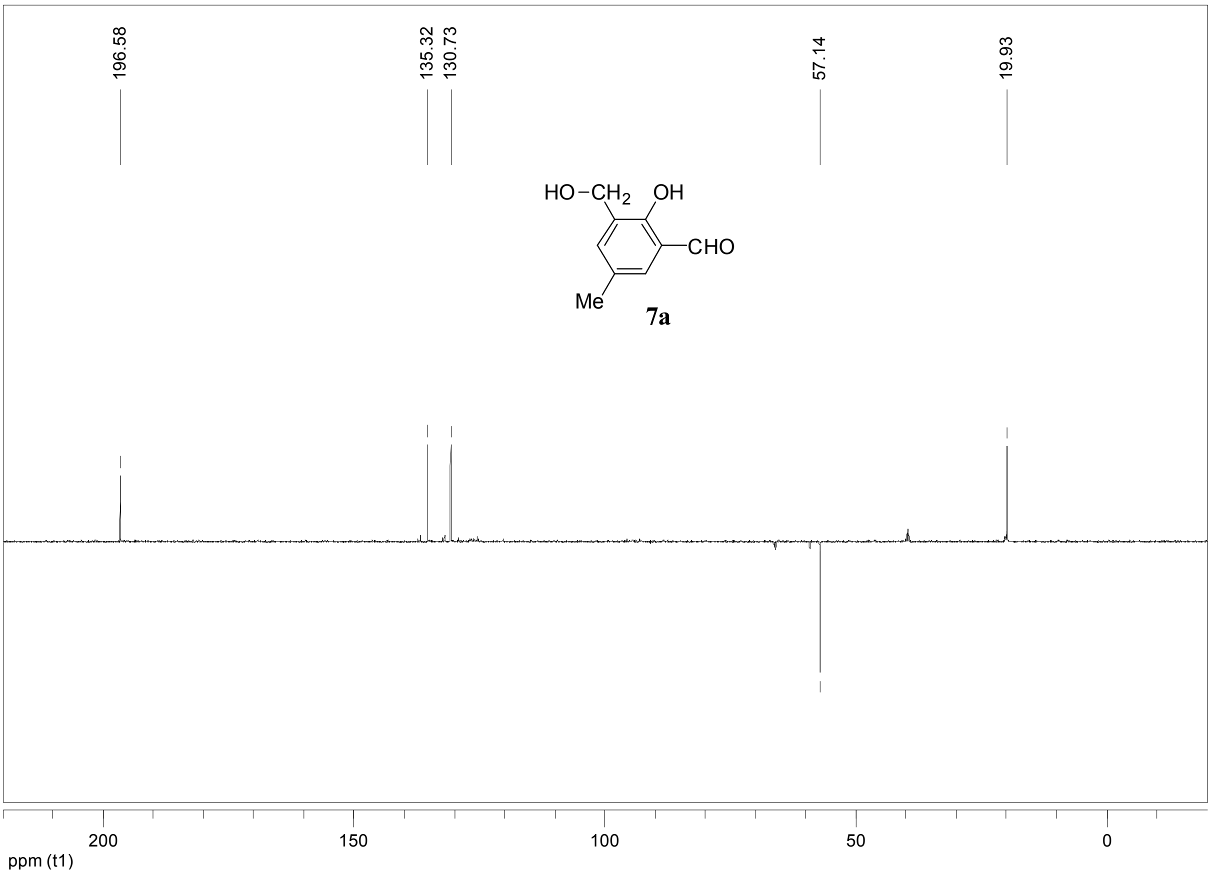

Figure S317. ^{13}C-NMR (100 MHz, DMSO-d_6) dept-135 experiment of 5-methyl-salicylic aldehyde **7a**.

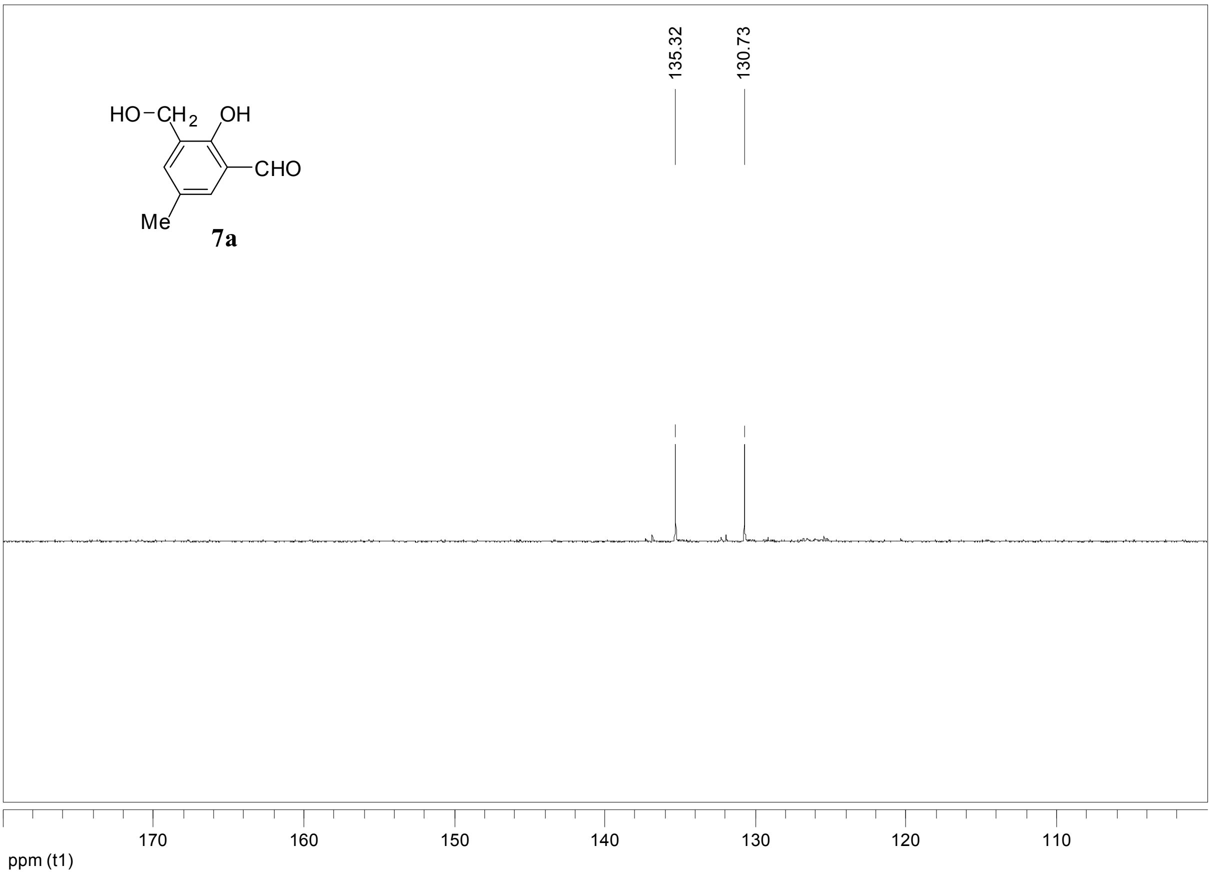

Figure S318. Expansion of ^{13}C-NMR (100 MHz, DMSO-d_6) dept-135 experiment of benzaldehyde **7a**.

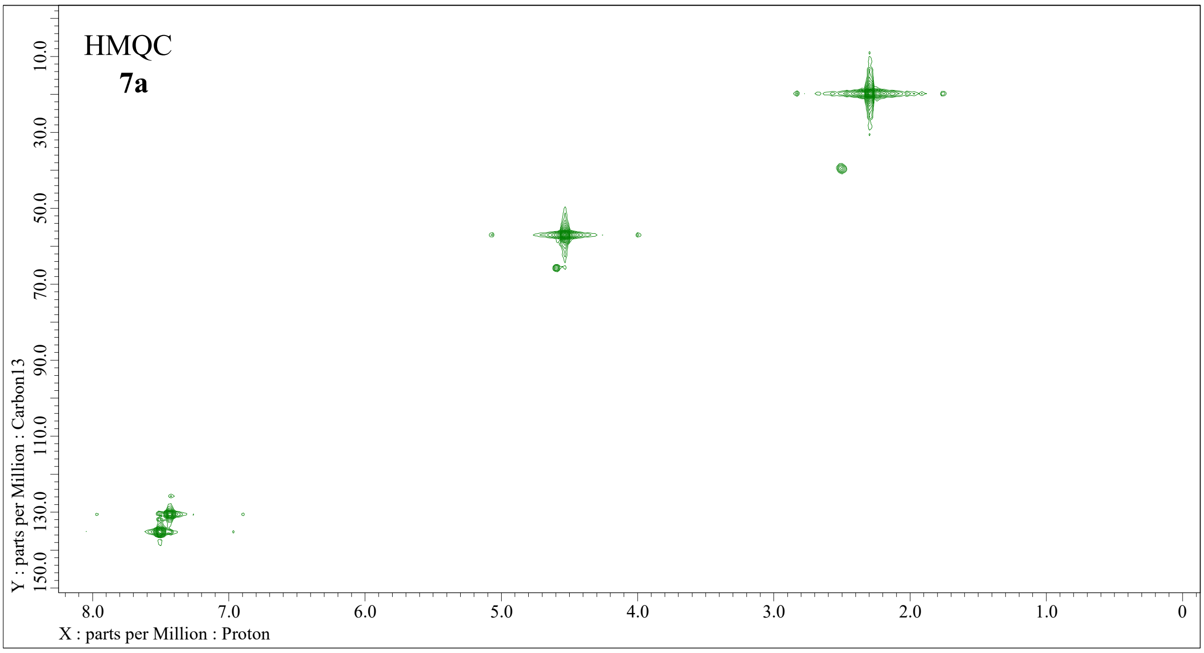

Figure S319. 2D-NMR (400 MHz, DMSO-d_6) HMQC experiment of 3-hydroxymethyl-5-methyl-salicylic aldehyde (**7a**).

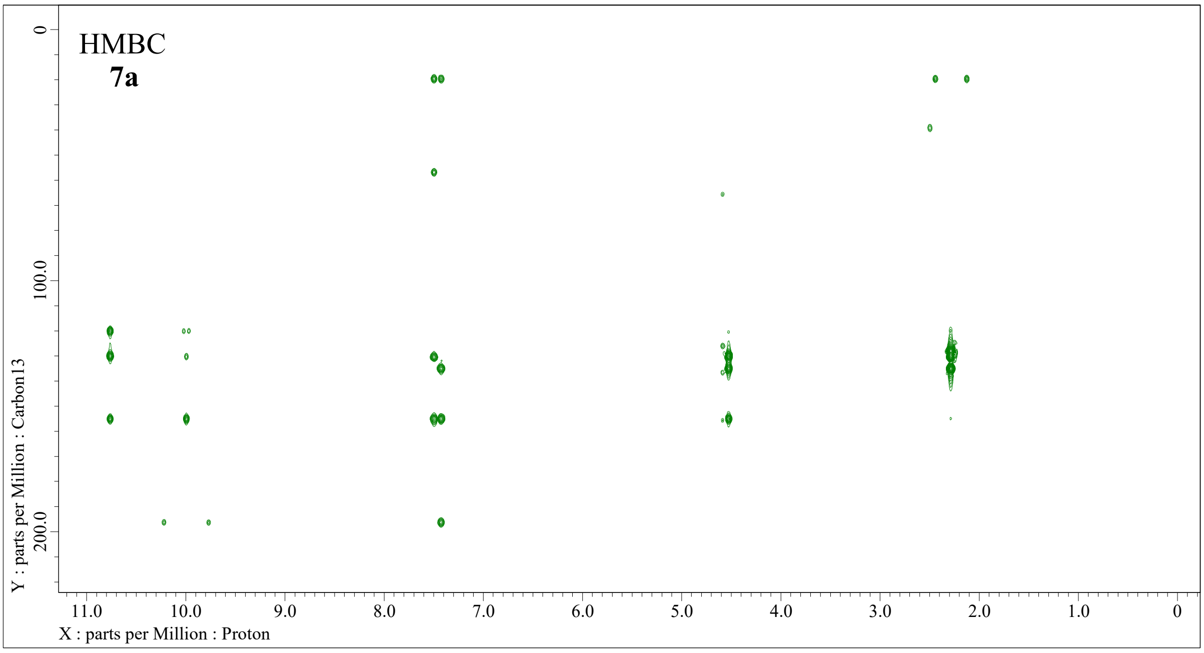

Figure S320. Expansion of 2D-NMR (400 MHz, DMSO-d_6) HMBC experiment of 3-hydroxymethyl-5-methyl-salicylic aldehyde (**7a**).

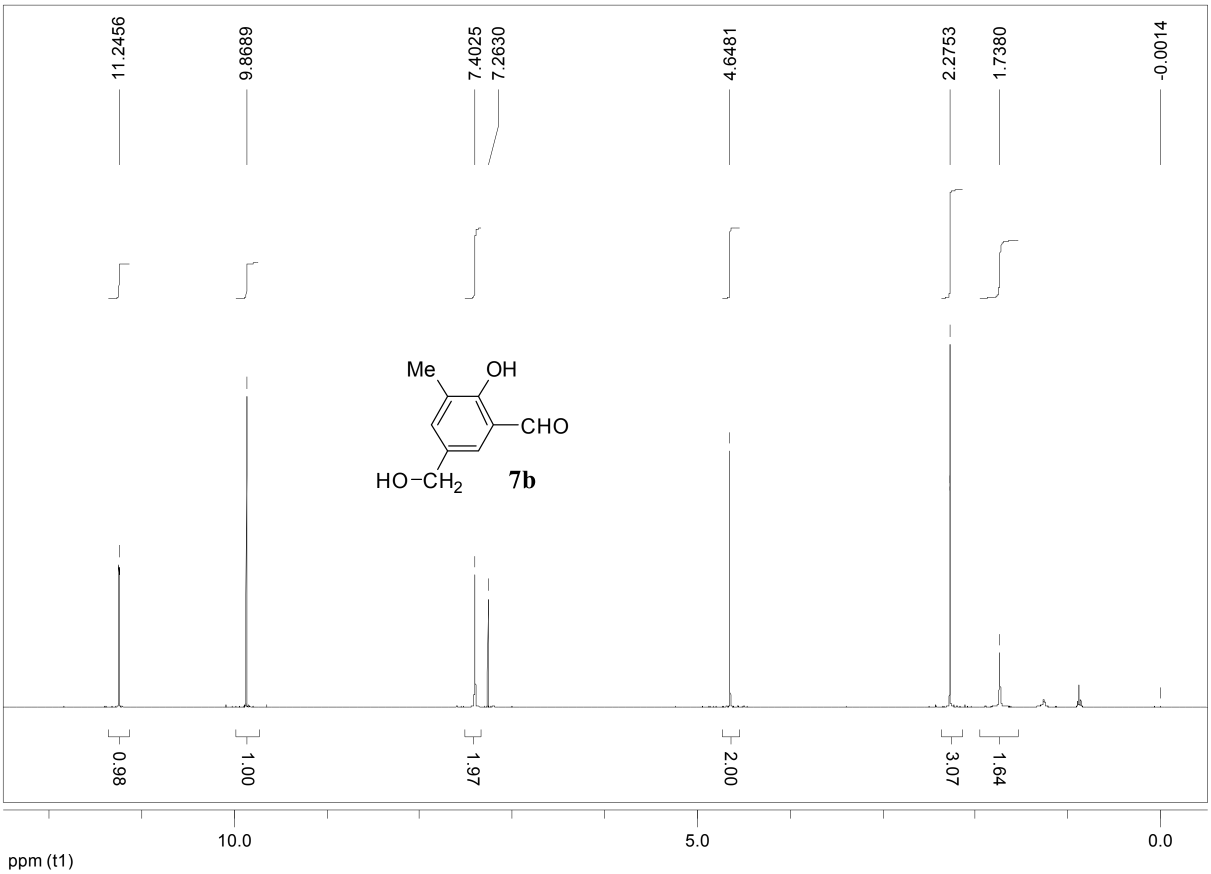

Figure S321. ^{1}H-NMR (400 MHz, $CDCl_3$) spectrum of hydroxymethyl-3-methyl-salicylic aldehyde **7b**.

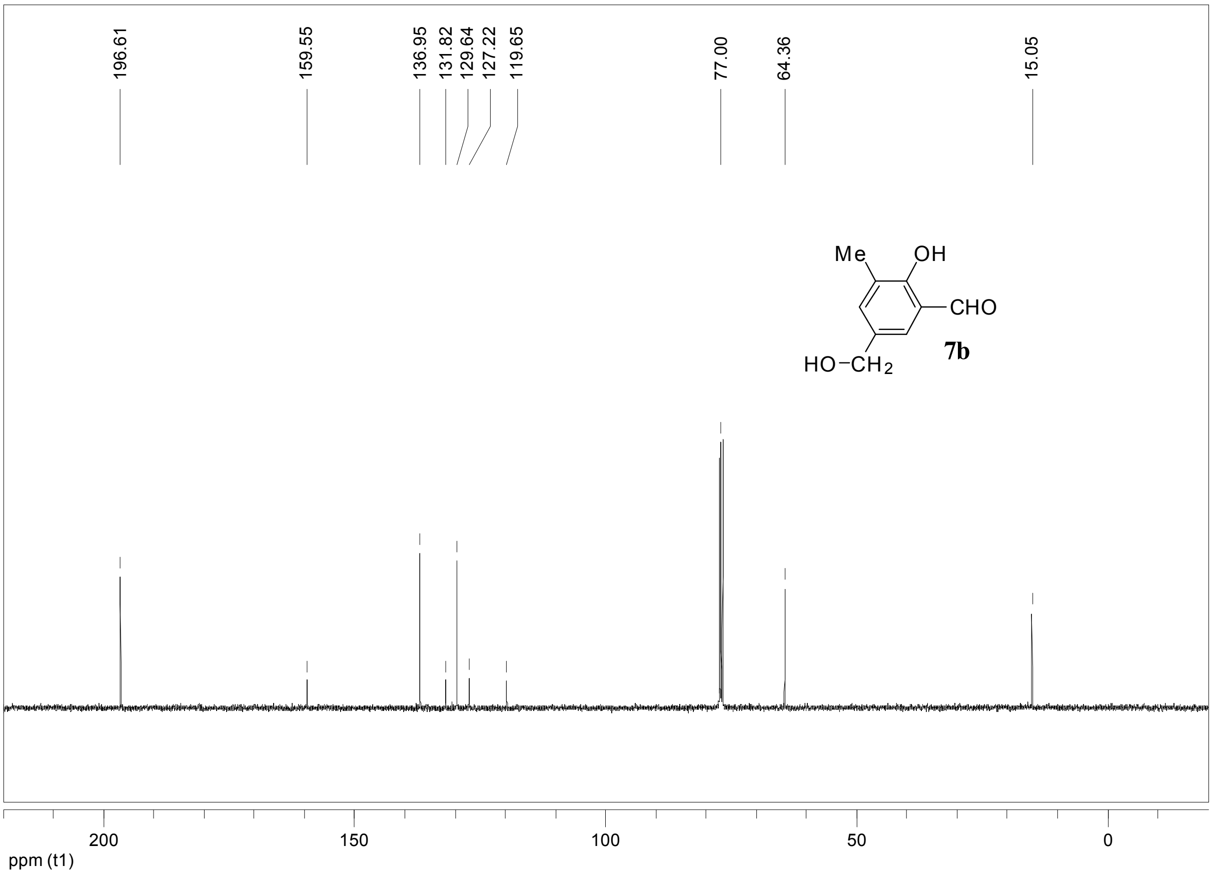

Figure S322. ^{13}C-NMR (100 MHz, $CDCl_3$) spectrum of hydroxymethyl-3-methyl-salicylic aldehyde **7b**.

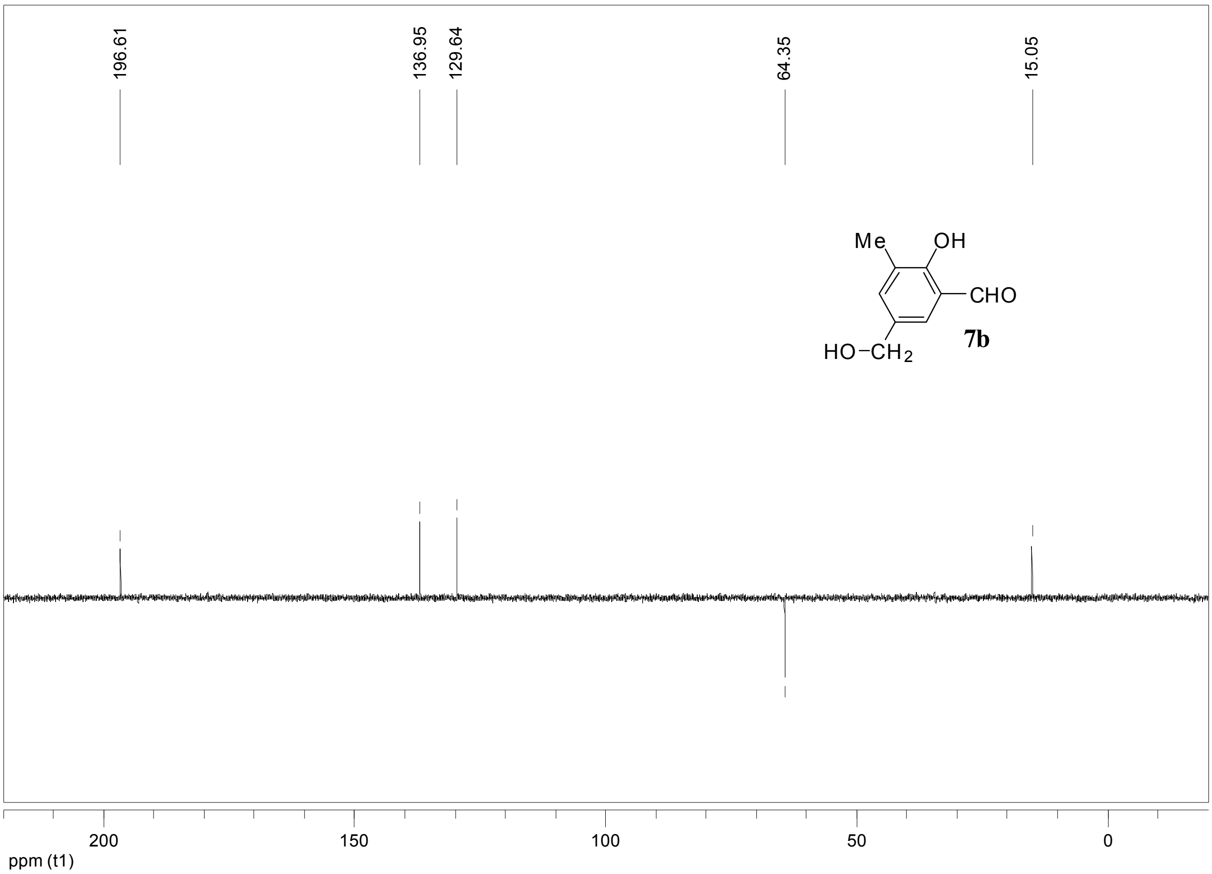

Figure S323. ^{13}C-NMR (100 MHz, $CDCl_3$) dept-135 experiment of 3-methyl-salicylic aldehyde **7b**.

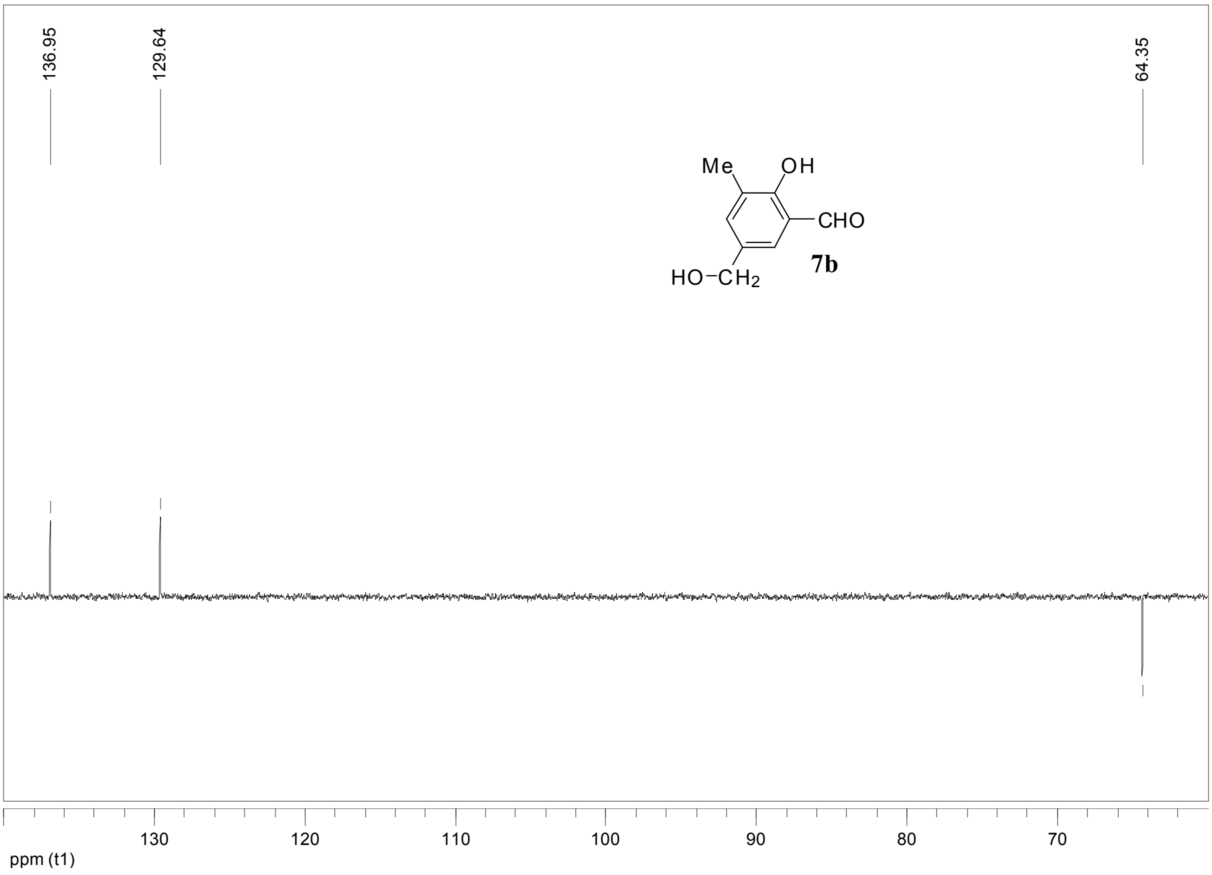

Figure S324. Expansion of ^{13}C-NMR (100 MHz, $CDCl_3$) dept-135 experiment of salicylic aldehyde **7b**.

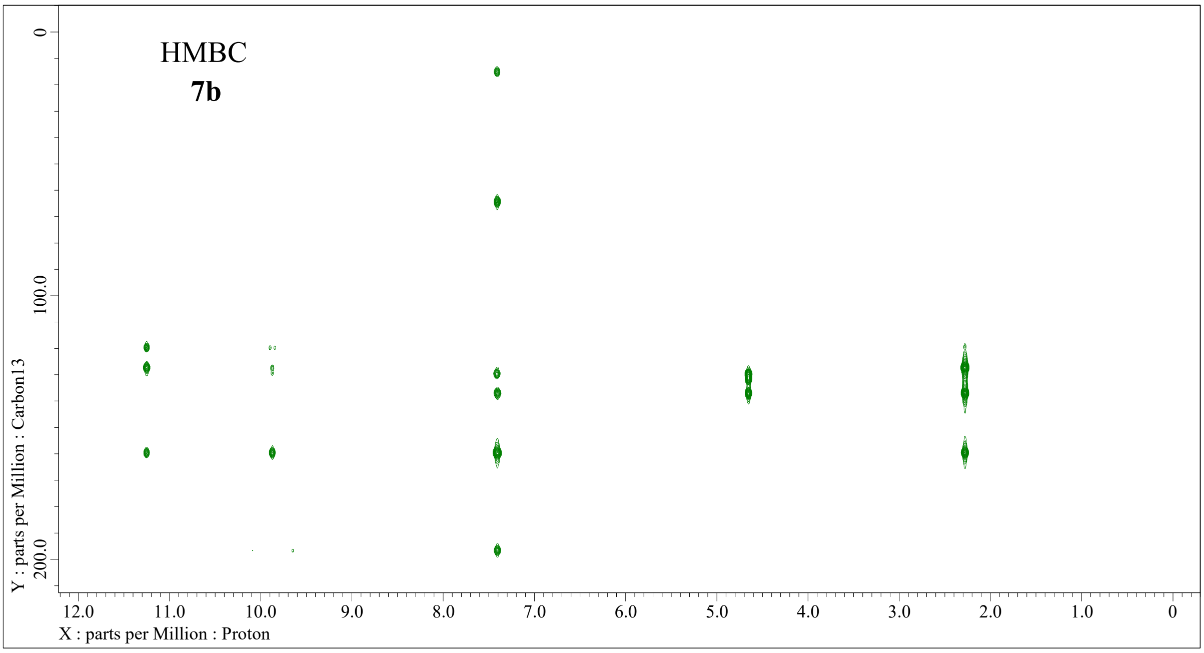

Figure S325. 2D-NMR (400 MHz, $CDCl_3$) HMBC experiment of 5-hydroxymethyl-3-methyl-salicylic aldehyde (**7b**).

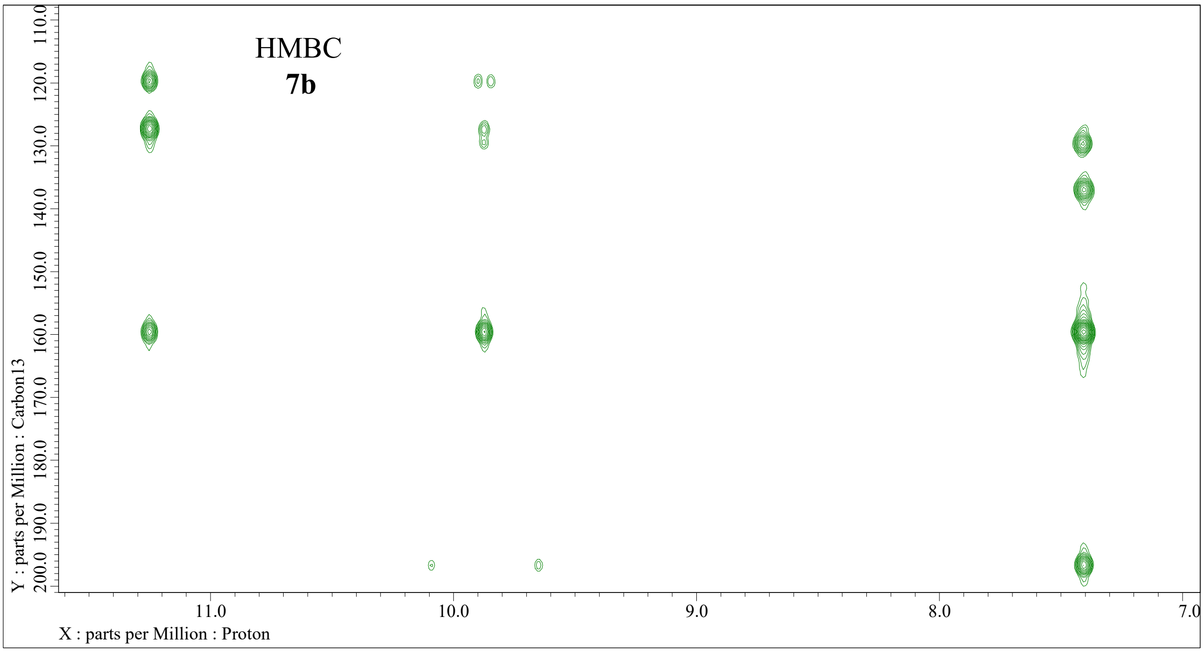

Figure S326. Expansion of 2D-NMR (400 MHz, $CDCl_3$) HMBC experiment 5-hydroxymethyl-3-methyl-salicylic aldehyde (**7b**).

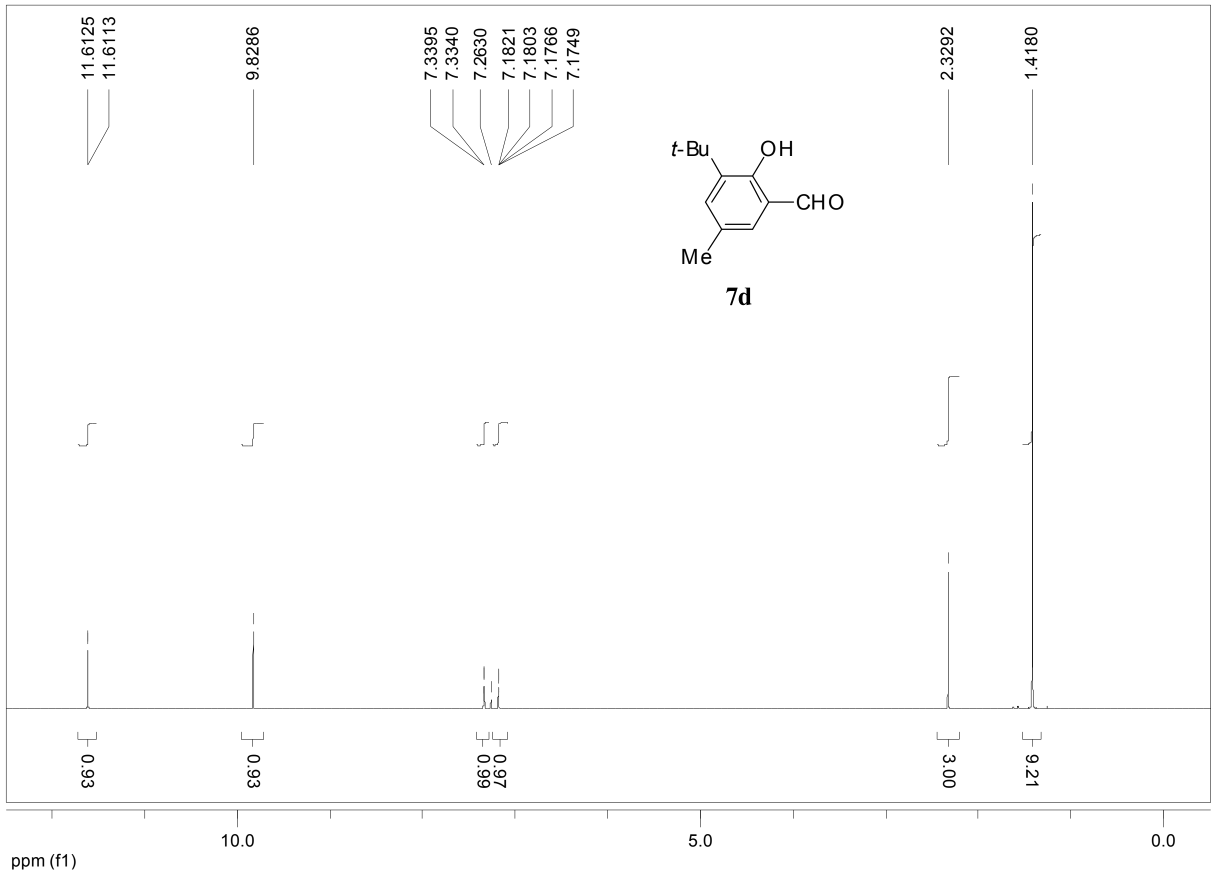

Figure S327. ^{1}H-NMR (400 MHz, $CDCl_3$) spectrum of 3-*tert*-butyl-5-methyl-salicylic aldehyde (**7d**).

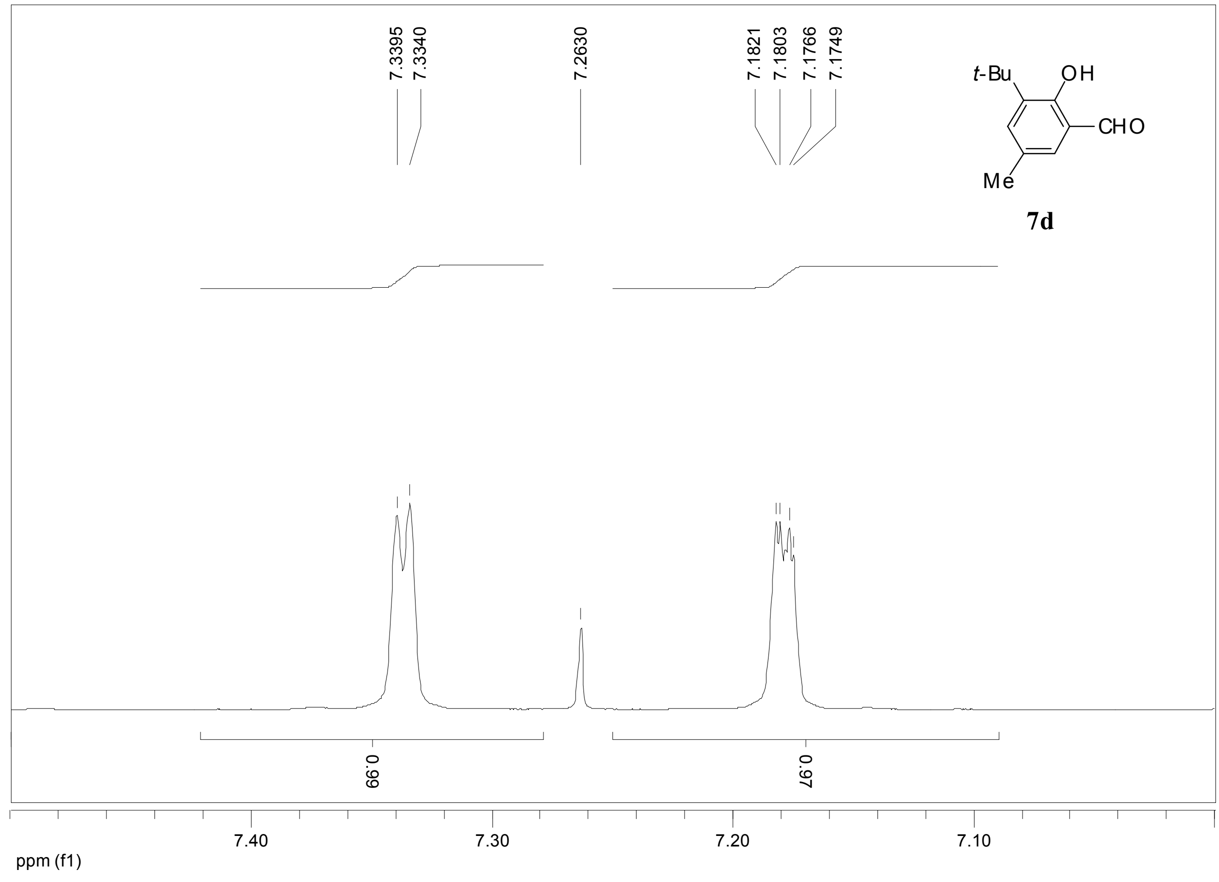

Figure S328. Expansion of ^{1}H-NMR (400 MHz, $CDCl_3$) spectrum of 5-methyl-salicylic aldehyde **7d**.

Molecules **2020**, *25*, 1255; doi:10.3390/molecules25051255 www.mdpi.com/journal/molecules

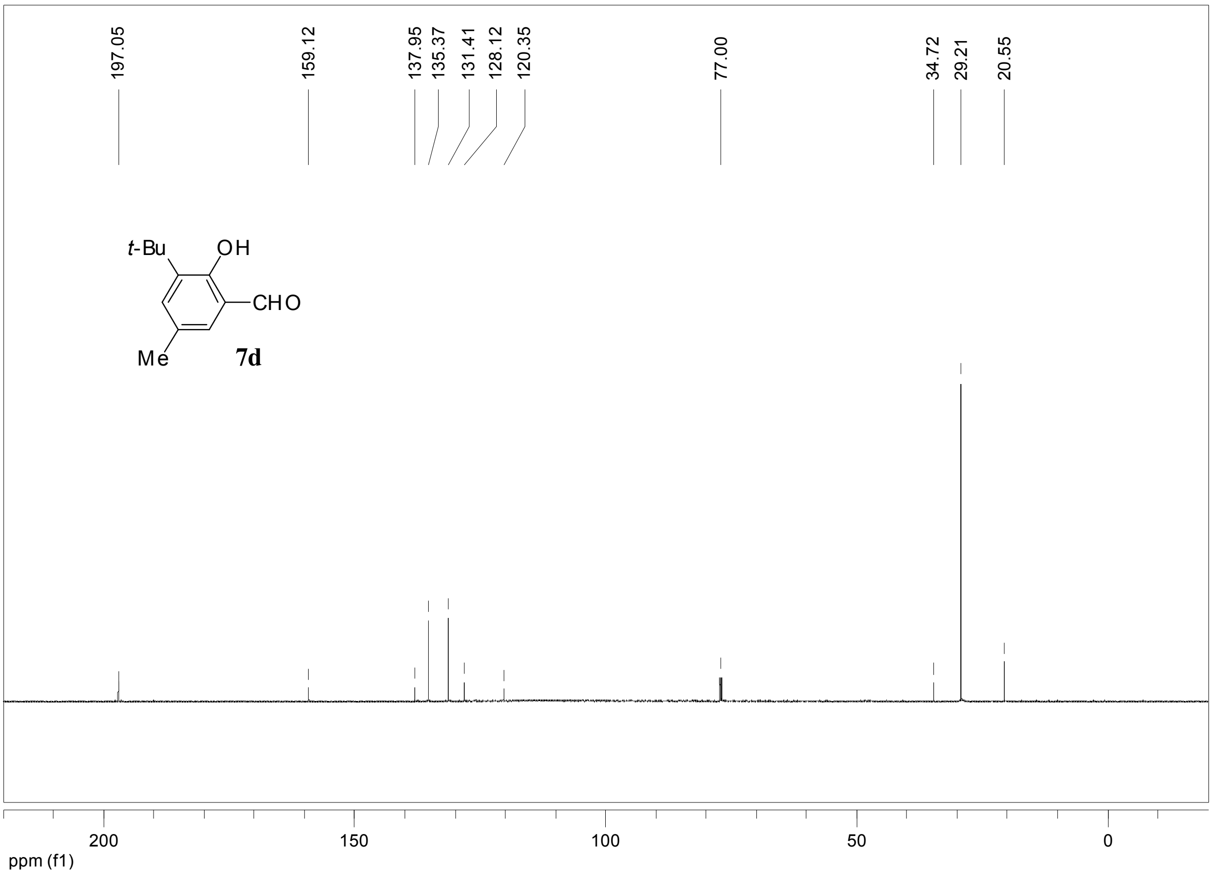

Figure S329. ^{13}C-NMR (150 MHz, $CDCl_3$) spectrum of 3-*tert*-butyl-5-methyl-salicylic aldehyde (**7d**).

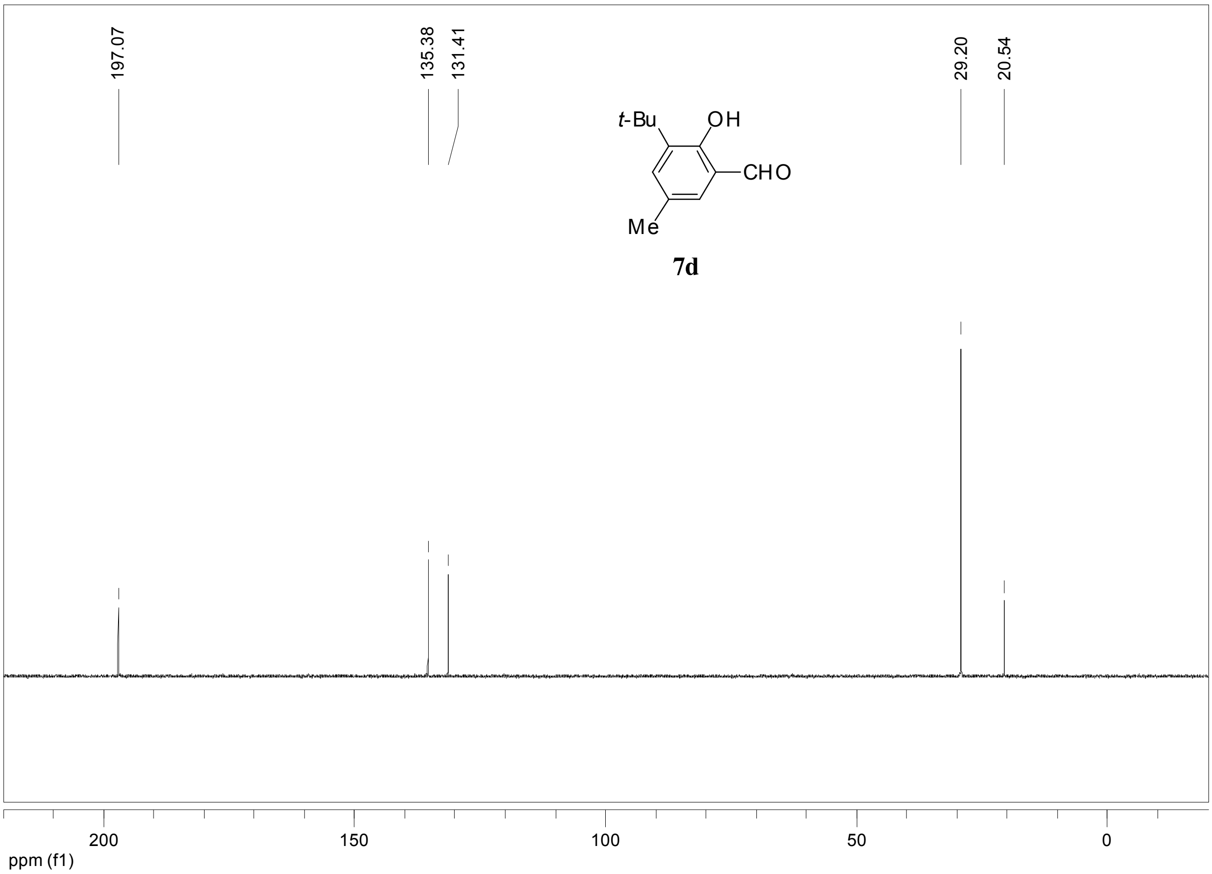

Figure S330. ^{13}C-NMR (100 MHz, $CDCl_3$) dept-135 experiment of 3-*tert*-butyl-salicylic aldehyde **7d**.

Molecules **2020**, *25*, 1255; doi:10.3390/molecules25051255 www.mdpi.com/journal/molecules

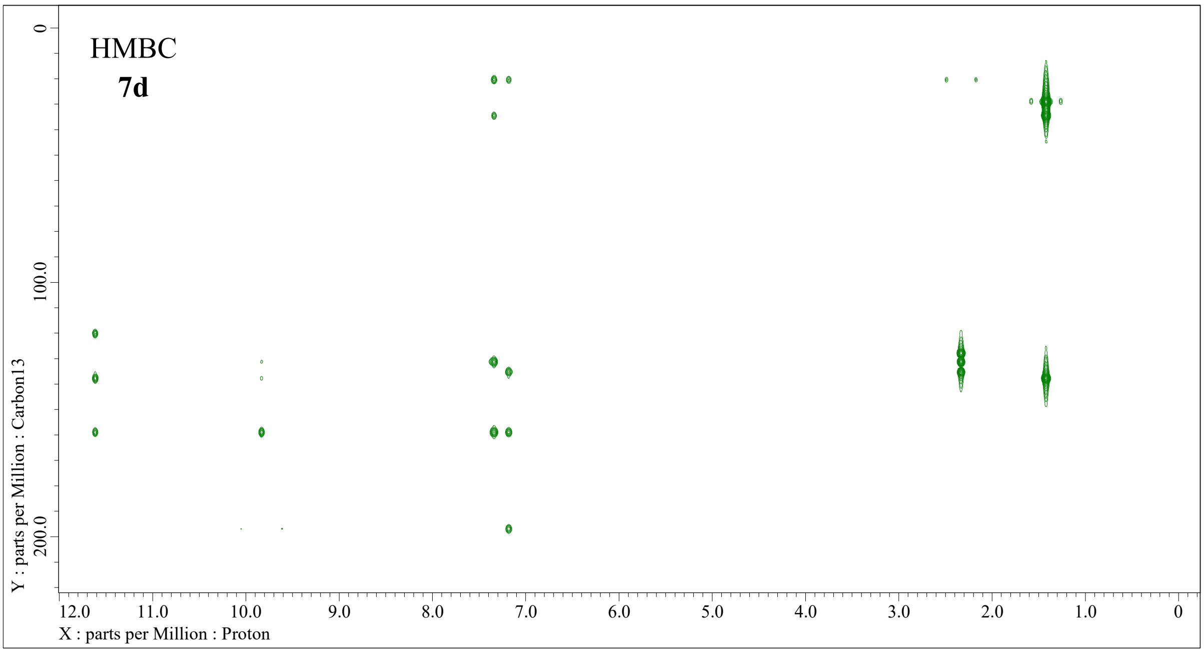

Figure S331. 2D-NMR (400 MHz, $CDCl_3$) HMBC experiment of 3-*tert*-butyl-5-salicylic aldehyde (**7d**).

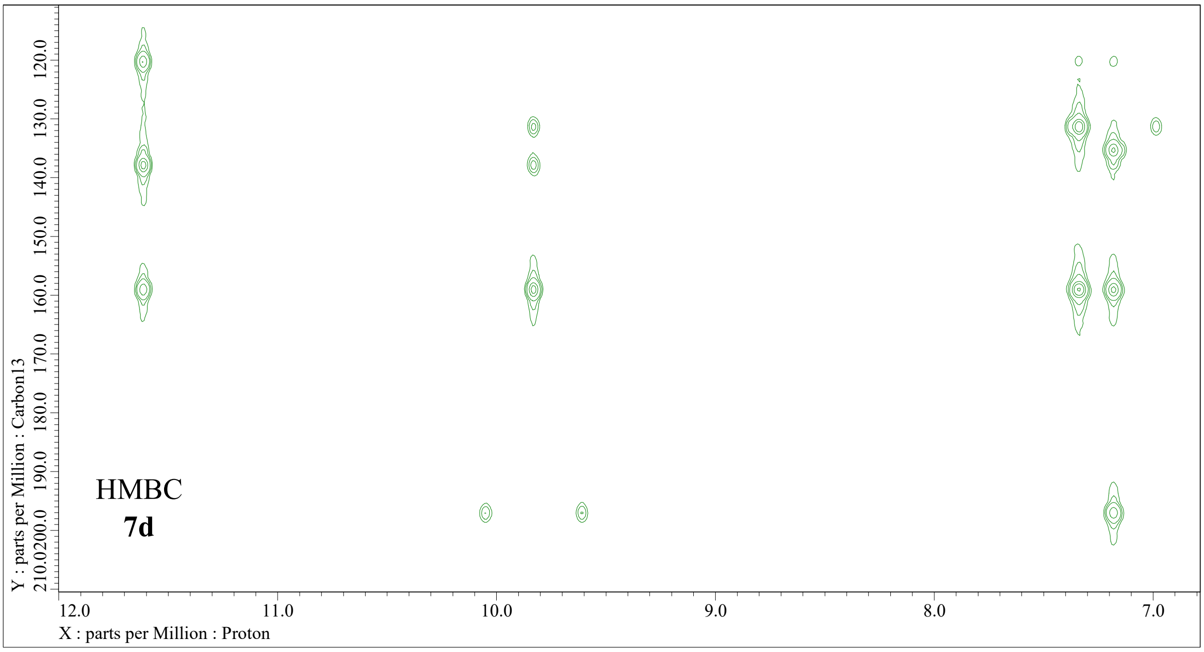

Figure S332. Expansion of 2D-NMR (400 MHz, $CDCl_3$) HMBC experiment 3-*tert*-butyl-5-salicylic aldehyde (**7d**).

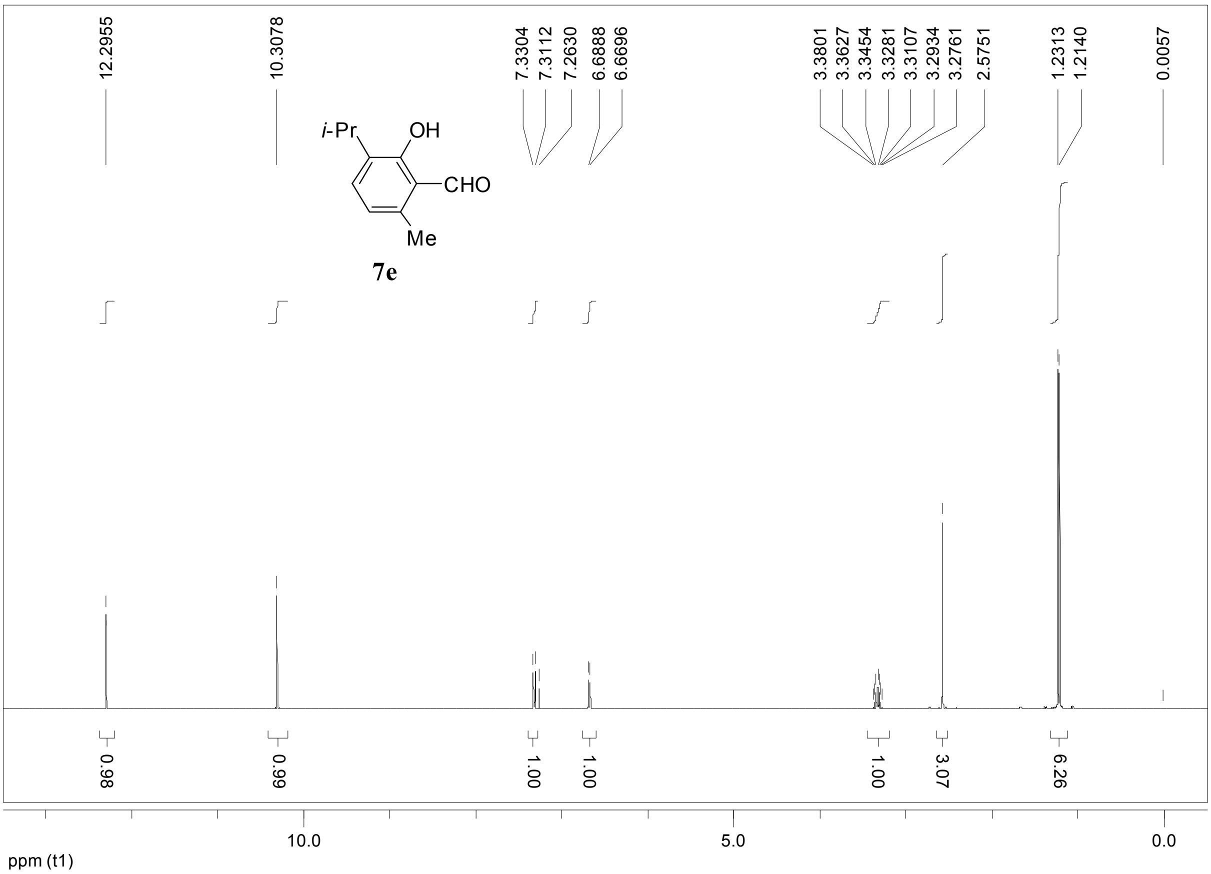

Figure S333. ^{1}H-NMR (400 MHz, $CDCl_3$) spectrum of *ortho*-formyl-thymol (**7e**).

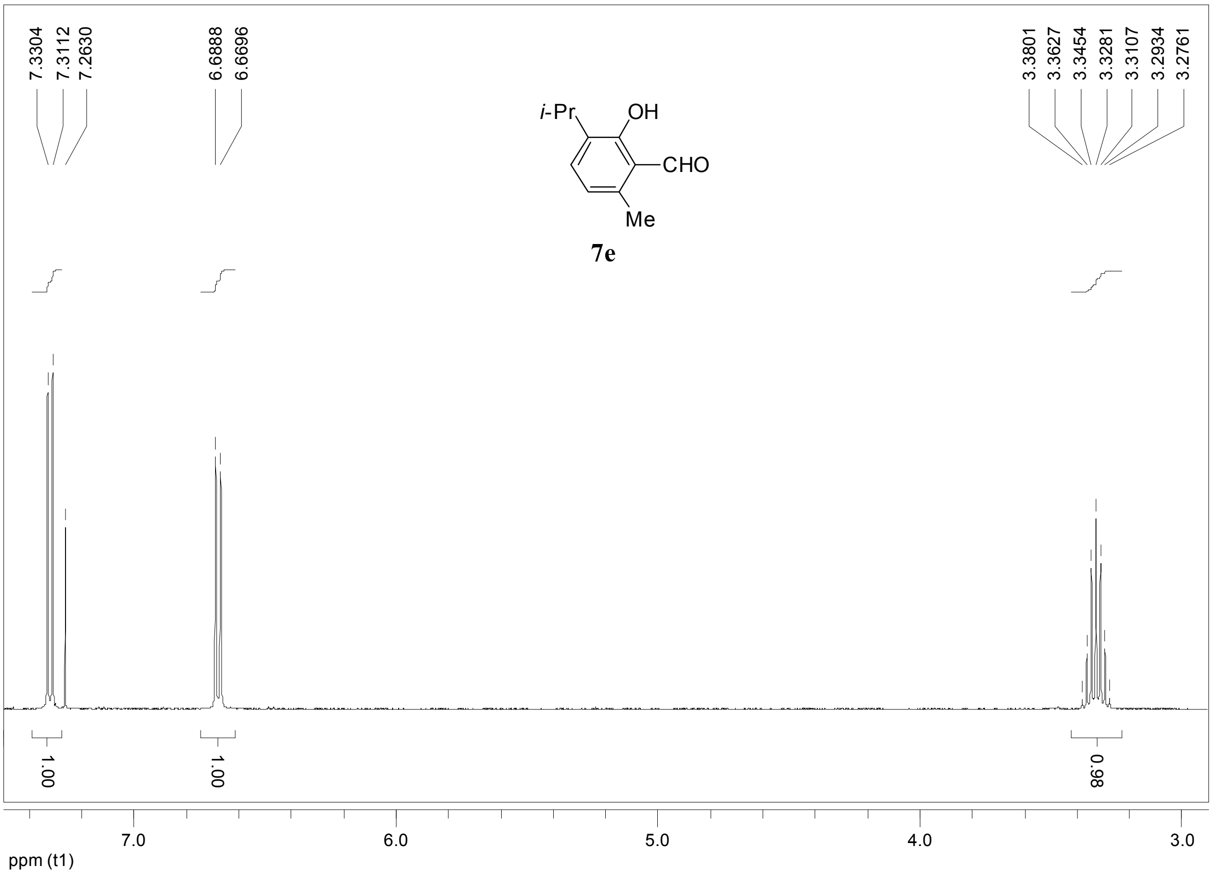

Figure S334. Expansion of ^{1}H-NMR (400 MHz, $CDCl_3$) spectrum of *ortho*-formyl-thymol (**7e**).

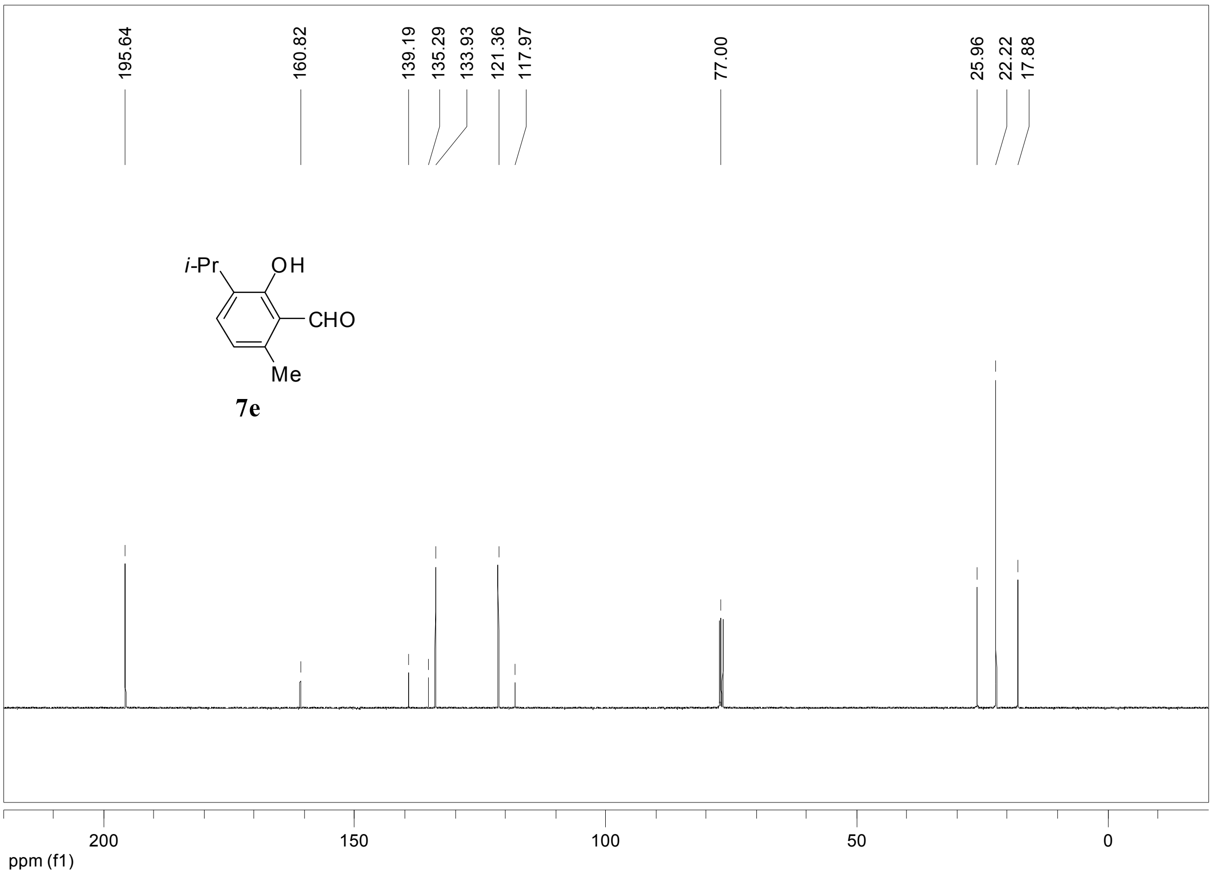

Figure S335. ^{13}C-NMR (100 MHz, $CDCl_3$) spectrum of 6-methyl-3-(propan-2-y)salicylic aldehyde (**7e**).

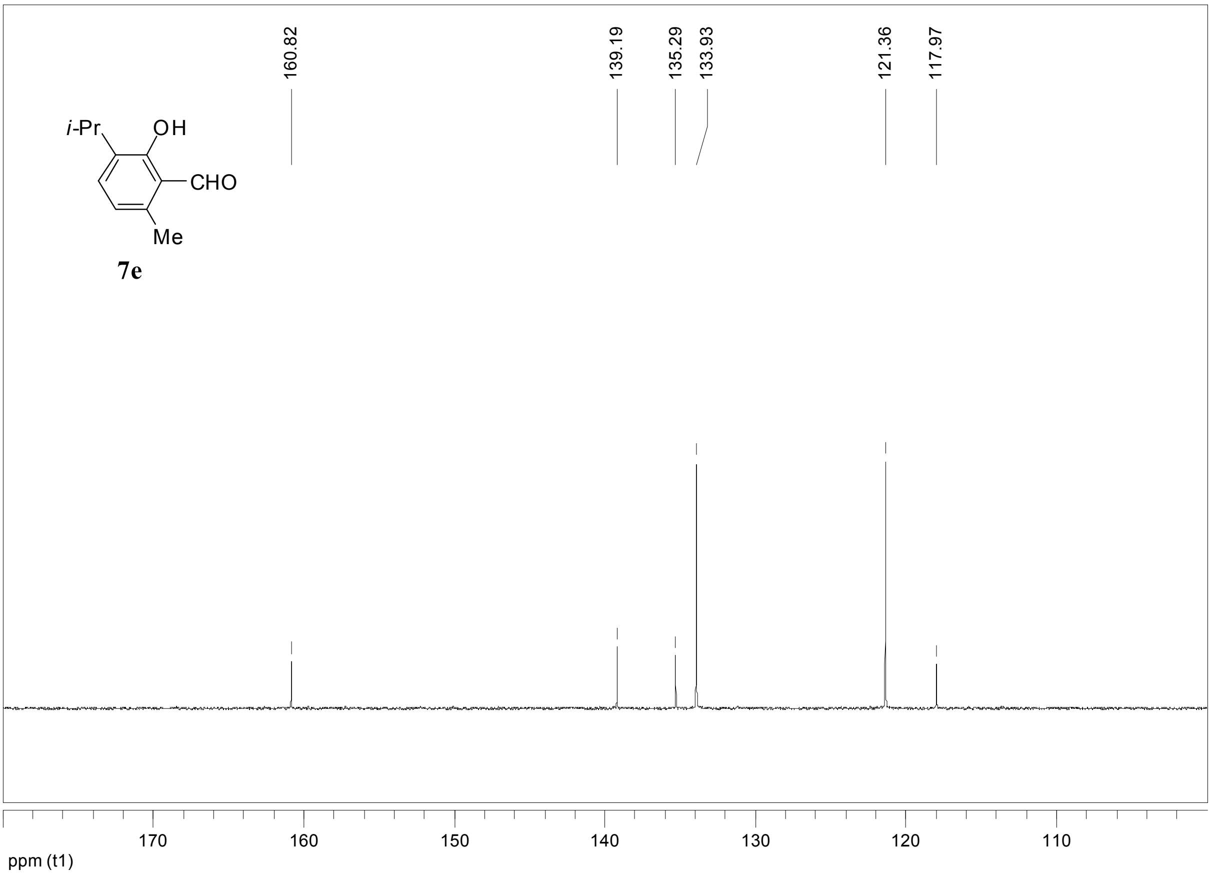

Figure S336. Expansion of ^{13}C-NMR (100 MHz, $CDCl_3$) spectrum of *ortho*-formyl-thymol (**7e**).

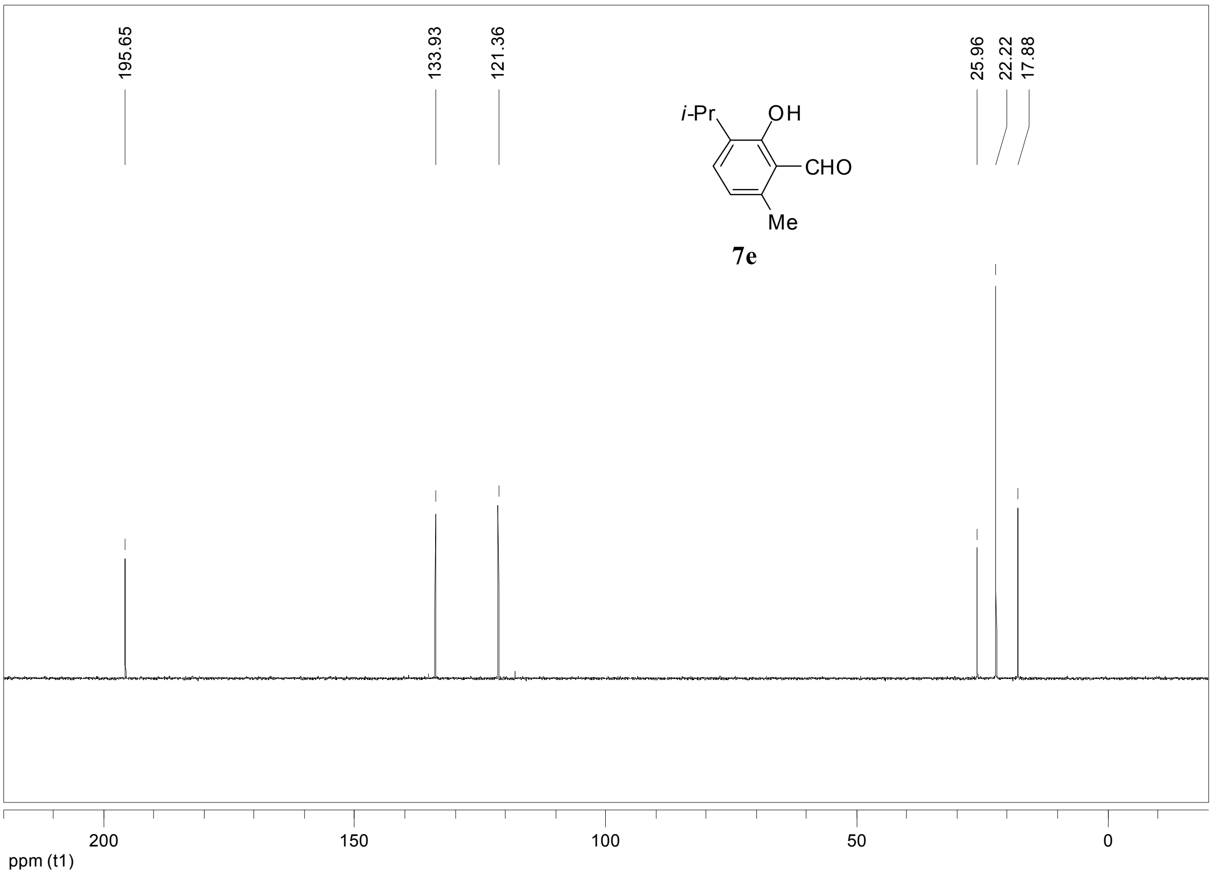

Figure S337. ^{13}C-NMR (100 MHz, $CDCl_3$) dept 135 experiment of *ortho*-formyl-thymol (**7e**).

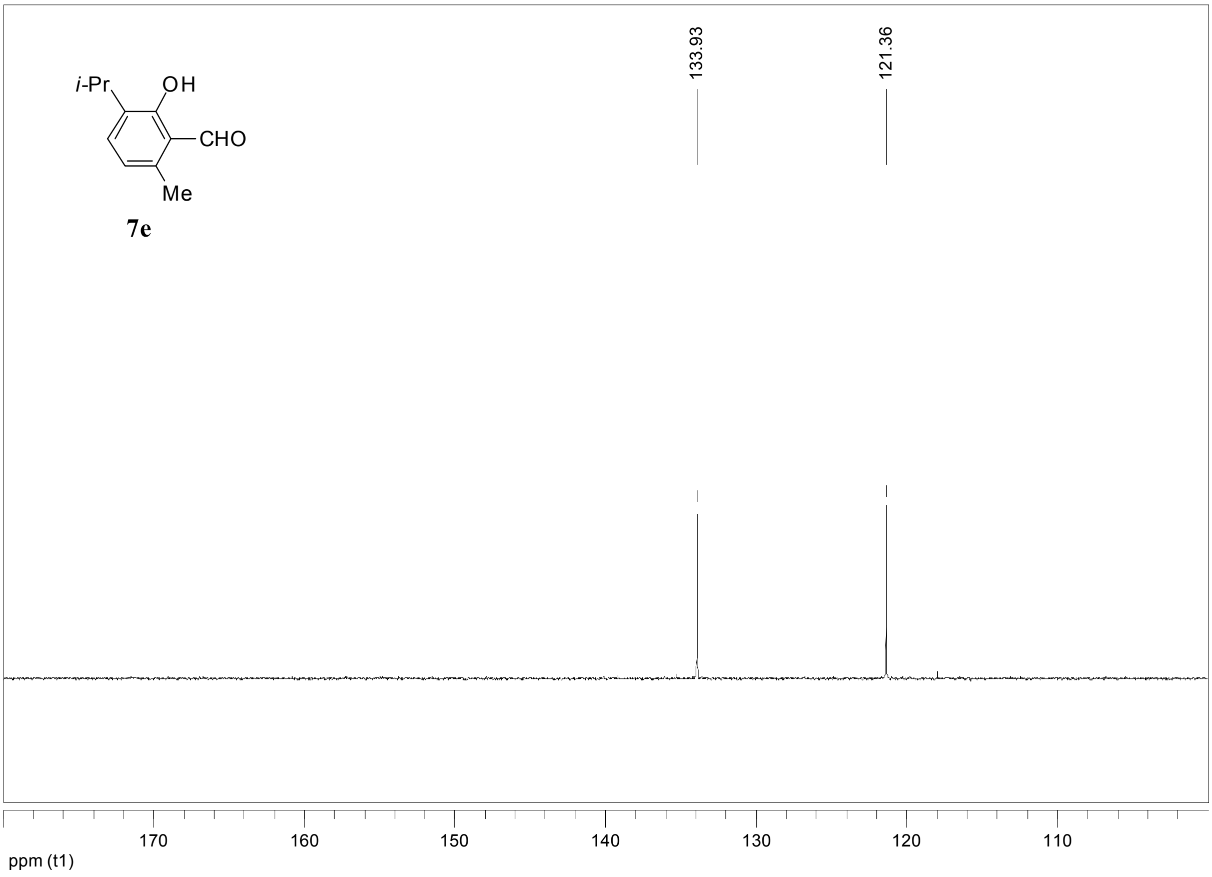

Figure S338. Expansion of ^{13}C-NMR (100 MHz, $CDCl_3$) dept 135 experiment of *o*-formyl-thymol (**7e**).

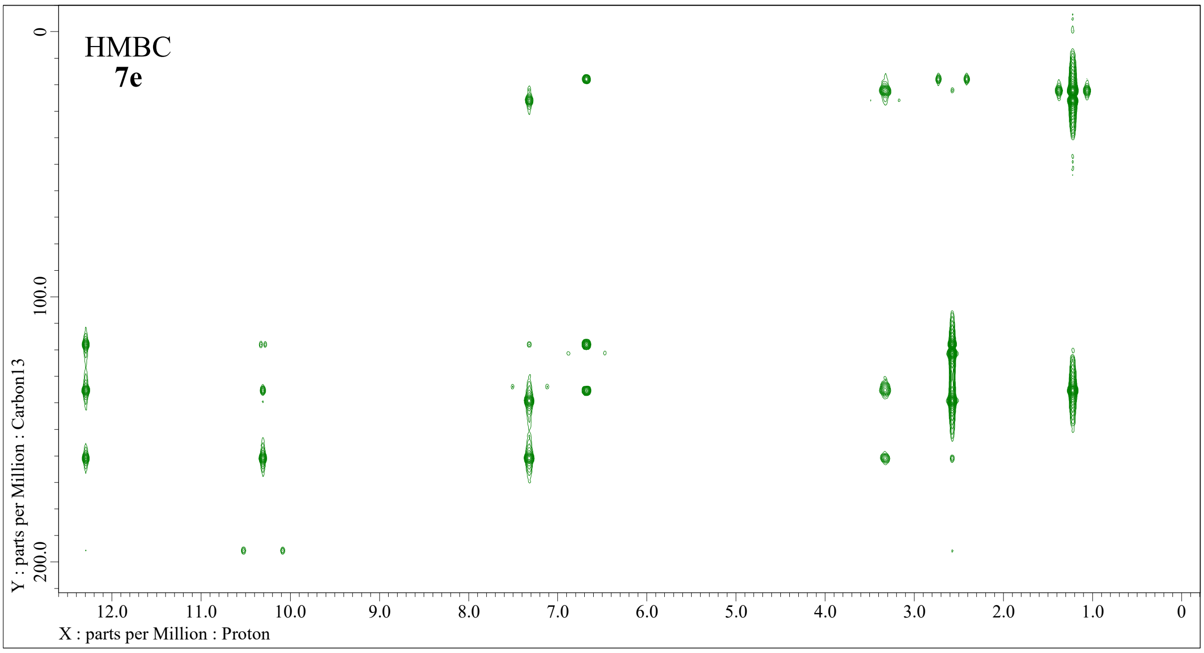

Figure S339. 2D-NMR (400 MHz, $CDCl_3$) HMBC experiment of 6-methyl-3-(propan-2-y)salicylic aldehyde (**7e**).

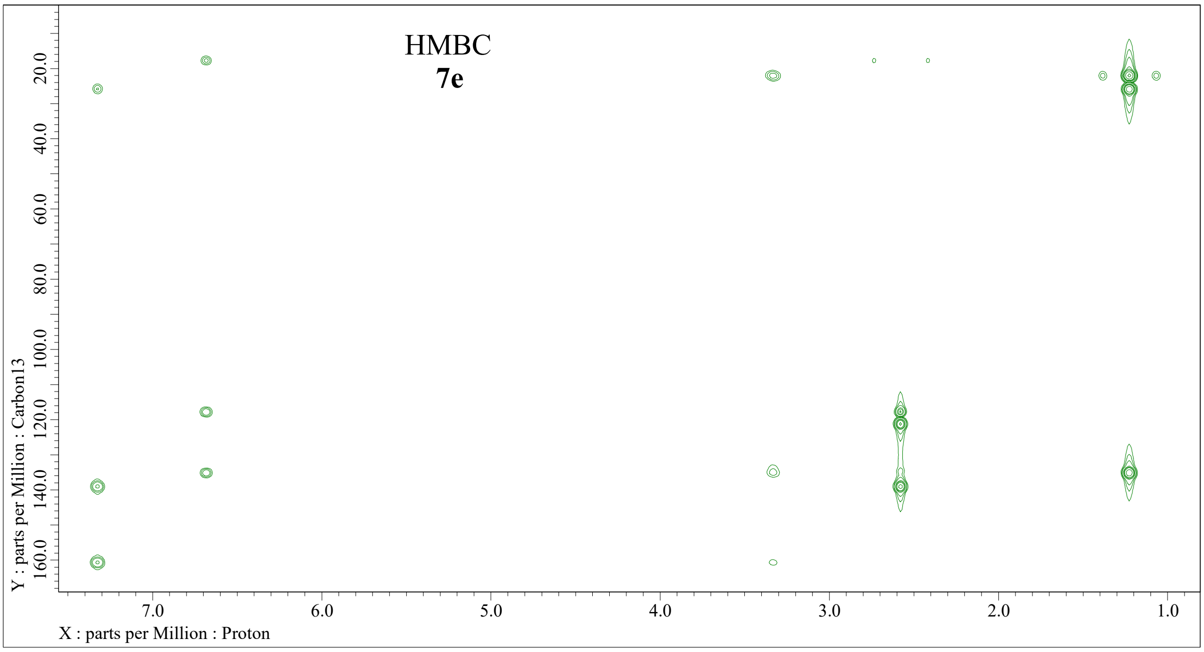

Figure S340. Expansion of 2D-NMR (400 MHz, $CDCl_3$) HMBC experiment of 6-methyl-3-(propan-2-y)salicylic aldehyde (**7e**).

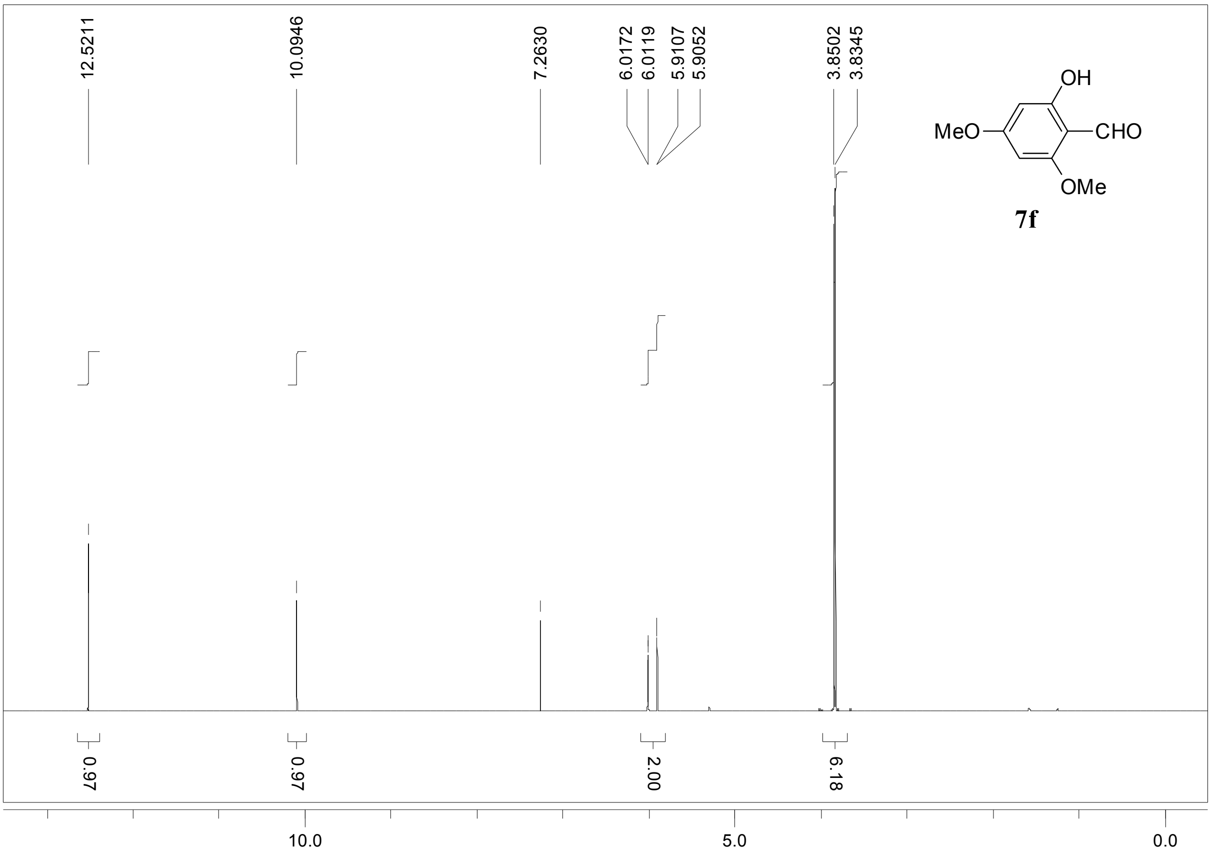

Figure S341. ^{1}H-NMR (400 MHz, $CDCl_3$) spectrum of 4,6-dimethoxy-salicylic aldehyde (**7f**).

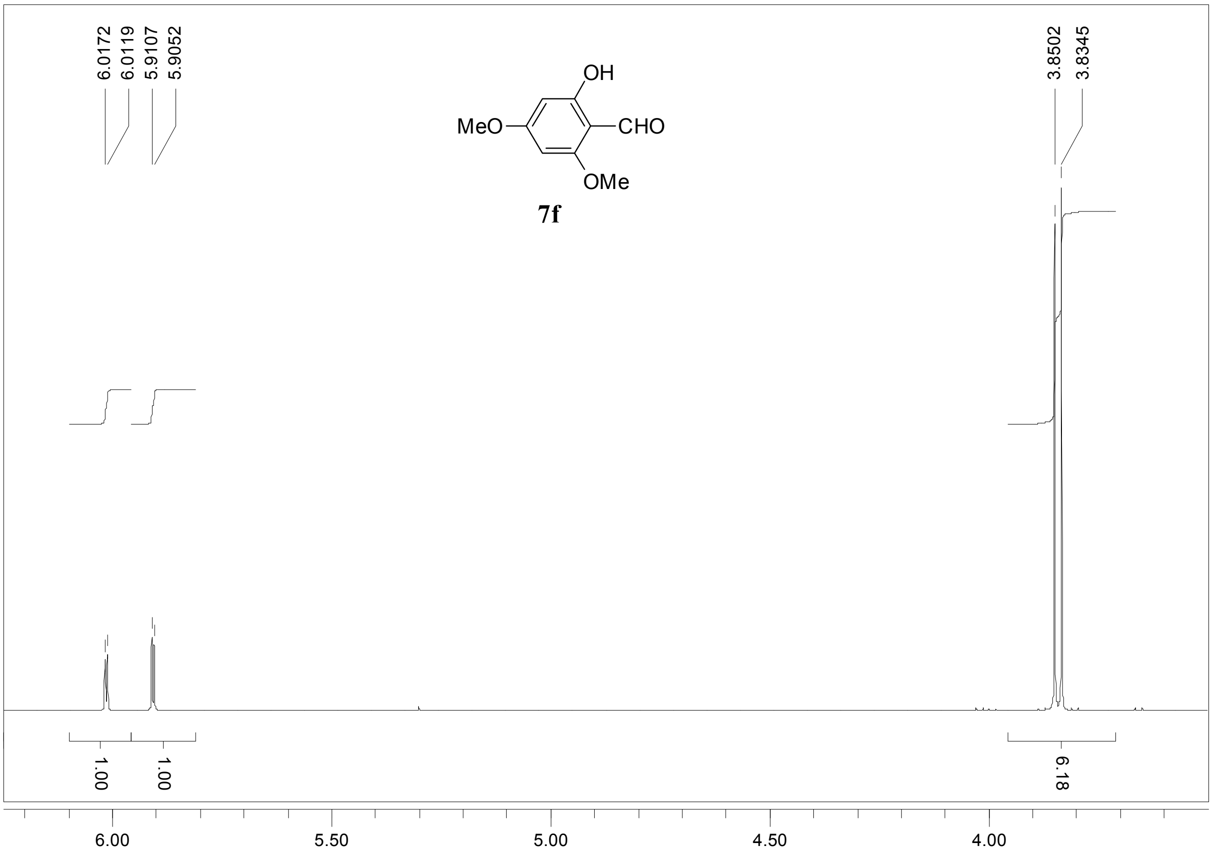

Figure S342. Expansion of ^{1}H-NMR (400 MHz, $CDCl_3$) spectrum of dimethoxy-salicylic aldehyde **7f**.

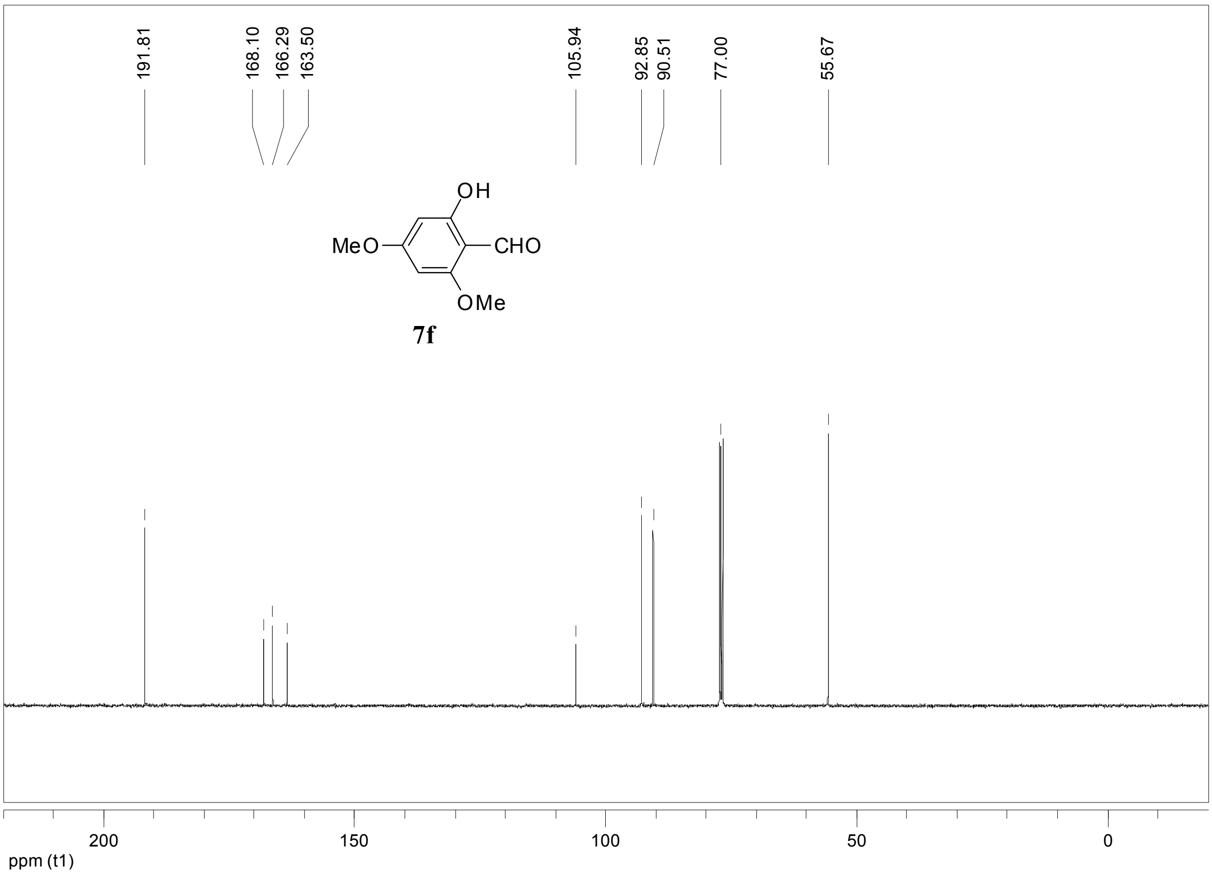

Figure S343. ^{13}C-NMR (100 MHz, $CDCl_3$) spectrum of 4,6-dimethoxy-salicylic aldehyde (**7f**).

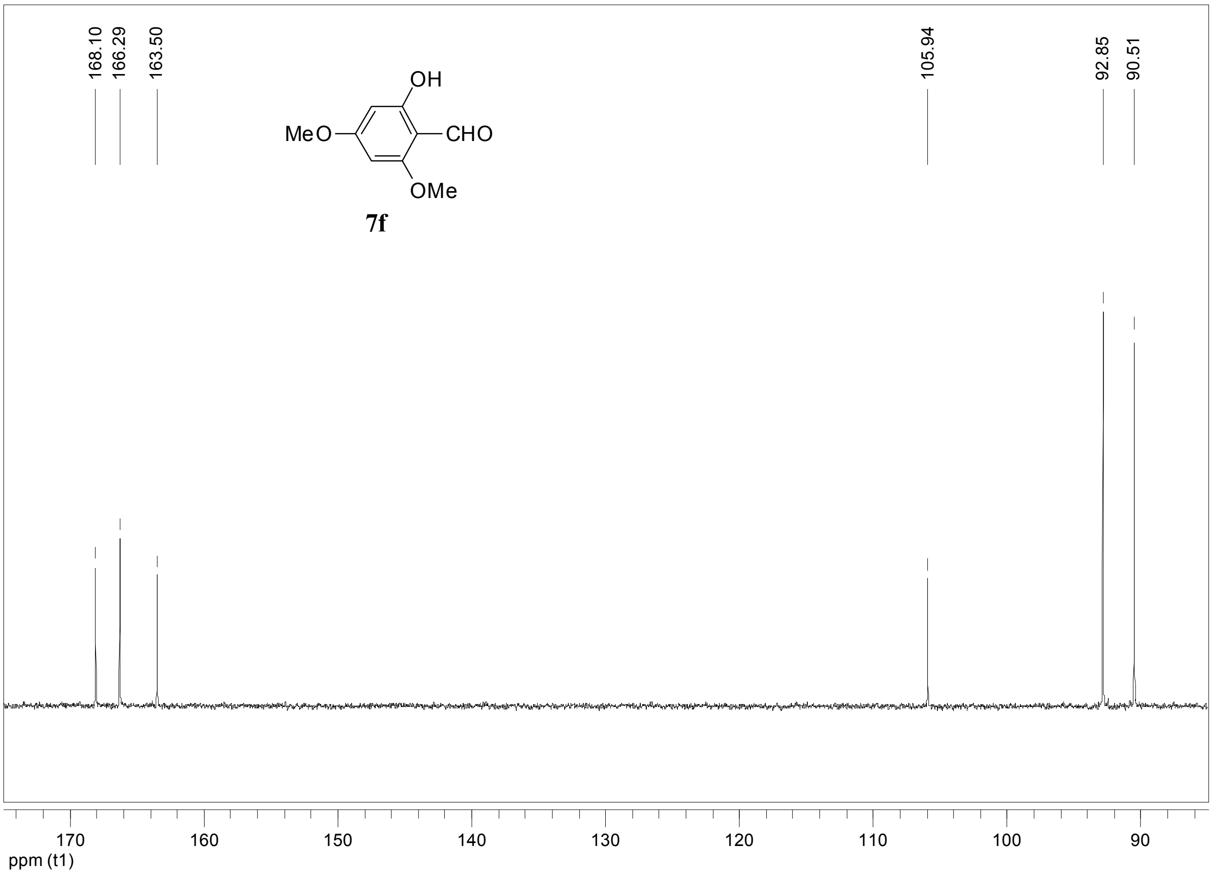

Figure S344. Expansion of ^{13}C-NMR (100 MHz, $CDCl_3$) spectrum of dimethoxy-salicylic aldehyde **7f**.

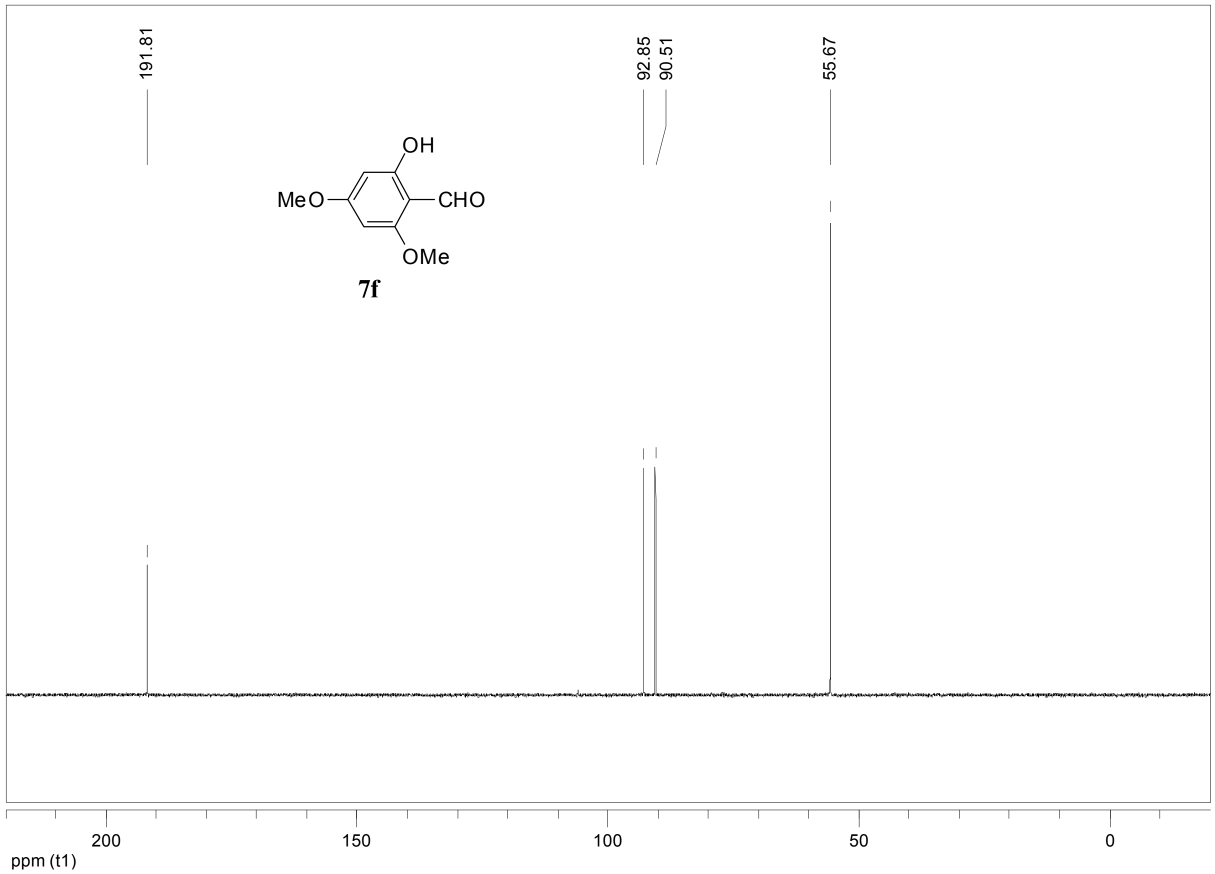

Figure S345. ^{13}C-NMR (100 MHz, $CDCl_3$) dept 135 experiment of dimethoxy-salicylic aldehyde **7f**.

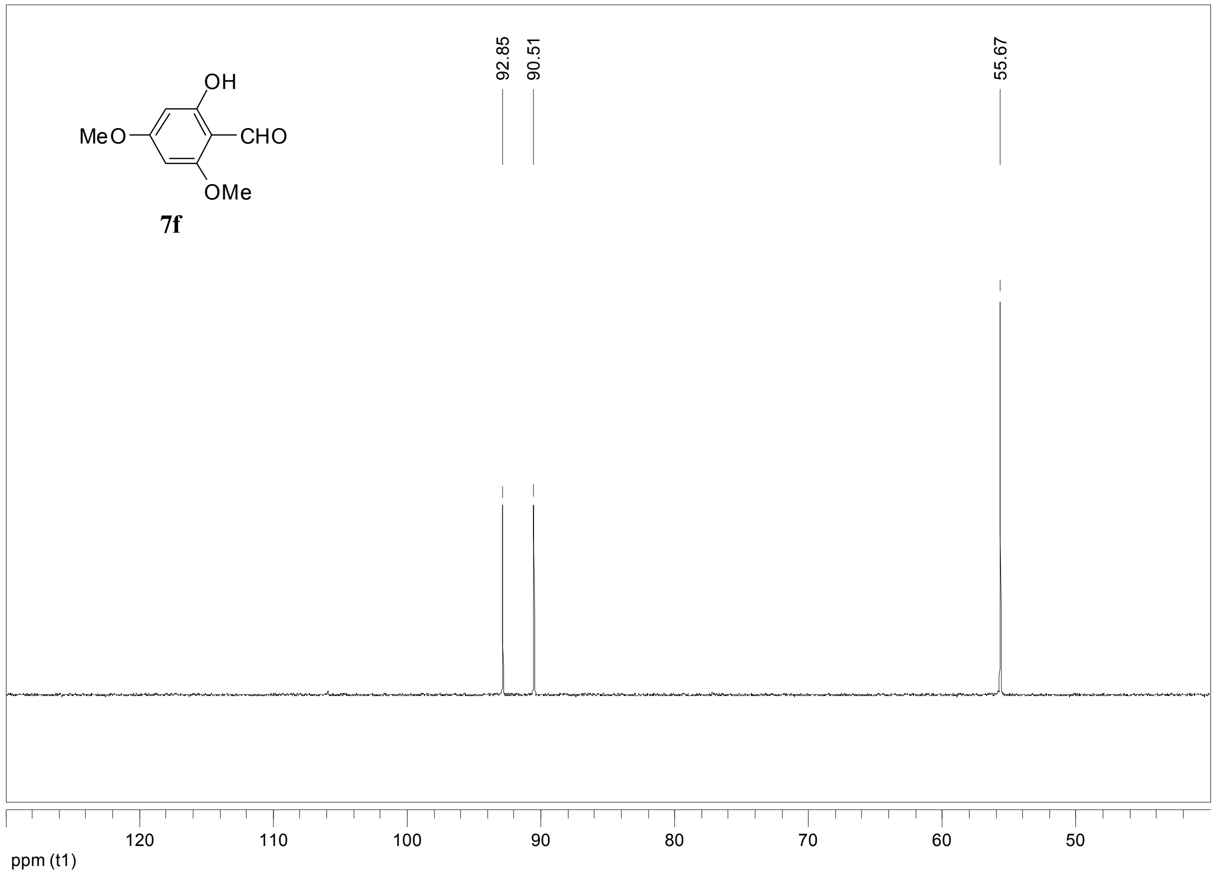

Figure S346. Expansion of ^{13}C-NMR (100 MHz, $CDCl_3$) dept 135 experiment of salicylic aldehyde **7f**.

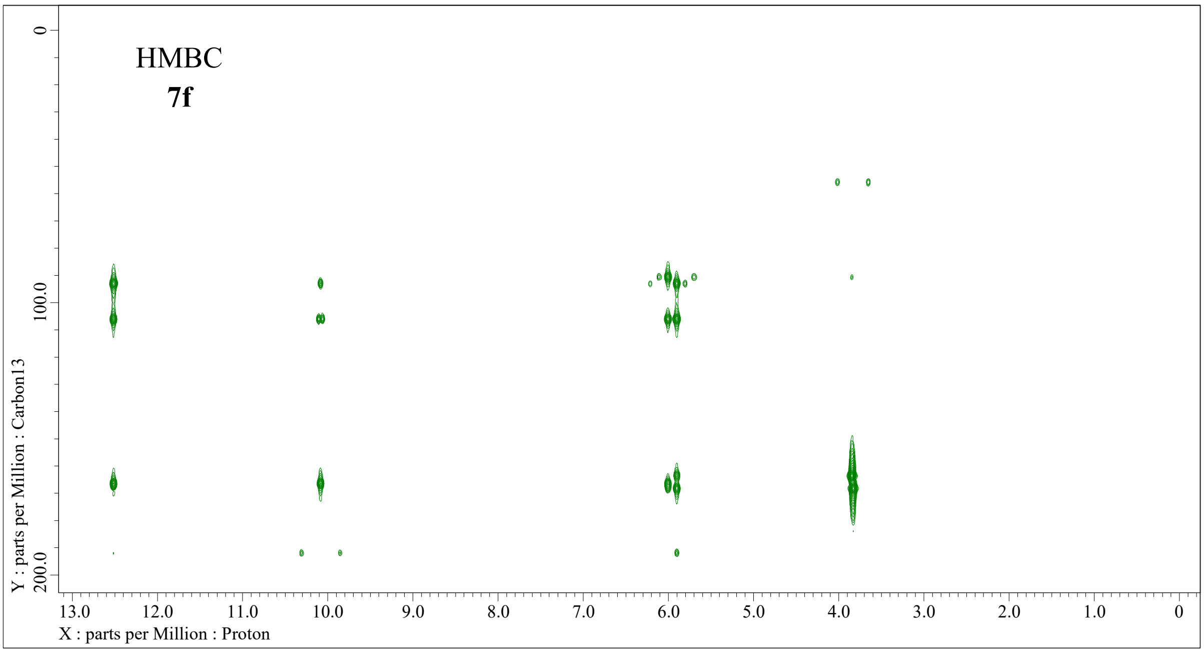

Figure S347. 2D-NMR (400 MHz, $CDCl_3$) HMBC experiment of 4,6-dimethoxy-salicylic aldehyde (**7f**).

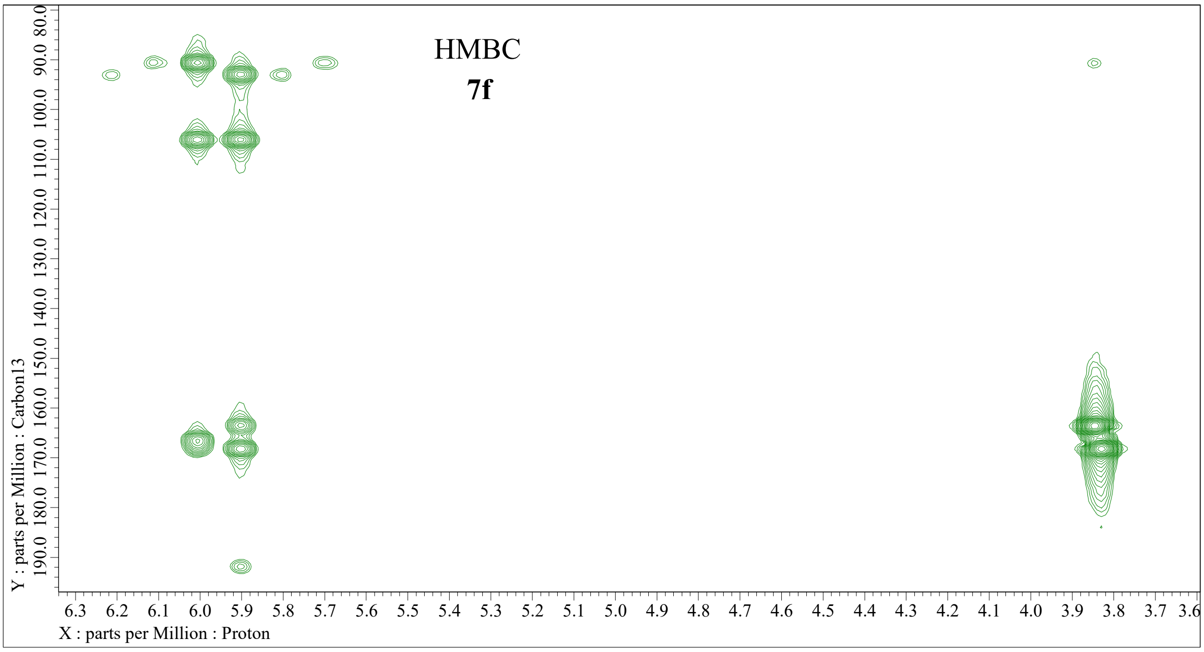

Figure S348. Expansion of 2D-NMR (400 MHz, $CDCl_3$) HMBC experiment of 4,6-dimethoxy-salicylic aldehyde (**7f**).

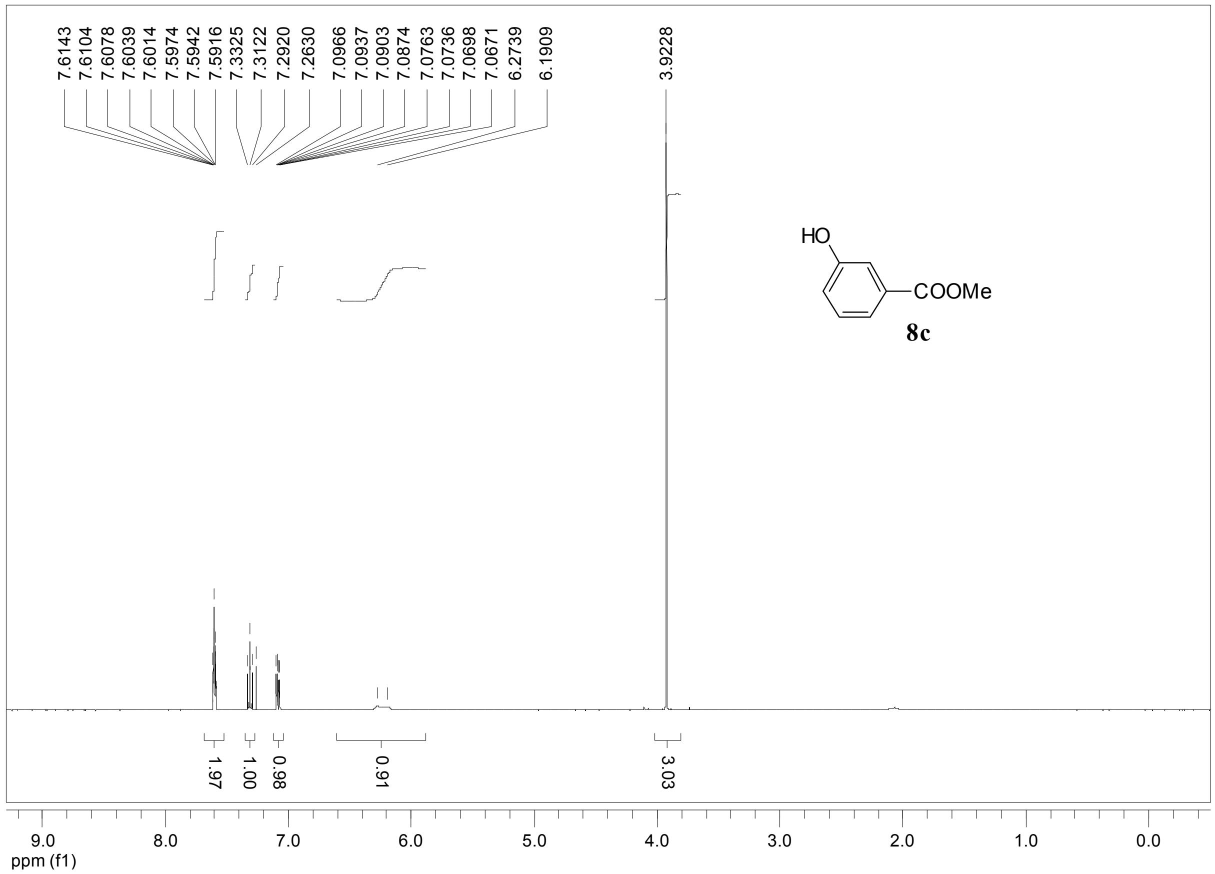

Figure S349. ^{1}H-NMR (400 MHz, $CDCl_3$) spectrum of methyl ester of 3-hydroxy-benzoic acid (**8c**).

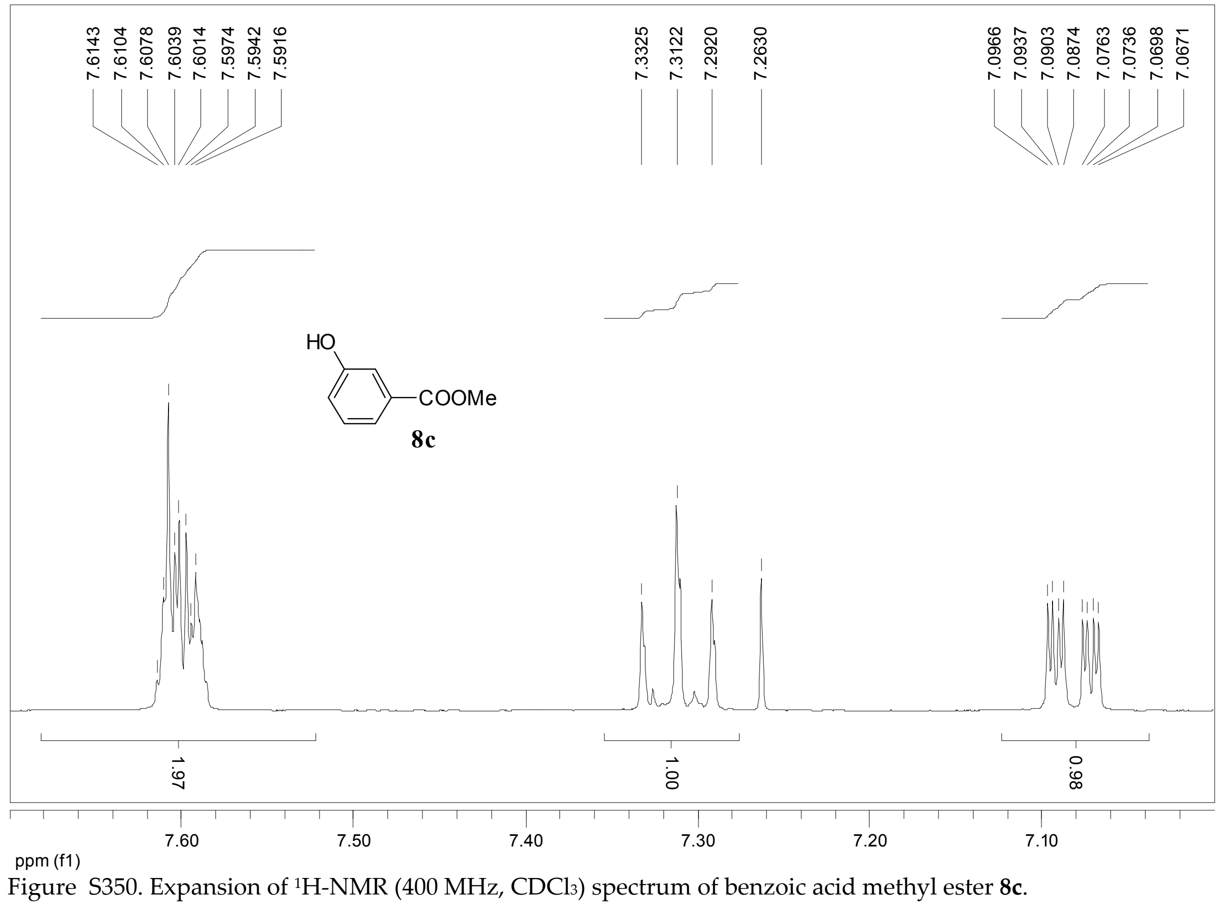

Figure S350. Expansion of ^{1}H-NMR (400 MHz, $CDCl_3$) spectrum of benzoic acid methyl ester **8c**.

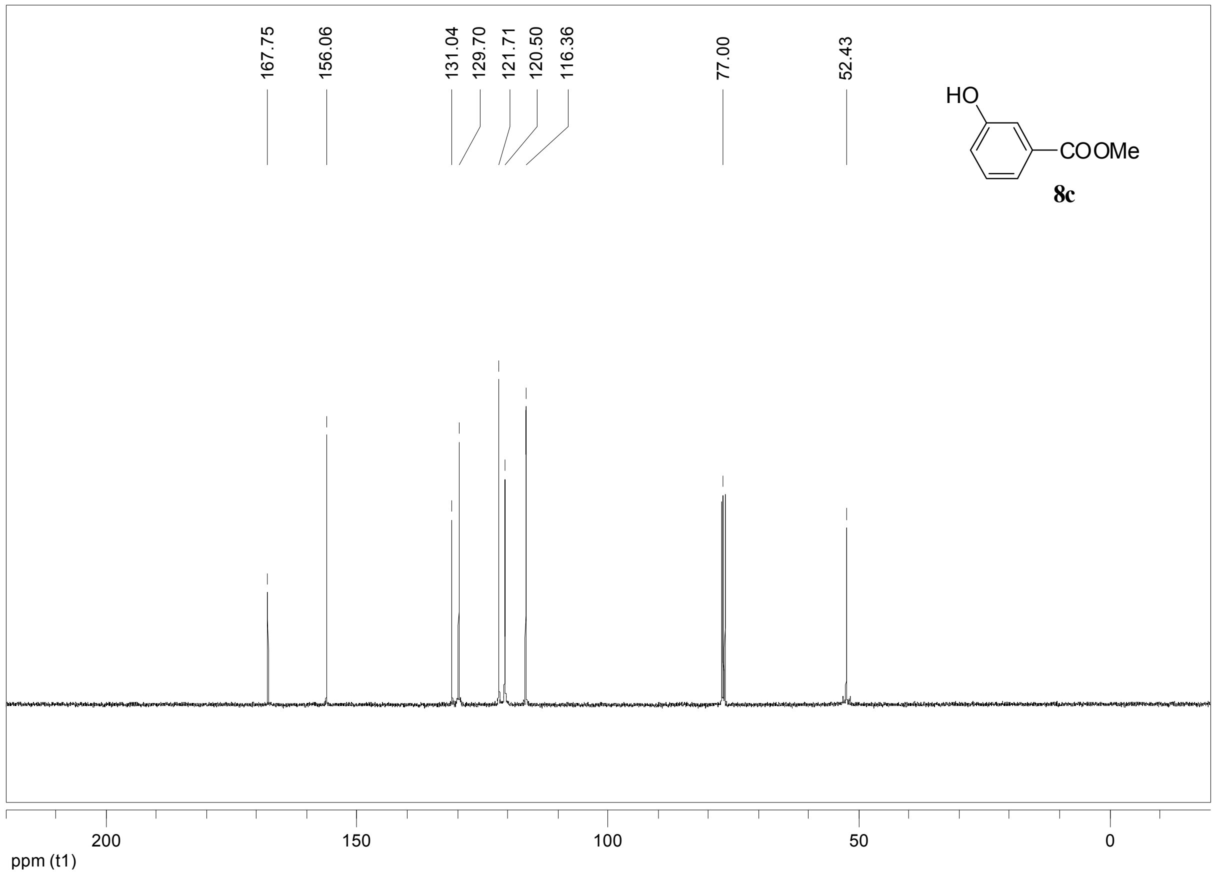

Figure S351. ^{13}C-NMR (100 MHz, $CDCl_3$) spectrum of 3-hydroxy-benzoic acid methyl ester (**8c**).

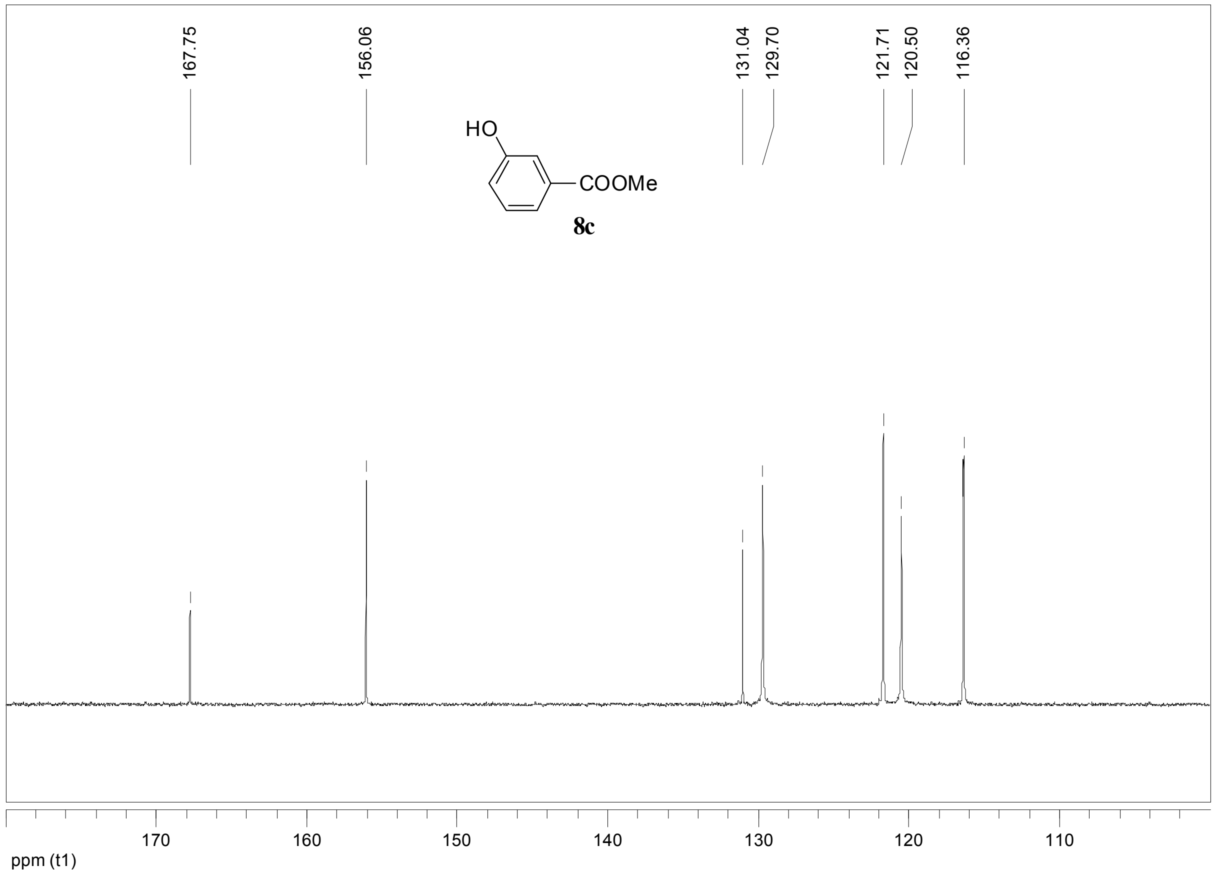

Figure S352. Expansion of ^{13}C-NMR (100 MHz, $CDCl_3$) spectrum of benzoic acid methyl ester **8c**.

Molecules **2020**, *25*, 1255; doi:10.3390/molecules25051255 www.mdpi.com/journal/molecules

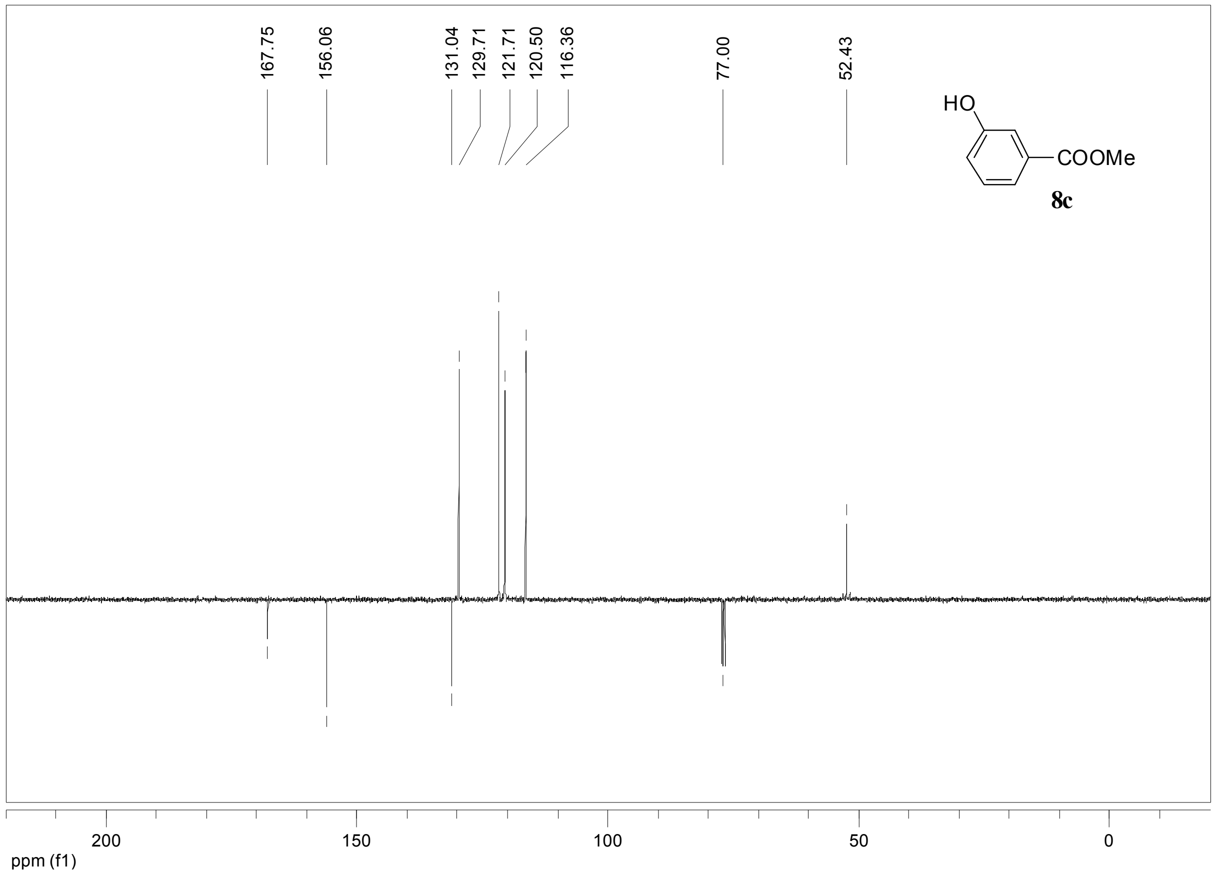

Figure S353. ATP (100 MHz, $CDCl_3$) experiment of 3-hydroxy-benzoic acid methyl ester (**8c**).

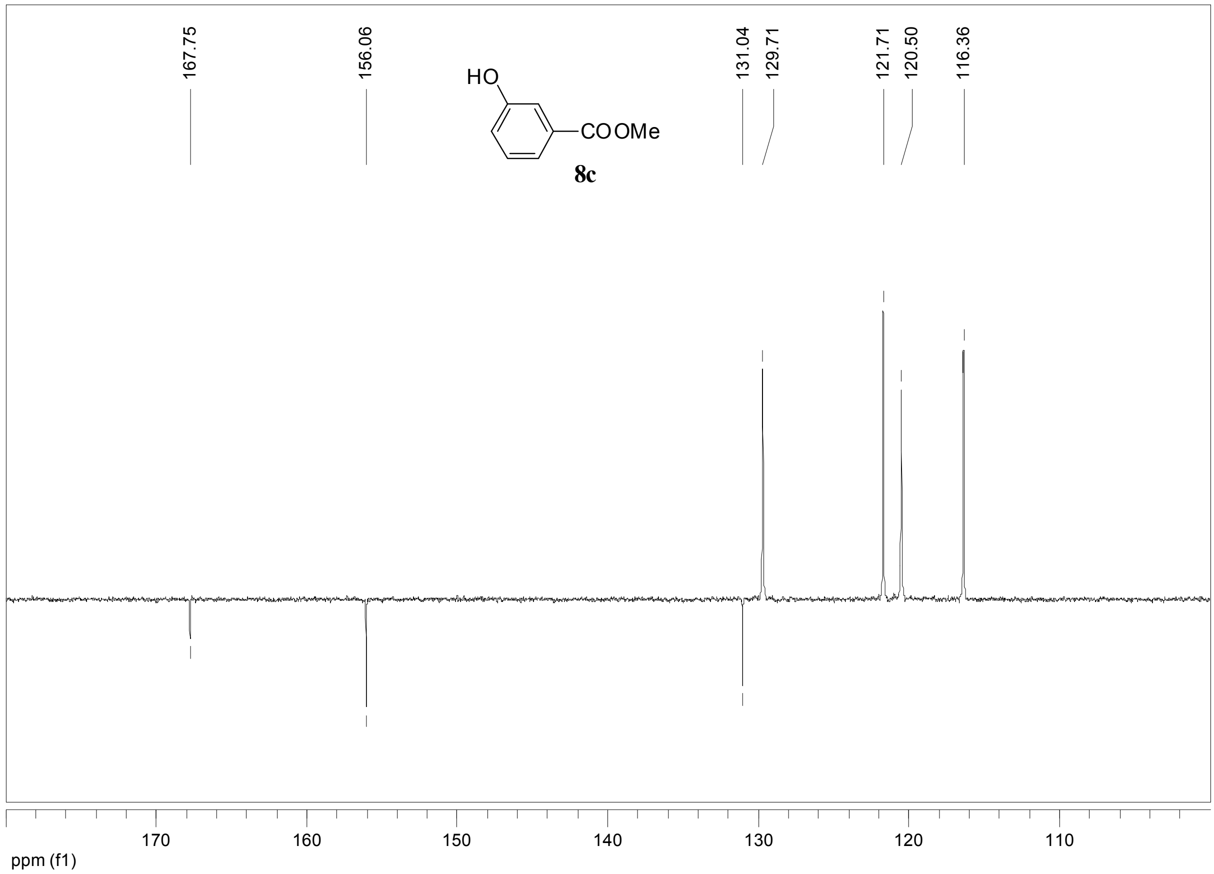

Figure S354. Expansion of ATP (100 MHz, $CDCl_3$) experiment of hydroxy-benzoic acid methyl ester **8c**.

6. Laccase stability in organic solvents

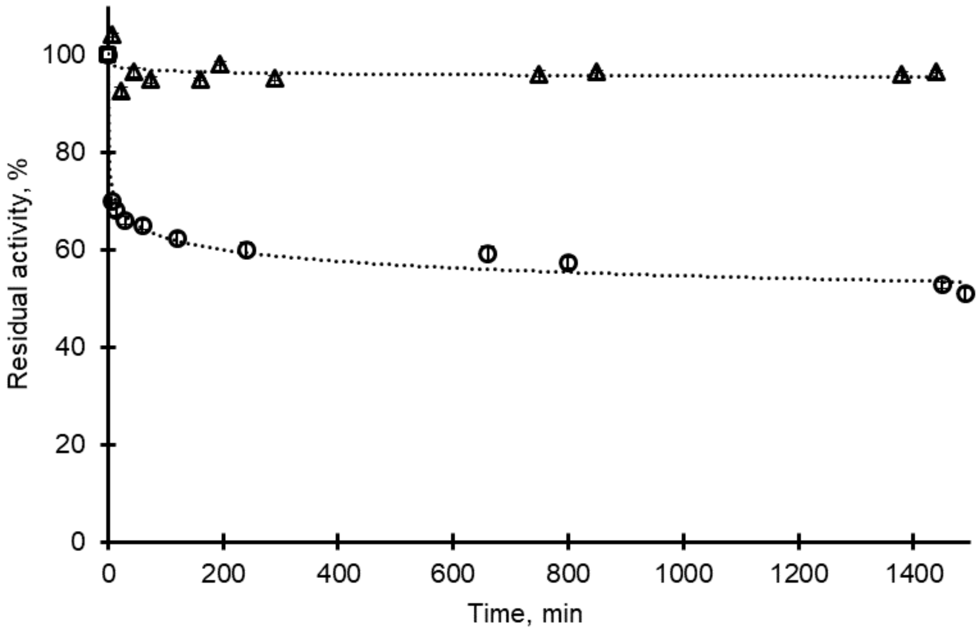

Figure S354. The stability of laccase from *T. versicolor* in buffer-organic co-solvent media. The concentration of the components in the incubated mixture was 6.25% (v/v) for methanol or DMSO and 43 nM for the enzyme. Triangles and circles correspond to experimental data performed with methanol and DMSO co-solvents, respectively. The stability of laccase in the particular solvent was calculated as a percentage of residual activity defined as the ratio of the enzyme activity at the time, t > 0 min to the enzyme activity at the time, t = 0 min.

1.

Molecules **2020**, *25*, 1255; doi:10.3390/molecules25051255 www.mdpi.com/journal/molecules

7. References

1. Said, S.B.; Elagamey, A.A.; Khadr, R.E. Facile oxidative conversion of aroyl hydrazones into 1,3,4-oxadiazoles. Egypt. J. Chem. **2003**, 46, 881–888.
2. Wang, Q.; Pan, Y.; Wang, J.; Peng, Q.; Luo, H.; Zheng, J. Synthesis and biological activities of substituted N′-benzoylhydrazone derivatives. African J. Biotechnol. **2011**, 10, 18013–18021. https://doi.org/10.5897/AJB10.2501.
3. Angelova, V.T.; Vassilev, N.G.; Nikolova-Mladenova, B.; Vitas, J.; Malbaša, R.; Momekov, G.; Djukic, M.; Saso, L. Antiproliferative and antioxidative effects of novel hydrazone derivatives bearing coumarin and chromene moiety. Med. Chem. Res. **2016**, 25, 2082–2092. https://doi.org/10.1007/s00044-016-1661-4.
4. Leigh, M.; Raines, D.J.; Castillo, C.E.; Duhme-Klair, A.K. Inhibition of xanthine oxidase by thiosemicarbazones, hydrazones and dithiocarbazates derived from hydroxy-substituted benzaldehydes. ChemMedChem **2011**, 6, 1107–1118. https://doi.org/10.1002/cmdc.201100054.
5. Bhat, A.K.; Bhamana, R.P.; Patel, M.R.; Bellare, R.A.; Deliwala, C.V. Chemotherapy of fungus infections. III. Alkyl or aryl thiosemicarbazones, acid hydrazones, and styryl aryl ketones of 5-bromo- and 5-nitrosalicylaldehydes. Indian J. Chem. **1972**, 10, 694–698.
6. Shalash, M.; Salhin, A.; Adnan, R.; Yeap, C.S.; Fun, H.-K. (E)-4-Hydroxy-N′-(4-hydroxy-3-methoxybenzylidene)benzohydrazide. Aca Crystallogr. Sect. E **2010**, E66, o3126–o3127. https://doi.org/10.1107/S1600536810045162.
7. Grimster, N.P.; Connelly, S.; Baranczak, A.; Dong, J.; Krasnova, L.B.; Sharpless, K.B.; Powers, E.T.; Wilson, I.A.; Kelly, J.W. Aromatic sulfonyl fluorides covalently kinetically stabilize transthyretin to prevent amyloidogenesis while affording a fluorescent conjugate. J. Am. Chem. Soc. **2013**, 135, 5656–5668. https://doi.org/10.1021/ja311729d.
8. Pereira, T.M.; Vitório, F.; Amaral, R.C.; Zanoni, K.P.S.; Murakami Iha, N.Y.; Kümmerle, A.E. Microwave-assisted synthesis and photophysical studies of novel fluorescent N-acylhydrazone and semicarbazone-7-OH-coumarin dyes. New J. Chem. **2016**, 40, 8846–8854. https://doi.org/10.1039/c6nj01532h.
9. Nisa, M. un; Munawar, M.A.; Iqbal, A.; Ahmed, A.; Ashraf, M.; Gardener, Q. tul A.A.; Khan, M.A. Synthesis of novel 5-(aroylhydrazinocarbonyl)escitalopram as cholinesterase inhibitors. Eur. J. Med. Chem. **2017**, 138, 396–406. https://doi.org/10.1016/j.ejmech.2017.06.036.
10. Ameryckx, A.; Thabault, L.; Pochet, L.; Leimanis, S.; Poupaert, J.H.; Wouters, J.; Joris, B.; Van Bambeke, F.; Frédérick, R. 1-(2-Hydroxybenzoyl)-thiosemicarbazides are promising antimicrobial agents targeting D-alanine-D-alanine ligase in bacteria. Eur. J. Med. Chem. **2018**, 159, 324–338. https://doi.org/10.1016/j.ejmech.2018.09.067.
11. Nomura, N.; Ishii, R.; Yamamoto, Y.; Kondo, T. Stereoselective ring-opening polymerization of a racemic lactide by using achiral salen- and homosalen-aluminum complexes. Chem. Eur. J. **2007**, 13, 4433–4451. https://doi.org/10.1002/chem.200601308.
12. Casiraghi, G.; Casnati, G.; Puglia, G.; Sartori, G.; Terenghi, G. Selective reaction between phenols and formaldehyde. A novel route to salicylaldehydes. Perkin Trans **1980**, 1980, 1862–1865.
13. Dixit, A.; Kumar, P.; Singh, S. Synthesis of chiral salalen ligands and their in-situ generated Cu-complexes for asymmetric Henry reaction. Chirality **2018**, 30, 1257–1268. https://doi.org/10.1002/chir.23019.
14. Bigi, F.; Conforti, M.L.; Maggi, R.; Sartori, G. Trialkylamine controlled phenol-for maldehyde reaction over clay catalysts: Selective and environmentally benign synthesis of salicylic aldehydes. Tetrahedron **2000**, 56, 2709–2712. https://doi.org/10.1016/S0040-4020(00)00171-X.
15. Zhang, S.; Wan, C.; Wang, Q.; Zhang, B.; Gao, L.; Zha, Z.; Wang, Z. Synthesis of chromones through LiOtBu/air-mediated oxidation and regioselective cyclization of o-hydroxyphenyl propargyl carbinols. European J. Org. Chem. **2013**, 2013, 2080–2083. https://doi.org/10.1002/ejoc.201201665.
16. Yadav, J.S.; Reddy, B.V.S.; Reddy, P.S.R.; Basak, A.K.; Narsaiah, A. V. Efficient halogenation of aromatic systems using N-halosuccinimides in ionic liquids. Adv. Synth. Catal. **2004**, 346, 77–82. https://doi.org/10.1002/adsc.200303229.
17. Haight, A.R.; Bailey, A.E.; Baker, W.S.; Cain, M.H.; Copp, R.R.; DeMattei, J.A.; Ford, K.L.; Henry, R.F.; Hsu, M.C.; Keyes, R.F.; King, S.A.; McLaughlin, M.A.; Melcher, L.M.; Nadler, W.E.; Oliver, P.A.; Parekh, S.I.; Patel, H.H.; Seif, L.S.; Staeger, M.A.; Wayne, G.S.; Wittenberger, S.J. Zhang, W. A scaleable synthesis of fiduxosin. Org. Process Res. Dev. **2004**, 8, 897–902. https://doi.org/10.1021/op049889k.
18. Ogawa, A.; Oohora, K.; Hayashi, T. Synthesis and characterization of meso-substituted cobalt tetradehydrocorrin and evaluation of its electrocatalytic behavior toward CO_2 reduction and H_2 evolution. Inorg. Chem. **2018**, 57, 14644–14652. https://doi.org/10.1021/acs.inorgchem.8b02333.

19. Bieszczad, B.; Barbasiewicz, M. The key role of the nonchelating conformation of the benzylidene ligand on the formation and initiation of Hoveyda-Grubbs metathesis catalysts. Chem. - A Eur. J. **2015**, 21, 10322–10325. https://doi.org/10.1002/chem.201501959.
20. Serra, S.; Alouane, A.; Le Saux, T.; Huvelle, S.; Plasson, R.; Schmidt, F.; Jullien, L.; Labruère, R. A chemically encoded timer for dual molecular delivery at tailored ranges and concentrations. Chem. Commun. **2018**, 54, 6396–6399. (See SI). https://doi.org/10.1039/c8cc03253j.
21. Gisch, N.; Balzarini, J.; Meier, C. Studies on enzyme-cleavable dialkoxymethyl-cycloSaligenyl-2′,3′-dideoxy-2′,3′-didehydrothymidine monophosphates. J. Med. Chem. **2008**, 51, 6752–6760. https://doi.org/10.1021/jm800853p.
22. Luehr, G.; Anik, S.T.; Peng, G.; Dotsenko, I.; Phiasivongsa, P.; Romanini, D. Pegylated carfilzomib compounds. WO 2017/205392 A1 2017.
23. (a) Casiraghi, G.; Casnati, G.; Puglia, G.; Sartori, G.; Terenghi, G. Selective reactions between phenols and formaldehyde. A novel route to salicylaldehydes. J. Chem. Soc., Perkin Trans 1 **1980**, 1980, 1862–1865. https://doi.org/10.1039/P19800001862; (b) Skarżewski, J.; Ostrycharz, E.; Siedlecka, R.; Zielińska-Błajet, M.; Pisarski, B. Substituted N-salicylidene β-aminoalcohols: Preparation and use as chiral ligands in enantioselective sulfoxidation and conjugate addition. J. Chem. Res. - Part S **2001**, 263–264. https://doi.org/10.3184/030823401103169847.
24. DiCiccio, A.M.; Longo, J.M.; Rodríguez-Calero, G.G.; Coates, G.W. Development of highly active and regioselective catalysts for the copolymerization of epoxides with cyclic anhydrides: An unanticipated effect of electronic variation. J. Am. Chem. Soc. **2016**, 138, 7107–7113. https://doi.org/10.1021/jacs.6b03113.
25. Rajput, J.D.; Bagul, S.D.; Hosamani, A.A.; Patil, M.M.; Bendre, R.S. Synthesis, characterizations, biological activities and docking studies of novel dihydroxy derivatives of natural phenolic monoterpenoids containing azomethine linkage. Res. Chem. Intermed. **2017**, 43, 5377–5393. https://doi.org/10.1007/s11164-017-2933-4.
26. Casiraghi, G.; Casnati, G.; Cornia, M.; Pochini, A.; Sartori, G.; Ungaro, R. Selective reactions using metal phenoxides. Part 2. Reaktions with aromatic alcohols. J. Chem. Soc. Perkin Trans. 1 **1978**, 1972–1999, 322–325.
27. Patel, J.J.; Laars, M.; Gan, W.; Board, J.; Kitching, M.O.; Snieckus, V. Directed remote lateral metalation: Highly substituted 2-naphthols and BINOLs by in situ generation of a directing group. Angew. Chemie - Int. Ed. **2018**, 57, 9425–9429. https://doi.org/10.1002/anie.201805203.
28. Kauch, M.; Hoppe, D. Efficient two-step synthesis of salicylaldehydes via directed ortho-lithiation of in situ N-silylated O-aryl N-isopropylcarbamates. Synthesis (Stuttg). **2006**, 1575–1577. https://doi.org/10.1055/s-2006-926461.
29. Ndikuryayo, F.; Kang, W.M.; Wu, F.X.; Yang, W.C.; Yang, G.F. Hydrophobicity-oriented drug design (HODD) of new human 4-hydroxyphenylpyruvate dioxygenase inhibitors. Eur. J. Med. Chem. **2019**, 166, 22–31. https://doi.org/10.1016/j.ejmech.2019.01.032.
30. Khusnutdinov, R.I.; Shchadneva, N.A.; Mayakova, Y.Y. Methylation of aliphatic and aromatic carboxylic acids with dimethyl carbonate under the influence of manganese and iron carbonyls. Russ. J. Gen. Chem. **2018**, 88, 15–19. https://doi.org/10.1134/S1070363218010036.
31. Dias, L.C.; Polo, E.C. Nhatrangin A: Total syntheses of the proposed structure and six of its diastereoisomers. J. Org. Chem. **2017**, 82, 4072–4112. https://doi.org/10.1021/acs.joc.6b03060.
